# Averting wheat blast by implementing a ‘wheat holiday’: In search of alternative crops in West Bengal, India

**DOI:** 10.1371/journal.pone.0211410

**Published:** 2019-02-20

**Authors:** Khondoker A. Mottaleb, Pawan K. Singh, Kai Sonder, Gideon Kruseman, Olaf Erenstein

**Affiliations:** 1 Socioeconomics Program, CIMMYT (International Maize and Wheat Improvement Center), Texcoco, México; 2 Global Wheat Program, CIMMYT, Texcoco, Mexico; 3 Geographical Information System Unit, Socioeconomics Program, CIMMYT, Texcoco, Mexico; ICAR-Indian Institute of Agricultural Biotechnology, INDIA

## Abstract

The emergence of wheat-blast in Bangladesh in the 2015–16 wheat (*Triticum aestivum* L.) crop threatens the food security of South Asia. A potential spread of the disease from Bangladesh to India could have devastating impacts on India’s overall food security as wheat is its second most important staple food crop. West Bengal state in eastern India shares a 2,217 km-long border with Bangladesh and has a similar agro-ecology, enhancing the prospects of the disease entering India via West Bengal. The present study explores the possibility of a ‘wheat holiday’ policy in the nine border districts of West Bengal. Under the policy, farmers in these districts would stop wheat cultivation for at least two years. The present scoping study assesses the potential economic feasibility of alternative crops to wheat. Of the ten crops considered, maize, gram (chickpea), *urad* (black gram), rapeseed and mustard, and potatoes are found to be potentially feasible alternative crops. Any crop substitution would need support to ease the transition including addressing the challenges related to the management of alternative crops, ensuring adequate crop combinations and value chain development. Still, as wheat is a major staple, there is some urgency to support further research on disease epidemiology and forecasting, as well as the development and dissemination of blast-resistant wheat varieties across South Asia.

## Introduction

The emergence and spread of virulent crop diseases and pests are continuously threatening global food security [1, 2]. Recent threats to global food security include wheat stripe rust (*Puccinia striiformis* f. sp. *tritici*) in Australia in 1978, and the invasion of a different aggressive race of the same pathogen in 2002 [3, 4]; the re-emergence of stem (or black) rust of wheat (*Puccinia graminis*) in Africa, the Middle East, the Arabian Peninsula, and parts of Asia [5]; Maize Lethal Necrosis [MLN] disease in Kenya [6]; and the spread of Fall Armyworm in Africa [7], and recently in India [8]. The recent emergence of wheat-blast disease in Bangladesh is another prominent addition in the series of such threats.

Wheat blast, caused by the fungus *Magnaporthe oryzae* pathotype *triticum* (MoT), was officially first reported in 1985 in the Brazilian state of Paraná [9]. By 1986, the disease had spread to northern and western Paraná, northwestern São Paulo State, and southern Mato Grosso do Sul, Brazil. Soon after, the wheat blast was detected in almost all major wheat-producing areas of Brazil [10, 11, 12, 13, 14, 15]. In 1996, the disease was reported for the first time outside of Brazil, in Bolivia’s most important wheat-production region, the Santa Cruz Department [16]. The disease reached Itapúa and Alto Paraná Departments of Paraguay in 2002 [17], and the province of Formosa in northeastern Argentina in 2007 [18]. In 2012, the blast was detected in an experimental station within the Buenos Aires Province, Argentina potentially threatening important wheat production areas of Argentina [19]. In 2016, a wheat blast outbreak was reported for the first time outside of South America, in Bangladesh, South Asia [20, 21, 22].

Wheat blast can result in a total wheat crop failure [23]. Fungicide application is not completely effective in controlling the disease. For example, in Parana State, Brazil, yield was reduced by 14–32% even after two applications of fungicides [23]. In February 2016, nearly 15,000 ha of wheat area in Bangladesh (3.4% of its total 436,817 ha of wheat) was affected by wheat blast, with wheat yield reductions ranging from 5–51% in the affected fields [20]. The disease emerged again in 2016–17, and the 2017–18 wheat seasons [24], making it now an established phenomenon in Bangladesh. In general, humid and warmer weather is the favorable condition for a wheat blast outbreak [25, 26]. The 2017–18 winter season in Bangladesh, however, was the coolest winter in 50 years [27]. Despite the less favorable season, the re-emergence indicates that MoT can survive and adjust to even harsh conditions, and the MoT in Bangladesh in particular, appears especially virulent [28].

The emergence of this disease in Bangladesh threatens the food security of more than a billion people in South Asia. Wheat consumption in South Asia has a long tradition in north-west India and Pakistan but has been increasing rapidly across South Asia [29, 30, 31, 32], making it the second major staple in Bangladesh and India, and the principal staple food in Pakistan. In 1961, the yearly per capita wheat consumption in Bangladesh, India, and Pakistan were 8.6kg, 27.9kg, and 90kg, respectively. In 2013, it had increased to 17.5kg in Bangladesh (+103%), 60.6kg in India (+117%) and 113.6kg (+26%) in Pakistan [33]. Currently, wheat supplies annual per capita dietary energy amount to 150kcal in Bangladesh, 517kcal in India and 903kcal in Pakistan [33]. Bangladesh is a net importer of wheat, India has recently emerged as a net exporter, and Pakistan is mostly self-sufficient in its wheat supply [33].

India shares a 4,096 km- long international border with Bangladesh, including 2,217 km in West Bengal, India alone [34]. Bangladesh’s border districts, such as Jashore, Jhenaidah, Chuadanga, and Rajshahi have now all reported wheat-blast [20, 21]. As MoT invasion can be seed borne [23], as well as airborne [35, 36], it points to a high possibility of the spread of MoT to India through West Bengal’s border districts that are agro-ecologically similar to Bangladesh, and possibly to relatively-warmer southern Pakistan and western India. A recent study [28] estimates that out of total 40.85 million ha of wheat land in Bangladesh, India, and Pakistan, 6.9 million ha (17.1%) is vulnerable to wheat blast ranging from Sindh, Pakistan to Sylhet Division, Bangladesh. A 5% loss in wheat production due to a potential outbreak of wheat blast in the three countries would reduce wheat production by 886 thousand metric ton worth of USD 132 million in a single year [28].

Such potential loss in wheat production in South Asia due to the possible spread of MoT from Bangladesh can have devastating consequences on the already-precarious food security situation in South Asia. For example, despite India’s tremendous success in alleviating abject poverty in the last two and half decades, still nearly 16% of the total Indian population is undernourished (281.4 million), and 44–50% of preschool-age children suffer from micronutrient deficiency [37]. In Bangladesh, currently 36% of the under five-year aged children are stunted, and 31% of the young married women are undernourished [38]. A reduction in wheat production due to a potential spread of MoT from Bangladesh can further worsen the overall food security situation of India and South Asia as a whole.

**Fig 1:**
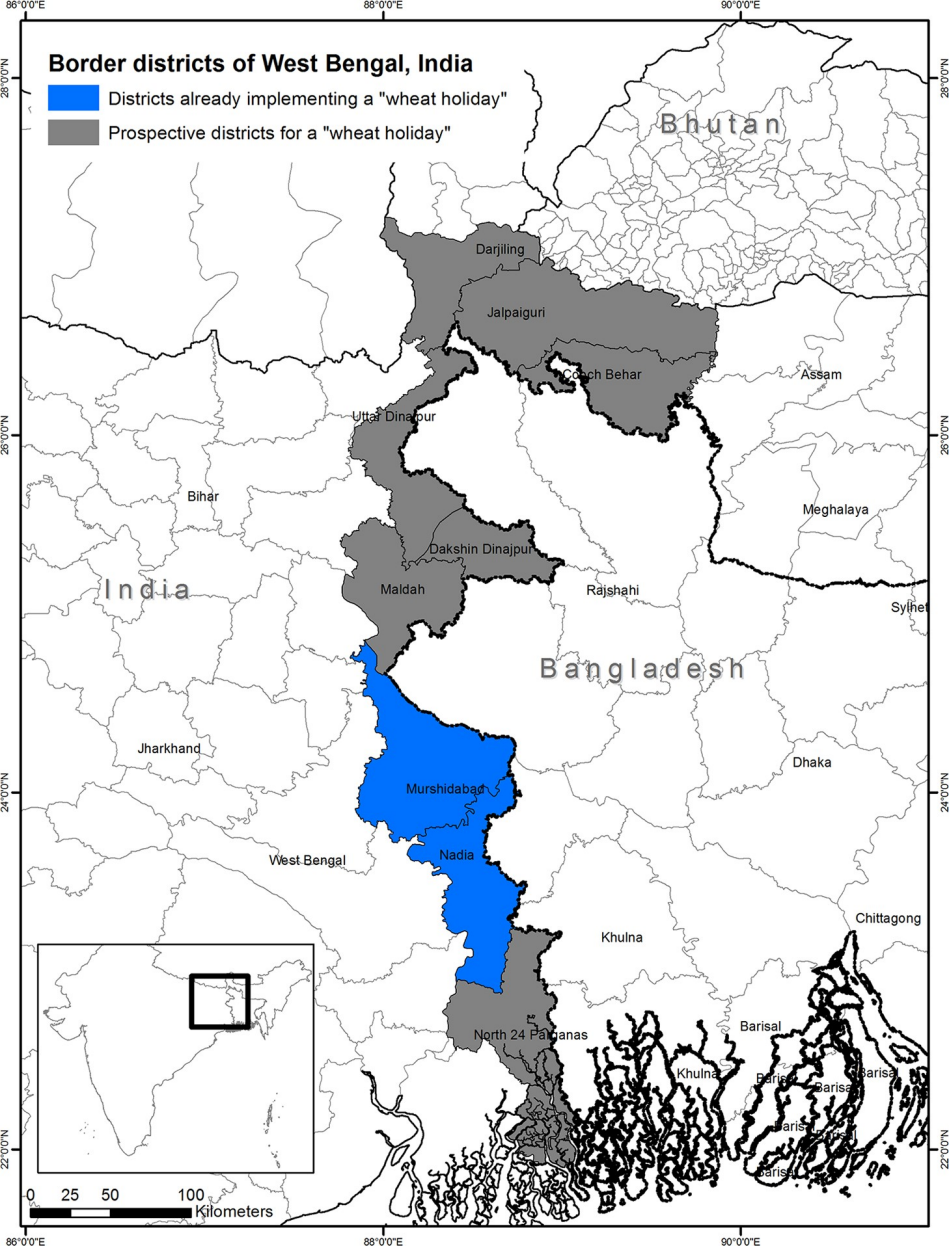
Location of the nine border districts of West Bengal, India [grey-blue]. Sources: Authors. The two districts where wheat cultivation is already banned (blue colored districts) is based on [34].

To avoid any such disaster, as a preventive control, the Indian Council for Agricultural Research (ICAR) has implemented a temporary ‘wheat holiday’ in Murshidabad and Nadia districts of West Bengal for three years (blue colored districts in Fig 1), suggesting legumes and oilseeds in place of wheat [34]. Also, wheat cultivation has been banned in the border districts of West Bengal, India, within five kilometers of the Bangladesh border [34]. For effective implementation of a ‘wheat-holiday’ policy–i.e., banning wheat cultivation for a few years in targeted areas–suggestions on economically-feasible alternative crops to wheat must be supplied to farmers to ensure the food security and livelihoods of the resource-poor farmers. The present scoping study is the first attempt to identify such substitute crops for wheat in West Bengal, India.

There are nine districts of West Bengal that border with Bangladesh: Darjeeling, Jalpaiguri, North 24 Parganas, Cooch Behar, North Dinajpur, South Dinajpur, Maldah, Murshidabad, and Nadia (Fig 1). Assuming a wheat holiday policy in the nine border districts of West Bengal, the present study examines the economic feasibility of ten potential substitute crops (Table 1). The present scoping study applies an *ex-ante* framework, in which it is assumed that the current wheat area in the nine border districts of West Bengal will be replaced by the sampled alternative crops (Table 1). It examines the economic feasibility of the sampled crops based on available secondary data for the production costs and returns per hectare.

**Table 1:** Cropping season for major *rabi* (winter) crops in West Bengal, India.

Sampled *Rabi* crop	Sowing/planting month [39]	Harvesting month [40]
Wheat	November-December	March-April
Paddy (non-monsoon, *Oryza sativa*)	November-December	April-May
Maize (*Zea mays*)	November	March
Lentil (*Lens culinaris*)	October-November	March-April
Gram/chick pea (*Cicer arietinum)*	November-December	March
*Urad*/black gram *(Vigna mungo)*	February-April	May-June
*Khesari/*grass pea (*Lathyrus sativus*)	October-November	March-April
Peas & beans (*Pisum sativum & Phaseolus vulgaris)*	October-November	March-April
Rapeseed & mustard (*Brassica napus & Brassica juncea*)	October	February-March
Linseed (*Linum usitatissimum)*	October-November	March-April
Potato [41] (*Solanum tuberosum)*	October-November	March

The rest of the study is organized as follows: the next section includes the context of wheat in India and West Bengal and explains the scoping *ex-ante* assessment process. The following section presents the economic viability of the alternative crops, and the last section presents the conclusions and policy implications.

## Materials and methods: Context and ex-ante estimation procedure

### State of wheat production and consumption in India

**Fig 2:**
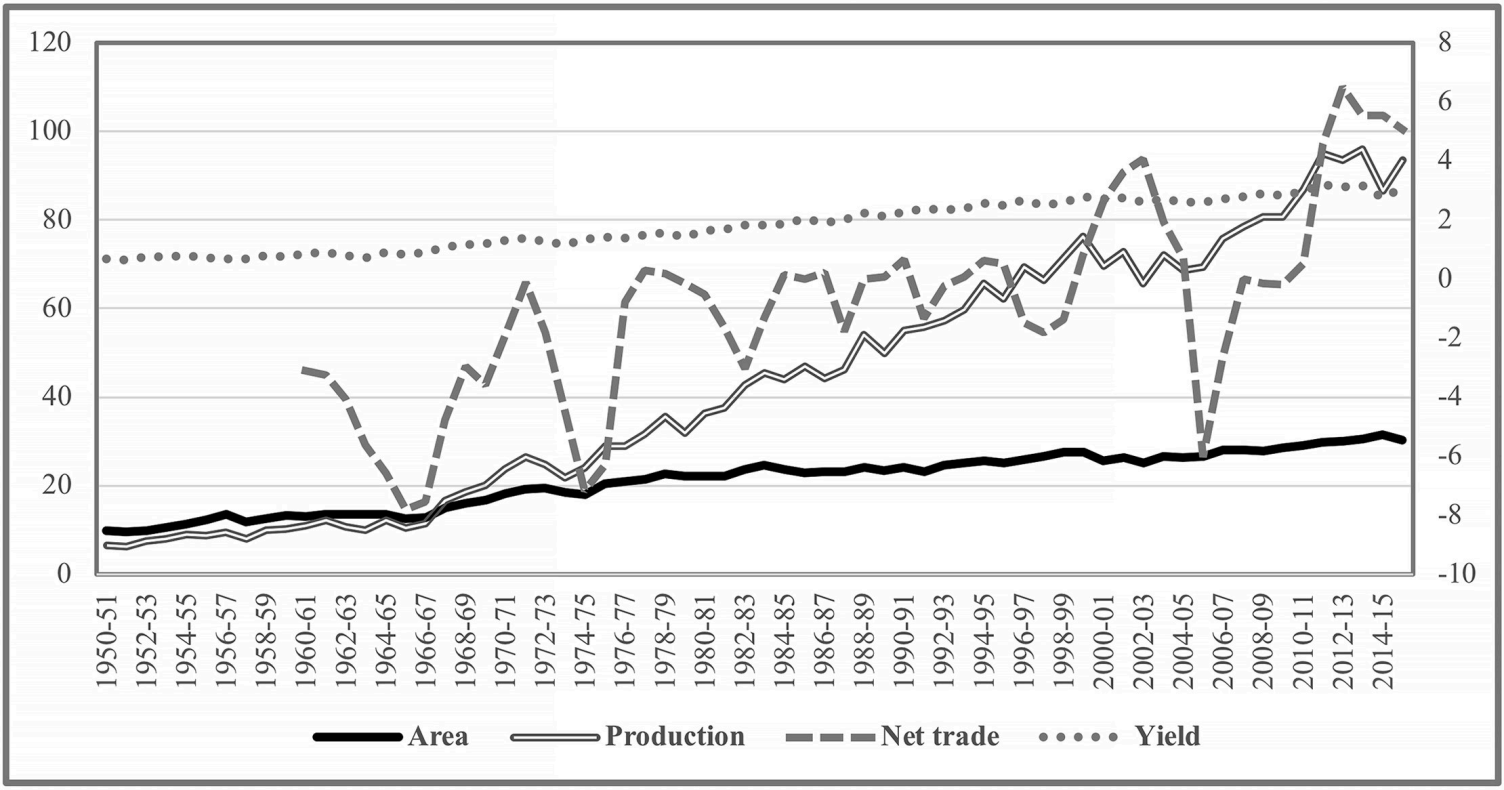
**Wheat indicators for India, 1960–2018 [area (million ha) and production (MMT, both left axis] and trade (MMT) and yield (ton/ha, both right axis)]**.

In India, wheat is the second major crop and staple food after rice regarding land allocation, production, and consumption. In 1950–51, the total area under wheat was 9.75 million ha, with a yield of 0.66 ton per ha; total production was 6.46 million metric tons (MMT), and 34% of the area irrigated [39]. In 1967–68, wheat yield in India for the first time exceeds more than a ton per ha (1.10 t/ha), with a total production of 16.5MMT from nearly 15 million ha of land of which 43.4% was irrigated. Despite the dramatic increase in yields, up to 1993, India was a net wheat importing country, with sporadic wheat exports [33]. In 2015–16, with 30.23 million ha of wheat with a yield of 3.09 ton/ha, and 93.5MMT production [39], India is the second largest wheat-producing country in the world after China. Fig 2 presents historical information on India’s wheat area, production and trade that is developed based on USDA [42]. India’s production is 12.5% of the total wheat in the world. At present, nearly 94% of the total wheat area in India is irrigated [39] and, since 2000–01, the country has emerged as a net exporting country [33]. India exported an average of 3.9MMT of wheat in the triennium ending 2013 (TE2013), worth USD 1.1 billion yearly. Uttar Pradesh (28.7% domestic production in 2015–16), Madhya Pradesh (18.9%), Punjab (17.2%), Haryana (12.1%) and Rajasthan (10.6%) are the major wheat producing states of India [39].

In pace with the increased production, the yearly per capita wheat consumption in India has also increased over the years [33, 43, 44, 45, 46, 47, 48, 49]. In 1961, the yearly per capita wheat consumption in India was less than 28kg. With the per capita annual growth in consumption at 3.12% from 1961 to 1970, it increased to 36.2kg by 1970. Finally, with the annual consumption growth rate of 1.91% from 1970 to 2013, the yearly per capita wheat consumption reached 60.6kg by 2013 [33]. Considering the per capita consumption growth in relation to growth in population and income, it is projected that in India [44], wheat consumption might increase by 4% per year in the future. Nagarajan [49], on the other hand, stressed that India needs to produce 109MMT of wheat by 2020 to maintain the self-sufficiency status in wheat supply. Note that in 2015–16, India produced 93.5MMT of wheat from 30.2 million ha of land. Considering the importance of wheat, a potential reduction in wheat production due to an intrusion of MoT from Bangladesh to India can have severe impacts, primarily on the food security of India’s 1.32 billion population.

#### Wheat in West Bengal, India—Base scenario

Located in the lower Gangetic Plains, rice is the dominant crop and staple of West Bengal similar to Bangladesh [29], with the rice-wheat cropping system being common in the border areas [50] with rice prevailing during the monsoon season and wheat in the cooler winter/*rabi* season. According to ICAR-CRIDA [51], total cultivable land in West Bengal is 5.65 million ha, and with three rice seasons, 53% of the land is allocated to rice only [52]. The wheat area (triennium average ending 2015–16) in West Bengal was 340 thousand ha, which was 1.1% of the total wheat area of India (30.2 million ha), and the production was 960 thousand tons, which was 1% of the total wheat produced in India (Table 2).

**Table 2:** Reference indicators of selected crops in India, West Bengal, and West Bengal’s sampled nine border districts (triennium ending [TE] 2015–16).

Sampled crop	Area (‘000 ha)^3^			Production (‘000 tonnes)^3^			Yield (ton/ha) ^3^			Price (USD/ton)^2^	Product value (Million USD)
	India^1^	West Bengal^1^	Sampled districts^3^	India^1^	West Bengal^1^	Sampled districts^3^	India^1^	West Bengal^1^	Sampled districts^3^	West Bengal	Sampled districts
Wheat	30,230	340	287	93,500	960	755	3.09	2.83	2.63	257.3	194.2
Paddy rice	43,390	5,460	1,355	104,320	15,750	3762	2.4	2.88	2.78	232.3	873.9
Maize	8,690	160	49.2	21,810	720	270	2.51	4.62	5.49	168.5^5^	90.8
Lentil	1,470	70	61.9	1,040	60.0	50.6	0.71	0.96	0.82	532.9	26.9
Gram	8,350	30.0	18.3	7,170	30.8	17.5	0.86	1.18	0.96	839.1	14.7
*Urad*^*2*^	4,019	75	9.3	1,868	53	8.8	0.57	0.72	0.94	708.3^6^	6.2
*Khesari*	394	33.0	20.7	282	41.1	17.8	0.72	1.24	0.86	532.9^7^	9.5
Peas and beans^3^	741	14.1	11.3	683	16.8	11.8	0.92	1.19	1.04	433.5^4^	5.1
Rapeseed and mustard	5,760	460	349	6,820	500	388	1.18	1.09	1.11	631.4	244.8
Linseed^2^	298	6.0	3.9	153	2.0	1.5	0.54	0.40	0.39	465.9^8^	0.72
Potato	2,134	412	132	43,770	13,908	3,618	20.5	29.2	27.4	197.3	713.8

Sources

^1^GoI [39]

^2^GoI [56]

^3^MoAFW [53]

^4^FAOSTAT [33].

Notes

^5^Average of winter and summer maize prices of Bihar

^6^National prices

^7^Lentil price is used as the proxy price of *Khesari*

^8^Price of linseed in Bihar.

The nine border districts of West Bengal are the major wheat-producing districts among its 23 districts. Altogether, these districts allocated 287 thousand ha of land to wheat, which was nearly 85% of the total land allocated to wheat in West Bengal in TE2015-16 (Table 2). The border district Murshidabad is the single largest wheat-producing district in West Bengal, and it allocated 117.5 thousand ha of land to wheat in 2015–16, which was 34.6% of the total wheat area of West Bengal. The other three border districts: Maldah, North Dinajpur and Nadia allocated 50.6, 46.7 and 37.2 thousand ha to wheat in 2015–16, respectively, which were 14.9%, 13.7% and 10.9% of the total wheat area of West Bengal [53]. These four border districts jointly comprise more than 74% of the total wheat area in West Bengal. In addition to wheat, land allocation, production and yield of the other ten sampled crops are reported (Table 2).

Finally, it is important to note the prevailing food habits and livelihoods in West Bengal, where rice is the major cereal consumed by the households. For example, in 2011–12, in rural India, the monthly average per capita cereal consumption was 11.2kg in which 55% was rice, 39% wheat, and 6% was other cereal. In West Bengal, however, a member of a rural household, on average consumed 12kg of cereals, in which 88% was rice and only 12% was wheat [54]. Rice is also prominent in the cereal consumption of urban households in West Bengal [54]. Thus, whereas wheat is prominent in India’s food security, wheat plays a less prominent role in West Bengal’s food security, and West Bengal only provides a marginal share of the nation’s wheat production. A replacement of wheat by other alternative crops in West Bengal may thus have somewhat less prominent negative effects on the state’s cereal intake as well as its rural livelihoods compared to India ‘s other wheat dependent states.

### *Ex ante* estimation procedure

A possible control measure to wheat blast can be to introduce a ‘wheat holiday’ policy in the border districts: completely suspending wheat production for at least two years as MoT can survive on seeds for up to 22 months [55]. In search of economically-feasible crop alternatives to wheat, the present study estimates the gross and net returns of ten current *rabi* (dry, cool winter season) crops to examine the economic viability of each crop as a replacement for wheat during the ‘wheat holiday’ period. As alternative crops, we consider ten crops that are all currently grown in the *rabi* season in West Bengal (Table 1).

Table 2 presents the base scenario of land allocation, production, and yield of the sampled crops, for India, West Bengal and the sampled nine border districts. The table also includes prices of each crop (USD/ton) for 2015–16, based on GoI [56], FAOSTAT [33] or relevant proxy prices.

To simulate alternative scenarios, we assume a complete replacement of the total current wheat area (TE2015-16) of 287 thousand ha (Table 2) in the nine border districts by the sampled alternative crops. For each alternative crop scenario, the current average yield (t/ha, Table 2) is multiplied with the current wheat area at the sampled district level to calculate the expected gross revenue from each sampled border district. Thus, the expected gross revenue from each crop in the alternative scenario, assuming a complete replacement of the current wheat area by an alternative crop (c), is calculated as follows:

$$GR_{c}=\sum_{d=1}^{9}\left(WA_{TA}\;x\;{Cyield}_{CTA}\;x\;P_{c}\right)$$ (1)

where *GR*_*c*_ is the gross revenue of an alternative crop *c* (= 1–10), *WA*_*TA*_ is the land area under wheat (ha) in district *d* (*d = 1——9*) for TE2015-16, *Cyield*_*TA*_ is the district-level yield of the alternate crop *c* (t/ha), and *Pc* is the domestic market price in India of the alternate crop *c* (United States Dollar, USD/ton). The average wholesale price/ton of sampled crops were considered and converted into US dollars using the prevailing exchange rate (June, 2018 USD 1 = Indian Rupee Rs. 68.26).

In the *ex-ante* estimation process, it is thus assumed that (1) the alternative crops can completely replace the current wheat area in the nine sampled border districts; and (2) that the yields and returns of the alternative crops on the entire substituted wheat area in each district would be comparable to those currently achieved in the same district. Based on these assumptions, the expected gross margin for each alternative crop *c* is calculated as follows:

$${GM}_{c}={GR}_{c}-({CC}_{c}\;x\;{WA}_{TA})$$ (2)

where *CC*_*c*_ is the production cost of crop *c* (USD/ha) in West Bengal and WA_TA_ is the current wheat area in the sampled nine districts. For production costs/ha, we relied on GoI [39, 57], which provide reference state-level production costs. The production cost considered in this study are categorized as all input costs (A2) + total labor costs [39, 57]: i.e., costs calculated based on all actual expenses in cash and in kind, including land rent and labor costs including family labor imputed based on the existing wage rate (Table 3). Potatoes have the highest production costs (USD 1729/ha), followed by paddy (USD 848/ha) and wheat (USD 567/ha) (Table 3). In contrast, the lowest production costs are reported for *urad* (USD 214/ha).

**Table 3:** Reference production costs (USD/ha) of the sampled crops (2015, unless otherwise indicated).

	Production costs (USD/ha)			
Wheat and alternative crops	All input costs (A2) + imputed value of family labor	Total labor cost	Family labor cost	Reference State
Wheat	567.2	253.6	92.6	West Bengal
Paddy	848.3	523.6	266.0	West Bengal
Maize	460.6	204.3	130.3	Bihar
Lentil	412.5	200.2	84.7	West Bengal
Gram	332.1	130.8	54.5	Bihar
*Urad*	214.2	133.8	124.9	Odisha
*Khesari*	412.5	200.2	84.7	West Bengal (lentil)
Peas & beans	483.3	174.4	—	Uttar Pradesh (2011–12)
Rapeseed & mustard	515.3	297.6	147.2	West Bengal
Linseed	408.9	255.8	120.9	West Bengal (sesame)
Potato	1729.6	614.7	294.7	West Bengal

Sources: GoI [39; 57]. Note: 2015 exchange rate: USD 1 = Rs. 65.7

The expected net margin of each alternate crop in the substitution scenario net of wheat is calculated as follows:

$${ENM}_{c}={GM}_{c}-{GM}_{w}$$ (3)

where, *ENM*_*c*_ is the expected net margin from a sampled crop *c*, *GM*_*c*_ is the gross margin of the sampled crop *c*, and *GM*_*w*_ is the wheat gross margin.

Labor cost is a component in our net margin estimation process of each sampled crop (Table 3). Still, it is useful to specifically compare the labor costs for wheat and the alternative crops. In India, labor comprises 19% of the total crop production costs [58]. Furthermore, the nominal agricultural labor wage rate increased by 20% per annum during 2009–10 to 2012–13 [58]. The total labor cost for wheat was USD 254/ha, in which USD 92.6 was the imputed costs of family labor (Table 3). The lowest labor costs were reported in the case of the gram (USD 131/ha), followed by *urad* (USD134/ha); and peas and beans (USD 174/ha). In contrast, the highest labor costs are reported for potato (USD 615 /ha) followed by paddy (USD 524 /ha). In suggesting alternative crops to wheat, in addition to the economic feasibility of the crops, we also need to consider their labor requirement considering wage rates as well as labor calendars.

Note that the present study only intends to provide an indicative scoping of alternative crops based on available secondary data. Based on the initial scope, more rigorous and grounded empirical data are needed to support the transition to the more promising alternatives. The scoping study also does not consider the eventual price and market effects due to the changes in the production and supply associated with the crop transition. The assumption of constant prices is largely warranted as the Indian agricultural sector is integrated into the global commodity markets. The country is a net exporter of rice, wheat, maize, rapeseed, mustard, linseed and potatoes; and imports lentils, and gram from the international market [33]. In an open economy with tradable commodities, the price effects from crop substitution are not expected to play critical roles in land and other input allocation. In addition, the study only considers the reallocation of wheat land in the nine border districts of West Bengal, which is a relatively minor share of India’s agricultural land. The present study also did not consider the financial and the agronomic learning costs of the farming practices that farmers need to incur to switch from wheat to other crops, nor the market and value chain costs associated with increased production and potential reversal of trade-flows (e.g., net importer to net exporter).

### Results and discussion: Economic feasibility of alternative crops to wheat

#### Rice

Table 4 presents information on production, trade and trading partners and the global ranks of India in terms of production share of the sampled commodities. Note that the information on linseed and *urad* production is taken from GoI [59], and the information on the major trading partners is collected from UNCOMTRADE [60]. With a production of 104MMT from 43.4 million ha of land of which 60% is irrigated [39], India is the second largest rice producing county in the world after China (Tables 2 and 4). In terms of per capita yearly consumption, rice is the principal staple food in India. Currently, the yearly per capita rice consumption in India is 69.5kg [33]. The top three rice-producing states of India are West Bengal that supplies more than 15% of total rice in India, Uttar Pradesh (12%), and Punjab that supplies more than 11% of the total rice (Table 4). Tamil Nadu, Andhra Pradesh, and Bihar are other major rice-producing states [39]. India produces more than 21% of the world’s rice and considering the international trade; the country is a net rice-exporting country (Table 4). The country exported yearly 10.6 MMT tons of rice worth of USD 6.1 billion (TE2016). The major importers of Indian rice are Nepal, Philippines, Burkina Faso, Vietnam and Bangladesh (Table 4). Broadly, rice in India can be winter (*rabi)* and summer (*kharif*) season rice. In our scenario analysis, we only consider a replacement of current wheat areas in nine border districts in West Bengal by *rabi* rice, which is wheat’s competing crop.

**Table 4:** Production and trade characteristics of selected crops in India and globally.

	World production 2016 (million ton)^1^	India’s rank and share (%)^1^	Major producing states in India^2^	Major producing country and share (%)^1^	Net trade ‘000 ton (million USD), TE2016^3^	Major trading partners
Wheat	749.47	2^nd^ (12.5)	Uttar Pradesh (28.7%), Madhya Pradesh (18.9%), Punjab (17.2%)	China 1^st^ (17.6)	770.4 (0.25)	Nepal, United Arab Emirates, Bangladesh
Paddy all	742.55	2^nd^ (21.4)	West Bengal (15.1%), Uttar Pradesh (12.0%), Punjab (11.3)	China 1^st^ (28.4)	10,637.2 (6532.5)	Nepal, Philippines, Burkina Faso, Vietnam, Bangladesh
Maize	1060.27	7^th^ (2.5)	Karnataka (15.0%), Madhya Pradesh (11.8%), Bihar (11.0%)	USA 1^st^ (36.3)	1584.1 (375.9)	Nepal, Bangladesh, Sri Lanka,
Rapeseed and mustard	69.55	3^rd^ (9.8)	Rajasthan (47.9%), Haryana (11.8%), Madhya Pradesh (10.3%)	Canada 1^st^ (26.8)	18.5 (11.7)	Nepal, United States of America, United Kingdom
Lentils	4.56	2^nd^ (16.3)	Madhya Pradesh (40.2%), Uttar Pradesh (22.7%), Bihar (18.7%)	Canada 1^st^ (50.0)	-871.5 -(662.9)	Canada, USA, Australia,
Gram (chick pea)	12.09	1^st^ (64.7)	Madhya Pradesh (45.5%), Karnataka (12.5%), Rajasthan (11.2%)	Australia 2^nd^ (7.2)	-475 (-273.6)	Australia, Russia, Tanzania
*Urad*	-	-	Madhya Pradesh (23.4%), Uttar Pradesh (16.3%), Andhra Pradesh and Telangana (16.1%)	-		
*Khesari*^4^	-	-	Chhattisgarh (61.1%), West Bengal (20.8%), Bihar (18.1%)	-		
Peas and beans^4^	108.24	2^nd^ (19.5)	Uttar Pradesh (70.8%), Rajasthan (8.2%); Assam (6.3%)	China 1^st^ (30.7)	-3159.7 (-1739.7)	China, USA, Thailand
Linseed	3.29	6^th^ (3.8)	Madhya Pradesh (39.3%), Bihar (10.6%), Uttar Pradesh (9.2%)	Russian Federation 1^st^ (20.5)	9.7 (10.5)	Germany, Netherlands, Canada
Potato	376.88	2^nd^ (11.6)	Uttar Pradesh, (31.0%), West Bengal (25.1%), Bihar (13.2%)	China 1^st^ (26.3)	242.7 (74.8)	Nepal, Sri Lanka, Oman, Mauritius,

Sources

^1^FAOSTAT [33]

^2^GoI [39]

^3^Trienninum ending 2016 calculated from FAOSTAT [33]

^4^MoAFW [53].

Considering the production cost of wheat in West Bengal USD 547/ha, and national price USD 257/t (Table 2), the present study calculated the gross return from wheat cultivation in 287 thousand ha of land in nine districts worth of USD 31.6 million (Table 5). Assuming a complete replacement of the current wheat area with winter rice with the average yield of 2.78 t/ha (Table 2), the simulation results show that, the total rice production would increase to nearly 842 thousand MT. However, considering the relatively high production costs compared to the other winter crops, which is USD 848/ha (Table 2), the overall returns to winter rice do not look favorable compared to the returns of wheat. This is under the current scenario and prices (*ceteris paribus*) and reflects the relatively high production cost of winter rice. The simulation exercise in Table 5 shows that given lower gross margins of rice compared to wheat, the substitution would imply a net loss worth USD 79 million (Table 5). Besides, winter rice is more intensely irrigated, and the replacement of the current wheat area in the border districts with rice could aggravate underground water extraction and ecological considerations. Overall, replacing wheat with winter rice to implement a ‘wheat-holiday’ policy in nine border districts of West Bengal may not be a feasible option.

**Table 5:** Simulated crop production economics for selected crops for the area corresponding to the current wheat area in sampled nine border districts of West Bengal.

Wheat and alternative crops	Production (‘000, tons)	Revenue (million USD)	Total production cost (million USD)	Gross margin (million USD)	Difference in gross margin relative to wheat (million USD)
Wheat	754.7	194.2	162.6	31.6	-
Paddy	841.6	195.5	243.3	-47.6	-79.3
Maize	1,163.8	196.1	132.1	64.0	32.5
Lentil	227.7	121.3	118.3	3.05	-28.5
Gram	276.0	231.6	95.2	136.4	104.8
*Urad*	207.7	147.1	61.4	85.7	54.1
*Khesari*	258.5	137.7	118.3	19.5	-12.1
Peas and beans	324.8	140.8	138.6	2.23	-29.3
Rapeseed and mustard	305.9	193.1	147.8	45.4	13.8
Linseed	120.8	56.3	117.3	-60.9	-92.5
Potato	8,218.0	1621.4	496.0	1125.5	1093.9

Source: Authors’ calculation.

#### Maize

With 2.5 t/ha yield from 8.69 million ha of land, India produced 21.8MMT of maize in 2015–16 (Table 2), and the country ranked 7^th^ in maize production in the world. The maize area in India increased rapidly in the late 1990s. In 1993–94, the total area under maize was 6 million ha, which was increased to 7.34 million ha in 2003–04. Currently, the total maize area is 8.69 million ha [39]. Karnataka, Madhya Pradesh, and Bihar are the top maize-producing states (Table 4). India is a net exporter of maize averaging exports of 1.6MMT of maize, worth USD 376 million (TE2016). The major importers of Indian maize are Bangladesh, Nepal and Sri Lanka (Table 4).

Assuming a complete replacement of 287 thousand ha of wheat area in the sampled districts with maize, the simulation exercise shows that, with the average yield in the sampled districts of 5.5 t/ha, the total additional maize production will be 1.16MMT, resulting in a positive net return of USD 32.5 million (Table 5). Considering the rapid increase in the domestic maize use as feed in the poultry industry [61], in the short-run, maize cultivation can be expanded in the border districts of West Bengal. Still, MoT reportedly survives in maize [36], undermining its effectiveness for wheat-blast eradication so that it would become potentially more of a permanent substitute crop for wheat.

#### Lentils

With the total production of 1.04MMT from 1.47 million ha of land, India is the second largest lentil producer in the world after Canada (Tables 2 and 4). It is the most common food item in India, and the country produces 16.3% of the total lentils in the world. In India, Madhya Pradesh alone produces more than 40% of the lentils; Uttar Pradesh produces nearly 23%, and Bihar produces nearly 19% of the total lentils in India (Table 4). Even though the area under lentils has doubled from 0.75 million ha in 1970–71 to 1.47 million ha in 2015–16 [39], the per capita overall consumption of pulses has been declining in India mainly due to the failure to maintain yield growth rate with population-driven consumption growth. For example, in 1961, the yearly per capita consumption of pulses in India was 17.9kg, which has been reduced to 10.06 kg in 2013 [33]. Despite being the second largest producer in the world, the country is a net importer of lentils. From 2013 to 16, on a triennium average, India imported 872 thousand tons of lentils, worth USD 663 million (Table 4). The major lentil trade partners are Australia, Canada, and the United States of America (USA).

The nine border districts of West Bengal are the major lentil producers in West Bengal. These districts allocate nearly 62 thousand ha of land to lentils, which is more than 88% of the total area in West Bengal (Table 2). Assuming a complete replacement of the current wheat area of the nine border districts by lentils, the simulation exercise shows that, with an average yield of 0.82 t/ha, the total additional lentil production will be 227.7 thousand MT. Considering production costs of USD 413/ha and the price of USD 533/ton (Table 2), the substitution would imply a net loss of USD 28.5 million (Table 5). Thus, lentils cannot be a feasible alternative crop to wheat in the border districts of West Bengal.

#### Gram

With a total production of 7.17MMT from 8.35 million ha of land, India ranked number one in gram (chickpea, *Cicer arietinum)* production in the world, as India produces nearly 65% of the total gram in the world (Table 2 and Table 4). Madhya Pradesh alone produces nearly 46% of the total gram. Karnataka and Rajasthan are the second and third largest gram-producing states, and they produce 12.5% and 11.2% of India’s total gram (Table 4). Land allocation to the gram in India is oscillating around 8.35 million ha since 2009. Despite being the single largest producer of the gram in the world, India is a net importer of the gram. From 2014 to 16, on a triennium average, India imported nearly 475 thousand MT of gram worth USD 274 million (Table 4). India imports gram mainly from Australia, Russia, and Tanzania.

With an average yield of 0.96 t/ha, the nine border districts of West Bengal are also the major gram producers in West Bengal. The sampled nine districts allocated 18.3 thousand ha of land to the gram, which was 61% of the total gram area in West Bengal (TE2015-16, Table 2). Assuming a complete replacement of the current wheat area of the nine border districts by the gram, the total additional gram production will be 276 thousand MT. Considering production costs of USD 332/ha and the price of USD 839/t (Tables 2 and 3), the substitution would yield a net gain of USD 105 million (Table 5). Thus, the gram can be a feasible alternative crop to wheat in the border districts of West Bengal.

#### Urad

The indigenous pulse *urad (*black gram, *Vigna mungo)* is one of the most popular pulses in India. With an average national yield 0.57 t/ha, India produced 1.87MMT of *urad* from more than four million ha of land in 2015–16 (Table 2) [39]. Madhya Pradesh, Uttar Pradesh, and Andhra Pradesh including Telangana state are the top producers of this domestic crop in India (Table 4). These states jointly supply more than 56% of the total *urad* in India. *Urad* is a non-tradable local pulse. With an average yield of 0.94 t/ha, the nine border districts of West Bengal are not the major *urad* producers in the State. These nine districts allocated only 12% of the total land allocated to *urad* in West Bengal (Table 2).

Assuming a complete replacement of the current wheat area of the nine border districts by *urad*, the total additional *urad* production would be nearly 208 thousand MT. Due to the low production costs of USD 214/ha and high prices of USD 708/t (Table 2), the substitution would yield a net gain of more than USD 54 million (Table 5). Thus, *urad* can be a feasible alternative crop to wheat in the border districts of West Bengal.

#### Khesari

*Khesari* (grass pea, *Lathyrus sativus)* is an important pulse indigenous to India, is mainly produced in Chhattisgarh, West Bengal, and Bihar. In 2015–16, India produced 282 thousand tons of *khesari* from 394 thousand ha of land with a national average yield 0.72 t/ha (Table 2). With an average yield of 0.86 t/ha, the nine border districts of West Bengal are the major producers of *khesari* in West Bengal. Note that *khesari* is used as feed in addition to human food [62]. Assuming a complete replacement of the current wheat area by *khesari*, the simulation exercise shows that the total additional *khesari* production would be 258.5 thousand MT (Table 5). Considering the production costs of USD 412/ha and with the price of USD 533/t, the result is a negative net return net of USD 12 million (Table 4). Thus, *khesari* cannot be a feasible alternative crop to wheat in the border districts of West Bengal.

#### Peas and beans

With a total production of 683 thousand MT from 741 thousand ha of land, India is the second largest peas and beans producer in the world after China (tables 2 and 4). India produces 19.5% of the total peas and beans in the world, and Uttar Pradesh alone produces more than 70% of peas and beans (Table 3). The other two major pea-and-bean-producing states are Rajasthan that produces more than 8% of the total peas and beans and Assam that supplies more than 6% of the peas and beans in India. Concerning net trade, India is a net importer of peas and beans and, on a triennium average, the country imported nearly 3.2MMT of peas and beans yearly from 2014 to 16 worth USD 1.7 billion (Table 4). In this case, the major trading partners are China, USA, and Thailand (Table 4). In our study, peas and beans include green and dry beans, dry and green chickpeas, cowpeas, and pigeon peas.

The nine border districts of West Bengal are the major peas and beans producers. Out of total 14.1 thousand ha of land allocated to peas and beans in West Bengal, the nine border districts allocated 11.3 thousand ha, which was more than 80% of the total land allocated to peas and beans in West Bengal (Table 2). Assuming a complete replacement of the current wheat area of the border districts by peas and beans, the simulation exercise shows that with an average yield of 1.04t/ha, the total additional peas and beans production would be nearly 325 thousand MT (Table 4). With the relatively higher production costs of USD 483.3/ha (Table 3) and lower yield and price (Table 2), the substitution would imply a net loss of USD 29.3 million (Table 5). Thus, peas and beans cannot be a feasible alternative crop to wheat in the border districts of West Bengal.

#### Rapeseed and mustard

As the major oilseeds, the land allocation to rapeseed and mustard has increased over the years in India. In 1950–51, 2.07 million ha of land were allocated to rapeseed and mustard and, with an average yield 0.37 t ha^-1^, India produced 0.76MMT of rapeseed and mustard [39]. In 2015–16, with an average yield 1.18 t ha^-1^, India produced 6.8MMT of rapeseed and mustard from 5.8 million ha of land (Table 2). Currently, India is ranked as the third largest rapeseed-and-mustard producers that supply 9.8% of the total rapeseed and mustard in the world (Table 4). Interestingly, nearly 48% of the total rapeseed and mustard is produced only in Rajasthan (Table 4). Haryana and Madhya Pradesh are also major producers, supplying nearly 12% and 10% of total rapeseed and mustard in India (Table 4). In terms of international trade, India is a net exporter of rapeseed and mustard with a triennium average (2014–16) export of 18.8 thousand MT worth of USD 11.7 million (Table 4). The major destinations of Indian rapeseed and mustards are Nepal, USA, and the UK.

In West Bengal, with a land allocation of nearly 349 thousand ha, which is nearly 76% of the total rapeseed and mustard land in West Bengal, the nine border districts are the major rapeseed and mustard producers. Assuming a complete replacement of the current wheat area of the nine border districts by rapeseed and mustard, the simulation exercise shows that, with an average yield of 1.11 t/ha, the total additional rapeseed-and-mustard production will be nearly 306 thousand MT worth USD 193.1 million (Table 5). With relatively high production costs of USD 515.3/ha (Table 4) and a high price of USD 631.4/t (Table 2), the replacement of rapeseed and mustard in current wheat areas would imply a net gain of nearly USD 14 million (Table 5). Thus, rapeseed and mustard can be a feasible alternative crop to wheat in the border districts of West Bengal.

#### Linseed

Currently, with 153 thousand MT production from 298 thousand ha of land with an average national yield 0.54 t/ha (Table 2), India is the sixth largest linseed-producing country in the world (Table 4). India produces nearly 4% of the total linseed in the world, and the country is a net exporter of the crop (Table 4). The major destinations of Indian linseed are Germany, Netherlands, and Canada (Table 4). Madhya Pradesh (39.3%), Bihar (10.6%), and Uttar Pradesh (9.2%) are the major linseed-producing states (Table 4). In West Bengal, the nine border districts are the major linseed-producing districts in terms of land allocation and production (Table 2). With an average-level yield 0.39 t/ha, the nine border districts supply 75% of the total linseed of West Bengal (Table 2).

Assuming a complete replacement of the current wheat area of the nine border districts by linseed, the simulation exercise shows that, with an average yield of 0.39t/ha, the total additional linseed production from 286.8 thousand ha of the current wheat land would be 120.8 thousand MT worth USD 56.3 million (Table 5). Despite the high price of USD 465.9/t, due to the lower yield of 0.39 t/ha (Table 2), the overall returns to linseed do not look favorable compared to the returns of wheat. The replacement of linseed for wheat would generate a net loss USD 92.5 million (Table 5). Thus, linseed cannot be a feasible alternative crop to wheat in the border districts of West Bengal.

#### Potatoes

With an average national yield 20.5 t/ha, India produced 43.8MMT of potatoes from 2.1 million ha of land in 2015–16 (Table 2), which was 11.6% of the total potatoes produced in the world, making India the second-largest potato-producing country in the world after China (Table 4). Because of exceptionally high yields, land allocation to potatoes has increased in India dramatically over the years. For example, in contrast to 2.13 million ha of land in 2015–16, in 1950–51, the land allocated to potatoes was only 0.24 million ha, and it had increased to 1.03 million ha by 1991–92 [39]. In terms of production, Uttar Pradesh produces nearly 31% of the total potatoes produced in India; West Bengal produces 25%, and Bihar produces 13% (Table 4). The highest potato yield (30.2 t/ha) was observed in Gujarat, followed by West Bengal (29.2 t/ha) and Punjab (25.1 t/ha) [39]. India is a net exporter of potatoes with an average export of 242.7 thousand MT worth USD 74.8 million (Table 4). The major destinations of Indian potatoes are Nepal, Sri Lanka, Oman, and Mauritius (Table 4). In West Bengal, the nine border districts allocate nearly 132 thousand ha of land to potatoes, which is nearly 32% of the total potato area in West Bengal.

Assuming a complete replacement of the current wheat area of the nine border districts by potatoes, the simulation exercise shows that with an average yield of 27.4 t/ha, the total additional potato production from 287 thousand ha of the current wheat land will be 8.2MMT worth USD 1.6 billion (Table 5). Despite the highest production cost of USD 1730/ha and the lowest price of USD 197/t, due to the highest average yield of 27.4 t/ha in the nine sampled districts (Table 2), the overall returns for potatoes is highly positive compared to the returns for wheat and any other sampled competing crops. Our simulation exercise shows that the replacement of potatoes for wheat would generate a net gain of nearly USD 1.1 billion (Table 5). Thus, potatoes can be a feasible alternative crop to wheat in the border districts of West Bengal.

### Conclusions and policy implications

Wheat is the second major staple of India, and a vast area of its wheat is vulnerable to wheat blast [28]. An intrusion of wheat blast (MoT) into India from the recent incidences in Bangladesh could have severe negative impacts on India’s overall food security. As a precautionary measure, the West Bengal government has already banned wheat cultivation within five kilometers of the Bangladesh border and banned wheat production in Murshidabad and Nadia districts of West Bengal [34]. However, to realistically implement a potential ‘wheat holiday’ policy in any wheat-producing region or country, it is imperative to suggest economically-viable alternative crops to replace wheat. Although West Bengal in itself is not a major wheat producer in the Indian context, it could potentially serve as a bridge for MoT intrusion from Bangladesh. The present study, therefore, examined the economic viability of alternative crops to wheat for a possible extension of the wheat holiday to the nine border districts of West Bengal.

Applying a scoping *ex-ante* estimation framework, this study ruled out the possibility of replacing wheat with winter paddy, peas and beans, and linseed in those districts of West Bengal, due to their negative net margins. On the other hand, growing maize, gram, *urad*, rapeseed, mustard, and potatoes in place of wheat appear to be profitable. This would need to be confirmed by more rigorous and grounded empirical data to support the transition to these more promising alternatives. In addition, some caution is needed when promoting cereal crops such as maize (for possibly being an alternate host) and non-cereal crops due to the associated investments needed in value chains, such as cold storage for potatoes, credit facilities, and marketing costs of the export-oriented commodities. Among the profitable crops, potato and maize also imply substantially higher labor costs, which may be an issue in the face of labor calendars and increasing labor scarcity in India. For simplicity, the study assumes a complete wheat substitution by the alternate crops, but in reality, combinations may be more realistic and profitable.

A potential ‘wheat holiday’ in the nine border districts in West Bengal may not severely affect India’s total domestic wheat production given its relatively modest share in India’s wheat area and production. However, the proposed wheat holiday policy in West Bengal may not bring the desired outcome, if Bangladesh continues wheat production in its border districts. The wheat-blast host-pathogen systems potentially undermine the feasibility of a potential one-sided ‘wheat holiday’ by India. To generate a desirable outcome, along with India, Bangladesh also would need to introduce a wheat holiday policy in its blast affected and particularly its border districts. Considering the coordination and logistics costs, such inter-country collaboration might be challenging in reality, in addition to the fact that Bangladesh already is a net wheat-importing country. Alarmingly, an implementation of a wheat holiday in West Bengal and Bangladesh may not eliminate wheat blast from the hot spots as alternative crops may harbor this particular pathogen (potentially including maize) or variants thereof (e.g., other pathotypes of the rice blast fungus, *Magnaporthe oryzae*).

Based on our findings, to avoid wheat blast intrusion from the border districts of Bangladesh, in the short-run, the Government of India may encourage farmers in all the border districts to cultivate economically viable legumes such as gram, *urad*, and oilseeds such as rapeseed and mustard, and potatoes instead of wheat. MoT can survive on seeds for up to 22 months [55], so the government may want to implement the wheat holiday policy for at least two years. Also in the short run, the government of India must make fungicide treatment mandatory to avoid any seed borne spread of wheat blast in the border districts of West Bengal.

For a more structural long-term solution, however, further investments are needed in wheat-blast-related research and development. Considering the importance of wheat for food security in South Asia and India in particular, the present study, therefore, calls for concerted action from the national governments in South Asia and international stakeholders. Until now, there is no specific molecular diagnostic tool for the determination of wheat blast [63] in suspected seeds, alternative hosts and symptomless plants. There is an urgent need to develop a convenient diagnostic tool for wheat blast to support surveillance and to invest in disease epidemiology and forecasting research. In addition, there is a need to develop a platform for open data and science to combat this worrisome fungus. Finally, there is a pressing need to develop and disseminate new blast-resistant wheat varieties and complementary management practices in the South Asia setting.

## Supporting information

S1 File Newspaper report. UK Researchers Find Important New Disease.
(PDF)

S2 File Minutes of a meeting on the technical meeting regarding occurrence of Blast disease on wheat held under the Chairmanship of Secretary (AC&FW) on 28. 06. 2016. (PDF)

S3 File Book on farm harvest prices of principal crops in India 2015–16. (PDF)

S4 File Book on agricultural statistics 2017. (PDF)

S5 File Book on annual report 2016–17. (PDF)

S6 File Data: Simulation exercise sheet. (XLSX)

S7 File Data. Data on cost of cultivation and production & related data.
(XLSX)
