# Supplementary material for: Averting wheat blast by implementing a ‘wheat holiday’: In search of alternative crops in West Bengal, India
Source: PLoS One. 2019 Feb 20;14(2):e0211410. doi: 10.1371/journal.pone.0211410 (PMC6382110; doi:10.1371/journal.pone.0211410)
Supplement: S1 File — UK Researchers Find Important New Disease. (PDF) [file pone.0211410.s001.pdf]

UK Researchers Find Important New Disease

LEXINGTON, KY.

University of Kentucky College of Agriculture specialists are encouraging Kentucky wheat producers and crop consultants to scout their fields for a new disease that could have important implications for future crop years.

UK soil scientist Lloyd Murdock found wheat blast on a single wheat head May 18, 2011 at a UK Research and Education Center research plot in Princeton. No additional instances of the disease were found even after extensive scouting of the involved research plots and neighboring fields by UK researchers. It is likely, however, that additional infected heads existed but at levels too low to make detection possible.

Wheat blast is a disease that is recognized as an emerging threat worldwide. Caused by the fungus Magnaporthe oryzae/Pyricularia grisea, the disease was first detected in southern Brazil in 1985 and has since become a problem in several of its neighboring countries. Crop losses of 40 percent are common and cases of 100 percent loss have been reported. Currently, there are no commercially available resistant varieties and fungicidal programs targeting wheat blast have generally been ineffective.

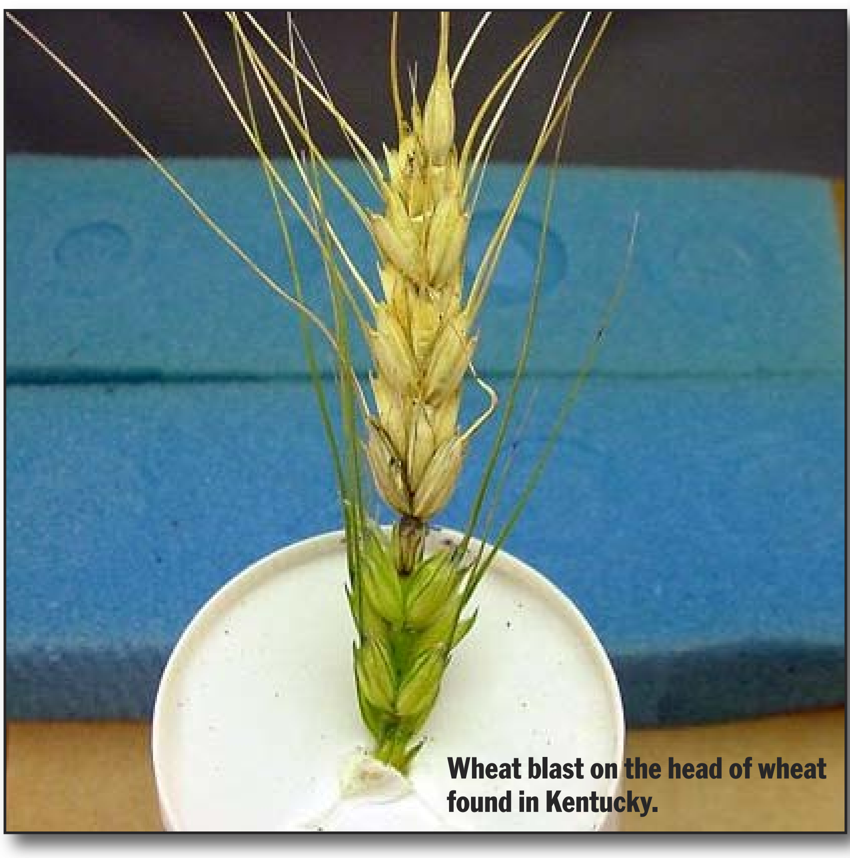

Wheat blast on the head of wheat found in Kentucky.

The Kentucky find is the first known occurrence of wheat blast outside of South America. However, a related pathogen has caused a disease called gray leaf spot on annual ryegrass in Louisiana and Mississippi since the 1970's and on perennial ryegrass in much of the humid regions of the United States since 1992. Annual ryegrass is a common forage crop in the South, and it is also recognized as a weed in Kentucky wheat fields. Perennial ryegrass is grown as a turfgrass in Kentucky, as well as an infrequent forage grass. There is also a disease common in rice produced in the Mid-South called rice blast. Although rice blast is caused by the same fungus, isolates of the fungus known to infect rice do not infect wheat and vice-versa. Thus, rice producers who grow wheat in extreme southwest Kentucky and surrounding states are not at increased risk from the wheat blast fungus.

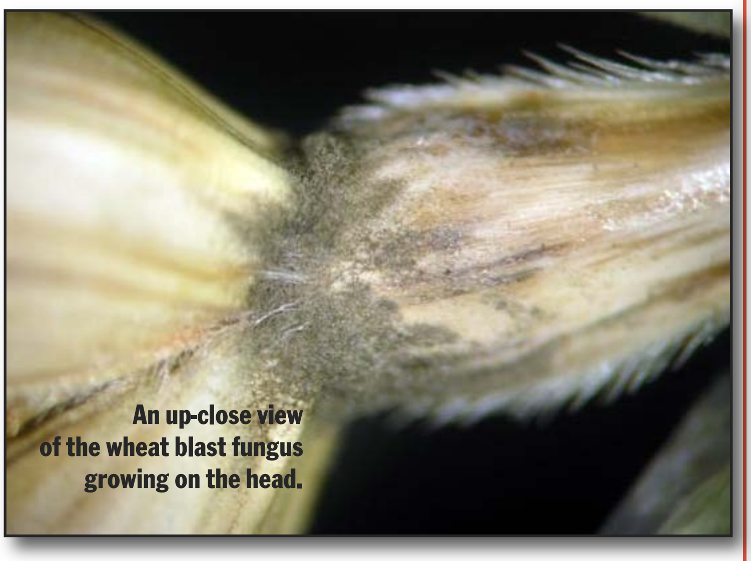

An up-close view of the wheat blast fungus growing on the head.

UK plant pathologist Mark Farman has spent most of his career studying the fungi that cause the blast and gray leaf spot diseases. He sequenced the genome of the isolate found at the UK Research and Education Center and compared it to the genetic structure of isolates found on ryegrasses and from South American wheat blast.

Farman found the annual ryegrass pathogen and the wheat blast pathogen discovered at the Research and Education Center to be genetically very similar. This led him to think that the annual ryegrass pathogen gained the genetic ability to infect wheat. The UK wheat blast pathogen's genetic structure was most different from the wheat blast pathogen known to affect South American wheat. His results, thus far, suggest that the blast fungus found on the Kentucky wheat head has probably been around for at least a decade or longer on annual and perennial ryegrass and perhaps on wheat, and almost certainly was not imported with grain originating from South America. This is important, because it means that the new find is not an exotic pathogen. Farman said the pathogen is usually host-specific, meaning the fungus that attacks annual ryegrass normally will only attack annual ryegrass or its close relative, perennial ryegrass. This is the first time he's seen this pathogen able to "jump" hosts.

Samples of the fungus collected from Kentucky wheat were also sent for testing to Gary Peterson, a researcher with the U.S. Department of Agriculture's Agriculture Research Service. He confirmed the fungus collected from wheat found in Princeton was able to infect wheat. Moreover, he found the Kentucky isolate was similar in its ability to cause disease as isolates that cause wheat blast in South America.

With Farman's research linking the wheat blast found in Kentucky to annual ryegrass, it's very unlikely this is an isolated find.

"It's very unlikely that we're the only ones who have it," said Don Hershman, UK extension plant pathologist. "Annual ryegrass is a very common forage crop in states to our south."

UK researchers suspect it may have been misdiagnosed in the past as Fusarium head blight, a very common disease with very similar symptoms to wheat blast. However, there are some distinct differences between the two diseases. Wheat blast will appear in fields slightly earlier than Fusarium head blight. In addition, Fusarium head blight tends to give infected glumes and kernels a salmon to pinkish tint, and the disease is often found sporadically throughout affected heads. Wheat blast, like Fusarium head blight, results in partial death of diseased heads but does not cause multiple infected areas within single heads. With blast, heads will turn white or tan from the point of infection upward and have what looks like grayish mold growing at the point of attachment of individual spikelets that make up the wheat head. If producers see what looks to be Fusarium head blight, but symptoms appear earlier than expected, and they do not see evidence of salmon or pinkish discoloration in diseased tissue, there is a good chance that it is wheat blast, said UK specialists.

The fungus that causes wheat blast thrives in warm, wet conditions. This is believed to be the reason why wheat blast in Kentucky is not currently a production problem, even though the causal fungus has apparently been around for years. The weather is simply not favorable for blast development during a normal Kentucky spring.

Farman said he usually looks for the disease on ryegrasses and other host plants in July and August. Finding the fungus infecting wheat in late May 2011 was highly unusual, but could indicate that wheat blast and related diseases in ryegrasses might become more problematic if average spring temperatures trend warmer in future years, especially if accompanied by increased moisture.

Although UK specialists do not believe wheat blast represents a current economical threat to Kentucky's wheat crop, they do believe it is important to get a better feel for how common the disease is in Kentucky. They are encouraging producers to scout wheat fields for blast, as well as observe annual and perennial ryegrasses for the occurrence of gray leaf spot lesions on leaves.

"Gray leaf spot first appears as dead lesions that are elliptical or roughly diamond-shaped and range in color from gray to tan with a thin, brown border," said UK extension plant pathologist Paul Vincelli. These lesions can quickly turn into leaf blight which causes the leaf to completely dry up.

If producers suspect they have wheat blast or gray leaf spot, they should send a sample to the UK Plant Disease Diagnostic laboratories in Princeton or Lexington.

Farman added it's fortunate that researchers found the disease at such a low level, as it provides an early warning of a possible emerging issue and, hopefully, will give researchers time to develop resistant varieties for combating the disease before it becomes a major threat to wheat production.

Farman is working with the International Wheat Blast Consortium led by researchers at Kansas State University. The group is trying to identify wheat varieties that are resistant to wheat blast and characterize the wheat blast pathogen using molecular markers. Δ

Cache River Valley Seed, LLC
888-CRV-SEED
Link Directly To: CASH RIVER
