# Supplementary material for: Averting wheat blast by implementing a ‘wheat holiday’: In search of alternative crops in West Bengal, India
Source: PLoS One. 2019 Feb 20;14(2):e0211410. doi: 10.1371/journal.pone.0211410 (PMC6382110; doi:10.1371/journal.pone.0211410)
Supplement: S2 File — (PDF) [file pone.0211410.s002.pdf]

S No 2(I)

File No. 4-9/2013-NFSM
Government of India
Ministry of Agriculture & Farmers Welfare
Department of Agriculture Cooperation & Farmers Welfare
Crops Division

Krishi Bhawan, New Delhi
Dated: 14th July, 2016

To

The Principal Secretary (Agriculture)/ Secretary (Agriculture)
Government of Assam, Bihar, Tripura, Uttar Pradesh and West Bengal

Subject: Minutes of the technical meeting regarding occurrence of Blast disease on wheat held under the Chairmanship of Secretary (AC&FW) on 28.06.2016 at 12:00 noon in Acharya Jagdish Chandra Bose Hall, Krishi Bhawan, New Delhi.

The undersigned is directed to enclose herewith a copy of the minutes of technical meeting regarding occurrence of Blast disease on wheat held under the Chairmanship of Secretary (AC&FW) on 28.06.2016 at 12:00 noon in Acharya Jagdish Chandra Bose Hall, Krishi Bhawan, New Delhi for information and necessary action.

Encl. As above:

14/7/16
(Dr. D. P. Malik)
Additional Commissioner (Crops)

O I C

Distribution to:

1. Deputy Director General (Crop Science), ICAR, Krishi Bhawan, New Delhi.
2. Assistant Director General (FFC), ICAR, Krishi Bhawan, New Delhi.
3. Assistant Director General (PP), ICAR, Krishi Bhawan, New Delhi.
4. Director, Indian Institute of Wheat & Barley Research, ICAR, Karnal.
5. Director of Agriculture, Government of Assam, Bihar, Tripura, Uttar Pradesh and West Bengal.
6. Plant Protection Advisor, Directorate of Plant protection & Quarantine, GoI, NH-IV, Faridabad, Haryana.
7. Director, National Institute of Biotic Stress Management, Raipur, Chhattisgarh.
8. Director, Directorate of Wheat Development, DAC&FW, Ghaziabad.
9. Country Head, CIMMYT-India, NASC Complex, DPSM, New Delhi.

Copy to:

1. PPS to Secretary (AC&FW), DA,C&FW, Krishi Bhawan, New Delhi.
2. PPS to Agriculture Commissioner (DA,C&FW), Krishi Bhawan, New Delhi.
3. PPS to Joint Secretary (Crops), DA,C&FW, Krishi Bhawan, New Delhi.
4. PPS to Joint Secretary (Seeds), DA,C&FW, Krishi Bhawan, New Delhi.
5. PPS to Joint Secretary (Trade), DA,C&FW, Krishi Bhawan, New Delhi.
6. PS to Addl. Commissioner (Crops), DA,C&FW, Krishi Bhawan, New Delhi.
7. Consultants, NFSM, DA,C&FW, Krishi Bhawan, New Delhi.
8. Programmer, , NFSM, DA,C&FW, Krishi Bhawan, New Delhi.

Issued 15/7/2016

Scanned by CamScanner

Minutes of the technical meeting regarding occurrence of Blast disease on Wheat held under the Chairmanship of Secretary (AC & FW) on 28.06.2016 at 12.00 noon in Acharya Jadish Chandra Bose Hall, Krishi Bhawan, New Delhi.

The technical meeting on recent reports of occurrence of Blast disease on wheat in Bangladesh and to take the remedial steps to prevent the spread of wheat blast in India was held under Chairmanship of Secretary (AC & FW) on 28.06.2016. Krishi Bhawan, New Delhi.

2. The list of participants is at Annexure I.

3. Agriculture Commissioner welcomed the Chairman and other participants in the meeting and informed the house that wheat blast disease was spotted in Bangladesh during March 2016 in six south western districts (Kushtia, Meherpur, Chuandaga, Jhenidah, Jessore and Magura) which affected 15000 ha area and expressed serious concern as Bangladesh is adjoining to our states like West Bengal, Tripura, Assam, and Meghalaya. The Chairman emphasized the need to take remedial steps to prevent the spread of wheat blast in our country.

4. Country Head, CIMMYT India informed that the blast disease of wheat was first reported from Brazil in 1985 and since then have been known to periodically occur in Latin America countries (Bolivia/Brazil/Paraguay/Argentina) and the wheat crop gets ruined within 15 days of its infestation. This disease is caused by *Magnaporthe oryzae, pethotype triticum*. Wheat blast is a potential threat to wheat production Worldwide. He also stated that disease severely varied with soil type, cultivars, higher temp and in late planted wheat even loss is upto 90%. The most severe field infections occur in seasons when the period around and anthesis features continuous rainfall and an average temperature is around 18-25^0 C followed by a period of sunny hot and humid weather. He said that wheat blast is a very dangerous disease which strikes quickly resulting in shrivelling and deforming of wheat grains, leaving farmers no time to act. Fungicides provide only a partial defense and must be applied before symptoms appear. As a long term strategy, there is need to focus on varietal resistance; crop management – early planting seed treatment, preventive foliar spray, residue management and varietal diversity.

14/7/16

Scanned by CamScanner

5 DDG (CS), ICAR apprised that as soon as the message of outbreak of wheat blast was received from Bangladesh Govt. on 31st March, 2016, the ICAR constituted the teams of scientists from Indian Institute of Wheat and Barley Research and carried out extensive survey in wheat growing areas of adjoining states to Bangladesh and other wheat growing areas of the Country. The teams reported that wheat blast disease has not occurred anywhere in wheat growing areas of the Country. He further shared that even in surveys conducted earlier no report of wheat blast was received. He expressed his apprehension that perhaps the disease has reached Bangladesh from the material brought from Latin American countries. He further reported that as a follow up action, 40 lines of Indian promising varieties of wheat have been identified and sent to CIMMYT for testing against the wheat blast at Latin America (Bolivia, Brazil, Paraguay and Argentina) which are the hot spots for this disease. DDG (CS) further apprised that ICAR is going to take following steps for management of wheat blast in India:

(i) Off season survey will be undertaken in different part of the Country with special emphasis of wheat blast.

(ii) A project will be initiated on study the impact of wheat blast in India on all grasses and wheat varieties, its impact and remedial measures to be under taken.

(iii) Continuous monitoring system would be put in place for monitoring of incidence of wheat blast in border areas of Bangladesh which will also include adjoining districts of West Bengal, Tripura and Assam where wheat crop is grown. DDG (CS) also requested to consider the action from the DAC&FW for stopping the import of wheat grain in any form from of Latin American countries i.e. Brazil, Bolivia, Parugvay & Argentina and Bangladesh.

6. Principal Scientist (PP), IIWBR, Karnal presented a brief report on wheat blast and informed that wheat blast is present in wheat production areas of Brazil, Bolivia, Paraguay, in a small area in northeast Argentina & Bangladesh and casual organism for wheat blast is *Magnaporthe oryzae* which is the same fungal species that causes rice blast. Wheat blast pathogen has a distinct population of *M. oryzae* (referred as *M. oryzae Triticum* population). This disease also occurs on Triticale, black oats and barley. *M. oryzae Triticum* pathotype infect all above ground parts of wheat plant. Most common symptom in field is head blast resembling head scab or Fusarium Head Blight. Infected

14/10/16

Scanned by CamScanner

spikes become bleached, shrivelled seed. Risk for introduction of wheat blast from Bangladesh is high due to its seed borne nature and trade and travel. He further said that teams of IIWBR carried out 250 surveys on wheat blast and no report of its occurrence was received from any part of the Country.

7. Plant Protection Advisor informed that wheat blast is not among the identified diseases of wheat so far hence it has to be included and similarly no specific fungicide is available for the control of wheat blast.

8. Chairman in his concluding remarks said that it's a very serious issue as wheat is major cereal crop of the Country. Although as of now there are no reports of occurrence of wheat blast in the Country but its occurrence in Bangladesh has given us warning signals and infestation of this disease of wheat crop will be devastating and we cannot afford it. Wheat being a rabi crop, we still have time to plan out the strategies for taking all the remedial steps on war footing and all essential steps should be taken to stop the entry of wheat blast in the Country.

9. During the deliberations the following actionable points emerged are as under:

(i) All forms of seed of wheat and wheat grain from Latin America should not be allowed.
(Action-Seed Division, Directorate of Plant Protection Quarantine & Storage (PPQ&S) of DAC&FW, ICAR & Foreign Trades)

(ii) Ensure that no wheat grain and seed enters from any of the entry points from Bangladesh to adjoining states (West Bengal, Tripura, Assam and Meghalaya)
(Action-Seed Division, PPQ&S of DAC&FW, ICAR & Foreign Trades)

(iii) Entry point in adjoining districts of West Bengal, Tripura, Assam, Mizoram and Meghalaya from Bangladesh may be identified in consultation with State Department of Agriculture and District Administration of the districts and close watch kept.
(Action-State Governments of West Bengal, Tripura, Assam, Mizoram and Meghalaya)

(iv) A corridor plan to be carved out in adjoining areas of Bangladesh border to ensure that no wheat cultivation is taken in this corridor.
(Action-State Governments of West Bengal, Tripura & Assam)

[illegible]

Scanned by CamScanner
