# Supplementary material for: Averting wheat blast by implementing a ‘wheat holiday’: In search of alternative crops in West Bengal, India
Source: PLoS One. 2019 Feb 20;14(2):e0211410. doi: 10.1371/journal.pone.0211410 (PMC6382110; doi:10.1371/journal.pone.0211410)
Supplement: S3 File — (PDF) [file pone.0211410.s003.pdf]

FARM HARVEST PRICES OF

PRINCIPAL CROPS IN INDIA

2015-2016

सत्यमेव जयते

DIRECTORATE OF ECONOMICS AND STATISTICS

DEPARTMENT OF AGRICULTURE, COOPERATION AND

FARMERS WELFARE

MINISTRY OF AGRICULTURE AND FARMERS WELFARE

GOVERNMENT OF INDIA

NEW DELHI

DECEMBER, 2017

पुष्पा थोट्टन
सलाहकार

भारत सरकार,
कृषि एवं किसान कल्याण मंत्रालय
कृषि, सहकारिता एवं किसान कल्याण विभाग
अर्थ एवं सांख्यिकी निदेशालय
कृषि भवन/शास्त्री भवन
नई दिल्ली-110001

सत्यमेव जयते

Pushpa Thottan
Adviser

Government of India,
Ministry of Agriculture & Farmers Welfare
Department of Agriculture, Cooperation & Farmers Welfare
Directorate of Economics & Statistics
Krishi Bhawan/Shastri Bhawan
New Delhi - 110001

FOREWORD

Directorate of Economics & Statistics, Ministry of Agriculture and Farmers Welfare has been bringing out "Farm Harvest Prices of Principal Crops in India" every year. The importance of this publication lies in the fact that data on farm gate prices facilitate fine-tuning of policies aimed to promote farmers' welfare. This issue contains data on farm harvest prices of principal crops for the year 2015-16, based on the returns received from the State Governments and Union Territories. A note on the methodology for collection of data on farm harvest prices and the particulars of harvesting seasons of principal crops in the major growing States has been provided at the end of the statistical tables. The data presented in this publication are available on the website: http://eands.dacnet.nic.in/ of the Ministry of Agriculture and Farmers Welfare.

The detailed work has been carried out by the Price and Market Division, Directorate of Economics & Statistics, Ministry of Agriculture & Farmers Welfare under the overall guidance and supervision of Shri B.L. Meena, Additional Economic Adviser. We are grateful to the States which have given us timely information for inclusion in this volume and solicit the continued cooperation of all States in the future.

It is hoped that the publication would be useful for a wide range of researchers and policy makers. We welcome suggestions for improvement of this publication.

(Pushpa Thottan)

NEW DELHI
DECEMBER, 2017

बत्तू लाल मीणा, आई ई एस
अतिरिक्त आर्थिक सलाहकार

सत्यमेव जयते

B. L. Meena, IES
Additional Economic Adviser

भारत सरकार,
कृषि एवं किसान कल्याण मंत्रालय
कृषि एवं सहकारिता विभाग
अर्थ एवं सांख्यिकी निदेशालय
कृषि भवन/शास्त्री भवन
नई दिल्ली-110001

Government of India,
Ministry of Agriculture & Farmers Welfare
Department of Agriculture & Cooperation
Directorate of Economics & Statistics
Krishi Bhawan/Shastri Bhawan
New Delhi - 110001

PREFACE

"Farm Harvest Prices of Principal Crops in India" is a publication, which is being brought out every year by the Directorate of Economics and Statistics of the Ministry of Agriculture & Farmers Welfare. The present volume contains information on farm harvest prices of 35 principal agricultural crops for the year 2015-2016, which have been compiled on the basis of information sent by all States and Union Territories. Agricultural crops covered are Paddy, Rice, Jowar, Bajra, Maize, Ragi, Wheat, Barley, Gram, Arhar, Sugar Raw (Gur), Sugarcane, Potato, Tobacco, Groundnut, Rapeseed, Sesamum, Linseed, Castor seed, Nigerseed, Soyabean, Sunflower, Cotton, Jute, Sannhemp, Mesta, Pepper, Ginger, Chilly, Turmeric, Arecanut, Banana, Tapioca, Coconut and Cashewnut.

Task of collection, compilation and presentation of farm harvest prices data for this publication has been carried out by the staff of Price and Market Division under the supervision of Shri Rakesh Agrawal, Deputy Director and Smt. Swathi S.Senan, Economic Officer. The staff members associated with the preparation of this publication are:

1. Smt. Jaishree Kumar, Senior Statistical Officer
2. Smt. Suman Devi, Junior Statistical Officer
3. Smt. Anita Bhatnagar, Technical Assistant
4. Shri. Hemendra Kumar Meena, Junior Statistical Officer
5. Shri. Anurag Bhullar, Junior Statistical Officer
6. Shri. Udai Pal, DEO (Grade-C)

Sincere efforts have been taken to bring out this publication. Suggestions for improvement are most welcome.

BMeena

(B.L. Meena)

NEW DELHI
DECEMBER, 2017

CONTENTS

Table No.	SUBJECT	Page No.
1	Crop-wise State-wise Weighted average of Farm Harvest Prices of Principal Crops In India 2014-15	1-12
2	Crop-wise District-wise Farm Harvest Prices of Principal Crops in India	
2.1	Andhra Pradesh	13-16
2.2	Andaman & Nicobar	17
2.3	Assam	18-19
2.4	Bihar	20-23
2.5	Chandigarh	24
2.6	Chhattisgarh	25-26
2.7	Delhi	27
2.8	Goa	28
2.9	Gujarat	29-31
2.10	Haryana	32-33
2.11	Himachal Pradesh	34-35
2.12	Jammu & Kashmir	36-37
2.13	Jharkhand	38-40
2.14	Karnataka	41-46
2.15	Kerala	47-48
2.16	Madhya Pradesh	49-52
2.17	Maharashtra	53-55
2.18	Manipur	56
2.19	Mijoram	57
2.20	Nagaland	58
2.21	Odisha	59-61
2.22	Pondicherry	62
2.23	Punjab	63-64
2.24	Rajasthan	65-67
2.25	Tamil Nadu	68-70
2.26	Telangana	71-72
2.27	Tripura	73
2.28	Uttar Pradesh	74-76
2.29	Uttranchal	77
2.30	West Bengal	78-79
	**APPENDIX**	
I	Methodology for Collection of Farm Harvest Prices	80-82
II	Harvesting Seasons of Principal Crops in Major Growing States	83-94
III	Minimum Support Price	95

Table 1 Crop-wise State-wise Weighted Average* of Farm Harvest Prices of Principal Crops in India

(Rs.per Quintal)

Crop Name	Variety	Year	Season $	Andhra Pradesh	Andman & Nicobar	Assam	Bihar	Chandigarh	Chhattisgarh	Delhi	Goa	Gujarat	Haryana	Himachal Pradesh	Jammu & Kashmir
Arecanut	-	2015-16	-	-	17500	2961	-	-	-	-	23000	-	-	-	-
			Kharif	-	-	-	-	-	-	-	-	-	-	-	-
			Summer	-	-	-	-	-	-	-	-	-	-	-	-
Bajra	-	2015-16	-	1384	-	-	-	-	2197	1650	-	-	1329	-	1157
			Kharif	1387	-	-	1177	-	-	-	-	1261	1329	-	-
			Rabi	1327	-	-	-	-	-	-	-	-	-	-	-
			Summer	-	-	-	-	-	-	-	-	-	-	-	-
Banana	-	2015-16	-	-	-	-	-	-	-	-	-	-	-	-	-
Barley	-	2015-16	-	-	-	-	-	-	2001	1450	-	-	-	1967	1555
			Rabi	-	-	-	1144	-	-	-	-	-	-	-	-
Cashewnut	-	2015-16	-	-	-	-	-	-	-	-	12200	-	-	-	-
			Summer	-	-	-	-	-	-	-	-	-	-	-	-
	Raw	2015-16	-	-	5000	-	-	-	-	-	-	-	-	-	-
CasterSeed	-	2015-16	-	3011	-	7500	-	-	2220	-	-	3794	-	-	-
			Kharif	3011	-	-	-	-	-	-	-	-	-	-	-
			Rabi	-	-	-	-	-	-	-	-	-	-	-	-
Chilly	-	2015-16	-	-	-	-	-	-	-	-	-	-	-	12775	8528
			Kharif	-	-	-	-	-	-	-	-	-	5984	-	-
			Rabi	-	-	-	5691	-	-	-	-	-	-	-	-
	Dry	2015-16	-	11321	-	-	-	-	11180	-	-	10237	-	-	-
			Kharif	11204	-	-	-	-	-	-	-	-	-	-	-
			Rabi	12045	-	-	-	-	-	-	-	-	-	-	-
	Green	2015-16	-	-	11250	-	-	-	-	-	-	-	-	-	-
Coconut	-	2015-16	-	-	-	-	-	-	-	-	-	-	-	-	-
			Kharif	-	-	-	-	-	-	-	-	-	-	-	-
			Rabi	-	-	-	-	-	-	-	-	-	-	-	-
			Summer	-	-	-	-	-	-	-	-	-	-	-	-
Cotton	-	2015-16	-	4035	-	4693	-	-	-	-	-	-	-	-	-
			Kharif	4034	-	-	-	-	-	-	-	-	-	-	-
			Rabi	-	-	-	-	-	-	-	-	-	-	-	-
			Summer	-	-	-	-	-	-	-	-	-	-	-	-
	American	2015-16	-	-	-	-	-	-	-	-	-	-	-	-	-
			Kharif	-	-	-	-	-	-	-	-	-	5021	-	-
	Desi	2015-16	-	-	-	-	-	-	-	-	-	3088	-	-	-
			Kharif	-	-	-	-	-	-	-	-	-	5371	-	-
	Hybrid	2015-16	-	-	-	-	-	-	-	-	-	4078	-	-	-

Table 1 (Contd.) Crop-wise State-wise Weighted Average* of Farm Harvest Prices of Principal Crops in India

(Rs.per Quintal)

Crop Name	Variety	Year	Season $	Jharkhand	Karnataka	Kerala	Maharashtra	Manipur	Mizoram	Nagaland	Orissa	Pondicherry	Punjab	Rajasthan	Tamil Nadu
Arecanut	-	2015-16	-	-	-	-	-	-	-	-	-	-	-	-	20861
			Kharif	-	23899	-	-	-	-	-	-	-	-	-	-
			Summer	-	26281	-	-	-	-	-	-	-	-	-	-
Bajra	-	2015-16	-	-	-	-	1454	-	-	-	-	1538	-	-	-
			Kharif	-	1339	-	-	-	-	-	-	-	-	1267	-
			Rabi	-	-	-	-	-	-	-	-	-	-	-	-
			Summer	-	1678	-	-	-	-	-	-	-	-	-	-
Banana	-	2015-16	-	-	-	2939	-	-	1261	-	-	-	-	-	2403
Barley	-	2015-16	-	-	-	-	-	-	-	-	-	-	1127	-	-
			Rabi	1486	-	-	-	-	-	-	-	-	-	1408	-
Cashewnut	-	2015-16	-	-	-	-	-	-	-	-	-	-	-	-	10860
			Summer	-	9075	-	-	-	-	-	-	-	-	-	-
	Raw	2015-16	-	-	-	-	-	-	-	-	-	-	-	-	-
CasterSeed	-	2015-16	-	-	-	-	3051	-	-	-	-	-	-	-	4391
			Kharif	-	3285	-	-	-	-	-	-	-	-	-	-
			Rabi	-	-	-	-	-	-	-	-	-	-	3183	-
Chilly	-	2015-16	-	-	-	-	-	-	17010	-	-	-	-	-	10858
			Kharif	-	-	-	-	-	-	-	-	-	-	-	-
			Rabi	-	-	-	-	-	-	-	-	-	-	-	-
	Dry	2015-16	-	-	-	-	-	-	-	-	-	-	-	-	-
			Kharif	-	7653	-	-	-	-	-	-	-	-	10905	-
			Rabi	-	-	-	-	-	-	-	-	-	-	-	-
	Green	2015-16	-	-	-	-	-	-	-	-	-	-	-	-	-
Coconut	-	2015-16	-	-	-	-	-	-	930	-	-	-	-	-	-
			Kharif	-	10998	-	-	-	-	-	-	-	-	-	-
			Rabi	-	7005	-	-	-	-	-	-	-	-	-	-
			Summer	-	10049	-	-	-	-	-	-	-	-	-	-
Cotton	-	2015-16	-	-	-	-	-	-	4520	-	-	2799	-	-	4326
			Kharif	-	4415	-	-	-	-	-	-	-	-	4565	-
			Rabi	-	4216	-	-	-	-	-	-	-	-	-	-
			Summer	-	4266	-	-	-	-	-	-	-	-	-	-
	American	2015-16	-	-	-	-	-	-	-	-	-	-	4691	-	-
			Kharif	-	-	-	-	-	-	-	-	-	-	-	-
	Desi	2015-16	-	-	-	-	-	-	-	-	-	-	4240	-	-
			Kharif	-	-	-	-	-	-	-	-	-	-	-	-
	Hybrid	2015-16	-	-	-	-	-	-	-	-	-	-	-	-	-

Table 1 (Contd.) Crop-wise State-wise Weighted Average* of Farm Harvest Prices of Principal Crops in India

(Rs.per Quintal)

Crop Name	Variety	Year	Season $	Telangana	Tripura	Uttar Pradesh	Uttranchal	West Bengal						
Arecanut	-	2015-16	-	-	-	-	-	-						
			Kharif	-	-	-	-	-						
			Summer	-	-	-	-	-						
Bajra	-	2015-16	-	1539	-	-	-	-						
			Kharif	1863	-	1417	-	-						
			Rabi	1434	-	-	-	-						
			Summer	-	-	-	-	-						
Banana	-	2015-16	-	-	-	-	-	-						
Barley	-	2015-16	-	-	-	-	-	-						
			Rabi	-	-	1384	1561	-						
Cashewnut	-	2015-16	-	-	-	-	-	-						
			Summer	-	-	-	-	-						
	Raw	2015-16	-	-	-	-	-	-						
CasterSeed	-	2015-16	-	3286	-	-	-	-						
			Kharif	3189	-	-	-	-						
			Rabi	-	-	-	-	-						
Chilly	-	2015-16	-	-	-	-	-	-						
			Kharif	-	-	-	-	-						
			Rabi	-	-	-	-	-						
	Dry	2015-16	-	10343	-	-	-	-						
			Kharif	9742	-	-	-	-						
			Rabi	12360	-	-	-	-						
	Green	2015-16	-	-	-	-	-	-						
Coconut	-	2015-16	-	-	-	-	-	-						
			Kharif	-	-	-	-	-						
			Rabi	-	-	-	-	-						
			Summer	-	-	-	-	-						
Cotton	-	2015-16	-	3932	4400	-	-	-						
			Kharif	3939	-	5568	-	-						
			Rabi	3861	-	-	-	-						
			Summer	-	-	-	-	-						
	American	2015-16	-	-	-	-	-	-						
			Kharif	-	-	-	-	-						
	Desi	2015-16	-	-	-	-	-	-						
			Kharif	-	-	-	-	-						
	Hybrid	2015-16	-	-	-	-	-	-						

Table 1 (Contd.) Crop-wise State-wise Weighted Average* of Farm Harvest Prices of Principal Crops in India

(Rs.per Quintal)

Crop Name	Variety	Year	Season $	Andhra Pradesh	Andman & Nicobar	Assam	Bihar	Chandigarh	Chhattisgarh	Delhi	Goa	Gujarat	Haryana	Himachal Pradesh	Jammu & Kashmir
Cotton	Lint	2015-16	Kharif	-	-	-	-	-	-	-	-	-	-	-	-
Ginger	-	2015-16	-	-	12000	-	-	-	-	-	-	-	-	6396	-
			Kharif	-	-	-	-	-	-	-	-	-	-	-	-
			Rabi	-	-	-	3208	-	-	-	-	-	-	-	-
	Dry	2015-16	-	-	-	-	-	-	-	-	-	-	-	-	-
	Fresh	2015-16	-	-	-	-	-	-	6461	-	-	-	-	-	-
Gram	-	2015-16	-	4610	-	4445	-	-	5609	5000	-	3451	-	7226	-
			Kharif	-	-	-	-	-	-	-	-	-	-	-	-
			Rabi	4610	-	-	3624	-	-	-	-	-	-	-	-
Ground Nut	-	2015-16	-	4691	-	-	-	-	5786	-	-	-	-	7665	-
			Kharif	4950	-	-	6602	-	-	-	-	3681	3851	-	-
			Rabi	5107	-	-	-	-	-	-	-	-	-	-	-
			Summer	-	-	-	-	-	-	-	-	-	-	-	-
Jowar	-	2015-16	-	1582	-	-	-	-	-	1750	-	2031	-	-	-
			Kharif	1623	-	-	869	-	2299	-	-	-	3107	-	-
			Rabi	1575	-	-	-	-	1888	-	-	-	-	-	-
			Summer	-	-	-	-	-	-	-	-	-	-	-	-
	HYV	2015-16	Kharif	-	-	-	-	-	-	-	-	-	-	-	-
			Rabi	-	-	-	-	-	-	-	-	-	-	-	-
	Local	2015-16	Kharif	-	-	-	-	-	-	-	-	-	-	-	-
Jute	-	2015-16	-	-	-	1890	-	-	-	-	-	-	-	-	-
			Kharif	-	-	-	3276	-	-	-	-	-	-	-	-
Linseed	-	2015-16	-	-	-	2809	-	-	5440	-	-	-	-	6907	5555
			Rabi	-	-	-	3060	-	-	-	-	-	-	-	-
Maize	-	2015-16	-	1328	3200	1081	-	1241	1702	1720	-	1284	-	1713	1264
			Kharif	1340	-	-	1079	-	-	-	-	-	1600	-	-
			Rabi	1325	-	-	1135	-	-	-	-	-	-	-	-
	HB	2015-16	Rabi	-	-	-	-	-	-	-	-	-	-	-	-
			Summer	-	-	-	-	-	-	-	-	-	-	-	-
Mesta	-	2015-16	-	3353	-	1522	-	-	-	-	-	-	-	-	-
			Kharif	3353	-	-	1895	-	-	-	-	-	-	-	-
Nigerseed		2015-16	-	-	-	3597	-	-	5327	-	-	-	-	-	-
Paddy	-	2015-16	-	1503	2200	-	-	1451	-	1450	1020	1437	-	2084	1713
			Autum	-	-	1035	-	-	-	-	-	-	-	-	-
			Kharif	1515	-	-	-	-	-	-	-	-	1753	-	-
			Rabi	1488	-	-	-	-	-	-	-	-	-	-	-

Table 1 (Contd.) Crop-wise State-wise Weighted Average* of Farm Harvest Prices of Principal Crops in India

(Rs.per Quintal)

Crop Name	Variety	Year	Season $	Jharkhand	Karnataka	Kerala	Maharashtra	Manipur	Mizoram	Nagaland	Orissa	Pondicherry	Punjab	Rajasthan	Tamil Nadu
Cotton	Lint	2015-16	Kharif	-	-	-	-	-	-	-	-	-	-	11026	-
Ginger	-	2015-16	-	-	-	-	-	-	1558	3000	-	-	-	-	-
			Kharif	-	1048	-	-	-	-	-	-	-	-	5062	-
			Rabi	8759	-	-	-	3500	-	-	-	-	-	-	-
	Dry	2015-16	-	-	-	14452	-	-	-	-	-	-	-	-	-
	Fresh	2015-16	-	-	-	-	-	-	-	-	-	-	-	-	-
Gram	-	2015-16	-	-	-	-	4169	-	-	-	-	-	2808	-	5933
			Kharif	-	2756	-	-	-	-	-	-	-	-	-	-
			Rabi	5010	4530	-	-	-	-	-	-	-	-	4318	-
Ground Nut	-	2015-16	-	-	-	-	3585	-	-	-	3672	5339	4030	-	5242
			Kharif	-	4212	-	-	-	-	-	-	-	-	3878	-
			Rabi	-	4121	-	-	-	-	-	-	-	-	-	-
			Summer	-	4494	-	-	-	-	-	-	-	-	-	-
Jowar	-	2015-16	-	-	-	-	1763	-	-	-	-	-	-	-	-
			Kharif	-	-	-	-	-	-	-	-	-	-	1807	-
			Rabi	-	1367	-	-	-	-	-	-	-	-	-	-
			Summer	-	1394	-	-	-	-	-	-	-	-	-	-
	HYV	2015-16	Kharif	-	1662	-	-	-	-	-	-	-	-	-	-
			Rabi	-	1257	-	-	-	-	-	-	-	-	-	-
	Local	2015-16	Kharif	-	1918	-	-	-	-	-	-	-	-	-	-
Jute	-	2015-16	-	-	-	-	-	-	-	-	2920	-	-	-	-
			Kharif	-	-	-	-	-	-	-	-	-	-	-	-
Linseed	-	2015-16	-	-	-	-	4290	-	-	-	-	-	-	-	-
			Rabi	-	8500	-	-	-	-	-	-	-	-	3803	-
Maize	-	2015-16	-	-	-	-	1351	-	1440	1200	1205	-	1322	-	1620
			Kharif	-	1378	-	-	-	-	-	-	-	-	1422	-
			Rabi	1257	-	-	-	1425	-	-	-	-	-	-	-
	HB	2015-16	Rabi	-	1391	-	-	-	-	-	-	-	-	-	-
			Summer	-	1460	-	-	-	-	-	-	-	-	-	-
Mesta	-	2015-16	-	-	-	-	-	-	-	-	-	-	-	-	-
			Kharif	-	-	-	-	-	-	-	-	-	-	-	-
Nigerseed		2015-16	-	-	-	-	5249	-	-	-	-	-	-	-	-
Paddy	-	2015-16	-	-	-	1835	1660	-	1912	-	1335	-	-	-	1333
			Autum	-	-	-	-	-	-	-	-	-	-	-	-
			Kharif	-	-	-	-	-	-	-	-	-	-	1662	-
			Rabi	-	1530	-	-	1433	-	-	-	-	-	-	-

Table 1 (Contd.) Crop-wise State-wise Weighted Average* of Farm Harvest Prices of Principal Crops in India

(Rs.per Quintal)

Crop Name	Variety	Year	Season $	Telangana	Tripura	Uttar Pradesh	Uttranchal	West Bengal						
Cotton	Lint	2015-16	Kharif	-	-	-	-	-						
Ginger	-	2015-16	-	-	-	-	-	-						
			Kharif	-	-	-	-	-						
			Rabi	-	-	-	-	-						
	Dry	2015-16	-	-	-	-	-	-						
	Fresh	2015-16	-	-	-	-	-	-						
Gram	-	2015-16	-	4489	7615	-	-	5511						
			Kharif	-	-	-	-	-						
			Rabi	4461	-	5603	-	-						
Ground Nut	-	2015-16	-	4544	-	-	-	-						
			Kharif	5331	-	6162	5375	-						
			Rabi	4937	-	-	-	-						
			Summer	-	-	-	-	-						
Jowar	-	2015-16	-	2092	-	-	-	-						
			Kharif	2057	-	1426	-	-						
			Rabi	2394	-	-	-	-						
			Summer	-	-	-	-	-						
	HYV	2015-16	Kharif	-	-	-	-	-						
			Rabi	-	-	-	-	-						
	Local	2015-16	Kharif	-	-	-	-	-						
Jute	-	2015-16	-	-	2985	-	-	4448						
			Kharif	-	-	-	-	-						
Linseed	-	2015-16	-	-	-	-	-	-						
			Rabi	-	-	4238	-	-						
Maize	-	2015-16	-	1367	2637	-	-	-						
			Kharif	1475	-	1211	1495	-						
			Rabi	1324	-	-	-	-						
	HB	2015-16	Rabi	-	-	-	-	-						
			Summer	-	-	-	-	-						
Mesta	-	2015-16	-	-	-	-	-	-						
			Kharif	-	-	-	-	-						
Nigerseed		2015-16	-	-	-	-	-	-						
Paddy	-	2015-16	-	1439	1399	-	-	1526						
			Autum	-	-	-	-	-						
			Kharif	1427	-	1317	1390	-						
			Rabi	1450	-	-	-	-						

Table 1 (Contd.) Crop-wise State-wise Weighted Average* of Farm Harvest Prices of Principal Crops in India

(Rs.per Quintal)

Crop Name	Variety	Year	Season $	Andhra Pradesh	Andman & Nicobar	Assam	Bihar	Chandigarh	Chhattisgarh	Delhi	Goa	Gujarat	Haryana	Himachal Pradesh	Jammu & Kashmir
Paddy	-	2015-16	Summer	-	-	1041	-	-	-	-	-	-	-	-	-
			Winter	-	-	1099	-	-	-	-	-	-	-	-	-
	Bhadea	2015-16	Kharif	-	-	-	1114	-	-	-	-	-	-	-	-
	Coarse	2015-16	-	-	-	-	-	-	1299	-	-	-	-	-	-
	Fine	2015-16	-	-	-	-	-	-	1619	-	-	-	-	-	-
	Local	2015-16	Kharif	-	-	-	-	-	-	-	-	-	-	-	-
	Medium	2015-16	-	-	-	-	-	-	1392	-	-	-	-	-	-
Pepper	-	2015-16	-	-	80000	-	-	-	-	-	-	-	-	-	-
Potato	-	2015-16	-	-	-	1410	-	1050	-	1800	-	1075	-	-	1458
			Kharif	-	-	-	787	-	-	-	-	-	-	2262	-
			Rabi	-	-	-	766	-	-	-	-	-	-	1702	-
			Summer	-	-	-	-	-	-	-	-	-	-	-	-
	Hills	2015-16	Summer	-	-	-	-	-	1794	-	-	-	-	-	-
			Winter	-	-	-	-	-	1547	-	-	-	-	-	-
Ragi	-	2015-16	-	1591	-	-	-	-	1744	-	3500	1400	-	-	-
			Kharif	1609	-	-	1066	-	-	-	-	-	-	-	-
			Rabi	1428	-	-	-	-	-	-	-	-	-	-	-
	HYV	2015-16	Kharif	-	-	-	-	-	-	-	-	-	-	-	-
			Rabi	-	-	-	-	-	-	-	-	-	-	-	-
			Summer	-	-	-	-	-	-	-	-	-	-	-	-
Rape/Mustard	-	2015-16	-	-	8000	2831	-	-	4976	-	-	3622	-	5522	3757
			Rabi	-	-	-	3395	-	-	-	-	-	-	-	-
Rice	-	2015-16	-	-	-	-	-	-	-	-	-	-	-	-	-
			Kharif	-	-	-	-	-	-	-	-	-	-	-	-
	Coarse	2015-16	-	-	-	-	-	-	2265	-	-	-	-	-	-
	Fine	2015-16	-	-	-	-	-	-	4352	-	-	-	-	-	-
	Medium	2015-16	-	-	-	-	-	-	2774	-	-	-	-	-	-
Sannhemp	-	2015-16	-	-	-	-	-	-	4213	-	-	-	-	-	-
			Kharif	-	-	-	-	-	-	-	-	-	-	-	-
Sesamum	-	2015-16	-	5487	5000	6164	-	-	-	-	-	9202	-	10809	6252
			Kharif	4864	-	-	4652	-	10056	-	-	-	-	-	-
			Rabi	5843	-	-	5489	-	11337	-	-	-	-	-	-
			Summer	-	-	-	-	-	-	-	-	-	-	-	-
Soyabean	-	2015-16	-	-	-	-	-	-	3598	-	-	-	-	5334	-
			Kharif	-	-	-	-	-	-	-	-	-	-	-	-
Sugar Raw	-	2015-16	-	2590	-	3835	-	-	3903	-	-	-	-	-	-

Table 1 (Contd.) Crop-wise State-wise Weighted Average* of Farm Harvest Prices of Principal Crops in India

(Rs.per Quintal)

Crop Name	Variety	Year	Season $	Jharkhand	Karnataka	Kerala	Maharashtra	Manipur	Mizoram	Nagaland	Orissa	Pondicherry	Punjab	Rajasthan	Tamil Nadu
Paddy	-	2015-16	Summer	-	1743	-	-	-	-	-	-	-	-	-	-
			Winter	-	-	-	-	-	-	-	-	-	-	-	-
	Bhadea	2015-16	Kharif	-	-	-	-	-	-	-	-	-	-	-	-
	Coarse	2015-16	-	-	-	-	-	-	-	-	-	-	-	-	-
	Fine	2015-16	-	-	-	-	-	-	-	-	-	2170	-	-	-
	Local	2015-16	Kharif	-	1592	-	-	-	-	-	-	-	-	-	-
	Medium	2015-16	-	-	-	-	-	-	-	-	-	-	-	-	-
Pepper	-	2015-16	-	-	-	-	-	-	-	-	-	-	-	-	-
Potato	-	2015-16	-	-	-	-	-	-	1902	-	871	-	-	-	1828
			Kharif	-	952	-	-	-	-	-	-	-	-	-	-
			Rabi	1211	1714	-	-	1211	-	-	-	-	-	939	-
			Summer	-	1151	-	-	-	-	-	-	-	-	-	-
	Hills	2015-16	Summer	-	-	-	-	-	-	-	-	-	-	-	-
			Winter	-	-	-	-	-	-	-	-	-	-	-	-
Ragi	-	2015-16	-	-	-	-	1837	-	-	-	1511	1642	-	-	2056
			Kharif	-	-	-	-	-	-	-	-	-	-	-	-
			Rabi	-	-	-	-	-	-	-	-	-	-	-	-
	HYV	2015-16	Kharif	-	1606	-	-	-	-	-	-	-	-	-	-
			Rabi	-	1607	-	-	-	-	-	-	-	-	-	-
			Summer	-	1639	-	-	-	-	-	-	-	-	-	-
Rape/Mustard	-	2015-16	-	-	-	-	3656	-	3534	-	4195	-	-	-	-
			Rabi	4326	-	-	-	3000	-	-	-	-	-	3826	-
Rice	-	2015-16	-	-	-	-	-	-	2433	-	-	-	-	-	-
			Kharif	-	-	-	-	-	-	-	-	-	-	2274	-
	Coarse	2015-16	-	-	-	-	-	-	-	-	-	-	-	-	-
	Fine	2015-16	-	-	-	-	-	-	-	-	-	-	-	-	-
	Medium	2015-16	-	-	-	-	-	-	-	-	-	-	-	-	-
Sannhemp	-	2015-16	-	-	-	-	-	-	-	-	-	-	-	-	-
			Kharif	-	-	-	-	-	-	-	-	-	-	2429	-
Sesamum	-	2015-16	-	-	-	-	6625	-	5525	-	4684	3868	9000	-	7491
			Kharif	-	5760	-	-	-	-	-	-	-	-	7046	-
			Rabi	-	4550	-	-	-	-	-	-	-	-	-	-
			Summer	-	2300	-	-	-	-	-	-	-	-	-	-
Soyabean	-	2015-16	-	-	-	-	3490	-	6474	-	-	-	-	-	-
			Kharif	-	-	-	-	-	-	-	-	-	-	-	-
Sugar Raw	-	2015-16	-	-	-	-	-	-	-	-	-	-	-	-	-

Table 1 (Contd.) Crop-wise State-wise Weighted Average* of Farm Harvest Prices of Principal Crops in India

(Rs.per Quintal)

Crop Name	Variety	Year	Season $	Telangana	Tripura	Uttar Pradesh	Uttranchal	West Bengal						
Paddy	-	2015-16	Summer	-	-	-	-	-						
			Winter	-	-	-	-	-						
	Bhadea	2015-16	Kharif	-	-	-	-	-						
	Coarse	2015-16	-	-	-	-	-	-						
	Fine	2015-16	-	-	-	-	-	-						
	Local	2015-16	Kharif	-	-	-	-	-						
	Medium	2015-16	-	-	-	-	-	-						
Pepper	-	2015-16	-	-	-	-	-	-						
Potato	-	2015-16	-	-	968	-	-	1296						
			Kharif	-	-	-	-	-						
			Rabi	-	-	688	-	-						
			Summer	-	-	-	-	-						
	Hills	2015-16	Summer	-	-	-	-	-						
			Winter	-	-	-	-	-						
Ragi	-	2015-16	-	1644	-	-	-	-						
			Kharif	1614	-	-	2964	-						
			Rabi	1678	-	-	-	-						
	HYV	2015-16	Kharif	-	-	-	-	-						
			Rabi	-	-	-	-	-						
			Summer	-	-	-	-	-						
Rape/Mustard	-	2015-16	-	-	6589	-	-	4147						
			Rabi	-	-	3623	3495	-						
Rice	-	2015-16	-	-	-	-	-	-						
			Kharif	-	-	-	-	-						
	Coarse	2015-16	-	-	-	-	-	-						
	Fine	2015-16	-	-	-	-	-	-						
	Medium	2015-16	-	-	-	-	-	-						
Sannhemp	-	2015-16	-	-	-	-	-	-						
			Kharif	-	-	-	-	-						
Sesamum	-	2015-16	-	6711	6738	-	-	-						
			Kharif	7241	-	6365	7381	-						
			Rabi	6695	-	-	-	-						
			Summer	-	-	-	-	-						
Soyabean	-	2015-16	-	-	-	-	-	-						
			Kharif	-	-	2640	3379	-						
Sugar Raw	-	2015-16	-	3164	-	-	-	-						

Table 1 (Contd.) Crop-wise State-wise Weighted Average* of Farm Harvest Prices of Principal Crops in India

(Rs.per Quintal)

Crop Name	Variety	Year	Season $	Andhra Pradesh	Andman & Nicobar	Assam	Bihar	Chandigarh	Chhattisgarh	Delhi	Goa	Gujarat	Haryana	Himachal Pradesh	Jammu & Kashmir
Sugar Raw	-	2015-16	Kharif	2590	-	-	-	-	-	-	-	-	-	-	-
			Rabi	-	-	-	-	-	-	-	-	-	-	-	-
Sugarcane	-	2015-16	-	-	2313	1044	-	-	-	-	250	-	-	-	1450
			Kharif	-	-	-	-	-	-	-	-	-	-	-	-
			Rabi	-	-	-	-	-	-	-	-	-	-	-	-
			Summer	-	-	-	-	-	-	-	-	-	-	-	-
Sunflower	-	2015-16	-	3439	-	-	-	-	-	-	-	-	-	-	-
			Kharif	3572	-	-	-	-	-	-	-	-	-	-	-
			Rabi	3406	-	-	-	-	-	-	-	-	-	-	-
			Summer	-	-	-	-	-	-	-	-	-	-	-	-
Tapioca	-	2015-16	-	-	2389	-	-	-	-	-	-	-	-	-	-
Tobacco	-	2015-16	-	12811	-	8510	-	-	11169	-	-	4611	-	-	-
			Kharif	-	-	-	-	-	-	-	-	-	-	-	-
			Rabi	12811	-	-	-	-	-	-	-	-	-	-	-
			Summer	-	-	-	-	-	-	-	-	-	-	-	-
Tur Arhar	-	2015-16	-	8073	9000	6725	-	-	-	-	-	4841	-	-	-
			Kharif	8073	-	-	-	-	-	-	-	-	-	-	-
			Rabi	-	-	-	3625	-	-	-	-	-	-	-	-
	Delay	2015-16	-	-	-	-	-	-	8436	-	-	-	-	-	-
	Early	2015-16	-	-	-	-	-	-	7990	-	-	-	-	-	-
Turmeric	-	2015-16	-	7717	11000	-	-	-	9611	-	-	-	-	10404	-
			Kharif	7717	-	-	-	-	-	-	-	-	-	-	-
			Rabi	-	-	-	8019	-	-	-	-	-	-	-	-
			Summer	-	-	-	-	-	-	-	-	-	-	-	-
	Dry	2015-16	-	-	-	10996	-	-	-	-	-	-	-	-	-
Wheat	-	2015-16	-	-	-	1081	-	1580	2102	1525	-	1608	-	1885	1505
			Rabi	-	-	-	1284	-	-	-	-	-	-	-	-
			Summer	-	-	-	-	-	-	-	-	-	-	-	-

Table 1 (Contd.) Crop-wise State-wise Weighted Average* of Farm Harvest Prices of Principal Crops in India

(Rs.per Quintal)

Crop Name	Variety	Year	Season $	Jharkhand	Karnataka	Kerala	Maharashtra	Manipur	Mizoram	Nagaland	Orissa	Pondicherry	Punjab	Rajasthan	Tamil Nadu
Sugar Raw	-	2015-16	Kharif	-	-	-	-	-	-	-	-	-	-	-	-
			Rabi	-	-	-	-	-	-	-	-	-	-	3323	-
Sugarcane	-	2015-16	-	-	-	-	-	-	921	-	240	-	300	-	-
			Kharif	-	2600	-	-	-	-	-	-	-	-	-	-
			Rabi	-	-	-	-	840	-	-	-	-	-	-	-
			Summer	-	1728	-	-	-	-	-	-	-	-	-	-
Sunflower	-	2015-16	-	-	-	-	3446	-	-	-	-	-	-	-	-
			Kharif	-	3401	-	-	-	-	-	-	-	-	-	-
			Rabi	-	3302	-	-	-	-	-	-	-	-	-	-
			Summer	-	3501	-	-	-	-	-	-	-	-	-	-
Tapioca	-	2015-16	-	-	-	1172	-	-	-	-	-	-	-	-	1561
Tobacco	-	2015-16	-	-	-	-	-	-	20922	-	-	-	-	-	-
			Kharif	-	-	-	-	-	-	-	-	-	-	-	-
			Rabi	-	-	-	-	-	-	-	-	-	-	-	-
			Summer	-	10172	-	-	-	-	-	-	-	-	-	-
Tur Arhar	-	2015-16	-	-	-	-	7741	-	3609	-	-	-	-	-	-
			Kharif	-	6134	-	-	-	-	-	-	-	-	-	-
			Rabi	6816	-	-	-	-	-	-	-	-	-	6425	-
	Delay	2015-16	-	-	-	-	-	-	-	-	-	-	-	-	-
	Early	2015-16	-	-	-	-	-	-	-	-	-	-	-	-	-
Turmeric	-	2015-16	-	-	-	-	-	-	1163	-	-	-	-	-	10074
			Kharif	-	8056	-	-	-	-	-	-	-	-	-	-
			Rabi	1000	-	-	-	-	-	-	-	-	-	-	-
			Summer	-	8056	-	-	-	-	-	-	-	-	-	-
	Dry	2015-16	-	-	-	-	-	-	-	-	-	-	-	-	-
Wheat	-	2015-16	-	-	-	-	1706	-	-	-	1651	-	1667	-	-
			Rabi	1631	1930	-	-	-	-	-	-	-	-	1596	-
			Summer	-	1493	-	-	-	-	-	-	-	-	-	-

Table 1 (Contd.) Crop-wise State-wise Weighted Average* of Farm Harvest Prices of Principal Crops in India

(Rs.per Quintal)

Crop Name	Variety	Year	Season $	Telangana	Tripura	Uttar Pradesh	Uttranchal	West Bengal						
Sugar Raw	-	2015-16	Kharif	3036	-	2955	3522	-						
			Rabi	-	-	-	-	-						
Sugarcane	-	2015-16	-	-	-	-	-	-						
			Kharif	-	-	248	286	-						
			Rabi	-	-	-	-	-						
			Summer	-	-	-	-	-						
Sunflower	-	2015-16	-	-	-	-	-	-						
			Kharif	-	-	-	-	-						
			Rabi	-	-	-	-	-						
			Summer	-	-	-	-	-						
Tapioca	-	2015-16	-	-	-	-	-	-						
Tobacco	-	2015-16	-	14275	-	-	-	-						
			Kharif	14275	-	-	-	-						
			Rabi	14275	-	3732	-	-						
			Summer	-	-	-	-	-						
Tur Arhar	-	2015-16	-	8609	-	-	-	-						
			Kharif	8615	-	-	-	-						
			Rabi	7565	-	-	-	-						
	Delay	2015-16	-	-	-	-	-	-						
	Early	2015-16	-	-	-	-	-	-						
Turmeric	-	2015-16	-	7914	-	-	-	-						
			Kharif	7937	-	-	-	-						
			Rabi	-	-	3354	-	-						
			Summer	-	-	-	-	-						
	Dry	2015-16	-	-	-	-	-	-						
Wheat	-	2015-16	-	-	-	-	-	1690						
			Rabi	-	-	1438	1629	-						
			Summer	-	-	-	-	-						

* State Weighted Average Provided by State Authority. $ The icon (-) is given wherever state has not mentioned specific season.

Table 2.1 Crop-wise District-wise Farm Harvest Prices of Principal Crops in India - Andhra Pradesh

(Rs.per Quintal)

Crop Name	Variety	Year	Season $	Anantapur	Chittor	East Godavari	Guntur	Kadapa	Krishna	Kurnool	Nellore	Prakasam	Srikakulam	Visakhapatnam	State Wtd Average
Bajra	-	2015-16	-	1724	1352	-	1317	1365	-	1376	-	1283	-	2178	1384
			Kharif	1724	1352	-	1317	1402	-	1376	-	1283	-	2178	1387
			Rabi	-	-	-	-	1327	-	-	-	-	-	-	1327
CasterSeed	-	2015-16	-	2383	-	-	2752	3487	-	3500	-	2693	-	-	3011
			Kharif	2383	-	-	2752	3487	-	3500	-	2693	-	-	3011
Chilly	Dry	2015-16	-	13587	13123	-	11225	-	11305	10947	11313	11413	10217	-	11321
			Kharif	13587	-	-	11186	-	11280	10947	-	11098	-	-	11204
			Rabi	-	13123	-	12258	-	11875	-	11313	12418	10217	-	12045
Cotton	-	2015-16	-	4346	-	3787	4049	4145	3904	4064	3449	4098	3810	3649	4035
			Kharif	4346	-	3787	4049	4145	3904	4064	3449	4098	3810	3649	4034
Gram	-	2015-16	-	4534	-	-	4762	4580	4198	4503	4514	4724	-	-	4610
			Rabi	-	-	-	4762	45804503	4198	4534	4514	4724	-	-	4610
Ground Nut	-	2015-16	-	4740	4158	-	5342	4816	5094	4849	5674	5219	4815	3342	4691
			Kharif	4676	3934	-	-	4534	4852	4805	5551	5260	4758	3342	4950
			Rabi	5364	5038	-	5342	5036	5410	4956	5776	5178	4860	-	5107
Jowar	-	2015-16	-	1801	-	-	1325	1773	-	1689	1316	1390	-	-	1582
			Kharif	1793	-	-	-	1411	-	1617	-	-	-	-	1623
			Rabi	1855	-	-	1325	1865	-	1702	1316	1390	-	-	1575
Maize	-	2015-16	-	1281	1293	1317	1367	1443	1369	1343	1375	1364	1274	1259	1328
			Kharif	1284	1293	-	1393	-	1328	1352	-	1818	1291	1260	1340
			Rabi	1273	-	1317	1367	1443	1375	1341	1375	1344	1252	1257	1325
Mesta	-	2015-16	-	-	-	-	-	-	-	-	-	-	3396	-	3353
			Kharif	-	-	-	-	-	-	-	-	-	3396	-	3353
Paddy	-	2015-16	-	1878	1389	1376	1812	1917	1601	1890	1577	1580	1390	1380	1503
			Kharif	2143	1271	1364	1817	1976	1608	1928	1330	1849	1392	1381	1515
			Rabi	1438	1402	1387	1562	1877	1472	1675	1600	1462	1289	1350	1488
Ragi	-	2015-16	-	2003	1843	-	-	-	-	-	-	1428	-	1465	1591
			Kharif	2003	1843	-	-	-	-	-	-	-	-	1465	1609
			Rabi	-	-	-	-	-	-	-	-	1428	-	-	1428
Sesamum	-	2015-16	-	-	-	5906	5592	5778	-	6067	5838	5534	5221	5958	5487
			Kharif	-	-	-	5470	-	-	-	-	5596	5221	5230	4864
			Rabi	-	-	5906	6010	5778	-	6067	5838	5483	-	6364	5843
Sugar Raw	-	2015-16	-	-	2331	3183	-	-	-	-	-	-	3352	2167	2590
			Kharif	-	2331	3183	-	-	-	-	-	-	3352	2167	2590
Sunflower	-	2015-16	-	3606	3492	-	-	3604	-	3543	3222	2848	-	-	3439
			Kharif	3600	-	-	-	3820	-	3505	-	-	-	-	3572

Table 2.1 (Contd.) Crop-wise District-wise Farm Harvest Prices of Principal Crops in India - Andhra Pradesh

(Rs.per Quintal)

Crop Name	Variety	Year	Season $	Vizianagaram	West Godavari	State Wtd Average
Bajra	-	2015-16	-	-	-	1384
			Kharif	-	-	1387
			Rabi	-	-	1327
CasterSeed	-	2015-16	-	-	-	3011
			Kharif	-	-	3011
Chilly	Dry	2015-16	-	-	11730	11321
			Kharif	-	-	11204
			Rabi	-	11730	12045
Cotton	-	2015-16	-	3604	4010	4035
			Kharif	3604	4010	4034
Gram	-	2015-16	-	-	-	4610
			Rabi	-	-	4610
Ground Nut	-	2015-16	-	4462	4209	4691
			Kharif	4836	-	4950
			Rabi	4072	4209	5107
Jowar	-	2015-16	-	-	-	1582
			Kharif	-	-	1623
			Rabi	-	-	1575
Maize	-	2015-16	-	1410	1261	1328
			Kharif	1422	1247	1340
			Rabi	1404	1262	1325
Mesta	-	2015-16	-	3323	-	3353
			Kharif	3323	-	3353
Paddy	-	2015-16	-	1408	1412	1503
			Kharif	1409	1400	1515
			Rabi	1363	1423	1488
Ragi	-	2015-16	-	1478	-	1591
			Kharif	1478	-	1609
			Rabi	-	-	1428
Sesamum	-	2015-16	-	4959	-	5487
			Kharif	4293	-	4864
			Rabi	5861	-	5843
Sugar Raw	-	2015-16	-	2501	2872	2590
			Kharif	2501	2872	2590
Sunflower	-	2015-16	-	-	-	3439
			Kharif	-	-	3572

Table 2.1 (Contd.) Crop-wise District-wise Farm Harvest Prices of Principal Crops in India - Andhra Pradesh

(Rs.per Quintal)

Crop Name	Variety	Year	Season $	Anantapur	Chittor	East Godavari	Guntur	Kadapa	Krishna	Kurnool	Nellore	Prakasam	Srikakulam	Visakhapatnam	State Wtd Average
Sunflower	-	2015-16	Rabi	3616	3492	-	-	3595	-	3583	3222	2848	-	-	3406
Tobacco	-	2015-16	-	-	-	11162	-	-	-	-	13713	12907	-	-	12811
			Rabi	-	-	11162	-	-	-	-	13713	12907	-	-	12811
Tur Arhar	-	2015-16	-	7503	6520	-	8723	6379	8197	8636	-	7898	-	6257	8073
			Kharif	7503	6520	-	8723	6379	8197	8636	-	7898	-	6257	8073
Turmeric	-	2015-16	-	-	-	-	7916	8116	7727	6995	-	-	-	7785	7717
			Kharif	-	-	-	7916	8116	7727	6995	-	-	-	7785	7717

Table 2.1 (Contd.) Crop-wise District-wise Farm Harvest Prices of Principal Crops in India - Andhra Pradesh

(Rs.per Quintal)

Crop Name	Variety	Year	Season $	Vizianagaram	West Godavari	State Wtd Average									
Sunflower	-	2015-16	Rabi	-	-	3406									
Tobacco	-	2015-16	-	-	12693	12811									
			Rabi	-	12693	12811									
Tur Arhar	-	2015-16	-	5285	-	8073									
			Kharif	5285	-	8073									
Turmeric	-	2015-16	-	-	-	7717									
			Kharif	-	-	7717									

Table 2.2 Crop-wise District-wise Farm Harvest Prices of Principal Crops in India - Andman & Nicobar

(Rs.per Quintal)

Crop Name	Variety	Year	Season $	Campbellba y	Car-Nicobar	Diglipur	Ferrargunj	Mayabunder	Nancowrie	Portblair	Rangat	State Wtd Average			
Arecanut	-	2015-16	-	18000	7000	20000	17500	20000	20000	18000	18000	17500			
Cashewnut	Raw	2015-16	-	-	-	4000	8000	4000	-	4000	-	5000			
Chilly	Green	2015-16	-	12000	15000	8000	9000	8000	-	10000	8000	11250			
Ginger	-	2015-16	-	20000	-	5000	9000	15000	12000	11000	16000	12000			
Maize	-	2015-16	-	-	-	3000	3000	5000	-	2000	-	3200			
Paddy	-	2015-16	-	-	-	2500	2000	2500	-	2000	2000	2200			
Pepper	-	2015-16	-	-	-	75000	75000	80000	80000	80000	90000	80000			
Rape/Mustard	-	2015-16	-	-	-	8000	-	8000	-	-	-	8000			
Sesamum	-	2015-16	-	-	-	-	-	5000	-	-	-	5000			
Sugarcane	-	2015-16	-	-	2000	1500	2000	4000	2500	1500	3000	2313			
Tapioca	-	2015-16	-	3000	3500	2000	1500	2000	3000	2000	2500	2389			
Tur Arhar	-	2015-16	-	-	-	9000	-	-	-	-	-	9000			
Turmeric	-	2015-16	-	-	-	10000	10000	10000	-	12000	9000	11000			

Table 2.3 Crop-wise District-wise Farm Harvest Prices of Principal Crops in India - Assam

(Rs.per Quintal)

Crop Name	Variety	Year	Season $	Barpeta	Bongaigaon	Cachar	Darrang	Dhubri	Dibrugarh	Goalpara	Golaghat	Hailkandi	Jorhat	Kamrup	State Wtd Average
Arecanut	-	2015-16	-	2785	-	3097	3168	2925	2991	3005	3018	2783	3026	2832	2961
CasterSeed	-	2015-16	-	-	-	-	-	-	-	-	-	-	-	-	7500
Cotton	-	2015-16	-	4657	-	-	-	-	-	-	-	-	-	-	4693
Gram	-	2015-16	-	4541	-	-	-	4504	-	-	-	-	4639	4299	4445
Jute	-	2015-16	-	1653	2034	-	1950	1932	-	1948	-	-	-	1788	1890
Linseed	-	2015-16	-	2688	-	-	2772	-	-	-	-	-	-	-	2809
Maize	-	2015-16	-	1118	-	1151	-	-	-	-	-	-	-	1117	1081
Mesta	-	2015-16	-	-	-	-	1470	-	-	-	-	-	-	-	1522
Nigerseed		2015-16	-	3562	-	-	-	-	-	-	-	-	-	-	3597
Paddy	-	2015-16	Autum	-	1011	-	10077	-	-	1000	-	995	989	1031	1035
			Summer	1050	1056	-	1028	1055	-	1040	-	1086	972	1043	1041
			Winter	1133	1101	1101	1121	-	1075	1100	1092	1080	1114	1116	1099
Potato	-	2015-16	-	1552	1387	1405	1378	1417	1542	1555	1422	1323	1340	1397	1410
Rape/Mustard	-	2015-16	-	2891	2814	-	2706	2574	3157	2676	2900	2995	2741	3120	2831
Sesamum	-	2015-16	-	5839	-	-	6179	6095	5919	6537	6218	-	-	6001	6164
Sugar Raw	-	2015-16	-	-	-	3876	4085	-	-	-	-	-	4379	3880	3835
Sugarcane	-	2015-16	-	-	-	930	952	-	-	-	-	-	1067	1182	1044
Tobacco	-	2015-16	-	8444	-	-	-	-	-	-	9864	-	8319	-	8510
Tur Arhar	-	2015-16	-	6634	6874	-	-	6867	-	-	-	-	-	6580	6725
Turmeric	Dry	2015-16	-	11024	12353	-	11707	12508	-	9183	11163	-	10036	12061	10996
Wheat	-	2015-16	-	1508	1370	-	1436	-	-	1464	-	-	-	1468	1081

Table 2.3 (Contd.) Crop-wise District-wise Farm Harvest Prices of Principal Crops in India - Assam

(Rs.per Quintal)

Crop Name	Variety	Year	Season $	Karbi Anglong	Karimganj	Kokrajhar	Lakhimpur	Morigaon	Nagaon	Nalbari	North Cachar Hills	Sivasagar	Sonitpur	Tinsukia	State Wtd Average
Arecanut	-	2015-16	-	2780	-	3068	2880	2992	3092	3239	2762	3019	3057	-	2961
CasterSeed	-	2015-16	-	-	-	-	-	7500	-	-	-	-	-	-	7500
Cotton	-	2015-16	-	-	-	-	-	-	-	-	-	-	4719	-	4693
Gram	-	2015-16	-	4397	-	-	-	-	-	-	-	-	-	-	4445
Jute	-	2015-16	-	-	-	1891	-	1939	1887	1651	-	1961	1884	-	1890
Linseed	-	2015-16	-	3906	-	-	-	-	3167	-	-	-	2695	-	2809
Maize	-	2015-16	-	1060	-	1062	-	-	1308	-	-	-	-	-	1081
Mesta	-	2015-16	-	-	-	-	-	-	1441	-	-	-	1601	-	1522
Nigerseed		2015-16	-	-	-	3672	-	3585	-	-	-	-	-	-	3597
Paddy	-	2015-16	Autum	-	-	1060	-	-	-	-	-	-	1042	-	1035
			Summer	-	-	-	-	1027	1034	1123	-	-	1022	-	1041
			Winter	1113	1099	1116	1097	1083	1121	1120	-	1081	1080	-	1099
Potato	-	2015-16	-	1454	-	1427	1418	1463	1495	1211	1494	1416	1278	-	1410
Rape/Mustard	-	2015-16	-	3271	-	2897	2896	2609	2748	-	2760	-	2544	3088	2831
Sesamum	-	2015-16	-	6288	-	6070	6122	6080	5821	6123	-	-	-	-	6164
Sugar Raw	-	2015-16	-	3762	4162	-	4125	3751	3657	3859	-	3998	3911	-	3835
Sugarcane	-	2015-16	-	1010	1102	-	1111	928	1101	930	-	1034	1090	-	1044
Tobacco	-	2015-16	-	8339	-	-	-	-	-	-	-	-	-	-	8510
Tur Arhar	-	2015-16	-	6738	-	-	-	-	-	-	-	-	-	-	6725
Turmeric	Dry	2015-16	-	10935	12384	11165	11277	9681	9158	9473	12988	-	-	-	10996
Wheat	-	2015-16	-	1446	-	-	-	1370	1532	-	-	-	-	-	1081

Table 2.4 Crop-wise District-wise Farm Harvest Prices of Principal Crops in India - Bihar

(Rs.per Quintal)

Crop Name	Variety	Year	Season $	Aurangabad	Jahanabad	Araia	Arbal	Banka	Begusarai	Bhabua (Kymmur)	Bhagalpur	Bhojpur (Aara)	Buxar	Darbhanga	State Wtd Average
Bajra	-	2015-16	Kharif	-	-	-	-	-	-	-	1177	1177	1177	-	1177
Barley	-	2015-16	Rabi	1144	1144	-	1125	1100	1144	1010	1100	1010	1010	1144	1144
Chilly	-	2015-16	Rabi	-	5500	5691	5500	-	5500	-	5691	5691	-	-	5691
Ginger	-	2015-16	Rabi	-	-	-	-	-	-	-	-	-	-	-	3208
Gram	-	2015-16	Rabi	3650	3650	3624	3650	3624	3980	3105	3624	3105	3105	3624	3624
Ground Nut	-	2015-16	Kharif	6602	-	-	-	-	6602	-	-	-	-	-	6602
Jowar	-	2015-16	Kharif	-	-	-	-	-	869	-	869	869	872	-	869
Jute	-	2015-16	Kharif	-	-	3550	-	-	-	-	-	-	-	-	3276
Linseed	-	2015-16	Rabi	3060	3060	-	3060	3100	2911	3060	3100	3060	3060	3060	3060
Maize	-	2015-16	Kharif	1079	1079	1037	1079	1057	937	1079	1057	1045	1275	1079	1079
			Rabi	920	920	1148	920	1135	1150	-	1135	1135	-	1135	1135
Mesta	-	2015-16	Kharif	-	-	2100	-	-	1895	-	1895	-	-	-	1895
Paddy	Bhadea	2015-16	Kharif	-	-	1097	-	-	1200	-	1114	1114	1114	1114	1114
Potato	-	2015-16	Kharif	800	787	787	787	787	750	900	787	816	787	700	787
			Rabi	655	655	667	655	766	800	-	766	766	766	766	766
Ragi	-	2015-16	Kharif	995	995	-	995	-	1066	-	-	-	-	1066	1066
Rape/Mustard	-	2015-16	Rabi	3250	3250	3395	3250	3600	3395	3395	3600	3395	3320	3395	3395
Sesamum	-	2015-16	Kharif	4652	-	-	-	-	4652	-	-	-	-	-	4652
			Rabi	5489	-	-	5489	-	5489	-	5489	5489	-	-	5489
Tur Arhar	-	2015-16	Rabi	3625	3625	3625	3625	3625	3625	3500	3625	3500	3500	3625	3625
Turmeric	-	2015-16	Rabi	7000	-	-	7000	-	8000	-	8019	-	-	-	8019
Wheat	-	2015-16	Rabi	1284	1284	1331	1284	1200	1284	1155	1200	1155	1155	1367	1284

Table 2.4 (Contd.) Crop-wise District-wise Farm Harvest Prices of Principal Crops in India - Bihar

(Rs.per Quintal)

Crop Name	Variety	Year	Season $	East Champaran	Gaya	Gopalganj	Jumui	Katihar	Khagaria	Kishanganj	Lakhisarai	Madhepura	Madhubani	Munger	State Wtd Average
Bajra	-	2015-16	Kharif	-	-	1177	-	-	-	-	-	-	-	-	1177
Barley	-	2015-16	Rabi	-	1300	-	1144	1144	1144	-	-	-	1144	-	1144
Chilly	-	2015-16	Rabi	5000	5500	-	-	5691	5500	5691	-	5800	5691	-	5691
Ginger	-	2015-16	Rabi	3090	-	-	-	-	3208	3208	-	3180	-	-	3208
Gram	-	2015-16	Rabi	-	3650	-	3980	3624	3980	3624	3980	-	3624	3980	3624
Ground Nut	-	2015-16	Kharif	-	6700	-	-	-	-	-	-	6602	-	-	6602
Jowar	-	2015-16	Kharif	-	-	-	-	-	-	-	-	-	-	-	869
Jute	-	2015-16	Kharif	-	-	-	-	3149	-	3276	-	3276	-	-	3276
Linseed	-	2015-16	Rabi	3225	3060	-	2911	3060	2911	3060	2911	3060	3060	2911	3060
Maize	-	2015-16	Kharif	1050	1300	1079	1079	1079	1079	1079	1079	1079	1079	1079	1079
			Rabi	1139	920	1011	1175	1135	1135	1095	1135	1135	1135	1135	1135
Mesta	-	2015-16	Kharif	-	-	-	-	2100	1895	2100	-	1895	1895	-	1895
Paddy	Bhadea	2015-16	Kharif	1114	-	1135	-	1114	955	922	-	1114	1200	1114	1114
Potato	-	2015-16	Kharif	787	730	848	750	787	787	846	750	787	700	750	787
			Rabi	800	655	665	766	667	766	667	766	766	766	817	766
Ragi	-	2015-16	Kharif	-	995	1225	-	-	-	1066	-	940	1066	-	1066
Rape/Mustard	-	2015-16	Rabi	3000	3250	3395	3395	3395	3395	3395	3395	3395	3395	3395	3395
Sesamum	-	2015-16	Kharif	-	3850	-	-	-	-	4652	-	4652	4652	-	4652
			Rabi	5500	-	-	-	-	-	5489	-	5483	5489	-	5489
Tur Arhar	-	2015-16	Rabi	3200	3150	3392	3625	3625	3625	-	3625	3625	3625	3625	3625
Turmeric	-	2015-16	Rabi	8019	7000	-	-	8019	8000	8019	-	8200	8019	-	8019
Wheat	-	2015-16	Rabi	1410	1284	1284	1284	1284	1284	1352	1284	1250	1367	1284	1284

Table 2.4 (Contd.) Crop-wise District-wise Farm Harvest Prices of Principal Crops in India - Bihar

(Rs.per Quintal)

Crop Name	Variety	Year	Season $	Muzaffarpur	Nalanda	Nawada	Patna	Purnia	Rohtas	Saharsa	Samastipur	Saran (Chapra)	Sheikhpura	Sheohar	State Wtd Average
Bajra	-	2015-16	Kharif	-	-	-	-	-	-	-	-	-	-	-	1177
Barley	-	2015-16	Rabi	1144	1010	-	1010	-	1010	1144	1144	1144	-	-	1144
Chilly	-	2015-16	Rabi	5000	5691	-	5691	5691	5691	-	5691	-	5500	5000	5691
Ginger	-	2015-16	Rabi	3090	-	-	-	-	3208	-	5000	-	-	3090	3208
Gram	-	2015-16	Rabi	3624	3105	3650	3105	-	3105	-	3624	3624	3980	-	3624
Ground Nut	-	2015-16	Kharif	-	6446	5690	-	-	6446	-	-	-	-	-	6602
Jowar	-	2015-16	Kharif	-	-	-	860	-	-	-	869	-	-	-	869
Jute	-	2015-16	Kharif	-	-	-	-	3276	-	3276	3276	-	-	-	3276
Linseed	-	2015-16	Rabi	3225	3060	3060	3060	3060	3060	3060	3060	3060	2911	3225	3060
Maize	-	2015-16	Kharif	1057	1074	1085	1079	948	1079	1073	1079	1079	1194	1079	1079
			Rabi	1139	1135	-	1135	1135	1135	1135	1135	1011	1135	1139	1135
Mesta	-	2015-16	Kharif	1895	-	-	-	2100	-	2060	1895	-	-	-	1895
Paddy	Bhadea	2015-16	Kharif	1045	-	1114	1114	837	1114	1060	1100	1135	-	1114	1114
Potato	-	2015-16	Kharif	787	787	750	787	803	787	787	700	848	750	787	787
			Rabi	766	805	655	766	667	755	766	766	665	766	766	766
Ragi	-	2015-16	Kharif	1260	1066	995	1066	-	-	940	1066	1066	-	1260	1066
Rape/Mustard	-	2015-16	Rabi	3000	3564	3250	3395	3395	3395	3395	3395	3395	3395	3000	3395
Sesamum	-	2015-16	Kharif	-	-	4650	-	-	-	4900	4652	4652	-	4652	4652
			Rabi	5500	-	-	5489	-	-	5483	5489	-	5489	5500	5489
Tur Arhar	-	2015-16	Rabi	3200	3500	4810	3500	3625	3500	-	3625	3392	3625	3200	3625
Turmeric	-	2015-16	Rabi	8019	8019	-	8019	-	8019	-	8019	-	8000	8019	8019
Wheat	-	2015-16	Rabi	1360	1155	1284	1155	1326	1155	1250	1367	1284	1284	1284	1284

Table 2.4 (Contd.) Crop-wise District-wise Farm Harvest Prices of Principal Crops in India - Bihar

(Rs.per Quintal)

Crop Name	Variety	Year	Season $	Sitamarhi	Siwan	Supaul	Vaishali	West Champaran	State Wtd Average						
Bajra	-	2015-16	Kharif	-	1177	-	-	-	1177						
Barley	-	2015-16	Rabi	1144	1144	-	1144	1144	1144						
Chilly	-	2015-16	Rabi	5000	5691	5800	5000	5000	5691						
Ginger	-	2015-16	Rabi	-	3208	3180	-	3090	3208						
Gram	-	2015-16	Rabi	-	3624	-	3624	3624	3624						
Ground Nut	-	2015-16	Kharif	-	-	-	-	6602	6602						
Jowar	-	2015-16	Kharif	-	-	-	-	-	869						
Jute	-	2015-16	Kharif	-	-	3276	-	-	3276						
Linseed	-	2015-16	Rabi	3225	3060	3060	3225	3225	3060						
Maize	-	2015-16	Kharif	1101	1079	1200	1295	1079	1079						
			Rabi	1139	1011	1135	1139	1139	1135						
Mesta	-	2015-16	Kharif	-	1895	1880	1895	1895	1895						
Paddy	Bhadea	2015-16	Kharif	1188	1135	1150	1114	1114	1114						
Potato	-	2015-16	Kharif	-	848	787	787	787	787						
			Rabi	766	665	766	782	766	766						
Ragi	-	2015-16	Kharif	1260	1526	940	1260	1260	1066						
Rape/Mustard	-	2015-16	Rabi	3000	3395	3395	3000	3000	3395						
Sesamum	-	2015-16	Kharif	4652	4652	4700	4652	4652	4652						
			Rabi	-	-	5483	-	5500	5489						
Tur Arhar	-	2015-16	Rabi	3200	3392	3600	3200	3200	3625						
Turmeric	-	2015-16	Rabi	-	8019	8200	-	8019	8019						
Wheat	-	2015-16	Rabi	1284	1284	1250	1284	1284	1284						

Table 2.5 Crop-wise District-wise Farm Harvest Prices of Principal Crops in India - Chandigarh

(Rs.per Quintal)

Crop Name	Variety	Year	Season $	Chandigarh	State Wtd Average										
Maize	-	2015-16	-	1241	1241										
Paddy	-	2015-16	-	1451	1451										
Potato	-	2015-16	-	1050	1050										
Wheat	-	2015-16	-	1580	1580										

Table 2.6 Crop-wise District-wise Farm Harvest Prices of Principal Crops in India - Chhattisgarh

(Rs.per Quintal)

Crop Name	Variety	Year	Season $	Bagipur	Balothe	Balrampur	Bastar	Bemethra	Bilaspur	Dantewada	Dhamtari	Durg	Janjgir-Champa	Jashpur	State Wtd Average
Bajra	-	2015-16	-	-	-	-	-	-	2500	-	-	-	-	-	2197
Barley	-	2015-16	-	-	-	2433	-	-	2033	-	-	-	-	-	2001
CasterSeed	-	2015-16	-	-	-	-	-	-	2220	-	-	-	-	-	2220
Chilly	Dry	2015-16	-	-	-	11258	13333	11830	12195	12000	-	12982	9900	13263	11180
Ginger	Fresh	2015-16	-	-	-	6775	6500	5069	7205	7143	-	-	5238	6363	6461
Gram	-	2015-16	-	7385	5050	5538	-	4767	4940	-	4597	4034	-	6353	5609
Ground Nut	-	2015-16	-	-	-	4810	-	5003	7003	6000	-	-	6063	4045	5786
Jowar	-	2015-16	Kharif	-	-	3908	-	-	1465	-	-	-	-	-	2299
			Rabi	-	-	1940	-	-	1767	-	-	-	-	-	1888
Linseed	-	2015-16	-	8667	-	4120	-	5000	4457	-	4044	-	-	-	5440
Maize	-	2015-16	-	-	-	970	2800	2000	2207	1000	-	-	1000	1612	1702
Nigerseed		2015-16	-	-	-	5069	-	-	6150	-	-	-	-	5000	5327
Paddy	Coarse	2015-16	-	-	1410	1425	1250	1250	1235	1000	1344	1410	1410	1148	1299
	Fine	2015-16	-	-	-	-	1441	1499	-	-	1610	1450	1450	1229	1619
	Medium	2015-16	-	1400	-	1737	1200	1343	1441	-	1450	1410	1450	1185	1392
Potato	Hills	2015-16	Summer	-	-	1600	-	1837	1715	2000	-	-	2000	1950	1794
			Winter	-	-	1600	-	2000	1514	1857	-	-	-	-	1547
Ragi	-	2015-16	-	-	-	1561	-	-	-	-	-	-	-	-	1744
Rape/Mustard	-	2015-16	-	-	-	4430	-	-	4416	-	-	-	-	3030	4976
Rice	Coarse	2015-16	-	3000	-	1954	2000	-	1893	-	-	-	-	2609	2265
	Fine	2015-16	-	8000	-	4017	4000	4657	-	4560	-	3755	-	4019	4352
	Medium	2015-16	-	4000	-	2982	2800	2610	2500	3350	-	2199	-	2677	2774
Sannhemp	-	2015-16	-	-	-	4213	-	-	-	-	-	-	-	-	4213
Sesamum	-	2015-16	Kharif	25000	-	7454	-	8600	-	-	11174	10000	-	-	10056
			Rabi	20462	-	7000	-	-	8393	-	-	-	-	7375	11337
Soyabean	-	2015-16	-	-	-	4280	-	3283	3946	-	-	-	-	-	3598
Sugar Raw	-	2015-16	-	-	-	3986	3533	3575	3862	3815	-	-	3800	3619	3903
Tobacco	-	2015-16	-	-	-	7179	-	14906	12954	-	-	15711	-	13010	11169
Tur Arhar	Delay	2015-16	-	-	11300	9322	-	7567	8127	-	-	5967	-	8350	8436
	Early	2015-16	-	-	11000	7117	-	7586	7893	-	-	6994	-	6929	7990
Turmeric	-	2015-16	-	-	-	7456	-	-	11326	-	-	-	-	9835	9611
Wheat	-	2015-16	-	2978	2471	1660	-	1507	1917	-	2397	2327	2133	1763	2102

Table 2.6 (Contd.) Crop-wise District-wise Farm Harvest Prices of Principal Crops in India - Chhattisgarh

(Rs.per Quintal)

Crop Name	Variety	Year	Season $	Kawardha	Korba	Koriya	Mahasamund	Mugeli	Narayanpur	Raigarh	Raipur	Rajnandgaon	Sukma	Surguja	State Wtd Average
Bajra	-	2015-16	-	-	-	-	-	-	3000	1414	-	-	-	-	2197
Barley	-	2015-16	-	-	-	-	-	-	-	1621	-	-	-	-	2001
CasterSeed	-	2015-16	-	-	-	-	-	-	-	-	-	-	-	-	2220
Chilly	Dry	2015-16	-	11323	-	10238	10048	8000	7678	12367	-	11958	11000	-	11180
Ginger	Fresh	2015-16	-	8017	-	7578	5025	-	7889	4928	-	-	7895	5000	6461
Gram	-	2015-16	-	5616	5917	6452	6308	-	4263	4688	9775	-	-	6333	5609
Ground Nut	-	2015-16	-	4430	-	6286	5533	-	10000	4259	-	-	-	7045	5786
Jowar	-	2015-16	Kharif	-	-	-	-	-	2368	2059	-	-	-	-	2299
			Rabi	-	-	-	-	-	-	1962	-	-	-	-	1888
Linseed	-	2015-16	-	3500	-	4230	10485	-	8000	5682	-	-	-	-	5440
Maize	-	2015-16	-	3100	1880	1318	2114	4571	1194	1330	-	-	-	1280	1702
Nigerseed		2015-16	-	-	-	-	-	-	7342	3750	-	-	-	-	5327
Paddy	Coarse	2015-16	-	1365	-	1190	-	-	-	1361	1300	1230	-	1367	1299
	Fine	2015-16	-	1473	-	2430	1700	3000	-	1450	1545	1426	-	-	1619
	Medium	2015-16	-	1298	-	1375	1496	-	-	1395	1375	1343	-	-	1392
Potato	Hills	2015-16	Summer	-	-	1783	2211	-	-	1487	-	-	-	1500	1794
			Winter	-	-	-	2000	-	1000	1181	-	-	-	-	1547
Ragi	-	2015-16	-	-	-	-	-	-	1700	-	-	2000	-	-	1744
Rape/Mustard	-	2015-16	-	3400	6269	4750	8500	4000	6159	7401	-	-	-	-	4976
Rice	Coarse	2015-16	-	2124	2225	2008	2477	2520	2120	2031	-	2468	-	2150	2265
	Fine	2015-16	-	4537	4050	4082	4260	-	3494	3680	-	4350	-	5000	4352
	Medium	2015-16	-	3080	3395	2410	2900	-	3075	2472	-	2408	-	2115	2774
Sannhemp	-	2015-16	-	-	-	-	-	-	-	-	-	-	-	-	4213
Sesamum	-	2015-16	Kharif	-	-	12595	7217	8600	6461	8709	-	13550	-	9000	10056
			Rabi	-	-	11330	15667	-	15000	9879	-	13268	-	-	11337
Soyabean	-	2015-16	-	3425	-	-	-	-	-	3175	-	-	-	-	3598
Sugar Raw	-	2015-16	-	3598	-	3959	4008	3145	4448	4214	-	-	-	4330	3903
Tobacco	-	2015-16	-	-	-	-	10442	-	-	14448	-	-	-	5667	11169
Tur Arhar	Delay	2015-16	-	-	-	7184	8500	-	-	10294	-	-	-	9000	8436
	Early	2015-16	-	7623	-	6000	7571	8250	6500	10256	-	-	-	9000	7990
Turmeric	-	2015-16	-	10000	-	7890	7869	12313	8464	12515	-	-	-	10000	9611
Wheat	-	2015-16	-	1727	-	2141	2708	-	2138	2102	1813	-	-	2400	2102

Table 2.7 Crop-wise District-wise Farm Harvest Prices of Principal Crops in India - Delhi

(Rs.per Quintal)

Crop Name	Variety	Year	Season $	Delhi	State Wtd Average										
Bajra	-	2015-16	-	1650	1650										
Barley	-	2015-16	-	1450	1450										
Gram	-	2015-16	-	5000	5000										
Jowar	-	2015-16	-	1750	1750										
Maize	-	2015-16	-	1720	1720										
Paddy	-	2015-16	-	1450	1450										
Potato	-	2015-16	-	1800	1800										
Wheat	-	2015-16	-	1525	1525										

Table 2.8 Crop-wise District-wise Farm Harvest Prices of Principal Crops in India - Goa

(Rs.per Quintal)

Crop Name	Variety	Year	Season $	Goa	State Wtd Average										
Arecanut	-	2015-16	-	23000	23000										
Cashewnut	-	2015-16	-	12200	12200										
Paddy	-	2015-16	-	1020	1020										
Ragi	-	2015-16	-	3500	3500										
Sugarcane	-	2015-16	-	250	250										

Table 2.9 Crop-wise District-wise Farm Harvest Prices of Principal Crops in India - Gujarat

(Rs.per Quintal)

Crop Name	Variety	Year	Season $	Ahmedabad	Amreli	Anand	Banaskanth	Bharuch	Bhavnagar	Dahod	Gandhinagar	Jamnagar	Junagadh	Kachchh	State Wtd Average
Bajra	-	2015-16	Kharif	1171	1852	1068	1194	1460	1010	-	1240	1174	-	1408	1261
			Summer	-	-	-	-	-	-	-	-	-	-	-	-
CasterSeed	-	2015-16	-	3897	-	3544	3972	3888	-	-	3800	3530	3623	4010	3794
Chilly	Dry	2015-16	-	-	6400	-	-	-	14952	-	-	6206	-	-	10237
Cotton	Desi	2015-16	-	3025	-	-	-	-	-	-	-	-	-	-	3088
	Hybrid	2015-16	-	3994	4741	4243	4142	4007	4564	-	3975	4173	4147	4089	4078
Gram	-	2015-16	-	3453	-	3288	-	4448	-	4041	-	2811	2977	-	3451
Ground Nut	-	2015-16	Kharif	3650	3276	-	-	3899	4421	-	3850	3471	3904	3668	3681
			Rabi	-	-	-	-	-	-	-	-	-	-	-	-
Jowar	-	2015-16	-	-	-	-	3081	2357	-	-	1575	-	1733	2143	2031
Maize	-	2015-16	-	-	-	-	1378	1375	-	1413	1350	-	-	-	1284
Paddy	-	2015-16	-	1607	-	1231	-	2020	-	1384	1540	-	-	-	1437
Potato	-	2015-16	-	-	-	-	1355	-	-	-	-	-	-	-	1075
Ragi	-	2015-16	-	-	-	-	-	-	-	-	-	-	-	-	1400
Rape/Mustard	-	2015-16	-	-	-	-	3292	-	-	-	-	-	-	2881	3622
Sesamum	-	2015-16	-	-	11533	6063	9182	-	6148	-	8365	11170	-	9502	9202
Sugarcane	-	2015-16	-	-	-	-	714	-	-	-	-	-	-	275	-
Tobacco	-	2015-16	-	-	-	5571	-	-	-	-	-	-	-	-	4611
Tur Arhar	-	2015-16	-	-	-	-	-	5433	-	-	4835	-	-	-	4841
Wheat	-	2015-16	-	1560	1812	1294	1527	2219	1631	1417	1520	1626	1420	1680	1608

Table 2.9 (Contd.) Crop-wise District-wise Farm Harvest Prices of Principal Crops in India - Gujarat

(Rs.per Quintal)

Crop Name	Variety	Year	Season $	Kheda	Mahesana	Narmada	Navsari	Panch Mahals	Patan	Porbandar	Rajkot	Sabar Kantha	Surat	Surendranagar	State Wtd Average
Bajra	-	2015-16	Kharif	1271	1176	1300	-	1198	1196	1280	-	1307	-	1125	1261
			Summer	-	-	-	-	-	-	-	-	-	-	-	-
CasterSeed	-	2015-16	-	3851	3975	3800	-	3558	4324	4255	-	3630	-	3689	3794
Chilly	Dry	2015-16	-	-	-	-	-	-	-	-	11079	-	-	-	10237
Cotton	Desi	2015-16	-	-	-	-	-	-	-	-	-	-	-	3150	3088
	Hybrid	2015-16	-	3719	3435	3580	-	3939	4135	4100	3964	4683	-	4000	4078
Gram	-	2015-16	-	2834	-	-	-	3390	3275	3767	4077	-	-	3625	3451
Ground Nut	-	2015-16	Kharif	-	3599	-	-	2910	-	3900	3786	3608	3550	4500	3681
			Rabi	-	-	-	-	-	-	-	-	-	3532	-	-
Jowar	-	2015-16	-	-	3146	1300	-	1525	-	1825	-	1785	-	-	2031
Maize	-	2015-16	-	1210	-	1400	-	1398	-	750	-	1342	-	-	1284
Paddy	-	2015-16	-	1331	1548	1800	1377	1266	-	-	-	1355	1389	-	1437
Potato	-	2015-16	-	1083	860	-	-	-	-	-	-	-	-	-	1075
Ragi	-	2015-16	-	-	-	-	-	-	-	-	-	-	-	-	1400
Rape/Mustard	-	2015-16	-	3111	3014	-	-	-	3199	7500	-	2874	-	-	3622
Sesamum	-	2015-16	-	-	-	-	-	7500	8589	11467	11445	-	-	6500	9202
Sugarcane	-	2015-16	-	-	-	-	-	-	-	-	-	-	-	-	-
Tobacco	-	2015-16	-	4062	-	-	-	-	-	-	-	-	-	-	4611
Tur Arhar	-	2015-16	-	-	-	3400	-	48755	-	-	-	3599	4462	-	4841
Wheat	-	2015-16	-	1521	1593	1700	-	1486	1688	1550	1773	1723	1785	1425	1608

Table 2.9 (Contd.) Crop-wise District-wise Farm Harvest Prices of Principal Crops in India - Gujarat

(Rs.per Quintal)

Crop Name	Variety	Year	Season $	Tapi	The Dangs	Vadodara	Valsad	State Wtd Average							
Bajra	-	2015-16	Kharif	-	-	1330	-	1261							
			Summer	-	-	1298	-	-							
CasterSeed	-	2015-16	-	-	-	3731	-	3794							
Chilly	Dry	2015-16	-	-	-	-	-	10237							
Cotton	Desi	2015-16	-	-	-	-	-	3088							
	Hybrid	2015-16	-	-	-	3898	-	4078							
Gram	-	2015-16	-	2970	-	-	-	3451							
Ground Nut	-	2015-16	Kharif	3758	3620	-	-	3681							
			Rabi	3758	-	-	-	-							
Jowar	-	2015-16	-	1602	1350	1766	2195	2031							
Maize	-	2015-16	-	1097	-	-	-	1284							
Paddy	-	2015-16	-	1270	1252	1450	1421	1437							
Potato	-	2015-16	-	-	-	-	-	1075							
Ragi	-	2015-16	-	-	1600	-	1200	1400							
Rape/Mustard	-	2015-16	-	3802	-	-	-	3622							
Sesamum	-	2015-16	-	10127	-	-	-	9202							
Sugarcane	-	2015-16	-	-	-	-	215	-							
Tobacco	-	2015-16	-	-	-	4200	-	4611							
Tur Arhar	-	2015-16	-	4868	3892	5164	8300	4841							
Wheat	-	2015-16	-	1475	-	1621	-	1608							

Table 2.10 Crop-wise District-wise Farm Harvest Prices of Principal Crops in India - Haryana

(Rs.per Quintal)

Crop Name	Variety	Year	Season $	Ambala	Bhiwani	Faridabad	Fatehabad	Gurgaon	Hisar	Jhajjar	Jind	Kaithal	Karnal	Kurukshetra	State Wtd Average
Bajra	-	2015-16	-	1350	1400	1300	1350	1300	1300	1300	1300	1300	1250	-	1329
			Kharif	-	1350	1400	1300	1350	1300	1300	1300	1300	1250	-	1329
Chilly	-	2015-16	Kharif	4000	-	-	3500	-	7100	-	5100	4200	4300	5000	5984
Cotton	American	2015-16	Kharif	-	5000	-	4600	4100	5150	4500	4600	5300	-	-	5021
	Desi	2015-16	Kharif	-	-	-	5000	-	5400	-	-	-	-	-	5371
Ground Nut	-	2015-16	Kharif	-	4700	-	4200	4200	3620	6000	-	-	4200	-	3851
Jowar	-	2015-16	Kharif	-	3000	4000	-	4000	3000	-	-	-	-	-	3107
Maize	-	2015-16	Kharif	1600	-	-	-	-	-	-	-	-	-	-	1600
Paddy	-	2015-16	Kharif	2800	3100	1850	2500	1500	1600	1450	2800	2600	1700	3000	1753

Table 2.10 (Contd.) Crop-wise District-wise Farm Harvest Prices of Principal Crops in India - Haryana

(Rs.per Quintal)

Crop Name	Variety	Year	Season $	Mahendragarh	Mewat	Palwal	Panchkula	Panipat	Rewari	Rohtak	Sirsa	Sonipat	Yamunanagar	State Wtd Average	
Bajra	-	2015-16	-	1350	1300	1300	1300	-	1300	1350	1350	1600	-	1329	
			Kharif	1350	1300	1300	1300	-	1300	1350	1350	1600	-	1329	
Chilly	-	2015-16	Kharif	8000	4600	-	4800	8300	-	-	8000	7000	5100	5984	
Cotton	American	2015-16	Kharif	5200	4200	4100	-	-	5300	5200	5200	4700	-	5021	
	Desi	2015-16	Kharif	-	-	-	-	-	-	-	5500	-	-	5371	
Ground Nut	-	2015-16	Kharif	4200	3600	-	4100	-	3700	4200	3600	-	3650	3851	
Jowar	-	2015-16	Kharif	-	3500	3000	-	-	-	3000	-	3000	-	3107	
Maize	-	2015-16	Kharif	-	-	-	1600	-	-	1600	-	-	-	1600	
Paddy	-	2015-16	Kharif	-	4500	2900	4200	3000	3500	3000	3000	2650	3500	1753	

Table 2.11 Crop-wise District-wise Farm Harvest Prices of Principal Crops in India - Himachal Pradesh

(Rs.per Quintal)

Crop Name	Variety	Year	Season $	Bilaspur	Chamba	Hamirpur	Kangra	Kinnaur	Kullu	Lahaul & Spiti	Mandi	Shimla	Sirmaur	Solan	State Wtd Average
Barley	-	2015-16	-	2000	1545	2050	1550	3500	2100	2200	2133	1750	1373	1573	1967
Chilly	-	2015-16	-	13000	20000	15000	-	-	16000	-	11400	9000	7723	7856	12775
Ginger	-	2015-16	-	10000	-	7000	-	-	-	-	6000	6350	3565	5860	6396
Gram	-	2015-16	-	11000	7500	6324	8000	-	8600	-	-	-	4283	5600	7226
Ground Nut	-	2015-16	-	-	-	-	-	-	-	-	-	-	7690	8180	7665
Linseed	-	2015-16	-	-	8000	8500	7200	-	-	-	6120	-	-	4620	6907
Maize	-	2015-16	-	1400	1363	1963	1600	3000	1800	1500	1553	1820	1553	1500	1713
Paddy	-	2015-16	-	1800	2240	1817	2000	-	2800	-	2127	2453	1550	2530	2084
Potato	-	2015-16	Kharif	-	2110	-	1500	3000	2800	3466	2022	2020	2400	1820	2262
			Rabi	2000	1566	1500	-	-	2100	-	1683	2020	1593	1670	1702
Rape/Mustard	-	2015-16	-	2500	4828	8800	8000	-	8100	3000	6764	5200	3653	5250	5522
Sesamum	-	2015-16	-	16000	-	11240	12000	-	-	-	6550	-	7720	10583	10809
Soyabean	-	2015-16	-	5400	-	-	5000	-	4600	-	5968	-	5700	-	5334
Turmeric	-	2015-16	-	8000	-	14000	-	-	-	-	5543	-	9100	-	10404
Wheat	-	2015-16	-	1500	1490	2000	1500	4000	1800	2000	1661	2000	1515	1650	1885

Table 2.11 (Contd.) Crop-wise District-wise Farm Harvest Prices of Principal Crops in India - Himachal Pradesh

(Rs.per Quintal)

Crop Name	Variety	Year	Season $	Una	State Wtd Average										
Barley	-	2015-16	-	1833	1967										
Chilly	-	2015-16	-	15000	12775										
Ginger	-	2015-16	-	6000	6396										
Gram	-	2015-16	-	6500	7226										
Ground Nut	-	2015-16	-	7166	7665										
Linseed	-	2015-16	-	7000	6907										
Maize	-	2015-16	-	1503	1713										
Paddy	-	2015-16	-	1527	2084										
Potato	-	2015-16	Kharif	1483	2262										
			Rabi	1187	1702										
Rape/Mustard	-	2015-16	-	4650	5522										
Sesamum	-	2015-16	-	11570	10809										
Soyabean	-	2015-16	-	-	5334										
Turmeric	-	2015-16	-	15375	10404										
Wheat	-	2015-16	-	1500	1885										

Table 2.12 Crop-wise District-wise Farm Harvest Prices of Principal Crops in India - Jammu & Kashmir

(Rs.per Quintal)

Crop Name	Variety	Year	Season $	Anantnag	Bandipora	Baramulla	Budgam	Doda	Ganderbal	Jammu	Kargil	Kathua	Kishtwar	Kulgam	State Wtd Average
Bajra	-	2015-16	-	-	-	-	-	-	-	1100	-	1200	-	-	1157
Barley	-	2015-16	-	-	-	-	-	1550	-	1500	-	1600	1500	-	1555
Chilly	-	2015-16	-	8500	-	8000	8000	-	-	-	-	9000	-	8000	8528
Linseed	-	2015-16	-	-	-	-	-	-	-	6000	-	6000	-	-	5555
Maize	-	2015-16	-	1200	1300	1300	1300	1300	1300	1100	-	1300	1250	1250	1264
Paddy	-	2015-16	-	1400	1500	1500	1500	1800	1500	2400	-	1600	1700	1400	1713
Potato	-	2015-16	-	1500	1500	1400	1400	-	1400	1200	1500	1400	-	1400	1458
Rape/Mustard	-	2015-16	-	3800	3300	3300	2800	5000	2500	5200	-	7500	5800	3800	3757
Sesamum	-	2015-16	-	-	-	-	-	-	-	-	-	6300	-	-	6252
Sugarcane	-	2015-16	-	-	-	-	-	-	-	-	-	-	-	-	1450
Wheat	-	2015-16	-	-	-	-	2000	1500	-	1500	1500	1500	1400	-	1505

Table 2.12 (Contd.) Crop-wise District-wise Farm Harvest Prices of Principal Crops in India - Jammu & Kashmir

(Rs.per Quintal)

Crop Name	Variety	Year	Season $	Kupwara	Leh	Poonch	Pulwama	Rajauri	Rambon	Reasi	Samba	Shopian	Srinagar	Udhampur	State Wtd Average
Bajra	-	2015-16	-	-	-	-	-	1400	-	1400	1100	-	-	1100	1157
Barley	-	2015-16	-	-	1450	-	-	1450	1500	1500	1800	-	-	1500	1555
Chilly	-	2015-16	-	-	-	-	8500	9000	-	-	-	-	8000	8000	8528
Linseed	-	2015-16	-	-	4000	-	4600	5800	-	-	-	-	-	-	5555
Maize	-	2015-16	-	1500	-	1250	1300	1300	1250	1300	1150	1300	1300	1250	1264
Paddy	-	2015-16	-	1600	-	1700	1500	1700	1700	1800	1700	1500	1500	1700	1713
Potato	-	2015-16	-	1500	1700	1450	1400	-	-	-	-	1400	1500	1400	1458
Rape/Mustard	-	2015-16	-	-	3500	5800	3500	5800	4800	3000	5000	3300	2500	5000	3757
Sesamum	-	2015-16	-	-	6200	-	-	6300	-	6300	6200	-	-	6500	6252
Sugarcane	-	2015-16	-	-	-	-	-	-	-	-	1450	-	-	-	1450
Wheat	-	2015-16	-	-	1500	1500	2000	1500	1500	1450	1500	-	-	1550	1505

Table 2.13 Crop-wise District-wise Farm Harvest Prices of Principal Crops in India - Jharkhand

(Rs.per Quintal)

Crop Name	Variety	Year	Season $	Chatra	Deoghar	Dhanbad	Dumka	East Singhbhum	Garhwa	Giridih	Godda	Gumla	Hazaribagh	Jamtara	State Wtd Average
Barley	-	2015-16	Rabi	-	1300	-	-	-	1333	-	1522	-	-	-	1486
Ginger	-	2015-16	Rabi	-	-	-	-	-	-	12554	-	-	-	-	8759
Gram	-	2015-16	Rabi	5095	5200	-	7945	4455	4061	4554	4161	3433	4539	6000	5010
Maize	-	2015-16	Rabi	-	-	-	-	-	-	-	-	-	-	-	1257
Potato	-	2015-16	Rabi	1237	1000	1340	-	1033	-	1152	989	1258	-	944	1211
Rape/Mustard	-	2015-16	Rabi	4585	3500	-	2863	3800	-	3838	2917	7378	6247	4800	4326
Tur Arhar	-	2015-16	Rabi	5191	10000	-	9958	5200	-	3379	4278	3640	5917	-	6816
Turmeric	-	2015-16	Rabi	-	1000	-	-	-	-	-	-	-	-	-	1000
Wheat	-	2015-16	Rabi	1586	1400	1627	1171	1944	1507	1429	1844	1860	1653	1922	1631

Table 2.13 (Contd.) Crop-wise District-wise Farm Harvest Prices of Principal Crops in India - Jharkhand

(Rs.per Quintal)

Crop Name	Variety	Year	Season $	Khunti	Koderma	Latehar	Lohardaga	Pakur	Palamu	Ramgarh	Ranchi	Sahibganj	Seraikela	Simdega	State Wtd Average
Barley	-	2015-16	Rabi	-	-	-	-	-	-	-	-	1830	-	-	1486
Ginger	-	2015-16	Rabi	-	-	-	8000	-	-	3350	-	1130	-	-	8759
Gram	-	2015-16	Rabi	6990	5907	4240	6000	4644	5913	6000	2990	6010	4200	4544	5010
Maize	-	2015-16	Rabi	-	-	-	-	-	1265	1332	-	1210	-	-	1257
Potato	-	2015-16	Rabi	1330	-	-	1400	-	-	1175	1131	1275	1583	1311	1211
Rape/Mustard	-	2015-16	Rabi	-	4435	-	4000	6454	-	2900	2695	3820	6100	4500	4326
Tur Arhar	-	2015-16	Rabi	6920	10010	5192	4500	6693	8606	8000	7151	7390	10267	5889	6816
Turmeric	-	2015-16	Rabi	-	-	-	-	-	-	-	-	-	-	-	1000
Wheat	-	2015-16	Rabi	1658	1855	1534	1600	1939	1635	1640	1674	1615	1717	1544	1631

Table 2.13 (Contd.) Crop-wise District-wise Farm Harvest Prices of Principal Crops in India - Jharkhand

(Rs.per Quintal)

Crop Name	Variety	Year	Season $	West Singhbhum	State Wtd Average
Barley	-	2015-16	Rabi	-	1486
Ginger	-	2015-16	Rabi	-	8759
Gram	-	2015-16	Rabi	3202	5010
Maize	-	2015-16	Rabi	-	1257
Potato	-	2015-16	Rabi	-	1211
Rape/Mustard	-	2015-16	Rabi	3040	4326
Tur Arhar	-	2015-16	Rabi	8129	6816
Turmeric	-	2015-16	Rabi	-	1000
Wheat	-	2015-16	Rabi	1367	1631

Table 2.14 Crop-wise District-wise Farm Harvest Prices of Principal Crops in India - Karnataka

(Rs.per Quintal)

Crop Name	Variety	Year	Season $	Bagalkot	Bangalore Rural	Bangalore Urban	Belgaum	Bellary	Bidar	Bijapur	Chamrajnagar	Chickaballapura	Chickmagalur	Chitradurga	State Wtd Average
Arecanut	-	2015-16	Kharif	-	-	-	-	-	-	-	-	-	-	-	23899
			Summer	-	-	-	-	-	-	-	-	-	-	27989	26281
Bajra	-	2015-16	Kharif	-	-	-	-	1473	1425	1006	1438	1394	-	-	1339
			Summer	-	-	-	-	-	-	-	-	-	-	-	1678
Cashewnut	-	2015-16	Summer	-	-	-	-	-	-	-	-	8619	-	-	9075
CasterSeed	-	2015-16	Kharif	-	-	-	-	-	-	-	-	-	-	-	3285
Chilly	Dry	2015-16	Kharif	-	-	-	-	11759	-	-	-	-	-	-	7653
Coconut	-	2015-16	Kharif	-	-	-	-	-	-	-	15894	-	-	-	10998
			Rabi	-	-	-	-	-	-	-	-	-	-	-	7005
			Summer	-	-	-	-	-	-	-	15894	-	-	9771	10049
Cotton	-	2015-16	Kharif	-	-	-	4350	4266	-	-	3996	-	-	4778	4415
			Rabi	-	-	-	-	-	-	-	-	-	-	-	4216
			Summer	-	-	-	-	4266	-	-	-	-	-	-	4266
Ginger	-	2015-16	Kharif	-	-	-	-	-	-	-	-	-	-	-	1048
Gram	-	2015-16	Kharif	-	-	-	-	-	-	3500	2209	2289	-	-	2756
			Rabi	-	-	-	-	-	-	-	-	-	-	-	4530
Ground Nut	-	2015-16	Kharif	-	-	-	4354	4505	-	4339	3892	3734	3817	4108	4212
			Rabi	-	-	-	4521	-	-	-	-	-	-	-	4121
			Summer	5063	-	-	3593	4936	-	5379	-	-	-	4820	4494
Jowar	-	2015-16	Rabi	1367	-	-	-	-	-	-	-	-	-	-	1367
			Summer	-	-	-	-	1483	-	-	-	-	-	-	1394
	HYV	2015-16	Kharif	-	-	-	2292	1371	1296	-	2138	-	-	-	1662
			Rabi	-	-	-	-	-	-	-	-	-	-	-	1257
	Local	2015-16	Kharif	-	-	-	-	-	1296	-	2075	-	1900	-	1918
Linseed	-	2015-16	Rabi	-	-	-	-	-	-	8500	-	-	-	-	8500
Maize	-	2015-16	Kharif	1435	1530	-	1402	1410	-	1479	1420	1399	1396	1448	1378
	HB	2015-16	Rabi	-	1490	-	-	1345	1474	1383	-	-	-	1439	1391
			Summer	-	1500	-	1546	1514	-	-	-	-	-	-	1460
Paddy	-	2015-16	Rabi	-	-	-	-	-	-	-	-	-	-	-	1530
			Summer	-	-	-	-	1476	-	-	-	-	1705	-	1743
	Local	2015-16	Kharif	-	-	-	-	-	-	-	-	-	1310	-	1592
Potato	-	2015-16	Kharif	-	-	-	-	-	-	-	-	-	1075	-	952
			Rabi	-	-	-	-	-	-	-	-	-	-	-	1714
			Summer	-	-	-	-	-	-	-	-	-	-	-	1151
Ragi	HYV	2015-16	Kharif	-	1441	2013	-	1375	-	-	1749	1620	1397	1411	1606
			Rabi	-	-	-	-	-	-	-	-	-	-	-	1607

Table 2.14 (Contd.) Crop-wise District-wise Farm Harvest Prices of Principal Crops in India - Karnataka

(Rs.per Quintal)

Crop Name	Variety	Year	Season $	D. Kannada	Davangere	Dharwad	Gadag	Gulbarga	Hassan	Haveri	Kodagu	Kolar	Koppal	Mandya	State Wtd Average
Arecanut	-	2015-16	Kharif	-	-	-	-	-	-	-	-	-	-	-	23899
			Summer	29725	27095	-	-	-	-	-	25750	-	-	-	26281
Bajra	-	2015-16	Kharif	-	-	-	1324	-	-	-	-	-	-	1392	1339
			Summer	-	-	-	-	-	-	-	-	-	1678	-	1678
Cashewnut	-	2015-16	Summer	5745	-	-	-	-	-	-	11450	-	-	-	9075
CasterSeed	-	2015-16	Kharif	-	-	-	-	-	-	-	-	-	-	-	3285
Chilly	Dry	2015-16	Kharif	-	-	6000	6063	8170	-	8823	-	-	5800	-	7653
Coconut	-	2015-16	Kharif	-	-	-	-	-	-	-	-	-	-	-	10998
			Rabi	-	-	-	-	-	-	-	-	-	-	-	7005
			Summer	15125	7968	-	-	-	8244	-	-	-	-	-	10049
Cotton	-	2015-16	Kharif	-	-	4856	4178	-	-	4773	-	-	-	-	4415
			Rabi	-	-	4594	4196	-	-	4294	-	-	3780	-	4216
			Summer	-	-	-	-	-	-	-	-	-	-	-	4266
Ginger	-	2015-16	Kharif	-	-	-	-	-	1048	-	-	-	-	-	1048
Gram	-	2015-16	Kharif	-	-	-	4368	-	-	-	-	-	1923	3270	2756
			Rabi	-	-	-	-	-	-	-	-	-	4530	-	4530
Ground Nut	-	2015-16	Kharif	-	4815	4004	4565	4490	-	4092	-	3716	-	4537	4212
			Rabi	-	-	-	4502	-	-	-	-	-	-	-	4121
			Summer	-	-	-	4719	-	-	3892	-	-	-	-	4494
Jowar	-	2015-16	Rabi	-	-	-	-	-	-	-	-	-	-	-	1367
			Summer	-	-	-	-	-	-	-	-	-	1305	-	1394
	HYV	2015-16	Kharif	-	1384	1520	-	2601	-	1439	-	-	1193	-	1662
			Rabi	-	-	-	-	-	-	-	-	-	1257	-	1257
	Local	2015-16	Kharif	-	-	-	-	2399	-	-	-	-	-	-	1918
Linseed	-	2015-16	Rabi	-	-	-	-	-	-	-	-	-	-	-	8500
Maize	-	2015-16	Kharif	-	1342	1340	1287	1365	1336	1405	1188	-	1320	1339	1378
	HB	2015-16	Rabi	-	1383	1396	1337	1425	1520	1348	-	-	1339	-	1391
			Summer	-	-	-	1335	-	-	1423	-	-	1400	-	1460
Paddy	-	2015-16	Rabi	1819	-	-	-	-	-	-	-	-	-	-	1530
			Summer	1794	-	-	-	-	1411	1488	-	1779	2560	1716	1743
	Local	2015-16	Kharif	1606	-	-	-	-	1623	-	1328	-	-	-	1592
Potato	-	2015-16	Kharif	-	-	-	-	906	874	-	-	-	-	-	952
			Rabi	-	-	-	-	-	-	-	-	1714	-	-	1714
			Summer	-	-	-	-	-	-	-	-	1151	-	-	1151
Ragi	HYV	2015-16	Kharif	-	1603	-	-	-	1451	-	-	1731	-	1693	1606
			Rabi	-	-	-	-	-	1478	-	-	-	-	1676	1607

Table 2.14 (Contd.) Crop-wise District-wise Farm Harvest Prices of Principal Crops in India - Karnataka

(Rs.per Quintal)

Crop Name	Variety	Year	Season $	Mysore	Raichur	Ramanagara	Shimoga	Tumkur	U.Kannada	Udupi	State Wtd Average				
Arecanut	-	2015-16	Kharif	-	-	-	-	24568	23229	-	23899				
			Summer	-	-	-	23388	22202	-	27817	26281				
Bajra	-	2015-16	Kharif	-	1274	-	-	-	-	-	1339				
			Summer	-	-	-	-	-	-	-	1678				
Cashewnut	-	2015-16	Summer	-	-	-	-	-	-	10484	9075				
CasterSeed	-	2015-16	Kharif	3220	-	3349	-	-	-	-	3285				
Chilly	Dry	2015-16	Kharif	-	-	-	-	6953	-	-	7653				
Coconut	-	2015-16	Kharif	-	-	7034	-	10470	10593	-	10998				
			Rabi	-	-	7005	-	-	-	-	7005				
			Summer	13453	-	4035	-	7469	-	8485	10049				
Cotton	-	2015-16	Kharif	4654	-	3884	-	-	-	-	4415				
			Rabi	-	-	-	-	-	-	-	4216				
			Summer	-	-	-	-	-	-	-	4266				
Ginger	-	2015-16	Kharif	-	-	-	-	-	-	-	1048				
Gram	-	2015-16	Kharif	2242	-	2248	-	-	-	-	2756				
			Rabi	-	-	-	-	-	-	-	4530				
Ground Nut	-	2015-16	Kharif	-	-	4009	-	3934	-	-	4212				
			Rabi	-	-	-	-	4925	-	-	4121				
			Summer	-	4925	-	-	3031	-	-	4494				
Jowar	-	2015-16	Rabi	-	-	-	-	-	-	-	1367				
			Summer	-	-	-	-	-	-	-	1394				
	HYV	2015-16	Kharif	1389	-	-	-	-	-	-	1662				
			Rabi	-	-	-	-	-	-	-	1257				
	Local	2015-16	Kharif	-	-	-	-	-	-	-	1918				
Linseed	-	2015-16	Rabi	-	-	-	-	-	-	-	8500				
Maize	-	2015-16	Kharif	1242	-	-	1381	1423	1433	-	1378				
	HB	2015-16	Rabi	1111	-	-	-	1484	-	-	1391				
			Summer	-	-	-	-	1504	-	-	1460				
Paddy	-	2015-16	Rabi	-	-	1450	-	1490	-	1604	1530				
			Summer	1675	2445	1517	-	1397	-	-	1743				
	Local	2015-16	Kharif	-	2076	-	-	1610	-	-	1592				
Potato	-	2015-16	Kharif	-	-	-	-	-	-	-	952				
			Rabi	-	-	-	-	-	-	-	1714				
			Summer	-	-	-	-	-	-	-	1151				
Ragi	HYV	2015-16	Kharif	-	-	1763	-	1629	-	-	1606				
			Rabi	1392	-	1881	-	-	-	-	1607				

Table 2.14 (Contd.) Crop-wise District-wise Farm Harvest Prices of Principal Crops in India - Karnataka

(Rs.per Quintal)

Crop Name	Variety	Year	Season $	Bagalkot	Bangalore Rural	Bangalore Urban	Belgaum	Bellary	Bidar	Bijapur	Chamrajnagar	Chickaballapura	Chickmagalur	Chitradurga	State Wtd Average
Ragi	HYV	2015-16	Summer	-	-	-	-	-	-	-	-	-	-	1433	1639
Sesamum	-	2015-16	Kharif	-	-	-	-	5133	6711	-	-	-	5308	5595	5760
			Rabi	-	-	-	-	-	-	-	-	-	-	-	4550
			Summer	-	-	-	-	-	-	-	-	-	-	-	2300
Sugarcane	-	2015-16	Kharif	-	-	-	-	-	-	-	-	-	-	-	2600
			Summer	-	-	-	-	-	-	-	-	-	-	-	1728
Sunflower	-	2015-16	Kharif	3234	-	-	3213	3356	3381	3809	3671	-	2905	3596	3401
			Rabi	3811	-	-	3013	3142	-	3694	-	-	-	3416	3302
			Summer	-	-	-	-	-	-	-	-	-	-	-	3501
Tobacco	-	2015-16	Summer	-	-	-	-	-	-	-	-	-	-	-	10172
Tur Arhar	-	2015-16	Kharif	-	-	-	-	6048	5636	8483	-	4420	-	7392	6134
Turmeric	-	2015-16	Kharif	-	-	-	-	-	-	-	8056	-	-	-	8056
			Summer	-	-	-	-	-	-	-	8056	-	-	-	8056
Wheat	-	2015-16	Rabi	2144	-	-	2041	-	-	2706	-	-	-	1465	1930
			Summer	-	-	-	-	-	-	-	-	-	-	-	1493

Table 2.14 (Contd.) Crop-wise District-wise Farm Harvest Prices of Principal Crops in India - Karnataka

(Rs.per Quintal)

Crop Name	Variety	Year	Season $	D. Kannada	Davangere	Dharwad	Gadag	Gulbarga	Hassan	Haveri	Kodagu	Kolar	Koppal	Mandya	State Wtd Average
Ragi	HYV	2015-16	Summer	-	-	-	-	-	-	-	-	-	-	-	1639
Sesamum	-	2015-16	Kharif	-	-	-	5072	5413	7347	-	-	-	4785	-	5760
			Rabi	-	-	-	-	-	-	-	-	-	-	-	4550
			Summer	-	-	-	-	-	-	-	-	-	-	-	2300
Sugarcane	-	2015-16	Kharif	-	-	-	-	-	-	-	-	-	-	-	2600
			Summer	-	1586	-	-	-	1869	-	-	-	-	-	1728
Sunflower	-	2015-16	Kharif	-	2975	-	3576	3266	3283	3376	-	-	3406	-	3401
			Rabi	-	-	3075	3518	3338	-	3441	-	-	3421	-	3302
			Summer	-	-	-	3501	-	-	-	-	-	-	-	3501
Tobacco	-	2015-16	Summer	-	-	-	-	-	-	-	-	-	-	-	10172
Tur Arhar	-	2015-16	Kharif	-	4009	-	7510	9380	-	6503	-	-	-	-	6134
Turmeric	-	2015-16	Kharif	-	-	-	-	-	-	-	-	-	-	-	8056
			Summer	-	-	-	-	-	-	-	-	-	-	-	8056
Wheat	-	2015-16	Rabi	-	-	1647	1775	2458	-	1637	-	-	1609	-	1930
			Summer	-	-	-	-	-	-	-	-	-	1493	-	1493

Table 2.14 (Contd.) Crop-wise District-wise Farm Harvest Prices of Principal Crops in India - Karnataka

(Rs.per Quintal)

Crop Name	Variety	Year	Season $	Mysore	Raichur	Ramanagara	Shimoga	Tumkur	U.Kannada	Udupi	State Wtd Average				
Ragi	HYV	2015-16	Summer	-	-	1893	-	1591	-	-	1639				
Sesamum	-	2015-16	Kharif	6974	-	5260	-	-	-	-	5760				
			Rabi	-	-	4550	-	-	-	-	4550				
			Summer	-	-	2300	-	-	-	-	2300				
Sugarcane	-	2015-16	Kharif	-	-	-	-	-	2600	-	2600				
			Summer	-	-	-	-	-	-	-	1728				
Sunflower	-	2015-16	Kharif	-	3532	-	-	3373	-	-	3401				
			Rabi	-	2501	-	-	-	-	-	3302				
			Summer	-	-	-	-	-	-	-	3501				
Tobacco	-	2015-16	Summer	10172	-	-	-	-	-	-	10172				
Tur Arhar	-	2015-16	Kharif	3228	-	4248	-	4295	8585	-	6134				
Turmeric	-	2015-16	Kharif	-	-	-	-	-	-	-	8056				
			Summer	-	-	-	-	-	-	-	8056				
Wheat	-	2015-16	Rabi	-	1815	-	-	-	-	-	1930				
			Summer	-	-	-	-	-	-	-	1493				

Table 2.15 Crop-wise District-wise Farm Harvest Prices of Principal Crops in India - Kerala

(Rs.per Quintal)

Crop Name	Variety	Year	Season $	Alappuzha	Ernakulam	Idukki	Kannur	Kasargod	Kollam	Kottayam	Kozhikode	Malappuram	Palakkad	Pathanamthitta	State Wtd Average
Banana	-	2015-16	-	3867	3108	2788	3110	3067	4016	3453	3168	2851	2773	3792	2939
Ginger	Dry	2015-16	-	-	-	-	-	14210	-	-	13393	-	15295	-	14452
Paddy	-	2015-16	-	1993	1719	1894	1694	1687	2027	2101	2083	1441	1839	2142	1835
Pepper	-	2015-16	-	-	61634	64149	62740	62228	61718	63904	62347	62951	62951	64466	-
Tapioca	-	2015-16	-	1119	941	1138	1187	1317	1200	1156	1083	913	1065	1248	1172

Table 2.15 (Contd.) Crop-wise District-wise Farm Harvest Prices of Principal Crops in India - Kerala

(Rs.per Quintal)

Crop Name	Variety	Year	Season $	Thiruvanant hapuram	Trichur	Wayanad	State Wtd Average								
Banana	-	2015-16	-	3607	3205	2374	2939								
Ginger	Dry	2015-16	-	-	-	14000	14452								
Paddy	-	2015-16	-	2036	1937	1369	1835								
Pepper	-	2015-16	-	62344	61385	64685	-								
Tapioca	-	2015-16	-	1408	1078	910	1172								

Table 2.16 Crop-wise District-wise Farm Harvest Prices of Principal Crops in India - Madhya Pradesh

(Rs.per Quintal)

Crop Name	Variety	Year	Season $	Alirajpur	Anuppur	Ashoknagar	Balaghat	Barwani	Betul	Bhind	Bhopal	Burhanpur	Chhindwara	Damoh	Datia
Bajra	-	2015-16	-	1044	-	-	-	1645	-	1305	-	-	-	-	1313
Barley	-	2015-16	-	-	-	-	-	-	-	1588	-	-	-	-	1410
CasterSeed	-	2015-16	-	-	-	-	-	-	-	-	-	-	-	-	-
Chilly	Dry	2015-16	-	-	-	-	-	-	-	10341	11428	15580	8489	-	-
Cotton	-	2015-16	-	4106	-	-	-	-	-	-	-	4089	4000	-	-
Ginger	Fresh	2015-16	-	-	-	7233	8900	7963	-	3525	-	7478	5699	5079	-
Gram	-	2015-16	-	4159	4057	-	-	-	-	5483	4813	5139	4606	4481	4910
Ground Nut	-	2015-16	-	3568	-	3693	-	5693	4947	-	6888	5000	4000	3404	-
Jowar	-	2015-16	Kharif	-	-	-	-	1430	-	1337	2200	1364	1520	1877	1985
			Rabi	-	-	-	-	-	-	-	-	-	-	-	-
Linseed	-	2015-16	-	-	4041	-	-	-	-	5700	4845	5620	-	-	-
Maize	-	2015-16	-	1220	1423	1719	1750	-	-	-	1615	-	1242	1331	1636
Nigerseed		2015-16	-	-	4500	-	-	-	-	-	-	-	-	-	-
Paddy	Coarse	2015-16	-	1260	-	-	-	-	-	-	1507	-	-	1668	1308
	Fine	2015-16	-	-	-	-	-	-	-	-	2100	-	-	-	1497
	Medium	2015-16	-	1440	-	-	-	-	-	-	1612	-	-	-	-
Potato	Hills	2015-16	Summer	-	-	-	-	-	-	-	-	-	-	-	-
			Winter	-	1153	-	-	-	-	-	-	-	-	-	-
Rape/Mustard	-	2015-16	-	-	-	-	-	-	-	3793	-	-	-	-	3696
Rice	Coarse	2015-16	-	-	2200	3513	-	3060	-	-	2165	2225	2538	1738	-
	Fine	2015-16	-	-	3400	4953	-	3500	-	-	3994	3240	4599	3773	-
	Medium	2015-16	-	-	2652	4083	-	-	-	-	2988	2936	3800	3094	-
Sannhemp	-	2015-16	-	-	-	-	-	3400	-	-	-	-	-	-	-
Sesamum	-	2015-16	Kharif	6125	-	12033	-	-	-	5412	-	9800	12240	8996	5653
			Rabi	-	-	-	-	-	-	-	-	-	-	-	-
Soyabean	-	2015-16	-	-	-	-	-	3355	-	-	3413	-	3254	-	-
Sugar Raw	-	2015-16	-	-	-	3263	3300	3935	-	-	3624	-	3469	-	-
Tobacco	-	2015-16	-	-	-	-	-	-	-	12500	-	-	-	-	-
Tur Arhar	Delay	2015-16	-	8000	7409	-	-	-	-	-	6800	8364	-	-	9884
	Early	2015-16	-	7500	-	-	-	-	-	-	7223	7210	-	-	9800
Turmeric	-	2015-16	-	-	-	-	-	-	-	-	-	-	-	-	-
Wheat	-	2015-16	-	1586	1798	-	-	-	-	1550	1991	1780	1754	1522	1507

Table 2.16 (Contd.) Crop-wise District-wise Farm Harvest Prices of Principal Crops in India - Madhya Pradesh

(Rs.per Quintal)

Crop Name	Variety	Year	Season $	Dewas	Dhar	Dindori	Gwalior	Harda	Hoshangabad	Indore	Jabalpur	Jhabua	Katni	Khargone	Mandla
Bajra	-	2015-16	-	-	1582	-	1489	-	-	-	-	-	-	-	-
Barley	-	2015-16	-	-	-	-	1341	-	-	-	-	-	-	-	-
CasterSeed	-	2015-16	-	-	-	-	-	-	-	-	-	3907	-	-	-
Chilly	Dry	2015-16	-	6200	14348	-	9063	8220	13937	15300	-	-	-	14500	-
Cotton	-	2015-16	-	4285	4061	-	-	5020	-	3982	-	-	-	4212	-
Ginger	Fresh	2015-16	-	-	4016	-	-	5750	5891	8000	5636	-	6537	10600	-
Gram	-	2015-16	-	4345	4719	-	3700	4300	5482	4000	4418	-	-	4460	4700
Ground Nut	-	2015-16	-	3700	4765	-	4892	-	-	-	-	-	-	-	-
Jowar	-	2015-16	Kharif	1395	-	-	2200	2523	2483	-	1733	-	-	1380	-
			Rabi	1489	-	-	-	-	-	-	-	-	-	1365	-
Linseed	-	2015-16	-	4461	5162	-	4000	4300	5940	-	4489	-	-	-	-
Maize	-	2015-16	-	1414	1316	1144	1218	-	-	1650	1450	-	1306	13271470	1500
Nigerseed		2015-16	-	-	-	-	-	-	-	-	-	-	-	-	-
Paddy	Coarse	2015-16	-	-	-	1483	1200	-	1402	-	1300	-	1750	-	1424
	Fine	2015-16	-	-	-	2075	-	-	2384	-	1814	-	-	-	1500
	Medium	2015-16	-	-	-	1545	1400	-	1950	-	-	-	-	-	1450
Potato	Hills	2015-16	Summer	-	-	-	-	-	-	-	-	-	-	-	-
			Winter	-	-	-	-	-	-	-	-	-	-	-	-
Rape/Mustard	-	2015-16	-	-	-	-	-	-	-	-	3450	-	-	-	2600
Rice	Coarse	2015-16	-	2958	3350	-	-	3571	2025	-	2400	-	1988	-	-
	Fine	2015-16	-	-	3750	-	2433	4000	3360	4029	3500	-	3785	-	-
	Medium	2015-16	-	-	3631	-	32400	3800	3000	-	2600	-	2520	-	-
Sannhemp	-	2015-16	-	-	-	-	-	-	-	-	2050	-	-	-	-
Sesamum	-	2015-16	Kharif	6200	-	-	7513	13855	13543	-	8800	-	-	-	10000
			Rabi	-	-	-	-	8625	-	-	-	-	-	-	-
Soyabean	-	2015-16	-	3218	3564	-	3215	3475	3293	3024	3656	-	-	-	-
Sugar Raw	-	2015-16	-	3550	3972	-	2920	-	-	4000	-	-	-	3900	-
Tobacco	-	2015-16	-	-	-	-	-	-	-	-	-	-	-	-	14000
Tur Arhar	Delay	2015-16	-	7904	-	-	7917	10000	-	12960	7950	-	-	15520	10000
	Early	2015-16	-	7135	-	-	9500	11030	-	8879	7860	-	-	-	-
Turmeric	-	2015-16	-	-	-	-	-	-	-	-	-	-	-	-	-
Wheat	-	2015-16	-	1585	1655	-	1476	-	1614	1700	1525	-	1598	1567	1800

Table 2.16 (Contd.) Crop-wise District-wise Farm Harvest Prices of Principal Crops in India - Madhya Pradesh

(Rs.per Quintal)

Crop Name	Variety	Year	Season $	Mandsaur	Morena	Narsinghpur	Panna	Raisen	Ratlam	Sagar	Satna	Sehore	Shahdol	Shajapur	Sheopur
Bajra	-	2015-16	-	-	1215	-	-	-	-	-	-	-	-	-	1509
Barley	-	2015-16	-	-	-	-	-	-	-	-	1255	-	1340	-	-
CasterSeed	-	2015-16	-	-	-	-	-	-	-	-	-	-	-	-	-
Chilly	Dry	2015-16	-	15569	-	-	-	-	9500	-	-	12917	-	10933	10782
Cotton	-	2015-16	-	-	-	-	-	-	4086	-	-	-	-	-	-
Ginger	Fresh	2015-16	-	-	-	5428	-	-	-	4538	5140	7480	-	-	-
Gram	-	2015-16	-	-	-	-	4224	4323	-	4364	4467	-	5200	4836	3550
Ground Nut	-	2015-16	-	6995	-	-	-	-	-	4773	-	-	-	3000	2971
Jowar	-	2015-16	Kharif	2423	-	2250	1950	1593	-	1642	2000	1501	-	2000	1500
			Rabi	-	-	-	-	-	-	-	-	-	-	-	-
Linseed	-	2015-16	-	-	-	-	4780	-	-	4964	4970	-	4000	5300	-
Maize	-	2015-16	-	1335	-	-	1434	2000	-	1529	-	1400	1500	-	-
Nigerseed		2015-16	-	-	-	3000	-	5036	-	-	-	-	3000	-	-
Paddy	Coarse	2015-16	-	-	-	-	-	1320	-	-	-	1750	-	-	-
	Fine	2015-16	-	-	-	-	-	2069	-	-	-	-	-	-	-
	Medium	2015-16	-	-	-	-	-	1626	-	-	-	-	-	-	-
Potato	Hills	2015-16	Summer	-	-	-	1385	-	-	-	-	-	1333	-	-
			Winter	-	-	-	1041	-	-	-	1057	-	1500	-	-
Rape/Mustard	-	2015-16	-	-	-	-	4000	-	-	-	-	-	4000	-	3160
Rice	Coarse	2015-16	-	-	2890	2385	2397	2615	-	2491	2309	-	2000	-	3500
	Fine	2015-16	-	-	3240	4113	3458	3030	-	4837	4409	4020	3000	-	3567
	Medium	2015-16	-	-	-	3582	2420	2810	-	2903	2987	2188	2500	-	3550
Sannhemp	-	2015-16	-	-	-	-	-	-	-	-	-	-	-	-	-
Sesamum	-	2015-16	Kharif	7318	9930	6929	9100	6300	-	10779	7314	-	9125	-	-
			Rabi	-	-	-	-	5508	-	-	-	-	-	-	-
Soyabean	-	2015-16	-	3670	-	3283	-	3542	3615	3509	-	3336	-	3408	3800
Sugar Raw	-	2015-16	-	-	-	2291	3348	-	-	-	-	3189	-	3000	4100
Tobacco	-	2015-16	-	-	-	13000	-	-	-	-	9500	-	10000	-	-
Tur Arhar	Delay	2015-16	-	5322	-	-	7540	13825	-	7263	7780	-	6000	-	9400
	Early	2015-16	-	5009	-	7755	8268	14750	-	8242	7333	7644	-	6667	9333
Turmeric	-	2015-16	-	-	-	-	-	-	-	-	-	-	16000	-	-
Wheat	-	2015-16	-	1864	-	-	1568	1980	-	1805	1540	-	1750	1514	1533

Table 2.16 (Contd.) Crop-wise District-wise Farm Harvest Prices of Principal Crops in India - Madhya Pradesh

(Rs.per Quintal)

Crop Name	Variety	Year	Season $	Shivani	Shivpuri	Sidhi	Tikamgarh	Umaria			
Bajra	-	2015-16	-	-	1452	-	-	-			
Barley	-	2015-16	-	-	-	1300	1357	-			
CasterSeed	-	2015-16	-	-	-	-	-	-			
Chilly	Dry	2015-16	-	-	15780	-	9494	13500			
Cotton	-	2015-16	-	-	-	-	-	-			
Ginger	Fresh	2015-16	-	-	-	-	4512	5900			
Gram	-	2015-16	-	3080	-	3340	5192	-			
Ground Nut	-	2015-16	-	6000	3574	-	-	-			
Jowar	-	2015-16	Kharif	-	-	1200	-	-			
			Rabi	-	-	-	-	-			
Linseed	-	2015-16	-	3080	-	4000	-	-			
Maize	-	2015-16	-	1274	1088	1053	-	1000			
Nigerseed		2015-16	-	-	-	-	-	-			
Paddy	Coarse	2015-16	-	-	-	1300	-	-			
	Fine	2015-16	-	-	2017	-	-	-			
	Medium	2015-16	-	-	-	-	-	-			
Potato	Hills	2015-16	Summer	-	1089	-	934	-			
			Winter	-	-	1180	828	-			
Rape/Mustard	-	2015-16	-	-	-	3200	-	-			
Rice	Coarse	2015-16	-	2306	-	2000	2338	-			
	Fine	2015-16	-	3300	-	3515	3275	5000			
	Medium	2015-16	-	2589	2516	2600	2588	3000			
Sannhemp	-	2015-16	-	-	-	-	-	-			
Sesamum	-	2015-16	Kharif	-	-	7125	6547	-			
			Rabi	-	-	-	-	-			
Soyabean	-	2015-16	-	3336	-	-	-	-			
Sugar Raw	-	2015-16	-	3525	3471	-	2926	-			
Tobacco	-	2015-16	-	-	-	-	-	15000			
Tur Arhar	Delay	2015-16	-	-	-	8250	-	-			
	Early	2015-16	-	-	-	8500	-	-			
Turmeric	-	2015-16	-	-	-	-	13710	11000			
Wheat	-	2015-16	-	-	-	1500	1537	-			

Table 2.17 Crop-wise District-wise Farm Harvest Prices of Principal Crops in India - Maharashtra

(Rs.per Quintal)

Crop Name	Variety	Year	Season $	Ahmednagar	Akola	Amravati	Aurangabad	Beed	Bhandara	Buldhana	Chandrapur	Dhule	Gadchiroli	Gondia	State Wtd Average
Bajra	-	2015-16	-	1460	1574	1666	1412	1510	-	1323	-	1330	-	-	1454
CasterSeed	-	2015-16	-	3640	3134	-	2875	2992	3280	-	2760	3138	-	-	3051
Gram	-	2015-16	-	3970	4269	4339	4068	4121	4300	4141	3926	4291	3313	4041	4169
Ground Nut	-	2015-16	-	3265	4644	4703	4490	4206	-	4644	4585	4729	-	-	3585
Jowar	-	2015-16	-	1687	1324	1446	1559	1894	-	1255	1350	1475	1248	-	1763
Linseed	-	2015-16	-	-	-	-	1590	1750	4333	-	4309	-	4067	4309	4290
Maize	-	2015-16	-	1321	1304	1335	1322	1391	-	1227	-	1297	1205	-	1351
Nigerseed		2015-16	-	4608	-	-	4608	4608	-	-	-	4608	-	-	5249
Paddy	-	2015-16	-	1642	-	1267	-	1051	1682	1267	1947	1380	1674	1535	1660
Ragi	-	2015-16	-	1678	-	-	-	-	-	-	-	1678	-	-	1837
Rape/Mustard	-	2015-16	-	3510	3056	3190	-	3606	2618	3246	2991	-	2991	1991	3656
Sesamum	-	2015-16	-	7785	6274	8127	8376	6963	5496	6483	5496	5968	5253	5496	6625
Soyabean	-	2015-16	-	3453	3390	3380	3541	3549	3159	3468	3409	3390	3280	-	3490
Sunflower	-	2015-16	-	3215	3279	2911	3164	3380	-	3180	-	3128	-	-	3446
Tur Arhar	-	2015-16	-	7344	7884	8038	7286	7431	6697	7703	7076	6765	7079	7290	7741
Wheat	-	2015-16	-	1718	1510	1569	1644	1839	1501	1529	1660	1580	1689	1689	1706

Table 2.17 (Contd.) Crop-wise District-wise Farm Harvest Prices of Principal Crops in India - Maharashtra

(Rs.per Quintal)

Crop Name	Variety	Year	Season $	Hingoli	Jalgaon	Jalna	Kolhapur	Latur	Nagpur	Nanded	Nandurbar	Nasik	Osmanabad	Parbhani	State Wtd Average
Bajra	-	2015-16	-	1409	1379	1400	1989	1460	-	1450	1308	1452	1474	1260	1454
CasterSeed	-	2015-16	-	3135	2525	3052	-	3002	2760	2800	3075	3160	3278	3156	3051
Gram	-	2015-16	-	4135	4111	4189	6178	4340	4152	4124	4118	4173	4200	4032	4169
Ground Nut	-	2015-16	-	-	4541	4424	2813	5491	4585	5024	3933	4731	3094	4902	3585
Jowar	-	2015-16	-	1564	1379	1493	2761	1768	1344	1339	1481	1565	1855	1453	1763
Linseed	-	2015-16	-	-	-	-	-	4871	4632	4511	-	-	4404	4511	4290
Maize	-	2015-16	-	1451	1320	1286	1666	-	1295	1393	1349	1383	1397	1350	1351
Nigerseed		2015-16	-	8786	4608	4608	4608	8028	-	8786	4608	4608	9100	8786	5249
Paddy	-	2015-16	-	-	-	-	1846	1022	1893	1181	1305	1859	1040	1051	1660
Ragi	-	2015-16	-	-	-	-	1678	-	-	-	2000	1678	-	-	1837
Rape/Mustard	-	2015-16	-	3100	4000	-	-	3640	3048	3505	4000	4000	3605	3610	3656
Sesamum	-	2015-16	-	6353	6366	6485	-	6152	6940	6990	8200	6000	6983	6558	6625
Soyabean	-	2015-16	-	3509	3302	3472	3563	3636	3421	3549	3553	3635	3586	3463	3490
Sunflower	-	2015-16	-	-	3169	2805	3440	3566	3110	3129	3999	3713	3345	3432	3446
Tur Arhar	-	2015-16	-	7973	6886	7525	7450	8697	7202	8028	7227	7809	7989	7347	7741
Wheat	-	2015-16	-	1760	1506	1674	2679	1920	1747	1513	1615	1596	2199	1616	1706

Table 2.17 (Contd.) Crop-wise District-wise Farm Harvest Prices of Principal Crops in India - Maharashtra

(Rs.per Quintal)

Crop Name	Variety	Year	Season $	Pune	Raigarh	Ratnagiri	Sangli	Satara	Sindhudurg	Solapur	Thane	Wardha	Washim	Yavatmal	State Wtd Average
Bajra	-	2015-16	-	1609	-	-	1666	1578	-	1463	-	-	1676	1582	1454
CasterSeed	-	2015-16	-	3100	-	-	3200	3200	-	3333	-	-	-	3134	3051
Gram	-	2015-16	-	4172	5222	-	4297	4135	-	4145	5222	3999	4201	4027	4169
Ground Nut	-	2015-16	-	4141	3957	3957	2862	2944	3957	4248	-	4585	4644	5093	3585
Jowar	-	2015-16	-	1960	-	-	2217	2018	-	1944	-	1359	1337	1184	1763
Linseed	-	2015-16	-	-	-	-	-	-	-	4000	-	5300	-	-	4290
Maize	-	2015-16	-	1449	-	-	1495	1447	-	1429	-	-	-	-	1351
Nigerseed		2015-16	-	4608	4608	4608	4608	4608	4608	4608	4608	-	-	-	5249
Paddy	-	2015-16	-	1642	1565	1503	2681	1959	1503	1532	1565	-	1267	-	1660
Ragi	-	2015-16	-	1678	2091	2091	-	1678	2091	-	2091	-	-	-	1837
Rape/Mustard	-	2015-16	-	4071	4000	4000	3704	3704	-	3510	4000	-	3142	-	3656
Sesamum	-	2015-16	-	6806	9671	9671	-	7785	9671	9200	9671	-	5430	-	6625
Soyabean	-	2015-16	-	3257	-	-	3562	3563	-	3643	-	3439	3542	3443	3490
Sunflower	-	2015-16	-	3193	3913	3913	3440	3440	3913	3446	3913	-	-	-	3446
Tur Arhar	-	2015-16	-	6636	7010	7010	7450	7450	7010	8049	7010	7689	7963	7843	7741
Wheat	-	2015-16	-	1915	-	-	2625	1745	-	1892	-	1515	1572	1520	1706

Table 2.18 Crop-wise District-wise Farm Harvest Prices of Principal Crops in India - Manipur

(Rs.per Quintal)

Crop Name	Variety	Year	Season $	Manipur	State Wtd Average										
Ginger	-	2015-16	Rabi	3500	3500										
Maize	-	2015-16	Rabi	1425	1425										
Paddy	-	2015-16	Rabi	1433	1433										
Potato	-	2015-16	Rabi	1211	1211										
Rape/Mustard	-	2015-16	Rabi	3000	3000										
Sugarcane	-	2015-16	Rabi	840	840										

Table 2.19 Crop-wise District-wise Farm Harvest Prices of Principal Crops in India - Mizoram

(Rs.per Quintal)

Crop Name	Variety	Year	Season $	Mizoram	State Wtd Average										
Banana	-	2015-16	-	1261	1261										
Chilly	-	2015-16	-	17010	17010										
Coconut	-	2015-16	-	930	930										
Cotton	-	2015-16	-	4520	4520										
Ginger	-	2015-16	-	1558	1558										
Maize	-	2015-16	-	1440	1440										
Paddy	-	2015-16	-	1912	1912										
Potato	-	2015-16	-	1902	1902										
Rape/Mustard	-	2015-16	-	3534	3534										
Rice	-	2015-16	-	2433	2433										
Sesamum	-	2015-16	-	5525	5525										
Soyabean	-	2015-16	-	6474	6474										
Sugar Raw	-	2015-16	-	4500	-										
Sugarcane	-	2015-16	-	921	921										
Tobacco	-	2015-16	-	20922	20922										
Tur Arhar	-	2015-16	-	3609	3609										
Turmeric	-	2015-16	-	1163	1163										

Table 2.20 Crop-wise District-wise Farm Harvest Prices of Principal Crops in India - Nagaland

(Rs.per Quintal)

Crop Name	Variety	Year	Season $	Nagaland	State Wtd Average										
Ginger	-	2015-16	-	3000	3000										
Maize	-	2015-16	-	1200	1200										

Table 2.21 Crop-wise District-wise Farm Harvest Prices of Principal Crops in India - Orissa

(Rs.per Quintal)

Crop Name	Variety	Year	Season $	Angul	Balasore	Bargarh	Bhadrak	Bolangir	Bouth	Cuttack	Deogarh	Dhenkanal	Gajpati	Ganjam	State Wtd Average
Ground Nut	-	2015-16	-	4027	4063	3325	4100	3200	4000	3900	-	4019	3400	3300	3672
Jute	-	2015-16	-	-	2900	-	2967	-	-	2985	-	-	-	-	2920
Maize	-	2015-16	-	1250	1350	1300	-	1265	-	1400	-	1300	1300	1325	1205
Paddy	-	2015-16	-	1361	1319	1330	1328	1289	1353	1310	1410	1360	1402	1359	1335
Potato	-	2015-16	-	906	800	875	867	-	925	900	950	900	833	825	871
Ragi	-	2015-16	-	1650	-	-	-	1600	1620	1585	-	1575	1500	1500	1511
Rape/Mustard	-	2015-16	-	4391	4263	4367	4300	4333	4300	4375	4300	4200	4367	-	4195
Sesamum	-	2015-16	-	4792	5081	5100	-	4950	5100	5000	-	4800	4500	4500	4684
Sugarcane	-	2015-16	-	267	258	233	257	245	266	250	-	250	245	230	240
Wheat	-	2015-16	-	1681	1538	1600	1565	1600	1600	1600	1575	1600	-	-	1651

Table 2.21 (Contd.) Crop-wise District-wise Farm Harvest Prices of Principal Crops in India - Orissa

(Rs.per Quintal)

Crop Name	Variety	Year	Season $	Jagatsinghpur	Jajpur	Jharsuguda	Kalahandi	Kandhamal	Kendrapara	Keonjhar	Khurda	Koraput	Malkangiri	Mayurbhanj	State Wtd Average
Ground Nut	-	2015-16	-	3700	3600	3500	3600	4000	3850	4183	3775	3600	4000	3729	3672
Jute	-	2015-16	-	3000	2975	-	-	-	2800	2900	-	-	-	-	2920
Maize	-	2015-16	-	1350	-	1320	1300	1375	-	1350	-	1320	1350	1283	1205
Paddy	-	2015-16	-	1285	1206	1361	1435	1363	1345	1340	1363	1279	1295	1308	1335
Potato	-	2015-16	-	860	833	1000	-	953	800	967	825	822	-	950	871
Ragi	-	2015-16	-	-	-	-	1600	1650	-	-	1685	1476	1463	-	1511
Rape/Mustard	-	2015-16	-	-	-	4350	4300	4295	4375	4233	4300	4300	-	4237	4195
Sesamum	-	2015-16	-	-	-	5100	5050	5000	-	5000	5000	5067	4500	4720	4684
Sugarcane	-	2015-16	-	250	250	-	262	-	248	260	250	235	-	-	240
Wheat	-	2015-16	-	-	1600	1600	1620	-	1620	1633	1650	-	1500	1620	1651

Table 2.21 (Contd.) Crop-wise District-wise Farm Harvest Prices of Principal Crops in India - Orissa

(Rs.per Quintal)

Crop Name	Variety	Year	Season $	Nabarangapur	Nayagarh	Nuapara	Puri	Rayagada	Sambalpur	Sonepur	Sundargarh	State Wtd Average			
Ground Nut	-	2015-16	-	4000	3863	4050	3800	4222	4200	4175	4200	3672			
Jute	-	2015-16	-	-	-	-	-	-	-	-	-	2920			
Maize	-	2015-16	-	1159	1199	-	-	1149	2160	-	-	1205			
Paddy	-	2015-16	-	1287	1360	1400	1354	1342	1356	1433	1274	1335			
Potato	-	2015-16	-	945	900	985	871	902	955	1000	1000	871			
Ragi	-	2015-16	-	1588	1675	1645	1700	1650	1665	1720	-	1511			
Rape/Mustard	-	2015-16	-	4000	4338	-	-	4400	4333	4427	4400	4195			
Sesamum	-	2015-16	-	-	4800	5100	4800	4700	4733	5100	4917	4684			
Sugarcane	-	2015-16	-	255	245	-	252	-	260	255	260	240			
Wheat	-	2015-16	-	1700	1550	-	-	-	1550	1575	1700	1651			

Table 2.22 Crop-wise District-wise Farm Harvest Prices of Principal Crops in India - Pondicherry

(Rs.per Quintal)

Crop Name	Variety	Year	Season $	Karaikal	Puducherry	Yanam	State Wtd Average								
Bajra	-	2015-16	-	-	1538	-	1538								
Cotton	-	2015-16	-	3559	2038	-	2799								
Ground Nut	-	2015-16	-	-	5339	-	5339								
Paddy	Fine	2015-16	-	1930	2170	1937	2170								
Ragi	-	2015-16	-	-	1642	-	1642								
Sesamum	-	2015-16	-	-	3868	-	3868								

Table 2.23 Crop-wise District-wise Farm Harvest Prices of Principal Crops in India - Punjab

(Rs.per Quintal)

Crop Name	Variety	Year	Season $	Amritsar	Barnala	Bhatinda	Faridkot	Fatehgarh Sahib	Fazilka	Firozpur	Gurdaspur	Hoshiarpur	Jalandhar	kapurthala	State Wtd Average
Barley	-	2015-16	-	-	-	1183	-	-	1050	-	-	-	-	-	1127
Cotton	American	2015-16	-	-	5400	5200	4300	-	4590	-	-	-	-	-	4691
	Desi	2015-16	-	-	-	3800	-	-	4350	-	-	-	-	-	4240
Gram	-	2015-16	-	-	-	3175	-	-	3175	-	-	3175	-	-	2808
Ground Nut	-	2015-16	-	-	-	-	-	-	-	-	-	4030	-	-	4030
Maize	-	2015-16	-	-	-	-	-	1225	-	-	1130	1325	1325	1325	1322
Sesamum	-	2015-16	-	9000	-	-	-	-	-	9000	-	-	-	-	9000
Sugarcane	-	2015-16	-	300	300	-	-	300	300	-	300	300	300	300	300
Wheat	-	2015-16	-	1500	1525	1400	1450	1525	1450	1400	1450	1450	1400	1450	1667

Table 2.23 (Contd.) Crop-wise District-wise Farm Harvest Prices of Principal Crops in India - Punjab

(Rs.per Quintal)

Crop Name	Variety	Year	Season $	Ludhiana	Mansa	Moga	Muktsar	Nawanshahar	Pathankot	Patiala	Rupnagar	S.A.S.Nagar	Sangrur	Tarean Taran	State Wtd Average
Barley	-	2015-16	-	1150	1183	-	1050	-	-	1150	-	-	1500	-	1127
Cotton	American	2015-16	-	-	4913	4590	4590	-	-	5200	-	-	5200	-	4691
	Desi	2015-16	-	-	-	-	-	-	-	-	-	-	-	-	4240
Gram	-	2015-16	-	-	3175	-	3175	-	5000	-	3175	3175	-	-	2808
Ground Nut	-	2015-16	-	-	-	-	-	-	-	-	-	-	-	-	4030
Maize	-	2015-16	-	1325	-	-	-	1225	1000	1325	1485	1225	1325	1130	1322
Sesamum	-	2015-16	-	-	-	-	-	-	9000	-	-	-	-	9000	9000
Sugarcane	-	2015-16	-	300	-	-	-	300	290	300	300	300	300	-	300
Wheat	-	2015-16	-	1450	1475	1400	1450	1450	1450	1400	1450	1400	1400	1400	1667

Table 2.24 Crop-wise District-wise Farm Harvest Prices of Principal Crops in India - Rajasthan

(Rs.per Quintal)

Crop Name	Variety	Year	Season $	Ajmer	Alwar	Banswara	Baran	Barmer	Bharatpur	Bhilwara	Bikaner	Bundi	Chittorgarh	Churu	State Wtd Average
Bajra	-	2015-16	Kharif	1319	1174	1336	1210	1404	1264	1249	1390	1444	-	1233	1267
Barley	-	2015-16	Rabi	1456	1345	1647	1322	830	1458	1517	1371	1410	1378	1451	1408
CasterSeed	-	2015-16	Rabi	5900	-	2663	-	3417	-	3212	-	-	-	-	3183
Chilly	Dry	2015-16	Kharif	12644	10151	10722	10715	-	-	12806	-	-	10963	-	10905
Cotton	-	2015-16	Kharif	4780	4308	4530	-	6250	4415	4493	-	-	4337	-	4565
	Lint	2015-16	Kharif	-	-	15935	-	-	-	8378	-	-	-	-	11026
Ginger	-	2015-16	Kharif	-	-	-	-	-	-	-	-	-	-	-	5062
Gram	-	2015-16	Rabi	5150	4566	4512	4963	4015	5355	5354	5366	4975	5389	5786	4318
Ground Nut	-	2015-16	Kharif	4414	5248	3406	4031	4629	3151	4077	3849	5023	4336	4613	3878
Jowar	-	2015-16	Kharif	1719	1150	1129	1608	1742	1664	1385	-	1803	1576	-	1807
Linseed	-	2015-16	Rabi	5900	-	-	4131	2444	-	2327	-	-	5168	-	3803
Maize	-	2015-16	Kharif	1580	1466	1342	1514	-	-	1455	-	1429	1334	-	1422
Paddy	-	2015-16	Kharif	-	-	1320	-	-	2239	-	-	-	-	-	1662
Potato	-	2015-16	Rabi	1950	915	1391	1451	-	932	1974	-	1748	824	-	939
Rape/Mustard	-	2015-16	Rabi	3978	3802	4230	3546	3971	3968	3515	3560	3747	3963	3729	3826
Rice	-	2015-16	Kharif	2686	2521	3890	2462	-	-	1783	-	2231	2958	-	2274
Sannhemp	-	2015-16	Kharif	-	-	2904	-	-	-	-	-	-	-	-	2429
Sesamum	-	2015-16	Kharif	8215	5410	7685	7083	6739	7429	8716	6188	7485	10221	6522	7046
Sugar Raw	-	2015-16	Rabi	3025	3230	3924	4158	-	3270	3569	-	-	3302	-	3323
Tur Arhar	-	2015-16	Rabi	-	6285	-	-	-	7243	-	-	9160	5575	-	6425
Wheat	-	2015-16	Rabi	1733	1541	-	1560	1554	1904	1525	1727	1623	1562	1592	1596

Table 2.24 (Contd.) Crop-wise District-wise Farm Harvest Prices of Principal Crops in India - Rajasthan

(Rs.per Quintal)

Crop Name	Variety	Year	Season $	Dausa	Dholpur	Dungarpur	Ganganagar	Hanumangarh	Jaipur	Jalor	Jhalawar	Jhunjunu	Jodhpur	Karauli	State Wtd Average
Bajra	-	2015-16	Kharif	1270	1177	1310	1352	1348	1310	1386	1500	1238	1344	1265	1267
Barley	-	2015-16	Rabi	1377	1451	-	1380	1225	-	1778	-	1428	1558	1457	1408
CasterSeed	-	2015-16	Rabi	-	-	-	-	3314	-	3028	-	-	3057	-	3183
Chilly	Dry	2015-16	Kharif	-	-	11648	-	12000	-	8000	11993	-	11864	-	10905
Cotton	-	2015-16	Kharif	-	-	-	4400	4503	-	6100	-	-	4928	-	4565
	Lint	2015-16	Kharif	-	-	-	-	11500	-	-	-	-	-	-	11026
Ginger	-	2015-16	Kharif	-	-	6762	-	-	-	-	-	-	-	-	5062
Gram	-	2015-16	Rabi	4659	6150	-	5193	4523	-	3500	-	5698	5069	5181	4318
Ground Nut	-	2015-16	Kharif	3865	6000	4773	-	3979	4320	3957	4195	4022	3926	-	3878
Jowar	-	2015-16	Kharif	1661	1132	1300	-	-	1730	1793	1817	-	1656	-	1807
Linseed	-	2015-16	Rabi	-	-	-	-	-	-	-	-	-	-	-	3803
Maize	-	2015-16	Kharif	1553	1460	1372	-	-	1690	1500	1322	-	1640	-	1422
Paddy	-	2015-16	Kharif	-	-	1664	-	1692	-	-	-	-	-	-	1662
Potato	-	2015-16	Rabi	-	957	-	-	518	-	925	-	-	-	-	939
Rape/Mustard	-	2015-16	Rabi	3838	3867	-	3839	3531	-	3311	-	3888	4246	3869	3826
Rice	-	2015-16	Kharif	-	-	2477	-	2875	-	-	2623	-	-	-	2274
Sannhemp	-	2015-16	Kharif	-	-	1305	-	-	-	-	-	-	-	-	2429
Sesamum	-	2015-16	Kharif	6667	9022	8404	-	6633	7100	6886	7399	-	6737	6625	7046
Sugar Raw	-	2015-16	Rabi	-	-	-	-	3014	-	-	-	-	-	-	3323
Tur Arhar	-	2015-16	Rabi	-	-	-	-	-	-	-	-	-	-	-	6425
Wheat	-	2015-16	Rabi	1693	1573	1451	-	1608	1506	1933	-	1642	2052	1577	1596

Table 2.24 (Contd.) Crop-wise District-wise Farm Harvest Prices of Principal Crops in India - Rajasthan

(Rs.per Quintal)

Crop Name	Variety	Year	Season $	Kota	Nagaur	Pali	Pratapgarh	Rajsamand	Sawai Madhopur	Sikar	Sirohi	Tonk	Udaipur	State Wtd Average
Bajra	-	2015-16	Kharif	-	1416	1414	-	1262	1257	1228	1571	1290	-	1267
Barley	-	2015-16	Rabi	1239	1528	1630	1335	1300	1492	1428	1756	1370	1416	1408
CasterSeed	-	2015-16	Rabi	-	-	3126	-	-	-	-	3349	2707	-	3183
Chilly	Dry	2015-16	Kharif	6828	10897	1160	-	9500	-	-	9371	-	12913	10905
Cotton	-	2015-16	Kharif	-	4822	4561	4385	4889	-	7937	4318	4150	4233	4565
	Lint	2015-16	Kharif	-	-	-	-	11214	-	-	-	-	-	11026
Ginger	-	2015-16	Kharif	-	-	-	-	-	-	-	-	-	4000	5062
Gram	-	2015-16	Rabi	3734	4791	5019	4631	3045	4756	4987	7306	4743	4365	4318
Ground Nut	-	2015-16	Kharif	3903	4010	5853	4380	4500	3578	4090	3897	3549	4155	3878
Jowar	-	2015-16	Kharif	2667	1964	1610	1469	1223	1362	-	1210	3940	1259	1807
Linseed	-	2015-16	Rabi	4152	-	-	3490	-	-	-	-	-	5550	3803
Maize	-	2015-16	Kharif	1373	1751	1610	1407	1530	1410	-	1676	1436	1458	1422
Paddy	-	2015-16	Kharif	-	-	-	-	-	-	-	-	-	1443	1662
Potato	-	2015-16	Rabi	1280	-	1060	-	1066	-	-	988	-	-	939
Rape/Mustard	-	2015-16	Rabi	3717	3894	4014	7097	2839	3829	3958	3972	3843	3312	3826
Rice	-	2015-16	Kharif	2382	-	-	2335	3440	3490	-	-	-	2053	2274
Sannhemp	-	2015-16	Kharif	-	-	-	-	-	-	-	-	-	2444	2429
Sesamum	-	2015-16	Kharif	6950	6924	7030	8582	10500	7620	8271	5862	6603	9822	7046
Sugar Raw	-	2015-16	Rabi	3941	-	-	-	2632	-	-	-	-	3935	3323
Tur Arhar	-	2015-16	Rabi	8076	-	-	-	-	6923	-	6585	-	-	6425
Wheat	-	2015-16	Rabi	1522	1904	1906	1610	1626	1527	1725	1895	1511	1607	1596

Table 2.25 Crop-wise District-wise Farm Harvest Prices of Principal Crops in India - Tamil Nadu

(Rs.per Quintal)

Crop Name	Variety	Year	Season $	Ariyalur	Coimbatore	Cuddalore	Dharmapuri	Dindugal	Erode	Kanchipuram	Kanyakumari	Karur	Krishnagiri	Madurai	State Wtd Average
Arecanut	-	2015-16	-	-	20688	-	20688	20688	20688	-	20688	-	20688	20688	20861
Banana	-	2015-16	-	3388	3388	2500	2836	1671	1682	2500	1269	2321	3388	2298	2403
Cashewnut	-	2015-16	-	10860	10860	10860	-	10860	10860	10860	10860	10860	-	10860	10860
CasterSeed	-	2015-16	-	6746	3329	2334	3693	3892	3329	-	-	3329	6746	6746	4391
Chilly	-	2015-16	-	10725	8025	13761	12516	9906	13043	11813	-	8848	12514	9906	10858
Cotton	-	2015-16	-	4700	4700	4279	4700	3874	4700	4279	-	4700	4700	3874	4326
Gram	-	2015-16	-	-	6311	-	5700	6190	-	6017	-	-	-	5683	5933
Ground Nut	-	2015-16	-	4000	4860	5668	4860	4771	6518	5668	4527	4000	4860	4771	5242
Maize	-	2015-16	-	1612	1709	1843	1398	1495	2498	1989	1688	1746	2275	1602	1620
Paddy	-	2015-16	-	1341	1318	1328	1318	1417	1318	1293	1439	1341	1318	1225	1333
Potato	-	2015-16	-	-	-	-	-	1831	1831	-	-	-	1794	-	1828
Ragi	-	2015-16	-	1913	2056	2245	1937	1670	2463	2161	2235	2506	2132	2115	2056
Sesamum	-	2015-16	-	8973	7712	6180	8119	7116	7683	6180	-	8973	8973	7116	7491
Tapioca	-	2015-16	-	1053	2118	1543	1757	1053	1053	1543	1932	2118	1826	1053	1561
Turmeric	-	2015-16	-	13447	10560	8000	12005	10673	8702	-	-	10061	13921	8000	10074

Table 2.25 (Contd.) Crop-wise District-wise Farm Harvest Prices of Principal Crops in India - Tamil Nadu

(Rs.per Quintal)

Crop Name	Variety	Year	Season $	Nagapattinam	Namakkal	Nilgiris	Perambalur	Pudukkottai	Ramanathapuram	Salem	Sivaganga	Thanjavur	Theni	Thirunelveli	State Wtd Average
Arecanut	-	2015-16	-	-	22000	20688	20688	-	-	20688	-	20688	20688	20688	20861
Banana	-	2015-16	-	1831	2836	3388	3388	1928	3388	2836	1671	2148	3388	1452	2403
Cashewnut	-	2015-16	-	10860	10860	-	-	10860	10860	10860	10860	10860	10860	10860	10860
CasterSeed	-	2015-16	-	-	3693	-	6746	-	-	3807	-	-	6746	3900	4391
Chilly	-	2015-16	-	12314	11813	-	8848	13445	8848	13147	8848	12314	9906	11587	10858
Cotton	-	2015-16	-	4700	4700	3874	4700	3874	3874	4279	3874	4700	3874	3874	4326
Gram	-	2015-16	-	-	6900	-	-	-	5641	5641	-	-	5641	-	5933
Ground Nut	-	2015-16	-	5000	5213	4527	4000	4740	4771	5107	4771	5000	4771	4527	5242
Maize	-	2015-16	-	1602	1538	-	1612	1440	1575	1500	1575	1830	1679	1396	1620
Paddy	-	2015-16	-	1270	1318	1439	1341	1428	1372	1318	1372	1383	1417	1372	1333
Potato	-	2015-16	-	-	-	1831	-	-	-	-	-	-	-	-	1828
Ragi	-	2015-16	-	-	1991	2043	1913	2231	1670	1857	1838	2216	1670	1838	2056
Sesamum	-	2015-16	-	8973	8119	-	8973	8973	7116	8119	7116	8973	7116	7116	7491
Tapioca	-	2015-16	-	1932	1543	1932	1053	1053	1932	1543	1053	1053	1053	1932	1561
Turmeric	-	2015-16	-	12288	10531	8000	10531	12500	-	9105	10883	12930	10723	10883	10074

Table 2.25 (Contd.) Crop-wise District-wise Farm Harvest Prices of Principal Crops in India - Tamil Nadu

(Rs.per Quintal)

Crop Name	Variety	Year	Season $	Thiruvanna malai	Thiruvarur	Thoothukudi	Tiruchirappa lli	Tiruppur	Tiruvallur	Vellore	Viluppuram	Virudhunag ar	State Wtd Average		
Arecanut	-	2015-16	-	20688	20688	-	20688	20688	-	20688	20688	20688	20861		
Banana	-	2015-16	-	3388	2148	1967	2373	3388	2500	2540	2500	3000	2403		
Cashewnut	-	2015-16	-	-	10860	10860	10860	10860	10860	10860	10860	10860	10860		
CasterSeed	-	2015-16	-	2334	-	3382	3329	3329	2334	2334	2334	-	4391		
Chilly	-	2015-16	-	11813	-	11446	13097	13256	11813	11813	12814	9165	10858		
Cotton	-	2015-16	-	4279	4700	3874	4700	4700	4279	4279	4279	3874	4326		
Gram	-	2015-16	-	5641	-	6642	6157	5751	5641	5688	-	5641	5933		
Ground Nut	-	2015-16	-	5648	5000	4527	4740	6518	5668	5648	4860	4771	5242		
Maize	-	2015-16	-	1340	-	1625	1612	1658	1989	1496	1649	1575	1620		
Paddy	-	2015-16	-	1318	1297	1439	1341	1318	1293	1318	1318	1372	1333		
Potato	-	2015-16	-	-	-	-	-	1831	-	-	-	-	1828		
Ragi	-	2015-16	-	2232	-	2043	1913	-	2161	1964	1792	1799	2056		
Sesamum	-	2015-16	-	8119	8973	7116	8973	7511	6180	6180	6180	7116	7491		
Tapioca	-	2015-16	-	1543	1932	1932	1757	2118	1543	1543	1543	1932	1561		
Turmeric	-	2015-16	-	10531	-	12365	11943	10091	10531	10531	11884	10883	10074		

Table 2.26 Crop-wise District-wise Farm Harvest Prices of Principal Crops in India - Telangana

(Rs.per Quintal)

Crop Name	Variety	Year	Season $	Telangana	Adilabad	Karimnagar	Khammam	Mahabubnagar	Medak	Nalgonda	Nizamabad	Rangareddy	Warangal	State Wtd Average
Bajra	-	2015-16	-	1539	-	-	-	-	-	-	-	-	-	1539
			Kharif	-	-	-	-	1807	-	1919	-	-	-	1863
			Rabi	-	1350	-	-	-	-	-	1518	-	-	1434
CasterSeed	-	2015-16	-	3286	-	-	-	-	-	-	-	-	-	3286
			Kharif	-	-	-	-	3287	-	3290	-	-	2989	3189
Chilly	Dry	2015-16	-	10343	-	-	-	-	-	-	-	-	-	10343
			Kharif	-	9968	12461	10107	5019	-	-	-	-	11156	9742
			Rabi	-	-	12461	13971	-	-	11338	-	-	11670	12360
Cotton	-	2015-16	-	3932	-	-	-	-	-	-	-	-	-	3932
			Kharif	-	3982	3980	3796	3973	3861	3973	4106	3861	3921	3939
			Rabi	-	-	-	-	-	3861	-	-	-	-	3861
Gram	-	2015-16	-	4489	-	-	-	-	-	-	-	-	-	4489
			Rabi	-	4580	4066	-	4357	4066	-	4580	4536	5043	4461
Ground Nut	-	2015-16	-	4544	-	-	-	-	-	-	-	-	-	4544
			Kharif	-	-	-	8750	4222	-	4371	-	-	3980	5331
			Rabi	-	4956	5007	5777	4322	4889	5124	4956	4889	4515	4937
Jowar	-	2015-16	-	2092	-	-	-	-	-	-	-	-	-	2092
			Kharif	-	1819	-	-	1879	2623	2218	1820	2558	-	2057
			Rabi	-	1819	-	-	2143	2623	3180	1820	2623	2550	2394
Maize	-	2015-16	-	1367	-	-	-	-	-	-	-	-	-	1367
			Kharif	-	1369	1343	1526	1348	1448	2206	1369	1448	1215	1475
			Rabi	-	1309	1388	1284	1314	1306	-	1309	1306	1376	1324
Paddy	-	2015-16	-	-	1347	1404	1489	1428	1428	1429	1503	1494	1424	1439
			Kharif	-	1289	1358	1606	1380	1407	1485	1644	1273	1399	1427
			Rabi	-	1404	1449	1371	1476	1449	1372	1362	1715	1449	1450
Ragi	-	2015-16	-	1644	-	-	-	-	-	-	-	-	-	1644
			Kharif	-	-	-	-	1756	-	-	-	1472	-	1614
			Rabi	-	-	-	-	-	-	-	-	1678	-	1678
Sesamum	-	2015-16	-	6711	-	-	-	-	-	-	-	-	-	6711
			Kharif	-	-	-	-	-	-	6545	-	-	7936	7241
			Rabi	-	6695	6695	-	-	-	-	6695	-	-	6695
Sugar Raw	-	2015-16	-	3164	-	-	-	-	-	-	-	-	-	3164
			Kharif	-	-	2900	3100	2900	3250	2900	3200	3000	-	3036
Tobacco	-	2015-16	-	14275	-	-	-	-	-	-	-	-	-	14275
			Kharif	-	-	-	-	14275	-	-	14275	-	-	14275
			Rabi	-	-	-	14275	14275	-	-	14275	-	-	14275

Table 2.26 (Contd.) Crop-wise District-wise Farm Harvest Prices of Principal Crops in India - Telangana

(Rs.per Quintal)

Crop Name	Variety	Year	Season $	Telangana	Adilabad	Karimnagar	Khammam	Mahabubnagar	Medak	Nalgonda	Nizamabad	Rangareddy	Warangal	State Wtd Average	
Tur Arhar	-	2015-16	-	8609	-	-	-	-	-	-	-	-	-	8609	
			Kharif	8615	-	-	-	-	-	-	-	-	-	8615	
			Rabi	-	7405	7293	-	-	-	-	-	-	7997	7565	
Turmeric	-	2015-16	-	7914	-	-	-	-	-	-	-	-	-	7914	
			Kharif	-	7578	8058	-	-	8058	-	7578	8058	8292	7937	

Table 2.27 Crop-wise District-wise Farm Harvest Prices of Principal Crops in India - Tripura

(Rs.per Quintal)

Crop Name	Variety	Year	Season $	Tripura	State Wtd Average										
Cotton	-	2015-16	-	4400	4400										
Gram	-	2015-16	-	7615	7615										
Jute	-	2015-16	-	2985	2985										
Maize	-	2015-16	-	2637	2637										
Paddy	-	2015-16	-	1399	1399										
Potato	-	2015-16	-	968	968										
Rape/Mustard	-	2015-16	-	6589	6589										
Sesamum	-	2015-16	-	6738	6738										

Table 2.28 Crop-wise District-wise Farm Harvest Prices of Principal Crops in India - Uttar Pradesh

(Rs.per Quintal)

Crop Name	Variety	Year	Season $	Agra	Azamgarh	Bagpat	Bahraich	Balrampur	Banda	Basti	Bijnor	Chitrakoot	Etah	Farrukhabad	State Wtd Average
Bajra	-	2015-16	Kharif	-	3500	-	-	-	1400	1425	1208	1300	1159	-	1417
Barley	-	2015-16	Rabi	-	1445	1621	-	-	1398	-	-	-	1394	-	1384
Cotton	-	2015-16	Kharif	4635	6500	-	-	-	-	-	-	-	-	-	5568
Gram	-	2015-16	Rabi	-	4113	-	-	5695	6128	-	-	-	4681	-	5603
Ground Nut	-	2015-16	Kharif	-	-	-	-	-	4500	-	-	-	-	-	6162
Jowar	-	2015-16	Kharif	1240	1000	-	-	-	2450	1120	1560	2000	-	-	1426
Linseed	-	2015-16	Rabi	-	-	-	-	-	6030	-	-	5399	-	-	4238
Maize	-	2015-16	Kharif	1170	1480	-	1180	1298	-	1081	1200	-	1265	-	1211
Paddy	-	2015-16	Kharif	1560	1330	1650	1270	1120	1420	1250	1300	1600	1580	-	1317
Potato	-	2015-16	Rabi	799	-	-	-	-	-	-	-	-	-	436	688
Rape/Mustard	-	2015-16	Rabi	3480	-	-	-	3579	-	-	-	-	3519	-	3623
Sesamum	-	2015-16	Kharif	-	-	-	-	-	6000	-	-	7000	7189	-	6365
Soyabean	-	2015-16	Kharif	-	-	-	-	-	-	-	-	-	-	-	2640
Sugar Raw	-	2015-16	Kharif	-	-	2544	-	3212	-	3500	2705	-	-	-	2955
Sugarcane	-	2015-16	Kharif	-	-	280	-	256	-	230	240	-	-	-	248
Tobacco	-	2015-16	Rabi	-	-	-	-	-	-	-	-	-	5236	-	3732
Turmeric	-	2015-16	Rabi	-	-	-	1094	-	-	-	-	-	-	-	3354
Wheat	-	2015-16	Rabi	1528	1413	1582	1434	1515	1527	1456	1568	1403	1553	-	1438

Table 2.28 (Contd.) Crop-wise District-wise Farm Harvest Prices of Principal Crops in India - Uttar Pradesh

(Rs.per Quintal)

Crop Name	Variety	Year	Season $	Fatehpur	Firozabad	Gautam Budha Nagar	Ghazipur	Gonda	Hamirpur	Hardoi	Hathras (M.M.Nagar)	Jhansi	Kannauj	Kaushambi	State Wtd Average
Bajra	-	2015-16	Kharif	1192	-	-	-	-	-	-	-	1210	-	1300	1417
Barley	-	2015-16	Rabi	1505	1341	-	1533	-	-	-	-	-	-	1899	1384
Cotton	-	2015-16	Kharif	-	-	-	-	-	-	-	-	-	-	-	5568
Gram	-	2015-16	Rabi	6466	-	-	6315	5444	5079	-	-	-	-	7640	5603
Ground Nut	-	2015-16	Kharif	-	-	-	-	-	-	-	-	-	-	-	6162
Jowar	-	2015-16	Kharif	1480	-	-	1021	-	2100	1255	-	1420	-	-	1426
Linseed	-	2015-16	Rabi	-	-	-	-	-	-	-	-	-	-	-	4238
Maize	-	2015-16	Kharif	-	-	-	-	-	-	1152	-	-	-	-	1211
Paddy	-	2015-16	Kharif	1150	-	1850	1303	-	-	1280	-	1140	-	1260	1317
Potato	-	2015-16	Rabi	1069	-	-	674	-	-	746	807	-	428	1208	688
Rape/Mustard	-	2015-16	Rabi	4435	-	-	-	3309	3578	-	-	-	-	-	3623
Sesamum	-	2015-16	Kharif	5864	-	-	-	-	6200	7200	-	-	-	-	6365
Soyabean	-	2015-16	Kharif	-	-	-	-	-	-	-	-	2640	-	-	2640
Sugar Raw	-	2015-16	Kharif	-	-	-	-	3140	-	2800	-	-	-	-	2955
Sugarcane	-	2015-16	Kharif	-	-	-	-	285	-	245	-	-	-	-	248
Tobacco	-	2015-16	Rabi	-	-	-	-	-	-	2982	-	-	-	-	3732
Turmeric	-	2015-16	Rabi	-	-	-	-	-	-	-	-	-	-	-	3354
Wheat	-	2015-16	Rabi	1261	-	1672	1539	1409	1482	1184	-	-	-	1546	1438

Table 2.28 (Contd.) Crop-wise District-wise Farm Harvest Prices of Principal Crops in India - Uttar Pradesh

(Rs.per Quintal)

Crop Name	Variety	Year	Season $	Lalitpur	Mahoba	Mainpuri	Mathura	Mirzapur	Moradabad	Sant Kabir Nagar	Shrawasti	Siddharthnagar	Sitapur	Sonbhadra	State Wtd Average
Bajra	-	2015-16	Kharif	-	-	-	-	1130	-	1200	-	-	-	-	1417
Barley	-	2015-16	Rabi	1191	1487	-	1432	1293	-	-	-	-	-	-	1384
Cotton	-	2015-16	Kharif	-	-	-	-	-	-	-	-	-	-	-	5568
Gram	-	2015-16	Rabi	4529	-	-	-	4711	-	-	-	-	-	-	5603
Ground Nut	-	2015-16	Kharif	4250	-	-	-	12100	-	-	-	-	3800	-	6162
Jowar	-	2015-16	Kharif	-	-	-	-	1000	-	1140	-	-	1180	-	1426
Linseed	-	2015-16	Rabi	-	5510	-	-	3741	-	-	-	2790	-	4197	4238
Maize	-	2015-16	Kharif	1190	-	-	-	-	-	1330	1050	-	1255	1092	1211
Paddy	-	2015-16	Kharif	-	-	-	-	1300	1266	1300	1080	1130	1080	-	1317
Potato	-	2015-16	Rabi	-	-	726	-	-	-	-	-	-	745	-	688
Rape/Mustard	-	2015-16	Rabi	2970	3957	3523	-	7315	3157	-	4505	-	-	3821	3623
Sesamum	-	2015-16	Kharif	5200	5500	-	-	-	-	-	-	-	7450	6050	6365
Soyabean	-	2015-16	Kharif	-	-	-	-	-	-	-	-	-	-	-	2640
Sugar Raw	-	2015-16	Kharif	-	-	-	-	-	-	2755	-	-	2950	-	2955
Sugarcane	-	2015-16	Kharif	-	-	-	-	-	-	270	-	-	240	-	248
Tobacco	-	2015-16	Rabi	-	-	-	-	-	-	-	-	-	2978	-	3732
Turmeric	-	2015-16	Rabi	-	-	-	-	-	-	-	-	-	3580	-	3354
Wheat	-	2015-16	Rabi	1394	1589	1458	1499	1426	1505	1438	1563	1459	1374	1426	1438

Table 2.29 Crop-wise District-wise Farm Harvest Prices of Principal Crops in India - Uttranchal

(Rs.per Quintal)

Crop Name	Variety	Year	Season $	Dehradoon	Haridwar	Nanital	Udamsingh nagar	State Wtd Average							
Barley	-	2015-16	Rabi	1561	-	-	-	1561							
Ground Nut	-	2015-16	Kharif	-	5375	-	-	5375							
Maize	-	2015-16	Kharif	-	1551	1227	1405	1495							
Paddy	-	2015-16	Kharif	-	2138	1253	1305	1390							
Ragi	-	2015-16	Kharif	2964	-	-	-	2964							
Rape/Mustard	-	2015-16	Rabi	-	-	3446	3544	3495							
Sesamum	-	2015-16	Kharif	-	7381	-	-	7381							
Soyabean	-	2015-16	Kharif	-	-	3379	-	3379							
Sugar Raw	-	2015-16	Kharif	-	3164	3221	4182	3522							
Sugarcane	-	2015-16	Kharif	-	288	276	282	286							
Wheat	-	2015-16	Rabi	1568	1598	1775	1577	1629							

Table 2.30 Crop-wise District-wise Farm Harvest Prices of Principal Crops in India - West Bengal

(Rs.per Quintal)

Crop Name	Variety	Year	Season $	Bardhaman	Birbhum	Dakhin Dinajpur	Darjiling	East Midnapore	Hooghly	Howrah	Malda	Murshidabad	Nadia	North 24 Parganas	State Wtd Average
Gram	-	2015-16	-	5450	-	5820	-	-	-	-	6376	4789	-	6100	5511
Jute	-	2015-16	-	2375	-	4880	-	-	3060	2250	5457	3436	5600	-	4448
Paddy	-	2015-16	-	1400	1250	1505	1500	-	1140	1450	1550	1560	1650	1800	1526
Potato	-	2015-16	-	1450	1250	1449	1296	-	-	1192	1470	1533	1558	1565	1296
Rape/Mustard	-	2015-16	-	4800	4800	4641	3391	-	-	-	4290	3611	4250	3967	4147
Wheat	-	2015-16	-	1650	1625	1680	1720	1700	-	-	1643	1750	-	-	1690

Table 2.30 (Contd.) Crop-wise District-wise Farm Harvest Prices of Principal Crops in India - West Bengal

(Rs.per Quintal)

Crop Name	Variety	Year	Season $	Puruliya	South 24 Parganas	West Midnapore	State Wtd Average								
Gram	-	2015-16	-	-	-	-	5511								
Jute	-	2015-16	-	-	-	-	4448								
Paddy	-	2015-16	-	1260	1780	1775	1526								
Potato	-	2015-16	-	-	1500	975	1296								
Rape/Mustard	-	2015-16	-	-	4533	4500	4147								
Wheat	-	2015-16	-	-	-	-	1690								

* State Weighted Average Provided by State Authority. $ The icon (-) is given wherever state has not mentioned specific season.

A P P E N D I C E S

APPENDIX-I

METHODOLOGY FOR COLLECTION OF FARM HARVEST PRICES

1. Farm Harvest prices of a commodity as reported in this publication is defined as the average wholesale price, at which the commodity is disposed of by the producer to the trader at the village site during the specified marketing period after the commencement of harvest.

2. The information on Farm Harvest Prices is provided by State Governments and the following procedure is followed by them for systematising the collection and compilation of these statistics:-

a) A certain number of representative villages are selected in each district at the rate of one, two or three villages from each Tehsil, depending upon the extent to which the crop is grown in the Tehsil. The total number of villages to be selected in each district should, however, not be less than ten.

b) In each selected village, the price at which the commodity is sold by the producer is recorded in the specified form (p.82), by the price reporter on every Friday during the peak period of marketing after the commencement of the harvesting season. If no sales take place on that Friday, the price at which the commodity was sold last during the week is recorded instead.

c) In cases, where village site transactions do not take place, the price reported relate to what the farmer receives for his produce, and is obtained by subtracting transport and other marketing charges from the wholesale prices quoted at the mandi where produce is disposed of.

d) The price recorded is the wholesale price of the specified variety of the commodity and is expressed in terms of Rupees and paise per quintal (net weight) exclusive of gunny bags / container.

3. The existence of different varieties and qualities of commodities with a wide variation in prices makes the task of giving a single harvest price for a commodity for the State as a whole difficult. In each district, however, it may be possible to determine a particular variety which is grown to the largest extent, and that variety is specified for the purpose of noting the farm harvest prices. In some cases, it may be necessary to give the farm harvest prices of two varieties separately if the difference in price is large, as for instance, in the case of prices of Bt Cotton and Desi cotton.

4. It is also necessary to fix the reference period of harvest and the subsequent peak period of marketing for each crop, as these tend to vary widely depending upon the variety of the crop and the nature of cultivation. In some cases, it is even difficult to define strictly the harvesting period, as for instance, in the case of cotton where there are more than four pickings in a year.

These periods, however, are fixed in respect of each-crop and each State by the State Governments having due regard to the local conditions. Usually, in case of most commodities, six to eight weeks during the peak period of marketing after the commencement of the harvest season is taken to be the period during which farmers are generally expected to dispose of their produce.

5. Data on farm harvest prices are viewed as an integral part of agricultural statistics. They need to be collected through the same agency employed for the collection of statistics of area and yield. The Supervisors, Kanungos, Revenue Inspectors or the equivalent officials, who are generally employed by the state government for the collection of statistics of area and yield are also entrusted with the task of collecting these statistics. However, in view of the wide divergences in the revenue machinery of different States, the choice of the reporting agency is left to the decision of the State Government. The price reporters are required to visit the selected villages on the appointed dates, enquire personally the prices at which the commodity has been sold by the farmer and record them in the specified form.

6. The method of arriving at the average price of a commodity for the State as a whole is the method of weighted average, with the district production figures for the concerned year as weights. However, the average price for the district for each week may be obtained as a simple arithmetic average of the Tehsil prices which in turn, are the simple arithmetic average of village prices. The average price for the season is the simple arithmetic average or the district prices for each commodity. The computation of the district average price is done at the district headquarters while the prices for the State as a whole is worked out at State headquarters.

7. The farm harvest prices are required to be collected in respect of all the important crops and, in any case, they are required to be submitted for all the crops which come under the purview of advance estimation. As the final estimate of every crop is published at least one month after the completion of harvesting, it may be feasible to get the farm harvest prices ready by the time of the final estimate.

FORM

VILLAGE:

CIRCLE:

TALUKA:

DISTRICT:

PRICE PREVAILING ON ……………………………………

COMMODITY	QUALITY	WHOLESALE PRICE (RS. PER QUINTAL)
1.		
2.		
3.		

NAME OF THE REPORTER:

DUE DATE: DESIGNATION:

DATE OF DESPATCH: REASON FOR DELAY:

DATE OF RECEIPT:

SIGNATURE OF REPORTER:

DEPTT. HEADQUARTERS:

Harvesting Season of Principal Crops in Major Growing States

STATE	PADDY/RICE (KHARIF)	PADDY/RICE (RABI)	PADDY/RICE (SUMMER)
Andhra Pradesh	Nov - Dec	Mar - Apr	Jul – Aug
Assam	Jun - Jul	Nov - Dec	May- Jun
Bihar	Sep - Nov	Apr - May	Jul – Aug
Gujarat	Oct - Nov		
Haryana	Sep - Oct		
Himachal Pradesh	Oct		
Jammu & Kashmir	Sep - Oct		
Karnataka	Sep - Oct	Jan - Feb	May – Jun
Kerala	Sep - Oct	Dec -Jan	Mar – Apr
Madhya Pradesh	Oct - Nov		
Maharashtra	Oct - Nov		
Meghalaya			
Nagaland			
Odisha	Sep - Oct	Nov -Dec	Apr – May
Punjab	Oct - Nov		
Rajasthan	Oct - Nov		
Tamilnadu	Sep - Oct	Jan - Feb	May – Jun
Telangana	Nov -Dec	Mar – Apr	
Uttar Pradesh	Oct - Nov	Apr – May	
Uttarakhand			
West Bengal	Aug - Nov		Apr – May
ALL - INDIA	Sep - Jan	Apr – May	Mar - Aug

Harvesting Season of Principal Crops in Major Growing States

STATE	BAJRA (KHARIF)	BAJRA (SUMMER)	WHEAT (RABI)
Andhra Pradesh	Sep – Oct		
Assam			Mar(E) - Apr(E)
Bihar			Mar(E) - Apr(E)
Gujarat	Jun(M) - Jul(M)	May(M) - Jun(E)	Feb(E) - Mar(E)
Haryana	Sep(E) - Sep(L)		Apr(B) - May(B)
Himachal Pradesh			Apr (M) - Jun(E)
Jammu & Kashmir			Apr (E) - Jun(E)
Karnataka	Sep(L) - Oct(M)	Apr(L) - May(E)	Feb(B) - Mar(E)
Kerala			
Madhya Pradesh	Oct(E) - Oct(L)	Jun(E) – Jun(M)	Feb (E) – Apr(M)
Maharashtra	Sep(L) - Oct(E)	May(E) – May(M)	Feb(B) – Mar(E)
Meghalaya			
Nagaland			
Odisha			Mar - Apr
Punjab	Oct(M) – Oct (L)		Apr(M) -May(B)
Rajasthan	Sep(L) - Oct(M)	May(L) – Jun(M)	Mar(B) - Apr(E)
Tamilnadu	Sep(M) – Oct(M)	May(M) - Jun(M)	
Telangana	Jul – Aug		
Uttar Pradesh	Oct(E) - Oct(M)	May(L) - Jun(E)	Mar(M) - Apr(E)
Uttarakhand			
West Bengal			Mar(B) - Apr(B)
ALL - INDIA	Sep - Nov	Apr – May	Feb - Jun

Harvesting Season of Principal Crops in Major Growing States

STATE	ARHAR / TUR (Early Duration)	ARHAR / TUR (Mid Early Duration)
Andhra Pradesh	Nov(M) - Nov(E)	Dec(M) - Dec(E)
Assam		
Bihar	Nov (B) - Nov(M)	
Chattisgarh	Nov(B) - Nov(M)	
Gujarat	Nov(M) - Nov(E)	Dec(M) - Dec (E)
Haryana	Oct (M) – Oct(E)	
Himachal Pradesh		
Jammu & Kashmir		
Karnataka	Nov(M) - Nov(E)	Dec(M) - Dec(E)
Kerala		
Madhya Pradesh	Nov (M)	Nov(E) - Dec(E)
Maharashtra	Nov(M) - Nov(E)	Dec(M) - Dec(E)
Meghalaya		
Manipur		
Nagaland	Nov(B) - Nov(M)	
Odisha	Nov(M) - Nov(E)	
Punjab	Oct (M) – Oct (E)	
Jharkhand	Nov(M) - Nov(E)	
Rajasthan	Nov(B) - Nov(M)	
Tamilnadu	Nov(B) - Nov(M)	Dec(M) - Dec (E)
Tripura	Nov(B) - Nov(M)	
Uttar Pradesh	Nov(B) - Nov(M)	
Uttarakhand	Nov(M) - Nov(E)	
ALL - INDIA	Nov(B) - Nov(M)	Nov(M) - Dec (E)

Harvesting Season of Principal Crops in Major Growing States

STATE	ARHAR / TUR (Medium Duration)	ARHAR / TUR (Long Duration)	SOYABEAN (KHARIF)
Andhra Pradesh	Jan (B) – Jan (M)		Sep (E) – Oct (B)
Assam			
Bihar	Jul(B) – Jul (M)	Apr (M) - Apr (E)	Sep (E) – Oct (M)
Chattisgarh	Dec (E) - Jan (M)		Sep (E) – Oct (B)
Gujarat	Jan (B) – Jan (M)		Sep (E) – Oct (M)
Haryana			
Himachal Pradesh			
Jammu & Kashmir			
Karnataka	Jan(B) - Jan (M)		Sep (E) – Oct (B)
Kerala			
Madhya Pradesh	Jan (B) – Jan (M)		Sep (E) – Oct (B)
Maharashtra	Jan (B) - Jan (M)		Sep (E) – Oct (B)
Meghalaya			Sep (E) – Oct (B)
Manipur			Sep (E) – Oct (B)
Nagaland			
Odisha	Jan (B) – Jan (M)		Sep (E) – Oct (M)
Punjab			
Jharkhand	Jan (B) – Jan (M)	Mar (M) - Apr (B)	Sep (E) – Oct (M)
Rajasthan			Sep (E) – Oct (B)
Tamilnadu	Jan (B) – Jan (M)		
Tripura			
Uttar Pradesh	Jul(B) – Jul(M)	Apr (B) - Apr (M)	Sep (E) – Oct (B)
Uttarakhand			
West Bengal			Sep (E) – Oct (B)
ALL - INDIA	Dec (E) - Jan (M)	Mar (M) - Apr (E)	Sep (E) – Oct (B)

Harvesting Season of Principal Crops in Major Growing States

STATE	NIGER (KHARIF)	NIGER (LATE KHARIF)	GRAM (RABI)
Andhra Pradesh	Oct(L) - Nov(L)	Nov(B) - Dec(B)	Feb(M) - Mar(B)
Assam			Mar(B) - Mar(E)
Bihar	Oct(L) - Dec(M)	Nov(B) - Dec(B)	Mar(B) - Mar(E)
Chattisgarh	Oct(L) - Nov(L)		Feb(E) - Mar(E)
Gujarat	Oct(M) - Nov(L)	Nov(B) - Dec(B)	Feb(M) - Mar(M)
Haryana			Mar(M) - Apr(B)
Himachal Pradesh			
Jammu &Kashmir			
Karnataka	Oct(M) - Nov(M)	Nov(B) - Dec(B)	Jan(M) - Feb(E)
Kerala			
Madhya Pradesh	Oct(L) - Nov(L)	Nov(B) - Dec(B)	Feb(M) - Mar(E)
Maharashtra	Oct(M) - Dec(E)	Nov(B) - Dec(B)	Feb(M) - Mar(E)
Meghalaya			Dec(B) - Jan(E)
Manipur			Mar – Apr
Nagaland			
Odisha	Oct(L) - Nov(L)	Nov(B) - Dec(B)	
Punjab			Mar(M) - Apr(E)
Jharkhand	Oct(L) - Dec(M)	Nov(B) - Dec(B)	Mar(B) - Mar(M)
Rajasthan			Mar(M) - Apr(E)
Tamilnadu			
Tripura			
Uttar Pradesh			Mar(M) - Apr(E)
Uttarakhand			
West Bengal	Sep(E) - Oct(B)		Mar(B) - Mar(E)
ALL - INDIA	Sep(E) - Nov(M)	Nov(B) - Dec(B)	Dec(B) – Apr(E)

Harvesting Season of Principal Crops in Major Growing States

STATE	GROUNDNUT (KHARIF)	GROUNDNUT (RABI)	GROUNDNUT (SUMMER/ SPRING)
Andhra Pradesh	Oct (B) - Nov (E)	Feb (E) – May (B)	
Assam		Nov (B) – Dec (E)	
Bihar			
Chattisgarh	Sep (M) – Nov (B)		
Gujarat	Sep (B) - Nov (E)		Apr (B) – May (E)
Jharkhand	Oct (B) – Nov (E)		
Karnataka	Oct (B) - Nov (E)	Feb (E) –May (B)	Mar (B) – Jun (E)
Madhya Pradesh	Oct (B) - Nov (M)		
Maharashtra	Oct (B) - Nov (B)		Apr (B) – May (E)
Manipur	Oct (B) - Nov (E)		
Odisha	Oct (E) - Nov (B)	Jan(B)	Apr (B)- May (E)
Punjab	Oct (B) - Nov (E)		Jun (M)- Jun (E)
Puducherry	Sep (B) - Oct (E)	Mar (B) - Apr (E)	
Rajasthan	Oct (B) – Nov (E)		May (M)- May (E)
Tamilnadu	Sep (B) - Oct (B)	Jan (B)	Apr (B) – May (E)
Tripura			
Uttar Pradesh	Oct (B) - Nov(B)		
Uttarakhand			
West Bengal	Sep (E) – Oct (B)	Jan (E) - Mar (B)	May (B) – Jun (E)
ALL - INDIA	Oct - Nov	Feb - May	Apr - May

Harvesting Season of Principal Crops in Major Growing States

STATE	LINSEED (RABI)	SESAMUM (KHARIF)	SESAMUM (PRE-RABI)
Andhra Pradesh	Feb(L) - Mar(L)	Oct(M) -Nov(M)	Dec(E) - Jan(M)
Assam	Mar(E) - Apr(L)		
Bihar	Mar(B) - Apr(E)		
Chattisgarh	Mar(E) - Mar(L)		
Gujarat		Oct - Nov	
Haryana			
Himachal Pradesh	Apr(E) - May(M)		
Jammu & Kashmir	Apr(E) - May(M)		
Karnataka	Feb(E) - Mar(M)	Oct(M) - Nov(M)	
Kerala		Oct(M) - Nov(M)	
Madhya Pradesh	Mar(E) - Mar(L)	Oct(M) - Nov(M)	Dec(E) - Jan(M)
Maharashtra	Mar(E) - Mar(L)	Oct(M) - Nov(M)	Dec(E) - Jan(M)
Meghalaya			
Manipur			
Nagaland	Mar(E) - Apr(L)		
Odisha	Mar(E) - Mar(L)	Oct(M) - Nov(M)	
Punjab	Mar(E) - Apr(E)		
Jharkhand	Mar(E) - Apr(L)		
Rajasthan	Mar(B) - Mar(E)	Oct(M) - Nov(M)	
Tamilnadu		Oct – Nov	Dec(E) - Jan(M)
Telangana		Oct(L) - Nov(E)	
Tripura			
Uttar Pradesh	Mar(E) - Apr(L)	Oct(M) - Nov(M)	
Uttarakhand			
West Bengal	Mar(E) - Apr(M)		
ALL - INDIA	Feb(E) - May(M)	Sep – Nov	Dec(E) - Jan(M)

Harvesting Season of Principal Crops in Major Growing States

STATE	SESAMUM (RABI)	SESAMUM (SUMMER)	RAPESEED & MUSTARD SEED
Andhra Pradesh		Apr(M) - May(E)	Feb - Mar
Assam			Feb - Mar
Bihar			Feb - Mar
Chattisgarh			Mar
NCT Delhi			Feb - Mar
Gujarat		May – Jun	Feb - Mar
Haryana			Feb - Mar
Himachal Pradesh			Mar - Apr
Jammu & Kashmir			Mar - Apr
Karnataka			Feb
Kerala		Apr(M) - May(E)	
Madhya Pradesh			Feb - Mar
Maharashtra		Apr(M) - May(E)	Feb - Mar
Meghalaya			
Manipur			Mar
Nagaland			
Odisha	Feb(M) - Mar(E)	May(M) - Jun(M)	Feb - Mar
Punjab			Mar - Apr
Jharkhand			Mar
Rajasthan			Feb - Mar
Tamilnadu	Feb(M) - Mar(E)	May(M) - Jun(M)	
Telangana	Feb(L) - Mar(M)	Apr(E) - May(E)	
Tripura			
Uttar Pradesh		May(M) - Jun(M)	Feb - Mar
Uttarakhand			Mar
West Bengal		May(M) - Jun(M)	Feb - Mar
ALL - INDIA	Feb(M) - Mar(E)	May – Jun	Feb - Apr

Harvesting Season of Principal Crops in Major Growing States

STATE	SUNFLOWER (KHARIF)	SUNFLOWER (RABI)	SUNFLOWER (SUMMER/ SPRING)
Andhra Pradesh	Sep - Nov	Jan	
Assam			
Bihar		Apr - May	
Chattisgarh			
Gujarat			
Haryana			May
Himachal Pradesh			
Jammu & Kashmir			
Karnataka	Sep - Nov	Jan	Mar - Apr
Kerala			
Madhya Pradesh			
Maharashtra	Oct - Nov	Jan	
Meghalaya			
Manipur			
Nagaland			
Odisha			
Punjab			Apr - May
Jharkhand			
Rajasthan			
Tamilnadu	Sep – Nov	Jan	
Telangana	Sep – Nov		
Tripura			
Uttar Pradesh			
Uttarakhand			
West Bengal			Apr - May
ALL - INDIA	Sep – Nov	Jan	Apr - May

Harvesting Season of Principal Crops in Major Growing States

STATE	CASTORSEED (KHARIF)	MAIZE (KHARIF)	MAIZE (RABI)
Andhra Pradesh	Dec - Jan	Sep (M) – Oct (E)	Feb (E) – May (B)
Assam			
Bihar		Oct (M) – Nov (M)	Feb (B) – Mar (B)
Chattisgarh	Feb		
Gujarat	Jan - Feb	Sep (B) – Nov (B)	
Haryana	Jan - Feb	Oct (M) – Oct (E)	
Himachal Pradesh		Sep (M) – Oct (M)	
Jammu & Kashmir		Oct (B) – Nov (B)	
Karnataka	Nov - Dec	Sep (B) – Oct (E)	Feb (B) – Mar (E)
Kerala			
Madhya Pradesh		Aug (M) – Nov (E)	
Maharashtra	Dec - Jan	Oct (B) – Nov (E)	
Meghalaya			
Manipur			
Nagaland			
Odisha	Dec - Jan	Sep - Oct	
Punjab		Sep (B) – Oct (E)	May (B) – May (E)
Jharkhand			
Rajasthan	Jan - Feb	Oct (B) - Nov (E)	
Tamilnadu	Dec - Jan	Oct (M) - Oct (E)	Jan (B) – Jan (E)
Telangana	Dec – Jan		
Tripura			
Uttar Pradesh		Sep (M) - Oct (M)	May (B) - May (E)
Uttarakhand			
West Bengal		Sep (M) - Oct (M)	Mar (B) - Mar (E)
ALL - INDIA	Jan - Feb	Sep - Nov	Jan – May

Harvesting Season of Principal Crops in Major Growing States

STATE	COTTON (KHARIF)	JUTE (KHARIF)	POTATO
Andhra Pradesh	Dec(E) - Mar(M)		Apr - Jun
Assam			Dec - Jan
Bihar	Oct(M) - Nov(M)		Jan - Feb
Chattisgarh			Jan - Mar
Gujarat	Oct(B) - Apr(E)		Jan
Haryana	Oct(M) - Nov(M)		Feb - Mar
Himachal Pradesh			May - Jun & Aug -Nov
Jammu & Kashmir			Apr & Jul
Karnataka			Feb - Mar & Sep - Oct
Kerala	Dec(B) - Mar(E)	Oct(B) - Jan(E)	
Madhya Pradesh	Nov - Jan		Feb - Mar
Maharashtra	Nov - Jan		Mar - May
Meghalaya			Apr
Manipur		Aug - Sep	Dec - Jan
Mizoram			Sep
Nagaland			Feb
Odisha	Nov - Jan	Aug - Sep	Feb – Mar
Punjab	Oct(B) - Dec(E)		Dec – Jan
Puducherry			Apr & Jul
Jharkhand			Dec – Feb
Rajasthan	Nov(B) - Dec(E)		Jan – Feb
Sikkim			Jun – Aug
Tamilnadu	Feb(B) - Apr(E)		Round the year
Tripura		Aug - Sep	
Uttar Pradesh	Sep(B) - Nov(E)		Mar – Apr
Uttarakhand			Jul - Aug & Oct - Dec
West Bengal	Jun(B) - Aug(E)	Jul(B) - Aug(E)	Mar
D & N. Haveli			Jan
Daman & Diu			Jan
ALL - INDIA	Sep - Dec	Aug - Oct	Round the year

Harvesting Season of Principal Crops in Major Growing States

STATE	SUGARCANE (KHARIF)	SUGARCANE (RABI)
Andhra Pradesh		Dec(E) – May(M)
Assam		Dec(B) - Jan(E)
Bihar	Oct(B) - Dec(E)	Nov(B) - Feb(E)
Chattisgarh		
Gujarat		
Haryana		Dec(M) - Mar(E)
Himachal Pradesh		
Jammu & Kashmir		
Karnataka		Aug(B) - May(E)
Kerala		Dec(B) - Jan(E)
Madhya Pradesh	Oct(E) - Mar(E)	
Maharashtra	Oct(B) - Nov(E)	
Meghalaya		
Manipur		
Nagaland		
Odisha		Nov – Feb
Punjab		Nov(B) - Feb(E)
Jharkhand		
Rajasthan		Dec(B) - Mar(E)
Tamilnadu	May(B) - Aug(E)	Dec(B) - Jan(E)
Tripura		Dec – Mar
West Bengal	Oct(B) – Mar(E)	
ALL - INDIA	Aug(B) - Nov(E)	Oct(B) - Jan(E)

Source:-Agricultural Statistics at a Glance 2016
B: Beginning, E: Early, M: Middle, L: Late

APPENDIX - III

MINIMUM SUPPORT PRICES
(According to Crop Year)

Rs. Per Qtl.

**S.NO.**	**Commodity**	**Variety**	**2015-16**
	**KHARIF CROPS**		
1	PADDY	Common	1410
		Grade ‘A’	1450
2	JOWAR	Hybrid	1570
		Maldandi	1590
3	BAJRA		1275
4	MAIZE		1325
5	RAGI		1650
6	ARHAR (TUR)		4625
7	MOONG		4850
8	URAD		4625
9	COTTON	Medium Staple	3800
		Long Staple	4100
10	GROUNDNUT IN SHELL		4030
11	SUNFLOWER SEED		3800
12	SOYABEAN	Black	0
		Yellow	2600
13	SESAMUM		4700
14	NIGERSEED		3650
	**RABI CROPS**		
15	WHEAT		1525
16	BARLEY		1225
17	GRAM		3500**
18	MASUR (LENTIL)		3400**
19	RAPESEED/MUSTARD		3350
20	SAFFLOWER		3300
21	TORIA		3290
	**OTHER CROPS**		
22	COPRA	Milling	5550
	(Calendar Year)	Ball	5830
23	DE-HUSKED COCONUT (Calendar Year)		1500
24	JUTE		2700
25	SUGARCANE $		230

$ Fair and Remunerative Price

** Including Bonus of Rs.75 per quintal.
