# Supplementary material for: Averting wheat blast by implementing a ‘wheat holiday’: In search of alternative crops in West Bengal, India
Source: PLoS One. 2019 Feb 20;14(2):e0211410. doi: 10.1371/journal.pone.0211410 (PMC6382110; doi:10.1371/journal.pone.0211410)
Supplement: S4 File — (PDF) [file pone.0211410.s004.pdf]

Pocket Book of
AGRICULTURAL STATISTICS
2017

Government of India

Ministry of Agriculture & Farmers Welfare

Department of Agriculture, Cooperation & Farmers Welfare

Directorate of Economics & Statistics

New Delhi

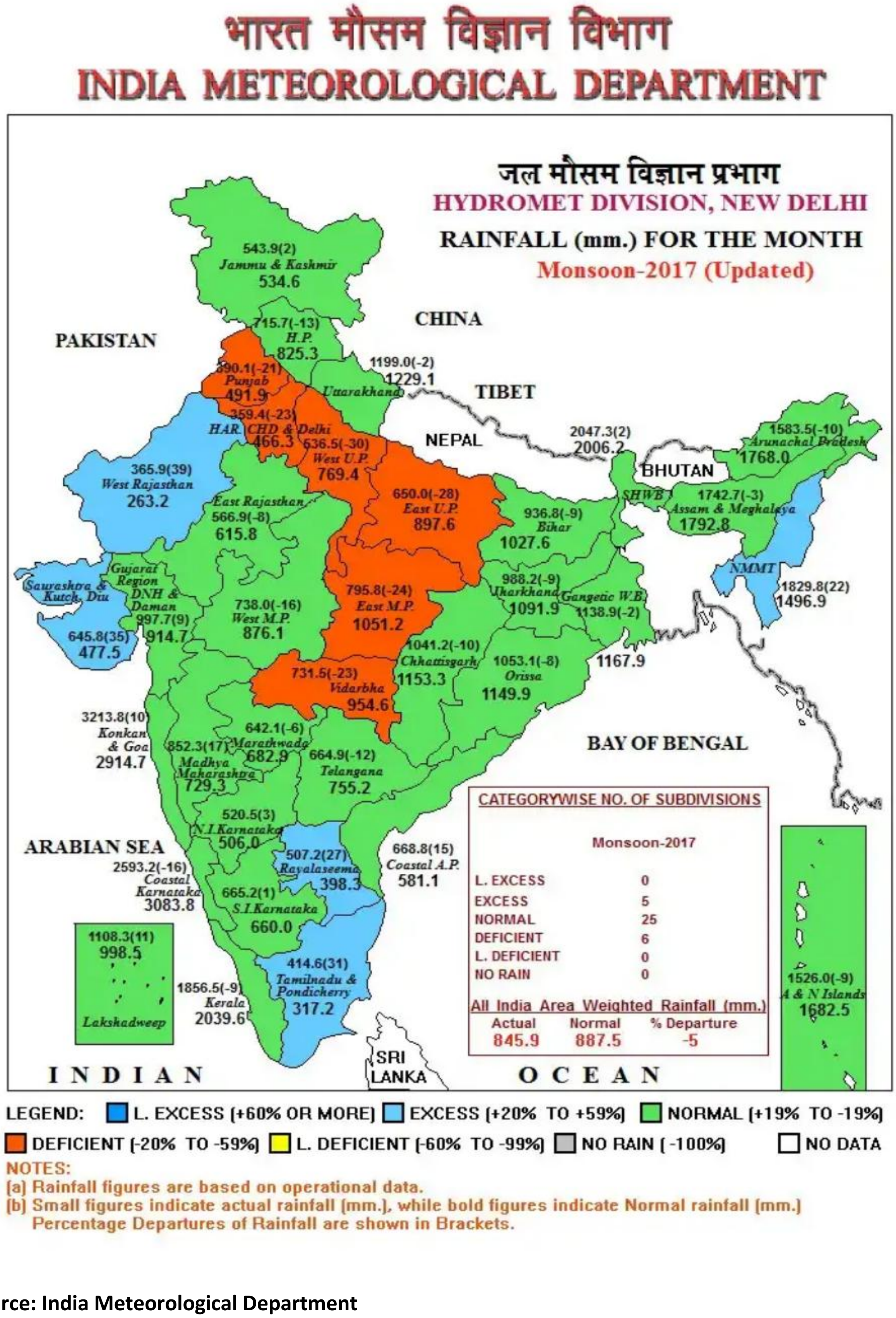

Source: India Meteorological Department

K. L. PRASAD
Senior Economic & Statistical Adviser

सत्यमेव जयते

अर्थ एवं सांख्यिकी निदेशालय
कृषि, सहकारिता एवं किसान कल्याण विभाग
कृषि एवं किसान कल्याण मंत्रालय
भारत सरकार
कृषि भवन, नई दिल्ली- 110001
Directorate of Economics & Statistics
Department of Agriculture, Cooperation & Farmers Welfare
Ministry of Agriculture & Farmers Welfare
Government of India
Krishi Bhawan, New Delhi-110001
Tele. : 011-23384036 Telefax : 23382016
E-mail : office.esa-agri@nic.in
kl.prasad@nic.in

PREFACE

The 'Pocket Book of Agricultural Statistics' begun in 2013 as a quick reference guide for all the essential data relating to agricultural sector. The Pocket Book has become a regular publication of the Directorate.

This year's Pocket Book takes forward our continuous endeavor towards improving ready access to key data on agriculture and allied sector. The Pocket Book includes updated information on key indicators of agriculture and allied sector. In addition, this has been enriched with the status on various new initiatives of the Government, viz., Soil Health Card Scheme, Pradhan Mantri Fasal Bima Yojana (PMFBY), micro irrigation, etc. A copy of this publication has also been uploaded on our website http://eands.dacnet.nic.in/ for information of public at large.

I am grateful to Shri P.C. Bodh, Adviser and his team for their efforts in bringing out this publication.

Suggestions for improvement are most welcome.

(K.L. Prasad)
Sr. Economic & Statistical Adviser

Date: 28-06-2018

New Delhi.

CONTENTS

S.No.	Particulars	Page No.
**ACRONYMS**		**viii**
**STATISTICAL TABLES**		
**1**	**Socio-Economic Indicators**	**1**
1.1	Selected Economic and Social Indicators	2
1.2	Gross Value Added(GVA) at basic prices by Economic Activity (at 2011-12 prices)	3
1.3	Gross Value Added (GVA) at basic prices by Economic Activity (at current prices)	3
1.4	Percentage share of Gross Value Added(GVA) at 2011-12 Prices	4
1.5	Percentage Growth of Gross Value Added(GVA) at 2011-12 Prices	5
**2**	**Outlays, Expenditure & Capital Formation**	**6**
2.1	Year-wise Expenditure by Departments under Ministry of Agriculture & Farmers Welfare	7
2.2	Public Sector Outlays/ Expenditure	8
2.3	Outlays / Expenditure under Agriculture and Allied Activities	8
2.4	Plan-wise share of Public Sector Outlays and Expenditure under Agriculture and Allied Activities	9
2.5	Comparison of States Outlay and Expenditure for Eleventh and Twelfth Plan	10
2.6	Expenditure on Agricultural Research and Education	11
2.7	Share (%) of Gross Capital Formation (GCF) to Gross Value Added (GVA) in Agriculture & Allied Sectors (Based on 2011-12 Series)	12
2.8	Public and Private Investment in Agriculture and Allied Sectors in Total GDP at Market Prices (2011-12 series)	12
**3**	**Labour Force, Poverty and Unemployment**	**13**
3.1	Population and Agricultural Workers	14
3.2	Population & Labour Force Projections	15
3.3	Population Below Poverty Line (Tendulkar Methodology)	16
3.4	All India Rural & Urban Unemployment Rates (2011-12)	17
**4**	**Agricultural Land-Use Statistics**	**18**
4.1	Agricultural Land by use in India	19
4.2	Percentage Distribution of Gross Cropped Area	20
4.3	Changes in Share of Area under Major Crops	21
4.4	Number and Area of Operational Holdings by Size Group	22
4.5	South West Monsoon Rainfall (1 Jun- 30 Sep.)	23
**5**	**Area, Production and Yield of Principal Crops**	**24**
5.1	Foodgrains: Area, Production and Yield during 2015-16 and 2016-17 in major Producing States alongwith coverage under Irrigation	25
5.2 (a)	Area under Major Crops from 1950-51 onwards (in million hectares)	26
5.2 (b)	Production of Major Agricultural Crops	27

5.3	Target and Achievement of Production of Major Crops	28
5.4	Three Largest Producing States of Important Crops during 2016-17	29
5.5	Production of Oilseeds/Oils and Net Domestic Availability of Edible Oils	31
5.6	All India Crop-wise Yield	32
5.7	All India Crop-wise Irrigated Area	33
5.8	State-wise Coverage of Irrigated Area under Major Crops during 2014-15	34
**6**	**Horticulture Statistics**	**35**
6.1	Area and Production of Horticulture Crops – All India	36
6.2	All-India Area, Production and Yield of Potato and Onion	38
6.3	All-India Area, Production and Yield of Banana	38
6.4	Foodgrain and Horticulture Production in India	39
6.5	Value of Output from Horticulture Crops vis-à-vis All Agriculture Crops (at 2011-12 prices)	40
**7**	**Livestock Statistics**	**41**
7.1	Livestock Population in India	42
7.2	All India Production of Milk, Eggs and Wool	43
7.3	Fish Production in India	43
**8**	**Agricultural Inputs, Costs & Prices**	**44**
8.1	Production and Use of Agricultural Inputs in India	45
8.2	Crop-wise Requirement and Availability of Certified/ Quality Seeds	46
8.3	Crop-wise Requirement and Availability of Certified/ Quality of Hybrid Seeds	47
8.4	Consumption, Production and Import of Fertilisers in terms of Nutrients (N, P & K)	48
8.5	Zone-wise Consumption of Fertilisers in terms of Nutrients (N, P & K)	49
8.6 (a)	State-wise Status of Soil Health Card Scheme Cycle-I as on 20.02.2018	50
8.6 (b)	State-wise Status of Soil Health Card Scheme Cycle-II as on 20.02.2018	51
8.7	Consumption of Electricity for Agricultural Purposes	52
8.8	Year-wise Sales of Tractors and Power tillers	53
8.9	Flow of Institutional Credit to Agriculture Sector	54
8.10	State-wise Agricultural Loan disbursed in 2016-17	55
8.11	State-wise and Agency-wise KCCs - Cumulative cards issued and amount Outstanding as on 31 March 2017	56
8.12	State-wise coverage under Pradhan Mantri Fasal Bima Yojana (PMFBY) - Cumulative upto Rabi 2016-17	57
8.13	State-wise coverage under Restructured Weather Based Crop Insurance Scheme (RWBCIS) – Cumulative upto Rabi 2016-17	58
8.14	Crop-wise area insured under all Insurance Schemes	59
8.15	State wise Crop Area Insured under all Insurance Schemes	60
8.16	State-wise Area Covered under Micro Irrigation as on 31.03.2017	61
8.17	State wise Cold Storage capacity as on 31.03.2017	62
8.18	Cost Estimate of Principal Crops 2014-15	63

8.19	All India projected Cost of Production (A2+FL) of Major Crops in India	67
8.20	All India Annual Average Daily Wage Rate	68
**9**	**Prices & Procurement**	**69**
9.1	Minimum Support Prices of various Agricultural Commodities	70
9.2	State-wise Procurement of Rice and Wheat in Major Rice and Wheat Producing States	71
9.3	Trends in Wholesale Price Index of Commercial Crops (Base: 2004-05=100)	72
**10**	**Per-Capita Availability & Expenditure**	**74**
10.1	Per Capita Net Availability of Foodgrains (Per Annum) in India	75
10.2	Per Capita Availability of Certain Important Articles of Consumption	75
10.3	Trends in Consumer Expenditure since 2004-05 (percentage composition)	76
10.4	Index of Terms of Trade between Agriculture and Non-Agricultural sectors	77
10.5	Index of Terms of Trade between Farmers and Non-Farmers	78
**11**	**Key Indicators of Situation of Agricultural Households in India**	**79**
11.1	Estimated Number of Rural Households, Agricultural Households and Indebted Agricultural Households	80
11.2	Indebtedness of Agricultural Households (all-India) in Different Size Classes of Land Possessed	81
11.3	Incidence of Indebtedness in Major States	82
11.4	Incidence of Indebtedness based on size of land possessed	83
11.5	Average monthly expenses and receipts for crop Production per agricultural household for major States during Jul' 2012 - Jun'2013	85
11.6	Average monthly expenses and receipts for farming of animals per agricultural household for major States during Jul' 2012 - Jun'2013	86
**12**	**Agricultural Projections**	**87**
12.1	Projected Demand and Supply of Food Commodities for the 12th Plan period	88
12.2	Average Annual Growth Rate of Production of selected Food Commodities in India: Actual Production Trends in 2005–15 and Projections by various agencies	88
12.3	Production, Consumption & Year-end Stocks Projections of Major Crops	89
12.4	Production Projections of Potatoes and Onion	89
**13**	**Imports & Exports of Agricultural Products**	**92**
13.1	India's Imports and Exports of Agricultural Commodities	93
13.2	India's Imports of Agricultural Commodities	94
13.3	India's Exports of Agricultural Commodities	95
13.4	Leading Exporters and Importers of Agricultural Products in 2016	96
**14**	**International Comparisons**	**97**
14.1	India's Position in World Agriculture in 2015	98
14.2	Area, Production and Yield of Principal Crops in various countries in 2015	99
14.3	Major Macro-Economic Indicators of India, G-20 and the World in 2016	101

14.4	Major Macro Economic, Land-Use and Social Indicators of BRICS Economies in 2015	102
14.5	Major Economic and Land-Use Indicators: India and China	103
14.6	Inflation (Consumer Prices) in Major Economies (Annual %)	104
**15**	**Global Agricultural Emissions**	**106**
**CHARTS**		
1 (a)	Sector-wise Share in Gross Value Added(GVA) at 2011-12 Prices	4
1 (b)	Sector-wise Growth Rate of GVA (2011-12 Prices)	5
4 (a)	Agriculture Land by Use in India	19
4 (b)	Changes in Gross Cropped and Gross Irrigated Areas	19
4 (c)	Distribution of Gross Cropped Area	20
4 (d)	Changes in share of Area under Major Crops	21
6 (a)	Trends in Foodgrain and Horticulture Production	39
8 (a)	Trends in Consumption, Production and Imports of Fertilizers in India	48
8 (b)	Trends in Average Daily Wage Rate of Male & Female Workers in Agricultural Operations	68
9 (a)	Month-wise Trend in Food Inflation of WPI and CPI	73
9 (b)	Trends in Inflation (WPI) of Food Products, Food Articles and all Commodities	73
11 (a)	Distribution of Agricultural Households over Social Groups	84
11 (b)	Distribution of Agricultural Households by principal source of income	84
12 (a)	Projected Production of Rice, Wheat and Coarse Grains in India	90
12 (b)	Projected Production of Vegetables and Fruits in India	90
12 (c)	Projected Market Support Prices in India and World Prices for Rice and Wheat	90
12 (d)	Projected Per capita Consumption of Cereals in India	91
12 (e)	Projected Calorie and Protein Consumption in India	91
13 (a)	Trends in Agricultural Imports and Exports and share out of Total National Imports/ Exports	93
14 (a)	Projected Wheat production in China and India	105
14 (b)	Projected Nutri Cereals production in U.S, China and India	105
14 (c)	Projected Oilseeds production in China, India and Canada	105
15 (a)	All GHG Emission from Agriculture sector by major Continents in 2014 (CO2 equivalent)	107
15 (b)	Source-wise Total GHG Emissions from Agriculture Sector in 2014	107
**APPENDICES**		**108**
A.1	Explanatory Notes	109
A.2	Conversion Factors between Important Primary and Secondary Agricultural Commodities	110
A.3	General Seed Rate of Sowing for Important Field Crops	112
A.4	Rainfall Position in India	114
A.5	Major Economic Groups/ Institutions	115

ACRONYMS

AAY	Antyodaya Anna Yojana
AE	Advance Estimate
AGDP	Gross Domestic Product from Agriculture
APL	Above Poverty Line
BE	Budget Estimates
BPL	Below Poverty Line
CACP	Commission for Agricultural Costs and Prices
CBB	Commercial Bank Branch
CCE	Crop Cutting Experiments
CCI	Cotton Corporation of India
CDSBO	Crude Degumed Soyabean Oil
CES	Crop Estimation Surveys
CFA	Central Financial Agency
CIF	Cost, Insurance & Freight
CIP	Central Issue Price
CPI-IW	Consumer Price Index for Industrial Workers
CWWG	Crop Weather Watch Group
DAC&FW	Department of Agriculture, Cooperation & Farmers Welfare
DAP	Di Ammonium Phosphate
DES	Directorate of Economics and Statistics
E	Estimated
EARAS	Establishment of an Agency for Reporting of Agricultural Statistics
FAO	Food and Agriculture Organisation of United Nations
FAQ	Fair Average Quality
F2	Black Soil
F-414/H-777/J-34	Cotton variety (of Medium Staple Length)
H-4/H-6	Cotton variety (of Long Staple Length)
FCI	Food Corporation of India

FDI	Foreign Direct Investment
FFB	Fresh Fruit Bunch
FOD	Field Operations Division of NSSO
FRL	Full Reservoir Level
FYM	Farm Yard Manure
GCA	Gross Cropped Area
GCES	General Crop Estimation Survey
GCF	Gross Capital Formation
GDP	Gross Domestic Product
GNP	Gross National Product
GSDP	Gross State Domestic Product
GVA	Gross Value Added
Ha	Hectares
HYV	High Yielding Varieties
I	Irrigated
IAS	Improvement of Agricultural Statistics
ICAR	Indian Council of Agricultural Research
ICOR	Incremental Capital Output Ratio
ICS	Improvement of Crop Statistics
K	Potash
KCC	Kisan Credit Card
Kgs	Kilograms
KMPH	Kilometers Per Hour
KWH	Kilo Watt Hour
LS	Light Soil
LPCPD	Litres Per Capita Per Day
MSCCGMF	Maharashtra State Co-operative Cotton Growers Marketing Federation
MIS	Market Intervention Scheme
MSP	Minimum Support Price
MSR	Marketed Surplus Ratio

N	Nitrogen
NA	Not Available/Not Announced
NC	Not Collected
NCAER	National Council of Applied Economic Research
NQ	Not Quoted
NAFED	National Agricultural Co-operative Marketing Federation of India Limited
NCAER	National Council of Applied Economic Research
NDDB	National Dairy Development Board
Neg.	Negligible
NNP	Net National Product
N,P&K	Nitrogen, Phosphate and Potash
NR	Not Recommended/ Not Reported
NS	New Series Estimates
NSA	Net Sown Area
NSSO	National Sample Survey Organisation
OECD	Organisation of Economic Cooperation and Development
OPDP	Oil Palm Development Programme
P	Provisional/ Phosphate
PACS	Primary Agricultural Credit Society
PDS	Public Distribution System
PLDB	Primary Land Development Bank
PMKSY	Pradhan Mantri Krishi Sinchayee Yojana
PMFBY	Pradhan Mantri Fasal Bima Yojana
PSE	Producers Support Estimate
PSEs	Public Sector Enterprises
PSS	Price Support Scheme
QE	Quick Estimate
R	Revised
R&D	Research & Development
R&M	Rapeseed & Mustard

RMC	Regional Ministers Conference
RE	Revised Estimates
RRBB	Regional Rural Bank Branch
SCB	State Cooperative Bank
SDP	State Domestic Product
SASA	State Agricultural Statistical Authority
SHC	Soil Health Card
SLDB	State Land Development Bank
SRS	Sample Registration Scheme
SSP	Single Super Phosphate
T	Target
T E	Triennium Ending
TD-5	Tossa Deshi variety of Jute
TPDS	Targeted Public Distribution System
TRQ	Tariff Rate Quota
TRRM	Tropical Rains Measuring Mission
TT	Tentative Targets
UI	Un-irrigated
UT	Union Territory
WPI	Wholesale Price Index
WTO	World Trade Organisation
Y	Yield (Index Number of Yield) per hectare

SOCIO-ECONOMIC INDICATORS

Table: 1.1 Selected Economic and Social Indicators

Indicators	1950-51	1960-61	1990-91	2000-01	2010-11	2011-12	2012-13	2013-14	2014-15	2015-16	2016-17 (PE)
(1)	(2)	(3)	(4)	(5)	(6)	(7)	(8)	(9)	(10)	(11)	(12)
**ECONOMIC INDICATORS**											
GVA [a] (at current prices in ₹ crore)	10036	17049	531814	2000743	7248860	8106946	9202692	10363153	11504279	12566646[#]	13841591[@]
GVA[a] (at constant prices in ₹ crore)	279618	410279	1347889	2348481	4918533	8106946	8546275	9063649	9712133	10503348[#]	11247629[@]
Per capita Net National Income at factor cost at constant prices (₹)	7513	9482	15996	22491	39270	63462	65538	68572	72805	77826[#]	82229[@]
Gross Domestic Capital Formation as percentage of GDP (at current market prices)	9.3	14.3	26.0	24.3	36.5	39.0	38.7	33.8	33.5	32.3	30.6
Gross domestic savings as percentage of GDP (at current market prices)	9.5	11.6	22.9	23.7	33.7	34.6	33.9	32.1	32.2	31.3	30.0
Index of agricultural production [base: Triennium ending 1981-82] for the data given till 2000-01 and base i.e. ending 2007-08 from 2009-10 onwards	46.2	68.8	148.4	165.7	121.1	124.5	124.2	129.8	124	120.8	130.6
05=100)[b]	7.9	15.6	91.6	162.6	165.5	170.3	103.3	106.7	111.0	114.7	120.0
Wholesale Price Index average [c]	6.8	7.9	73.7	155.7	143.3	156.1	106.9	112.5	113.9	109.7	111.6
Consumer Price Index for Industrial workers[d]	17	21.0	193.0	444.0	180.0	195.0	215.0	236.0	251.0	265.0	276.0
**OUTPUT**											
(a) Foodgrains [millon tonnes]	50.8	82.0	176.4	196.8	244.5	259.3	257.1	265.0	252.0	251.6	275.1
(b) Coal and lignite [millon tonnes]	32.3	55.2	225.5	332.6	570.4	582.3	602.9	610.0	657.4	683	704.4(P)
(c) Crude oil [millon tonnes]	0.3	0.5	33.0	32.4	37.7	38.1	37.9	37.8	37.4	36.9	36.0
(d) Electricity generated [utilities only] [billon KWH]	5.0	17.0	264.0	500.0	844.8	922.5	964.5	1026.6	1105.1	1167.6	1236.4
Plan outlay (₹ crore)	2601	1117	58369	185737	826268	936292	971951	1281022 (RE)	420882[f]	582707(RE)[f]	706248.4(BE)
FOREIGN TRADE											
(i) Exports (₹ Crore)	606	642	32553	203571	1136964	1465959	1634319	1905011	1896348	1716378	1849429
(US $ million)	1269	1346	18143	44076	251136	305964	300401	314405	310338	262290	275852
(ii) Imports (₹ Crore)	608	1122	43198	230873	1683467	2345463	2669162	2715434	2737087	2490298	2577666
(US $ million)	1273	2353	24075	49975	369769	489319	490737	450200	448033	381007	384356
Foreign exchange reserves [g] [excluding gold,SDRs and Reverse Tranche Position at IMF]; (₹ Crore)	911	186	4388	184482	1224883	1330511	1412631	1660914	1985458	2219061	2244939
(US $ million)	1914	390	2236	39554	274330	260069	259726	276359	295947	336104	346319
**SOCIAL INDICATORS**											
Population (Million)[h]	361	439.2	846.4	1028.7	1186.0	1220.0	1235.0	1251.0	1267.0	1283.0	1299.0
Birth Rate (per 1000)[i]	39.9	41.7	29.5	25.4	21.8[k]	21.6[k]	21.4[k]	21.0[k]	20.8	20.4	NA
Death Rate (per 1000) [i]	27.4	22.8	9.8	8.4	7.1[k]	7.0[k]	7.0[k]	6.7[k]	6.5	6.4	NA
Life Expectancy at Birth (in Years) [j]	32.1	41.3	58.7	62.5	67.0	67.5	67.9	68.3	NA	NA	NA
(a) Male	32.5	41.9	58.6	61.6	65.4	65.8	66.4	66.9	NA	NA	NA
(b) Female	31.7	40.6	59.0	63.3	68.8	69.3	69.6	70.0	NA	NA	NA
Education: Literacy Rate (%) [l]	18.3	28.3	52.2	64.8	73.0	NA	NA	NA	NA	NA	NA
(a) Male	27.2	40.4	64.1	75.3	82.1	NA	NA	NA	NA	NA	NA
(b) Female	8.9	15.4	39.3	53.7	65.5	NA	NA	NA	NA	NA	NA

BE: Budget Estimate, RE: Revised Estimate, AE: Advance Estimate, PE: Provisional Estimate NA: Not Available # : 2nd Revised Estimates (New Series)
^The figures before 2012-13 are measured at factor cost. The figures from 2012-13 onwards are measured at basic pric @ : 1st Revised Estimates (New
a: GVA at factor cost estimates prior to 2011-12 are based on 2004-05 series. Estimates from Year 2011-12 onwards (with Base 2011-12) are available at Basic Prices only.
b: The Index of Industrial Production has been revised since 2012-13 on base 2011-12. The
figures from 2012-13 onwards are based on current series (2011-12). and earlier data are
c: The figures from 2012-13 onwards are based on current series 2011-12=100 and earlier data are based on old base years.
d: The figures from 2009-10 onwards are based on current series 2001=100 and earlier data are based on old base years.
e: Final Estimates.
f: Outlays are only of the Centre as Outlays of State & UTs are Not Available from 2014-15 onwards.
g: As on end-March.
h: Relates to mid-financial year (as on October 1) based on population figures of C.S.O.
i: For calendar year. Figure shown against 1990-91 is for calendar year 1991 and so on. Source: Office of R.G.I.
j: Data for 1950-51, 1960-61, 1970-71 and 1980-81 relate to the decades 1941-50, 1951-60, 1961-70 and 1971-80 respectively, centered at midpoints of the decade, i.e., 1946, 1956, 1966 and 1976. The estimates for 1990-91 refer to the period 1988-92 and so on.
Estimates for 2010-11 refers to abridged life table of period 2008-12, 2011-12 refers to 2009-13, 2012-13 refers to 2010-14 and 2013-14 refers to 2011-15.
k: Estimates from Sample Registration System and SRS Statistical Report, Office of RGI.
l: Data for 1950-51, 1960-61, 1970-71, 1980-81, 1990-91 and 2000-01 are as per Census of India 1951,1961, 1971, 1981, 1991 and 2001. The figures for 1951 1961 and 1971 relate to population aged 5 years and above and those for 1981, 1991, 2001 and 2011 to population aged 7 years and above. All India literacy rates exclude Assam for 1981 and J&K for 1991.
Source : Ministry of Finance, Department of Commerce, CSO and RBI.

Table 1.2: Gross Value Added(GVA) at basic prices by Economic Activity (at 2011-12 prices)

(₹ crore)

S.No.	Industry	2011-12	2012-13	2013-14	2014-15	2015-16*	2016-17#	2017-18 @
(1)	(2)	(3)	(4)	(5)	(6)	(7)	(8)	(9)
1.	**Agriculture, forestry and fishin[illegible]**	**1501947**	**1524288**	**1609198**	**1605715**	**1615216**	**1716746**	**1774573**
	crops	982151	983809	1037060	998425	966345	1033008	-
	livestock	327334	344375	363558	390449	421369	448964	-
	forestry and logging	124436	124743	132093	134609	137022	138779	-
	fishing and aquaculture	68027	71362	76487	82232	90480	95996	-
II	**Industry**	**2635023**	**2721167**	**2824188**	**3021899**	**3317881**	**3542820**	**3739212**
	Mining and quarrying	261035	262609	263107	288685	328453	371066	381965
	Manufacturing	1409986	1486873	1560709	1683938	1898790	2048711	2166267
	Electricity, gas, water supply & other utility services	186668	191635	199601	214047	224198	244934	262496
	Construction	777335	780050	800771	835229	866440	878110	928484
**III**	**Services**	**3969975**	**4300819**	**4630263**	**5084519**	**5570251**	**5988063**	**6462370**
	**TOTAL GVA at basic prices**	**8106946**	**8546275**	**9063649**	**9712133**	**10503348**	**11247629**	**11976155**

Source: Central Statistics Office

* : 2nd Revised Estimates (New Series) # : 1st Revised Estimates (New Series) @ : Provisional Estimates (New Series)

Table 1.3: Gross Value Added (GVA) at basic prices by Economic Activity (at current prices)

(₹ crore)

S.No.	Industry	2011-12	2012-13	2013-14	2014-15	2015-16*	2016-17#	2017-18 @
(1)	(2)	(3)	(4)	(5)	(6)	(7)	(8)	(9)
1.	**Agriculture, forestry and fishin[illegible]**	**1501947**	**1675107**	**1926372**	**2093612**	**2225368**	**2484005**	**2594729**
	crops	982151	1088814	1248776	1292874	1327997	1530137	-
	livestock	327334	368823	422733	510411	584070	639912	-
	forestry and logging	124436	137558	156674	173760	182247	180465	-
	fishing and aquaculture	68027	79911	98190	116567	131053	133492	-
**II**	**Industry**	**2635024**	**2923394**	**3190871**	**3448189**	**3746625**	**4054112**	**4411640**
	Mining and quarrying	261035	285842	295794	308476	301230	332947	374689
	Manufacturing	1409986	1572837	1713452	1878369	2116119	2329220	2530311
	Electricity, gas, water supply & other utility services	186668	215350	260155	282258	336978	363482	387694
	Construction	777335	849365	921470	979086	992298	1028463	1118946
**III**	**Services**	**3969975**	**4604192**	**5245909**	**5962478**	**6594653**	**7303474**	**8176002**
	**Total GVA at basic prices**	**8106946**	**9202692**	**10363153**	**11504279**	**12566646**	**13841591**	**15182371**

Source: Central Statistics Office

* : 2nd Revised Estimates (New Series) # : 1st Revised Estimates (New Series) @ : Provisional Estimates (New Series)

Table 1.4: Percentage share of Gross Value Added(GVA) at Current Prices

S.No.	Industry	2011-12	2012-13	2013-14	2014-15	2015-16*	2016-17#	2017-18@
(1)	(2)	(3)	(4)	(5)	(6)	(7)	(8)	(9)
**1**	**Agriculture,Forestry & Fishing**	**18.5**	**18.2**	**18.6**	**18.2**	**17.7**	**17.9**	**17.1**
1.1	crops	12.1	11.8	12.1	11.2	10.6	11.1	
1.2	livestock	4.0	4.0	4.1	4.4	4.6	4.6	
1.3	forestry and logging	1.5	1.5	1.5	1.5	1.5	1.3	
1.4	fishing and aquaculture	0.8	0.9	0.9	1.0	1.0	1.0	
**2**	**Industry**	**32.5**	**31.8**	**30.8**	**30.0**	**29.8**	**29.3**	**29.1**
2.1	mining & quarrying	3.2	3.1	2.9	2.7	2.4	2.4	2.5
2.2	manufacturing	17.4	17.1	16.5	16.3	16.8	16.8	16.7
2.3	electricity, gas, water supply & other utility services	2.3	2.3	2.5	2.5	2.7	2.6	2.6
2.4	construction	9.6	9.2	8.9	8.5	7.9	7.4	7.4
**3**	**Services**	**49.0**	**50.0**	**50.6**	**51.8**	**52.5**	**52.8**	**53.9**
**4**	**Gross Value Added at basic prices**	**100.0**	**100.0**	**100.0**	**100.0**	**100.0**	**100.0**	**100.0**

* : 2nd Revised Estimates (New Series) # : 1st Revised Estimates (New Series) @ : Provisional Estimates (New Series)

Source: Central Statistics Office

Chart 1 (a): Sector-wise Share in Gross Value Added(GVA) at Current Prices

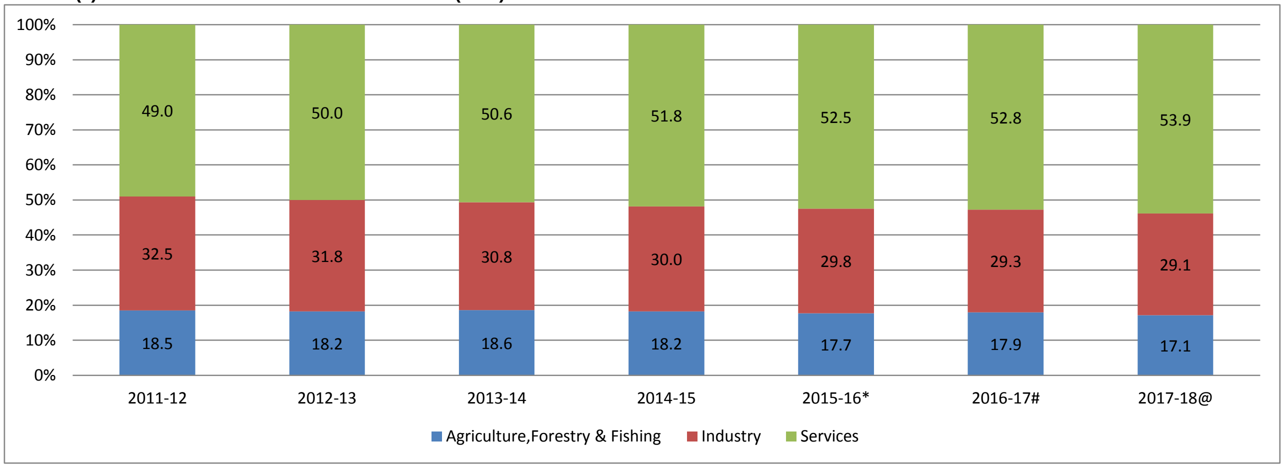

* : 2nd Revised Estimates (New Series) # : 1st Revised Estimates (New Series) @ : Provisional Estimates (New Series)

Source: Central Statistics Office.

Table 1.5: Percentage Growth of Gross Value Added(GVA) at 2011-12 Prices

S.No.	Industry	2012-13	2013-14	2014-15	2015-16*	2016-17#	2017-18@
(1)	(2)	(3)	(4)	(5)	(6)	(7)	(8)
**1**	**Agriculture,forestry & fishing**	**1.5**	**5.6**	**-0.2**	**0.6**	**6.3**	**3.4**
1.1	crops	0.2	5.4	-3.7	-3.2	6.9	
1.2	livestock	5.2	5.6	7.4	7.9	6.5	
1.3	forestry and logging	0.2	5.9	1.9	1.8	1.3	
1.4	fishing and aquaculture	4.9	7.2	7.5	10.0	6.1	
**2**	**Industry**	**3.3**	**3.8**	**7.0**	**9.8**	**6.8**	**5.5**
2.1	mining & quarrying	0.6	0.2	9.7	13.8	13.0	2.9
2.2	manufacturing	5.5	5.0	7.9	12.8	7.9	5.7
2.3	electricity, gas, water supply & other utility services	2.7	4.2	7.2	4.7	9.2	7.2
2.4	construction	0.3	2.7	4.3	3.7	1.3	5.7
**3**	**Services**	**8.3**	**7.7**	**9.8**	**9.6**	**7.5**	**7.9**
**4**	**Gross Value Added at basic prices**	**5.4**	**6.1**	**7.2**	**8.1**	**7.1**	**6.5**

* : 2nd Revised Estimates (New Series) # : 1st Revised Estimates (New Series) @ : Provisional Estimates (New Series)

Source: Central Statistics Office

Chart 1 (b): Sector-wise Growth Rate of GVA at 2011-12 Prices

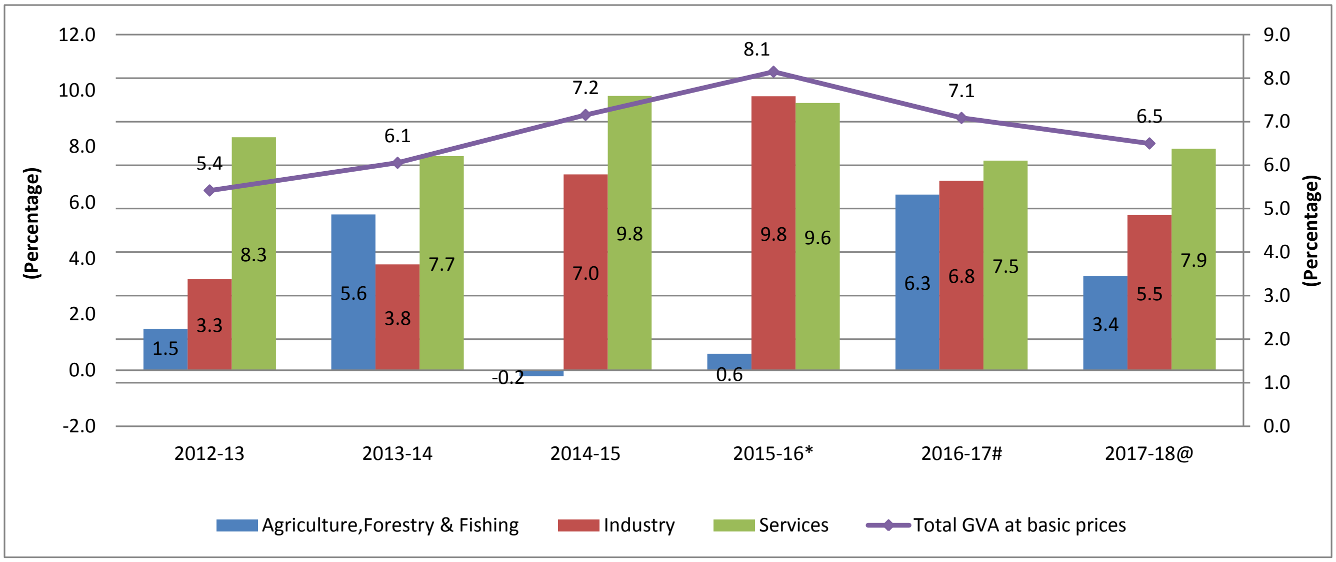

* : 2nd Revised Estimates (New Series) # : 1st Revised Estimates (New Series) @ : Provisional Estimates (New Series)

Source: Central Statistics Office.

OUTLAYS, EXPENDITURE & CAPITAL FORMATION

Table 2.1: Year-wise Expenditure by Departments under Ministry of Agriculture & Farmers Welfare

(₹ crore)

Year	DAC&FW			DARE			DAHD&F		
	Budget Estimate	Revised Estimate	Actual Expenditure	Budget Estimate	Revised Estimate	Actual Expenditure	Budget Estimate	Revised Estimate	Actual Expenditure
(1)	(2)	(3)	(4)	(5)	(6)	(7)	(8)	(9)	(10)
1991-92	1041.35	1016.93	957.86	190.00	180.51	173.07	-	-	-
2001-02	1985.00	1985.00	1792.92	684.00	550.00	683.69	300.00	240.00	239.68
2004-05	2650.00	-	2657.42	1000.00	900.00	881.81	500.00	-	566.22
2009-10	11307.07	10965.23	10870.15	1833.37	1821.32	1821.26	1100.00	930.00	873.38
2010-11	15042.00	17254.00	17052.59	2307.50	2307.50	2529.29	1300.00	1257.00	1104.68
2011-12	17122.87	16515.05	16354.70	2808.54	2858.54	2831.60	1600.00	1356.52	1243.12
2012-13	20208.00	17867.32	17730.72	3232.00	2520.00	2519.09	1910.00	1800.00	1736.71
2013-14	21609.00	19000.00	18721.70	3415.00	2600.00	2599.97	2025.00	1800.00	1748.66
2014-15	22309.00	19530.00	19191.61	3715.00	2500.00	2456.82	2174.00	1800.00	1738.12
2015-16	16646.35	15500.00	15028.00	3691.00	3000.00	2988.61	1491.14	1491.14	1410.34
2016-17	20400.00	29411.32	26639.66	3700.00	3166.58	2989.65	1600.00	1748.02	1743.12
2017-18	41855.00	41105.00	28908.08*	6800.00	NA	3138.06**	2371.00	2166.74	1511.20***

Source: Ministry of Agriculture & Farmers Welfare

* As on 31.12.2017 ** As on 31.10.17 *** As on 31.12.2017

DAC&FW: D/o Agriculture, Cooperation & Farmers Welfare, DARE: D/o Agricultural Research and Education

DAHD&F: D/o Animal Husbandry, Dairying & Fisheries

Table 2.2: Public Sector Outlays/ Expenditure

(₹ Crore)

Sector	2011-12		12th Plan*	2012-13		2013-14		2014-15		2015-16		2016-17
	BE	AE	Outlays	BE	AE	BE	RE	BE(##)	RE(##)	BE(##)	RE(##)	BE(##)
(1)	(2)	(3)	(4)	(5)	(6)	(7)	(8)	(9)	(10)	(11)	(12)	('13)
Agriculture and Allied Activities	46255	46037	363273	56669	52521	64098	61356	11531	10199	11657	10942	19394
Rural Development	75821	37599	457464	72248	66194	77307	73255	3082	1877	3110	3005	2623
Special Area Programmes	12413	0	80370	15342	11289	18392	18095	0	0	0	0	0
Irrigation & Flood Control	64001	506	422012	72169	54394	78211	71253	1797	896	772	1105	1024
Energy	208436	121855	1438466	208711	179438	221095	238019	166275	154878	167342	171519	205877
Industry & Minerals	56400	36235	377302	71491	46786	64602	51428	40209	39397	43113	45512	49371
Transport	171344	107532	1204172	183015	145002	196628	175543	116202	106242	193417	178502	229874
Communications	20256	6586	80984	15411	6289	12380	9333	13009	13027	12032	13451	13806
Science, Technology & Environment	22160	11735	167350	24220	18304	26874	22664	18792	14821	19023	17965	20926
General Economic Services	35179	19697	305612	51525	37079	63022	47660	26318	17303	20333	38597	46686
Social Services	356713	145352	2664843	419267	340266	476980	443357	79411	64284	81024	83555	100420
General Services	28226	5305	107959	66040	14390	71347	69060	7906	3887	26558	18554	16247
**Total**	**1097204**	**538439**	**7669807**	**1256108**	**971951**	**1370936**	**1281023**	**484532**	**426811**	**578382**	**582707**	**706248**

Source: Economic Survey and Budget Documents, Ministry of Finance.

Note: Figures include Outlays and Expenditure by Centre, States and Union Territories.

For Centre only as figures for States & UTs are not available. * 12th Plan Projections.

Table 2.3: Outlays / Expenditure under Agriculture and Allied Activities

(₹ Crore)

Sector	2011-12		2012-13		2013-14		2014-15		2015-16	
	BE	AE	BE	RE	BE	AE	BE	RE	BE	RE
(1)	(2)	(3)	(4)	(5)	(6)	(7)	(8)	(9)	(10)	(11)
Crop Husbandry	7891	8066	9034	8660	9876	9185	4432	3857	4339	4794
Horticulture	NA	NA	NA	NA	NA	NA	NA	NA	NA	NA
Soil & Water Conservation	15	15	16	15	19	16	18	17	18	17
Animal Husbandry	950	724	1063	888	975	925	172	151	130	156
Dairy Development	223	196	353	524	525	502	411	365	482	488
Fisheries	270	304	299	297	317	316	423	302	411	392
Forestry & Wild Life	786	914	907	806	1041	884	379	318	275	284
Plantation	415	595	446	501	458	468	370	347	360	360
Food Storage & Warehousing	644	703	787	764	801	1222	727	1169	1090	639
Agriculture, Research & Edn.	2492	2573	2898	2461	3113	2451	3354	2211	3321	2700
Agricultural Financial Inst.	NA	NA	NA	NA	NA	NA	NA	NA	NA	NA
Cooperation	222	124	125	104	121	112	125	99	112	125
Other Agricultural Programmes	836	1980	1764	2008	1535	1707	1120	1364	1120	986
**Total**	**14744**	**16194**	**17692**	**17030**	**18781**	**17788**	**11531**	**10199**	**11657**	**10942**

Source: 1. Budget Documents, Ministry of Finance.

2. Plan Documents, Planning Commission. For State Outlays, State Plan Division, Planning Commission.

Notes : Totals may not tally due to rounding off.

Figures are for Centre only as separate figures for States & UTs are not available sub-head wise.

Table 2.4: Plan-wise share of Public Sector Outlays and Expenditure under Agriculture and Allied Activities

(₹ Crore)

Five Year Plan / Annual Plan	Agriculture and Allied Activities		Total Plan Outlay		% share of Agriculture and Allied Activities	
	Plan Outlays	Actual Expenditure	Plan Outlays	Actual Expenditure	Plan Outlays	Actual Expenditure
(1)	(2)	(3)	(4)	(5)	(6)	(7)
**Ninth Plan (1997-2002)**	**42462**	**37239 $**	**859200**	**941041 $**	**4.9**	**4.0**
**Tenth Plan (2002-07)**	**58933**	**60702 $**	**1525639**	**1618460 $**	**3.9**	**3.8**
**Eleventh Plan (2007-12)**	**136381**	**163105 $**	**3644718**	**3676936 $**	**3.7**	**4.4**
Annual Plan (2007-08)	17971	20083	558765	475012	3.2	4.2
Annual Plan (2008-09)	27270	27117	684288	628161	4.0	4.3
Annual Plan (2009-10)	28772	29498	794616	717035	3.6	4.1
Annual Plan (2010-11)	36983	40370	929725	826268	4.0	4.9
Annual Plan (2011-12)	46255	46037	1097204	1030461	4.2	4.5
**Twelfth Plan (2012-17)***	**363273**	**NA**	**7669807**	**NA**	**4.7**	**N.A**
Annual Plan (2012-13)	56669	52521	1251715	971951	4.5	5.4
Annual Plan (2013-14)	64098	61356(RE)	1370936	1281022(RE)	4.7	4.8
Annual Plan (2014-15)#	11531	9795	484532	420882	2.4	2.4
Annual Plan (2015-16)#	11657	10942(RE)	578382	582707(RE)	2.0	1.9
Annual Plan (2016-17)#	19394	NA	706248	NA	NA	NA

Source: Economic Survey and Budget Documents, Ministry of Finance

Notes:

1. Figures include Outlays and Expenditure by Centre, States and Union Territories.
2. Actual Central Expenditure figures are including IEBR from 2009-10 onwards

* 12th Plan projections (At current prices).

\# For Centre only as figures for States & UTs are not yet available.

$ Indicates 9th, 10th and 11th Plan Realization.

Table 2.5: Comparison of States Outlay and Expenditure for Eleventh and Twelfth Plan

(₹ in crore at current prices)

State	Eleventh Plan Outlay		Eleventh Plan Expenditure		Twelfth Plan Outlay		
	Agriculture and Allied Sector	% of Total Plan	Agriculture and Allied Sector	% of Total Plan	Agriculture and Allied Sector	% of Total Plan	Increase in Twelfth Plan over Eleventh Plan Expenditure (%)
(1)	(2)	(3)	(4)	(5)	(6)	(7)	(8)
Andhra Pradesh	3487.44	2.4	9510.46	6.0	17138	5	80
Arunachal Pradesh	752	9.5	617.71	5.7	1114	5.3	80
Assam	877.86	2.1	2335.56	7.8	3272	5.9	40
Bihar	3672.73	4.8	4805.33	6.3	15613	6	225
Chhattisgarh	4613	8.6	5637	12.7	8284	6.9	47
Goa	211.76	2.5	325.39	3.6	1046	3.9	221
Gujarat	9092.94	0.7	8879.8	6.9	19712	7.8	122
Haryana	1638.82	4.7	2733.02	5.7	6288	5.4	130
Himachal Pradesh	1470.08	10.7	1642.82	12.1	2174	9.7	32
Jammu & Kashmir	1818.21	7	892.98	3.5	2843	9.7	218
Jharkhand	3130.53	0.8	2319.85	5.9	4157	3.8	79
Karnataka	8426.85	8.3	10484.4	7.7	19824	8.9	89
Kerala	2649.11	7.8	2931.54	7.6	8831	11.5	201
Madhya Pradesh	3408.18	4.8	6057.09	7.3	17076	8.5	182
Maharashtra	9507.64	5.9	10636.4	7.3	19325	7.03	82
Manipur	386.55	4.7	234.04	3.2	643	3.1	175
Meghalaya	735.52	8	845.2	9.8	2114	10.7	150
Mizoram	536.31	9.6	387.86	7.1	346	2.8	-
Odisha	1230.29	3.8	3580.37	8.2	8387	7.4	134
Nagaland	434.31	8.3	725.08	11.3	1795	13.8	148
Punjab	1309.13	4.5	1410.77	4	1524	2.9	8
Rajasthan	2919.07	4.1	5990.67	6.2	7255	5.6	21
Sikkim	260.43	6.9	228.27	6.4	469	4.1	106
Tamil Nadu	7831.57	9.2	8170.01	8.8	20680	10	153
Tripura	798.51	9	858.79	11.3	980	6.8	14
Uttar Pradesh	19146.37	10.6	14164.8	7.8	24354	8.5	72
Uttarakhand	2478.5	8.4	2079.25	10	2673	5.9	29
West Bengal	1846.5	2.9	3339.26	5.1	8583	5.5	157
**Total States**	**94670.21**	**3.6**	**111823.72**	**7.2**	**226500**	**7.1**	**103**

Source: 12th Plan Document, Planning Commission

Table 2.6: Expenditure on Agricultural Research and Education

(₹ Crore at 2006-07 prices)

State/ Centre		Tenth Plan	2007-08	2008-09	2009-10	2010-11	2011-12	Eleventh Plan
(1)	(2)	(3)	(4)	(5)	(6)	(7)	(8)	(9)
State	Plan	4151	694	965	1070	1289	1382	5401
	Non-Plan	6477	1464	1315	1497	1755	1599	7629
	Total	10629	2158	2279	2567	3044	2981	13030
Centre	Plan	4977	1210	1418	1402	1909	1998	7938
	Non-Plan	4125	852	1040	1235	2168	1512	6808
	Total	9102	2063	2458	2636	4077	3510	14745
RKVY	Plan	-	55	197	63	100	160	576
**Centre and States**	**Plan**	**9128**	**1961**	**2580**	**2534**	**3298**	**3540**	**13914**
	**Non-Plan**	**10603**	**2316**	**2355**	**2732**	**3923**	**3111**	**14437**
	**Total**	**19732**	**4277**	**4935**	**5266**	**7221**	**6652**	**28351**
GDP Agriculture and Allied (2006-07 prices)		3340648	764890	765601	773565	827969	850812	3982837
Research/Education as % GDP Agriculture		0.59%	0.55%	0.61%	0.67%	0.86%	0.76%	0.70%

Source: 12th Plan Document, Planning Commission

Table 2.7: Share (%) of Gross Capital Formation (GCF) to Gross Value Added (GVA) in Agriculture & Allied Sectors (Based on 2011-12 Series)

(Percentage)

Year	At 2011-12 Prices			At Current Prices		
	Public	Private	Total	Public	Private	Total
(1)	(2)	(3)	(4)	(5)	(6)	(7)
2011-12	2.4	15.9	18.2	2.4	15.9	18.3
2012-13	2.4	14.1	16.5	2.4	14.0	16.3
2013-14	2.1	15.6	17.7	2.1	15.1	17.2
2014-15	2.3	15.0	17.3	2.3	14.1	16.3
2015-16	2.8	13.6	16.4	2.8	12.6	15.4
2016-17	2.7	12.8	15.5	2.6	11.2	13.8

Source : Central Statistics Office

Table 2.8: Public and Private Investment in Agriculture and Allied Sectors in Total GDP at Market Prices (Based on 2011-12 Series)

(₹ Crore)

Year	Public Investment	Private Investment	Total	GDP at market price	Share (%) in Total		
					Public	Private	Total
(1)	(2)	(3)	(4)	(5)	(6)	(7)	(8)
2011-12	35696	238175	273871	8736329	0.4	2.7	3.1
2012-13	36019	215075	251094	9213017	0.4	2.4	2.7
2013-14	33925	250499	284424	9801370	0.3	2.6	2.9
2014-15	36714	240700	277414	10536984	0.3	2.3	2.6
2015-16	44957	220081	265038	11381002	0.4	1.9	2.3
2016-17	45981	219371	265352	12196006	0.4	1.8	2.2

Source : Central Statistics Office(CSO)

LABOUR FORCE, POVERTY AND UNEMPLOYMENT

Table 3.1: Population and Agricultural Workers

(Million)

Year	Total Population	Average Annual Exponential Growth Rate (%)	Rural Population	Total Workers	Agricultural Workers		
					Cultivators	Agricultural Labourers	Total
(1)	(2)	(3)	(4)	(5)	(6)	(7)	(8)
1951	361.1	1.25	298.6	139.5	69.9	27.3	97.2
			(82.7)		(71.9)	(28.1)	(69.7)
1961	439.2	1.96	360.3	188.7	99.6	31.5	131.1
			(82.0)		(76.0)	(24.0)	(69.5)
1971	548.2	2.20	439.0	180.4	78.2	47.5	125.7
			(80.1)		(62.2)	(37.8)	(69.7)
1981	683.3	2.22	525.6	244.6	92.5	55.5	148.0
			(76.9)		(62.5)	(37.5)	(60.5)
1991	846.4	2.16	630.6	314.1	110.7	74.6	185.3
			(74.5)		(59.7)	(40.3)	(59.0)
2001	1028.7	1.97	742.6	402.2	127.3	106.8	234.1
			(72.2)		(54.4)	(45.6)	(58.2)
2011	1210.9	1.50	833.7	481.9	118.8	144.3	263.1
			(68.9)		(45.1)	(54.9)	(54.6)

Source: Registrar General of India.

1. For 2001, figures include estimated figures for those of the three sub-divisions viz. Mao Maram, Paomata and Purul of Senapati district of Manipur as census results of 2001 Census in these three sub-divisions were cancelled due to technical and administrative reasons.
2. The 1991 Census could not be held owing to disturbed conditions prevailing in Jammu & Kashmir. Hence the population figures for 1991 of Jammu & Kashmir have been worked out by 'interpolation'. The data on workers in Col. 5-7 exclude J&K.
3. The 1981 census could not be held in Assam. The figures for 1981 for Assam have been worked out by interpolation. The data on workers in Col. 5-7 exclude Assam.
4. Figures within parentheses in Col.-4 are percentages to the Total Population.
5. Figures within parentheses in Col.-5 and 6 are percentages to Col.-7.
6. Figures within parentheses in Col.-8 is percentage share of Agricultural Workers in Total Workers.

Table 3.2: Population & Labour Force Projections

(Percent per annum)

Year	2011	2012	2013	2014	2015	2016	2017
(1)	(2)	(3)	(4)	(5)	(5)	(6)	(7)
Total Population (0+) (in million)	1210.2	1227.1	1244.0	1260.6	1277.1	1293.5	1309.7
15 & above Population (%)	70.2	71.0	71.8	72.6	73.4	74.2	75.0
Population (15 & above) (in million)	849.6	871.3	893.2	915.2	937.4	959.8	982.2
LFPR for 15 and above age group (%)	56.3	55.4	54.5	53.7	52.8	52.0	51.2
Labour Force (15 & above) (in million)	477.9	482.7	487.2	491.5	495.4	499.1	502.4

Source: 12th Plan Document, Planning Commission

Table 3.3: Population Below Poverty Line (Tendulkar Methodology)

(Percentage)

States/UTs	2009-10			2011-12		
	Rural	Urban	Total	Rural	Urban	Total
(1)	(2)	(3)	(4)	(5)	(6)	(7)
Andhra Pradesh	22.80	17.70	21.10	10.96	5.81	9.20
Arunachal Pradesh	26.20	24.90	25.90	38.93	20.33	34.67
Assam	39.90	26.10	37.90	33.89	20.49	31.98
Bihar	55.30	39.40	53.50	34.06	31.23	33.74
Chhattisgarh	56.10	23.80	48.70	44.61	24.75	39.93
Delhi	7.70	14.40	14.20	12.92	9.84	9.91
Goa	11.50	6.90	8.70	6.81	4.09	5.09
Gujarat	26.70	17.90	23.00	21.54	10.14	16.63
Haryana	18.60	23.00	20.10	11.64	10.28	11.16
Himachal Pradesh	9.10	12.60	9.50	8.48	4.33	8.06
Jammu & Kashmir	8.10	12.80	9.40	11.54	7.20	10.35
Jharkhand	41.60	31.10	39.10	40.84	24.83	36.96
Karnataka	26.10	19.60	23.60	24.53	15.25	20.91
Kerala	12.00	12.10	12.00	9.14	4.97	7.05
Madhya Pradesh	42.00	22.90	36.70	35.74	21.00	31.65
Maharashtra	29.50	18.30	24.50	24.22	9.12	17.35
Manipur	47.40	46.40	47.10	38.80	32.59	36.89
Meghalaya	15.30	24.10	17.10	12.53	9.26	11.87
Mizoram	31.10	11.50	21.10	35.43	6.36	20.40
Nagaland	19.30	25.00	20.90	19.93	16.48	18.88
Odisha	39.20	25.90	37.00	35.69	17.29	32.59
Punjab	14.60	18.10	15.90	7.66	9.24	8.26
Rajasthan	26.40	19.90	24.80	16.05	10.69	14.71
Sikkim	15.50	5.00	13.10	9.85	3.66	8.19
Tamil Nadu	21.20	12.80	17.10	15.83	6.54	11.28
Tripura	19.80	10.00	17.40	16.53	7.42	14.05
Uttar Pradesh	39.40	31.70	37.70	30.40	26.06	29.43
Uttarakhand	14.90	25.20	18.00	11.62	10.48	11.26
West Bengal	28.80	22.00	26.70	22.52	14.66	19.98
A&N Islands	0.40	0.30	0.40	1.57	0.00	1.00
Chandigarh	10.30	9.20	9.20	1.64	22.31	21.81
Dadra & Nagar Haveli	55.90	17.70	39.10	62.59	15.38	39.31
Damn & Diu	34.20	33.00	33.30	0.00	12.62	9.86
Lakshadweep	22.20	1.70	6.80	0.00	3.44	2.77
Puducherry	0.20	1.60	1.20	17.06	6.30	9.69
**All-India**	**33.80**	**20.90**	**29.80**	**25.70**	**13.70**	**21.92**

Source: Perspective Planning Division, Planning Commission.

1. Poverty estimates are based on methodology suggested by the expert group on Methodology for estimation of poverty, chaired by Dr. Suresh.D. Tendulkar

2. Number of persons below poverty line in 2009-10 are estimated based on Population as on 1st March 2010 (interpolated between 2001 and 2011 population Census)

3. Number of persons below poverty line in 2011-12 are estimated based on Population as on 1st March 2012 (2011 Census population extrapolated)

4. Poverty line of Tamil Nadu is used for Andaman and Nicobar Island.

5. Urban Poverty Line of Punjab is used for both rural and urban areas of Chandigarh.

6. Poverty Line of Maharashtra is used for Dadra & Nagar Haveli.

7. Poverty Line of Goa is used for Daman & Diu

8. Poverty Line of Kerala is used for Lakshadweep.

Table 3.4: All India Rural & Urban Unemployment Rates (2011-12)

(Percentage)

Estimates	Rural			Urban			Rural+ Urban		
	Male	Female	M+F	Male	Female	M+F	Male	Female	M+F
(1)	(2)	(3)	(4)	(5)	(6)	(7)	(8)	(9)	(10)
Usual Principal Status	2.1	3.0	2.3	3.2	6.4	3.8	2.5	3.8	2.8
Usual Status	1.8	1.6	1.7	3.0	5.2	3.5	2.2	2.4	2.2
Current Weekly Status	3.3	3.5	3.4	3.8	6.9	4.4	3.5	4.3	3.7
Current Daily Status	5.5	6.2	5.7	4.9	7.9	5.4	5.3	6.7	5.6

Source: NSSO Report (68th Round)- Employment and Unemployment Situation in India, 2011-12

AGRICULTURAL LAND-USE STATISTICS

Table 4.1: Agricultural Land by use in India

(Million Hectares)

S.No.	Classification	1950-51	1990-91	2000-01	2009-10 (P)	2010-11 (P)	2011-12 (P)	2012-13 (P)	2013-14 (P)	2014-15 (P)
(1)	(2)	(3)	(4)	(5)	(6)	(7)	(8)	(9)	(10)	(11)
**I.**	**Geographical Area**	**328.73**	**328.73**	**328.73**	**328.73**	**328.73**	**328.73**	**328.73**	**328.73**	**328.73**
**II.**	**Reporting Area for Land Utilisation Statistics**	**284.32**	**304.86**	**305.19**	**307.41**	**307.48**	**307.39**	**307.49**	**307.80**	**307.82**
	1. Forest	40.48	67.81	69.84	71.56	71.59	71.60	71.57	71.83	71.79
	2. Not Available for Cultivation (A+B)	47.52	40.48	41.23	43.33	43.58	43.53	43.58	43.86	43.88
	(A) Area Under Non-agricultural Uses	9.36	21.09	23.75	26.16	26.40	26.31	26.50	26.91	26.88
	(B) Barren & Un-culturable Land	38.16	19.39	17.48	17.18	17.18	17.22	17.07	16.95	17.00
	3. Other Uncultivated land excluding Fallow	49.45	30.22	27.74	26.50	26.15	26.11	26.08	25.83	25.83
	(A) Permanent Pasture & other Grazing Land	6.68	11.40	10.66	10.34	10.30	10.31	10.26	10.26	10.26
	(B) Land under Miscellaneous Tree Crops &	19.83	3.82	3.44	3.21	3.20	3.16	3.18	3.19	3.10
	(C) Culturable Waste Land	22.94	15.00	13.63	12.95	12.65	12.64	12.64	12.39	12.47
	4. Fallow Lands (A+B)	28.12	23.37	25.04	26.85	24.60	25.18	26.32	24.85	26.18
	(A) Fallow Lands other than Current Fallows	17.45	9.66	10.27	10.84	10.32	10.67	11.04	10.69	11.09
	(B) Current Fallows	10.68	13.70	14.78	16.01	14.28	14.51	15.29	14.15	15.09
	5. Net Area Sown (6-7)	118.75	143.00	141.34	139.17	141.56	140.98	139.94	141.43	140.13
	6. Total Cropped Area (Gross Cropped Area)	131.89	185.74	185.34	189.19	197.68	195.80	194.25	200.95	198.36
	7. Area Sown more than once	13.15	42.74	44.00	50.02	56.12	54.82	54.31	59.52	58.23
	8. Cropping Intensity*	111.07	129.89	131.13	135.94	139.64	138.88	138.81	142.09	141.55
	**Net Irrigated Area**	**20.85**	**48.02**	**55.20**	**61.94**	**63.67**	**65.71**	**66.29**	**68.12**	**68.38**
	**Gross Irrigated Area**	**22.56**	**63.20**	**76.19**	**85.09**	**88.94**	**91.79**	**92.25**	**95.77**	**96.46**

* Cropping Intensity is percentage of Gross Cropped Area to Net Area Sown.

(P) Provisional

Notes:

1. In 2009-10 there is significant decline in Total Cropped Area and Net Area Sown due to decline in net area sown in the states of Andhra Pradesh, Bihar, Jharkhand, Rajasthan, Tamil Nadu, Uttar Pradesh and West Bengal. This was mainly due to deficient rainfall.

Source: Land-Use Statistics 2014-15, Directorate of Economics & Statistics, DAC&FW

Chart 4 (a): Agriculture Land by Use in India

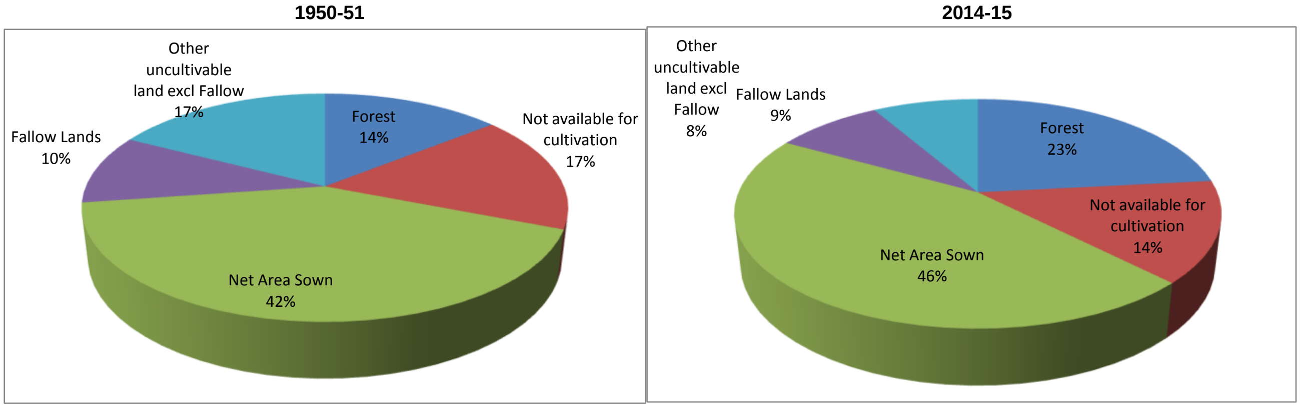

Source: Directorate of Economics & Statistics, DAC&FW

Chart 4 (b): Changes in Gross Cropped and Gross Irrigated Areas

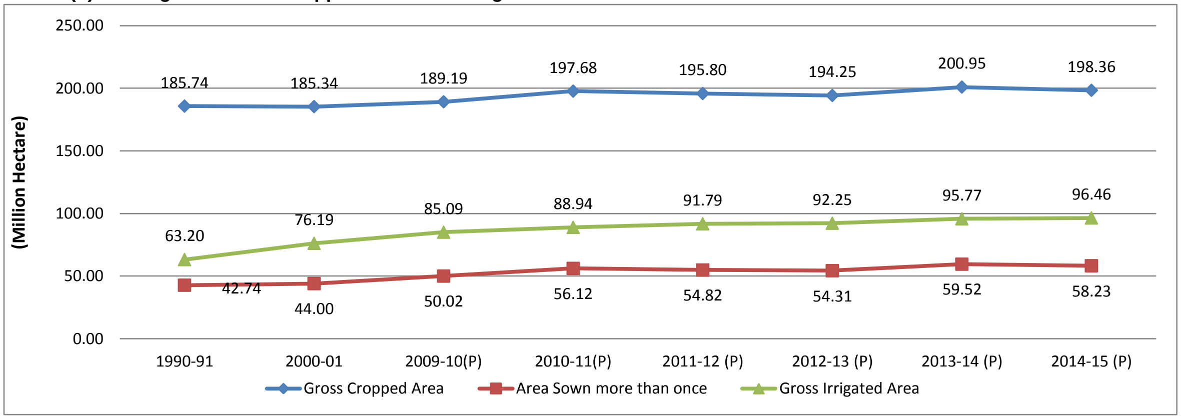

Source: Directorate of Economics & Statistics, DAC&FW

Table 4.2: Percentage Distribution of Gross Cropped Area

Crops	1950-51	1960-61	1970-71	1980-81	1990-91	2000-01	2010-11 (P)	2011-12 (P)	2012-13 (P)	2013-14 (P)	2014-15 (P)
(1)	(2)	(3)	(4)	(5)	(6)	(7)	(8)	(9)	(10)	(11)	(12)
Rice	23.5	22.3	22.5	23.3	23.0	24.2	21.9	22.3	22.0	22.1	22.3
Jowar	11.8	12.1	10.2	9.5	7.6	5.3	3.7	3.2	3.2	2.9	3.1
Bajra	7.4	7.5	8.1	6.8	5.8	5.4	4.9	4.5	3.9	4.0	4.0
Maize	2.5	2.9	3.5	3.5	3.2	3.7	4.3	4.4	4.4	4.5	4.4
Ragi/Marua	1.7	1.6	1.5	1.5	1.2	1.0	0.6	0.6	0.6	0.6	0.6
Wheat	7.6	8.5	11.0	12.9	12.9	13.9	15.2	15.4	15.7	15.6	16.2
Barley	2.4	2.1	1.5	1.0	0.5	0.4	0.4	0.3	0.4	0.3	0.4
Other Cereals & Millets	4.2	3.3	3.0	2.3	1.3	0.8	0.4	0.4	0.4	0.4	0.3
Total Cereals & Millets	61.1	60.2	61.4	60.8	55.5	54.7	51.4	**51.1**	**50.7**	**50.4**	**51.3**
Gram	5.9	6.1	4.7	3.8	4.0	2.9	4.4	4.0	4.1	4.7	3.9
Tur or Arhar	1.7	1.6	1.6	1.7	1.9	2.0	2.1	1.9	1.8	1.8	1.7
Other pulses (Excl. Gram & Tur or Arhar)	8.0	7.8	7.6	7.7	7.4	6.7	6.3	6.1	5.4	5.4	5.4
Total Pulses	15.6	15.5	13.9	13.2	13.4	11.5	12.8	**12.0**	**11.3**	**11.8**	**10.9**
**Total Foodgrains**	**76.7**	**75.6**	**75.3**	**73.9**	**68.9**	**66.2**	**64.3**	**63.1**	**62.0**	**62.3**	**62.3**
Sugar-cane	1.3	1.6	1.6	1.7	2.1	2.5	2.6	2.8	2.8	2.7	2.8
Total Condi-ments and Spices	0.9	1.0	1.1	1.2	1.3	1.5	1.7	1.9	1.7	1.6	1.7
Total Fruits & Vege-tables	1.7	1.7	2.2	2.9	3.6	4.4	4.8	4.9	4.9	4.9	5.0
Other Food Crops	0.5	0.8	0.1	0.1	0.1	0.1	0.1	0.1	0.1	0.1	0.1
**Total Food Crops**	**81.2**	**80.8**	**80.3**	**79.7**	**75.9**	**74.7**	**73.4**	**72.7**	**71.5**	**71.7**	**72.0**
Ground-nut	3.3	4.2	4.6	3.9	4.5	3.6	2.9	2.7	2.7	2.7	2.6
Castor- seed	0.4	0.3	0.3	0.3	0.5	0.6	0.5	0.7	0.6	0.5	0.5
Sesamum	1.5	1.0	1.1	1.0	1.3	0.9	1.1	1.0	0.9	0.8	0.9
Rape-seed & Mustard	0.8	0.7	0.9	1.3	2.8	2.3	2.8	2.8	3.1	3.0	2.7
Lin-seed	0.9	0.8	0.7	0.7	0.5	0.3	0.1	0.1	0.1	0.1	0.1
Coco-nut	0.5	0.5	0.6	0.6	0.8	1.0	0.9	1.0	1.0	0.9	1.0
Other Oilseeds	0.8	0.7	0.7	1.2	3.2	4.6	6.3	6.1	6.5	6.9	6.5
Total Oilseeds	8.3	8.4	8.9	9.1	13.5	13.3	14.6	14.3	14.9	15.0	14.3
Cotton	4.3	5.0	4.7	4.5	4.1	4.6	5.5	6.2	6.1	5.9	6.4
Jute	0.4	0.4	0.5	0.5	0.4	0.5	0.4	0.4	0.4	0.4	0.4
Other Fibres	0.3	0.3	0.3	0.3	0.2	0.2	0.1	0.1	0.1	0.1	0.1
Total Fibres	5.1	5.7	5.5	5.3	4.7	5.2	6.0	6.7	6.6	6.4	6.8
Indigo	0.0	0.0	0.0	0.0	0.0	0.0	0.0	0.0	0.0	0.0	0.0
Opium	0.0	0.0	0.0	0.0	0.0	0.0	0.0	0.0	0.0	0.0	0.0
Tobacco	0.3	0.3	0.3	0.3	0.2	0.2	0.2	0.2	0.2	0.2	0.2
Tea	0.2	0.2	0.2	0.2	0.2	0.3	0.3	0.3	0.3	0.3	0.3
Coffee	0.1	0.1	0.1	0.1	0.2	0.2	0.2	0.2	0.2	0.2	0.2
Fodder Crops	3.3	3.8	4.2	4.7	4.5	5.0	3.9	4.0	4.8	4.9	4.6
Other Non-Food Crops	1.5	0.8	0.4	0.5	0.8	1.1	0.7	1.6	1.4	1.4	1.5
**Total non-food crops**	**18.8**	**19.2**	**19.6**	**20.2**	**24.1**	**25.3**	**26.6**	**27.3**	**28.5**	**28.3**	**28.0**
**Total area under crops**	**100**	**100**	**100**	**100**	**100**	**100**	**100.0**	**100.0**	**100.0**	**100.0**	**100.0**

(P) Provisional

Source: Land-Use Statistics 2014-15, Directorate of Economics & Statistics, DAC&FW

Chart 4 (c): Distribution of Gross Cropped Area

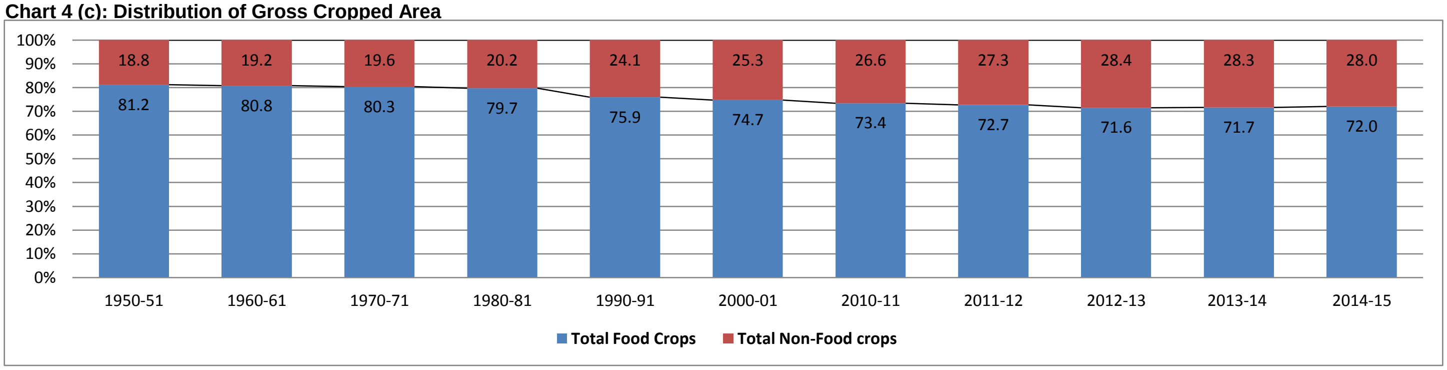

Source: Directorate of Economics & Statistics, DAC&FW

Table 4.3: Changes in the Share of Area under Major Crops

(Percentage)

Year	Rice	Wheat	Nutri Cereals	Pulses	Food grains	Oilseeds	Sugarcane	Cotton	Others	All Crops
(1)	(2)	(3)	(4)	(5)	(6)	(7)	(8)	(9)	(10)	(11)
2007-08	23.31	14.88	15.12	12.54	**65.85**	14.17	2.68	5.00	12.30	100.00
2008-09	24.17	14.73	14.57	11.72	**65.19**	14.63	2.34	4.99	12.85	100.00
2009-10	22.59	15.33	14.91	12.54	**65.38**	13.99	2.25	5.46	12.93	100.00
2010-11	21.85	14.82	14.44	13.46	**64.56**	13.88	2.49	5.73	13.34	100.00
2011-12	22.48	15.25	13.50	12.49	**63.72**	13.44	2.57	6.22	14.05	100.00
2012-13	22.13	15.53	12.82	12.04	**62.51**	13.71	2.59	6.20	15.00	100.00
2013-14	22.07	15.24	12.61	12.61	**62.54**	14.03	2.50	5.98	14.95	100.00
2014-15	22.22	15.90	12.72	11.90	**62.80**	12.93	2.56	6.48	15.23	100.00
2015-16	22.90	15.45	12.39	12.65	**62.60**	13.25	2.50	6.24	15.40	100.00
2016-17	22.55	15.98	12.93	15.38	**66.84**	13.68	2.29	5.66	11.52	100.00

Note: (1) Area estimates are based on the data provided by State Statistical Authroities (SASAs) whch is further cross checked and validated with estimates provided by other agencies, viz., MNCFC and CWWG.

(2). Others include Jute & Mesta and Horticulture Crops

(3) Data for Foodgrains, Oilseed and Commercial Crops are as per fourth Advance Estimates for 2016-17.

(4) Data for Horticulture crops are as per third Advance Estimates for 2016-17

Source: Directorate of Economics & Statistics, DAC&FW

Chart 4 (d): Changes in the share of Area under Major Crops

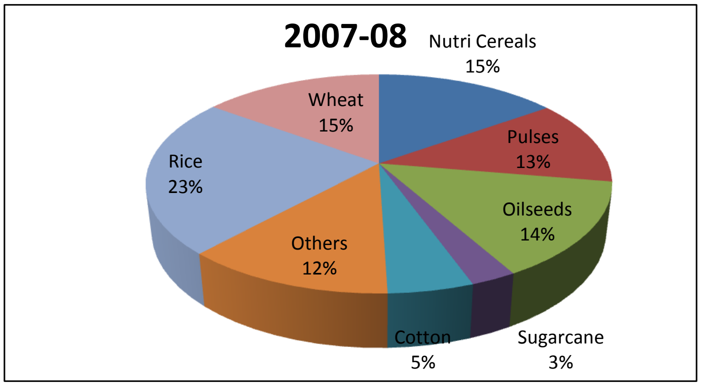

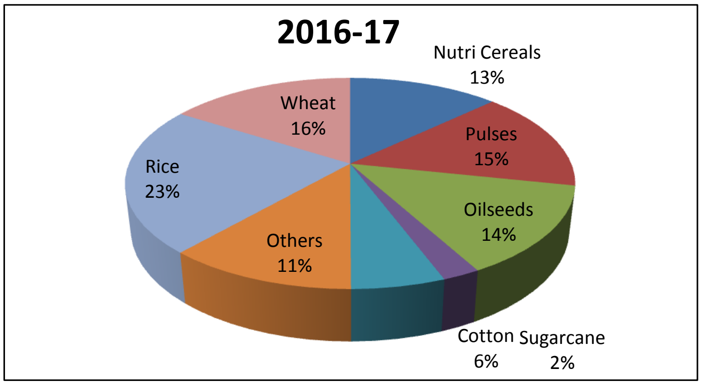

Source: Directorate of Economics & Statistics, DAC&FW

Table 4.4 Number and Area of Operational Holdings by Size Group

No. of Holdings: ('000 Number)
Area Operated: ('000 Hectares)
Average size: (Hectares)

Category of Holdings	Number of Holdings			Area			Average Size of Holdings		
	2000-01*	2005-06*	2010-11	2000-01*	2005-06*	2010-11	2000-01*	2005-06*	2010-11
(1)	(2)	(3)	(4)	(5)	(6)	(7)	(8)	(9)	(10)
Marginal	75408	83694	92826	29814	32026	35908	0.40	0.38	0.39
(Less than 1 hectare)	(62.9)	(64.8)	(67.1)	(18.7)	(20.2)	(22.5)			
Small	22695	23930	24779	32139	33101	35244	1.42	1.38	1.42
(1.0 to 2.0 hectares)	(18.9)	(18.5)	(17.9)	(20.2)	(20.9)	(22.1)			
Semi-Medium	14021	14127	13896	38193	37898	37705	2.72	2.68	2.71
(2.0 to 4.0 hectares)	(11.7)	(10.9)	(10.0)	(24.0.)	(23.9)	(23.6)			
Medium	6577	6375	5875	38217	36583	33828	5.81	5.74	5.76
(4.0 to 10.0 hectares)	(5.5)	(4.9)	(4.2)	(24.0)	(23.1)	(21.2)			
Large	1230	1096	973	21072	18715	16907	17.12	17.08	17.38
(10.0 hectares and above)	(1.0)	(0.8)	(0.7)	(13.2)	(11.8)	(10.6)			
**All Holdings**	**119931**	**129222**	**138348**	**159436**	**158323**	**159592**	**1.33**	**1.23**	**1.15**
	**(100.0)**	**(100.0)**	**(100.0)**	**(100.0)**	**(100.0)**	**(100.0)**			

*-Excluding Jharkhand

Source: Department of Agriculture, Cooperation & Farmers Welfare (Agriculture Census 2010-11, Phase-2).

Note: Figures in parentheses indicate percentage share in total.

Table 4.5: South West Monsoon Rainfall (1 June-30 September)

Year	Number of Meteorological Sub-Divisions @ Excess/ Normal Rainfall	Number of Meteorological Sub-Divisions @ Deficient/ Scanty Rainfall	Percentage of Districts with Normal/ Excess Rainfall	Actual Rainfall as % of Normal Rainfall (All India)
(1)	(2)	(3)	(4)	(5)
1990	32	3	88	119
1991	27	8	68	91
2001	30	5	68	92
2010	31	5	69	102
2011	33	3	76	101
2012	23	13	58	92
2013	30	6	72	106
2014	24	12	55	88
2015	19	17	51	86
2016	27	9	68	97
2017	30	6	66	95

@ Total number of Meteorological sub-divisions was 35 upto 2001. From 2002 onwards, the no. of meteorological sub-divisions is 36.

Excess : + 20% or more of Long Period Average Rainfall

Normal : Between + 19% and -19% of Long Period Average Rainfall

Deficient : Between -20% and -59% of Long Period Average Rainfall

Scanty : Between -60% and -99% of Long Period Average Rainfall

Source: Directorate of Economics and Statistics, DAC&FW

AREA, PRODUCTION AND YIELD OF PRINCIPAL CROPS

Table 5.1: Foodgrains: Area, Production and Yield during 2015-16 and 2016-17 in major Producing States alongwith coverage under Irrigation

Area - Million Hectares
Production - Million Tonnes
Yield - Kg./Hectare

State	2016-17#					2015-16					Area Under Irrigation(%) 2014-15*
	Area	% to All - India	Production	% to All - India	Yield	Area	% to All - India	Production	% to All - India	Yield	
(1)	(2)	(3)	(4)	(5)	(6)	(7)	(8)	(9)	(10)	(11)	(12)
Uttar Pradesh	19.92	15.56	49.14	17.83	2467	19.36	15.71	42.55	16.91	2198	80.4
Madhya Pradesh	17.03	13.30	32.98	11.96	1937	15.66	12.71	30.39	12.08	1941	59.7
Punjab	6.42	5.02	27.99	10.15	4360	6.65	5.40	28.40	11.29	4269	99.0
Rajasthan	14.11	11.02	19.28	7.00	1367	12.98	10.53	18.04	7.17	1390	35.9
Haryana	4.59	3.59	17.16	6.22	3735	4.48	3.64	16.36	6.50	3648	92.7
West Bengal	5.98	4.67	17.06	6.19	2853	6.38	5.18	18.01	7.16	2823	48.4
Maharashtra	12.16	9.50	15.79	5.73	1298	11.21	9.10	8.75	3.48	781	18.0
Bihar	6.61	5.17	15.58	5.65	2355	6.57	5.33	14.51	5.77	2208	69.8
Andhra Pradesh	3.97	3.10	10.37	3.76	2610	4.14	3.36	10.63	4.23	2571	66.5
Karnataka	7.29	5.69	9.64	3.50	1323	7.33	5.95	9.92	3.94	1354	27.3
Chhattisgarh	5.05	3.95	9.23	3.35	1827	4.99	4.05	6.65	2.65	1334	31.6
Odisha	4.80	3.75	9.06	3.29	1887	4.82	3.91	6.41	2.55	1330	29.0
Telangana	3.29	2.57	8.37	3.03	2545	2.18	1.77	5.13	2.04	2353	64.3
Gujarat	3.80	2.97	7.42	2.69	1953	3.14	2.55	6.28	2.50	2000	45.4
Tamil Nadu	2.99	2.33	6.22	2.26	2084	3.75	3.04	11.48	4.56	3063	56.8
Assam	2.67	2.08	5.47	1.98	2049	2.68	2.18	5.36	2.13	1997	10.4
Jharkhand	2.89	2.25	5.37	1.95	1860	2.65	2.15	4.09	1.63	1546	8.8
Uttarakhand	0.88	0.69	1.87	0.68	2131	0.88	0.71	1.75	0.69	1989	44.8
Others	3.59	2.81	7.69	2.79	@	3.38	2.74	6.86	2.73	@	-
**All India**	**128.03**	**100.00**	**275.68**	**100.00**	**2153**	**123.22**	**100.00**	**251.57**	**100.00**	**2042**	**53.1**

@ - Since area/ production is low in individual states, yield rate is not worked out.

Note: States have been arranged in descending order of percentage share of production during 2016-17.

* Provisional # Fourth Advance Estimates.

Source : Directorate of Economics & Statistics, DAC&FW

Table 5.2 (a): Area under Major Crops from 1950-51 onwards (in million hectares)

Crops	1950-51	1960-61	1970-71	1980-81	1990-91	2000-01	2010-11	2011-12	2012-13	2013-14	2014-15	2015-16	2016-17*
(1)	(2)	(3)	(4)	(5)	(6)	(7)	(8)	(9)	(10)	(11)	(12)	(13)	(14)
Rice	30.81	34.13	37.59	40.15	42.69	44.71	42.86	44.01	42.75	44.14	44.11	43.50	43.19
Wheat	9.75	12.93	18.24	22.28	24.17	25.73	29.07	29.86	30.00	30.47	31.47	30.42	30.60
Nutri Cereals	37.67	44.96	45.95	41.78	36.32	30.26	28.34	26.42	24.76	25.22	25.17	24.39	24.77
Pulses	19.09	23.56	22.54	22.46	24.66	20.35	26.40	24.46	23.26	25.21	23.55	24.91	29.46
Foodgrains	97.32	115.58	124.32	126.67	127.84	121.05	126.67	124.75	120.78	125.04	124.30	123.22	128.03
Oilseeds	10.73	13.77	16.64	17.60	24.15	22.77	27.22	26.31	26.48	28.05	25.59	26.08	26.20
Sugarcane	1.71	2.42	2.62	2.67	3.69	4.32	4.88	5.04	5.00	4.99	5.06	4.92	4.38
Cotton	5.88	7.61	7.61	7.82	7.44	8.53	11.24	12.18	11.98	11.96	12.81	12.29	10.84
Jute & Mesta	0.57	0.90	1.08	1.30	1.02	1.02	0.87	0.90	0.86	0.83	0.81	0.78	0.76

* 4th Advance Estimates

Source: Department of Agriculture, Cooperation & Farmers Welfare

Table 5.2 (b): Production of Major Agricultural Crops

(Million Tonnes)

S.No.	Crops	1950-51	1960-61	1970-71	1980-81	1990-91	2000-01	2010-11	2011-12	2012-13	2013-14	2014-15	2015-16	2016-17*
(1)	(2)	(3)	(4)	(5)	(6)	(7)	(8)	(9)	(10)	(11)	(12)	(13)	(14)	(15)
1	**Foodgrains**	**50.82**	**82.02**	**108.42**	**129.59**	**176.39**	**196.81**	**244.49**	**259.29**	**257.13**	**265.04**	**252.02**	**251.57**	**275.68**
	Rice	20.58	34.58	42.22	53.63	74.29	84.98	95.98	105.30	105.23	106.65	105.48	104.41	110.15
	Wheat	6.46	11.00	23.83	36.31	55.14	69.68	86.87	94.88	93.51	95.85	86.53	92.29	98.38
	Maize	1.73	4.08	7.49	6.96	8.96	12.04	21.73	21.76	22.26	24.26	24.17	22.57	26.26
	Nutri Cereals	15.38	23.74	30.55	29.02	32.70	31.08	43.40	42.01	40.04	43.29	42.86	38.52	44.19
2	**Pulses**	**8.41**	**12.70**	**11.82**	**10.63**	**14.26**	**11.08**	**18.24**	**17.09**	**18.34**	**19.25**	**17.15**	**16.35**	**22.95**
	Gram	3.65	6.25	5.20	4.33	5.36	3.86	8.22	7.70	8.83	9.53	7.33	7.06	9.33
	Tur (Arhar)	1.72	2.07	1.88	1.96	2.41	2.25	2.86	2.65	3.02	3.17	2.81	2.56	4.78
	Lentil (Masur)	--	--	0.37	0.47	0.85	0.92	0.94	1.06	1.13	1.02	1.04	0.98	-
3	**Oilseeds**	**5.16**	**6.98**	**9.63**	**9.37**	**18.61**	**18.44**	**32.48**	**29.80**	**30.94**	**32.75**	**27.51**	**25.25**	**32.10**
	Groundnut	3.48	4.81	6.11	5.01	7.51	6.41	8.26	6.96	4.70	9.71	7.40	6.73	7.565
	Rapeseed & Mustard	0.76	1.35	1.98	2.30	5.23	4.19	8.18	6.60	8.03	7.88	6.28	6.80	7.98
	Soy Bean	--	--	0.01	0.44	2.60	5.28	12.74	12.21	14.67	11.86	10.37	8.57	13.79
	Sunflower	--	--	0.08	0.07	0.87	0.65	0.65	0.52	0.54	0.50	0.43	0.30	0.24
4	**Cotton #**	3.04	5.60	4.76	7.01	9.84	9.52	33.00	35.20	34.22	35.90	34.80	30.01	33.09
5	**Jute & Mesta @**	3.31	5.26	6.19	8.16	9.23	10.56	10.62	11.40	10.93	11.68	11.13	10.52	10.60
6	**Sugarcane**	57.05	110.00	126.37	154.25	241.05	295.96	342.38	361.04	341.20	352.14	362.33	348.45	306.72
7	**Tobacco**	0.26	0.31	0.36	0.48	0.56	0.34	0.80	0.75	0.66	0.74	0.84	0.80	-

* 4th Advance Estimates # Million bales of 170 kg. each @ Million bales of 180 kg. each

Source: Directorate of Economics and Statistics, DAC&FW

Table 5.3: Target and Achievement of Production of Major Crops

(Million Tonnes)

Crop	2013-14		2014-15		2015-16		2016-17*	
	Target	Achievement	Target	Achievement	Target	Achievement	Target	Achievement
(1)	(2)	(3)	(4)	(5)	(6)	(7)	(8)	(9)
Rice	105.00	106.65	106.00	105.48	106.10	104.41	108.50	110.15
Wheat	92.50	95.85	94.00	86.53	94.75	92.29	96.50	98.38
Nutri Cereals	42.50	43.29	41.50	42.86	43.20	38.52	44.35	44.19
Pulses	19.00	19.25	19.50	17.15	20.05	16.35	20.75	22.95
Foodgrains	259.00	265.04	261.00	252.02	264.10	251.57	270.10	275.68
Oilseeds	31.00	32.75	33.00	27.51	33.00	25.25	35.00	32.10
Sugarcane	340.00	352.14	345.00	362.33	355.00	348.45	355.00	306.72
Cotton #	35.00	35.90	35.00	34.80	35.15	30.01	36.00	33.09
Jute & Mesta@	12.00	11.69	11.20	11.13	11.70	10.52	11.70	10.60

* 4th Advance Estimates. @ Million Bales of 180 kg. each.

\# Million Bales of 170 kg. each.

Source: Directorate of Economics and Statistics, DAC&FW

Table 5.4: Three Largest Producing States of Important Crops during 2016-17*

Production: Million Tonnes

Group of Crops	Crops	States	Production	% Share in All India Production
(1)	(2)	(3)	(4)	(5)
**I. Foodgrains**	Rice	West Bengal	15.09	13.70
		Uttar Pradesh	12.95	11.76
		Punjab	11.03	10.01
		**All - India**	**110.15**	**100.00**
	Wheat	Uttar Pradesh	30.06	30.55
		Madhya Pradesh	17.94	18.24
		Punjab	16.44	16.71
		**All - India**	**98.38**	**100.00**
	Maize	Maharashtra	3.80	14.47
		Karnataka	3.26	12.41
		Madhya Pradesh	3.17	12.07
		**All - India**	**26.26**	**100.00**
	Total Nutri Cereals	Maharashtra	6.96	15.75
		Rajasthan	6.78	15.34
		Karnataka	5.22	11.81
		**All - India**	**44.19**	**100.00**
	Total Pulses	Madhya Pradesh	6.25	27.23
		Maharashtra	3.81	16.60
		Rajasthan	3.06	13.33
		**All - India**	**22.95**	**100.00**
	Total Foodgrains	Uttar Pradesh	49.14	17.83
		Madhya Pradesh	32.98	11.96
		Punjab	27.99	10.15
		**All - India**	**275.68**	**100.00**
**II .Oilseeds**	Groundnut	Gujarat	3.16	41.80
		Rajasthan	1.14	15.08
		Andhra Pradesh	0.71	9.39
		**All - India**	**7.56**	**100.00**
	Rapeseed & Mustard	Rajasthan	3.71	46.49
		Madhya Pradesh	0.92	11.53
		Haryana	0.90	11.28
		**All - India**	**7.98**	**100.00**
	Soy Bean	Madhya Pradesh	7.08	51.34
		Maharashtra	4.77	34.59
		Rajasthan	1.13	8.19
		**All - India**	**13.79**	**100.00**
	Sunflower	Karnataka	0.10	41.67
		Andhra Pradesh	0.02	8.75
		Bihar	0.02	*8.33*
		**All - India**	**0.24**	**100.00**
	Total Oilseeds	Madhya Pradesh	8.68	27.04
		Rajasthan	6.31	19.66
		Maharashtra	5.26	16.39

contd.

Table 5.4: Three Largest Producing States of Important Crops during 2016-17*

Production: Million Tonnes

Group of Crops	Crops	States	Production	% Share in All India Production
		**All - India**	**32.10**	**100.00**
**III. Other Cash Crops**	Sugarcane	Uttar Pradesh	144.78	1363.28
		Maharashtra	50.64	476.84
		Karnataka	23.54	221.66
		**All - India**	**306.72**	**100.00**
	Cotton @	Maharashtra	10.62	104.58
		Gujarat	8.22	37.28
		Telangana	2.93	420.99
		**All - India**	**33.09**	**100.00**
	Jute & Mesta$	West Bengal	7.86	75.11
		Bihar	1.68	16.06
		Assam	0.83	7.93
		**All - India**	**10.60**	**100.00**

Note:*Production Estimates are as per 4th Advance Estimates

@ : Production in million bales of 170 kg. each.

$: Production in million bales of 180 kg. each.

Source: Directorate of Economics and Statistics, DAC&FW

Table 5.5: Production of Oilseeds & Oils and Net Availability of Edible Oils

(in lakh tonnes)

Oilseeds / Oils	2014-15		2015-16*		2016-17**	
	Oilseeds	Oils	Oilseeds	Oils	Oilseeds	Oils
(1)	(2)	(3)	(4)	(5)	(6)	(7)
**A. Primary Source**						
Groundnut	74.02	17.02	67.71	15.36	75.65	17.16
Rapeseed & Mustard	62.82	19.47	68.21	21.16	79.77	24.74
Soy Bean	103.74	16.60	85.92	13.76	137.94	22.1
Sunflower	4.34	1.43	3.31	1.10	2.41	0.8
Sesamum	8.68	2.57	8.66	2.70	7.84	2.45
Nigerseed	0.76	0.23	0.77	0.22	0.83	0.24
Safflower	0.90	0.27	0.64	0.18	0.78	0.22
Castor	18.70	7.48	16.50	5.80	14.21	4.99
Linseed	1.55	0.47	1.32	0.34	1.54	0.39
**Sub Total**	**275.51**	**65.54**	**253.04**	**60.62**	**320.97**	**73.09**
**B. Secondary Source**						
Coconut	-	4.80	-	4.32	-	5.2
Palm Oil	-	-		-	-	2.3
Cottonseed	-	12.15	-	10.05	-	12.24
Ricebran	-	9.20	-	9.90	-	10.31
Solvent Extracted Oils	-	3.00	-	3.50	-	2.85
Tree & Forest Origin	-	1.60	-	1.50	-	1.5
**Sub Total**	-	**30.75**	-	**29.27**	-	**34.4**
**Total(A+B)**	-	**96.29**	-	**89.89**	-	**107.49**
**C. Less: Exports & Industrial Use**	-	**5.94**	-	**5.50**	-	**6.5**
**D. Net Domestic Availability of Edible Oils**	-	**92.06**	-	**86.37**	-	**100.99**
**E.Import of Edible Oils $**	-	**138.53**	-	**148.20**	-	**153.11**
**F. Total Availability/ Consumption of Edible Oils**	-	**230.59**	**-**	**234.57**		**254.1**

* Based on Final Estimate released by Ministry of Agriculture and Farmers Welfare on 16.08.2017

** Based on 4th Advance Estimates released by Ministry of Agriculture and Farmers Welfare on 16.08.2017

$ Directorate General of Commercial Intelligence & Statistics (Department of Commerce)

Source: Directorate of Vanaspati, Vegatable Oils and Fats

Table 5.6: All India Crop-wise Yield

(Quintal/ hectare)

Crops	1950-51	1990-91	2000-01	2010-11	2011-12	2012-13	2013-14	2014-15	2015-16	2016-17*
(1)	(2)	(3)	(4)	(5)	(6)	(7)	(8)	(9)	(10)	(11)
Rice	6.68	17.40	19.01	22.39	23.93	24.61	24.16	23.91	24.00	25.50
Jowar	3.53	8.14	7.64	9.49	9.57	8.50	9.57	8.85	6.97	8.89
Bajra	2.88	6.58	6.88	10.79	11.71	11.98	11.84	12.54	11.32	13.11
Maize	5.47	15.18	18.22	25.40	24.78	25.66	26.76	26.30	25.63	26.64
Wheat	6.63	22.81	27.08	29.88	31.77	31.17	31.45	27.50	30.34	32.16
Nutri Cereals	4.08	9.00	10.27	15.31	15.90	16.17	17.17	17.03	15.79	17.84
Gram	4.82	7.12	7.44	8.95	9.28	10.36	9.60	8.88	8.40	9.73
Tur or Arhar	7.88	6.73	6.18	6.55	6.62	7.76	8.13	7.30	6.46	8.85
Total Pulses	4.41	5.78	5.44	6.91	6.99	7.89	7.64	7.28	6.56	7.79
**Total Foodgrains**	**5.22**	**13.80**	**16.26**	**19.30**	**20.78**	**21.29**	**21.20**	**20.28**	**20.42**	**21.53**
Sugarcane	334.22	653.95	685.78	700.91	703.17	682.54	705.22	715.12	707.22	698.86
Groundnut	7.75	9.04	9.77	14.11	13.05	9.95	17.64	15.52	14.65	14.24
Mustard	3.68	9.04	9.36	11.85	11.45	12.62	11.85	10.83	11.83	13.24
Soy Bean	4.26	10.15	8.23	13.27	12.07	13.53	10.12	9.51	7.38	12.19
Sunflower	6.53	5.35	6.05	7.01	6.92	6.55	7.50	7.36	6.08	6.99
**Total Oilseeds**	**4.81**	**7.71**	**8.10**	**11.93**	**11.35**	**11.68**	**11.68**	**10.75**	**9.68**	**12.25**
Cotton	0.88	2.25	1.90	4.99	4.91	4.86	5.10	4.62	4.15	5.19
Tobacco	7.31	13.53	13.18	16.87	16.13	15.42	16.12	18.42	17.81	NA

NA: Not Available * 4th advanced estimates

Source: Directorate of Economics and Statistics, DAC&FW

Table 5.7: All India Crop-wise Irrigated Area

(Percentage)

Crops	1950-51	1990-91	2000-01	2010-11	2011-12 (P)	2012-13	2013-14	2014-15 (P)
(1)	(2)	(3)	(4)	(5)	(6)	(7)	(8)	(9)
Rice	31.7	45.5	53.6	58.8	58.6	58.5	59.7	60.1
Jowar	3.0	5.6	7.9	8.7	9.7	9.7	9.6	9.9
Bajra	3.4	5.1	8.0	8.0	8.1	9.1	9.0	9.5
Maize	11.4	19.7	22.4	24.5	25.7	25.9	27.2	26.6
Wheat	34.0	81.1	88.1	92.2	93.0	93.5	93.6	94.2
Barley	NA	NA	65.5	69.9	74.8	76.4	76.6	80.3
**Total Cereals**	**NA**	**NA**	**49.8**	**56.3**	**57.7**	**58.6**	**59.5**	60.1
Gram	12.5	20.5	30.9	29.7	33.5	36.5	35.2	38.6
Tur or Arhar	0.5	5.5	4.2	4.0	3.8	3.9	4.3	4.0
Total Pulses	9.4	10.5	12.5	14.9	16.1	18.5	19.7	19.9
**Total Foodgrains**	**18.1**	**35.1**	**43.4**	**48.1**	**49.8**	**51.3**	**51.9**	53.1
Sugarcane	67.3	86.9	92.1	92.7	94.8	95.2	95.3	90.2
Groundnut	NA	18.6	17.6	21.8	24.3	25.3	25.8	26.7
Rapeseed & Mustard	NA	59.8	66.1	69.8	73.5	76.5	76.6	76.6
Soy Bean	NA	NA	1.4	0.6	0.6	0.6	0.6	0.7
Sunflower	NA	NA	27.6	31.2	32.3	29.0	28.6	30.1
**Total Oilseeds**	**NA**	**22.9**	**23.0**	**24.9**	**27.5**	**28.2**	**27.3**	27.4
Cotton	8.2	32.9	34.3	33.8	35.9	33.8	32.5	33.7
Tobacco	NA	43.2	52.8	46.4	51.0	53.8	51.6	58.8
**Total area under all crops**	**…**	**…**	**40.2**	**45.0**	**46.9**	**47.5**	**47.7**	**48.6**

NA: Not Available , (P): Provisional

Source: Directorate of Economics and Statistics, DAC&FW

Table 5.8: State-wise Coverage of Irrigated Area under Major Crops during 2014-15

(Percentage)

State	Rice	Wheat	Total Cereals	Total Pulses	Total Foodgrains	Sugarcane	Fruits & Vegetables	Total Oilseeds	Cotton	Total Irrigated area under all crops
(1)	(2)	(3)	(4)	(5)	(6)	(7)	(8)	(9)	(10)	(11)
Andhra Pradesh	97.1	15.0	89.5	2.0	66.5	92.4	49.5	18.7	19.9	50.5
Arunachal Pradesh	20.6	52.9	20.9	-	20.9	0.0	112.6	0.0	-	18.7
Assam	11.0	10.9	10.9	1.7	10.4	1.5	0.0	2.5	0.0	9.2
Bihar	65.0	94.9	75.2	4.5	69.8	79.3	68.2	57.2	-	68.7
Chhattisgarh	35.7	75.7	34.9	15.0	31.6	98.9	55.6	4.5	57.6	31.2
Goa	33.7	-	33.7	97.6	44.5	100.0	10.1	16.5	-	24.6
Gujarat*	61.5	90.8	52.5	13.1	45.4	94.5	91.9	29.8	58.7	47.1
Haryana	99.9	99.5	94.0	20.7	92.7	100.0	96.8	83.1	99.8	89.1
Himachal Pradesh*	66.1	21.8	21.4	12.3	21.0	54.1	17.3	16.4	100.0	21.0
Jammu & Kashmir*	90.5	28.5	40.9	13.5	40.2	40.9	40.5	69.7	-	42.8
Jharkhand	5.0	90.9	9.4	3.9	8.8	68.3	71.4	23.2	150.0	14.3
Karnataka	76.0	60.9	36.2	8.6	27.3	99.4	49.5	29.9	27.2	34.2
Kerala	76.1	0.0	75.9	0.0	74.6	99.9	14.2	20.8	0.0	17.9
Madhya Pradesh	34.2	93.4	67.2	42.8	59.7	99.9	88.4	5.3	60.4	43.3
Maharashtra*	26.1	73.9	21.0	10.9	18.0	70.5	81.6	3.2	2.7	18.2
Manipur*	30.7	0.0	27.3	0.0	24.4	0.0	0.0	0.0	-	18.0
Meghalaya	90.7	100.0	76.3	0.0	71.9	0.0	15.9	45.5	0.0	37.1
Mizoram	72.5	-	58.2	94.3	63.0	0.0	0.0	0.0	0.0	14.5
Nagaland	51.7	17.2	36.5	2.6	32.5	0.0	0.0	4.6	0.0	21.2
Odisha	33.3	100.0	32.0	2.2	29.0	100.0	100.0	12.7	-	28.7
Punjab	99.7	99.1	99.0	87.7	99.0	97.7	99.7	86.8	100.0	98.7
Rajasthan	68.6	99.6	41.3	20.7	35.9	98.4	97.0	63.8	95.2	42.0
Sikkim*	98.8	0.4	18.8	0.2	17.0	-	3.4	0.2	-	8.9
Tamil Nadu	94.4	100.0	71.8	10.9	56.8	100.0	54.5	66.5	26.5	56.6
Telangana	98.1	99.2	75.3	5.1	64.3	100.0	80.2	40.5	12.5	47.6
Tripura*	33.9	100.0	33.5	28.3	33.3	50.3	23.7	18.8	0.0	24.0
Uttarakhand	70.0	59.5	47.3	7.9	44.8	98.7	39.7	25.0	-	49.5
Uttar Pradesh	86.7	98.8	87.0	27.4	80.4	95.4	88.6	42.5	97.2	80.2
West Bengal*	46.9	98.7	49.7	15.0	48.4	84.6	89.5	79.1	80.0	58.8
**All India**	**60.1**	**94.2**	**60.1**	**19.9**	**53.1**	**90.2**	**65.6**	**27.4**	**33.7**	**48.6**

* The figures related to irrigated area (Part-II) are either estimated based on the data for the latest available year received from the State/UT or are estimated/taken from Agriculture Census.

Source: Directorate of Economics and Statistics, DAC&FW

HORTICULTURE STATISTICS

Table: 6.1 Area and Production of Horticulture Crops - All India

Area in '000 Ha
Production in '000 MT

Crops	2015-16 (Final)		2016-17 (3rd Adv. Est.)	
	Area	Production	Area	Production
(1)	(2)	(3)	(4)	(5)
**Fruits**				
Almond	12	8	12	8
Aonla/Gooseberry	88	972	91	1025
Apple	277	2521	278	2258
Banana	841	29135	852	30275
Ber	44	425	50	526
Citrus				
(i) Lime/Lemon	245	2438	240	2555
(ii) Mandarin	397	4113	424	4640
(iii) Sweet Orange(Mosambi)	244	3468	209	3187
(iv) Others	138	1562	165	1670
**Citrus Total (i to iv)**	1024	11581	1037	12053
Custardapple	37	298	45	385
Grapes	122	2590	136	2784
Guava	255	4048	260	3615
Jackfruit	151	1732	153	1722
Kiwi	4	11	4	12
Litchi	90	559	91	578
Mango	2209	18643	2267	20295
Muskmelon	45	935	47	987
Papaya	132	5667	138	6145
Passion Fruit	13	78	14	86
Peach	18	107	18	105
Pear	40	323	42	352
Picanut	1	1	1	1
Pineapple	110	1924	114	1969
Plum	22	82	23	80
Pomegranate	197	2306	216	2521
Sapota	107	1294	105	1252
Strawberry	1	5	1	5
Walnut	92	229	93	230
Watermelon	95	2325	91	2169
Others	275	2386	279	2268
**Total Fruits**	**6301**	**90183**	**6457**	**93707**
**Vegetables**				
Beans	232	2334	230	2408
Bittergourd	93	1046	98	1106
Bottlegourd	149	2458	155	2573
Brinjal	663	12515	727	12323
Cabbage	394	8806	394	8720
Capsicum	46	288	24	333
Carrot	82	1338	88	1379
Cauliflower	426	8090	451	8484
Cucumber	71	1202	77	1246
Chillies (Green)	292	2955	292	3390
Elephant Foot Yam	28	733	29	731
Mushroom	170	436	183	459
Okra/Ladyfinger	511	5849	501	5783
Onion	1320	20931	1293	21718
Parwal/Pointed gourd	18	264	20	289
Peas	498	4811	540	5252

contd.

Production in '000 MT

Crops	2015-16 (Final)		2016-17 (3rd Adv. Est.)	
	Area	Production	Area	Production
(1)	(2)	(3)	(4)	(5)
Potato	2117	43417	2151	48237
Radish	199	2844	203	2889
Pumpkin/Sitaphal/Kaddu	68	1509	72	1611
Sweet Potato	126	1454	132	1473
Tapioca	204	4344	203	4421
Tomato	774	18732	799	19542
Others	1625	22707	1631	21811
**Total Vegetables**	**10106**	**169064**	**10295**	**176177**
**Aromatic**	634	1022	665	1042
Flowers Cut		528		582
Flowers Loose	278	1656	328	1695
**Total Flowers**	**278**	**2184**	**328**	**2277**
**Honey**		88		95
**Plantation Crops**				
Arecanut	474	714	459	718
Cashewnut	1036	671	1041	779
Cocoa	81	17	83	19
Coconut	2088	15256	2076	16837
**Total Plantation**	**3680**	**16658**	**3659**	**18353**
**Spices**				
Ajwain	24	16	29	26
Cardamom	86	24	85	28
Chillies (Dried)	811	1520	845	2126
Cinnamon/Tejpata	3	5	3	5
Celery,Dill & Poppy	26	23	36	35
Clove	2	1	2	1
Coriander	582	585	704	900
Cumin	808	503	781	489
Fenugreek	219	247	210	256
Fennel	76	129	91	153
Garlic	281	1617	322	1697
Ginger	164	1109	168	1076
Nutmeg	21	14	23	15
Pepper	129	55	131	72
Vanilla	4	0	4	0
Tamarind	53	194	49	191
Turmeric	186	943	222	1132
**Total Spices**	**3474**	**6988**	**3705**	**8202**
**Total**	**24472**	**286188**	**25109**	**299853**

Source : Department of Agriculture, Cooperation & Farmers Welfare

Table 6.2: All-India Area, Production and Yield of Potato and Onion

Area - Million Hectares
Production - Million Tonnes
Yield - Kg./Hectare

Year	Potato			Onion		
	Area	Production	Yield	Area	Production	Yield
(1)	(2)	(3)	(4)	(5)	(6)	(7)
1980-81	0.73	9.67	13258	0.25	2.50	9961
1990-91	0.94	15.21	16254	0.30	3.23	10686
2000-01	1.22	22.49	18404	0.42	4.55	10786
2010-11	1.86	42.34	22724	1.06	15.12	14210
2011-12	1.91	41.48	21753	1.09	17.51	16109
2012-13	1.99	45.34	22760	1.05	16.81	15989
2013-14	1.97	41.56	21060	1.20	19.40	16120
2014-15	2.08	48.01	23126	1.17	18.93	16111
2015-16	2.11	43.40	20569	1.32	20.93	15857
2016-17 (3rd Adv. Est.)	2.15	48.23	22433	1.29	21.72	16801

Source : Department of Agriculture, Cooperation & Farmers Welfare

Table 6.3: All-India Area, Production and Yield of Banana

Area - Million Hectares
Production - Million Tonnes
Yield - Kg./Hectare

Year	Banana		
	Area	Production	Yield
(1)	(2)	(3)	(4)
2000-01	0.46	12.90	28133
2009-10	0.77	26.47	34363
2010-11	0.83	29.78	35880
2011-12	0.80	28.46	35725
2012-13	0.78	26.51	34161
2013-14	0.80	29.72	37037
2014-15	0.82	29.22	35549
2015-16	0.84	29.13	34635
2016-17 (3rd Adv. Est.)	0.85	30.28	35544

Source : Department of Agriculture, Cooperation & Farmers Welfare

Table:6.4 Foodgrain and Horticulture Production in India

(Million Tonnes)

Year	Foodgrains Production	Horticulture Production
(1)	(2)	(3)
2004-05	198.36	166.94
2005-06	208.60	182.82
2006-07	217.28	191.81
2007-08	230.78	211.24
2008-09	234.47	214.72
2009-10	218.11	223.09
2010-11	244.50	240.53
2011-12	259.29	257.28
2012-13	257.13	268.85
2013-14	265.57	277.35
2014-15	252.02	280.99
2015-16	251.57	286.19
2016-17*	275.68	299.85

* 4th Advance Estimate for Food grain production and 3rd Advance Estimate for Horticulture Production

Source: Department of Agriculture, Cooperation & Farmers Welfare

Chart 6(a): Trends in Foodgrain and Horticulture Production

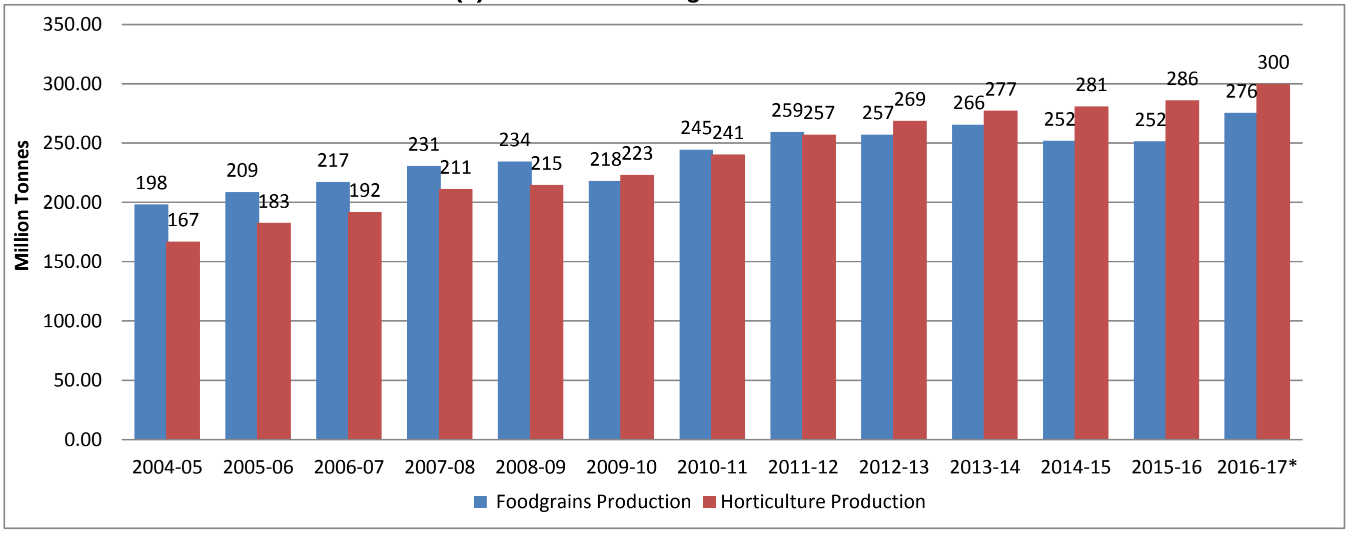

Source: Department of Agriculture, Cooperation & Farmers Welfare

Table 6.5: Value of Output from Horticulture Crops vis-à-vis All Agricultural Crops (at 2011-12 prices)

(₹ in '00 crores)

Item/Year	2011-12	2012-13	2013-14	2014-15	2015-16
(1)	(2)	(3)	(4)	(5)	(6)
i) Total Fruits & Vegetables	2657	2793	2929	3037	3094
ii) Total Condiments & Spices	385	377	416	442	464
iii) Total Floriculture	174	173	178	169	176
iv) Total Plantation crops	257	253	255	270	271
**I. Total Horticulture crops (i+ii+iii+iv)**	**3473**	**3596**	**3778**	**3918**	**4005**
**II. All Agricultural crops**	**11915**	**11986**	**12571**	**12278**	**12147**
Percentage share of Value of Output from Horticulture to total Agricultural Output	*29.1*	*30.0*	*30.1*	*31.9*	*33.0*

Source: National Accounts Statistics 2017, CSO

LIVESTOCK STATISTICS

Table 7.1: Livestock Population in India

(Million Numbers)

Species	1951	1982	1987	1992	1997	2003	2007	2012
(1)	(2)	(3)	(4)	(5)	(6)	(7)	(8)	(9)
Cattle	155.30	192.45	199.69	204.58	198.88	185.18	199.08	190.90
Buffalo	43.40	69.78	75.97	84.21	89.92	97.92	105.34	108.70
**Total Bovines**	**198.70**	**262.36**	**275.82**	**289.00**	**289.00**	**283.10**	**304.76**	**299.98**
Sheep	39.10	48.76	45.70	50.78	57.49	61.47	71.56	65.07
Goats	47.20	95.25	110.21	115.28	122.72	124.36	140.54	135.17
Horses & Ponies	1.50	0.90	0.80	0.82	0.83	0.75	0.61	0.63
Camels	0.60	1.08	1.00	1.03	0.91	0.63	0.52	0.40
Pigs	4.40	10.07	10.63	12.79	13.29	13.52	11.13	10.29
Mules	0.06	0.13	0.17	0.19	0.22	0.18	0.14	0.20
Donkeys	1.30	1.02	0.96	0.97	0.88	0.65	0.44	0.32
Yaks	NC	0.13	0.04	0.06	0.06	0.06	0.08	0.08
**Total Livestock**	**292.80**	**419.59**	**445.29**	**470.86**	**485.39**	**485.00**	**529.70**	**512.06**
Poultry	73.50	207.74	275.32	307.07	347.61	489.1	648.88	729.21
Dogs	NC	18.54	17.95	21.77	25.48	29.03	19.09	11.67
Rabbits	NC	NC	NC	NC	NC	0.48	0.42	0.59

Source : Department of Animal Husbandry, Dairying & Fisheries.

NC : Not Collected

Note: Total may not tally due to rounding off

Table 7.2 All India Production of Milk, Eggs and Wool

Year	Milk (Million Tonnes)	Eggs (Billion Nos.)	Wool (Million Kgs.)
(1)	(2)	(3)	(4)
1990-91	53.9	21.1	41.2
2000-01	80.6	36.6	48.4
2009-10	116.4	60.3	43.1
2010-11	121.8	63.0	43.0
2011-12	127.9	66.5	44.7
2012-13	132.4	69.7	46.1
2013-14	137.7	74.8	47.9
2014-15	146.3	78.5	48.1
2015-16	155.5	82.9	43.6
2016-17	163.7	88.1	43.5

Source: Department of Animal Husbandry, Dairying & Fisheries.

Table 7.3 Fish Production in India

('000 Tonnes)

Year	Marine	Inland	Total
(1)	(2)	(3)	(4)
1950-51	534	218	752
1980-81	1555	887	2442
1990-91	2300	1536	3836
2000-01	2811	2845	5656
2009-10	3104	4894	7998
2010-11	3250	4981	8231
2011-12	3372	5294	8666
2012-13	3321	5719	9040
2013-14	3443	6136	9579
2014-15	3569	6691	10260
2015-16	3600	7162	10762
2016-17 (P)	3641	7768	11409

Source: Department of Animal Husbandry Dairying & Fisheries

(P) : Provisional

AGRICULTURAL INPUTS, COSTS & PRICES

Table 8.1: Production and Use of Agricultural Inputs in India

Programme	Unit	2000-01	2010-11	2011-12	2012-13	2013-14	2014-15	2015-16	2016-17
(1)	(2)	(3)	(4)	(5)	(6)	(7)	(8)	(9)	(10)
**1. Seeds**									
(i) Production of Breeder Seeds	Thousand Qtls.	42.69	118.85	123.38	110.20	82.29	86.21	90.37	110.71
(ii) Production of Foundation Seeds	Lakh Qtls.	5.91	17.53	22.36	16.17	17.43	15.76	14.95	22.09
(iii) Distribution of Certified/Quality Seeds	Lakh Qtls.	86.27	277.34	294.85	313.44	301.39	303.12	304.04	348.58
**2. Consumption of Chemical Fertilisers**									
Nitrogenous (N)	Lakh Tonnes	109.20	165.58	173.00	168.21	167.50	169.46	173.72	167.35
Phosphatic(P)	Lakh Tonnes	42.15	80.50	79.14	66.53	56.33	60.98	69.79	67.05
Potassic(K)	Lakh Tonnes	15.67	35.14	25.76	20.62	20.99	25.32	24.02	25.08
Total (N+P+K)	Lakh Tonnes	167.02	281.22	277.90	255.36	244.82	255.76	267.53	259.49
Per Hectare *	Kg.	89.63	142.52	142.05	131.36	118.49	127.45	130.66	123.41
**3. Consumption of Pesticides** (Technical Grade Material)	Thousand Tonnes	43.58	55.54	52.98	45.62	60.28	56.12	50.41	52.75**
**4. Area Covered Under Soil Conservation** (Cumulative)	Lakh Hectares	4.36	7.49	4.72	5.46	-	-	-	-

* Per capita consumption from Gross Cropped Area upto 2016-17 is based on State Governments estimates

** as on 20.11.2017 (doesn't include information from State of Telangan and Assam)

# : Soil Consevation programmes namely; National Watershed Development Project for Rainfed Areas, Centrally Sponsored Programme of Soild Conservation in the catchment of River Vally & Flood Prone Rivers (RVP&FPR), Reclamation & Development of Alkali & Acid Soils (RDAAS) Watershed Development Project in Shifting Cultivation Area (WDPSCA), Watershed Development Fund have been closed from Ist April 2013.

Source: Department of Agriculture, Cooperation & Farmers Welfare

Table 8.2 : Crop-wise requirement and availability of Certified/ Quality Seeds

(Lakh Quintals)

Year	2014-15				2015-16				2016-17			
	Require-	Availability			Require-	Availability			Require-	Availability		
Crop Name	ment	Public	Private	Total	ment	Public	Private	Total	ment	Public	Private	Total
(1)	(2)	(3)	(4)	(5)	(6)	(7)	(8)	(9)	(10)	(11)	(12)	(13)
Wheat	112.53	44.78	72.07	116.86	113.46	51.25	66.73	117.98	117.55	61.67	74.91	136.58
Paddy	84.8	46.46	46.46	92.92	82.86	47.96	47.14	95.1	87.74	63.14	37.33	100.47
Ragi	0.31	0.25	0.11	0.36	0.32	0.47	0.07	0.53	0.34	0.43	0.13	0.56
Barley	2.23	0.61	2.26	2.87	2.25	0.69	2.48	3.17	2.44	0.58	2.37	2.95
Maize	10.84	1.15	11.1	12.25	10.7	0.73	11.96	12.69	12.47	1.67	11.87	13.54
Bajra	2.42	0.16	2.53	2.69	2.55	0.27	2.57	2.84	2.36	0.25	2.39	2.64
Jowar	2.42	0.62	2.13	2.75	2.95	0.77	2.39	3.16	2.82	0.80	2.09	2.89
Other Cereals	0.03	0.02	0.02	0.04	0.05	0.01	0.06	0.06	0.07	0.04	0.05	0.09
**Total**	**215.58**	**94.06**	**136.68**	**230.74**	**215.15**	**102.14**	**133.39**	**235.53**	**225.79**	**128.58**	**131.14**	**259.72**
Gram	16.11	12.36	3.36	15.72	18.14	7.3	7.56	14.86	17.65	9.95	6.05	16.00
Urd	2.68	2	1.31	3.31	2.62	1.36	1.36	2.71	2.67	1.79	1.11	2.90
Cowpea	0.36	0.2	0.15	0.35	0.26	0.14	0.15	0.29	0.19	0.14	0.09	0.23
Moong	2.79	1.72	1.58	3.31	2.87	1.63	1.6	3.23	2.68	1.90	1.37	3.27
Horse Gram	0.17	0.07	0.11	0.18	0.24	0.04	0.18	0.22	0.26	0.26	0.10	0.36
Lentil	1.79	0.53	0.85	1.38	1.3	0.35	0.71	1.06	1.47	0.44	0.72	1.16
Lathyrus	0.01	0.01	-	0.01	0	0.01	0	0.01	0.08	0.05	0.03	0.08
Peas	1.96	0.5	1.06	1.57	2.12	0.59	1.24	1.83	2.67	1.41	1.50	2.91
Grain Amaranthus					0	0	0	0				0.00
Moth	0.25	0.07	0.06	0.14	0.21	0.02	0.11	0.13	0.20	0.11	0.10	0.21
Arhar	2.64	1.15	1.63	2.78	2.51	1.11	1.6	2.72	2.71	1.32	1.65	2.97
Rajma	0	0	-	0	0.06	0.03	0.03	0.06	0.02	0.04		0.04
Kehsari	0.06	0.02	0.04	0.06	0.06	0.03	0.03	0.06				0.00
Indian bean	0.07	0	0.07	0.07	0.08	0.01	0.07	0.08	0.21	0.04	0.17	0.21
Rice bean	0	-	0	0	0	0	0	0	0.01	0.01		0.01
Faba bean									0.01	0.01		0.01
Guar									0.81	0.17	0.77	0.94
Other Local Pulses									0.00			0.00
**Total**	**28.88**	**18.63**	**10.24**	**28.87**	**30.49**	**12.61**	**14.63**	**27.24**	**31.64**	**17.64**	**13.66**	**31.30**
Castor	0.72	0.19	0.66	0.85	0.61	0.16	**0.47**	0.62	0.58	0.11	0.73	0.84
R/Mustard	2.64	1.45	1.25	2.7	2.52	1.19	1.46	2.65	2.49	1.24	1.23	2.47
Groundnut	28.47	18.1	11.9	29.99	24.3	(18)	11.72	23.64	23.48	15.78	9.38	25.16
Niger	0.02	0.01	0.01	0.02	0.01	0.01	0	0.01	0.01	0.01	0	0.01
Til	0.31	0.14	0.23	0.37	0.32	0.16	0.21	0.36	0.28	0.22	0.18	0.40
Linseed	0.12	0.01	0.08	0.08	0.08	0.01	0.03	0.04	0.13	0.07	0.04	0.11
Soybean	34.29	10.96	16.37	27.33	31.02	11.4	12.04	23.45	29.00	12.62	17.02	29.64
Sunflower	0.45	0.04	0.42	0.46	0.48	0.04	0.47	0.51	0.39	0.01	0.39	0.40
Safflower	0.12	0.02	0.1	0.12	0.1	0.02	0.09	0.11	0.12	0.02	0.11	0.13
**Total**	**67.13**	**30.91**	**31.02**	**61.93**	**59.45**	**24.9**	**26.49**	**51.39**	**56.48**	**30.08**	**29.08**	**59.16**
Cotton	2.22	0.05	2.57	2.63	1.95	0.22	1.86	2.08	2.22	0.13	2.22	2.35
Jute/Mesta	0.45	0.19	0.42	0.61	0.36	0.11	0.03	0.14	0.43	0.3	0.08	0.38
**Total**	**2.68**	**0.24**	**3**	**3.24**	**2.31**	**0.33**	**1.89**	**2.22**	**2.65**	**0.43**	**2.30**	**2.73**
Potato	27.19	6.74	18.11	24.85	28.64	6.91	19.19	26.1	36.53	7.26	19.82	27.09
Fodders	2.09	0.51	1.63	2.14	1.05	0.39	0.65	1.05	0.37	0.25	0.09	0.34
**Grand Total**	**343.56**	**151.09**	**200.68**	**351.77**	**337.09**	**147.28**	**196.24**	**343.52**	**353.46**	**184.24**	**196.09**	**380.34**

Source: Department of Agriculture, Cooperation & Farmers Welfare

Table 8.3: Crop-wise requirement and availability of Certified/ Quality of Hybrid Seeds

(Thousand Tonnes)

S.No.	Crop	2010-11		2011-12		2012-13		2013-14		2014-15		2015-16		2016-17	
		Req.	Av.	Req.	Av.	Req.	Av.	Req.	Av.	Req.	Av.	Req.	Av.	Req.	Av.
(1)	(2)	(3)	(4)	(5)	(6)	(7)	(8)	(9)	(10)	(11)	(12)	(13)	(14)	(15)	(16)
1	Paddy	9.80	10.90	9.90	9.20	31.60	31.30	42.80	34.20	15.10	19.50	38.50	61.40	19.21	22.09
2	Maize	75.40	92.20	101.70	122.00	97.00	103.40	94.00	96.60	93.50	106.90	101.60	120.50	100.20	125.29
3	Jowar	11.50	13.90	13.00	13.90	13.20	13.90	7.90	10.20	15.60	19.00	17.70	20.00	13.25	19.57
4	Bajra	21.90	26.00	24.60	28.40	24.00	27.10	23.60	33.20	21.00	24.80	24.10	26.60	19.64	24.98
5	Arhar	0.06	0.06	0.02	0.02	0.00	0.01	0.00	0.00	0.09	0.09	0.10	0.00	0.00	0.00
6	Sunflower	5.80	6.30	6.90	9.60	6.40	6.60	4.90	5.40	2.90	3.00	4.40	4.60	6.10	6.26
7	Safflower	0.05	0.00	0.01	0.00	0.00	0.00	0.00	0.00	0.00	0.00	0.00	0.00	0.00	0.00
8	Castor	2.50	3.10	3.37	4.50	4.00	4.30	3.40	4.30	7.10	8.40	6.00	6.10	4.91	5.78
9	Cotton	14.40	15.60	19.50	22.50	22.40	25.40	15.90	17.90	17.90	21.70	18.70	19.80	18.36	19.73
	**Total**	**141.41**	**168.06**	**179.00**	**210.12**	**198.60**	**212.01**	**192.50**	**201.80**	**173.19**	**203.39**	**211.10**	**259.00**	**181.68**	**223.69**

Req.: Requirement Av: Availability

Source: Department of Agriculture, Cooperation & Farmers Welfare

Table 8.4 : Consumption, Production and Import of Fertilisers in terms of Nutrients (N, P & K)

(Lakh Tonnes)

Year	Consumption				Production			Imports				C &F Value of Urea Imports (₹ Crore)
	N	P	K	TOTAL	N	P	TOTAL	N	P	K	TOTAL	
(1)	(2)	(3)	(4)	(5)	(6)	(7)	(8)	(9)	(10)	(11)	(12)	(13)
1981-82	40.69	13.22	6.73	60.64	31.44	9.49	40.93	10.54	3.43	6.44	20.41	716.62
1990-91	79.97	32.21	13.28	125.46	69.93	20.52	90.45	4.14	10.16	13.28	27.58	1335.82
2000-01	109.20	42.15	15.67	167.02	109.61	37.43	147.04	1.54	3.96	15.41	20.91	#
2010-11	165.58	80.50	35.14	281.22	121.57	42.23	163.80	44.92	38.02	40.69	123.63	8348.89
2011-12	173.00	79.14	25.75	277.90	122.59	43.68	166.27	52.40	44.27	33.35	130.02	15442.02
2012-13	168.21	66.53	20.62	255.36	121.94	38.30	160.24	46.90	27.78	12.30	86.98	15980.22
2013-14	167.50	56.33	20.99	244.82	123.78	39.60	163.38	38.08	15.90	13.33	67.31	14987.95
2014-15	169.45	60.98	25.32	255.76	123.94	41.21	165.15	47.66	18.32	25.37	91.35	12035.26
2015-16	173.72	69.79	24.02	267.53	134.16	43.94	178.10	50.68	28.88	20.53	100.09	13984.93
2016-17	167.35	67.05	25.08	259.49	133.54	45.95	179.49	33.88	21.29	23.18	78.35	7024.32**

There was no import of Urea in 2000-01 in Government account.

** Exchange rate is taken as 1 USD = Rs.67.07

Note : 1. Figures relate to imports made on Govt. Account only.

2. The imports made after decanalisation of phosphatic fertilisers (w.e.f. 17.9.1992) and potassic fertilisers (w.e.f. 17.6.1993) include the quantities imported by private parties also.

Source: Department of Fertilizers and Department of Agriculture, Cooperation & Farmers Welfare (DAC&FW)

Chart 8(a): Trends in Consumption, Production and Imports of Fertilizers in India

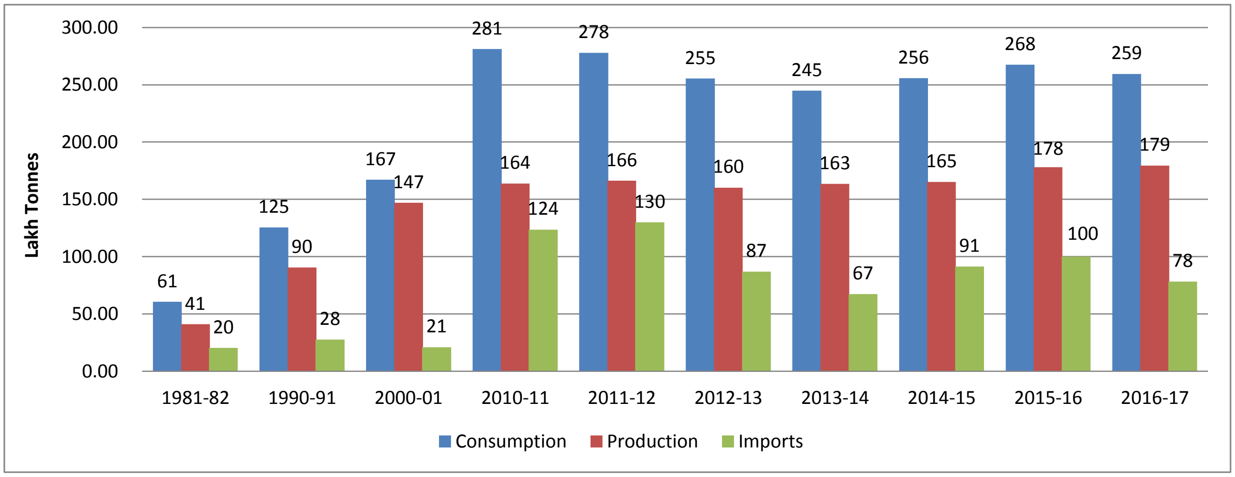

Source: Department of Fertilizers and Department of Agriculture, Cooperation & Farmers Welfare (DAC&FW)

Table 8.5: Zone-wise Consumption of Fertilizers in terms of Nutrients (N, P & K)

(Thousand Tonnes)

S.No.	State/Zone	2010-11	2011-12	2012-13	2013-14	2014-15	2015-16	2016-17
(1)	(2)	(3)	(4)	(5)	(6)	(7)	(8)	(9)
1	South Zone	7150.24	7266.62	5519.08	6004.82	5985.38	6177.58	**5788.00**
		(198.47)	(211.13)	(166.33)	(164.05)	(174.68)	(179.46)	**(166.43)**
2	West Zone	9152.32	8607.24	7636.3	8042.24	8205.58	8382.02	**8426.41**
		(102.46)	(97.84)	(87.33)	(85.93)	(92.61)	(93.85)	**(90.38)**
3	North Zone	7918.70	7922.37	8284.48	7039.97	7634.61	7906.44	**7805.66**
		(183.87)	(181.67)	(191.11)	(162.67)	(173.58)	(174.47)	**(170.18)**
4	East Zone	3588.57	3683.41	3775.01	3078.25	3427.26	4001.47	**3653.88**
		(158.06)	(157.45)	(156.11)	(108.51)	(118.47)	(140.90)	**(125.07)**
5	North East Zone	312.38	310.36	321.28	317.13	323.29	285.09	**275.2**
		(50.39)	(49.75)	(51.42)	(66.48)	(65.97)	(39.01)	**(38.41)**
**6**	**All India**	**28122.21**	**27790.00**	**25536.15**	**24482.41**	**25576.12**	**26752.61**	**25949.15**
		**(142.52)**	**(142.05)**	**(131.36)**	**(118.49)**	**(127.45)**	**(130.66)**	**(123.41)**

Note : Figures in parentheses indicates estimated Consumption of Fertilizers (NPK) per hectare

Source: Department of Agriculture, Cooperation & Farmers Welfare

Table 8.6 (a): State-wise Status of Soil Health Card Scheme Cycle-I as on 20.02.2018

S.No.	States	Cumulative Target for Soil Samples Collection & Testing during Cycle-I (2015-16 & 2016-17)	No. of Samples Collected (Cycle-I)	No. of Samples Tested (Cycle-I)	Cumulative Target for Printing & Distribution of SHCs for Cycle-I (2015-16 & 2016-17)	No. of SHCs Printed (Cycle-I)	No. of SHCs Distributed (Cycle-I)
(1)	(2)	(3)	(4)	(5)	(6)	(7)	(8)
**Group - I**							
1	Uttar Pradesh **	4770399	4770399	4770399	16991000	16581748	16581748
2	Maharashtra	2347121	2347121	2347121	12977232	12977232	12977232
3	Madhya Pradesh *	2313977	2313977	2313977	8872377	8872377	8872377
4	Rajasthan *	2308013	2308013	2308013	6886000	6886000	6886000
**Group - II**							
1	Karnataka *	1665765	1665765	1665765	7832189	7832204	7832204
2	Gujarat *	1589236	1589236	1589236	5108923	5108923	5108923
3	Andhra Pradesh	1348382	1348382	1348382	7455204	7455204	7455204
4	Bihar *	1308778	1308778	1308778	6469650	6469650	6469650
5	West Bengal *	1300349	1300349	1300349	5040510	5040510	4139000
6	Tamil Nadu *	1274536	1274536	1274536	7000000	7000000	7000000
7	Telangana	1034678	1034678	1034678	5720737	5720737	5720737
**Group - III**							
1	Punjab *	835526	835526	482145	1053000	937406	900431
2	Haryana *	788670	788670	788670	4227238	4227238	4227238
3	Chhattisgarh	703691	703691	703691	3890709	3890709	3890709
4	Odisha *	668635	668635	668635	2374233	2374233	2374233
**Group - IV**							
1	Kerala	127585	127585	127585	705420	705420	705420
2	Goa *	25000	25000	25000	25000	25000	25000
3	Uttarakhand	135738	135738	135738	750494	750494	750494
4	Himachal Pradesh	69635	69635	69635	385011	385011	385011
5	J & K *	160687	160687	160687	692062	692062	692062
6	Jharkhand	115302	115302	115302	637507	637507	637507
7	Arunachal Pradesh *	20532	20532	20532	20532	20532	20532
8	Assam	278707	278707	278707	1540968	675334	230027
9	Manipur	20713	20713	20713	114522	114522	114522
10	Meghalaya *	39372	39372	39372	209561	209561	209561
11	Mizoram	11986	11986	11986	11986	11986	11986
12	Nagaland	33423	33423	33423	184797	184797	184797
13	Sikkim *	13217	13217	13217	46000	46000	46000
14	Tripura *	32736	32736	32736	117723	117723	117723
**Union Territories**							
1	Andaman & Nicobar *	1405	1405	1405	10000	10000	10000
2	Dadar Nagar & Haveli *	2162	2162	2162	2162	2162	2162
3	Puducherry	3530	3530	3530	19594	19594	19594
**Total**		**25349486**	**25349486**	**24996105**	**107372341**	**105981876**	**104598084**

* As per changed targets by States ** State that has revised target twice

Source: Department of Agriculture, Cooperation & Farmers Welfare

Table 8.6 (b):State-wise Status of Soil Health Card Scheme Cycle-II as on 20.02.2018

S.No.	States	Target for Soil Samples Collection & Testing during Cycle-II (2017-18)	No. of Samples Collected (Cycle-II)	No. of Samples Tested (Cycle-II)	Target for Printing & Distribution of SHCs for Cycle-II (2017-18)	No. of SHCs Printed (Cycle-II)	No. of SHCs Distributed (Cycle-II)
(1)	(2)	(3)	(4)	(5)	(6)	(7)	(8)
**Group - I**							
1	Uttar Pradesh **	2385200	2108367	1266927	11662730	1074766	467817
2	Maharashtra	1173561	1495437	1239632	6488616	2441389	2079442
3	Madhya Pradesh *	1156989	1071969	802130	4436189	2018400	2017388
4	Rajasthan *	1154007	622000	457000	3443000	1128000	923000
**Group - II**							
1	Karnataka *	832883	679767	544713	3916095	1415867	920802
2	Gujarat *	794618	833561	735828	2554462	0	0
3	Andhra Pradesh	674191	449084	283430	3727602	1011863	1011863
4	Bihar *	654389	569002	282996	3618117	1036844	925446
5	West Bengal *	650175	331000	190000	2520255	806000	0
6	Tamil Nadu *	637268	652245	595070	3500000	769587	628941
7	Telangana	517339	487627	345943	2860369	425529	100063
**Group - III**							
1	Punjab *	417763	339907	0	2309811	0	0
2	Haryana *	394335	622550	165740	2180278	47625	26268
3	Chhattisgarh	351846	468805	416950	1945355	1447665	1380566
4	Odisha *	334318	232146	176621	1848441	395193	333640
**Group - IV**							
1	Kerala	103392	0	0	2852208	0	0
2	Goa *	12500	6757	4453	12500	4223	4223
3	Uttarakhand	67869	67139	63461	375247	217157	198587
4	Himachal Pradesh	50000	56939	54558	480383	328743	328743
5	J & K *	82659	38361	17175	457022	71281	71281
6	Jharkhand	57651	47386	42390	318754	96367	96367
7	Arunachal Pradesh *	10266	0	0	56762	0	0
8	Assam	139354	0	0	770484	0	0
9	Manipur	10357	3542	1465	57261	0	0
10	Meghalaya *	19686	12972	9755	104781	35904	33519
11	Mizoram	5993	227	127	5993	5	5
12	Nagaland	16712	600	500	92399	0	0
13	Sikkim *	6609	0	0	23000	0	0
14	Tripura *	16368	11668	7795	58862	12343	12343
**Union Territories**							
1	Andaman & Nicobar *	703	570	333	3900	0	0
2	Dadar Nagar & Haveli *	1081	0	0	6000	0	0
3	Puducherry	1765	3223	2053	9797	0	0
**Total**		**12731841**	**11212851**	**7707045**	**62696665**	**14784751**	**11560304**

* As per changed targets by States ** State that has revised target twice

Source: Department of Agriculture, Cooperation & Farmers Welfare

Table 8.7: Consumption of Electricity for Agricultural Purposes

Year	Consumption for Agricultural Purposes (GWh)	Total Consumption (GWh)	% Share of Agricultural Consumption to Total Consumption
(1)	(2)	(3)	(4)
1982-83	17817	95589	18.64
1985-86	23422	122999	19.04
1990-91	50321	190357	26.44
2000-01	84729	316600	26.76
2009-10	119492	569618	20.98
2010-11	126377	616969	20.48
2011-12	140960	672933	20.95
2012-13	147462	708843	20.80
2013-14	152744	751908	20.31
2014-15	168913	814250	20.74
2015-16	173185	863364	20.06

Source: Central Electricity Authority, Delhi

Table 8.8: Year-wise Sales of Tractors and Power tillers

Year	Tractor Sales (No.)	Power tiller Sales (No.)
(1)	(2)	(3)
2004-05	247531	17481
2005-06	296080	22303
2006-07	352835	24791
2007-08	346501	26135
2008-09	342836	35294
2009-10	393836	38794
2010-11	545109	55100
2011-12	535210	60000
2012-13	590672	47000
2013-14	696828	56000
2014-15	551463	46000
2015-16	571249	48882
2016-17	582662	45200
**Total**	**6052812**	**522980**

Source: Indian Council of Agricultural Research (ICAR)

Table 8.9: Flow of Institutional Credit to Agriculture Sector

(₹ in Crore)

Particulars/Agency	2007-08	2008-09	2009-10	2010-11	2011-12	2012-13	2013-14*	2014-15*	2015-16*	2016-17*
(1)	(2)	(3)	(4)	(5)	(6)	(7)	(8)	(9)	(10)	(11)
**I. Production (ST) Credit**										
Co-operative Banks	43294	40164	56946	69038	81829	102592	113574	130350	143803	131880
RRBs	21133	22413	29802	38121	47401	55957	70697	89326	101579	105001
Commercial Banks	116966	147818	189908	228391	266928	314951	364164	415736	419930	452576
Other Agencies		66								
**Sub Total (A)**	**181393**	**210461**	**276656**	**335550**	**396158**	**473500**	**548435**	**635412**	**665312**	**689457**
**II. MT/LT Credit**										
Co-operative Banks	4964	5802	6551	9083	6134	8611	6389	8119	9492	10878
RRBs	4179	4352	5415	6172	7049	7724	11956	13157	17681	18215
Commercial Banks	64122	81133	95892	117486	101688	117540	163342	188640	223024	347205
Other Agencies		160								
**Sub Total (B)**	**73265**	**91447**	**107858**	**132741**	**114871**	**133875**	**181687**	**209916**	**250197**	**376298**
**Total Credit (ST + MT/LT)**										
Co-operative Banks	48258	45966	63497	78121	87963	111203	119963	138469	153295	142758
RRBs	25312	26765	35217	44293	54450	63681	82653	102483	119260	123216
Commercial Banks	181088	228951	285800	345877	368616	432491	527506	604376	642954	799781
Other Agencies		226								
**Grand Total (A+B)**	**254658**	**301908**	**384514**	**468291**	**511029**	**607375**	**730122**	**845328**	**915509**	**1065755**

* Figures have been revised based on the updated information from the respective agencies.

ST: Short Term LT:Long Term MT: Medium Term

Source : Department of Agriculture, Cooperation & Farmers Welfare

Table 8.10: State-wise Agriculture Loan disbursed in 2016-17

(No. of accounts in thousands and Amount in ₹ crore)

Sl. No	State/ UT	Crop Loan		Term Loan		Total	
		Disbursment during the year		Disbursment during the year		Disbursment during the year	
		No. of Accounts	Amount disbursed	No. of Accounts	Amount disbursed	No. of Accounts	Amount disbursed
(1)	(2)	(3)	(4)	(5)	(6)	(7)	(8)
1	Andaman & Nicobar	3.11	35.97	12.32	99.01	15.43	134.98
2	Andhra Pradesh	8564.23	61391.48	1987.55	31477.14	10551.78	92868.62
3	Arunachal Pradesh	4.04	27.77	3.93	104.81	7.97	132.59
4	Assam	389.93	1568.20	644.34	4533.88	1034.27	6102.07
5	Bihar	2915.49	14218.62	1591.90	11965.96	4507.38	26184.58
6	Chandigarh	3.97	752.79	3.70	653.16	7.67	1405.95
7	Chhattisgarh	1546.50	9363.50	149.58	2873.92	1696.07	12237.42
8	Dadra & Nagar Haveli	2.37	27.13	2.48	53.04	4.86	80.17
9	Daman & Diu	1.04	12.79	1.07	21.79	2.11	34.58
10	Delhi	49.64	8377.70	27.90	11563.95	77.54	19941.65
11	Goa	21.47	218.85	30.12	792.43	51.58	1011.28
12	Gujarat	2950.02	33864.46	543.99	20412.24	3494.01	54276.70
13	Haryana	2441.83	36274.86	254.53	13206.21	2696.35	49481.07
14	Himachal Pradesh	568.63	4419.53	122.85	1696.61	691.48	6116.15
15	Jammu & Kashmir	618.13	6702.30	28.23	594.44	646.36	7296.74
16	Jharkhand	984.02	2821.40	162.74	1558.59	1146.76	4379.99
17	Karnataka	5236.67	41677.18	1702.71	36405.55	6939.38	78082.72
18	Kerala	5556.74	44415.65	880.63	23323.12	6437.36	67738.76
19	Lakshadweep	0.09	0.55	0.44	2.72	0.53	3.27
20	Madhya Pradesh	6031.36	42909.85	679.27	13239.22	6710.63	56149.06
21	Maharashtra	6131.94	41338.83	2054.08	40045.01	8186.01	81383.84
22	Manipur	13.75	58.99	13.90	192.13	27.66	251.12
23	Meghalaya	36.05	281.70	8.45	86.61	44.50	368.31
24	Mizoram	7.25	30.36	7.36	84.00	14.62	114.36
25	Nagaland	21.30	72.90	4.03	56.49	25.33	129.39
26	Odisha	4303.37	15811.64	541.19	5453.32	4844.57	21264.96
27	Puducherry	455.87	3338.56	161.56	1951.52	617.43	5290.08
28	Punjab	2475.41	58034.30	357.67	16267.16	2833.08	74301.47
29	Rajasthan	6079.00	57875.53	578.72	16428.32	6657.71	74303.86
30	Sikkim	5.03	107.97	4.08	53.72	9.11	161.70
31	Tamil Nadu	11380.28	79593.90	4248.45	52550.67	15628.72	132144.57
32	Telangana	5030.37	47289.22	862.23	20596.13	5892.59	67885.35
33	Tripura	86.81	335.08	220.23	1178.05	307.03	1513.13
34	Uttarakhand	9011.92	59592.66	929.68	21991.35	9941.60	81584.01
35	Uttar Pradesh	431.86	3671.18	97.72	2834.26	529.58	6505.43
36	West Bengal	2700.53	12943.91	2088.81	21951.82	4789.34	34895.72
	**Total**	**86059.98**	**689457.32**	**21008.41**	**376298.35**	**107068.39**	**1065755.67**

Source: Department of Agriculture, Cooperation & Farmers Welfare

Table: 8.11 State-wise and Agency-wise KCCs - Cumulative cards issued and amount Outstanding (as on 31 March 2017)

(Amount in Rs crore and Number in lakh)

S. No.	State/UT	Commercial Banks		Cooperative Banks		RRBs		Total	
		Cumulative cards issued since inception	Amount outstanding	Cumulative cards issued since inception	Amount outstanding	Cumulative cards issued since inception	Amount outstanding	Cumulative cards issued since inception	Amount outstanding
(1)	(2)	(3)	(4)	(5)	(6)	(7)	(8)	(9)	(10)
1	A & N Islands	0.05	2.83	0.09	11.93	0.00	0.00	0.14	14.76
2	Andhra Pradesh	130.23	21295.41	23.55	6862.49	17.23	6574.02	171.01	34731.92
3	Arunachal Pradesh	0.39	54.68	0.01	1.97	0.07	13.21	0.47	69.86
4	Assam	10.77	2746.55	0.04	11.60	6.49	988.56	17.30	3746.71
5	Bihar	42.18	10068.21	2.04	316.40	37.43	8436.18	81.64	18820.79
6	Chandigarh	0.09	494.27					0.09	494.27
7	Chhattisgarh	6.06	3759.53	21.71	1163.95	3.99	1020.56	31.77	5944.04
8	Dadra & Nagar Haveli	0.02	7.61					0.02	7.61
9	Daman & Diu	0.01	3.79					0.01	3.79
10	Delhi	0.37	118.80	0.01	10.45	0.00	0.00	0.38	129.25
11	Goa	0.22	125.03	0.04	16.40	0.00		0.25	141.43
12	Gujarat	25.29	22591.34	21.23	7818.47	6.37	3596.61	52.89	34006.42
13	Haryana	19.97	24964.53	18.50	8707.42	5.05	3458.94	43.53	37130.89
14	Himachal Pradesh	4.54	3294.15	1.33	1191.25	0.87	461.53	6.73	4946.93
15	Jammu & Kashmir	4.97	3423.42	0.15	36.66	1.39	534.32	6.51	3994.40
16	Jharkhand	17.01	2431.82	0.39	34.98	8.10	1231.98	25.50	3698.78
17	Karnataka	52.17	20504.67	37.41	10734.27	16.57	8534.74	106.15	39773.68
18	Kerala	25.02	8570.96	12.21	2830.58	3.38	1203.59	40.61	12605.13
19	Lakshadweep	0.03	2.44					0.03	2.44
20	Madhya Pradesh	37.41	31834.71	81.07	12196.24	11.54	6369.00	130.02	50399.95
21	Maharashtra	74.77	30821.13	63.09	18150.22	8.07	3378.52	145.92	52349.87
22	Manipur	0.41	103.37	0.00	0.03	0.17	22.20	0.58	125.60
23	Meghalaya	1.39	301.75	0.24	31.66	0.44	88.27	2.07	421.68
24	Mizoram	0.37	83.24	0.01	0.00	0.15	91.28	0.53	174.52
25	Nagaland	0.86	158.88	0.06	13.56	0.02	1.70	0.95	174.14
26	Odisha	27.25	3380.06	53.07	7767.97	13.37	2310.37	93.69	13458.40
27	Puducherry	1.39	472.73	0.09	3.33	0.03	10.03	1.51	486.09
28	Punjab	27.98	46685.33	14.82	7231.79	2.92	3872.69	45.72	57789.81
29	Rajasthan	47.73	46145.56	51.44	9787.59	13.14	10979.21	112.31	66912.36
30	Sikkim	0.27	28.53	0.12	10.00	0.00		0.39	38.52
31	Tamil nadu	72.41	9508.38	19.66	4230.63	6.80	1822.88	98.88	15561.89
32	Telangana	77.24	17335.55	15.26	2703.82	26.55	6888.07	119.06	26927.44
33	Tripura	3.70	235.28	1.10	55.76	2.41	150.51	7.21	441.55
34	Uttarakhand	13.90	6223.74	5.25	964.63	1.09	302.50	20.25	7490.87
35	Uttar Pradesh	142.15	61096.56	66.47	5827.83	70.39	27716.17	279.02	94640.56
36	West Bengal	52.26	4664.32	27.87	3479.58	11.47	2359.10	91.60	10503.00
	**Total**	**920.89**	**383539.16**	**538.36**	**112203.46**	**275.49**	**102416.74**	**1734.73**	**598159.36**

Source: Department of Agriculture, Cooperation & Farmers Welfare

Table: 8.12: State-wise coverage under Pradhan Mantri Fasal Bima Yojana (PMFBY) - Cumulative upto Rabi 2016-17

(₹ in lakh)

S.No.	State / UT	No. of Farmers covered	Area Insured (In Ha)	Sum Insured	Farmers' Premium	GoI Premium (Share)	State Govt. Premium	Gross Premium	Claims Reported	Claims Paid	No. of Farmers Benefitted
(1)	(2)	(3)	(4)	(5)	(6)	(7)	(8)	(9)	(10)	(11)	(12)
1	Andhra Pradesh	1005181	785883.28	547931.27	12443.30	19637.87	19637.87	51719.05	33011.12	29249.20	125476.00
2	Andaman & Nicobar Islands	324	253.20	46.84	0.23	0.46	0.93	1.62	14.56	14.56	295.00
3	Arunachal Pradesh	0	0.00	0.00	0.00	0.00	0.00	0.00	0.00	0.00	0.00
4	Assam	8516	4312.95	3011.99	86.28	43.47	43.47	173.22	0.00	0.00	0.00
5	Bihar	2713178	2465249.21	1172428.36	20391.73	60850.73	60850.73	142093.19	32717.98	13015.92	149698.00
6	Chhatisgarh	1542449	2414598.31	721301.74	13554.96	9260.35	9260.35	32075.67	15597.73	15587.40	142088.00
7	Dadra & Nagar Haveli	0	0.00	0.00	0.00	0.00	0.00	0.00	0.00	0.00	0.00
8	Daman & Diu	0	0.00	0.00	0.00	0.00	0.00	0.00	0.00	0.00	0.00
9	Goa	757	547.81	579.83	6.77	0.33	0.33	7.42	2.68	2.68	111.00
10	Gujarat	1975139	2841567.15	1232347.92	24939.21	98789.79	112341.58	236070.58	126193.89	121733.24	678766.00
11	Haryana	1336028	2084575.90	1178213.12	19652.59	6486.39	10202.49	36341.46	29482.05	29007.87	216907.00
12	Himachal Pradesh	260565	85416.19	37135.81	483.05	530.64	530.64	1544.34	569.86	569.86	23050.00
13	Jammu & Kashmir	0	0.00	0.00	0.00	0.00	0.00	0.00	0.00	0.00	0.00
14	Jharkhand	877754	373764.29	200043.32	3963.05	11589.03	11589.03	27141.12	2726.52	2043.47	46008.00
15	Karnataka	2611964	2861980.68	985944.20	21267.15	59626.45	59626.45	140520.04	127254.37	98913.49	718326.00
16	Kerala	18415	12961.11	12827.61	262.22	111.74	111.74	485.70	343.11	0.00	2288.00
17	Lakshadweep	0	0.00	0.00	0.00	0.00	0.00	0.00	0.00	0.00	0.00
18	Madhya Pradesh	6667721	12457496.77	3633475.41	66337.67	161501.99	161501.99	389341.65	178525.66	173146.14	960521.00
19	Maharashtra	11793372	7227810.17	2233694.84	58429.40	168897.21	168897.21	396223.82	191800.33	191800.33	2781403.00
20	Manipur	8366	9120.89	3693.96	73.88	142.69	142.69	359.26	195.91	127.24	8358.00
21	Meghalaya	89	37.71	47.13	1.34	1.36	1.36	4.06	2.62	0.00	48.00
22	Mizoram	0	0.00	0.00	0.00	0.00	0.00	0.00	0.00	0.00	0.00
23	Nagaland	0	0.00	0.00	0.00	0.00	0.00	0.00	0.00	0.00	0.00
24	Orissa	1820236	1318711.78	726234.75	14262.52	19822.72	19822.74	53907.97	43135.18	43127.63	168490.00
25	Puducherry	8537	7978.62	3398.78	26.33	129.14	154.30	309.77	757.21	757.21	4299.00
26	Punjab	0	0.00	0.00	0.00	0.00	0.00	0.00	0.00	0.00	0.00
27	Rajasthan	8983491	10137879.63	1686084.44	35489.08	105329.08	105329.08	246147.24	175776.96	170849.62	2651299.00
28	Sikkim	574	130.50	45.93	0.74	0.14	0.14	1.02	10.77	10.06	225.00
29	Tamil Nadu	1449133	1322585.20	689549.74	29658.72	46995.06	47038.10	123734.93	327111.87	313064.14	1037390.00
30	Telengana	887013	733621.29	463416.28	8559.84	7204.77	7204.77	22969.39	11712.64	11133.29	134278.00
31	Tripura	12528	4916.54	2957.70	28.52	5.19	5.19	38.89	11.40	11.40	542.00
32	Uttar Pradesh	6751422	5819591.51	2542192.07	46427.94	29821.49	29821.49	106070.93	53191.36	53127.61	1107682.00
33	Uttarakhand	208175	107229.41	69722.53	840.86	184.22	184.22	1209.30	376.90	376.59	18135.00
34	West Bengal	4130164	2023047.46	1226977.48	23295.05	23873.67	25407.37	72576.10	37060.66	30589.22	513840.00
	**Total**	**55071091**	**55101268**	**19373303**	**400482**	**830836**	**849706**	**2081068**	**1387583**	**1298258**	**11489523**

Source : Department of Agriculture, Cooperation & Farmers Welfare

Table: 8.13: State-wise coverage under Restructured Weather Based Crop Insurance Scheme (RWBCIS)- Cumulative upto Rabi 2016-17

(₹ in lakh)

S.No.	State / UT	No. of Farmers covered	Area Insured (In Ha)	Sum Insured	Farmers' Premium	GoI Premium (Share)	State Govt. Premium	Gross Premium	Claims Reported	Claims Paid	No. of Farmers Benefitted
(1)	(2)	(3)	(4)	(5)	(6)	(7)	(8)	(9)	(10)	(11)	(12)
1	Andhra Pradesh	766454	766660.41	312978.41	7453.17	10049.42	10049.45	27552.04	57038.00	56952.76	758244
2	Andaman & Nicobar Islands	0	0.00	0.00	0.00	0.00	0.00	0.00	0.00	0.00	0
3	Arunachal Pradesh	0	0.00	0.00	0.00	0.00	0.00	0.00	0.00	0.00	0
4	Assam	51749	36692.13	20547.43	410.96	140.24	140.24	691.45	501.68	501.68	23370
5	Bihar	0	0.00	0.00	0.00	0.00	0.00	0.00	0.00	0.00	0
6	Chhatisgarh	6701	2170.48	1889.43	94.47	203.48	203.48	501.43	135.90	130.76	1106
7	Dadra & Nagar Haveli	0	0.00	0.00	0.00	0.00	0.00	0.00	0.00	0.00	0
8	Daman & Diu	0	0.00	0.00	0.00	0.00	0.00	0.00	0.00	0.00	0
9	Goa	0	0.00	0.00	0.00	0.00	0.00	0.00	0.00	0.00	0
10	Gujarat	0	0.00	0.00	0.00	0.00	0.00	0.00	0.00	0.00	0
11	Haryana	0	0.00	0.00	0.00	0.00	0.00	0.00	0.00	0.00	0
12	Himachal Pradesh	119360	44015.25	52758.04	2626.95	1496.03	1496.03	5619.01	3841.48	3796.54	90083
13	Jammu & Kashmir	0	0.00	0.00	0.00	0.00	0.00	0.00	0.00	0.00	0
14	Jharkhand	0	0.00	0.00	0.00	0.00	0.00	0.00	0.00	0.00	0
15	Karnataka	132026	100650.26	94288.41	4714.39	4879.33	4879.33	14473.05	28092.85	26810.44	148290
16	Kerala	58990	40143.94	20420.07	459.64	1185.79	1185.79	2831.22	1792.23	1787.08	24895
17	Lakshadweep	0	0.00	0.00	0.00	0.00	0.00	0.00	0.00	0.00	0
18	Madhya Pradesh	513595	328852.73	223891.21	10838.09	5668.04	5669.19	22175.33	18603.91	18603.91	341692
19	Maharashtra	207843	210735.63	216144.26	10807.22	33445.68	33445.68	77698.57	38768.89	38170.52	123167
20	Manipur	0	0.00	0.00	0.00	0.00	0.00	0.00	0.00	0.00	0
21	Meghalaya	0	0.00	0.00	0.00	0.00	0.00	0.00	0.00	0.00	0
22	Mizoram	0	0.00	0.00	0.00	0.00	0.00	0.00	0.00	0.00	0
23	Nagaland	0	0.00	0.00	0.00	0.00	0.00	0.00	0.00	0.00	0
24	Orissa	0	0.00	0.00	0.00	0.00	0.00	0.00	0.00	0.00	0
25	Puducherry	0	0.00	0.00	0.00	0.00	0.00	0.00	0.00	0.00	0
26	Punjab	0	0.00	0.00	0.00	0.00	0.00	0.00	0.00	0.00	0
27	Rajasthan	44150	34958.80	11572.19	436.04	2267.29	2267.29	4970.62	5148.14	5148.14	42728
28	Sikkim	0	0.00	0.00	0.00	0.00	0.00	0.00	0.00	0.00	0
29	Tamil Nadu	0	0.00	0.00	0.00	0.00	0.00	0.00	0.00	0.00	0
30	Telengana	88772	98174.53	56294.06	2814.71	1822.93	1822.93	6460.57	6066.73	6066.16	83578
31	Tripura	0	0.00	0.00	0.00	0.00	0.00	0.00	0.00	0.00	0
32	Uttar Pradesh	6250	2814.20	2751.80	137.59	178.87	178.87	495.32	351.14	345.40	6250
33	Uttarakhand	53396	25133.29	22416.96	1115.21	917.01	917.01	2949.22	2370.16	2370.16	43570
34	West Bengal	1713	146.37	357.50	17.88	5.44	5.44	28.76	0.17	0.17	13
	**Total**	**2050999.00**	**1691148.01**	**1036309.78**	**41926.30**	**62259.56**	**62260.73**	**166446.60**	**162711.30**	**160683.71**	**1686986.00**

Source : Department of Agriculture, Cooperation & Farmers Welfare

Table 8.14: Crop-wise Area Insured under all Insurance Schemes

(Area in Lakh Hect.)

S.No.	Crops	2014-15			2015-16			2016-17		
		Gross Area Sown	Area Insured	% of Area Insured	Gross Area Sown*	Area Insured	% of Area Insured	Gross Area Sown*	Area Insured	% of Area Insured
(1)	(2)	(3)	(4)	(5)	(6)	(7)	(8)	(9)	(10)	(11)
1	Paddy	442.38	96.50	21.81	442.38	114.93	25.98	442.38	130.29	29.45
2	Wheat	320.78	78.83	24.57	320.78	87.82	27.38	320.78	100.57	31.35
3	Nutri Cereals	255.01	50.35	19.75	255.01	59.61	23.38	255.01	54.32	21.30
4	Sugarcane	55.65	1.46	2.62	55.65	2.29	4.11	55.65	2.86	5.13
5	Cotton	126.60	15.32	12.10	126.60	14.49	11.44	126.60	35.82	28.29
6	Jute & Mesta	8.19	0.69	8.46	8.19	0.04	0.43	8.19	0.73	8.91
7	Oilseeds	284.24	101.41	35.68	284.24	132.51	46.62	284.24	84.24	29.64
8	Pulses	217.07	65.35	30.10	217.07	71.19	32.80	217.07	58.89	27.13
9	Vegetables	57.87	21.05	36.38	57.87	20.54	35.49	57.87	7.13	12.32
10	Fruits**	41.92	2.27	5.41	41.92	3.97	9.47	41.92	3.51	8.37
11	Others	173.89	8.17	4.70	173.89	16.48	9.48	173.89	89.58	51.52
**Area under all Crops**		**1983.60**	**441.41**	**22.25**	**1983.60**	**523.86**	**26.41**	**1983.60**	**567.92**	**28.63**

* Latest data for Gross area sown is available till 2014-15 only

Source: Department of Agriculture, Cooperation & Farmers Welfare.

Table 8.15: State-wise Crop Area Insured under all Insurance Schemes

(Area in Lakh Hect.)

State/UT	2014-15			2015-16			2016-17		
	Gross Area Sown	Area insured	% of Area Insured	Gross Area Sown*	Area insured	% of Area Insured	Gross Area Sown*	Area insured	% of Area Insured
(1)	(2)	(3)	(4)	(5)	(6)	(7)	(8)	(9)	(10)
Andhra Pradesh	76.90	5.41	7.04	76.90	23.12	30.07	76.90	15.53	20.19
Andaman & Nicobar	0.24	0.01	5.13	0.24	0.01	5.75	0.24	0.00	1.06
Arunachal Pradesh	2.99	0.00	0.00	2.99	0.00	0.00	2.99	0.00	0.00
Assam	40.83	0.30	0.74	40.83	0.29	0.70	40.83	0.41	1.00
Bihar	76.73	37.33	48.65	76.73	28.06	36.57	76.73	24.65	32.13
Chandigarh	0.02	0.00	0.00	0.02	0.00	0.00	0.02	0.00	0.00
Chhattisgarh	57.28	2.06	3.59	57.28	23.43	40.90	57.28	24.17	42.19
Dadar & Nagar Haveli	0.23	0.00	0.00	0.23	0.00	0.00	0.23	0.00	0.00
Daman & Diu	0.03	0.00	0.00	0.03	0.00	0.00	0.03	0.00	0.00
Delhi	0.35	0.00	0.00	0.35	0.00	0.00	0.35	0.00	0.00
Goa	1.58	0.00	0.11	1.58	0.00	0.07	1.58	0.01	0.35
Gujarat	127.73	13.89	10.87	127.73	10.31	8.08	127.73	28.42	22.25
Haryana	65.36	0.00	0.00	65.36	0.00	0.00	65.36	20.85	31.89
Himachal Pradesh	9.18	0.54	5.83	9.18	0.54	5.89	9.18	1.29	14.10
Jammu & Kashmir	11.78	0.01	0.07	11.78	0.00	0.00	11.78	0.00	0.00
Jharkhand	15.54	2.50	16.08	15.54	4.17	26.83	15.54	3.74	24.05
Karnataka	122.47	14.07	11.49	122.47	17.18	14.03	122.47	29.63	24.19
Kerala	26.25	0.48	1.85	26.25	0.65	2.46	26.25	0.53	2.02
Lakshdweep	0.03	0.00	0.00	0.03	0.00	0.00	0.03	0.00	0.00
Madhya Pradesh	238.10	108.03	45.37	238.10	121.08	50.85	238.10	127.86	53.70
Maharashtra	234.74	48.63	20.72	234.74	83.00	35.36	234.74	74.39	31.69
Manipur	3.83	0.06	1.70	3.83	0.17	4.39	3.83	0.09	2.38
Meghalaya	3.43	0.01	0.41	3.43	0.01	0.29	3.43	0.00	0.01
Mizoram	1.45	0.00	0.00	1.45	0.00	0.00	1.45	0.00	0.00
Nagaland	5.00	0.00	0.00	5.00	0.00	0.00	5.00	0.00	0.00
Odisha	51.73	0.00	0.00	51.73	0.00	0.00	51.73	13.19	25.49
Puducherry	0.27	0.00	0.00	0.27	0.00	0.00	0.27	0.08	29.55
Punjab	78.57	0.00	0.00	78.57	0.00	0.00	78.57	0.00	0.00
Rajasthan	242.35	119.78	49.42	242.35	114.55	47.27	242.35	101.73	41.98
Sikkim	1.36	0.00	0.01	1.36	0.00	0.08	1.36	0.00	0.10
Tamil Nadu	59.95	8.69	14.49	59.95	12.45	20.77	59.95	13.23	22.06
Telangana	53.15	0.00	-	53.15	0.00	0.00	53.15	8.32	15.65
Tripura	4.83	0.00	0.09	4.83	0.01	0.21	4.83	0.05	1.02
Uttar Pradesh	261.47	20.43	7.81	261.47	37.98	14.53	261.47	58.22	22.27
Uttarakhand	10.97	0.51	4.67	10.97	1.11	10.12	10.97	1.32	12.07
West Bengal	96.90	9.97	10.29	96.90	8.73	9.01	96.90	20.23	20.88
**All IndiaTotal**	**1983.60**	**392.72**	**19.80**	**1983.60**	**486.86**	**24.54**	**1983.60**	**567.92**	**28.63**

* Latest data for Gross area sown is available till 2014-15 only

Source : Department of Agriculture, Cooperation & Farmers Welfare

Table 8.16: State-wise Area covered under Micro Irrigation as on 31.03.2017

(Hectares)

S.No.	Name of State	Drip	Sprinkler	Total
(1)	(2)	(3)	(4)	(5)
1	Andhra Pradesh	1012093	386415	1398508
2	Arunachal Pradesh	613	0	613
3	Assam	310	129	439
4	Bihar	10309	101124	111433
5	Chattisgarh	20399	263857	284256
6	Goa	1086	993	2079
7	Gujarat	557606	580396	1138002
8	Haryana	28081	556079	584160
9	Himachal Pradesh	4441	3652	8093
10	Jammu & Kashmir	23	57	80
11	Jharkhand	16641	14227	30868
12	Karnataka	514090	536443	1050533
13	Kerala	22890	8080	30970
14	Madhya Pradesh	265194	216470	481664
15	Maharashtra	1004175	408365	1412540
16	Manipur	288	30	318
17	Meghalaya	308	307	615
18	Mizoram	3064	1364	4428
19	Nagaland	444	5005	5449
20	Odisha	22575	87038	109613
21	Punjab	35208	12473	47681
22	Rajasthan	212406	1576139	1788545
23	Sikkim	6044	3042	9086
24	Tamil Nadu	352375	45136	397511
25	Telangana	107828	24608	132436
26	Tripura	444	1651	2095
27	Uttar Pradesh	20118	50674	70792
28	Uttrakhand	3622	2059	5681
29	West Bengal	604	50576	51180
30	Others	15169	30636	45805
	**Total**	**4238448**	**4967025**	**9205473**

Source : Department of Agriculture, Cooperation & Farmers Welfare

Table 8.17: State wise Cold Storage capacity (31.03.2017)

S.No.	State/UT	No. of Project	Capacity (MT)
(1)	(2)	(3)	(4)
1	Andaman & Nicobar	2	210
2	Andhra Pradesh & Telangana	432	1757785
3	Arunachal Pradesh	1	5000
4	Assam	36	157906
5	Bihar	305	1416095
6	Chandigarh	7	12462
7	Chhattisgarh	98	484557
8	Delhi	97	129857
9	Goa	29	7705
10	Gujarat	753	2875713
11	Haryana	336	741446
12	Himachal Pradesh	63	119167
13	Jammu & Kashmir	36	112206
14	Jharkhand	58	236680
15	Karnataka	194	553401
16	Kerala	196	78105
17	Lakshadweep	1	15
18	Madhya Pradesh	294	1253715
19	Maharashtra	581	896730
20	Manipur	1	3000
21	Meghalaya	4	8200
22	Mizoram	3	4471
23	Nagaland	2	6150
24	Odisha	170	538139
25	Puducherry	3	85
26	Punjab	655	2152003
27	Rajasthan	161	527893
28	Sikkim	2	2100
29	Tamil Nadu	168	316583
30	Tripura	14	45477
31	Uttar Pradesh	2285	14139098
32	Uttrakhand	45	151421
33	West Bengal	511	5940511
	**All India**	**7543**	**34673886**

Source: Data upto 2009-10 is from Directorate of Marketing and Inspection (DMI) and National Horticulture Board (NHB), National Horticulture Mission (NHM) & Ministry of Food Processing Industries(MoFPI) for later years.

Table 8.18: Cost Estimates of Principal Crops- 2014-15

Crop	State	Cost of Cultivation (₹/Hectare)		Cost of Production (₹/Qtl.)	Yield (Qtl./Hec.)
		A2+FL	C2	C2	
(1)	(2)	(3)	(4)	(5)	(6)
**Paddy**	Andhra Pradesh	52092.45	78968.33	1282.17	58.33
	Assam	37414.70	49886.91	1427.84	32.45
	Bihar	26862.34	40096.73	1054.94	30.69
	Chhattisgarh	31680.98	45775.07	1229.85	33.76
	Gujarat	43303.53	55798.06	1095.87	42.33
	Haryana	46032.95	78947.58	1531.08	50.53
	Himachal Pradesh	26893.42	37070.51	1099.80	25.09
	Jharkhand	24997.56	38847.53	1083.41	28.35
	Karnataka	48704.20	68315.20	1195.71	53.24
	Kerala	53391.99	71971.60	1479.24	43.64
	Madhya Pradesh	29281.08	41380.77	1416.12	25.44
	Maharashtra	55262.14	68262.45	2237.11	25.64
	Orissa	43151.95	56913.99	1405.45	36.62
	Punjab	39631.03	73254.48	1091.75	66.74
	Tamil Nadu	55778.57	74076.91	1397.70	49.49
	Uttar Pradesh	40977.39	58982.33	1479.86	37.08
	Uttarakhand	30735.11	45634.39	896.47	47.76
	West Bengal	55746.71	71840.42	1411.34	44.69
**Wheat**	Bihar	27472.02	41449.06	1230.82	26.52
	Gujarat	32999.68	42296.08	1239.07	31.39
	Haryana	33649.03	61318.70	1240.99	39.96
	Himachal Pradesh	22866.27	34047.93	1750.37	14.32
	Jharkhand	20858.74	28809.65	1734.07	13.86
	Karnataka	21188.53	27278.85	2354.04	10.16
	Madhya Pradesh	26220.56	43954.60	1127.43	32.73
	Maharashtra	34275.35	45942.98	1992.76	22.44
	Punjab	27772.52	52590.47	1081.84	42.88
	Rajasthan	35872.93	50089.47	1156.25	34.58
	Uttar Pradesh	32744.41	51080.58	1502.89	25.88
	Uttarakhand	22637.88	38400.52	1277.32	24.24
	West Bengal	40418.69	52547.92	1585.96	30.82
**Maize**	Andhra Pradesh	41509.60	65916.32	1130.40	55.67
	Bihar	30413.80	43478.29	1138.29	31.96
	Gujarat	35859.96	43289.81	1931.00	16.04
	Himachal Pradesh	22425.48	30457.83	1673.25	13.07
	Jharkhand	32602.11	39886.92	1007.96	33.02
	Karnataka	30760.69	41402.31	1273.66	29.57
	Madhya Pradesh	24803.22	34791.77	1262.09	22.91
	Maharashtra	59230.95	74156.68	1993.66	32.70
	Odisha	39792.25	51302.50	1336.26	37.50
	Punjab	38426.75	51749.20	1154.79	38.21
	Rajasthan	33710.97	45020.96	1628.96	21.51
	Tamil Nadu	60448.07	80010.51	1253.92	60.41
	Uttar Pradesh	20482.40	28770.46	1888.58	12.73

contd.

Crop	State	Cost of Cultivation (₹/Hectare)		Cost of Production (₹/Qtl.)	Yield (Qtl./Hec.)
		A2+FL	C2	C2	
(1)	(2)	(3)	(4)	(5)	(6)
**Jowar**	Andhra Pradesh	23309.78	37177.30	1732.18	16.16
	Karnataka	17515.09	24811.88	2266.91	9.06
	Madhya Pradesh	23800.24	28541.25	2710.85	8.16
	Maharashtra	32734.56	44764.79	1951.96	13.78
	Rajasthan	17854.82	25352.68	1537.81	7.82
	Tamil Nadu	20763.49	30604.77	1678.65	8.88
**Bajra**	Gujarat	35920.62	46812.70	1125.22	27.86
	Haryana	26208.19	37615.55	1487.42	17.71
	Maharashtra	35622.93	43373.26	2749.83	13.22
	Rajasthan	17888.42	24548.14	1149.16	10.77
	Uttar Pradesh	21824.39	33693.02	1038.43	24.33
**Barley**	Rajasthan	34436.27	45200.62	1002.76	32.16
	Uttar Pradesh	24638.13	41970.24	1450.72	21.70
**Ragi**	Karnataka	43421.41	54317.49	3240.98	12.26
	Uttarakhand	14948.91	19826.99	905.79	18.76
**Arhar**	Andhra Pradesh	25539.03	36458.55	6381.70	5.54
	Gujarat	32507.72	41891.52	4550.86	8.87
	Karnataka	22525.78	33585.73	3963.24	8.27
	Madhya Pradesh	20302.81	33433.45	4568.56	6.84
	Maharashtra	42313.15	59401.50	5715.19	10.10
	Orissa	13008.82	20897.95	6806.19	3.00
	Uttar Pradesh	21950.62	40712.59	4184.48	7.92
**Gram**	Andhra Pradesh	32334.44	47413.80	3085.18	15.02
	Bihar	19029.27	33262.94	2389.66	13.49
	Chhattisgarh	15971.53	23683.24	3101.72	7.34
	Haryana	17993.23	32706.69	3313.40	9.17
	Jharkhand	13486.73	17706.39	1631.31	10.38
	Karnataka	20886.46	31942.05	2919.60	10.73
	Madhya Pradesh	21376.69	33952.35	2911.56	11.00
	Maharashtra	26147.88	37788.79	3401.88	10.73
	Rajasthan	16872.39	24002.81	3440.21	6.40
	Uttar Pradesh	22456.03	32529.72	5507.26	5.39
**Urad**	Andhra Pradesh	14608.64	33527.11	3394.44	9.83
	Chhattisgarh	23655.42	31650.43	3394.59	8.69
	Madhya Pradesh	17185.26	25638.75	3482.43	7.05
	Maharashtra	21192.57	28123.66	6107.01	4.54
	Orissa	14857.29	21957.61	5574.98	3.79
	Tamil Nadu	18007.38	27987.31	4446.77	6.19
	Uttar Pradesh	14438.71	22741.98	4756.45	4.67
**Moong**	Andhra Pradesh	15185.13	25132.57	4655.63	5.37
	Gujarat	20823.73	23778.18	11264.37	1.99
	Karnataka	15987.69	23174.39	5790.59	3.95
	Maharashtra	24601.39	32001.87	8098.91	3.92
	Orissa	13274.41	19257.07	5618.72	3.29
	Rajasthan	14806.99	20624.38	5267.63	3.48
**Lentil**	Bihar	16457.57	30105.16	2399.78	11.98
	Madhya Pradesh	19037.02	31121.42	3575.36	8.34
	Uttar Pradesh	18572.54	30043.84	6797.93	4.20
	West Bengal	22327.40	38680.61	2518.08	14.98

contd.

Crop	State	Cost of Cultivation (₹/Hectare)		Cost of Production (₹/Qtl.)	Yield (Qtl./Hec.)
		A2+FL	C2	C2	
(1)	(2)	(3)	(4)	(5)	(6)
**Groundnut**	Andhra Pradesh	36939.57	53753.88	4607.64	10.79
	Gujarat	54841.87	70827.74	3409.54	17.07
	Karnataka	37367.82	49950.00	4541.52	10.27
	Maharashtra	44470.14	57079.41	5783.84	8.87
	Orissa	41989.55	59255.51	4516.02	12.82
	Rajasthan	29477.47	38686.20	3777.03	9.72
	Tamil Nadu	57324.24	75449.68	3571.59	19.65
**R & M**	Assam	27502.48	35801.81	4353.30	8.22
	Bihar	16038.56	28849.77	2307.82	11.83
	Gujarat	26250.95	39449.70	2068.79	18.41
	Haryana	24941.42	47679.26	2930.17	14.79
	Madhya Pradesh	18674.03	34489.10	2216.82	14.64
	Rajasthan	23925.44	34818.04	2610.63	12.44
	Uttar Pradesh	25621.24	41477.32	3849.39	9.98
	West Bengal	32240.39	45335.11	3113.76	14.21
**Soy Bean**	Andhra Pradesh	28024.29	40321.28	3854.21	10.36
	Chhattisgarh	15940.30	23946.41	2510.75	9.19
	Madhya Pradesh	23971.63	34985.38	2661.81	12.53
	Maharashtra	36054.83	45160.23	4521.97	9.72
	Rajasthan	23017.67	29402.36	3916.13	6.96
**Sunflower**	Andhra Pradesh	23847.41	32370.95	7923.19	4.06
	Karnataka	19913.10	28340.18	3159.53	8.83
**Safflower**	Karnataka	10248.46	18231.29	2113.43	8.39
	Maharashtra	18491.84	22335.92	6972.54	3.19
**Nigerseed**	Madhya Pradesh	13824.32	17978.15	5697.21	3.00
	Orissa	12341.82	16023.77	6302.23	2.48
**Sesamum**	Andhra Pradesh	19282.00	29638.95	7177.67	4.05
	Gujarat	29055.91	39359.26	6903.85	5.64
	Madhya Pradesh	16725.67	29141.57	5289.28	5.38
	Orissa	16560.41	24694.29	5701.60	4.28
	Rajasthan	13848.45	26346.97	7863.26	3.30
	Uttar Pradesh	10461.47	16090.93	6973.32	2.25
	West Bengal	29269.19	41501.10	3375.65	11.88
**Sugarcane**	Andhra Pradesh	108236.30	163567.80	218.45	730.90
	Karnataka	73659.48	118220.50	143.45	808.81
	Maharashtra	145489.58	193364.50	182.74	993.77
	Tamil Nadu	124126.89	160892.00	171.05	926.39
	Uttar Pradesh	54449.19	97917.09	173.25	539.07
	Uttarakhand	45667.40	92069.24	148.79	578.17
**Cotton**	Andhra Pradesh	56730.97	79837.62	4661.68	17.08
	Gujarat	57070.13	73271.72	3659.32	19.67
	Haryana	45095.36	64904.91	5573.05	10.85
	Karnataka	42891.68	59042.24	4084.52	14.02
	Madhya Pradesh	59274.65	76832.02	5564.46	12.82
	Maharashtra	54887.56	72391.18	4631.87	15.31
	Orissa	35654.78	44347.32	6348.29	6.82
	Punjab	52923.06	76777.61	3890.58	18.39
	Rajasthan	49320.05	67922.05	3821.91	16.73
	Tamil Nadu	69912.63	88992.70	3770.22	23.51

contd.

Crop	State	Cost of Cultivation (₹/Hectare)		Cost of Production (₹/Qtl.)	Yield (Qtl./Hec.)
		A2+FL	C2	C2	
(1)	(2)	(3)	(4)	(5)	(6)
**Jute**	Assam	55065.78	68361.55	2627.43	24.51
	Bihar	18814.86	29738.61	2415.46	11.77
	Orissa	52753.86	66408.93	3495.91	16.95
	West Bengal	58351.73	80286.87	2655.57	27.29
**Onion**	Gujarat	92472.64	125716.20	852.24	146.81
	Karnataka	41828.75	54731.59	1210.34	44.32
	Maharashtra	120342.20	171271.10	754.14	225.89
**Potato**	Assam	101036.96	124987.60	1117.94	111.80
	Bihar	60663.42	79846.72	448.18	178.16
	Himachal Pradesh	86109.88	110717.80	993.84	111.40
	Uttar Pradesh	105856.91	134770.90	712.73	189.09
	West Bengal	123377.36	146702.30	565.97	259.21

Source: Directorate of Economics and Statistics, DAC&FW

Note: 1. Cost A2 includes all actual expenses in cash & kind incurred in production by Owner and rent paid for leased-in-land.

2 Cost A2 includes all actual expenses in cash & kind incurred in production by owner, interest on value of owned fixed capital assets(excluding land), rental value of owned land(net of land revenue) and imputed value of family labour.

3 Family Labour is calculated on the basis of statuory wage rate or the actual market rate, whichever is higher.

Table 8.19: All-India projected Cost of Production (A2+FL) of Major Crops in India

(₹ per quintal)

Sl. No.	Commodity	2013-14	2014-15	2015-16	2016-17	2017-18
(1)	(2)	(3)	(4)	(5)	(6)	(7)
	**KHARIF CROPS***					
1	PADDY	961	978	1020	1045	1117
2	JOWAR	1269	1370	1467	1501	1556
3	BAJRA	768	832	893	925	949
4	MAIZE	860	914	941	966	1044
5	RAGI	1338	1474	1688	1733	1861
6	ARHAR(Tur)	3090	3105	3237	3241	3318
7	MOONG	3775	3890	3993	4065	4286
8	URAD	3144	3225	3455	3584	3265
9	COTTON	2485	2510	2753	2889	3276
10	GROUNDNUT IN SHELL	2720	3232	3314	3371	3159
11	SUNFLOWER SEED	1692	1729	1770	1852	3481
12	SoyBEEN (BLACK)	3000	3129	3282	3479	2121
13	SESAMUM	2919	3765	4132	4188	4067
14	NIGERSEED	2279	3084	3146	3366	3912
	**RABI CROPS****					
1	WHEAT	687	679	744	785	797
2	BARLEY	620	676	735	776	816
3	GRAM	1926	1786	1902	2124	2241
4	MASUR (LENTIL)	1696	1799	1866	2015	2174
5	RAPESEED/MUSTARD	1260	1307	1504	1702	1871
6	SAFFLOWER	2754	2558	3025	3057	3049
	**OTHER CROPS*****					
1	JUTE	1015[@]	1702	2042	2125	2160
2	SUGARCANE	120	123	140	140[#]	145
3	COPRA	3705	4398^	4138	4676	4758

Source: Price Policy Reports of Commission for Agricultural Costs and Prices (CACP).

* Figures are for Kharif Marketing Season of the respective year

** For Rabi Marketing Season of the respective year *** For Marketing Season of respective year

@ : The figure consists of only A2 and not FL

#Unadjusted for 9.5% Recovery of Sugarcane for 2016-17 Sugar Season

^The figure for this year is only available for Kerala

Table 8.20: All India Annual Average Daily Wage Rate

Crop Year	Agri. Wages (in Rupees)	
	Male	Female
(1)	(2)	(3)
2006-07	82	62
2007-08	91	70
2008-09	108	82
2009-10	124	95
2010-11	149	115
2011-12	183	134
2012-13	214	158
2013-14	229	178
2014-15	268	200
2015-16	281	218

Source: Directorate of Economics & Statsitics

Note 1. All India annual average is calculated for 20 major States.

2. Average Agricultural Wages is taken as average of five operations.

Chart 8 (b): Trends in Average Daily Wage Rate of Male & Female Workers in Agricultural Operations

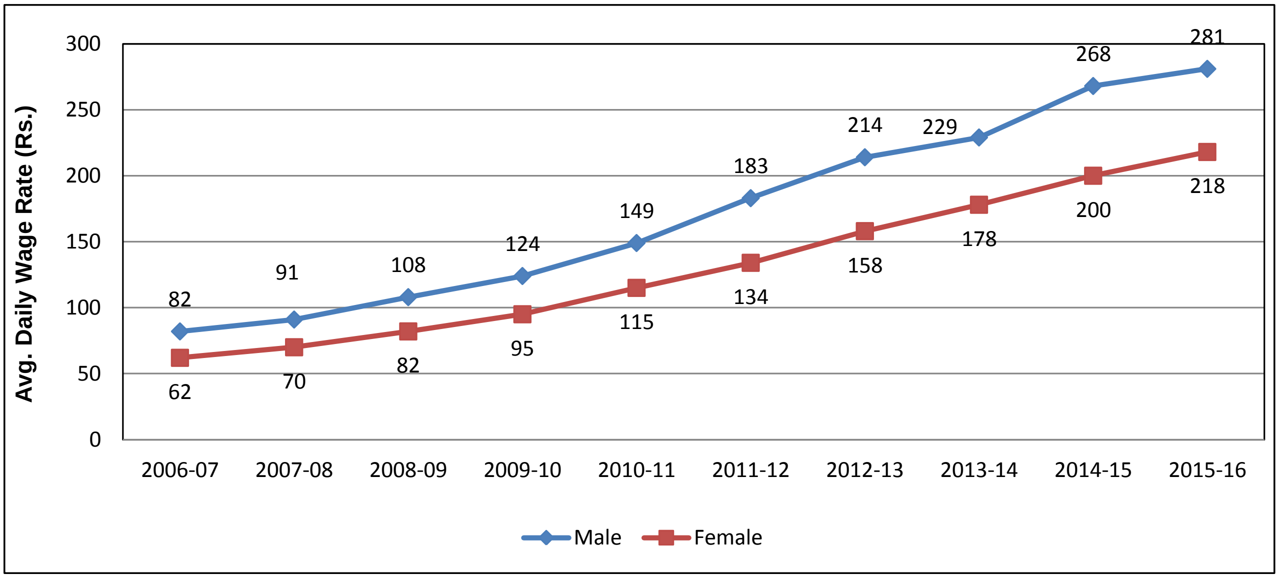

Source: Directorate of Economics & Statsitics

PRICES
&
PROCUREMENT

Table 9.1 : Minimum Support Prices of various Agricultural Commodities (According to Crop Year)

(As on 24.10.2017)

(₹ per quintal)

S. No.	Commodity	Variety	2013-14	2014-15	2015-16	2016-17	2017-18
(1)	(2)	(3)	(4)	(5)	(6)	(7)	(8)
	**Kharif Crops**						
1	Paddy	Common	1310	1360	1410	1470	1550
		Grade 'A'	1345	1400	1450	1510	1590
2	Jowar	Hybrid	1500	1530	1570	1625	1700
		Maldandi	1520	1550	1590	1650	1725
3	Bajra		1250	1250	1275	1330	1425
4	Maize		1310	1310	1325	1365	1425
5	Ragi		1500	1550	1650	1725	1900
6	Arhar(Tur)		4300	4350	4625^	5050^^	5450^
7	Moong		4500	4600	4850^	5225^^	5575^
8	Urad		4300	4350	4625^	5000^^	5400^
9	Cotton	Medium Staple	3700	3750	3800	3860	4020
		Long Staple	4000	4050	4100	4160	4320
10	Groundnut In Shell		4000	4000	4030	4220*	4450^
11	Sunflower Seed		3700	3750	3800	3950*	4100*
12	Soybeen		2560	2560	2600	2775*	3050^
13	Sesamum		4500	4600	4700	5000^	5300*
14	Nigerseed		3500	3600	3650	3825*	4050*
	**Rabi Crops**						
15	Wheat		1400	1450	1525	1625	1735
16	Barley		1100	1150	1225	1325	1410
17	Gram		3100	3175	3500**	4000^	4400@
18	Masur (Lentil)		2950	3075	3400**	3950@	4250*
19	Rapeseed/Mustard		3050	3100	3350	3700*	4000*
20	Safflower		3000	3050	3300	3700*	4100*
21	Toria		3020	3020	3290	3560	
	**Other Crops**						
22	Copra (Calendar Year)	Milling	5250	5250	5550	5950	6500
		Ball	5500	5500	5830	6240	6785
23	De-Husked Coconut (Calendar Year)		1425	1425	1500	1600	1760
24	Jute		2300	2400	2700	3200	3500
25	Sugarcane$		210	220	230	230	255

Figures in brackets indicate percentage increase.

$ Fair and remunerative price.

* Including Bonus of Rs. 100 per quintal.

** Including Bonus of Rs. 75 per quintal .

^ Including Bonus of Rs. 200 per quintal.

^^ Including Bonus of Rs. 425 per quintal.

@ Including Bonus of Rs. 150 per quintal

Source: Directorate of Economics & Statistics, DAC&FW

Table 9.2: State-wise Procurement of Rice and Wheat in Major Rice and Wheat Producing States

('000 Tonnes)

State	2011-12	2012-13	2013-14	2014-15	2015-16	2016-17	2017-18*
(1)	(2)	(3)	(4)	(5)	(6)	(7)	(8)
**Rice (Oct-Sept)**							
Punjab	7731	8558	8106	7786	93.5	11052	
Haryana	2007	2609	2406	2015	2861	3583	
Uttar Pradesh	3355	2286	1127	1698	2910	2354	
Andhra Pradesh	7540	6464	3737	3596	4336	3724	
Telangana	-	-	4353	3504	158	3595	
Madhya Pradesh	635	898	1045	807	849	1314	
Odisha	2864	3613	2801	3357	3369	3630	
Tamil Nadu	1596	481	684	1051	1192	144	
West Bengal	2036	1766	1359	2032	1568	1923	
Chhattisgarh	4115	4804	4290	3423	3442	4022	
Uttarakhand	378	497	463	465	598	706	
Others	2784	2068	1474	2603	2162	2058	
**All-India**	**35041**	**34044**	**31845**	**32040**	**34217**	**38105**	
**Wheat (April-March)**							
Punjab	10958	12834	10897	11641	10344	10649	11706
Uttar Pradesh	3461	5063	682	628	2267	797	3699
Haryana	6928	8665	5873	6495	6778	6752	7432
Rajasthan	1303	1964	1268	2159	1300	762	1245
Chhattisgarh	-	-	9	-			
Uttarakhand	42	139	5	1	4	2	2
Bihar	557	772	0	0	0	**-**	
Chandigarh	7	17	8	5	11	**7**	8
Delhi	8	31	Neg.	0	2	-	
Gujarat	105	156	0	0	73	-	7
Himachal Pradesh	1	1	Neg.	Neg.	0	-	
Jammu & Kashmir	-	9	0	0	0	-	
Madhya Pradesh	4965	8493	6355	7094	7309	3992	6725
Maharashtra	-	2	0	0	0	-	
Jharkhand	-	-	0	0	0	-	
West Bengal	-	2	2	0	0	-	
**All-India**	**28335**	**38148**	**25092**	**28023**	**28088**	**22962**	**30824**

* Position as on 21.09.2017 and prior to Kharif 2017-18 harvesting

Neg : Below 500 tonnes.

Source : Department of Food and Public Distribution.

Table 9.3: Trends in Wholesale Price Index of Commercial Crops (Base: 2004-05=100)

Commodity	Weight	2011-12	2012-13	2013-14	2014-15	2015-16	2016-17
(1)	(2)	(3)	(4)	(5)	(6)	(7)	(8)
**A. Oilseeds**	**1.78**	**158.8**	**198.0**	**202.6**	**208.9**	**214.9**	**217.3**
Groundnut Seed	0.40	200.0	246.9	219.1	210.3	246.2	264.9
Rape & Mustard Seed	0.34	149.8	202.7	189.3	193.9	225.0	231.0
Cotton Seed	0.21	143.4	162.1	177.6	172.8	196.7	227.7
Copra (Coconut)	0.25	112.9	92.4	117.4	186.2	147.4	124.9
Gingelly Seed (Sesamum)	0.07	203.8	312.4	417.5	425.5	312.5	312.4
Niger Seed	0.01	167.1	192.3	174.2	199.1	334.3	325.2
Safflower (Kardi Seed)	0.01	138.4	149.5	158.0	136.0	151.9	160.1
Sunflower	0.08	162.7	181.7	193.8	183.3	194.5	180.4
Soy Bean	0.37	140.8	211.9	226.3	216.5	210.0	196.4
A1 Edible Oils	3.04	135.7	148.1	147.0	145.0	148.6	156.0
Gingelly Oil	0.05	146.4	171.3	183.1	178.5	165.3	185.0
Mustard & Rapeseed Oil	0.45	135.9	154.2	154.0	157.5	180.4	182.5
Copra Oil	0.10	119.9	114.4	121.2	139.8	149.0	140.3
Groundnut Oil	0.30	163.8	192.6	181.6	167.3	192.3	214.4
Cotton Seed Oil	0.26	149.5	175.5	176.8	175.7	184.9	198.2
Sunflower Oil	0.17	132.0	138.0	131.8	124.9	131.1	133.5
Soy Bean Oil	0.38	147.2	162.2	159.2	153.5	148.8	156.7
**B. Fruits and Vegetables**	**3.84**	**183.2**	**198.4**	**244.3**	**257.3**	**254.0**	**255.2**
B1. Vegetables	1.74	179.3	210.1	294.5	276.6	268.5	252.0
Potato	0.20	129.0	207.0	220.1	297.2	164.1	227.1
Onion	0.18	186.7	232.5	482.2	333.7	447.3	247.1
B2. Fruits	2.11	186.4	188.8	202.9	241.5	242.0	257.8
Banana	0.34	173.4	210.5	246.6	288.2	265.5	298.9
Apple	0.10	220.4	241.4	228.6	240.1	234.5	271.8
Orange	0.13	237.4	228.3	208.9	237.7	219.6	234.3
**C. Condiments & Spices**	**0.57**	**237.5**	**209.5**	**245.6**	**298.8**	**342.6**	**348.1**
Black Pepper	0.03	402.9	519.0	553.2	736.7	724.9	739.2
Chillies(Dry)	0.16	277.1	235.1	267.0	296.7	358.6	392.1
Turmeric	0.08	214.9	166.3	215.3	229.6	255.6	246.1
**Raw Cotton**	**0.70**	**225.2**	**206.0**	**236.5**	**206.1**	**189.9**	224.2
**Raw Jute**	**0.06**	**222.5**	**242.4**	**261.9**	**288.1**	**403.5**	**447.9**

Source : Office of the Economic Adviser, Ministry of Commerce & Industry.

Chart 9(a): Month-wise trend in Food Inflation under WPI and CPI

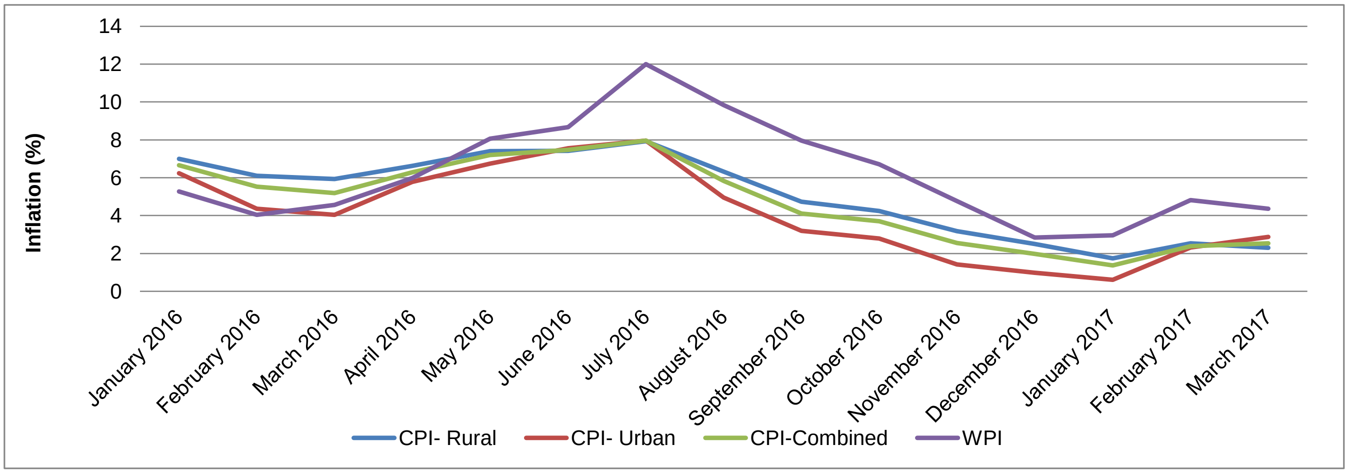

Note: CPI is based on new series with Base 2012=100 and WPI with Base 2004-05=100

Source: Ministry of Commerce & Industry and CSO

Chart 9(b): Trends in Inflation (WPI) of Food Products, Food Articles and All Commodities

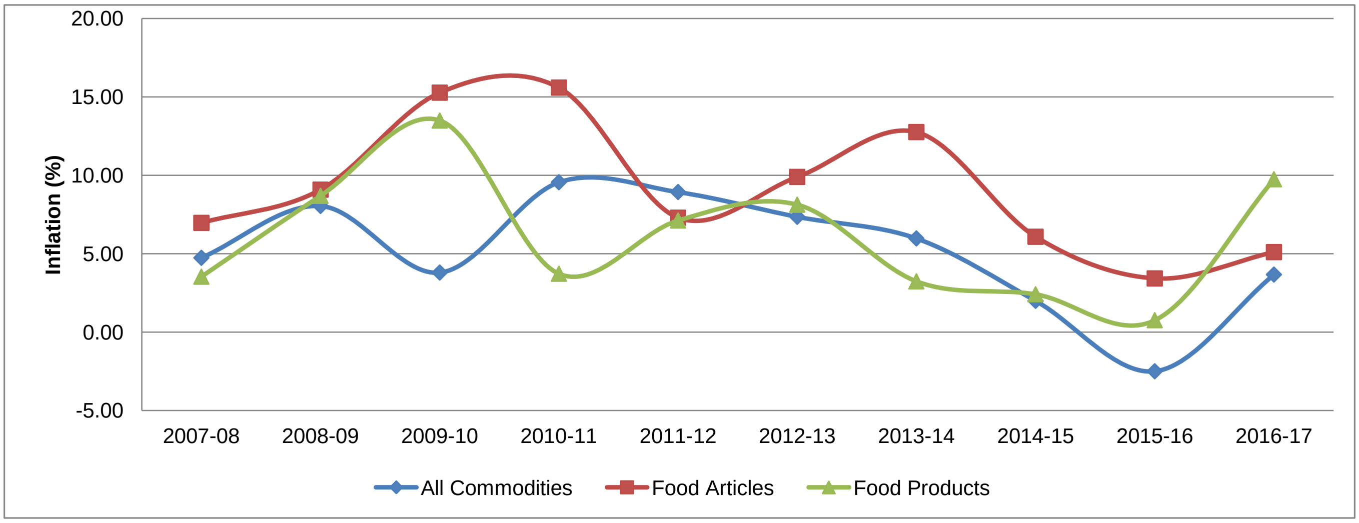

Source: Ministry of Commerce & Industry and CSO

PER CAPITA AVAILABILITY & EXPENDITURE

Table 10.1 Per Capita Net Availability of Foodgrains (Per Annum) in India

(Kgs Per Year)

Year	Rice	Wheat	Other Cereals	Cereals	Gram	Pulses	Food Grains
(1)	(2)	(3)	(4)	(5)	(6)	(7)	(8)
1951	58.0	24.0	40.0	122.0	8.2	22.1	144.1
1961	73.4	28.9	43.6	145.9	11.0	25.2	171.1
1971	70.3	37.8	44.3	152.4	7.3	18.7	171.1
1981	72.2	47.3	32.8	152.3	4.9	13.7	166.0
1991	80.9	60.0	29.2	171.0	4.9	15.2	186.2
2001	69.5	49.6	20.5	141.0	2.9	10.9	151.9
2011	66.3	59.7	23.9	.149.9	5.3	15.7	170.9
2012	69.4	57.8	21.9	149.1	4.9	15.2	169.3
2013	72.1	66.8	19.2	158.1	5.6	15.8	179.5
2014	72.3	66.8	22.6	161.6	6.0	16.9	178.6
2015	67.9	61.3	28.4	153.8	5.1	16.0	169.8
2016	67.2	72.9	26.1	162.0	4.8	15.9	177.9
2017(P)	69.3	70.1	30.0	164.9	6.3	19.9	184.7

P- Provisional figures based on 4th Advance Estimates of production for 2016-17

Note:- The net availability of foodgrains is estimated to be Gross Production (-) seed, feed & wastage (-) exports (+) imports(+/-) change in stocks.

The net availability of foodgrains divided by the population estimates for a particular year indicate per capita availability of foodgrains in terms of kg/year. Net availability, thus worked out further divided by the number of days in a year i.e., 365 days gives us net availability of foodgrains in terms of grams/ day.

Figures in respect of per capita net availability given above are not strictly representative of actual level of consumption in the country especially as they do not take in to account any change in stocks in possession of traders, producers and consumers.

Foodgrains includes rice, wheat,other cereals and all pulses

Source : Directorate of Economics and Statistics, DAC&FW

Table 10.2 Per Capita Availability of Certain Important Articles of Consumption

Year	Edible Oils [a] (kg.)	Vanaspati [b] (kg.)	Sugar (Nov-Oct) (kg.) [c]	Cloth (meters)[d] Cotton[e]	Man-made	Total	Tea (gm)	Coffee (gm)
(1)	(2)	(3)	(4)	(5)	(6)	(7)	(8)	(9)
1980-81	3.8	1.2	7.3	12.9	4.4	17.3	511	79
1990-91	5.5	1.0	12.7	15.1	9.0	24.1	612	59
2000-01	8.2	1.3	15.8	14.2	16.5	30.7	631	58
2009-10	13.3	1.1	17.9	19.7	23.4	43.1	709	86
2010-11	13.6	1.0	17.0	21.4	22.6	44.0	715	90
2011-12	13.8	1.0	18.7	19.8	20.7	40.5	728	95
2012-13	15.8	0.7	18.7	19.9	18.6	38.5	779	97
2013-14	16.8	0.8	19.5	19.9	16.4	36.2	744	100
2014-15	18.3	0.8	20.3	23.6	17.0	40.6	752	100
2015-16	17.7	0.8	19.4(P)	24.6	15.9	40.5	758	100

(P) Provisional

Note: a: Includes Groundnut Oil, Rapeseed and Mustard Oil, Sesamum Oil, Nigerseed Oil, Soy Bean Oil and Sunflower Oil but excludes oil for manufacture of Vanaspati.

b: Relates to calendar year.

c: Relates to actual releases for domestic consumption.

d: The data of cloth; prior to 1980-81 is calendar year wise, in meters up to 1984-85 and square meter from 1985-86 onwards.

e: Figures for blended/mixed fabrics were not separately available prior to 1969. These have been included under man-made fibre fabrics after 1969.

f: Figures upto 1971-72 relate to coffee season and thereafter on calendar year basis. The figures for 1972-73 correspond to 1973 and so on.

Source: Economic Survey 2017-18

Table 10.3: Trends in Consumer Expenditure since 2004-05 (percentage composition)

Item group	Rural			Urban		
	Share in total consumer expenditure			Share in total consumer expenditure		
	2004-05	2009-10	2011-12	2004-05	2009-10	2011-12
(1)	(2)	(3)	(4)	(5)	(6)	(7)
Cereal	18.0	15.6	12.0	10.1	9.1	7.3
Gram	0.1	0.2	0.2	0.1	0.1	0.1
Cereal substitutes	0.1	0.1	0.1	0.0	0.0	0.1
Pulses & products	3.1	3.7	3.1	2.1	2.7	2.1
Milk & products	8.5	8.6	9.1	7.9	7.8	7.8
Edible oil	4.6	3.7	3.8	3.5	2.6	2.7
Egg fish & meat	3.3	3.5	3.6	2.7	2.7	2.8
Vegetables	6.1	6.2	4.8	4.5	4.3	3.4
Fruits & nuts	1.9	1.6	1.9	2.2	2.1	2.3
Sugar	2.4	2.4	1.8	1.5	1.5	1.2
Salt & spices	2.5	2.4	2.4	1.7	1.5	1.7
Beverages, etc.	4.5	5.6	5.8	6.2	6.3	7.1
**Food Total**	**55.0**	**53.6**	**48.6**	**42.5**	**40.7**	**38.6**
Pan, tobacco, intox.	2.7	2.2	2.4	1.6	1.2	1.4
Fuel & light	10.2	9.5	9.2	9.9	8.0	7.6
Clothing & bedding	4.5	4.9	6.3	4.0	4.7	5.3
Footwear	0.8	1.0	1.3	0.7	0.9	1.2
Misc. & services	23.4	24.0	26.1	37.2	37.8	39.7
Durable goods	3.4	4.8	6.1	4.1	6.7	6.3
**Non-food Total**	**45.0**	**46.4**	**51.4**	**57.5**	**59.3**	**61.5**
**Total expenditure**	**100.0**	**100.0**	**100.0**	**100.0**	**100.0**	**100.0**

Note: Figures may not tally due to rounding off

Source: NSSO, M/O Statistics & Programme Implementation , Key Indicators of Household Consumer Expenditure NSSO 68th Round Survey (July'11 - June'12)

Table 10.4: Index of Terms of Trade between Agriculture and Non-Agricultural sectors

(Base: Triennium Ending 2011-12=100)

Year	Index of Prices Received for			Index of Prices Paid for Final Consumption for			Index of Prices Paid for		Combined Index for Prices Paid (IPP)	Index of Terms of Trade (ITT)
	Farmers	Agricultural Labourers	Index of Prices Received	Farmers	Agricultural Labourers	Combined Index for Final	Intermediate Consumption (IPP-IC)	Capital Formation (IPP-CF)		
Weight→	94.04	5.96	100.00	71.35	28.65	55.38	25.62	19.00	100.00	Col-4/10*100
(1)	(2)	(3)	(4)	(5)	(6)	(7)	(8)	(9)	(10)	(11)
2004-05	62.35	49.07	61.56	80.09	76.40	79.03	69.58	73.06	75.48	81.56
2005-06	61.40	55.22	61.03	80.25	77.01	79.32	71.69	74.53	76.46	79.82
2006-07	63.97	58.29	63.63	77.62	75.05	76.88	75.55	78.37	76.82	82.82
2007-08	72.06	61.83	71.45	82.85	82.10	82.63	80.51	84.11	82.37	86.74
2008-09	82.06	76.28	81.72	85.63	84.55	85.32	87.09	92.10	87.06	93.86
2009-10	90.89	85.61	90.57	91.70	91.40	91.62	92.67	92.71	92.10	98.35
2010-11	101.29	98.17	101.10	97.31	97.47	97.36	99.42	99.33	98.26	102.89
2011-12	107.82	116.22	108.32	110.98	111.14	111.03	107.91	107.96	109.64	98.79
2012-13	119.49	133.83	120.34	119.99	118.76	119.64	121.44	115.21	119.26	100.91
2013-14	131.67	151.40	132.85	128.36	129.25	128.62	132.30	117.84	127.51	104.19
2014-15	138.21	179.32	140.66	132.49	133.48	132.77	135.30	122.70	131.51	106.96
2015-16	142.78	185.04	145.30	139.46	141.16	139.94	138.33	121.90	136.10	106.76
2016-17(P)	151.46	197.32	154.19	127.34	126.92	127.22	143.42	122.67	130.50	118.15

* Provisional

Source: Directorate of Economics and Statistics, DAC&FW

Table 10.5: Index of Terms of Trade between Farmers and Non-Farmers

(Base: Triennium Ending 2011-12=100)

Year	Index of Prices Received (IPR)	Index of Price Paid (IPP) for				Index of Terms of Trade (ITT)
		Final Consumptio n (IPP-FC)	Intermediate Consumption (IPP-IC)	Capital Formation (IPP-CF)	Combined Index (IPP)	
Weight→		38.19	43.44	18.37	100.00	(Col-2/6*100)
(1)	(2)	(3)	(4)	(5)	(6)	(7)
2004-05	62.35	80.09	62.12	73.06	70.99	87.82
2005-06	61.40	80.25	64.60	74.53	72.40	84.80
2006-07	63.97	77.62	67.75	78.37	73.47	87.06
2007-08	72.06	82.85	71.52	84.11	78.16	92.20
2008-09	82.06	85.63	74.72	92.10	82.08	99.98
2009-10	90.89	91.70	89.09	92.71	90.75	100.15
2010-11	101.29	97.31	98.94	99.33	98.39	102.95
2011-12	107.82	110.98	111.98	107.96	110.86	97.26
2012-13	119.49	119.99	128.36	115.21	122.75	97.34
2013-14	131.67	128.36	145.19	117.84	133.74	98.46
2014-15	138.21	132.49	157.70	122.70	141.64	97.58
2015-16	142.78	139.46	164.85	121.90	147.26	96.96
2016-17(P)	151.46	127.34	171.57	122.67	145.69	103.96

(P): Provisional

Source: Directorate of Economics and Statistics, DAC&FW

KEY INDICATORS OF SITUATION OF AGRICULTURAL HOUSEHOLDS IN INDIA

Table 11.1: Estimated Number of Rural Households, Agricultural Households and Indebted Agricultural Households

State	Estimated Number of Rural Households ('00)	Estimated Number of Agricultural Households ('00)	Agricultural Households as percentage of rural households (%)	Estimated Number of Agricultural Households having loan ('00)	% of Agricultural Households indebted
(1)	(2)	(3)	(4)	(5)	(6)
Andhra Pradesh*	86763	35968	41.5	33421	92.9
Arunachal Pradesh	1659	1080	65.1	206	19.1
Assam	52494	34230	65.2	5995	17.5
Bihar	140611	70943	50.5	30156	42.5
Chhattisgarh	37472	25608	68.3	9538	37.2
Gujarat	58719	39305	66.9	16743	42.6
Haryana	25849	15693	60.7	6645	42.3
Himachal Pradesh	13251	8811	66.5	2457	27.9
Jammu & Kashmir	13746	11283	82.1	3463	30.7
Jharkhand	37516	22336	59.5	6464	28.9
Karnataka	77430	42421	54.8	32775	77.3
Kerala	51377	14043	27.3	10908	77.7
Madhya Pradesh	84666	59950	70.8	27414	45.7
Maharashtra	125182	70970	56.7	40672	57.3
Manipur	2584	1762	68.2	421	23.9
Meghalaya	4721	3544	75.1	84	2.4
Mizoram	936	758	81.0	47	6.2
Nagaland	4128	2621	63.5	65	2.5
Odisha	78120	44935	57.5	25830	57.5
Punjab	27552	14083	51.1	7499	53.2
Rajasthan	82722	64835	78.4	40055	61.8
Sikkim	1150	674	58.6	97	14.4
Tamil Nadu	93607	32443	34.7	26780	82.5
Telangana*	49309	25389	51.5	22628	89.1
Tripura	6635	2445	36.9	559	22.9
Uttar Pradesh	241328	180486	74.8	79081	43.8
Uttranchal	16498	10608	64.3	5387	50.8
West Bengal	141359	63624	45.0	32787	51.5
UTs	2394	718	30.0	267	37.2
**All India**	**1561442**	**902011**	**57.8**	**468481**	**51.9**

Source: Situation Assessment Survey of Agricultural Households (Jan-Dec 2013), National Sample Survey Office (NSSO)

* Separate estimates for the newly formed States of Telangana and Andhra Pradesh are provided in place of erstwhile State of Andhra Pradesh

Note;1 Reference period for Indebtedness is "as on the date of survey"

2 Indebtedness relates to all kind of outstanding loans irrespective of the purpose for which taken

Table 11.2: Indebtedness of Agricultural Households (all-India) in Different Size Classes of Land Possessed

Land Possessed (hectare)	Estimated Number of Agricultural Households (lakh)	% to Total in each Class	Estimated No. of Indebted Agricultural Households (lakh)	% to Total in each Class	% of Indebted Agricultural Households to Total	Average Outstanding Loan Amount (₹)
(1)	(2)	(3)	(4)	(5)	(6)	(7)
< 0.01	23.89	2.65	10.02	2.1	41.9	31100
0.01-0.40	287.66	31.89	135.97	29.0	47.3	23900
0.41-1.00	314.81	34.90	152.16	32.5	48.3	35400
1.01-2.00	154.58	17.14	86.11	18.4	55.7	54800
2.01-4.00	84.35	9.35	56.10	12.0	66.5	94900
4.01-10.00	33.02	3.66	25.21	5.4	76.3	182700
10 & above	3.71	0.41	2.92	0.6	78.7	290300
**All India**	**902.01**	**100.00**	**468.48**	**100.0**	**51.9**	**47000**

Source: Situation Assessment Survey of Agricultural Households (Jan-Dec 2013), National Sample Survey Office (NSSO)

Note:1 Reference period for land possession and Indebtedness is "as on the date of survey"
2 Indebtedness relates to all kind of outstanding loans irrespective of the purpose for which taken

Table 11.3: Incidence of Indebtedness in Major States

State	Estimated Number of Indebted Agricultural Households	% to Total Indebted Agricultural Households	% Share in Total Foodgrains (2016-17)*	
			Area	Production
(1)	(2)	(3)	(4)	(5)
1. Uttar Pradesh	79081	16.9	15.56	17.83
2. Maharashtra	40672	8.7	9.50	5.73
3. Rajasthan	40055	8.5	11.02	7.00
4. Andhra Pradesh**	33421	7.1	3.10	3.76
5. West Bengal	32787	7.0	4.67	6.19
6. Karnataka	32775	7.0	5.69	3.50
7. Bihar	30156	6.4	5.17	5.65
8. Madhya Pradesh	27414	5.9	13.3	11.96
9. Odisha	25830	5.5	3.75	3.29
10. Telangana**	22628	4.8	2.57	3.03
11. Punjab	7499	1.6	5.02	10.15
12. Other States/UTs	96163	20.5	20.65	21.91
**All India**	**468481**	**100.0**	**100.00**	**100.00**

* As per the Fourth Advance Estimates

**Separate estimates for the newly formed States of Telangana and Andhra Pradesh are provided in place of erstwhile State of Andhra Pradesh

Note: Reference period for Indebtedness is "as on the date of survey"

Source: Situation Assessment Survey of Agricultural Households (Jan-Dec 2013), National Sample Survey Office(NSSO) for Col-2 and 3 and Directorate of Economics & Statistics for Col-4 and 5

Table 11.4: Incidence of Indebtedness based on size of land possessed

State	% of Marginal Indebted Agricultural Households (upto 1.0 ha of land)	% of Small Indebted Agricultural Households (1.01 to 2.00 ha)	% of Semi-Medium Indebted Agricultural Households (2.01 to 4.00 ha)	% of Medium Indebted Agricultural Households (4.01 to 10.00 ha)	% of Large Indebted Agricultural Households (>10.00 ha)
(1)	(2)	(3)	(4)	(5)	(6)
1. Uttar Pradesh	77.2	13.4	7.1	2.1	0.2
2. Maharashtra	35.6	29.3	22.8	11.6	0.7
3. Madhya Pradesh	42.3	27.4	21.4	7.5	1.3
4. Rajasthan	50.9	18.3	16.8	12.1	1.9
5. Karnataka	52	24.4	14.8	7.4	1.3
6. Andhra Pradesh*	52.2	23.9	16.1	6.9	0.9
7. Telangana*	51	23.6	19.6	5	0.9
8. Bihar	86.7	10	2.6	0.7	0
9. West Bengal	91.1	7.4	1.4	0.1	0
10. Punjab	46.2	15.9	17.9	17.6	2.4
11. Odisha	78.7	15.2	4.3	1.8	0.1
**All India**	63.6	18.4	12	5.4	0.6

*Separate estimates for the newly formed States of Telangana and Andhra Pradesh are provided in place of erstwhile State of Andhra Pradesh

Note:1 Reference period for land possession and Indebtedness is "as on the date of survey"

2 Indebtedness relates to all kind of outstanding loans irrespective of the purpose for which taken

Source: Situation Assessment Survey of Agricultural Households (Jan-Dec 2013), National Sample Survey Office (NSSO)

Chart 11 (a): Distribution of Agricultural Households over Social Groups

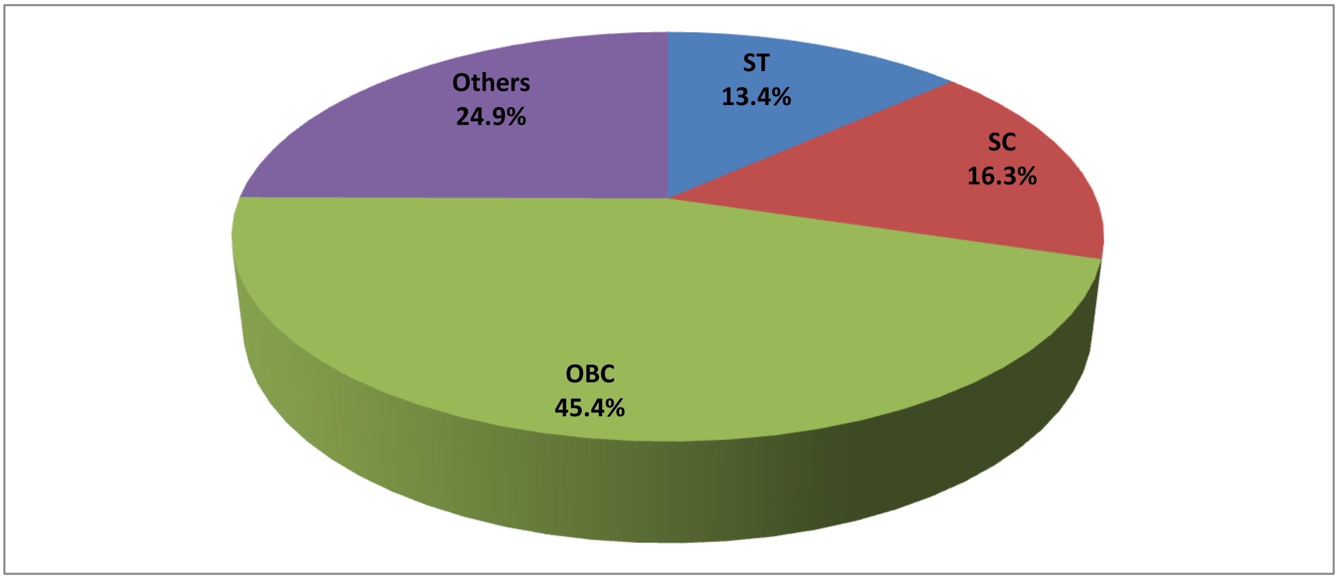

Source: Situation Assessment Survey of Agricultural Households (Jan-Dec 2013), National Sample Survey Office (NSSO)

Chart 11 (b): Distribution of Agricultural Households by principal source of income

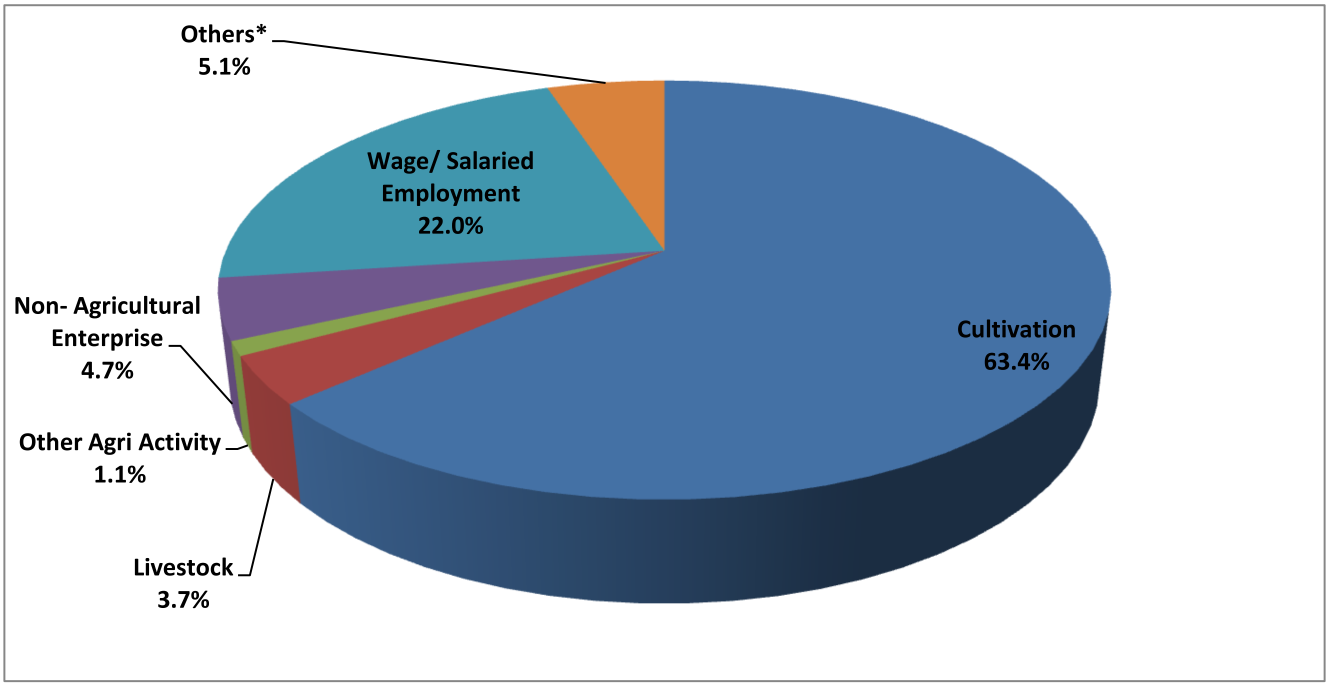

* Others include pension and remittance also

Source: Situation Assessment Survey of Agricultural Households (Jan-Dec 2013), National Sample Survey Office (NSSO)

Table 11.5: Average Monthly Expenses and Receipts for Crop Production per Agricultural Household for Major States during July 2012- June 2013

State	Average Total Expenses (Rs.)	Average Total Receipts (Rs.)	No. per 1000 households engaged in crop production	Estimated No. of Households engaged in Crop Production (00)
(1)	(2)	(3)	(4)	(5)
Andhra Pradesh	6191	8482	883	31747
Assam	788	5197	955	32689
Bihar	1454	3358	900	63872
Chhattisgarh	1128	4551	978	25035
Gujarat	2250	5773	832	32787
Haryana	6228	17144	721	11310
Jharkhand	571	2049	982	21939
Karnataka	2779	7908	961	40770
Kerala	2270	5872	980	13765
Madhya Pradesh	2284	6538	944	56604
Maharashtra	2654	6675	959	68051
Odisha	1001	2438	979	43999
Punjab	11768	28117	664	9357
Rajasthan	1730	5192	906	58705
Tamil Nadu	2538	5012	775	25148
Telangana	4267	8666	961	24397
Uttar Pradesh	1790	4912	914	165029
West Bengal	1819	2836	962	61206
**All India**	**2192**	**5542**	**920**	**829485**

Source: Situation Assessment Survey of Agricultural Households (Jan-Dec 2013), National Sample Survey Office (NSSO)

Table 11.6: Average Monthly Expenses and Receipts from Farming of Animals per Agricultural Household for Major States during July 2012- June 13

State	Average Total Expenses (Rs.)	Average Total Receipts (Rs.)	No. per 1000 households engaged in farming of animals	Estimated No. of Households engaged in farming of animals ('00)
(1)	(2)	(3)	(4)	(5)
Andhra Pradesh	1405	3619	486	17478
Assam	627	1890	633	21661
Bihar	1324	1810	574	40743
Chhattisgarh	491	426	298	7624
Gujarat	2399	4874	780	30726
Haryana	3256	6089	933	14649
Jharkhand	283	2420	558	12478
Karnataka	1524	2485	624	26452
Kerala	2249	3356	519	7288
Madhya Pradesh	914	2210	565	33881
Maharashtra	2118	3039	586	41572
Odisha	501	2919	544	24432
Punjab	3561	5303	952	13412
Rajasthan	1868	3131	766	49626
Tamil Nadu	1370	3196	603	19556
Telangana	1044	1994	394	9995
Uttar Pradesh	1068	1845	699	126168
West Bengal	916	1261	652	41506
**All India**	**1388**	**2604**	**627**	**565620**

Source: Situation Assessment Survey of Agricultural Households (Jan-Dec 2013), National Sample Survey Office (NSSO)

AGRICULTURAL PROJECTIONS

Table 12.1: Projected Demand and Supply of Food Commodities for the 12th Plan period

(Million tonnes)

Crop/Group of Crops	Projected Demand		Projected Supply	Actual Production	
	2016-17	2020-21	2016-17	2006-07	2011-12
(1)	(2)	(3)	(4)	(5)	(6)
Rice	110	117	98-106	93	104*
Wheat	89	98	93-104	76	94*
Maize	19	22		15	22*
Nutri Cereals	36	38	42-48	34	42*
Cereals	235	253	240-251	203	240*
Pulses	22	25	18-21	14	17*
Foodgrains	257	277	258-272	217	257*
Oilseeds/Edible oils	59	71	33-41	24	30*
Sugarcane/Sugar	279	312	365-411	355	358*
Vegetables	161	189		116	147**
Fruits	97	124		59	75**
Milk	141	173		103	122**
Fish	11			6.9	8.3**
Meat, other than poultry	3.7	5.0		2.3	2.7**
Poultry Meat	3.3	4.3			2.2 @

*4th advance estimate for 2011-12; **Production for the year 2010-11; @ Production 2010-11 for only commercial poultry meat.

Source: 12th Plan Document, Planning Commission

Table 12.2: Average Annual Growth Rate of Production of Selected Food Commodities in India: Actual Production Trends in 2005–15 and Projections by various agencies

Crops	Actual Growth rate	FAO/ OECD	USDA	FAPRI	IGC	NCAER	
						India stand-alone Cosimo Model	Econometric Model
	2004-2015	2015-24	2016-25	2015-21	2016-21	2016-25	2016-24
(1)	(2)	(3)	(4)	(5)	(6)	(7)	(8)
Wheat	3.21	1.4	1.78	1.3	2.05	1.69	0.96-1.73
Rice	1.52	1.76	1.19	NA	1.76	1.88	2.46-2.75
Nutri Cereals	-0.93	2.70	2.16	0.49	1.27	1.78	-0.41-3.30
Pulses	3.19						2.74-3.21
Total Oilseeds	0.81	2.21	3.72	1.47	2.66		3.47-4.29
Vegetable Oil	-0.93	2.05	4.20	1.48	NA	1.88	
Sugar	2.64	0.32	NA	3.24	NA	3.31	
Egg	4.44					3.66	
Milk	1.9					2.15	
Poultry	5.39					3.12	

Source: Agricultural Outlook & Situation Analysis Report (Fifth Medium-term Agricultural Outlook Report), July 2016, NCAER

Table 12.3: Production, Consumption & Year-end Stocks Projections of Major Crops

('000 Tonnes)

Commodity	Agency	Production			Consumption			Stocks (Year-end)		
		2014	2020	2024	2014	2020	2024	2014	2020	2024
(1)	(2)	(3)	(4)	(5)	(6)	(7)	(8)	(9)	(10)	(11)
**Wheat**	FAO/OECD	95,910	103,612	110,178	87,100	99,673	108,585	26,838	32,774	31,214
	USDA	88,940	104,293	110,039	93,940	103,418	108,945	11,900	9,426	10,930
**Rice**	FAO/OECD	104,800	114,898	122,637	98,750	108,700	115,287	20,000	15,980	14,979
	USDA	104,800	112,446	117,078	98,097	102,751	105,953	17,660	11,062	13,109
**Maize**	USDA	482 083	482 580	499 861	463 665	487 989	506 352	74 965	67 248	71 713
**Soy Bean**	USDA	1,255	1,869	2,012	4,049	5,187	5,781	260	250.1	247.4
**Sugar**	FAO/OECD	26,300	25,978	27,430	25,525	28,691	31,284	12,138	11,447	13,935

Source: Agricultural Outlook & Situation Analysis Report (Fifth Medium-term Agricultural Outlook Report), July 2016, NCAER

Table 12.4: Production Projections of Potatoes and Onion

Commodity	Production	2013	2014	2017	2023
(1)	(2)	(3)	(4)	(5)	(6)
**Potato**	Production (MMT)	41.5	46.0	53.6	76.5
	India share in world (%)	*11.3*	*12.4*	*14.0*	*18.8*
**Onion**	Production (MMT)	19.4	18.7	25.4	51.5
	India share in world (%)	*22.5*	*20.8*	*24.8*	*37.4*

Note: There are no global medium-term projections for potato and onions. Projections here are made by NCAER using the Directorate of Economics and Statistics data and FAO data on global area, production and yield of these commodities (2000–2014) and applying a trend growth rate

Source: Agricultural Outlook & Situation Analysis Report, (Fifth Medium-term Agricultural Outlook Report), July 2016, NCAER

Chart 12 (a): Projected Production of Rice, Wheat and Coarse Grains in India

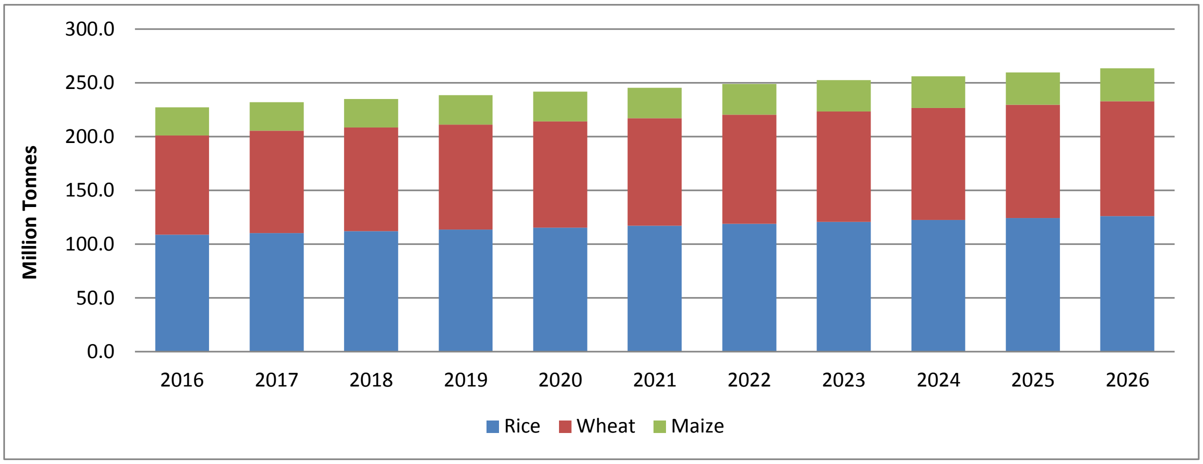

Source: OECD- FAO Agriculture Outlook (2017-2026)

Chart 12 (b): Projected Production of Vegetables and Fruits in India

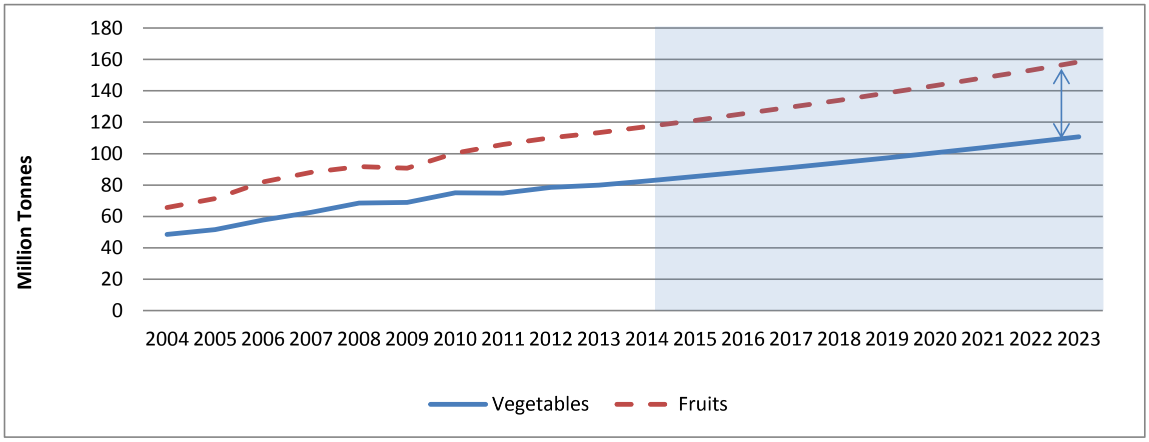

Source: OECD- FAO Agriculture Outlook (2014-2023)

Chart 12 (c): Projected Market Support Prices in India and World Prices for Rice and Wheat

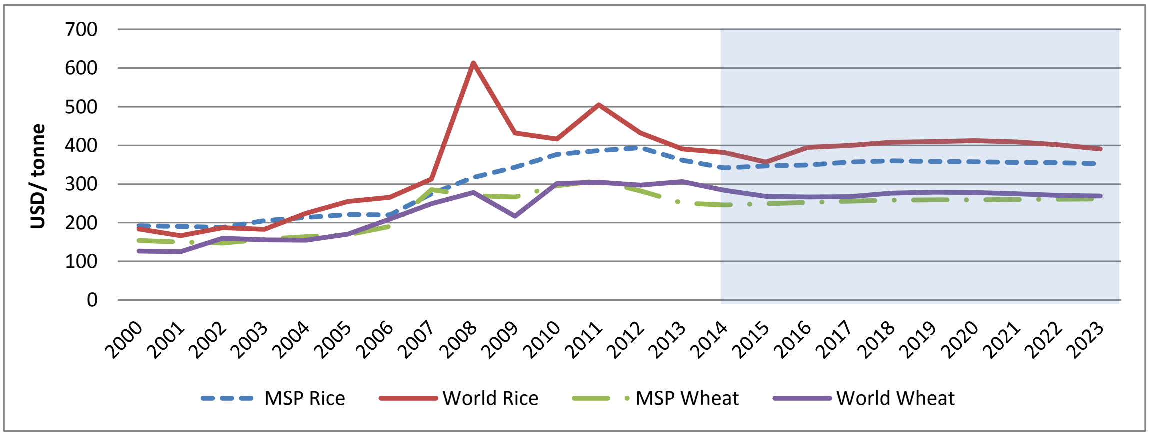

Note: Minimum Support Price (MSP) for paddy rice is adjusted to milled basis.

Market Margins of 15% are added to MSP. The world refernce price for wheat is US Gulf HRW, and for rice is Vietnam 5%.

Source: OECD- FAO Agriculture Outlook (2014-2023)

Chart 12 (d): Projected Per capita Consumption of Cereals in India

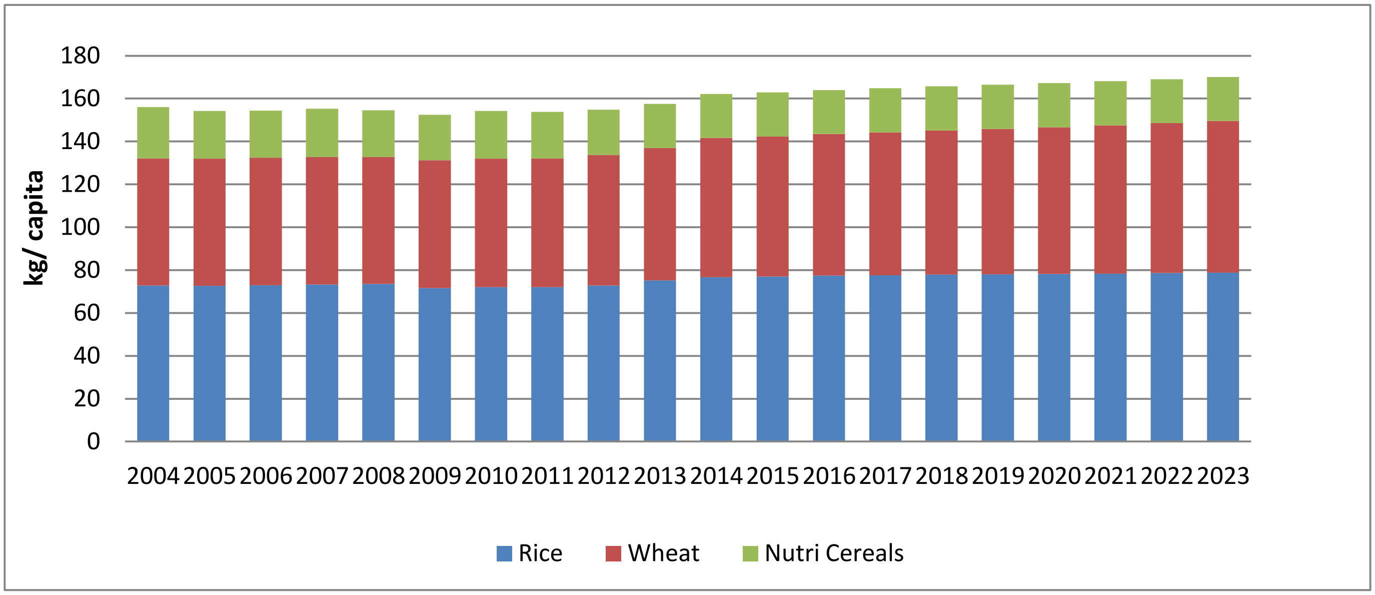

Source: OECD- FAO Agriculture Outlook (2014-2023)

Chart 12 (e): Projected Calorie and Protein Consumption in India

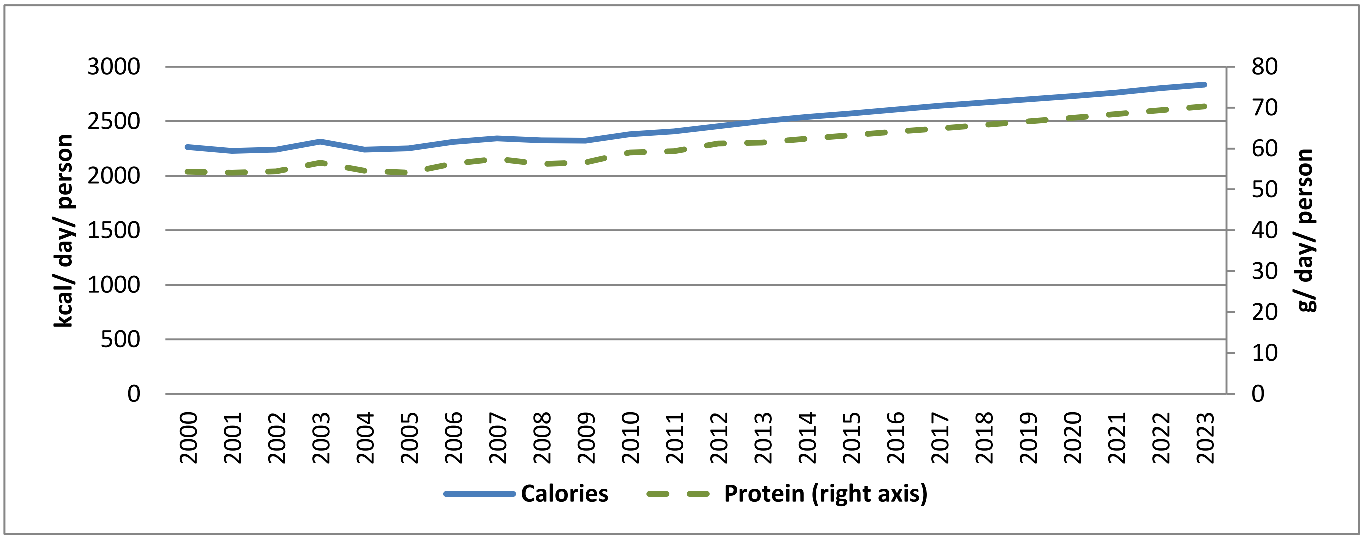

Source: OECD- FAO Agriculture Outlook (2014-2023)

IMPORTS & EXPORTS OF AGRICULTURAL PRODUCTS

Table 13.1: India's Imports and Exports of Agricultural Commodities

(Value in ₹ Crore)

Year	Agricultural Imports	Total Imports	Percentage share of Agricultural Imports in Total Imports	Agricultural Exports	Total Exports	Percentage share of Agricultural Exports in Total Exports
(1)	(2)	(3)	(4)	(5)	(6)	(7)
1990-91	1205.86	43170.82	2.79	6012.76	32527.28	18.49
2000-01	12086.23	228306.64	5.29	28657.37	201356.45	14.23
2010-11	51073.97	1683466.96	3.03	113046.58	1136964.22	9.94
2011-12	70164.51	2345463.24	2.99	182801.00	1465959.31	12.47
2012-13	95718.89	2669161.96	3.59	227192.61	1634318.29	13.90
2013-14	85727.30	2715433.91	3.16	262778.54	1905011.00	13.79
2014-15	121319.02	2737086.58	4.43	239681.04	1896445.47	12.64
2015-16	140289.22	2490298.08	5.63	215396.55	1716378.05	12.55
2016-17 (P)	164726.83	2577665.59	6.39	226651.94	1849428.76	12.26

(P) - Provisional

Note: Commodities covered under agriculture sector have been revised after inter-Departmental consultation

Source: Directorate General of Commercial Intelligence & Statistics, D/o Commerce, Kolkata.

Chart 13 (a): Trends in Agricultural Imports/ Exports and their percentage share in Total Imports/ Exports

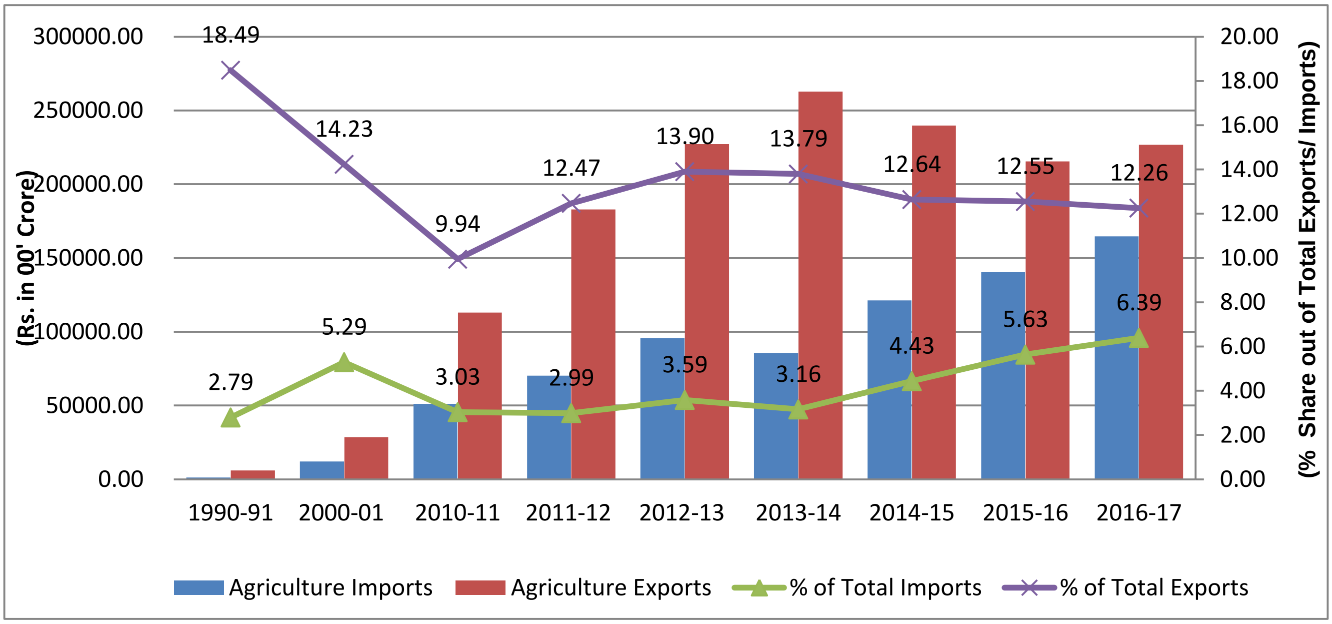

Source: Directorate General of Commercial Intelligence & Statistics, D/o Commerce, Kolkata.

Table 13.2 : India's Imports of Agricultural Commodities

(Quantity: '000 tonnes)
(Value in ₹ Crore)

Commodity	2014-15		2015-16		2016-17 (P)	
	Quantity	Value	Quantity	Value	Quantity	Value
(1)	(2)	(3)	(4)	(5)	(6)	(7)
Tea	28.39	388.66	23.72	377.47	24.89	338.35
Coffee	74.88	930.47	65.61	801.83	78.04	926.81
Rice- Basmati	0.00	0.00	0.00	0.00	0.00	0.00
Rice(other than Basmati)	1.96	10.83	1.02	5.91	1.14	7.25
Wheat	29.49	61.34	517.67	872.59	5749.43	8509.05
Other cereals	23.40	61.76	206.14	344.31	311.37	493.18
Pulses	4584.85	17062.94	5797.71	25619.06	6609.49	28523.18
Tobacco unmanufactured	1.93	98.17	2.88	137.30	1.97	77.21
Tobacco manufactured	-	200.28	-	193.92	0.00	228.54
Spices	163.09	4393.25	197.06	5399.95	242.29	5760.25
Cashew	933.19	6599.74	961.67	8701.28	774.51	9027.09
Cashew nut shell liquid	1.72	10.03	1.86	5.60	1.69	3.67
Sesame seeds	34.77	379.99	23.60	179.66	69.03	442.15
Niger seeds	0.70	3.73	5.78	44.14	10.66	82.82
Groundnut	0.13	0.49	0.11	0.31	0.33	1.39
Other oil seeds	51.56	163.29	62.51	218.62	116.64	392.36
Vegetable oils	12731.60	64889.60	15643.74	68676.62	14007.39	73038.98
Oil meals	165.10	272.65	256.55	429.91	550.43	974.59
Guargam meal	0.13	5.72	0.63	13.93	0.18	2.41
Castor oil	0.05	1.81	0.03	1.10	0.11	1.50
Shellac	1.77	59.54	0.71	19.48	0.46	13.43
Sugar	1538.64	3668.21	1943.13	4037.86	2146.15	6868.61
Mollases	60.28	30.14	17.27	7.50	13.84	9.04
Fruits / vegetable seeds	14.01	611.53	14.33	703.03	14.07	653.33
Fresh fruits	900.98	9566.81	857.90	11071.57	1057.51	11290.62
Fresh vegetables	8.24	11.14	140.73	394.45	8.55	11.12
Processed vegetables	10.96	104.45	15.38	120.33	13.32	115.26
Processed fruits and juices	33.55	499.54	40.49	526.49	42.99	548.10
Cereal preparations	72.72	583.93	61.70	575.42	66.46	579.03
Cocoa products	65.39	1551.63	56.42	1398.91	63.61	1542.28
Milled products	3.47	17.72	4.39	21.40	3.56	16.22
Misc processed items	-	1785.23	-	1811.12	0.00	2115.82
Animal casings	0.00	0.00	0.00	0.00	0.00	0.00
Buffalo meat	0.00	0.00	0.00	0.00	0.00	
Sheep/goat meat	0.09	8.73	0.05	4.80	0.13	8.50
Other meat	0.47	19.50	0.50	17.18	0.59	19.03
Processed meat	0.17	5.17	0.07	2.75	0.13	4.47
Dairy products	43.84	375.01	18.30	371.58	16.91	254.84
Poultry products	-	38.22	-	26.42	0.00	29.46
Floriculture products	4.82	113.37	4.77	114.40	5.57	133.81
Alcoholic beverages	-	2508.66	-	2935.85	452.72	3590.33
Marine products	27.72	453.90	50.35	639.77	52.02	633.39
Ayush and herbal products	23.48	352.58	11.67	354.35	11.36	358.87
Jute, raw	44.00	139.58	88.37	363.44	138.84	704.13
Jute hessian	-	177.79	-	181.41	0.00	57.45
Cotton raw incld. Waste	289.39	3101.92	233.14	2566.21	499.62	6338.92
**Total Agricultural Imports**	-	**121319.02**	-	**140289.22**	**-**	**164726.83**
**Total National Imports**	-	**2737086.58**	-	**2490298.08**	**-**	**2577665.59**
**% Share of Agricultural Import in National Imports**	-	**4.43**	-	**5.63**	**-**	**6.39**

(P) - Provisional

Note: Commodities covered under agriculture sector have been revised after inter-Departmental consultation.

Source: Directorate General of Commercial Intelligence & Statistics, D/o Commerce, Kolkata.

Table 13.3: India's Exports of Agricultural Commodities

(Quantity: '000 tonnes)
(Value in ₹ Crore)

Commodity	2014-15		2015-16		2016-17 (P)	
	Quantity	Value	Quantity	Value	Quantity	Value
(1)	(2)	(3)	(4)	(5)	(6)	(7)
Tea	234.39	4171.25	245.70	4719.00	243.43	4905.64
Coffee	463.55	4973.25	255.74	5125.45	288.61	5646.43
Rice -Basmati	3698.93	27586.71	4045.83	22718.60	3985.21	21512.91
Rice(other than Basmati)	8302.21	20441.55	6464.59	15483.39	6770.83	16929.88
Wheat	2924.05	4991.81	666.68	1061.77	265.61	447.85
Other cereals	3515.35	5262.16	967.93	1702.50	734.77	1425.77
Pulses	222.14	1218.31	255.72	1655.90	136.72	1277.73
Tobacco unmanufactured	219.57	4162.71	215.32	4373.45	204.45	4249.85
Tobacco manufactured	-	1705.88	-	2078.91	-	2174.12
Spices	939.01	14847.74	831.68	16630.37	1014.45	19111.25
Cashew	134.57	5565.85	103.13	5027.99	91.79	5278.61
Cashew nut shell liquid	10.94	55.81	11.68	57.59	11.40	43.99
Sesame seeds	375.66	4717.77	328.46	3012.31	307.33	2695.84
Niger seeds	18.16	108.96	14.12	123.27	14.07	117.22
Groundnut	788.31	4675.37	542.73	4075.63	725.71	5444.33
Other oil seeds	247.54	1135.36	204.62	964.47	193.27	846.58
Vegetable oils	423.72	580.13	30.60	522.94	60.47	779.97
Oil meals	3904.59	8129.18	2056.36	3599.56	2632.26	5410.10
Guargam meal	665.11	9478.26	325.25	3233.87	419.95	3106.62
Castor oil	566.46	4710.42	586.78	4616.10	599.20	4521.51
Shellac	5.24	267.47	6.39	203.31	6.06	225.53
Sugar	1955.19	5328.83	3844.45	9824.52	2544.01	8659.54
Mollases	247.61	193.01	818.57	656.84	390.67	314.94
Fruits / vegetable seeds	12.50	427.04	13.10	529.19	11.29	522.75
Fresh fruits	539.23	3160.08	654.66	4191.24	817.06	4974.21
Fresh vegetables	2081.80	4666.45	2104.36	5237.10	3404.07	5790.71
Processed vegetables	186.04	1721.89	174.43	1697.22	192.86	1765.75
Processed fruits and juices	588.38	3626.86	532.29	3767.08	533.15	3921.08
Cereal preparations	313.67	3036.64	316.54	3358.12	339.95	3565.55
Cocoa products	33.37	848.66	32.65	1267.61	25.65	1086.77
Milled products	420.85	1030.61	431.46	1102.73	255.80	813.54
Misc processed items	-	2772.44	-	2907.85	-	3053.79
Animal casings	0.26	19.33	0.21	17.02	0.17	13.84
Buffalo meat	1503.51	29282.58	1314.22	26684.22	1323.58	26161.38
Sheep/goat meat	23.61	828.11	21.95	837.76	22.01	869.84
Other meat	-	2.67	-	0.00	-	0.21
Processed meat	0.41	14.20	0.28	6.16	-	4.58
Dairy products	104.17	2169.03	77.53	1677.46	90.35	1701.18
Poultry products	-	651.19	-	769.14	-	530.44
Floriculture products	22.95	460.80	22.69	483.41	22.02	546.71
Alcoholic beverages	-	2264.89	-	2030.92	232.18	2004.79
Marine products	1231.81	33688.38	978.04	31219.48	1185.27	39593.78
Ayush and herbal products	92.06	2169.49	95.88	2385.49	83.36	2693.57
Jute, raw	37.39	119.20	25.11	113.58	18.18	76.63
Jute hessian	-	770.08	-	824.89	-	927.32
Cotton raw incld. Waste	1142.53	11642.64	1347.07	12821.13	996.09	10907.32
**Total Agricultural Exports**	**-**	**239681.04**	**-**	**215396.55**	**-**	**226651.94**
**Total National Exports**	**-**	**1896445.47**	**-**	**1716378.05**	**-**	**1849428.76**
**% Share of Agricultural Exports in National Exports**	**-**	**12.64**	**-**	**12.55**	**-**	**12.26**

(P) - Provisional

Note: Commodities covered under agriculture sector have been revised after inter-Departmental consultation.

Source: Directorate General of Commercial Intelligence & Statistics, D/o Commerce, Kolkata.

Table 13.4: Leading Exporters and Importers of Agricultural Products in 2016

(Billion dollars and Percentage)

Country/Group	Value	Share in world exports/imports				Annual percentage change			
	2016	2000	2005	2010	2016	2010-16	2014	2015	2016
(1)	(2)	(3)	(4)	(5)	(6)	(7)	(8)	(9)	(10)
**Exporters**									
European Union (28)	598	41.9	44.2	39.3	37.7	2	1	-13	3
extra-EU (28) exports	160	10.0	9.7	9.4	10.1	4	1	-12	2
United States of America	165	13.0	9.7	10.5	10.4	2	4	-12	3
Brazil	77	2.8	4.1	5.0	4.9	2	-3	-9	-4
China	76	3.0	3.4	3.8	4.8	7	6	-3	5
Canada	63	6.3	4.8	3.8	4.0	3	4	-7	-1
Indonesia[a]	38	1.4	1.6	2.6	2.4	1	3	-10	-4
Argentina	37	2.2	2.2	2.5	2.3	1	-10	-9	7
Thailand	37	2.2	2.1	2.6	2.3	1	-2	-8	1
Australia	34	3.0	2.5	2.0	2.1	4	3	-7	-6
**India**	**34**	**1.1**	**1.2**	**1.7**	**2.1**	**6**	**-3**	**-19**	**-5**
**Above 10**	**1159**	**76.9**	**75.9**	**73.9**	**73.0**	**-**	**-**	**-**	**-**
**Importers**									
European Union (28)	602	42.7	45.3	40.0	36.8	1	1	-12	2
extra-EU (28) imports	166	13.2	12.6	11.1	10.2	1	3	-10	0
United States of America	160	11.6	10.6	8.4	9.8	5	7	0	2
China	155	3.3	5.0	7.8	9.5	6	3	-6	-3
Japan	75	10.4	7.3	5.6	4.6	-1	-5	-10	2
Canada[b]	38	2.6	2.4	2.3	2.3	3	4	-5	-1
Korea, Republic of	32	2.2	1.9	1.9	2.0	3	5	-6	-3
**India**	**29**	**0.7**	**0.8**	**1.3**	**1.8**	**8**	**12**	**1**	**5**
Hong Kong, China	28	-	-	-	-	5	5	-6	3
retained imports[a]	18	1.1	0.8	1.0	1.1	5	6	-9	1
Mexico[b]	28	1.8	1.8	1.7	1.7	3	3	-8	1
Russian Federation[b]	26	1.3	1.9	2.6	1.6	-5	-8	-33	-6
**Above 10**	**1162**	**77.5**	**77.9**	**72.8**	**71.1**	**-**	**-**	**-**	**-**

Note: 'a' Includes WTO Secretariat's estimates and 'b' Imports are valued f.o.b.

Source: World Trade Statistical Review 2017, World Trade Organisation

INTERNATIONAL COMPARISONS

Table 14.1: India's Position in World Agriculture in 2015

Item	India	World	% Share	India's Rank	Next to
(1)	(2)	(3)	(4)	(5)	(6)
**1. Total Area (Million Hectares)**	329	13467	2.4	Seventh	Russian Federation, Canada, U.S.A., China,Brazil, Australia
Land Area	297	13009	2.3	Seventh	Russian Federation, China, U.S.A., Canada, Brazil, Australia
Arable Land	156	1426	11.0	Second	U.S.A.
**2. Population (Million)**					
Total	1309	7383	17.7	Second	China
Rural	862	3368	25.6	First	
**3. Crop Production (Million Tonnes)**					
**(A) : Total Cereals**	284	2796	10.2	Third	China, U.S.A
Wheat	87	737	11.7	Second	China
Rice (Paddy)	157	740	21.2	Second	China
**(B): Total Pulses**	17	78	22.5	First	
**(C) : Oilseeds**					
Groundnut (in shell)	7	45	15.0	Second	China
Rapeseed	6	71	8.8	Third	Canada, China
**(D): Commercial Crops**					
Sugarcane	362	1887	19.2	Second	Brazil
Tea	1.2	5.7	21.8	Second	China
Coffee (green)	0.3	8.9	3.7	Seventh	Brazil, Vietnam, Columbia, Indonesia, Ethiopia, Honduras
Jute & Jute like Fibres	1.9	3.5	54.0	First	
Cotton (lint)	6.2	26.2		Second	China
Tobacco Unmanufactured	0.7	7.0	10.7	Third	China, Brazil
**4. Fruits & Vegetables Production (Million Tonnes)**					
(A) :Vegetables& Melons	119	1195	10.0	Second	China
(B) : Fruits excluding Melons	87	710	12.3	Second	China
(C) : Potatoes	48	377	12.7	Second	China
(D) : Onion (Dry)	19	91	20.8	Second	China
**5. Livestock (Million Heads)**					
(A) : Cattle	185	1452	12.7	Second	Brazil
(B) : Buffaloes	111	196	56.4	First	
(C) : Camels	0.4	28	1.3	Twelfth	Somalia, Sudan, Kenya, Niger, Mauritania, Chad, Ethiopia, Pakistan, Mali, Yemen, U.A.E
(D) : Sheep	62	1160	5.4	Third	China, Australia
(E) : Goats	132	979	13.5	Second	China
(F) : Chicken	740	22112	3.3	Sixth	China, U.S.A, Indonesia, Brazil, Iran
**6. Animal Products (Million Tonnes)**					
(A) : Milk Total	156	807	19.3	First	
(B) : Eggs (Primary) Total	4	78	5.5	Third	China, U.S.A
(C) : Meat Total	7	324	2.2	Sixth	China, U.S.A, Brazil, Russian federation, Germany

Source : FAOSTAT (as on 11.01.2018)

Table 14.2: Area, Production and Yield of Principal Crops in various Countries in 2015

Area - "000" Hectares
Production - "000" Tonnes
Yield - Kg./Hectare

Country	Area	Production	Yield	Production (%)
(1)	(2)	(3)	(4)	(5)
**1. Paddy**				
**World**	**160762**	**740085**	**4604**	**100.00**
China	30216	208230	6891	28.14
India	43390	156540	3608	21.15
Indonesia	14117	75398	5341	10.19
Bangladesh	11372	51278	4509	6.93
Viet Nam	7831	45105	5760	6.09
Thailand	9718	27702	2851	3.74
Myanmar	6769	26210	3872	3.54
Philippines	4656	18150	3898	2.45
Brazil	2138	12301	5753	1.66
Japan	1506	9986	6631	1.35
**2. Wheat**				
**World**	**222157**	**736985**	**3317**	**100.00**
China	24141	130185	5393	17.66
India	31470	86530	2750	11.74
USA	19058	55840	2930	7.58
France	5480	42750	7801	5.80
Canada	9577	27594	2881	3.74
Germany	3283	26550	8088	3.60
Ukraine	6840	26532	3879	3.60
Pakistan	9204	25086	2726	3.40
Oceania	12431	24156	1943	3.28
Australia	12384	23743	1917	3.22
**3. Maize**				
**World**	**182490**	**1010609**	**5538**	**100.00**
USA	32678	345486	10572	34.19
China	38119	224630	5893	22.23
Brazil	15406	85285	5536	8.44
Argentina	4627	33818	7309	3.35
Mexico	7100	24694	3478	2.44
Ukraine	4084	23328	5713	2.31
India	8690	22570	2597	2.23
Indonesia	3787	19612	5178	1.94
France	1637	13716	8378	1.36
Canada	1312	13559	10337	1.34
**4. Pulses**				
**World**	**81616**	**77573**	**950**	**100.00**
India	27040	17482	647	22.54
Myanmar	4250	6289	1480	8.11
Canada	3251	6068	1867	7.82
China, mainland	2392	4164	1741	5.37
Brazil	2885	3097	1074	3.99
Ethiopia	1615	2955	1830	3.81
United States of America	1420	2575	1813	3.32
Russian Federation	1582	2467	1560	3.18
Nigeria	3776	2371	628	3.06
United Republic of Tanzania	2084	2016	968	2.60

(Contd..)

Country	Area	Production	Yield	Production (%)
(1)	(2)	(3)	(4)	(5)
**5. Sugarcane**				
**World**	**26664**	**1886875**	**70764**	**100.00**
Brazil	10111	750290	74203	39.76
India	5070	362333	71466	19.20
China	1600	116968	73121	6.20
Thailand	1401	94138	67206	4.99
Pakistan	1131	65482	57873	3.47
Mexico	759	55396	73023	2.94
Colombia	409	36710	89654	1.95
Oceania	421	34276	81373	1.82
Indonesia	456	25349	55611	1.34
Philippines	421	22926	54417	1.22
**6. Groundnut (in shell)**				
**World**	**26800**	**45077**	**1682**	**100.00**
China	4616	16440	3562	36.47
India	4560	6771	1485	15.02
Nigeria	2802	3467	1238	7.69
USA	634	2817	4442	6.25
United Republic of Tanzania	1625	1836	1130	4.07
Myanmar	950	1518	1598	3.37
Senegal	1135	1050	925	2.33
Sudan	1465	1042	711	2.31
Argentina	425	1011	2380	2.24
Indonesia	454	605	1333	1.34
**7. Tobacco Unmanufactured**				
**World**	**3889**	**6985**	**1796**	**100.00**
China	1314	2832	2156	40.55
Brazil	406	867	2137	12.42
India	444	747	1683	10.69
USA	133	326	2453	4.67
Indonesia	209	194	927	2.77
Zimbabwe	132	171	1295	2.45
Malawi	142	120	851	1.72
Pakistan	54	120	2231	1.72
Argentina	55	109	1977	1.56
Turkey	106	75	708	1.07

Source: FAOSTAT (as on 26.12.2017).

Table 14.3: Major Macro-Economic Indicators of India, G-20 and the World in 2016

Country	Area (Sq. Km.) 2010	Population (Million)	GDP Billion (US$)	Domestic Product Per Capita Income (US$)	Purchasing Power in Billion US $
(1)	(2)	(3)	(4)	(5)	(6)
Argentina	2766890	43	579	13,428	964
Australia	7686850	24	1241	51,642	1,137
Brazil	8511965	204	1800	8,802	3,208
Canada	9976140	36	1573	43,935	1,628
China	9596960	1375	11385	8,280	19,510
France	547030	64	2423	37,728	2,647
Germany	357021	82	3371	41,267	3,842
**India**	**3287590**	**1293**	**2182**	**1,688**	**8,027**
Indonesia	1919440	255	873	3,416	2,839
Italy	301230	61	1819	29,847	2,174
Japan	377835	127	4116	32,481	4,842
Mexico	1972550	121	1161	9,592	2,220
Russia	17075200	146	1236	8,447	3,474
Saudi Arabia	1960582	31	632	20,139	1,681
South Africa	1219912	55	317	5,784	724
Republic of Korea	98480	51	1393	27,513	1,849
Turkey	780580	78	722	9,290	1,576
United Kingdom	244820	65	2865	44,118	2,660
USA	9629091	321	17968	55,904	17,968
European Union	4324782	514	16266	37,802	19,176
**World**	**510072000**	**7256**	**73507**	**10,699**	**113,162**

Source : Statistical Year Book India- 2016, Ministry of Statistics & Programme Implementation

Table 14.4: Major Macro Economic, Land-Use and Social Indicators of BRICS Economies in 2015

Indicators	Brazil	Russia	China	India	South Africa
(1)	(2)	(3)	(4)	(5)	(6)
Macro-Economic Indicators					
Total Population (Million)	205.96	144.10	1371.22	1309.05	55.01
Urban Population (% of Total)	85.69	74.00	55.61	32.75	64.80
Share in World Exports (%)	1.20	2.10	13.80	1.60	0.50
GDP in PPP (US $ Billion) - *current international $	3222.82	3470.24	19852.73	8036.54	729.29
GDP (US $ Billion)	1803.65	1365.86	11064.67	2089.87	317.61
Per Capita GDP (US $)	8757.21	9329.30	8069.21	1596.47	5773.48
Land Use Indicators					
Land Area (Million ha)	835.81	1637.69	938.82	297.32	121.31
Arable Land Area (Million Ha)	80.02	123.12	119.00	156.46	12.50
Irrigated Land (Million Ha)	5.40	4.30	71.74	70.40	1.67
Irrigated Land (% of arable land)	6.75	3.49	60.29	45.00	13.36
Area Harvested for Cereals (Million Ha)	21.20	42.84	95.92	99.53	3.37
Production of Cereals (Million Tonnes)	106.03	102.45	573.80	284.33	11.93
Major Health Indicators*					
Health expenditure per capita (current US$)	947.43	892.85	419.73	74.99	570.21
Health expenditure, public (% of GDP)	3.83	3.69	3.10	1.41	4.24
Health expenditure, total (% of GDP)	8.32	7.07	5.55	4.69	8.80
Life expectancy at birth, total (years)	74.96	70.74	75.96	68.05	60.95
Mortality rate, infant (per 1,000 live births)	14.70	7.10	9.90	37.90	35.60
Labor and Social Protection Indicators					
Age dependency ratio, young (% of working-age population)	32.38	24.16	24.35	43.62	44.75
Birth rate, crude (per 1,000 people)	14.41	13.30	12.10	19.27	21.30
Population growth (annual %)	0.85	0.19	0.51	1.17	1.58
Labor participation rate, total (% of total population ages 15+)	64.10	63.66	69.74	53.95	54.65
Unemployment, total (% of total labor force)	8.50	5.59	4.50	3.50	25.20

** Figure relates to 2014*

Source:

1. World Development Indicators, World Bank
2. World Trade Statistical Review 2016, WTO
3. FAO Statistics

Table 14.5: Major Economic and Land-Use Indicators : India and China

Indicator Name		China			India		
		2014	2015	2016	2014	2015	2016
(1)		(2)	(3)	(4)	(5)	(6)	(7)
GDP growth (annual %)		7.30	6.90	6.69	7.51	8.01	7.11
Population growth (annual %)		0.51	0.51	0.54	1.19	1.17	1.15
Arable land (% of land area)		11.25	12.67		52.62	52.62	
Agricultural area ('000 Hectare)		515358	528635		179721	179721	
Agricultural area (% of land area)		54.81	56.22		60.45	60.45	
Total area equipped for irrigation (% of total agricultural area)		13.66	13.57		39.17	39.17	
Agriculture, value added (% of GDP)		9.06	8.83	8.56	18.02	17.46	17.35
Methane emissions from agriculture (CO2 equivalent in gigagrams)		349729.11			406866.33		
Fertilizer consumption (kilograms per hectare of arable land and land under permanent crops)		565.25			165.12		
Inflation, consumer prices (annual %)		1.99	1.44	2.01	6.65	4.91	4.94
Land-Use Indicators (2016)							
**Area Harvested (Million Hectares)**	Wheat	24.07	24.14	24.35	30.47	31.47	30.23
	Rice (Paddy)	30.58	30.47	30.45	44.11	43.39	42.96
	Maize	37.15	38.15	38.98	9.26	8.69	10.20
	Soy Bean	6.80	6.51	6.64	11.09	11.67	11.50
**Production (Million Tonnes)**	Wheat	126.22	130.19	131.70	95.85	86.53	93.50
	Rice (Paddy)	208.24	209.81	211.09	157.20	156.54	158.76
	Maize	215.81	224.80	231.84	24.17	22.57	26.26
	Soy Bean	12.16	11.79	11.97	10.37	8.57	14.01

Source: FAO Statistics and World Development Indicators, World Bank

Note: Figures for China pertains to China Mainland only

Table 14.6: Inflation (Consumer Prices) in Major Economies (Annual %)

Country	2011	2012	2013	2014	2015	2016
(1)	(2)	(3)	(4)	(5)	(6)	(7)
Australia	3.3	1.8	2.4	2.5	1.5	1.3
Brazil	6.6	5.4	6.2	6.3	9.0	8.7
Canada	2.9	1.5	0.9	1.9	1.1	1.4
China	5.4	2.6	2.6	2.0	1.4	2.0
Euro area	3.3	2.5	1.4	0.2	0.0	0.2
Germany	2.1	2.0	1.5	0.9	0.2	0.5
European Union	3.3	2.7	1.4	0.2	-0.1	0.2
Italy	2.7	3.0	1.2	0.2	0.0	-0.1
India	8.9	9.3	10.9	6.4	5.9	4.9
Indonesia	5.4	4.3	6.4	6.4	6.4	3.5
Hong Kong SAR, China	5.3	4.1	4.4	4.4	3.0	2.4
Japan	-0.3	0.0	0.4	2.7	0.8	-0.1
Mexico	3.4	4.1	3.8	4.0	2.7	2.8
OECD members	3.3	2.5	1.4	0.6	0.2	0.4
Russian Federation	8.4	5.1	6.8	7.8	15.5	7.1
Singapore	5.3	4.5	2.4	1.0	-0.5	-0.5
South Asia	9.7	9.4	7.6	6.7	3.5	3.7
South Africa	5.0	5.7	5.4	6.4	4.6	6.3
Switzerland	0.2	-0.7	-0.2	0.0	-1.1	-0.4
United Kingdom	4.5	2.8	2.6	1.5	0.1	0.6
United States	3.2	2.1	1.5	1.6	0.1	1.3
World	5.0	3.9	2.7	2.7	1.4	1.6

Source: World Bank

Chart 14(a): Projected Wheat Production in China and India

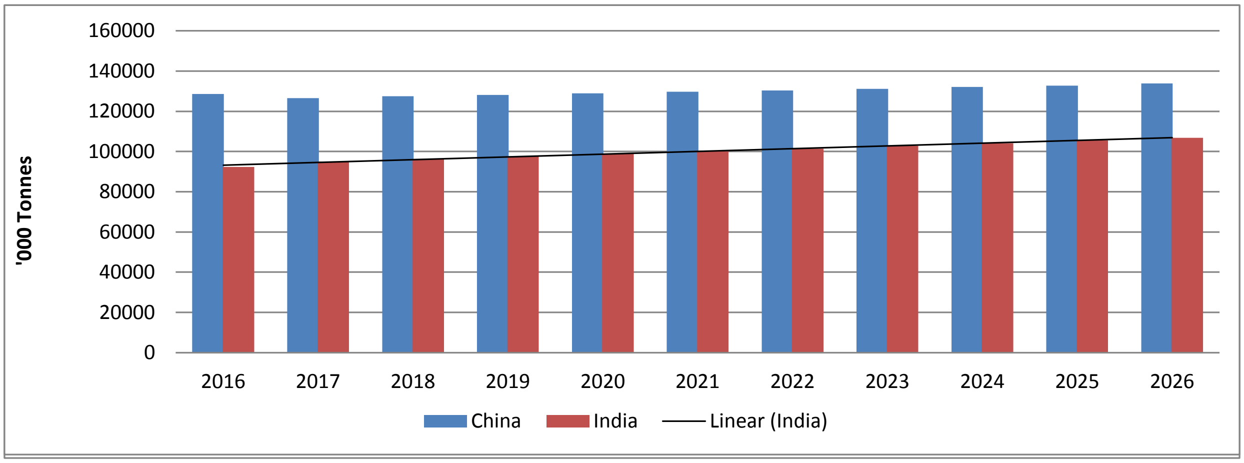

Source: OECD- FAO Agriculture Outlook 2017-26

Chart 14 (b): Projected Nutri Grains Production in U.S, China and India

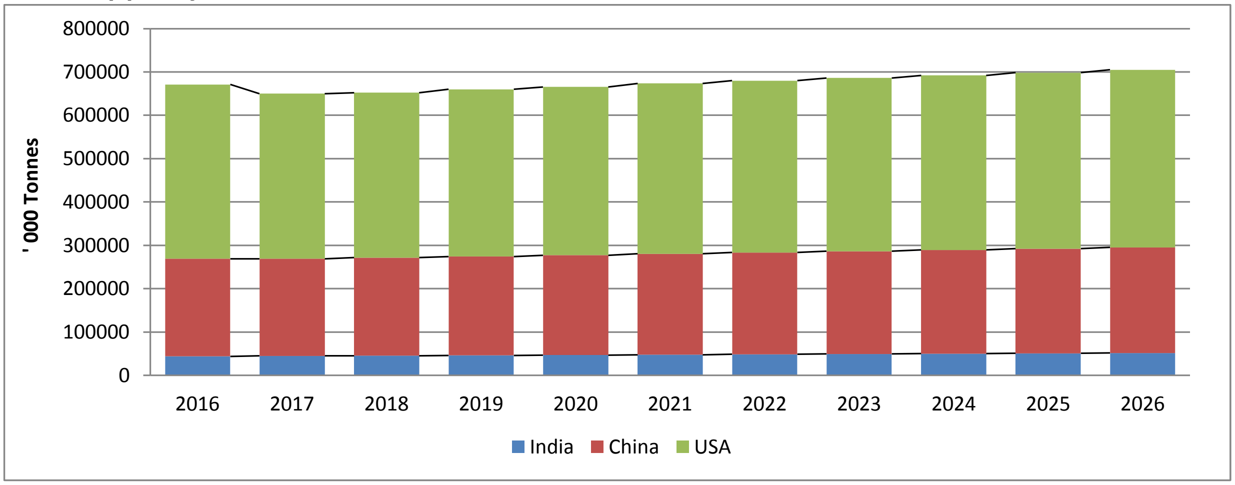

Source: OECD- FAO Agriculture Outlook 2017-26

Chart 14 (c): Projected Oilseeds Production in China, India and Canada

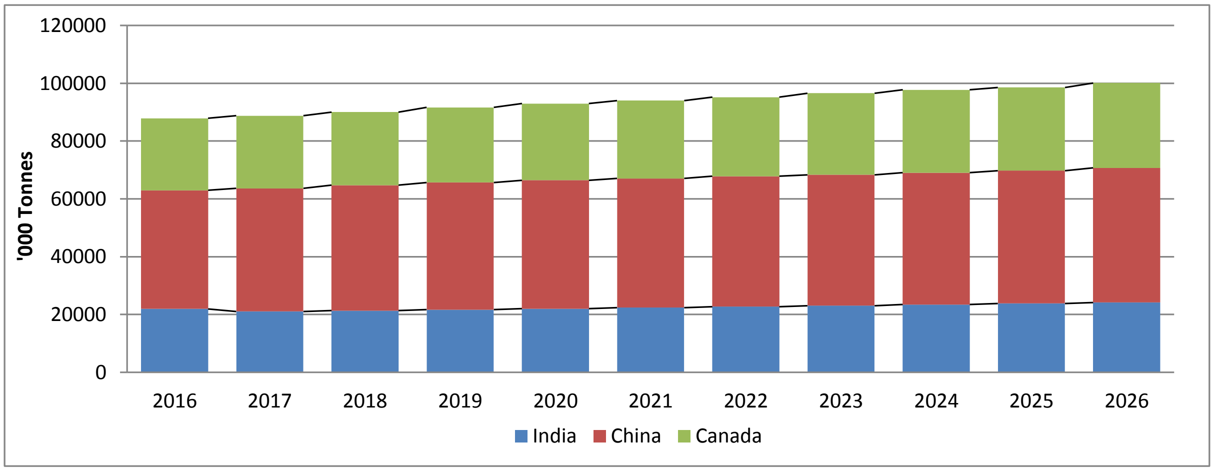

Note: Oilseeds include Soy Beans, sunflower and rapeseed/canola. Year beginning 1st August

Source: OECD- FAO Agriculture Outlook 2017-26

GLOBAL AGRICULTURAL EMISSIONS

Chart 15 (a): All GHG Emission from Agriculture sector by major Continents in 2015 (CO_2 equivalent)

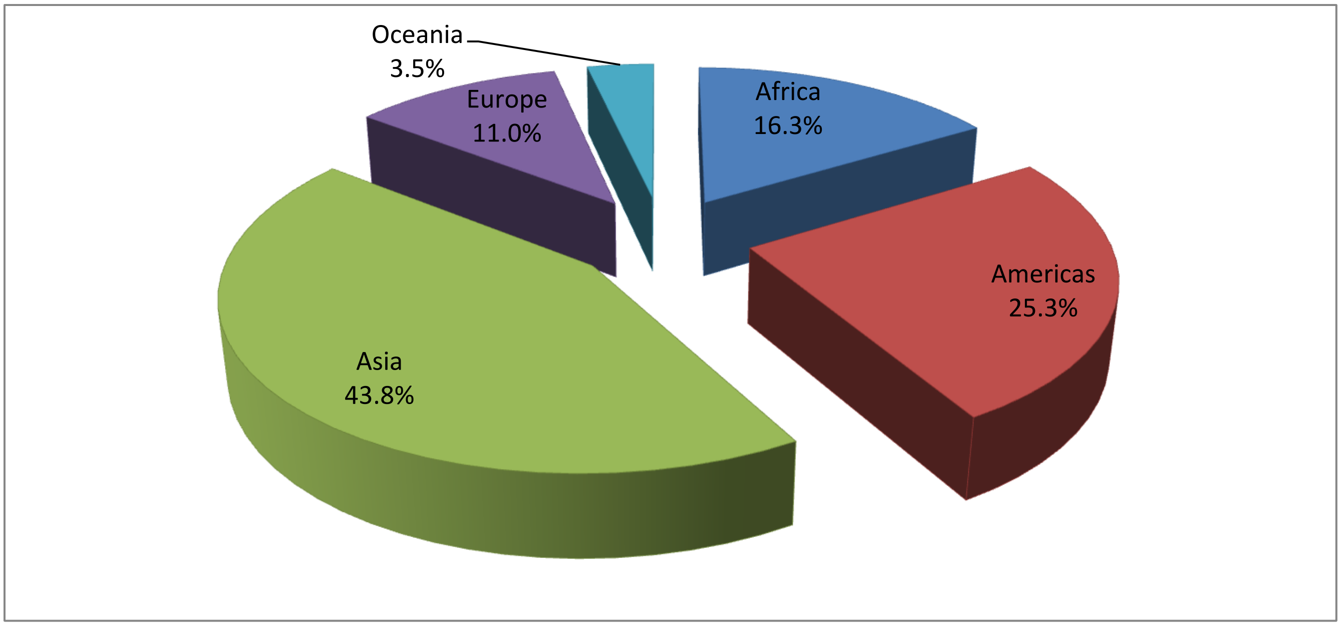

Source : FAOSTAT

Chart 15 (b): Source-wise Total GHG Emissions from Agriculture Sector in 2015

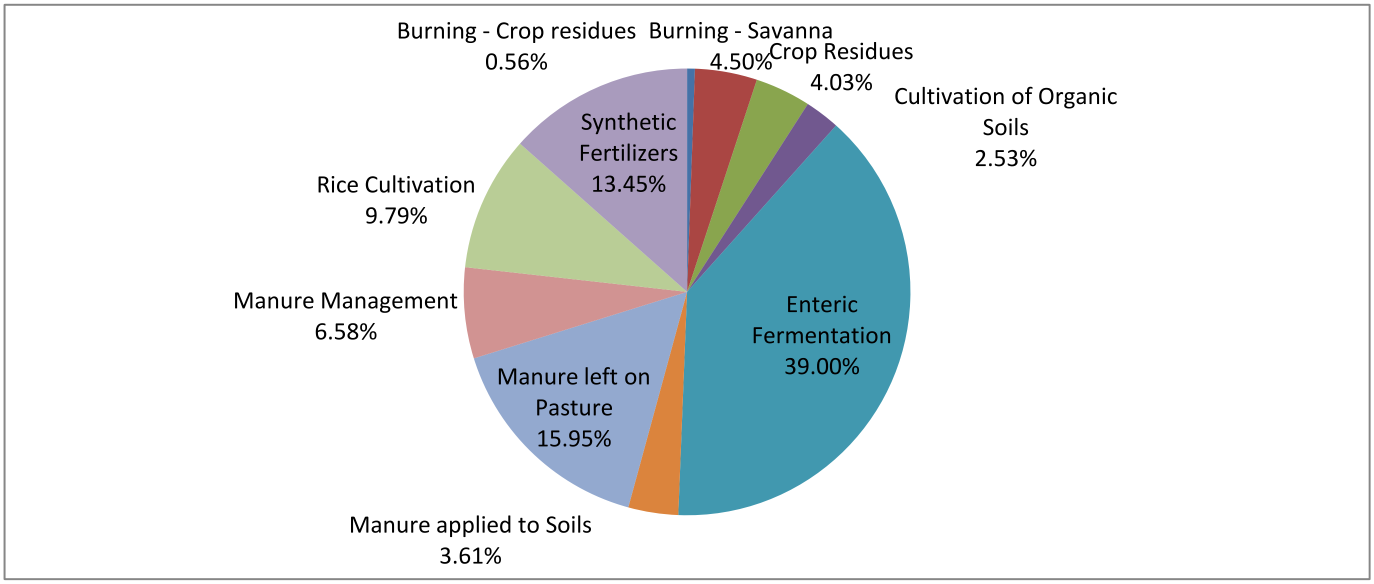

Source : FAOSTAT

APPENDICES

A.1: Explanatory Notes

Advance Estimates

The estimates of area, production and yield rates for 2016-17 are “Advance Estimates” as on 16-08-2017 and are based on deliberations held in weekly inter-disciplinary meetings of Crop Weather Watch Group, availability of water in important reservoirs in the country, availability/supply of important inputs including credit to farmers community, feedback received from states etc. These estimates are “advance” in the sense that these have been prepared in advance of availability of results of CCE (Crop Cutting Experiments) and are subject to revision as more precise information flows from states to DES.

Yield

Yield rate is defined as production per unit of area. However, yield rates of various crops given in this publication may not exactly tally with the ratio of production to the corresponding area because figures of area and production of various crops have been generally rounded off in ten thousands.

Principal Crops

Principal Crops as referred to in this publication include only those crops which are covered for preparation of index numbers.

Estimation Procedure for Non-Availability of Data for 2016-17

The index numbers of area, production and yield rate for 2015-16 (from where growth rates have been derived) are based on advance estimates of principal crops as on 16-08-2017. However, there are a number of ‘non-forecast’ crops such as plantation crops and spices for which no advance estimates for 2016-17 (or for that matter any year) are prepared. In such cases, the estimates of area and production for the previous years are repeated for the purpose of index numbers.

Nine Oilseeds

Wherever “Nine oilseeds” is mentioned in this publication, it includes Castor seed, Groundnut, Linseed, Nigerseed, Safflower, Sesamum, Soyabean, Sunflower and Rapeseed & Mustard.

Total Oilseeds

Total oilseeds include cottonseed and coconut besides nine oilseeds mentioned in the above paragraph.

Net Availability of Foodgrains

The Net availability of foodgrains is defined as, where

$$NAF = GP - SFW - e + i + s$$

NAF = Net availability of Foodgrains
GP = Gross Production of Foodgrains
SFW = Seed, feed and wastages of Foodgrains
e = Exports of Foodgrains
i = Imports of Foodgrains
s = Change in Stocks of Foodgrains

To work out per capita net availability of foodgrains in terms of kg. per year, NAF is divided by the estimated population for a particular year. When this is further divided by the number of days in a year i.e. 365 days, it gives net availability of foodgrains per day.

A.2 : Conversion Factors between Important Primary and Secondary Agricultural Commodities

Commodity	Conversion Factor
(1)	(2)
**Rice** (Cleaned) Production	2/3 of Paddy Production
**Cotton**	
Cotton Lint Production	1/3 of Kapas Production
Cotton Seed Production	2/3 of Kapas Production
	2 Times of Cotton Lint Production
**Jute**	
100 Yards of Hessian	54 lbs. of Raw Jute
4148 Yards of Hessian	1 Ton of Raw Jute
	5.55 Bales of Raw Jute (of 180 Kgs. Each)
1 Ton of Sacking	1.11 Tons of Raw Jute
	6.17 Bales of Raw Jute (of 180 Kgs. Each)
1 Ton of Hessian	1.05 Tons of Raw Jute
Sacking etc.	5.85 Bales of Raw Jute (of 180 Kgs. Each)
**Groundnut**	
Kernel to Nuts in Shell	70 Percent
Oil to Nuts in Shell	28 Percent
Oil to Kernels Crushed	40 Percent
Cake to Kernels Crushed	60 Percent
**Sesamum**	
Oil to Seeds Crushed	40 Percent
Cake to Seeds Crushed	60 Percent
**Rapseed and Mustard**	
Oil to Seeds Crushed	33 Percent
Cake to Seeds Crushed	67 Percent
**Linseed**	
Oil to seeds Crushed	33 Percent
Cake to Seeds Crushed	67 Percent
**Castorseed**	
Oil to Seeds Crushed	37 Percent
Cake to Seeds Crushed	63 Percent
**Cotton Seed**	
Oil to Seeds Crushed	14 to 18 Percent
Cake to Seeds Crushed	82 to 86 Percent
**Coconut**	
Copra to Nuts	One Ton of Copra = 6773 Nuts
Oil to Copra Crushed	62 Percent
Cake to Copra Crushed	38 Percent
**Nigerseed**	
Oil to Seeds Crushed	28 Percent
Cake to Seeds Crushed	72 Percent

(Contd.)

Commodity	Conversion Factor
(1)	(2)
**Kardi Seed**	
Oil to Seeds Crushed	40 Percent
Cake to Seeds Crushed	60 Percent
**Mahua Seed**	
Oil to Seeds Crushed	36 Percent
Cake to Seeds Crushed	64 Percent
**Neem Seed**	
Oil to Kernels Crushed	45 to 50 Percent
Cake to Kernels Crushed	50 to 55 Percent
**Soyabean Seed**	
Oil to Soyabean Seed Crushed	18 Percent
Meal to Soyabean Seed Crushed	73 Percent
Hull from Soyabean Seed Crushed	8 Percent
Wastage from Soyabean Seed Crushed	1 Percent
**Sugar**	
Gur from Cane Crushed	11.20 Percent to 11.50 Percent
Crystal Sugar from Gur Refined (Gur Refineries)	62.5 Percent
Crystal Sugar from Cane Crushed (Cane Factories)	10.20 Percent
Khandasari Sugar (Sulpher and Non-sulpher) from standard Gur Refined	46 Percent
Molasses from Cane Crushed	4.0 Percent to 4.5 Percent
Cane - Trash* from Cane Harvested	8.0 Percent to 12.0 Percent
**Lac**	
Seed Lac	66.0 Percent of Stick Lac
Shell Lac	57.4 Percent of Stick Lac
**Cashewnut**	
Cashew Kernel	25 Percent of Cashewnuts

* This consists of leaves and portion of the top of stalk which are removed from the canestalk, while harvesting and before sending the cane for milling.

A.3 : Seed Rate of Sowing for Important Field Crops

(Kg/ ha)

Crop	Variety Seed Rate	Hybrid Seed Rate
(1)	(2)	(3)
**Cereals**		
Rice	35-40	12-15 for normal planting<br>5 under System of Rice Intensification (SRI) planting
Wheat	100	
Barley	100	
Sorghum	Grain Rabi 8-10; Grain Kharif 10	7.5
Pearl Millet	4-5	42494.00
Maize	18-20	20
Ragi	10-12.5	10-12.5
**Pulses**		
Pigeon pea	Early duration 17-19<br>Medium duration 15-17<br>Long duration 12-15	
Chickpea(Large seeded)	75-100	
Chickpea Desi (small seeded)	65-75	
Chickpea Kabuli	100-120	
Mungbean	Kharif-20, Rabi 25-30, Summer 30-35	
Urdbean	Kharif-20, Rabi 25-30, Summer 30-35	
Fieldpea	80-100	
Lentil	50-60	
Rajmash	75-100	
Mothbean	15-18	
Kulthi	22-30	
**Oilseeds**		
Groundnut	140-160	
Rapeseed & Mustard	3-5	3.5
Soybean	65-75	
Sunflower	5	5
Sesame	5	
Niger	5	
Safflower	7	
Linseed	30 for seed type<br>45 for dual purpose type	
Castor	5	5

(Contd..)

Crop	Variety Seed Rate	Hybrid Seed Rate
(1)	(2)	(3)
**Forage crops**		
Sorghum	25 in small seeded varieties;<br>40 in bold seeded varieties	
Maize	30	
Guar	20	
Pearl Millet	20	
Oats	70	
Berseem	20-25	
Gobhi Sarson	4	
Lucerne	15	
**Fibre Crops**		
Cotton	10-12	Bt. Cotton - 2.25<br>Non-Bt. Cotton 10
Jute	5 (olitorius), 7 (capsularis)	
Sunhemp	25	
Mesta	12	
**Sugar Crops**		
Sugarcane	60 qtls.<br>(40,000 setts of 3 buds in sub tropics)<br>(20,000-25000 sets of 3 buds in tropics)	-

Source:- Indian Council of Agricultural Research (ICAR)

A.4: Rainfall Position in India during 2017

1) Rainfall during 2017 (January – December, 2017)

In 2017, the rainfall (January to December, 2017) over the country as a whole was 94% of Long Period Average (LPA). Out of 36 meteorological subdivisions, 29 received excess/normal rainfall and 07 received deficient rainfall. Season wise rainfall distribution over the country as a whole was as follows:

**Winter (January to February 2017):**	**95% of LPA**
**Pre-monsoon (March to May 2017):**	**98% of LPA**
**Monsoon (June to September 2017):**	**95% of LPA**
**Post-Monsoon (October to December 2017):**	**89% of LPA**

Season–wise Performance

Winter Season (January- February 2017)
During the winter season rainfall activity over the country as a whole was 95% of the LPA rainfall. At met sub-division level, 05 subdivisions received large excess/excess rainfall, 05 subdivisions received normal rainfall, 08 received deficient, 12 received large deficient rainfall and 06 received no rainfall out of 36 met sub-divisions.

Pre-Monsoon Season (March- May 2017)

Rainfall activity during the Pre-monsoon season over the country as a whole was recorded normal at 98% of LPA rainfall. Out of 36 meteorological sub-divisions, 05 received large excess/excess rainfall, 13 received normal rainfall, 15 received deficient rainfall and 03 met sub-divisions received large deficient rainfall.

Monsoon Season (June-September 2017)
During the monsoon season for the country as a whole, rainfall was 95% of its Long Period Average (LPA). Seasonal rainfall was 90% of LPA over Northwest India, 94% of its LPA over Central India, 96% of its LPA over East and Northeast (NE) India and equal to LPA in south Peninsula. At met sub-division level, out of 36 met sub-divisions, 05 met sub-divisions received excess rainfall, 25 sub-divisions received normal rainfall and 06 sub-divisions received deficient rainfall. Excess/normal rainfall sub-divisions covered 83% of the geographical area of the country. Out of 630 districts for which rainfall data were available, 23 districts (4%) received large excess rainfall, 79 districts (12%) received excess rainfall, 313 districts (50%) normal rainfall, 207 districts (33%) deficient rainfall and 08 districts (1%) received large deficient rainfall.

Post-Monsoon Season (October- December 2017)
Rainfall activity during the Post-monsoon season for the country as a whole was recorded deficit rainfall with 89% of LPA. Out of 36 meteorological sub-divisions, 05 subdivisions received large excess/excess rainfall, 14 received normal rainfall 08 received deficient rainfall and 09 met sub-divisions received large deficient rainfall.

A.5 : Major Economic Groups/ Institutions

Group/ Institution	Member Countries
(1)	(2)
Association of Southeast Asian Nations (ASEAN)	Brunei Darussalam, Cambodia, Indonesia, Lao PDR, Malaysia, Philippines, Singapore, Thailand, Vietnam
BASIC Countries	Brazil, South Africa, India and China
Bay of Bengal Initiative for Multi-Sectoral Technical and Economic Cooperation (BIMSTEC)	Bangladesh, India, Myanmar, Sri Lanka, Thailand, Bhutan, and Nepal
BRICS Economies	Brazil, Russian Federation. India, China, South Africa
Association of Southeast Asian Nations (ASEAN)	Brunei Darussalam, Cambodia, Indonesia, Lao PDR, Malaysia, Philippines, Singapore, Thailand, Vietnam
Group of Four (G-4)	Brazil, Germany, India, and Japan
Group of Eight (G-8)	Canada, France, Germany, Italy, Japan, Russia, United Kingdom, USA, European Union*
Group of Twenty (G-20)	Australia, Brazil, Canada, France, Germany, India, Indonesia, Italy, Japan, Mexico, Russian Federation, South Africa, Turkey, United Kingdom, United States, Argentina, China, South Korea, European Union, Saudi Arabia
South Asian Association for Regional Cooperation (SAARC)	Afghanistan, Bangladesh, Bhutan, India, Maldives, Nepal, Pakistan, Sri Lanka

* EU is represented within the G8 but cannot host or chair summits

Note: Since 2014, the G8 effectively comprises seven nations and the European Union as the eighth member.

© Directorate of Economics & Statistics, Government of India

Government of India
Ministry of Agriculture & Farmers Welfare
Department of Agriculture, Cooperation & Farmers Welfare
Directorate of Economics & Statistics
New Delhi
