# Supplementary material for: Averting wheat blast by implementing a ‘wheat holiday’: In search of alternative crops in West Bengal, India
Source: PLoS One. 2019 Feb 20;14(2):e0211410. doi: 10.1371/journal.pone.0211410 (PMC6382110; doi:10.1371/journal.pone.0211410)
Supplement: S5 File — (PDF) [file pone.0211410.s005.pdf]

सत्यमेव जयते

Annual Report

2016-17

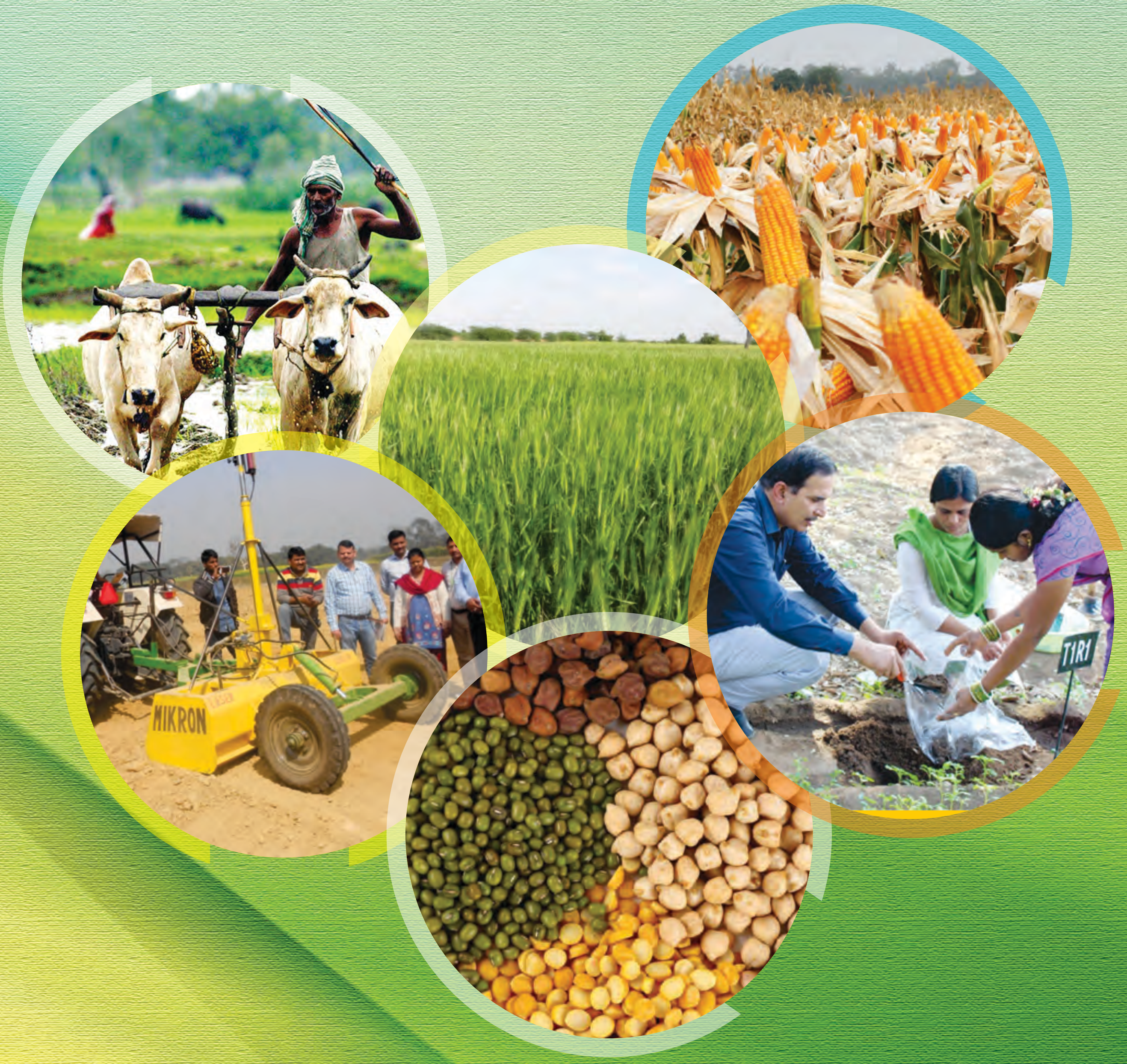

DEPARTMENT OF AGRICULTURE, COOPERATION & FARMERS WELFARE

Ministry of Agriculture & Farmers Welfare

Government of India

Krishi Bhawan, New Delhi-110 001

ANNUAL REPORT
2016-17

सत्यमेव जयते

DEPARTMENT OF AGRICULTURE, COOPERATION & FARMERS WELFARE
Ministry of Agriculture & Farmers Welfare
Government of India
Krishi Bhawan, New Delhi-110 001
www.agricoop.nic.in

Contents

S. No.	Chapter	Page No.
1.	Overview	1
2.	Functions and Organisational Structure	8
3.	Directorate of Economics & Statistics (DES)	11
4.	National Food Security Mission (NFSM)	22
5.	Mission for Integrated Development of Horticulture (MIDH)	27
6.	National Mission on Oilseeds and Oil Palm (NMOOP)	43
7.	National Mission for Sustainable Agriculture (NMSA)	49
8.	National Mission on Agricultural Extension &Technology (NMAET)	65
9.	National Crop Insurance Programme (NCIP)	99
10.	Integrated Scheme on Agriculture Census & Statistics	105
11.	Agricultural Marketing	107
12.	Agricultural Cooperation	117
13.	Rashtriya Krishi Vikas Yojana (RKVY)	126
14.	Drought Management	132
15.	International Cooperation	133
16.	Agricultural Trade	139
17.	Agricultural Credit	143
18.	Gender Perspective in Agriculture	147
	Annexures	157

Chapter 1

Overview

1.1 Agriculture plays a vital role in India's economy. 54.6% of the population is engaged in agriculture and allied activities (census 2011) and it contributes 17% to the country's Gross Value Added (current price 2015-16, 2011-12 series). Given the importance of agriculture sector, Government of India took several steps for its sustainable development. Steps have been taken to improve soil fertility on a sustainable basis through the soil health card scheme, to provide improved access to irrigation and enhanced water efficiency through Pradhan Mantri Krishi Sinchai Yojana (PMKSY), to support organic farming through Paramparagat Krishi Vikas Yojana (PKVY) and to support for creation of a unified national agriculture market to boost the income of farmers. Further, to mitigate risk in agriculture sector a new scheme "Pradhan Mantri Fasal Bima Yojana (PMFBY) has been launched for implementation from Kharif 2016.

1.2 As per the land use statistics 2013-14, the total geographical area of the country is 328.7 million hectares, of which 141.4 million hectares is the reported net sown area and 200.9 million hectares is the gross cropped area with a cropping intensity of 142 %. The net sown area works out to be 43% of the total geographical area. The net irrigated area is 68.2 million hectares.

Agriculture Gross Value Added (GVA)

1.3 Central Statistics Office (CSO), Ministry of Statistics & Programme Implementation has released the New Series of National Accounts, based upon revising the base year from 2004-05 to 2011-12. As per the Provisional Estimates released by CSO on 31.05.2016, the Agriculture and Allied sector contributed approximately 17.0% of India's Gross Value Added (GVA) at current prices during 2015-16. Gross Value Added (GVA) of Agriculture and Allied sector and its share in total GVA of the country during the last 3 years including the current year, at current prices of 2011-12 series is as follows:

1.4 There has been a continuous decline in the share of Agriculture and Allied sector in the GVA from 18.2 percent in 2012-13 to 17.0 percent in 2015-16 at current prices. Falling share of Agriculture and Allied sector in GVA is an expected outcome in a fast growing and structurally changing economy.

Share of Agriculture & Allied Sectors in Total GVA

(Rs. in Crore)

Items	Year			
	2012-13	2013-14	2014-15	2015-16
GVA of Agriculture and Allied Sectors	1680797	1902452	1995251	2093081
Per cent to total GVA	18.2	18.3	17.4	17.0

Source: Central Statistics Office, Ministry of Statistics and Programme Implementation, Govt. of India.

1.5 Growth (over the previous year) in the Total GVA of the Economy and that in the GVA of Agriculture and Allied sector at 2011-12 basic prices is given below:

Growth in Gross Value Added (at 2012-13 basis prices)

(In percent)

Period	Total GVA	Agriculture & Allied Sector GVA
2012-13	5.4	1.5
2013-14	6.3	4.2
2014-15	7.1	-0.2
2015-16	7.2	1.2

Source: Central Statistics Office.

1.6 The Agriculture and Allied sector witnessed a growth of 1.5 per cent in 2012-13, 4.2 per cent in 2013-14, -0.2 per cent in 2014-15 and 1.2 in 2015-16 at 2011-12 basic prices.

Rainfall 2016-17

Monsoon Rainfall (June – September)

1.7 In the Year 2016, the cumulative monsoon rainfall for the country as a whole during 1st June to 30th September, 2016 was 3% lower than Long Period Average (LPA). Rainfall in the four broad geographical divisions of the country during the above period was higher than LPA by 6% in Central India but lower than LPA by 11% in East & North East India, 8% in South Peninsula and 5% in North West India. Out of 36 met sub-divisions, 27 met sub-divisions have received excess/normal rainfall and 9 met sub-divisions received deficient rainfall. Out of 629 districts, 111 (18%) districts received excess, 314 (50%) districts received normal, 189 (30%) districts received deficient and 15 (2%) districts received scanty rainfall during the above period.

Post-Monsoon (October - December)

1.8 During the post-monsoon season (1st October to 31st December, 2016) the cumulative rainfall for the country, as a whole, has been 45% lower than the LPA. Out of 36 meteorological subdivisions, 03 subdivisions received large excess rainfall, 07 received Excess normal rainfall, 13 received deficient rainfall and 13 received large deficient scanty rainfall.

Production Scenario 2015-16

1.9 Notwithstanding the setback in kharif crops 2015-16 due to bad monsoon and Rabi crops due to warm winter, total foodgrain production in the country increased marginally in 2015-16. As per 4th Advance Estimates for 2015-16, total production of rice is estimated at 104.32 million tonnes which is lower by 1 .17 million tonnes than the production of 105.48 million tonnes during the preceding year. Production of wheat, estimated at 93.50 million tonnes, is higher by 6.97 million tonnes than the production of 86.53 million tonnes achieved during 2014-15. Total production of Coarse Cereals estimated at 37.94 million tonnes is lower by 4.38 million tonnes than their production during 2014-15.

1.10 Total foodgrains production in the country is estimated at 252.22 million tonnes which is marginally higher by 0.20 million tonnes than the previous year's foodgrains production of 252.02 million tonnes. Production of pulses estimated at 16.47 million tonnes is lower by 0.68 million tonnes than their production during 2014-15. With a decline of 2.21 million tonnes over the last year, total production of oilseeds in the country is estimated at 25.30 million tonnes. Production of sugarcane is estimated at 352.16 million tonne. Total production of cotton estimated at 30.15 million bales (of 170 kgs each) is lower than previous year's production of 34.81 million bales and also lower by 4.48 million bales than its five years average production of 34.63 million bales. Production of jute & mesta estimated at 10.47

million bales (of 180 kg each) is lower by 0.66 million bales (6.3%) than their production during the previous year.

2015-16 to 136.09 lakh hectares in 2016-17. Consequently, the total area coverage under Kharif foodgrains has increased to 715.54 lakh hectares as compared to 687.58

Area, production and yield of major Crops

Crops	Area (Lakh hectare)			Production (Million Tonnes)			Yield (kg/hectare)		
	2013-14	2014-15	2015-16*	2013-14	2014-15	2015-16*	2013-14	2014-15	2015-16*
Rice	441.36	441.10	433.88	106.65	105.48	104.32	2416	2391	2404
Wheat	304.73	314.65	302.27	95.85	86.52	93.50	3145	2750	3093
Coarse cereals	252.19	251.70	237.75	43.29	42.86	37.93	1717	1703	1596
Pulses	252.12	235.54	252.59	19.25	17.15	16.47	764	728	652
Foodgrains	1250.41	1243.00	1226.50	265.04	252.02	252.22	2120	2028	2056
Oilseeds	280.50	255.96	261.34	32.74	27.51	25.30	1168	1075	968
Sugarcane	49.93	50.66	49.53	352.14	362.33	352.16	70522	71512	71095
Cotton@	119.60	128.19	118.72	35.90	34.80	30.15	510	462	432
Jute & Mesta#	8.38	8.09	7.85	11.69	11.12	10.47	2512	2473	2399

* 4th advance estimates @ Production in million bales of 170 kg each.

# Production in million bales 180 Kg. each.

Production Scenario during Kharif 2016-17

1.11 As result of good monsoon rainfall in the country, area coverage under Kharif coarse cereals has increased to 186.68 lakh hectares as compared to 178.62 lakh hectares during 2015-16 which was a monsoon-deficit year. Further, area coverage under pulses has increased from 113.61 lakh hectares in lakh hectares last year, i.e. an increase of 27.96 lakh hectares. A comparative position of production of Food grains, Oilseeds, Sugarcane and Cotton during kharif 2016-17 vis-à-vis kharif 2015-16 is given below:

1.12 As per the First Advance Estimates, total production of Kharif Foodgrains during 2016-17 is estimated at record 135.03 million tonnes which is higher by 11.02 million tonnes

Kharif production in 2015-16 and 2016-17

(Million Tonnes)

Crop	2015-16 (4th Advance estimates)	2016-17 (1st Advance estimates)	Absolute difference	Percentage Increase/decrease
Foodgrains	124.01	135.03	11.02	8.89
Oilseeds	16.59	23.36	6.77	40.81
Sugarcane	352.16	305.25	-46.91	-13.32
Cotton@	30.15	32.12	1.97	6.53

@ Production in million bales of 170 kg each.

Source: Directorate of Economics & Statistics, Department of Agriculture, Cooperation & Farmers Welfare

as compared to last year's Kharif foodgrains production of 124.01 million tonnes. Total production of Kharif rice is estimated at record level of 93.88 million tonnes which is higher by 2.57 million tonnes more than the last year's production of 91.31 million tonnes. Total production of coarse cereals in the country has increased to 32.45 million tonnes as compared to 27.17 million tonnes during 2015-16 (4th Advance Estimates). The production of kharif pulses estimated at 2.54 million tonnes more than the last five years' average production.

Capital Formation in Agriculture and Allied Sector:

1.13 Gross Capital Formation (GCF) in Agriculture and Allied sector is estimated separately for public, private corporate and the household sectors. Items included in the estimates of GCF are:

(i) Improvement of land and Irrigation works

(ii) Laying of new orchards and plantations

(iii) Purchase of agricultural machinery and implements

(iv) Agriculture construction works

(v) Additions to livestock

(vi) Fishing boats and nets etc.

1.14 Gross Capital Formation (GCF) in Agriculture and Allied sector relative to GVA in this sector has been showing a fluctuating trend from 18.3 per cent in 2011-12 to 16.2 per cent in 2014-15.

National Policy for Farmers (NPF), 2007:

1.15 Government of India approved the National Policy for Farmers (NPF) in 2007. The Policy provisions, inter alia, include asset reforms in respect of land, water, livestock, fisheries, and bio-resources; support services and inputs like application of frontier technologies; agricultural bio-security systems; supply of good quality seeds and disease-free planting material, improving soil fertility and health, and integrated pest management systems; support services for women like crèches, child care centres, nutrition, health and training; timely, adequate, and easy reach of institutional credit at reasonable interest rates, and farmer-friendly insurance instruments; use of Information and Communication Technology (ICT) and setting up of farmers' schools to revitalize agricultural extension; effective implementation of MSP across the country, development of agricultural market infrastructure, and rural non-farm employment initiatives for farm households; integrated approach for rural energy, etc.

Gross Capital Formation (GCF) in Agriculture and Allied sector relative to Gross Value Added (GVA) at 2011-12 basic prices.

(Rs.in crore)

Period	GCF in Agriculture & Allied Sectors			GVA in Agriculture & Allied Sectors	GCF as percentage of GVA		
	Public	Private	Total		Public	Private	Total
2011-12	35715	238717	274432	1501816	2.4	15.9	18.3
2012-13	36078	217230	253308	1524398	2.4	14.3	16.6
2013-14	32472	244694	277165	1588237	2.0	15.4	17.5
2014-15 *	36061	220434	256495	1584293	2.3	13.9	16.2

Source: Central Statistics Office, MOSPI

*As per Advance Estimates for 2015-16 (latest available) released on 8.2.2016

1.16 Many of the provisions of the NPF are being operationalised through various schemes and programmes which are being implemented by different Central Government Departments and Ministries. For the operationalisation of the remaining provisions of the Policy, an Action Plan has been finalized and circulated to the Ministries and Department concerned, as well as to all States and UTs for necessary follow up action. An Inter-Ministerial Committee constituted for the purpose also monitors the progress of the Plan of Action for the operationalisation of the NPF.

1.17 To achieve the target of doubling of income of farmers by 2022, as announced during presentation of Budget, 2016-17, a Committee has been constituted under the Chairmanship of Additional Secretary, Department of Agriculture, Cooperation and Farmers Welfare to examine the various issues involved. All the State Governments have been requested to hold across-the–board deliberations and prepare strategy for the states that will lead to doubling the income of the farmers. NABARD has also organized six Regional Conferences to sensitize all the States for the strategy that will lead to doubling the income of farmers by 2022.

1.18 The strategy of the Government is to focus on farmers' welfare by making farming viable. Farm viability is possible, when cost of cultivation is reduced, yield per unit of farm is increased and farmers get remunerative prices on their produce. The Department is implementing various schemes to meet this objective.

Pradhan Mantri Krishi Sinchayee Yojana (PMKSY):

1.19 The scheme has been approved with an outlay of Rs. 50,000 crore for a period of 5 years (2015-16 to 2019-20). The major objective of PMKSY is to achieve convergence of investments in irrigation at the field level, expand cultivable area under assured irrigation, improve on-farm water use efficiency to reduce wastage of water, enhance the adoption of precision irrigation and other water saving technologies (More crop per drop), promote sustainable water conservation practices etc. Cabinet decision has been made in July, 2016 for implementation of PMKSY on mission mode approach. The mission is administered by Ministry of Water Resources, River Development and Ganga Rejuvenation.

1.20 During 2016-17, as on 31st December, 2016, an amount of Rs. 1211.75 cr and Rs.430.30 crore has been released to states for implementation of Micro-irrigation activities and for Other Interventions activities respectively. Till December, 2016, an area of about 3.54 lakh ha. has been covered under Micro Irrigation.

Agriculture Credit

1.21 Government announces annual target for agriculture credit in the budget every year. Agricultural credit flow has shown consistent progress every year. Agriculture Credit of Rs. 711,621 crore was provided to the farmers against target of Rs.7,00,000 crore in 2013-14. In the year 2014-15, agriculture credit flow was Rs. 845,328.23 crore against the target of Rs.8,00,000 crore. Target for the year 2015-16 was fixed at Rs. 850,000 crore and achievement is Rs. 877,224 crore. The Target for the year 2016-17 has been fixed at Rs. 9,00,000 crore and a sum of Rs. 755,995.17 crore has been disbursed as agriculture credit during April-September, 2016.

Interest Subvention Scheme

1.22 The Government is providing interest subvention to make short-term crop loans upto Rs. 3 lakh for a period of one year available to farmers at the interest rate of 7%

per annum and in case of timely repayment, the same gets reduced to 4%.

Kisan Credit Card

1.23 The Kisan Credit Card Scheme is in operation throughout the country and is implemented by Commercial Banks, Cooperative Banks and Regional Rural Banks (RRB). The scheme has facilitated in augmenting credit flow for agricultural activities. The scope of the KCC has been broad-based to include term credit and consumption needs. The KCC Scheme has since been simplified and converted into ATM enabled debit card with, inter-alia, facilities of one-time documentation, built-in cost escalation in the limit, any number of drawals within the limit etc. which eliminates the need for disbursement through camps and mitigates the vulnerability of farmers to middlemen.

Crop Insurance

1.24 In order to protect farmers against crop failure due to natural calamities, pests & diseases, weather conditions, Government of India had introduced the National Crop Insurance Programme (NCIP) with component schemes of Modified National Agricultural Scheme (MNAIS), Weather Based Crop Insurance Scheme (WBCIS) and Coconut Palm Insurance Scheme (CPIS). In addition, National Agricultural Insurance Scheme (NAIS) which was to be withdrawn after implementation of NCIP from Rabi 2013-14 was extended further upto 2015-16.

1.25 The erstwhile crop insurance schemes have recently been reviewed in consultation with various stakeholders including States/ UTs. As a result of the review, a new scheme **"Pradhan Mantri Fasal Bima Yojana (PMFBY)** has been approved for implementation from Kharif 2016 along with restructured pilot Unified Package Insurance Scheme (UPIS) and Weather Based Crop Insurance Scheme (WBCIS). Under the PMFBY & RWBCIS, a uniform maximum premium of only 2% will be paid by farmers for all Kharif crops and 1.5% for all Rabi crops. In case of annual commercial and horticultural crops, the maximum premium to be paid by farmers will be only 5%. The premium rates to be paid by farmers are very low and balance premium will be paid by the Government, to be shared equally by Central and State Government, to provide full insured amount to the farmers against crop loss on account of natural calamities.

Commission for Agricultural Costs and Prices:

1.26 Commission for 'Agricultural Costs and Prices' (CACP), set up with a view to evolve a balanced and integrated price structure, is mandated to advice on the price policy (MSP) of 23 crops. These include seven cereal crops (paddy, wheat, jowar, bajra, maize, ragi and barley), five pulse crops (gram, tur, moong, urad and lentil), seven oilseeds (groundnut, sunflower seed, soyabean, rapeseed-mustard, safflower, nigerseed and seasmum), copra (dried coconut), cotton, raw jute and sugarcane {Fair and Remunerative prices (FRP)}. CACP submits its recommendations to the government in the form of Price Policy Reports every year, separately for five groups of commodities namely Kharif crops, Rabi crops, Sugarcane, Raw Jute and Copra. Before preparing these five pricing policy reports, the Commission seeks views of various state governments, concerned National organizations and Ministries.

Determinants of MSP:

1.27 Cost of production (CoP) is one of the important factors in the determination of MSP of mandated crops. Besides cost, the Commission considers other important factors such as demand and supply, price trend in the domestic and international

markets, inter-crop price parity, terms of trade between agricultural and non-agricultural sectors and the likely impact of MSPs on consumers, in addition to ensuring rational utilization of natural resources like land and water. Thus, pricing policy is rooted not in 'cost plus' approach, though cost is an important determinant of MSP.

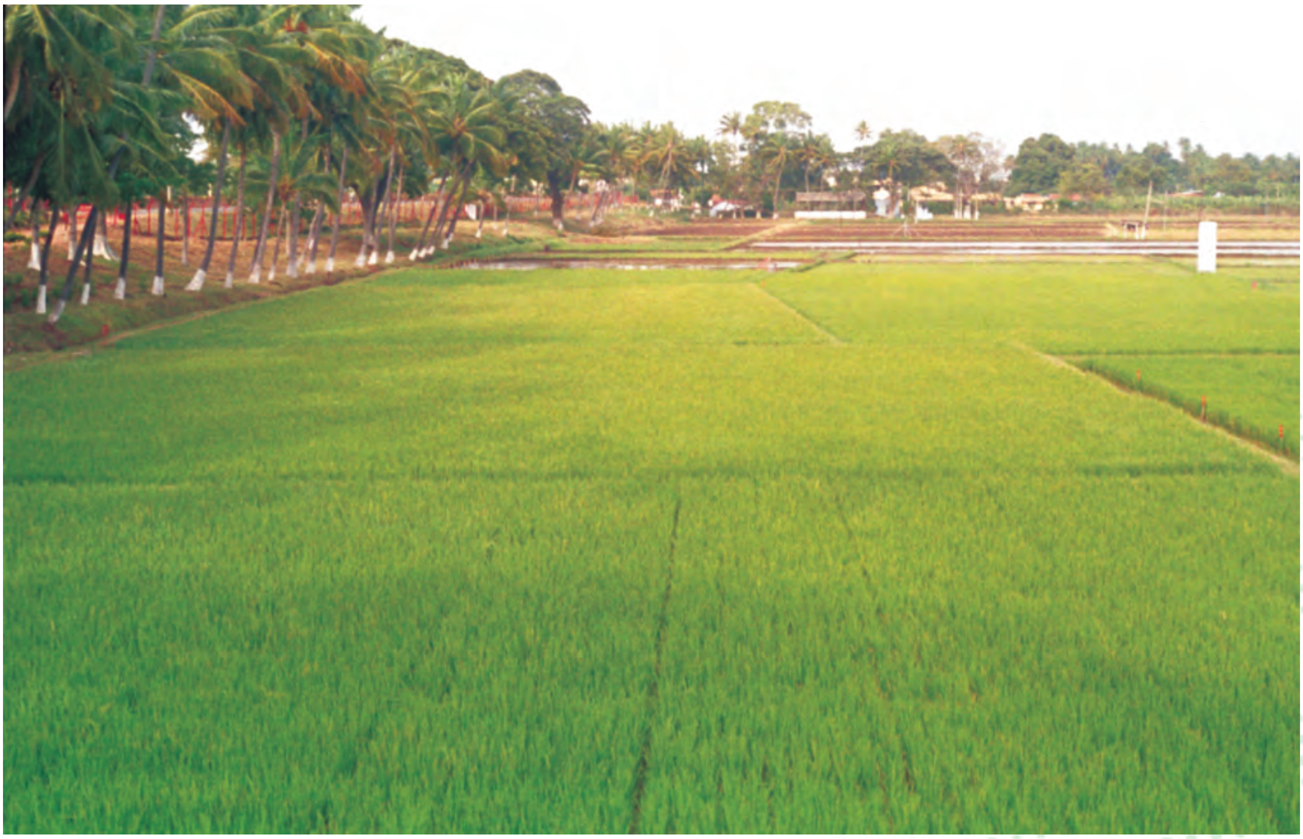

******

Chapter 2

Functions and Organizational Structure

2.1 Structure: The Department of Agriculture, Cooperation & Farmers Welfare (DAC&FW) is one of the three constituent Departments of the Ministry of Agriculture & Farmers Welfare, the other two being Department of Animal Husbandry, Dairying & Fisheries (DAHD&F) and Department of Agricultural Research and Education (DARE). This Department is headed by Agriculture & Farmers Welfare Minister and is assisted by three Minister of State. The Secretary (AC & FW) is the administrative head of the Department. The Secretary is assisted by one Principal Adviser, five Additional Secretaries including one Financial Adviser, Agriculture Commissioner, 13 Joint Secretaries including Mission Director (Mission on Integrated Development of Horticulture) & Mission Director (National Mission on Sustainable Agriculture), Horticulture Commissioner, Horticulture Advisor, Economic Advisor and Deputy Director General. In addition, Chairman of Commission for Agriculture Costs and Prices (CACP) advises Department on pricing policies for selected agricultural crops.

2.2 The DAC&FW is organized into 27 divisions **(Annexure-2.1)** and has five attached offices and twenty-one subordinate offices **(Annexure-2.2)** which are spread across the country for coordination with state level agencies and implementation of Central Sector Schemes in their respective fields. Further, one Public Sector Undertaking, eight autonomous bodies, ten national-level cooperative organizations and two authorities are functioning under administrative control of Department **(Annexure-2.3).**

2.3 Administrative Improvements: An Administrative Vigilance Unit (AVU) functions in the Department under a Joint Secretary, designated as Chief Vigilance Officer (CVO), to ensure a transparent, clean and corruption free work environment through surveillance, preventive and punitive measures. The complaints/allegations received in the unit are authenticated as per the direction of Central Vigilance Commission (CVC) and after authentication the complaints/allegations are reviewed through meetings, reports/returns etc. and necessary follow-up action is taken. Further, identification of sensitive posts in DAC&FW is also undertaken as per the guidelines of CVC to enable periodical rotation of staff posted in these posts by competent authority. AVU prepares list of Officers of doubtful integrity and Agreed List in consultation with CBI. This year Vigilance Awareness week was observed from 31st October to 5th November, 2016.

2.4 A Public Grievance Cell has been set up and is fully functional in the Department of Agriculture, Cooperation & Farmers Welfare under the Joint Secretary (Administration) who not only acts as Grievance Officer of the Department but also nominated as nodal officer for monitoring redressal of public grievances received in the Department at Headquarters. One Director has been nominated as Staff Grievance Officer to deal with grievances of the employees working in the Department of Agriculture, Cooperation & Farmers Welfare for this purpose. Similar arrangements have been made at the level of all Attached and Subordinate Offices and

all organizations under the administrative control of this Department in order to ensure expeditious redressal of grievances. During the year 2016-17 (from 01.04.2016 to 31.12.2016) 4050 public grievance petitions/ suggestions have been received and 906 cases were carried forward from previous year, out of which 4220 cases have been disposed of and 736 cases were pending in this Department at the end of December, 2016.

2.5 A SEVOTTAM Compliant "Citizens'/ Clients' Charter" has been prepared as per latest instructions/guidelines issued by Cabinet Secretariat and Department of Administrative Reforms and Public Grievances. The Citizens'/ Clients' Charter is available on the website of the Department (www.agricoop.nic.in).

2.6 Implementation of the Right to Information Act, 2005:

During year 2016-17 (as on 19-12-2016), 4737 applications (including physical and online) and 204 appeals and 20 CIC Hearing cases seeking information under the Right to Information Act, 2005 were received in RTI/ IFC and replies were sent to applicants in time.

2.7 Information and Facilitation Counter:

This counter provides information in respect of Department of Agriculture, Cooperation & Farmers Welfare and D/o Animal Husbandry, Dairying & Fisheries under the Ministry of Agriculture & Farmers Welfare. During the year 2016-17, various visitors from among NGOs as well as from general public visited the counter to obtain information. Numerous telephone calls were also received in RTI/IFC from general public to obtain information pertaining to DAC&FW and D/o AHD&F of Ministry of Agriculture & Farmers Welfare.

2.8 Progressive use of Hindi:

The Department has an Official Language Implementation Committee (OLIC), chaired by Joint Secretary (Administration), to monitor the implementation of the Official Language Policy of the Union and progressive use of Hindi in the official work of the Department. During the year under review, quarterly meetings of the Official Language Implementation Committee were held regularly. In accordance with the guidelines issued by the Department of Official Language, a Joint Hindi Salahkar Samiti for all the three departments of the Ministry of Agriculture and Farmers Welfare has been reconstituted in consultation with the Department of Official Language under the Chairmanship of the Honourable Agriculture and Farmers Welfare Minister.

2.9 The Hindi Division continued to review the position of the progressive use of Hindi in the Department and subordinate offices regularly, through quarterly progress reports and inspections. Besides, officers of the Hindi Division also participated in the meetings of the Official Language Implementation Committees of the Attached and Subordinate offices, Corporations, etc., and extended necessary guidance to them in the implementation of Official Language Act and Rules. In addition to this, offices under the control of this Department, wherein 80 per cent of the officers and employees have acquired working knowledge of Hindi, was notified in the Gazette of India under Rule 10 (4) of the Official Language Rules, 1976.

2.10 Every year, the Department nominates Clerks and Stenographers for training in Hindi Shorthand and Typing, under the Hindi Teaching Scheme of the Department of Official Language.

2.11 Five Employees have been given Cash awards for doing original noting and

drafting in Hindi under the incentive scheme for promoting the use of Hindi in the official work of the Department.

2.12 With a view to create awareness regarding the use of Hindi in the official work of the Department, a Hindi Fortnight was held from the 1-15 September 2016. On this occasion, the Honourable Minister for Agriculture and Farmers Welfare issued an appeal to all officers and staff of the Department of Agriculture, Cooperation and Farmers Welfare to do more of their official work in Hindi. During the Hindi Fortnight, various Hindi competitions, such as essay writing, noting and drafting, translation and vocabulary, poetry recitation, debate and dictation were organized, and a large number of officers and employees participated in these competitions, cash awards and certificates of appreciation were given to the winners of these competitions.

2.13 The Second Sub-Committee of the Committee of Parliament on Official Language conducted inspection of nine offices of the Department of Agriculture, Cooperation and Farmers Welfare to review the position regarding the progressive use of Hindi in official work during the year. The officers of this Department were also present at these inspection meetings.

2.14 Employees possessing a working knowledge for proficiency in Hindi were deputed to undergo training in Hindi workshops organized by the Hindi Division from time to time during the year under report.

2.15 Reservation for Scheduled Castes/ Scheduled Tribes /Other Backward Castes:

Department of Agriculture and Cooperation continued its endeavour for strict implementation of the orders issued by the Government of India from time to time, regarding reservation in services for SCs, STs, OBCs, minorities, ex-servicemen and physically disabled persons.

2.16 Prevention of Harassment of Women Employees:

A complaints committee regarding prevention of sexual harassment of women at their work place was reconstituted by the Department. This committee is chaired by a senior lady officer of the Department. The committee is represented by 5 members (including chairman), which comprises of 5 women members, (2 of these belongs to two NGO) and one male member of the Department. Three meetings of the Committee were held during the year.

2.17 The Internal Complaint Committee (ICC) of this Department has provided their Report according to section 21 of the abovementioned act as follows:

No. of complains received	No. of complaints disposed of	No. of cases pending for more than 90 days	No. of workshops/ programmes carried out	Nature of action taken by the employer
1	1	Nil	Nil	Nil

******

Chapter 3

Directorate of Economics & Statistics

3.1 Directorate of Economics and Statistics, an attached office of DAC&FW, is guided by the vision of enriching economic and statistical data and analytical inputs required by the Department of Agriculture, Cooperation & Farmers Welfare for providing better agricultural economic development policy. Its mission is to provide important statistics on area, production and yield of principal crops, Minimum Support Prices, to implement schemes related to improvement of agricultural statistics, and carrying out agro-economic research. The main objectives of the Directorate are generation and dissemination of agricultural statistics and research and analysis. The Directorate provides inputs to DAC&FW, CACP and also places a large volume of data and information in the public domain for use of all stakeholders.

Major Programmes/Activities

Agricultural Statistics Division

3.2 The DES releases four advance estimates and final estimates of area, production and yield in respect of major food grains, oilseeds, sugarcane and fibers. This is based on the information received from State Agricultural Statistics Authorities (SASAs) which have been designated the nodal agencies responsible for the collection/compilation of Agriculture Statistics in the States. In addition to the inputs received from SASAs, the Division also utilizes the forecast of MNCFCC, SAC, IEG, etc. for related crops to firm up estimates. The first advance estimates are released in the month of September. Second advance estimates are released in the month of February. By this time, the assessment for first advance estimates of Rabi crops is also available. The third advance estimates are released in April when the State Governments are ready with better assessment of production for both Kharif and Rabi crops. The fourth advance estimates are released in July. By this time, most of the Rabi crops are harvested and SASAs are also in a position to supply the yield estimates of both Kharif and Rabi seasons based on reasonable number of Crop Cutting Experiments. The final estimates are released in the month of February of the following year. The Fourth Advance Estimates for 2015-16 were released on 02.08.2016 **(Annexure 3.1)** and First Advance Estimates for 2016-17 were released on 22.09.2016 **(Annexure 3.2).**

Hravesting the experimental plot for crop cutting

3.3 The Agricultural Statistics Division organizes a National Workshop on Improvement of Agricultural Statistics every two years. Officers from the State Department of Agriculture, Revenue, Economics and Statistics, Horticulture, Agricultural Marketing and Central Ministries/Organizations participate in the Workshop. The Workshop deliberates on the ways and means to improve the collection, compilation, dissemination

Hravesting the experimental plot for crop cutting

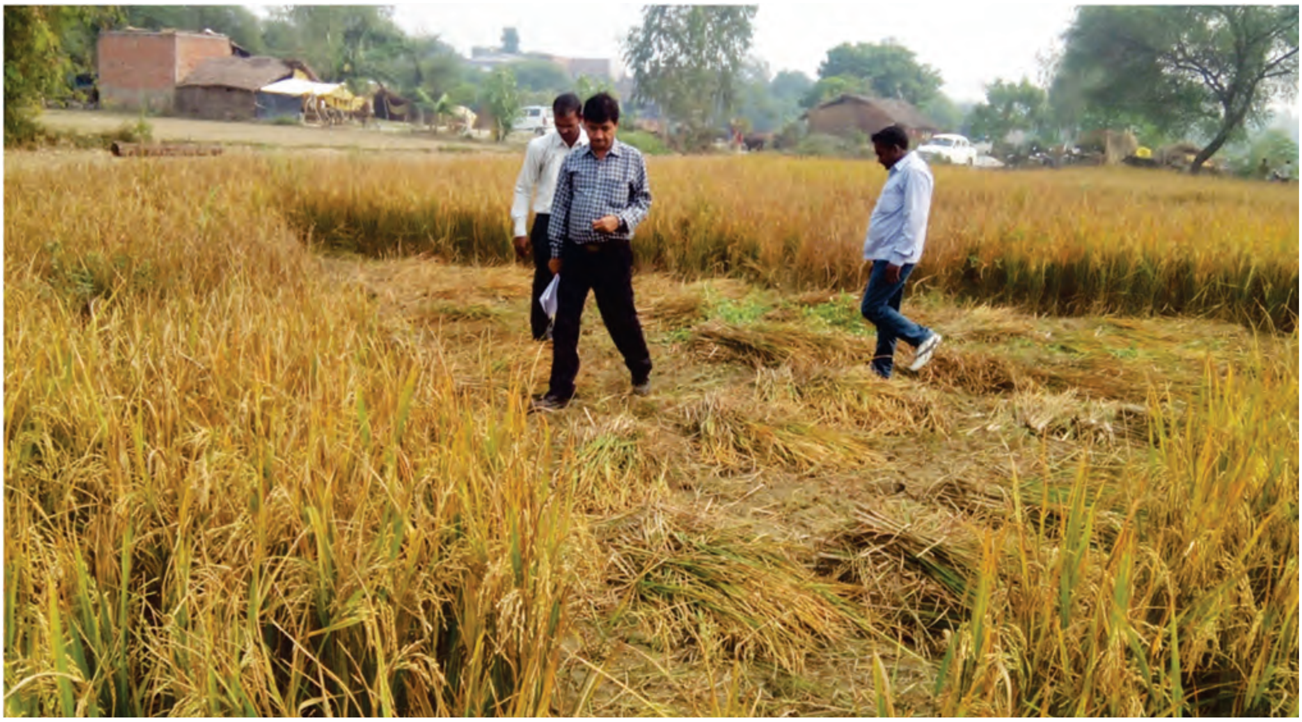

Weighing the Produce

and use of agricultural statistics and the changes required in the light of changing characteristics of agriculture with a focus on improving quality, timeliness and reliability of data.

3.4 In addition to this, the Directorate coordinates with international bodies such as the FAO on global efforts to improve agricultural statistics. It is also responsible for providing necessary data to the CSO for GDP compilation and meets international obligations related to standards of data dissemination. It contributes information on market prices of agricultural commodities for compilation of monthly WPI of the country.

Cost Study (CS) Division

3.5 Cost Study Division is mainly responsible for implementation and monitoring of Comprehensive Scheme for Studying the Cost of Cultivation of Principal Crops in India and other issues relating to this scheme including answering parliament questions, generating cost of cultivation and production estimates and providing to CACP for the purpose of arriving at the recommendations of the Minimum Support Prices (MSP) etc.

3.6 Cost of cultivation surveys are an important data source for decision making on sectors of national importance. These are very intensive surveys wherein data are collected on the various inputs which are used for the cultivation of crop.

Food Economics and Commercial Crops Divisions

3.7 Food Economics Division examines the Kharif and Rabi Price Policy Reports of CACP. Commercial Crops Division examines the reports concerning Copra & Jute. The Government's price policy for major agricultural commodities seeks to ensure remunerative prices to the

growers for their produce with a view to encouraging higher investment and production and to safeguard the interest of consumers by making available supplies at reasonable prices. The price policy also seeks to evolve a balanced and integrated price structure in the perspective of the overall needs of the economy. Towards this end, the Government announces Minimum Support Prices (MSPs) in each season for major agricultural commodities and organizes purchase operations through public and cooperative agencies. The designated central nodal agencies intervene in the market for undertaking procurement operations with the objective that the market prices do not fall below the MSPs fixed by the Government.

3.8 The Government has fixed the MSPs for Kharif crops and Rabi crops of 2016-17 seasons **(Annexure 3.3).** The MSP of Paddy (common) for Kharif crops 2016-17 has been fixed at Rs. 1470 per quintal and of Paddy (Grade A) at Rs. 1510 per quintal, an increase of Rs. 60 per quintal each over the last year's MSPs. The MSP of Jowar (Hybrid) has been fixed at Rs. 1625 per quintal and Rs. 1650 per quintal for Jowar (Maldandi); Bajra at Rs. 1330 per quintal; Maize at Rs. 1365 per quintal and of Ragi at Rs. 1725 per quintal. The MSPs of Arhar (Tur), Moong and Urad have been fixed at Rs. 4625 per quintal, Rs. 4800 per quintal and Rs. 4575 per quintal respectively. To incentivise pulse production, a bonus of Rs. 425 per quintal may be payable over and above the proposed MSPs for Arhar (Tur), Moong and Urad.

3.9 The MSPs of Sesamum, Groundnut-in-shell, Sunflowerseed, Soyabean, and Nigerseed have been fixed at Rs. 4800 per quintal, Rs. 4120 per quintal, Rs. 3850 per quintal, Rs. 2675 per quintal and Rs. 3725 per quintal respectively. To incentivise Sesamum production, a bonus of Rs. 200 per quintal over and above MSP be given; in addition, to incentivise production of other oilseeds a bonus of Rs. 100 per quintal, payable over and above the MSP, be given for Groundnut-in-shell, Sunflowerseed, Soyabean, and Nigerseed. The MSP of Cotton has been raised by Rs. 60 per quintal and fixed at Rs. 3860 per quintal for Medium Staple length (mm) and at Rs. 4160 per quintal for Long Staple.

3.10 The MSP of Wheat for Rabi crops 2016-17 has been fixed at Rs. 1625 per quintal marking an increase of Rs. 100 per quintal over the last year's MSP. The MSP of Barley has been fixed at Rs. 1325 per quintal marking an increase of Rs. 100 per quintal over the last year's MSP. The MSP of Gram and Masur (Lentil) has been fixed at Rs. 3800 per quintal each marking an increase of Rs. 300 per quintal and Rs. 400 per quintal respectively over their last year's MSPs. In addition, bonus of Rs. 200 per quintal & Rs. 150 per quintal are payable over and above the MSP of Gram and Masur (Lentil). The MSPs of Rapeseed/Mustard and Safflower have been increased by Rs. 250 per quintal & Rs. 300 per quintal respectively over the last year's MSPs and has been fixed at Rs. 3600 per quintal each. In addition, bonus of Rs. 100 per quintal is payable over and above the MSP of Rapeseed/Mustard and Safflower. Details are given at **Annexure-3.3.**

3.11 The Government has fixed the Minimum Support Price (MSP) for Copra for 2016 season at Rs. 5950 per Quintal for Fair and Average Quality (FAQ) variety for Milling Copra and Rs. 6240 per Quintal for FAQ variety of Ball Copra. For 2016-17 seasons the MSP for Raw Jute has been fixed at Rs. 3200 per Quintal for FAQ variety. The MSP for Toria to be marketed in 2016-17 has been fixed at Rs. 3560 per Quintal.

Special Data Dissemination Standards (SDDS) Division

3.12 The Division generates quarterly estimates of agricultural production for use in the compilation of quarterly National Accounts by the Central Statistics Office. This activity was undertaken in order to meet the obligations concerning supply of data to the International Monetary Fund. In the absence of direct data, quarterly production is estimated by using the estimates of Kharif and Rabi seasons in conjunction with the crop calendar. The Division is also involved in collection and compilation of data relating to nine-fold classification of land, irrigated area (source-wise and crop-wise) and total area under crops for States and UTs. The compiled data is brought out in the Directorate's publications of 'Land Use Statistics', and 'Agricultural Statistics at a Glance'. The compiled data on Land Use Statistics (District wise and State- wise) from 1998-99 to 2013-14 are available on the website. The URL of the website is http://aps.dac.gov.in/LUS. Similarly, the district wise compiled data on Area, Production and Yield (APY) from 1998-99 to 2013-14 are also available on the website. The URL of the website is http://aps.dac.gov.in/APY.

Market Intelligence & Price Analysis Division

Wholesale Prices

3.13 The Division provides necessary market intelligence to the DAC&FW. Wholesale prices of 170 selected agricultural commodities are compiled on weekly basis from approximately 700 market centres spread all over the country. Of these, weekly wholesale prices of 80 agricultural commodities from 153 market/ centres spread all over the country, is provided to Office of the Economic Adviser, Department of Industrial Policy & Promotion (DIPP), Ministry of Commerce & Industry, for preparation of monthly Wholesale Prices Index (WPI) with base year 2004-05. For the revised series of WPI with base year 2011-12, this Division started providing to the Office of the Economic Adviser, DIPP, wholesale prices for a revised list of 100 commodities from about 200 markets making a total of 745 quotations.

3.14 Monthly wholesale prices of certain agricultural commodities and animal husbandry products at selected market / centres in India as well as international prices of selected agricultural commodities compiled in the Division are published in monthly journal, viz. Agricultural Situation in India.

3.15 An annual publication "Agricultural Prices in India" compiling statistics on wholesale, retail and international prices of selected agricultural commodities is also prepared.

Retail Prices

3.16 Retail prices of 45 food items every Friday from 87 market centres across the country are collected and compiled in the Division.

Farm Harvest Prices

3.17 Collection and compilation of data on farm harvest prices of 30 major crops from all States/Union Territories are obtained from their State Agricultural Departments/ Directorate of Economics & Statistics/ Directorate of Land Records, etc. A publication "Farm Harvest Prices of Principal Crops in India" is brought out.

International Prices

3.18 The Division maintains an international prices database comprising prices of 56 agricultural commodities across the world,

compiled from data obtained from State Trading Corporation (STC) and the UK based international journal - The Public Ledger.

Terms of Trade (ToT)

3.19 Index of Terms of trade (ToT) between agriculture and non- agricultural sectors is also prepared on annual basis as per the methodology recommended by the Working Group headed by Prof. S. Mahendra Dev, Director, Indira Gandhi Institute of Development Research (IGIDR), Mumbai. This index is used as an input for price policy formulation of agricultural crops by Commission for Agricultural Cost and Prices (CACP).

Agricultural Market Information System (AMIS)

3.20 In order to maintain timely, accurate and transparent information for addressing food price volatility and to track global food availability, AMIS- FAO (Food and Agricultural Organisation) prepares a Food Balance Sheet, where data on inputs of production, trade, food, feed and seed use as well as wastage of wheat, rice, maize and soyabean are maintained globally. DES, being assigned as the focal point, provides the data at regular intervals to AMIS (FAO) in the required format.

3.21 For strengthening the AMIS in India using innovative methods and digital technology, the FAO-funded AMIS Baby Project was initiated, jointly in collaboration with the Ministry of Agriculture and Farmers Welfare. The following activities were undertaken as parts of the Baby Project:

a) Field visits to selected mandis were undertaken by a team of DES - FAO officials to assess the existing modus operandi

b) A five day Computer Assisted Personal interviewing (CAPI) Training programme was organised for selected technical staff and officers from DES at IASRI, New Delhi as part of capacity building and technology transfer. But the feasibility of using CAPI in our data requirements need to be further examined

c) A Study Visit of Indian officials to the Philippines Statistical Authority (PSA) and National Food Authority (NFA) of Philippines for sharing experiences in Agricultural statistics as well to study methodology used by them was also funded by FAO.

d) An international seminar on "Approaches and Methodologies for Food Grain Stock Measurement" was organised during 9-11 November, 2016 to evolve a standardised methodology for measuring private food grain stock in India.

Agricultural Wages Division

3.22 Statistics relating to daily wages paid to different categories of agricultural and skilled rural labour at selected centres are collected and compiled by Directorate of Economics & Statistics. These are published in an annual publication "Agricultural Wages in India", and also in the monthly journal, viz. Agricultural Situation in India.

International Agriculture & Compilation Division

3.23 International Agriculture and Compilation Division(IAC) is concerned with preparing comments on international issues pertaining to agriculture sector dealt by various bilateral and multilateral organizations like G20, FAO, WTO, BRICS, etc. In addition, the Division carries out compilation and manuscript preparation works related to flagship publications of the Department namely, Agriculture Statistics at a Glance and State of Indian Agriculture.

Publication Division

3.24 Publication Division publishes the monthly Journal titled *Agricultural Situation in India* (ASI). The Journal is brought out by the Directorate of Economics and Statistics (DES), Ministry of Agriculture& Farmers Welfare. It aims at presenting a factual and integrated picture of the Food and Agricultural situation in India on monthly basis. The Journal intends to provide a forum for academic work and also to promote technical capability for research in agricultural and allied subjects. The Journal is being distributed every month among State agriculture Secretaries, libraries, Universities, AERC centers, and other Ministries. This Division mainly involves in the preparation of the manuscript of the Journal. In this regard, data on various agricultural units are collected and compiled from different Divisions of DES and other Ministries on monthly basis. The contents of farm sector news are collated and edited from the website of press information bureau, GoI. Articles received from agricultural economists, research scholars, Professors across various Indian Universities are considered for publication in ASI after rigorous editing job. One report from AERC centers which are being monitored by DES, is usually incorporated in each publication of ASI.

The following are the major contents of ASI:

- General Survey of Agriculture
- Farm sector news
- Articles
- Agro-Economic Research
- Commodity Reviews
- Statistical tables on wages, prices & crop production

This Division is also involved in publishing the following periodical of DES:

- Land Use Statistics at a Glance

Coordination

3.25 The Coordination Division's main work is to coordinate between Department of Agriculture, Cooperation & Farmers Welfare and Directorate of Economics & Statistics in matters relating to various issues. Its main activities include DAC&FW level Coordination for preparing Economic Survey Chapter on Agriculture & Food Management and for routine matters wherein several Divisions of DES and those of DAC&FW are involved, preparation of material for Annual Report, providing information on release and utilization of funds under plan schemes, Parliament Questions etc.

Plan Scheme

3.26 The DES implements the Plan Scheme "Integrated Scheme on Agriculture Census, Economics & Statistics". This scheme comprises six components of which one component is 'Agriculture Census' pertain to Agriculture Census Division of DAC&FW. The remaining five components are being implemented by the Directorate of Economics & Statistics. The details of the five components are as follows:

(i) Comprehensive Scheme for studying the Cost of Cultivation of principal crops in India

3.27 The Cost of Cultivation of principal crops in India is being implemented in India since 1970-71 as a Central Sector Plan Scheme. The main objectives of the scheme are to collect and compile data on cost of cultivation and production in respect of principal crops and to generate crop-wise and State-wise cost of cultivation and production estimates of mandated crops.

3.28 The Scheme is implemented through 16 Agricultural/Central Universities/ Colleges. Under the Scheme cost of cultivation/

production estimates in respect of principal crops are worked out and are transmit to the CACP so as to enable them to recommend the Minimum Support Price to the Government of India.

3.29 The cost estimates generated under the Schemes are also used for policy formulations by the Central Ministries and State Governments. Agricultural/ General Universities, Government/Non-government Research Organizations, individual researchers (both at domestic and international levels), etc. are also using the data for research purposes.

3.30 The Scheme is implemented in 20 States namely Assam, Andhra Pradesh, Bihar, Gujarat, Chattisgarh, Telangana, Haryana, Himachal Pradesh, Jharkhand, Karnataka, Kerala, Madhya Pradesh, Maharashtra, Orissa, Punjab, Rajasthan, Tamil Nadu, Uttar Pradesh, Uttrakhand and West Bengal. The studies in the States except newly created States of Chhatisgarh, Jharkhand, Uttarakhand and Telengana are undertaken by the parent States Agriculture universities/ colleges located in the respective States.

3.31 During the block period 2014-17, the Scheme covers 25 principal crops i.e. paddy, wheat, jowar, bajra, maize, ragi, barley, moong, urad, arhar, gram, lentil, groundnut, rapeseed and mustard, nigerseed, soyabean, sunflower, sesamum, cotton, jute, sugarcane, onion, potato, safflower and coconut.

3.32 The field data are collected on the basis of Cost Accounting Method by the 16 implementing agencies. Under the Scheme, daily entries of debit/credit for expenditure/ income are made in order to assess the total cost/benefit incurred/accrued to each sample farmer. The field data is collected by field-men who fill-up/updates the detailed questionnaire through direct interaction with the sample farmers of 10 selected farm holdings. These consist of 2 each from 5 different size classes viz. up to 1 hectare, 1-2 hectare, 2-4 hectare, 4-6 hectare and above 6 hectare allotted to each fieldman.

3.33 In the year 2015-16 (July to June), 156 cost estimates are generated and provided to CACP. Out of these estimates, 39 for Rabi, 98 for Kharif, 6 for sugarcane and 19 for others were generated in respect of 25 mandated crops.

Development of FARMAP 2.0 Software

3.34 The Directorate is in the process of development of web enabled FARMAP 2.0 software with the help of NIC. It will be utilized for online collection, compilation of cost data and generation of reports FARMAP 2.0 software will be helpful in collection and will also reduce the time lag in generation of cost estimates.

3.35 During the year 2016-17, against the allocated amount of Rs.50.15crore, Rs. 23.3 crore has been released till 31.10.2016.

(ii) Agro-Economic Research (AER) Scheme

3.36 The Agro-Economic Research (AER) Scheme initiated in 1954-55 through a network of 15 AER Centres/Units which conduct research and evaluation studies on yearly basis on Agricultural Economics and Rural Development for meeting the needs of the Department of Agriculture, Cooperation & Farmers Welfare and other Ministries/ Departments which have a bearing on the performance of the Agriculture Sector for policy formulation and as well as providing a feed back for implementation of the various schemes of the Government. The Centre also caters conducting to studies and offering economic analysis on the basis of the requirement put forward by the various State Governments in the feld of agriculture and rural development. The scheme is staff oriented and fully funded by

Ministry of Agriculture & Farmers' Welfare grant-in-aid under the "Integrated Scheme on Agriculture Census, Economics and Statistics" (component No. IV- AERC) of Central Sector Plan Scheme.

3.37 Presently there are 12 AERCs and 3 AERUs situtated in different states. The 3 AER Units namely ADRT, Banglore, CMA, Ahmedabad and AER Unit, Delhi are functioning under the Institute for Social and Economic Change, Banglore, Indian Institute of Management, Ahmedabad and Institute of Economic Growth, Delhi respectively. AERC Pune is functioning under the Gokhle Institute of Politics and Economics (a deemed university). The rest 11 Centres are functioning under different Universities, out of which AERCs at Jorhat, Ludhiana and Jabalpur are under Agriculture Universites following ICAR guidelines; AERCs at Allahabad, Delhi and Visva-Bharti, Shantiniketan are under Central Universities and the rest 5 Centres at Bhagalpur, Chennai, Shimla, Vallabh Vidyanagar and Waltair are functioning under the respective State Universities. Agro-Economic Research Centre, Jorhat was set up in 1960 and is functioning under the administrative control of Assam Agricultural University, Jorhat for conducting studies on various agricultural issues and problems of the North-Eastern States.

3.38 These AER centres/units have so far conducted about 2500 research/evaluation studies covering the core areas of concerning agriculture, such as fertilizer, seeds, credit, water resources as well as emerging new thurst areas, like horticulture, rainfed farming, agricultural subsidies, food economy, fishery, international cooperation, trade, environment and forest, agro rural industries, food processing industries etc. During 12th Five Year Plan, 137 studies have been conducted up to the year 2016-17 (till October, 2016). In the year 2016-17 till October, 2016-17 till December, 2016, 11 studies have been conducted on various fields. The most important of them are National Food Security Mission (NFSM); pre and post harvest losses of crops; and possibilities and constraints in adoption of alternative crops to paddy in green revolution belt of North India. Evaluation and Assesssment of Economic losses on account of inadequate Post Harvest infrastructure Facilities for Fisheries sector in India; and Estimation of losses to the pulses by blue-bulls in Uttar Pradesh.

3.39 The Department of Agriculture, Cooperation & Farmers Welfare, Ministry of Agriculture & Farmers Welfare has setup a Review Committee under the Chairmanship of Dr. S.M. Jharwal, Vice-Chancellor, Indira Gandhi National Tribal University to examine the various aspects of Agro-Economic Research Centres including the decision of discontinuation of grants-in-aid to the Agro-Economic Research Centres after the 12th Five Year Plan. The Committee has submitted its report to Ministry on 11th January, 2016. The Committee has recommended that grants-in-aid support to AERCs needs to be continued and the existing arrangement of AERCs should not be substituted by contract/bidding system. Directorate of Economics & Statistics is in process to submit a note to CCEA for implementation of the recommendation of the review Committee.

3.40 During 2015-16, Rs. 22.04 crore was allocated to the AERCs/Us and they have completed 27 research studies. For the Current Financial Year 2016-17 an allocation of Rs.2235.00 lakhs as Revised Estimate (RE) is provided (including Rs.175.00 lakhs for Northeast regions) out of which Rs.960.15 lakhs has been released (including Rs.69.09 lakhs for Northeast regions) by December, 2016. The AERCs have completed 11 research studies in 2016-17 (till December, 2016).

(iii) Planning, Management and Policy Formulation:

3.41 Presently these AER Centres/Units are conducting a fast track study on Neem coated Urea after 100% urea coating was allowed by the government. The interim report has already been submitted to the government and concerned Ministries/ Departments. The studies allotted to the AER centres are monitored according to the scheduled work plans and completed studies are then circulated to the concerned Ministries/Department so that they can be used in policy formulation.

3.42 In order to assess the impact of changes on the farm economy with focus on the state of Indian farmers, Planning and Management of Agriculture, a Central Sector Scheme, was formulated during 1998-99 with a view to organizing conferences and seminars involving eminent economists, agricultural scientist, experts, etc., to conduct short term studies, engage consultancy services for preparation of new decentralized strategy for development of crops, animals, dairy, poultry, irrigation, soil and water conservation etc. and to bring out papers/ reports based on the recommendations of the workshops, seminars, conferences, etc.

3.43 For the year 2016-17, an allocation of Rs.60.00 lakhs was made out of which an amount of Rs.7.15 lakhs has been released.

(iv) Improvement of Agricultural Statistics

3.44 The basic objective of the Central Sector Scheme, Improvement of Agricultural Statistics, is to collect and improve agricultural statistics of principal agricultural crops. The Scheme has three components; i) Timely Reporting Scheme (TRS), ii) Establishment of an Agency for Reporting of Agricultural Statistics (EARAS) and iii) Improvement of Crop Statistics (ICS). From 2007-08, the Scheme has been converted to Central Sector Scheme from Centrally Sponsored Scheme and is funded 100 percent by the Central Government. The Component-wise details of the scheme are as under:

Timely Reporting Scheme (TRS):

3.45 The objective of this component is to obtain estimates of area under principal crops in each season, with the breakup of area under irrigated/irrigated and traditional/high yielding varieties of crops on the basis of random sample of 20% of villages by a specific date. These estimates are used for generating advance estimates of production of principal crops. This component is being implemented in 17 land record States and also Union Territories of Delhi and Pondicherry.

Improvement of Crop Statistics (ICS):

3.46 The objective of this component is to improve the quality of statistics on area and production of crops through supervision and monitoring. Under this component, a sample check of area enumeration and crop cutting experiments of 10,000 villages and approximately 30,000 experiments at harvest stage are undertaken. These samples are equally shared by the Central Agency i.e. National Sample Survey Organization; and the State Agricultural Authorities. These checks specifically relate to (a) Enumeration of crop-wise area covered in the selected villages as recorded by the Patwari; (b) Total of the Area under each crop recorded in Khasra Register of villages; and (c) Supervision of Crop Cutting Experiments at the harvest stage. This component is being implemented in all TRS states and the Union Territory of Puducherry. The performance of the implementation of this component also is being closely monitored through Quarterly and Seasonal progress Reports.

Establishment of an Agency for Reporting of Agricultural Statistics (EARAS):

3.47 This component is being implemented in the permanently settled States of Kerala, Orissa and West Bengal and North Eastern States of Nagaland, Sikkim, Arunachal Pradesh and Tripura. Under the component, an agency has been established in these states for generating estimates of area and production of principal crops and land use statistics on the basis of complete enumeration of 20% villages in each year. The performance of the implementation of the component is being closely monitored thorough quarterly and seasonal progress report

3.48 For the financial year 2016-17, under Improvement of Agricultural Statistics Scheme against total allocation of Rs. 101.67 Crore, a sum of Rs. 78.10 Crore have been released till 16th November 2016.

Activities Undertaken for Welfare of Women and North- Eastern States

3.49 As the main objective of the scheme is to collect and compile agricultural statistics and it is not a welfare oriented scheme, no specific activity either for women or for the North-eastern States are undertaken under the Scheme. However, in North Eastern States (NES), the Scheme is in operation in Arunachal Pradesh, Assam, Nagaland, Sikkim & Tripura. For the financial year 2016-17 under the Scheme a sum of Rs. 6.03 crore has been released to NES by 16th November 2016.

Main activities of the year

Pilot study to Indian Agricultural Statistics Research Institute (IASRI):

3.50 In order to implement the recommendations of Professor Vaidyanathan Committee set up by the Government for improving agricultural statistics and to examine use of remote sensing applications in agricultural statistics, after the approval of CCEA, the Government has entrusted a pilot study to Indian Agricultural Statistics Research Institute, PUSA, New Delhi. The basic objective of the study will be to examine the reliability of estimates of crop area and crop production at State and national level on the basis of sample sizes recommended by the Vaidyanathan committee report. The Study will be conducted in five States namely Assam, Gujarat, Karnataka, Orissa and Uttar Pradesh.

(v) Forecasting Agricultural Output using Space Agro Meteorology and Land Based Observations (FASAL)

3.51 Department of Agriculture, Cooperation and Farmers' Welfare (DAC&FW) has been implementing a Central Sector Plan Scheme namely "Forecasting Agricultural output using Space, Agro-meteorology and Land based observations (FASAL)" since 2007 with partner organizations namely India Meteorological Department (IMD), New Delhi, Institute of Economic Growth (IEG), New Delhi and Space Application Centre (SAC), Ahmedabad to provide multiple-in-season forecast based on Agromet, Econometric and Remote Sensing based methodology. The mandate of FASAL Scheme is to generate crop forecast in respect of 11 crops, namely; Rice (Kharif & Rabi), Jowar (Kharif & Rabi), Maize, Bajra, Jute, Ragi, Cotton, Sugarcane and Groundnut (Kharif & Rabi), Rapeseed & Mustard and Wheat. Presently, the forecast in respect of 8 crops are being prepared.

Extended Range Forecast System (ERFS) Project

3.52 Department of Agriculture, Cooperation and Farmers Welfare is

implementing ERFS project in collaboration with 5 partner organizations viz. IIT, India Meteorological Department (IMD), National Centre for Medium Range Weather Forecasting (NCMRWF), Space Application Centre (SAC) Ahmedabad and Indian Council of Agriculture Research (ICAR). This project aims at forecasting the rainfall and temperature parametes on a seasonal/ monthly time scale. Another objective as the project is to develop and implement alternate strategies/plans for the farmers to manage their risk in the event of bad weather forecast. By disseminating these forecasts to the farming community and advice them about the various measures.

Financial Progress

3.53 During the current financial year 2016-17, out of Budget Estimates (BE) of Rs. 984.0 lakh and Revised Estimates (RE) of Rs. 796.00 lakh under the FASAL Scheme, the expenditure of Rs. 750.00 lakh has been incurred till 31st December, 2016.

******

Chapter 4

National Food Security Mission

4.1 National Food Security Mission was launched in 2007-08 to increase the production of rice, wheat and pulses by 10, 8 and 2 million tonnes, respectively by the end of 11th Plan through area expansion and productivity enhancement; restoring soil fertility and productivity; creating employment opportunities; and enhancing farm level economy. The Mission has continued during 12th Plan with new target of additional production of 25 million tonnes by the end of 12th Plan and promotion of commercial crops like cotton, jute & sugarcane.

4.2 The basic strategy of the Mission is to promote and extend improved technologies, i.e., seed, micro-nutrients, soil amendments, integrated pest management, farm machinery and implements, irrigation devices along with capacity building of farmers. The major interventions/activities covered under NFSM include cluster demonstrations of rice, wheat, pulses and coarse cereals, distribution of improved varieties/hybrid seeds, need based inputs, resource conservation techniques / energy management, efficient water/ application tools, cropping system based trainings and local initiatives; award for best performing districts etc.

4.3 During 12th Plan, in 2016-17, NFSM is implemented in 638 districts of 29 states. NFSM-Rice is implemented in 194 districts of 25 states viz. Andhra Pradesh, Arunachal Pradesh, Assam, Bihar, Chhattisgarh, Gujarat, Himachal Pradesh, Jammu & Kashmir, Jharkhand, Karnataka, Kerala, Madhya Pradesh, Maharashtra, Manipur, Meghalaya, Mizoram, Nagaland, Odisha, Sikkim, Tamil Nadu, Telangana, Tripura, Uttar Pradesh, Uttarakhand and West Bengal; NFSM-Wheat in 126 districts of 11 states viz., Bihar, Gujarat, Haryana, Himachal Pradesh, Jammu & Kashmir, Madhya Pradesh, Maharashtra, Punjab, Rajasthan, Uttar Pradesh and Uttarakhand; NFSM- Pulses in 638 districts of all 29 States viz. Andhra Pradesh, Arunachal Pradesh, Assam, Bihar, Chhattisgarh, Gujarat, Goa, Haryana, Himachal Pradesh, Jammu & Kashmir, Jharkhand, Karnataka, Kerala, Madhya Pradesh, Maharashtra, Manipur, Meghalaya, Mizoram, Nagaland, Odisha, Punjab, Rajasthan, Sikkim, Tamil Nadu, Telangana, Tripura, Uttar Pradesh, Uttarakhand and West Bengal ; NFSM-Coarse cereals in 265 districts of 28 States.

4.4 Monitoring & Evaluation at National level: A three-tier monitoring mechanism was inbuilt in NFSM at National, State and District levels. At National level General Council (GC) under the chairmanship of Union Agriculture Minister and Farmers Welfare was constituted to oversee the implementation of NFSM and take policy decisions for mid-term corrections. National Food Security Mission Executive Committee (NFSMEC) under the chairmanship of Secretary (AC & FW) periodically reviewed the State Action Plan and the progress of the programme of each state. National Level Monitoring Teams (NLMTs) reconstituted for monitoring the National Food Security Mission (NFSM) activities in respect of NFSM

States. Each team is comprised of experts in the field of rice, wheat, pulses and coarse cereals as per requirement, and the respective Directors of the Directorates of Crop Development of Ministry of Agriculture and Farmers Welfare as Coordinator. Altogether, there are eight (8) National Level Monitoring Teams (NLMTs).

4.5 At state level, the monitoring is undertaken by State Food Security Mission Executive Committee (SFSMEC) under the Chairmanship of Chief Secretary. At district level, the monitoring is undertaken by District Food Security Mission Executive Committee (DFSMEC) under the chairmanship of District Collector.

4.6 The Mid-Term Evaluation and the Impact Evaluation study of NFSM for 11th Plan was conducted by an independent agency to assess the impact of the programme in increasing production and productivity of rice, wheat, pulses and enhancement of farm income. The finding of evaluation reveals that NFSM has helped to widen the food basket of the country with significant contribution coming from the NFSM districts. Significant outputs were recorded under NFSM since inception of the programme in 2007-08. The Mission accomplished the target of additional production of 20 million tones within 4 years of its implementation.

4.7 Funding pattern: Till 2014-15 the programme was implemented on 100 % GOI assistance. However, from the year 2015-16, the programme is being implemented on 60:40 sharing basis between GOI and state and 90:10 sharing basis for North Eastern states and Hill states.

4.8 Interventions during 2015-16: Recommended agronomic practices have been encouraged through Cluster demonstrations in 15.98 lakh ha area. Nearly, 10.88 lakh quintals of high yielding varieties/hybrid rice, wheat, pulses and Coarse Cereals have been distributed. 20.14 lakh ha area has been treated with soil ameliorants (Micronutrients/Gypsum/Lime/ Bio-fertilizer) and 18.49 lakh ha area has been covered under Integrated Pest Management (IPM). Nearly 2.81 lakh improved farm machineries including pumpset & mobile raingun have been distributed. 0.19 lakh ha area has been covered under sprinkler sets. 84.47 lakh metres water carrying pipes have been distributed. About 9506 cropping system based trainings were conducted for capacity building of farmers.

4.9 A programme on Additional Area coverage of Pulses during Rabi/Summer 2016-17 has been launched with an allocation of Rs. 210.00 crores (GOI Share) to increase production of rabi/ summer pulses through area expansion of Rabi Pigeonpea, Gram, Pea and Lentil during Rabi and Green Gram and Black Gram during summer. The additional allocation for implementation of the above said programme has been made to 16 states covered under NFSM pulses.

4.10 Revamped NFSM from 2014-15: Cabinet Committee of Economic Affairs has cleared the continuation of NFSM programme in the XII Plan with approved outlay of Rs.12,350 crores with addition of coarse cereals and commercial crops. NFSM has been revamped during the XII Plan to cover all food grain crops including coarse cereals. During XII plan, the target of additional 25 million tonnes of food grains consisting of 10 million tonnes of rice, 8 million tonnes of wheat, 4 million tonnes of pulses and 3 million tonnes of coarse cereals has been fixed.

4.11 Government of India has approved implementation of crop development programme on cotton, jute and sugarcane for enhancing production & productivity of these crops under National Food Security

Mission (NFSM)-Commercial Crops (CC) w.e.f. 2014-15. Under this scheme thrust has been given on transfer on technology through frontline demonstrations and training in order to extend benefits to the farmers. Under NFSM-CC, an allocation of Rs. 4366.91 lakh has been made during 2016-17. This includes Rs.4100.11 lakh (Rs. 1235.50 lakh for Sugarcane, Rs. 1510.99 lakh for Cotton & Rs. 1335.62 lakh for Jute) for States with sharing pattern between Centre and States Governments on 60:40 basis for General category States & 90:10 basis for North East & Hilly States and Rs.266.80 lakh for Central Agencies implemented with 100% funding by Centre.

4.12 Outcome of NFSM since inception: The focused and target oriented implementation of mission initiatives has resulted in bumper production of wheat, rice and pulses in the country. During 2014-15, the productions of rice, wheat and pulses have been achieved at the level of 105.48 million tonnes, 86.53 million tonnes and 17.15 million tones, respectively As per 4th Advance Estimates during 2015-16, the production of wheat increased from 75.81 million tonnes in 2006-07 (pre-NFSM year) to 93.50 million tonnes, i.e. an increase of 17.69 million tonnes. Similarly, the total production of rice increased from 93.36 million tonnes in 2006-07 to 104.32 million tonnes in 2015-16 i.e. an increase of nearly 10.96 million tonnes. The total production of Pulses also increased from 14.20 million tonnes during 2006-07 to 16.47 million tonnes during 2015-16 with an increase of 2.27 million tonnes.

4.13 New Initiative for Pulses: From 2016-17, new initiatives like distribution of seed minikits, subsidy on production of quality seed, creation of seed hubs, strengthening breeder seed production programme and strengthening/establishing production units of bio-fertilizers and bio-control agents at ICAR institutes and State Agriculture Universities (SAUs), cluster frontline demonstrations through KVKs are being undertaken under NFSM to increasing productivity and production of pulses in the country.

4.14 Bringing Green Revolution to Eastern India (BGREI), a sub scheme of Rashtriya Krishi Vikas Yojana was initiated in 2010-11 to address the constraints limiting the productivity of "rice based cropping systems" in eastern India comprising seven (7) States namely; Assam, Bihar, Chhattisgarh, Jharkhand, Odisha, Eastern Uttar Pradesh and West Bengal. An amount of Rs. 400 crores each was allocated for the programme during 2010-11 & 2011-12 and an amount of Rs. 1000 crores each during 2012-13 to 2014-15. Till 2014-15 the programme was implemented on 100 % GOI assistance. However, from the year 2015-16, the programme is being implemented on 60:40 sharing basis between GOI and States and 90:10 sharing basis in NE State (Assam). An amount of Rs. 630.00 crore as central share has been earmarked for implementation of the programme during 2016-17.

4.15 Under this programme, interventions like; (i), Block demonstrations of rice and wheat in cluster mode; (ii) asset building activities such as construction of shallow tube, Dug well / Bore well, promotion of farm implements like seed drill, pumpset, cono-weeder, drum seeder and other need based farm implementions for cultivation of Rice/ wheat as per NFSM-Rice/ Wheat; (iii) need based site specific activities and (iv) Marketing support are also included.

4.16 During 2015-16, demonstrations on rice were conducted in 4.68 lakh ha area under line transplanting, SRI, Stress tolerant Varieties, Hybrid rice, Cropping System based demonstrations, Demonstrations on pulses and oilseeds in rice fallow areas. Similarly, 0.55 lakh ha was covered under

wheat demonstration. Under asset building component, 451 bore well were installed, 154 Drum Seeder, 39 Zero till Seed Drill, 1431 Seed Drill, 402 self Propelled Paddy Transplanter, 60855 Pump set, 4079 Conoweeder, 15574 Manual Sprayer, 4310 Power Knap Sack Sprayer, 240 Power Weeder, 2661 Paddy Thresher, 2042 Multi crop Thresher, 7144 rotavator, 65 tractor, 814 Power Tiller, and 930048 meter of water carrying pipe were distributed. For site-specific activities, 268 check dam, 15 Minor Irrigation Tank, 80 Community Surface lift irrigation projects, 111 water harvesting structures and 831 Dobha were constructed. For marketing support, 4 Nos of marketing shed (100 % assistance), 125 community threshing floor, 10 pre-fabricated storage godown, 2762 Nos of improved low cost paddy and grain storage were constructed and 2758 Farm family level paddy processing unit and 2758 Manually operated grain drier with paddy husk were distributed.

4.17 Crop Diversification Programme: Crop Diversification Programme is being implemented in Original Green Revolution States viz: Punjab, Haryana and Western Uttar Pradesh as a sub scheme of RKVY since 2013-14 to divert the area of water guzzling paddy to alternate crops like pulses, oilseeds, maize, cotton and agro forestry with the objective of tackling the problem of declining of soil fertility and depleting water table in these states. In order to encourage tobacco growing farmers to shift to alternate crops/ cropping systems, Crop Diversification Programme has been extended to tobacco growing states of Andhra Pradesh, Bihar, Gujarat, Karnataka, Maharashtra, Odisha, Tamil Nadu, Telangana, Uttar Pradesh and West Bengal to encourage tobacco growing farmers to shift to alternate crops/ cropping system w.e.f. 2015-16. An amount of Rs. 500.00 crore & Rs. 250.00 crore were allocated for Crop Diversification Programme in Original Green Revolution States (Punjab, Haryana and Western Uttar Pradesh) during year 2013-14 & 2014-15 on 100 % GOI assistance. However, from the year 2015-16, the programme is being implemented on 60:40 sharing basis between GOI and State Governments. During 2015-16, financial allocation of Rs. 150.00 crore as a central share (Rs. 125.00 crore for Crop Diversification Programme in Original Green Revolution States and Rs. 25.00 crore for replacing tobacco farming with alternate crops/cropping system) have been allocated. During 2016-17, an amount of Rs. 180.00 crore as a central share (Rs. 150.00 crore for Crop Diversification Programme in Original Green Revolution States and Rs. 30.00 crore for replacing tobacco farming with alternate crops/cropping system) have been earmarked.

4.18 Interventions during 2015-16: Cluster demonstrations of alternate crops, farm mechanization & value addition, site-specific activities and awareness campaigns/training programmes are the major interventions of the programme. The scheme has encouraged farmers to adopt alternate crops in place of paddy in Original Green Revolution States (Punjab, Haryana and Western Uttar Pradesh) and encourage tobacco growing farmers to shift to alternate crops/cropping system in tobacco growing states. Under Crop Diversification Programme in Original Green Revolution States, cluster demonstration on alternate crops like pulses, oilseeds, maize, cotton, agro forestry and inter cropping were conducted in 83,366 hectare area. In Punjab, major achievements was on distribution of farm machinery/equipments like 3000 rotavator, 250 laser land leveler, 200 Seed drill/Zero till drill/ Spatial zero till drill/ DSR drill cum planter, 5400 power operated spray pumps, 4500 battery powered sprayers, 100 tractor operated hydraulic sprayers and 50 raised bed planter/ridge planter. In Haryana,

612 tractor operated sprayer, 1569 zero till seed cum fertilizer drill, 724 laser land leveler, 469 rotavator and 3 happy seeder were distributed under farm mechanization & value addition. Under site-specific activities, 15237 ha underground pipeline were given to the farmers. Under awareness activities 1 State level, 8 district level and 47 block level Kisan Mela were organized during 2015-16. In Western UP, 1202 battery/power operated sprayer, 306 manual sprayer/knap sack sprayer, 91 tractor mounted sprayer, 1682 multi crop thresher, 4730 rotavator, 138 seed drill, 20 zero till seed drill, 77 power weeder, 803 chaff cutter (manual), 6933 power chaff cutter, 20 reaper cum binder self propelled machine, 149 tractor drawn reaper, 5 reaper self propelled machine, 3 straw chopper and 32 power tiller distributed under farm mechanization & value addition. Under site-specific activities, 4514 pump set and 79 sets of 600m water carrying pipes, 130 ha of underground water carrying pipe line, 1 surface solar water pump, 26 submersible solar water pump, 1064 laser land leveler, 926 cultivator and 1041 harrow were distributed to the farmers. Under awareness activities, 269 village/block level training, 115 district/block/tehsil level Krishak Gosthi, 23 outside the state exposure visit and 12 within state exposure visit for farmers were organized during 2015-16.

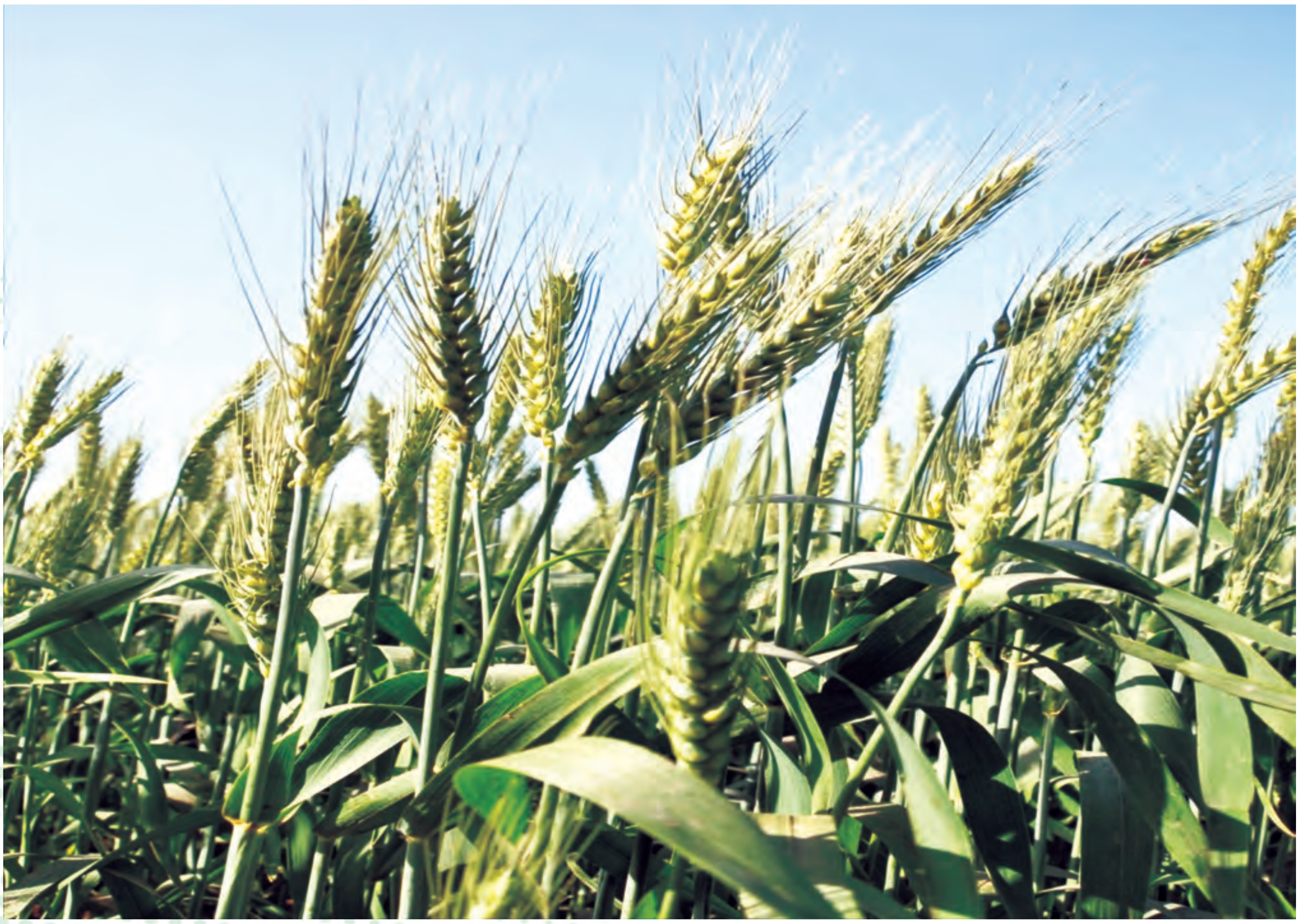

******

Chapter 5

Mission for Integrated Development of Horticulture (MIDH)

Progress of Horticulture during XII Plan

5.1 During XII Plan, with effect from 2014-15 Department of Agriculture, Cooperation & Farmers Welfare has launched Mission for Integrated Development of Horticulture (MIDH) by subsuming schemes on horticulture viz. (i) National Horticulture Mission (NHM), (ii) Horticulture Mission for North East and Himalayan States (HMNEH), (iii) National Bamboo Mission (NBM), (iv) National Horticulture Board (NHB), (v) Coconut Development Board (CDB), (vi) Central Institute of Horticulture (CIH), Nagaland.

5.2 The budget allocation of Rs. 1620.00 crore has been earmarked for MIDH during 2016-17. As on 31st December, 2016, funds to the tune of Rs. 1047.29 crore have been released for implementation of activities of MIDH i.e. Rs. 605.61 crore under NHM, Rs. 181.29 crore under HMNEH, Rs. 149.87 crore under NHB, Rs. 97.50 crore under CDB, Rs. 9.76 crore to NABM and Rs.3.26 crore to CIH.

National Horticulture Mission (NHM):

5.3 This Centrally Sponsored Scheme was launched in the year 2005-06 aims at the holistic development of horticulture sector by ensuring forward and backward linkage through a cluster approach with the active participation of all stake holders. 384 districts in 19 States and 4 Union Territories were covered under NHM. Eighteen (18) National Level Agencies (NLAs) have also been included for providing support for developmental efforts which require inputs at the National level.

5.4 Supply of quality planting material through establishment of nurseries and tissue culture units production and productivity improvement programmes through area expansion and rejuvenation, technology promotion, technology dissemination, human resource development, creation of infrastructure for post harvest management and marketing in consonance with the comparative advantages of each State/region and their diverse agro-climatic conditions were major programmes implemented during 2014-15. The summary details of progress achieved so far during 2016-17 are given table below:

Table 1: Progress under NHM

(Area in lakh ha)

S. No.	Components	Unit	Progress during 2016-17 (as of 31 Dec. 2016)
1	Nursery	No.	32
2	Area Expansion	Ha.	78123
3	Rejuvenation of old & senile orchards	Ha.	5501
4	Organic Farming	Ha.	4450
5	Integrated Pest/Nutrient Management	Ha.	24862
6	Pack house	No.	1376
7	Cold Storage	No.	70
8	Primary/mobile processing units	No.	108
9	Market	No.	7
i	Wholesale	No.	-
ii	Rural	No.	7

Financial progress:

5.5 During 2016-17, as on 31st December, 2016, funds to the tune of Rs. 605.61 crore have been released to States/Union Territories and National Level Agencies implementing NHM scheme against BE of Rs.890.00 crore.

Horticulture Mission for North East and Himalayan States (HMNEH)

5.6 Ministry of Agriculture & Farmers Welfare, Government of India has been implementing a Centrally Sponsored Scheme - Horticulture Mission for North East and Himalayan States (HMNEH) earlier known as "Technology Mission for Integrated Development of Horticulture in North Eastern States since 2001-02. During the X Plan (2003-04), the scheme was further extended to three Himalayan States namely: Himachal Pradesh, Jammu and Kashmir and Uttrakhand. The Mission covers entire spectrum of horticulture, right from planting to consumption, with backward and forward linkages. With effect from 2014-15, HMNEH scheme has been subsumed under the Mission for Integrated Development of Horticulture (MIDH).

Physical progress

5.7 The summary details of progress achieved so far under MIDH during 2016-17 are given table below:

Financial Progress

5.8 During the current financial year, funds to the tune of Rs. 181.29 crore have been released to States implementing HMNEH scheme against BE of Rs.320.00 crore.

National Agro-forestry & Bamboo Mission (NABM)

5.9 Funds amounting to Rs. 9.76 crore have been released to NABM implemented states only for maintenance of already created infrastructure under the sub-scheme.

Coconut Development Board (CDB)

5.10 During the current financial year, funds to the tune of Rs. 97.50 crore have been released to CDB for coconut development in the country against BE of Rs. 130.00 crore.

National Horticulture Board (NHB)

5.11 Funds amounting to Rs. 149.87 crore have been released to NHB against BE of Rs. 275.00 crore.

Table 2: Progress under HMNEH

(Area in lakh ha)

S. No.	Components	Unit	Progress during 2016-17 (as of Dec. 2016)
1	Nursery	No.	16
2	Area Expansion	Ha.	8238
3	Rejuvenation of old & senile orchards	Ha.	1363
4	Organic Farming	Ha.	-
5	Integrated Pest/Nutrient Management	Ha.	11143
6	Pack house	No.	307
7	Cold Storage	No.	-
8	Primary/mobile processing units	No.	-
9	Market	No.	5
i	Wholesale	No.	-
ii	Rural	No.	5

Central Institute of Horticulture (CIH), Nagaland

5.12 During the current financial year, an amount of Rs.3.26 crore have been released to CIH, Nagaland against BE of Rs. 5.00 crore.

Area, Production & Productivity of Horticulture Crops

5.13 The comparative details of area, production and productivity of various horticulture crops during 2015-16 with reference to 2004-05 viz. pre and post NHM scenario are given in the following Table 3.

Table 3: Pre and Post NHM Scenario: Area, Production and Productivity

(Area in 000'ha, Production in 000'Tonne, Productivity: Tonne/ha)

Crop	Area				Production				Productivity		
	2004-05	2014-15	2015-16 (3rd adv. esti-mates)	Annual Growth Rate% (2015-16 over 2014-15)	2004-05	2014-15	2015-16 (3rd adv. esti-mates)	Annual Growth Rate% (2015-16 over 2014-15)	2004-05	2014-15	2015-16 (3rd adv. esti-mates)
Fruits	5049	6110	6405	4.83	50867	86602	91443	5.59	10.07	14.2	14.3
Vegetables	6744	9542	9575	0.35	101246	169478	166608	(-)1.69	14.99	17.8	17.4
Flowers											
Loose	118	249	243	(-)2.53	659	1659	1545	(-)6.86	5.65	8.6	6.4
Spices	3150	3317	3264	-1.61	4001	6108	6350	3.97	1.36	1.8	1.9
Medicinal &Aromatic Plants	131	659	617	(-)6.33	159	1000	1156	15.64	1.18	1.5	1.9
Plantations	3147	3534	3683	4.23	9835	15575	15477	(-)0.63	4.22	4.4	4.2
Others	106				172		782				
**Total**	**18445**	**23410**	**23787**	**1.61**	**166939**	**280986**	**283360**	**0.84**	**9.1**	**12**	**11.9**

5.14 The wide and varied nature of horticulture sector covering fruits, vegetables, root and tuber crops, flowers, aromatic and medicinal crops, spices and plantation crops facilitates better returns per unit of area besides opportunities for diversification in agriculture.

5.15 Horticulture crops cover an area of 23.8 million ha (m. ha) at present by registering increase of about 17.8% as compared to 20.2 m ha in 2007-08. However, with a production of about 283.4 million MT, horticulture production has witnessed an increase of about 34.3% during the period 2007-08 to 2015-16. The significant feature is that there has been improvement of productivity of horticulture crops, which increased by about 13.8% during this period.

5.16 Area, production and productivity of horticulture crops during past 9 years are given in **Table 4**.

Table 4:-Area, Production and Productivity of Horticulture Crops

(Area : m.Ha, Prod.: m. MT, Pvty. MT/ha)

Year	Area	Production	Productivity
2007-08	20.2	211.0	10.4
2008-09	20.5	214.4	10.5
2009-10	20.8	223.2	10.7
2010-11	21.8	240.4	11.0
2011-12	23.2	257.3	11.1
2012-13	23.7	268.8	11. 3
2013-14	24.2	277.4	11.5
2014-15	23.4	280.99	12.01
2015-16* (3rd adv. Estimates)	23.7	283.36	11.91

5.17 The area under fruit crops during 2015-16 was 6.4 m. ha with a total production of 91.4 m. MT. During the period, production of fruits increased by about 39% while the area increased by about 7%. Comparative details of area, production and productivity of fruit crops are given are in **Figure 1**.

Figure 1:-Area, Production and Productivity of Fruits

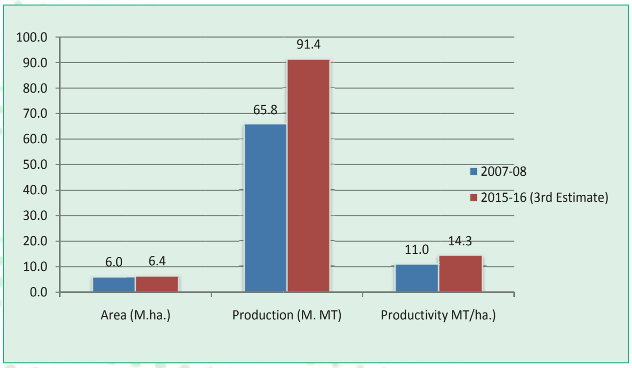

5.18 India has retained its status as the second largest producer of fruits in the world. The country is first in the production of fruits like mango, banana, sapota, pomegranate and aonla.

5.19 Vegetables are an important crop in horticulture sector, occupying an area of 9.6 million ha during 2015-16 with a total production of 166.6 million tonnes with average productivity of 17.4 tonnes/ha. In fact vegetables constitute about 59% of horticulture production. During the period (2007-08 to 2015-16), area and production of vegetables increased by 22% and 29% respectively, Comparative details are depicted in the **Figure 2**

Figure 2:-Area, Production and Productivity of Vegetables

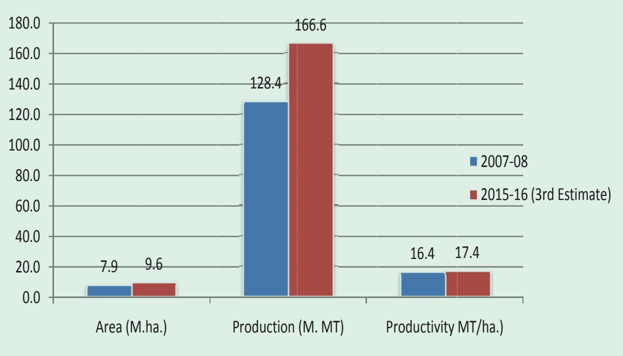

5.20 India continued to be second largest producer of vegetables after China. India is a leader in production of vegetables like peas and okra. Besides, India occupies the second position in production of brinjal, cabbage, cauliflower and onion and third in potato and tomato in the world. Vegetables such as potato, tomato, okra and cucurbits are produced abundantly in the country.

5.21 Interventions in horticulture in the country, have led to increase per capita availability of fruits from 133 gm/person/day in 2004-05 to 195 gms/person/day in 2015-16. Similarly, per capita availability of vegetables has increased form 264 gm/person/day in 2004-05 to 355 gm/person/day in 2015-16.

5.22 India has also made noticeable advancements in production of flowers, particularly cut flowers, which have a high potential for exports. Floriculture during 2015-16 covered an area of 0.24 million ha with a production of 1.55 m. MT of loose flowers and 0.69 m. MT of cut flowers. There

has been phenomenal growth in cut flower production, which is finding place in export market.

5.23 India is the largest producer, consumer and exporter of spices and spice products, the total production of spices during 2015-16 was 6.4 m. MT from an area of 3.26 m. MT.

National Agro forestry & Bamboo Mission (NABM)

5.24 With the launch of Sub-mission on Agro Forestry (SMAF), funds towards maintenance are only provided for infrastructure already created under NABM.

National Horticulure Board

5.25 The National Horticulture Board (NHB) was established in the year 1984 by the Government of India as an autonomous organization and registered as a society with the Registrar of Firms and Societies Chandigarh with its headquarters in Gurgaon and 35 field offices located all over the country. The broad aims and objectives of the Board are the creation of production hubs for commercial horticulture development, post harvest infrastructure and cold chain facilities, promotion of new crops, and promotion of growers' associations.

5.26 The Board is implementing the following schemes:

(1) Development of Commercial Horticulture through Production and Post-Harvest Management: Under this scheme, credit linked back-ended subsidy (i) @ 40% of the total project cost limited to Rs.30.00 lakh per project in general areas and @50% of project cost limited to Rs. 37.50 lakh in NE Region, Hilly and Scheduled areas, for commercial horticulture development in open field conditions on project mode (ii) @ 50% of the total project cost limited to Rs. 56.00 lakh per project as per admissible cost norms for commercial horticulture development in protected cover on project mode for green houses, shade net house, plastic tunnel, anti bird/hail nets & cost of planting material, credit linked back-ended subsidy and (iii) @ 35% of the cost limited to Rs. 50.75 lakhs per project in general area and @ 50% of project cost limited to Rs. 72.50 lakhs per project in NE, Hilly and Scheduled Areas is provided for the projects relating to production, Post Harvest Management (pack house, ripening chambers, refer van, retail outlets, pre-cooling units etc.) marketing and primary processing of horticulture produce.

(2) Capital Investment Subsidy for Construction/ Expansion/ Modernization of Cold Storages and Storages for Horticulture Produce: Under this scheme, credit linked back-ended subsidy @ 35% of the project cost (50% in NE, Hilly and Scheduled Areas) for capacity above 5000 MT and upto 10000 MT is provided for Construction/Expansion/ Modernization of Cold Storages including Controlled Atmosphere (CA).

(3) Technology Development and Transfer for Horticulture Produce: Under this scheme, the Board is also providing grant for popularization of identified new technologies/tools/techniques for commercialization and adoption through following programmes:

- Setting up of block / mother plant and root stock nursery (Area above 4 ha).
- Acquisition of technologies including import of planting material.
- Import of new machines and Tools for horticulture.
- Development & Transfer of Technology.
- Long Distance Transport Solution
- Product Promotion and Market Development Services- Horti-fairs

- Exposure visit of farmers (Outside State)
- Visit Abroad for Government Officers
- Organization/Participation in Seminar/ symposia/workshop for development of horticulture
- Accreditation and Rating of Fruit Plant Nurseries

(4) Market Information Service for Horticulture Crops: NHB is implementing this scheme by establishing a network connecting 35 major wholesale fruit and vegetable markets in the country. The information generated under this scheme is published in the form of technical reports and bulletins and also made available online on NHB website.

(5) Horticulture Promotion Services/ Expert Services and Strengthening Capability of NHB: Under this scheme, specialized studies and surveys are carried out and study/survey reports are brought out for use by targeted beneficiaries. In addition, technical laboratories are set up or cause to be set up and also provide technical services including advisory and consultancy services.

Coconut Development Board

5.27 Coconut Development Board Kochi, (CDB) is a statutory body established by Govt. of India in 1981. Coconut is cultivated in 16 states and 4 UTs in the country and provides food and livelihood security to more than 12 million people. There are 5 million coconut holdings in the country and the average size of these holdings is less than one hectare. India is the largest coconut producing country in the world contributing 31% of the world production. As per the all India estimates for the year 2014-15, the area and production of coconut in the country is 1.98 million hectares and 20439.61 million nuts respectively. The four southern states of Kerala, Karnataka, Tamil Nadu and Andhra Pradesh account for 88 percent of the coconut area and 90 percent of the coconut production in the country. The productivity of coconut at national level for 2014-15 is 10345 nuts per hectare.

5.28 The thrust areas identified by the Coconut Development Board programmes under MIDH are: production and distribution of quality planting material, expansion of area under coconut cultivation especially in potential and non-traditional areas, improving the productivity of coconut in major coconut producing states, developing technology in post-harvest processing and marketing activities, product diversification and by-product utilization of coconut for value addition.

5.29 The major programs that are being implemented by the Board are;

A1. Production and Distribution of Quality Planting Materials

- **(a)** Establishment of Demonstration-cum-Seed Production Farms
- **(b)** Establishment of Regional Coconut Nurseries
- **(c)** Distribution of Hybrids/Dwarf Seedlings in Govt/Pvt sector
- **(d)** Establishment of Nucleus Coconut Seed Garden
- **(e)** Establishment of Small Coconut Nurseries

A2. Expansion of Area under Coconut

A3. Integrated Farming for productivity improvement

- **(a)** Laying out of Demonstration Plots
- **(b)** Aid to Organic Manure Units

A4. Technology Demonstration/Quality Testing Lab

A5. Marketing, market Intelligent services, Statistics and Strengthening of Export Promotion Council

A6. Information & Information Technology

A7. Technical Service & Project Management

A8. Infrastructure and Administration

B. Technology Mission on Coconut

C. Rejuvenation and Replanting

D. Coconut Palm Insurance Scheme

E. Kera Suraksha Insurance Scheme

F. Field Research

New Activities

a. Centre of Excellence (CoE) for Coconut

b. Establishment of New Coconut Orchards

A1. Production and distribution of quality planting material

a. Establishment of Demonstration cum Seed Production (DSP) Farms in different parts of the country for creating infrastructure facilities for production of quality planting materials besides demonstrating and educating the scientific coconut cultivation and processing to various stake holders in those regions. The Board has established 10 DSP Farms in the States of Kerala, Karnataka, Tamilnadu, Andhra Pradesh, Maharashtra, Bihar, Assam, Chatisgarh, Odisha and Tripura. It is targeted to produce 10 lakh coconut seedlings during 2016-17 from the DSP Farms

b. Establishment of Regional Coconut Nurseries: Extending support to the participating States for strengthening the seedling production programme of the concerned States by establishing Regional Coconut Nurseries by way of extending financial assistance on 50:50 basis. As per the revised guidelines of MIDH, the pattern of assistance is 50% of the cost of Rs.32/- per seedling. The current year allocation is production of 3.12 lakh coconut seedlings utilizing Rs.50 lakh.

c. Distribution of hybrids/dwarf seedlings in Govt./Private sector: The scheme for the production and distribution of quality planting material of hybrids and local recommended cultivars is implemented through various State Agri./Hort. Depts. on 50:50 sharing basis and realizing back the 50 percent share of receipts. As per the revised guidelines of MIDH, the pattern of assistance is 25% of the cost of Rs.36/- per seedling for a maximum of 25000 seedlings per acre. The current year allocation is production of 2 lakh hybrid coconut seedlings utilizing Rs.18 lakh.

d. Establishment of Nucleus Coconut Seed Garden: The scheme is for developing infrastructure for continuous production of quality coconut seedlings in different States. Assistance is provided for establishing the seed gardens @ 25% of the total expenditure incurred limited to a maximum of Rs.6 lakhs over a period of 3 years. The current year allocation is Rs.34 lakh for establishing Nucleus Seed Gardens in various states on need basis.

e. Establishment of Small Coconut Nurseries: The scheme is for developing infrastructure for continuous production of quality coconut seedlings in different States. Financial assistance is at the rate of Rs.2.00 lakhs or 25% of the cost of production of seedlings whichever is less and is disbursed in two equal annual installments. As per the revised guidelines of MIDH, the assistance is 100% of the cost. Minimum eligible subsidy is Rs.50,000/- for a unit to produce at least 6250 certified coconut seedlings annually and the maximum limit of subsidy of Rs.2.00

lakhs is to produce at least 25,000 certified quality seedlings per annum. The current year allocation is Rs.30 lakh for establishing Small Coconut Nurseries in various states on need basis.

5.30 During the period under report, a total of 15.12 lakh quality coconut seedlings will be produced and distributed to farmers for new planting, replanting and under planting out of which 10 lakh seedlings will be produced in the Nurseries attached to DSP Farms of the Board, 3.12 lakh seedlings in the Regional Coconut Nurseries established by the Department of Agriculture/Horticulture of the participating states and 2 lakh hybrid seedlings will also be produced under the scheme 'Distribution of hybrids/dwarf seedlings in Govt./.Pvt sector.

A2. Expansion of Area under Coconut

5.31 The objective of the programme is to extend adequate technical and financial support to the farmers to take up coconut cultivation on scientific lines in potential areas to attain a significant achievement in the future production potential. For establishment of new plantations subsidy of Rs.8,000/- per hectare in two years as two equal installments was being provided. As per the revised guidelines of MIDH, the assistance is revised as follows: Normal area: 25% of the cost of Rs.26,000/-,Rs.27,000/- and Rs.30, 000/- per ha for Tall, Hybrid and Dwarf respectively. Hilly and scheduled areas: 25% of the cost of Rs.55,000/-,Rs.55,000/- and Rs.60,000/- per ha for Tall, Hybrid and Dwarf respectively.

5.32 During the current year it is targeted for 1862 ha area coverage under new planting of coconut utilizing Rs. 200 lakh.

A3. Integrated Farming for Productivity Improvement

5.33 The objective of the programme is to improve production and productivity of the coconut holdings through an integrated approach and thereby increasing the net income from unit holdings with the following component programmes.

(a) Laying out of Demonstration Plots:

5.34 Financial assistance of Rs.35,000/- per hectare in two annual installments is provided for adoption of integrated management practices in disease affected gardens. The objective of the programme is to encourage the farmers to adopt the technology developed for the management of disease affected coconut gardens. The Demonstration Plots will also help motivate farmers in adoption of result oriented production technologies. During the year 2016-17, it is proposed to implement the scheme in all coconut growing states through various State Governments and directly by the Board through Farmer Producer Organizations (FPOs) covering an area of 5808 ha and maintenance of 11334.16 ha of the previous year with a total financial outlay of Rs.3000 lakhs.

5.35 Under the scheme "Laying out of Demonstration Plots", an amount of Rs.2052.80 lakhs was incurred as on 11.11.2016 for covering 8765 ha fresh area and 10811.71ha maintenance area. Under direct implementation of the scheme, the efficacy of scientific management of coconut palms and coconut based farming systems was demonstrated in an area of 7635 ha so far on a cluster basis with farmer participation incurring Rs.1774.84 lakhs. The scheme "Laying out of Demonstration Plots" was also implemented through various State Agriculture/Horticulture Departments and Rs.277.95 lakh has been released to the State Governments for the implementation covering 1130 ha.

5.36 During the year 2016-17, apart from the regular budget for LODP, an amount

of Rs.20 crores is allocated to Board for implementation of this scheme under SCSP/TSP funds. Board has allocated the fund to 18 coconut growing states/UTs. So far Rs.321.91 lakh has been incurred for covering 2445 ha in 7 States/UTs through state departments as well as direct implementation.

(b) Assistance for Organic Manure Units:

5.37 The objective of the scheme is to promote the use of organic manure like vermicompost, coir pith compost etc in coconut holdings. As per the revised guidelines of MIDH, the assistance is revised as 100% of the cost of Rs.60,000/- per unit. An amount of Rs.40 lakhs is earmarked for 2016-17 for establishing 100 organic manure units in coconut gardens for promoting the use of organic manures in various coconut growing states.Under the scheme establishment of Organic Manure Unit, an amount of Rs.7.69 lakh has been utilized for setting up of 21 units in various states.

A4. Technology Demonstration

5.38 The Technology Development Centre at Vazhakulam, Aluva, Kerala is engaged in the development and demonstration of technologies for product diversification and by-product utilisation of coconut. Training is being given to interested entrepreneurs and self help groups for acquiring technologies on post harvest coconut processing and process demonstration. A full fledged Quality Testing Laboratory (QTL) is operating at the Technology Development Centre, Vazhakulam for chemical and microbial analysis of coconut based products. The laboratory is equipped with advanced analytical instruments and modern facilities as per NABL requirements to carry out chemical/microbiological tests of coconut based products.

A5. Marketing, market Intelligent services, Statistics and Strengthening of Export Promotion Council

a) Market information and intelligence service

b) Modernisation of coconut processing by introduction of improved copra dryer through FPOs(CPS/CPF/CPC)

c) Surveys and Evaluation Studies

d) Export Promotion Council

374 exporters have registered with the Board during the period 1st April to11th November 2016.

Farmer Producers organization (FPOs)

5.39 The Board has initiated the formation of Coconut Producer Societies (CPS) by associating 40-100 coconut growers in a contiguous area with a consolidated minimum of 4000-6000 palms. The objective is socio economic upliftment of the farmers through productivity improvement, cost reduction, efficient collective marketing, processing and product diversification. The Board is facilitating formation of Coconut Producers Federation (CPF) by aggregation of 15-25 CPSs as its members and having 1, 00,000 bearing palms, and further to Coconut Producer Company (CPC) having 10 CPFs and 10, 00,000 bearing palms.

5.40 As on 11.11.16 a total of 9293 CPSs, 721 CPFs and 64 CPCs have been registered with Board in different states.

A6. Information and Information Technology

5.41 The Board has several programmes under information technology and HRD with a view to disseminate information on various aspects of coconut cultivation and

industry through various media, publication etc besides organizing training programmes to impart skills and knowledge to farmers, unemployed youths and rural women in various fields related to coconut. For recognizing out standing performance in coconut sector a scheme of National Award is also being implemented biannually. Production of publications, undertaking multimedia product promotional campaigns, participation in exhibitions and fairs, management of MIS in the organization etc; are the major activities undertaken under the scheme.

5.42 To create awareness about the activities of the Board and health aspects of coconuts and coconut products, Board participates in exhibitions, seminars etc.

5.43 The major programmes attended by Coconut Development Board during the current financial year are Horticulture Mango Festival at Vijayawada, Andhra Pradesh, 7th Krishi Fair (a National level exhibition) at Puri,'Inter-State Horti. Fair Sangam' at Navsari Agricultural University, Navsari, Gujarat, 'Agri Summit Exhibition' at PGP of Agriculture Science, Palani Nagar, Namakkal, Tamilnadu, 'Infra Educa' at Pragati Maidan, New Delhi, 'Inter-State Horti Sangam' at Hajipur, Bihar, India International Coir Fair organized by the Coir Board at Coimbatore, Tamilnadu, 'Agri Intex' at Coimbatore, Tamilnadu, 5th Science Expo at Solan, Himachal Pradesh, 23rd Zonal Work Shop of Krishi Vigyan Kendra at Bhubanewsar.

Friends of Coconut tree

5.44 With a serious view to tackle the problem of acute shortage of trained coconut palm climbers for harvesting and plant protection activities, the Board is conducting a massive program, "Friends of Coconut Tree" to train underemployed youth in developing special skills and confidence in coconut palm climbing and plant protection activities for the benefit of the coconut community as a whole. As on 11.11.16, 53406 nos. have been trained under this programme.

A7&A8. Infrastructure & Administration including Technical Services & Project Management

5.45 During the year 2016-17 an amount of Rs.1950 lakh is allocated under Infrastructure & Administration. Besides, Rs.430 lakh is allocated for Technical Services & Project Management.

B. Technology Mission on Coconut

5.46 Technology Mission on coconut was sanctioned during the financial year 2001-02 with the objectives of (a) development of technology for new value added coconut products and by-products by research, bring these value added products to commercial production by assisting to promising entrepreneurs (b) providing assistance for the controlling of specific disease/pest in any specific area including development of technology for controlling of specific disease/pest to ensure undisrupted supply of raw materials to the coconut industry for the production of value added products and by products (c) develop and promote market for such newly developed values added products and by-products including traditional products (ball copra, copra and oil) by research, surveys and brand promotion. The implementation of this scheme is on time bound project basis.

5.47 An amount of Rs.1200 lakhs is allotted for implementation of the Technology Mission on Coconut during 2016-17. Against this, an amount of Rs.5.23 crores has been released for 25 new projects and other ongoing projects to entrepreneurs, various Research Institutions, State Governments, Co-operative Societies, etc. for development,

demonstration and adoption of technology for product diversification and by product utilization, management of pests and diseases and market research and market promotion.

C. Replanting and Rejuvenation of Coconut Gardens.

5.48 The main objective of the scheme is to enhance the productivity and production of coconut by removal of disease advanced, unproductive, old and senile palms and rejuvenating the remaining palms.

5.49 The component wise subsidy has been revised from 2014-15 as per MIDH norms and the details are as follows: (i) Cutting and removal of all old, senile, unproductive and disease advanced palms: A subsidy @ Rs.1000 per palm, subject to a maximum of Rs.32000/ha is provided for cutting and removal of old, senile, unproductive and disease advanced palms. (ii) Rejuvenation of the existing coconut palms by Integrated Management: For adoption of Integrated management practices a subsidy of Rs.17500 /ha is provided in two installments of Rs.8750/- each.(iii) Assistance for replanting: For replanting, a subsidy of Rs.40/- per seedling subject to a maximum of Rs 4,000/- ha is extended. During the current year it is proposed to implement the scheme in the state of Kerala, Karnataka and Tamil Nadu. An amount of Rs.4000 lakhs allocated under the scheme during 2016-17.

5.50 During the current financial year, total area covered is 3464.34 ha comprising 8709 farmers. A total of 47721 palms have removed. As on 11.11.2016 an amount of Rs.60.74 lakh has utilized under this scheme.

D. Coconut Palm Insurance Scheme (CPIS)

5.51 The Coconut Palm Insurance Scheme intends to provide Insurance coverage to coconut crop. Under the scheme all healthy nut bearing palms in the age group from 4 years to 60 years are eligible to get insurance coverage against natural perils leading to death / loss of palm becoming unproductive. 50% of the premium is borne by the Board and balance is shared between the State Govt and Farmers @ 25% each. The premium for the category of palms belonging to the age group of 4-15 is Rs. 9 and that for 16-60 is Rs.14/- . The insured amount for the corresponding categories is Rs. 900 and 1750 respectively. The scheme is implemented in selected districts of all coconut growing states. An amount of Rs.40 lakhs is earmarked under the scheme for the year 2016-17 to cover major coconut growing States.

Budget Outlay for 2016-17 under Coconut Palm Insurance Scheme

COCONUT PALM INSURANCE SCHEME (NCIP) - State wise fund allocation 2016-17

States/UT's	Physical Target		Financial Outlay (Rs in lakhs)			
	No: of Palms in lakh	Area in ha	CDB share (50 %) of premium	State Government share (25 %) of premium	Farmer's share (25 %) of premium	Total Premium amount
Kerala	0.71	476.19	5.00	2.50	2.50	10.00
Tamil Nadu	1.43	1142.86	10.00	5.00	5.00	20.00
Karnataka	0.71	571.43	5.00	2.50	2.50	10.00
Andhra Pradesh	1.43	1142.86	10.00	5.00	5.00	20.00
Maharashtra	0.71	571.43	5.00	2.50	2.50	10.00
West Bengal	0.29	228.57	2.00	1.00	1.00	4.00
Odisha	0.29	228.57	2.00	1.00	1.00	4.00
Goa	0.07	57.14	0.50	0.25	0.25	1.00
Gujarat	0.07	57.14	0.50	0.25	0.25	1.00
**Total**	**5.71**	**4476.19**	**40.00**	**20.00**	**20.00**	**80.00**

*N.B: in Kerala @ 150 palms / Ha, for all other state @ 125 palms/ Ha

E. 'Kera Suraksha' Insurance Scheme for Coconut Tree Climbers (CTC)

5.52 The 'Kera Suraksha' Insurance Scheme provides insurance coverage to the coconut tree climbers @ Rs.2 lakh against 24 hours against accident related risk including death. The annual premium of the policy is only Rs.92/- of which 75% is borne by the Board and 25% is contributed by the CTC. The scheme is implemented in all coconut growing states.

5.53 An amount of Rs.8 lakhs is allocated under the scheme for the year 2016-17 to cover maximum climbers in major coconut growing States. So far 2133 palm climbers are insured under the scheme.

F. Field Research

5.54 The Board initiated in house field research activities for conducting applied research activities in the DSP Farms of the Board. The researchable areas identified are standardization of quality parameters of seedlings, screening of mother palms, demonstration and standardization of traditional wisdom of farmers for pest and disease management, study on efficacy of Ayurvedic medicine on root wilt disease, strengthening biological control of pest and disease and study the effect of climate change on yield of coconut.

5.55 An amount of Rs.20 lakhs is earmarked under the scheme for the year 2016-17 to continue in- house research & development.

New Activities

a. Centre of Excellence (CoE) in Coconut: Coconut Development Board proposed to establish Centre of Excellence (CoE) in coconut

5.56 Basic objectives of establishing CoE will be to act as a Demonstration and training centre for farmers, entrepreneurs, staff of CDB and State Department of Agriculture/ Horticulture; large scale production of coconut seedlings; sharing best practices in coconut. CDB propose to strengthen DSP farms of the Board by creating infrastructure for farmers training, strengthening mother gardens, irrigation facilities, parasite breeding lab etc under the scheme. It is proposed to implement the scheme under MIDH center of excellence scheme with financial assistance of Rs. 605 lakhs

b. Establishment of new Coconut Orchards:

5.57 The Coconut Development Board proposed to implement a new scheme "Establishment of new coconut orchard" under MIDH during the year 2016-17. The objective of the scheme is to improve the production and productivity of coconut by establishing coconut orchards with high yielding varieties released by research institutes for different agro climatic conditions. The scheme is implementing by Coconut Development Board through Farmer Producers Organization (FPO) in all coconut growing states including scheduled and hilly areas. The financial assistance @ 25% of cost of cultivation for three years limited to Rs. 40,000 per ha is extended under the scheme. The subsidy is extended in two annual equal installments. During the year 2016-17 it is proposed to implement 250 ha in scheduled/hilly areas. A total out lay of Rs.40 lakhs is proposed for the year 2016-17. As on 11.11.2016 an amount Rs.15.52 lakh has utilized under this scheme.

Directorate of Arecanut and Spices Development, Calicut

5.58 The Directorate of Arecanut and Spices Development (DASD) is a subordinate office under Ministry of Agriculture & Farmers' Welfare, Government of India, to look after the development of Spices, Arecanut, Betel

vine and Aromatic plants at National level. The Directorate monitors the development programmes implemented by the states in spices under MIDH. The Directorate is directly involved in the production and distribution of quality planting material of high yielding varieties of spices and technology transfer programmes in association with State Agricultural Universities and Central Institutes across the country. During the year 2016-17, the directorate had an outlay of Rs. 11 crores under MIDH. DASD has launched a website www.spicenurseries.in to create a platform where both the nursery owners and farmers are brought together to share the information on availability of planting material. To improve the quality of planting material disbursed through various nurseries, the Directorate is also engaged in accreditation of nurseries. On 21st - 22nd April 2016, the Directorate organised a National level seminar at Calicut on 'Planting material production in spices' to commemorate the Golden Jubilee year of the directorate. Innovative technologies such as single bud planting and protray method of nursery raising in ginger and turmeric had been promoted by the directorate. Special programme on micro-rhizome production has been undertaken in association with Tamilnadu Agriculture University, Kerala Agriculture University and Indian Institute of Spices Research to promote disease free seed material in these crops.

Directorate of Cashewnut and Cocoa Development, Kochi

5.59 The Directorate of Cashewnut and Cocoa Development is implementing cashew & cocoa development programmes viz. New plantation development, replanting / rejuvenation of cashew plantations, front line technology demonstrations, upgradation of nursery infrastructure, strengthening of data base, human resource development, publicity for crop promotion under MIDH for the development of these two crops during 2016-17. The DCCD acts as a nodal agency for accreditation of existing cashew / cocoa nurseries. The Salient achievements under the scheme during 2016-17 are as follows:

- Under the scheme for new plantation development, an additional area aof 1200 ha under cashew and 1700 ha under cocoa with clones of high yielding varieties has been covered. Around 3000 farmers benefitted under the scheme out of which 30% will be women beneficiaries.
- 1085 ha old and senile cashew plantations owned by corporation of major cashew growing states have been replanted with high yielding varieties.
- 31 cashew / cocoa nurseries were given accreditation by the Directorate based on the assessment made by the expert team constituted for this purpose.
- As part of publicity and crop promotion activities, one National level seminar, 4 field days and 31 district level seminars on cashew and cocoa and 3 workshops cum exhibitions under SAGY will be organized in various states in association with State Agricultural Universities, Corporations, ICAR institutes and NGO's.
- 1550 farmers and entrepreneurs will be trained on cocoa and cashew farming practices in association with SAU's and ICAR Institutes.
- Under the HRD programme, 1000 unemployed women will be given training on preparation of value added edible products from cashew apple.
- The Directorate has prepared citizen / client charter in line with performance monitoring and evaluation system and sevottam guidelines to place in the website.

Central Institute of Horticulture, Medziphema, Nagaland

5.60 Central Institute of Horticulture was established in the year 2006 for the holistic development of horticulture sector in the North East Region. The main thrust areas of the Institute are refinement and demonstration of identified technologies pertaining to the region; production and supply of quality seed and planting material; training and capacity building of state govt. officials, field functionaries and farmers on different aspects of horticulture development including organic farming, monitoring of centrally sponsored programmes in the area of horticulture, post harvest management, processing, value addition, marketing and agribusiness promotion. The salient achievements of the institute during 2016-17 are as below:-

Production of Quality Planting Material

5.61 Raising of rootstock (approx. 18000 plants) for crops such as citrus, cashewnut, guava, mango and rose was done for further multiplication. During 2016-17, the Institute has propagated Guava (var. L-49-2762 nos., Sweta-943 nos., Allahabad Safeda-1990 nos., Lalit-600 nos.), Citrus (var. Khasi Mandarin-2815 nos.), Cashew (var. H-1608-420 nos, BBSR-1- 250 nos., H-2/16- 60 nos., VRI-3- 165 nos.), Sweet Orange (var. Mosambi) -514 nos.

Demonstration of Production Technologies

Establishment of demonstration plots in NE states

5.62 The activities carried out and crops grown for off farm demonstration during the year 2016-17 are establishment of demonstration plot of mango and Litchi at Sechu Zubza, Kohima District, Nagaland in an area of 2 ha, establishment of demonstration plot of mango and Litchi at Thoubal District, Manipur in an area of 2 ha, establishment of demonstration plot of mango and Khasi Mandarin at Karbi Anglong, Assam in an area of 2 ha. The Institute has also distributed planting material to the farmers for establishment of demonstration plot.

Nursery Accreditation

5.63 11 horticultural nurseries in North East region (10 nurseries in Meghalaya & 1 nursery in Arunachal Pradesh) was assessed by the Accreditation team and were assigned star rating to those nurseries.

Certificate Course:

5.64 03 numbers of the courses were undertaken during the period on following subjects for rural youth and women to promote the entrepreneurship development in the field of horticulture in NE region.

- Certificate Course on Protected Cultivation of Horticulture Crops.
- Certificate course on Organic Farming and Certification.
- Certificate course on Nursery Management

CIH is now authorized training partner of Agriculture Skill Council of India (ASCI) and is actively engaged in skill development training in various skill sets in horticulture sector.

Other Extension Activities:

5.65 The Institute has organized 19 farmer's trainings which were attended by 805 farmers and 2 training for trainers (76 officials) in identified areas of horticulture in the region. The institute has organised 3 numbers of exhibition & farmers meet and 4 numbers of seminars and conferences for the farmers and extension workers.

Glimpses of Activities carried out in CIH, Nagaland Production of quality planting material

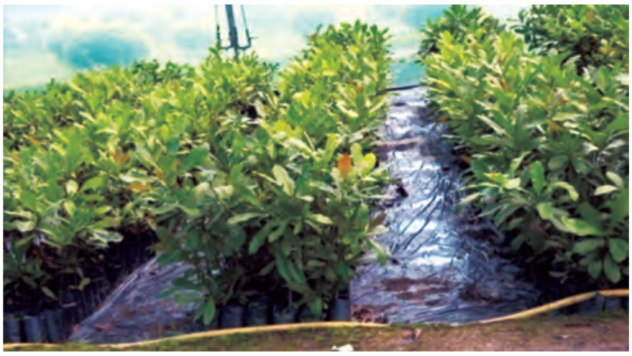
Raising of cashew rootstock

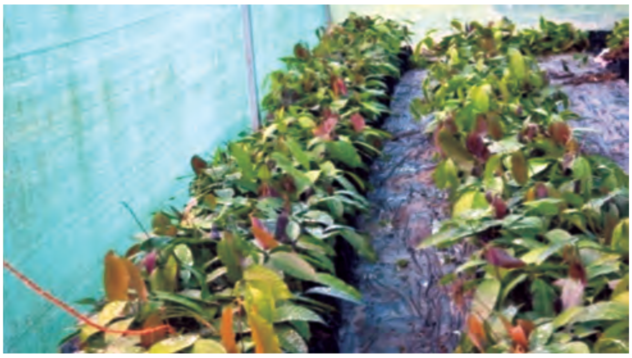
Raising of mango rootstock

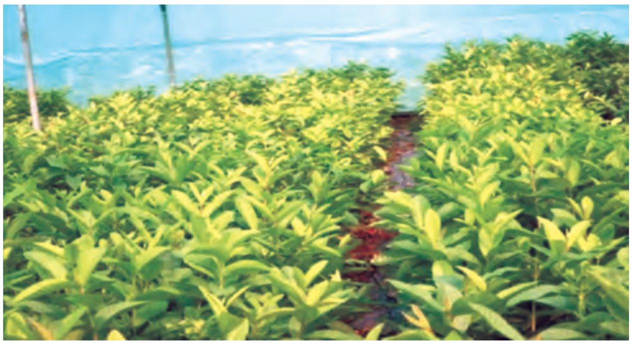
Raising of guava rootstock

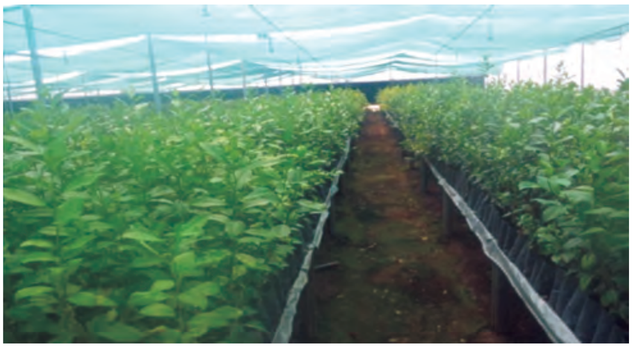
Raising of citrus rootstock

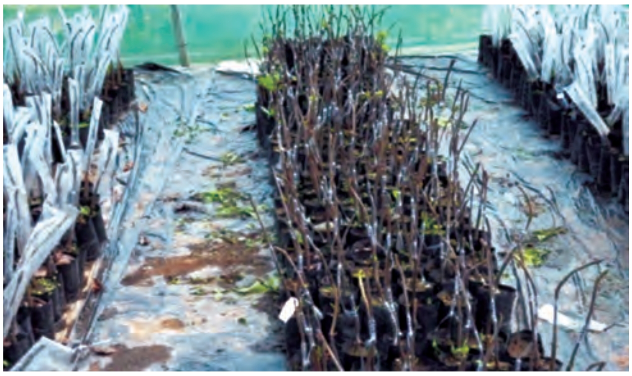
Soft wood grafting in cashew plants

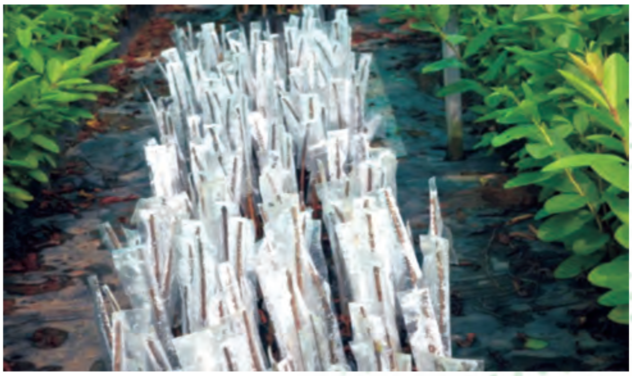
Wedge grafting in guava plants

Capacity Building Programme conducted by CIH

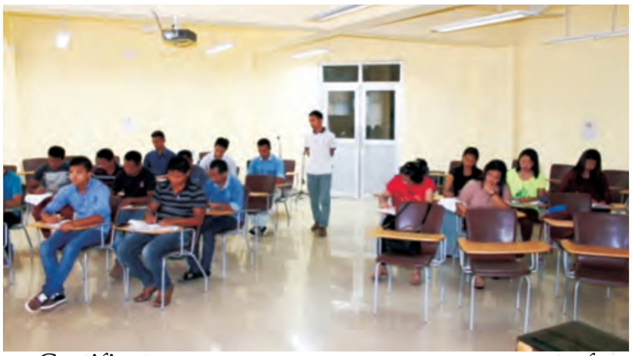
Certificate course on nursery management of horticultural crops

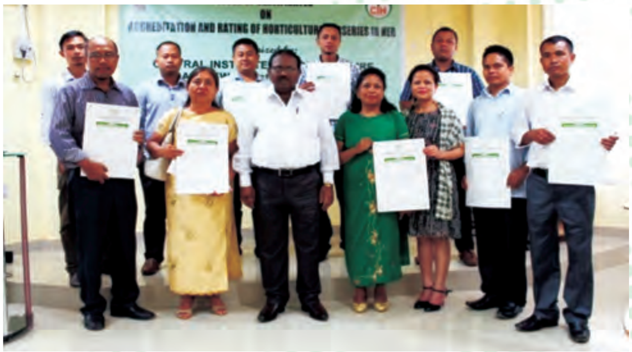
Issue of certificates to 11 horticultural nurseries in North East region

Capacity Building Programme conducted by CIH

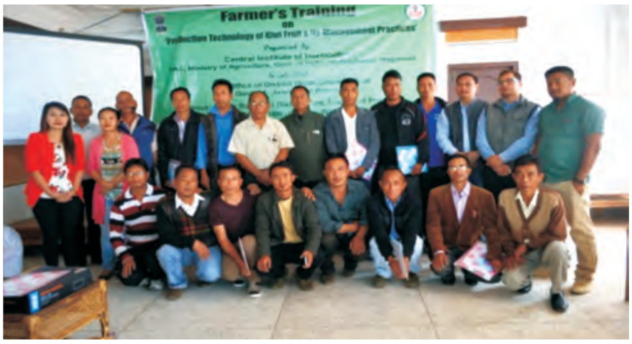

Exposure trip cum training for Phek farmers, Nagaland at Ziro, Arunachal Pradesh.

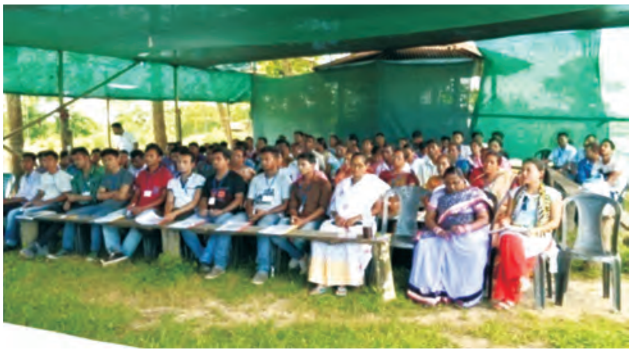

Farmers training in Manipur

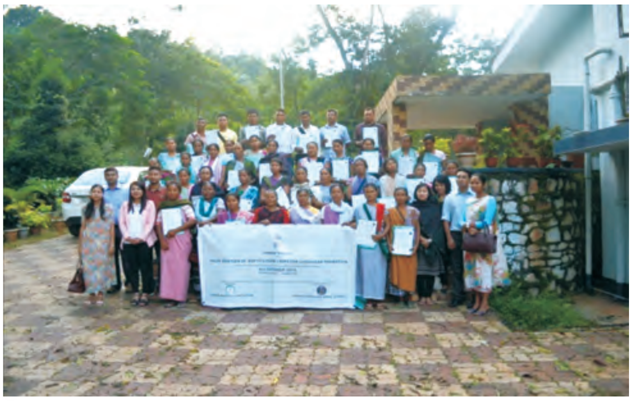

Farmers training in Meghalaya

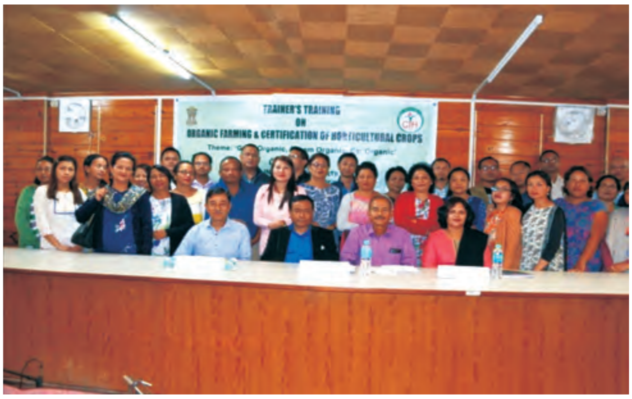

Trainers training in Meghalaya

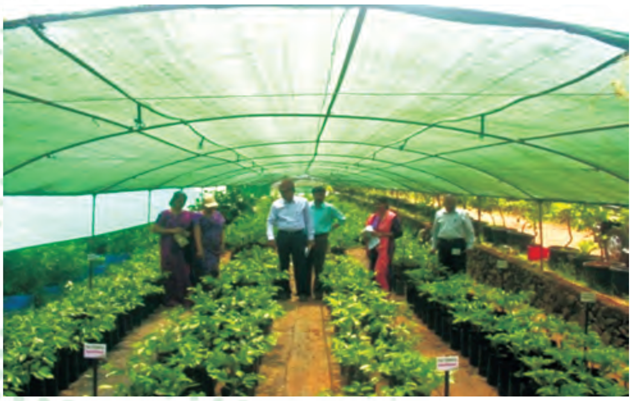

DASD Accreditation Team visiting spices nursery

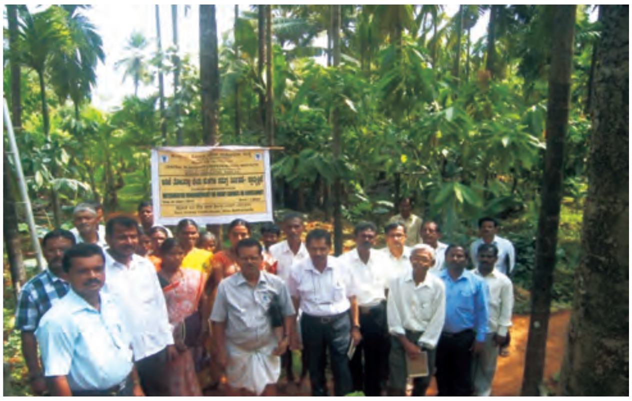

Demonstration Plot on Arecanut Root Grub Management

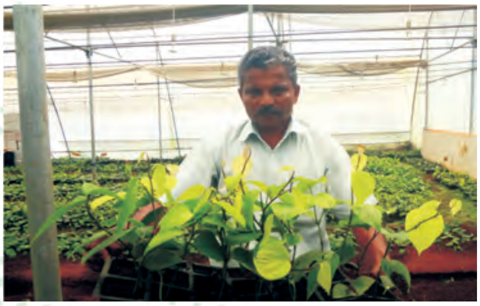

Protray raised black paper cuttings

Chapter 6

National Mission on Oilseeds and Oil Palm (NMOOP)

6.1 Vegetable Oil constitutes an important part of our daily diet being source of energy, essential fatty acids and amino acids. Domestic consumption of edible oils has increased substantially over the years and has touched the level of 24.16 million tonnes in 2014-15 (Nov-Oct) and is likely to increase further with enhancement in income and population against the domestic availability of 9.58 million tonnes. Considering the importance of oilseeds, various oilseeds development schemes have been funded by the Government for encouraging cultivation of oilseeds including Oil Palm.

6.2 The Centrally Sponsored Scheme of Integrated Scheme of Oilseeds, Oil Palm and Maize (ISOPOM), which was launched in 2004-05 and remained under implementation till March, 2014, Oil Palm Area Expansion (OPAE) programme, a sub-scheme of RKVY implemented during 2011-12 to 2013-14 have made significant contribution in increasing the oilseeds production and area expansion under oil palm. Implementations of these schemes have given fillip in augmenting the availability of vegetable oil in the country.

6.3 The scheme of ISOPOM, Tree Borne Oilseeds (TBOs) and Oil Palm Area Expansion (OPAE) programme have been restructured into National Mission on Oilseeds and Oil Palm (NMOOP) during 12th Plan and launched during 2014-15 and are implemented in 28 States. The sharing pattern of NMOOP between GOI and States is 60:40 for general states and 90:10 for NE and hilly states. Few components especially seed production & distribution, minikit of oilseeds for farmers, R&D support, FLDs by KVKs/ICAR etc. are on 100% funding for Central Agencies and ICAR/SAUs.

Oilseeds Scenario

6.4 The diverse agro-ecological conditions in the country are favorable for growing nine annual oilseeds which include 7 edible oilseeds viz. groundnut, rapeseed-mustard, soybean, sunflower, sesamum, safflower and niger and two non-edible oilseeds, castor and linseed.

6.5 Oilseeds are raised mostly under rainfed conditions and important for the livelihood of small and marginal farmers in arid and semi arid areas of the country. The production of oilseeds has increased from 24.35 million tonnes in 2004-05 to 27.51 million tonnes in 2014-15 and 25.30 million tonnes in 2015-16. The yield of oilseeds, which was 885 kg. per hectare in 2004-05 increased to 1037 kg per hectare in 2014-15 and 968 kg per hectare in 2015-16 (as per 4th Advance Estimates). The highest production and yield of oilseeds were achieved during the year 2013-14 i.e. 32.75 million tonnes and 1167 kg per year, respectively. The acreage of oilseeds has slightly decreased during 2014-15. During 2014-15 and 2015-16, major oilseeds producing States experienced late monsoon at the time of sowing, insufficient rain during crop growing phase, untimely rain during pod maturity stage, yellow mosaic virus in soybean. As per the 1st Advance estimate of DES during Kharif 2016-

17, oilseeds production is 23.36 million tones which is much higher than Kharif 2014-15 and 2015-16.

6.6 The area, production and yield of oilseeds during last three years are as under:

(Area in million hectare, Production in million tonnes & Yield in Kg/ha)

Year	Kharif			Rabi			`Total		
	Area	Prod.	Yield	Area	Prod.	Yield	Area	Prod.	Yield
2004-05	17.24	14.15	820	10.28	10.20	993	27.52	24.35	885
2014-15	18.21	19.22	1056	7.39	8.32	1130	25.73	27.51	1037
2015-16*	18.85	16.59	880	7.29	8.71	1195	26.13	25.30	968
2016-17@	18.75	23.36	1246						

*As per 4th Advance Estimates.

@As per 1st Advance Estimates (only Kharif).

6.7 The crop wise average production of nine major oilseeds during the XII plan period and for the year 2012-13 to 2016-17are as follow:

(Production in million tonnes)

S. No	Crop	Average XI Plan (2007-08 to 2011-12)	2012-13	2013-14	2014-15	2015-16	2016-17 @
1	Groundnut	7.40	4.70	9.71	7.40	6.77	6.50
2	Castorseed	1.38	1.96	1.73	1.87	1.65	1.73
3	Sesamum	0.74	0.69	0.72	0.83	0.87	0.68
4	Nigerseed	0.11	0.10	0.10	0.08	0.08	0.10
5	Rapeseed & Mustard	6.89	8.03	7.88	6.28	6.82	0.00
6	Linseed	0.16	0.15	0.14	0.16	0.13	0.00
7	Safflower	0.18	0.11	0.11	0.09	0.06	0.00
8	Sunflower	0.93	0.54	0.50	0.43	0.33	0.13
9	Soyabean	11.16	14.67	11.86	10.37	8.59	14.22
Total Nine Oilseeds		28.93	30.94	32.75	27.51	25.30	23.36

@As per 1st Advance Estimates (only Kharif).

Objective of NMOOP

6.8 National Mission on Oilseeds and Oil Palm (NMOOP) comprises of three Mini Missions (MM) one each for Oilseeds (MM-I), Oil Palm (MM-II) and Tree Borne Oilseeds (MM-III). The Missions with specific targets of 12th Plan is detailed below:

Mini Mission (MM)	Target of 12th Plan
MM I on Oilseeds	Achieve Production of 35.51 million tonnes and productivity of 1328 kg/ha of oilseeds from the present average production & productivity of 28.93 million tonnes and 1081 kg/ha during the 11th Plan period respectively.
MM II on Oil Palm	Bring additional 1.25 lakh hectare area under oil palm cultivation through area expansion approach in the States and productivity of fresh fruits bunches (FFBs) from 4927 kg per ha to 15000 kg per ha.
MM III on TBOs	Enhance seed collection of TBOs

Strategy for NMOOP

6.9 The strategy to implement the proposed Mission includes increasing Seed Replacement Ratio (SRR) with focus on Varietal Replacement; increasing irrigation coverage under oilseeds, diversification of area from low yielding cereals crops to

oilseeds crops; inter-cropping of oilseeds with cereals/pulses/sugarcane; use of fallow land after paddy/potato cultivation; expansion of cultivation of Oil Palm, increasing availability of quality planting materials of Oil Palm & TBOs Maintenance cost and Inter-cropping during gestation period of oil palm and TBOs would provide economic return to the farmers when there is no production. The scheme would be implemented in a mission mode through active involvement of all the stakeholders. Fund flow would be monitored to ensure that benefit of the Mission reaches the targeted beneficiaries in time to achieve the targeted results.

6.10 Mission wise States under NMOOP are given below:

(i) **Mini Mission – I on Oilseeds**: Andhra Pradesh, Arunachal Pradesh, Assam, Bihar, Chhattisgarh, Gujarat, Haryana, J&K, Jharkhand, Karnataka, Madhya Pradesh, Maharashtra, Manipur, Meghalaya, Nagaland, Odisha, Punjab, Rajasthan, Sikkim, Tamil Nadu, Telangana, Tripura, Uttar Pradesh, Uttarakhand and West Bengal.

(ii) **Mini Mission-II on Oil Palm:** Andhra Pradesh , Chhattisgarh, Goa, Gujarat, Maharashtra, Mizoram, Karnataka, Kerala, Odisha, Tamil Nadu, Arunachal Pradesh, Assam, Bihar, Manipur, Meghalaya, Nagaland, Sikkim, Tripura and West Bengal.

(iii) **Mini Mission - III on TBOs** :Andhra Pradesh, Assam, Arunachal Pradesh, Bihar, Chhattisgarh, Gujarat, Goa, Haryana, Himachal Pradesh, Jammu & Kashmir, Jharkhand, Karnataka, Kerala, Madhya Pradesh, Maharashtra, Manipur, Meghalaya, Mizoram, Nagaland, Odisha, Punjab, Rajasthan, Sikkim, Tamil Nadu, Tripura, Uttar Pradesh, Uttarakhand and West Bengal.

6.11 All the North Eastern States have been included under all the three Mini Missions of NMOOP.

6.12 Mission-wise allocation of funds under NMOOP to the States during 2016-17 are as follow:

Amount (Rs. in crore)

Mission	Allocation
Mini Mission-I	378.18
Mini Mission-II	79.99
Mini Mission-III	5.45
R&D institutions and Central Agencies	114.84
**Total**	**578.46**

Mini Mission-I (Oilseeds)

6.13 State-wise Area, Production and Productivity of Oilseeds during 2010-11 to 2015-16 are at **Annexure-6.1**. Under this Mission, financial assistance is being provided for Seed Components (production & distribution of certified seeds & minikits, variety specific targeted seed production); Inputs (Plant Protection Equipments/ eco friendly, light-trap, Bio-pesticides, Distribution of Micro-nutrients, bio-fertilizers, improved farm implements, pipes, sprinklers, seed storage bins, seed treatment drums) and Transfer of Technology (Block demonstrations, Frontline demonstrations, farmers and extension workers training etc.). The scheme is being implemented through State Department of Agriculture. Mini Mission – I is also supporting ICAR institutes for undertaking FLDs on oilseeds crops and ICAR-KVKs for organizing cluster demonstration on oilseeds.

Mini Mission-II (Oil Palm)

6.14 Oil palm is comparatively a new crop in India and is the highest vegetable oil yielding perennial crop. With quality planting material, irrigation and proper management, there is potential of 20-30 MT Fresh Fruit Bunches (FFBs) per hectare after

attaining the age of 5 years which would yield 4-5 MT of palm oil and 0.4-0.5 MT palm kernel oil (PKO). In comparative terms yield of palm oil is 5-6 times the yield of edible oil obtainable from traditional oilseeds.

6.15 Oil palm development programme was started in India during 1991-92 and continued till 2002-03, mainly for area expansion under oil palm in selected states. Subsequently, in order to increase the production & productivity of oilseeds and oil palm cultivation, Integrated Schemes of Oilseeds, Pulses, Oil Palm and Maize (ISOPOM) was implemented from 2004-05 to 2013-14 in 10 states of the country. Besides, area expansion of oil palm was also supported through Oil Palm Area Expansion (OPAE) programme under RKVY from 2011-12 to 2013-14.

6.16 In order to bring more area under oil palm cultivation, Government of India has launched a new National Mission on Oilseeds and Oil Palm (NMOOP) during the XIIth Five Year Plan under which Mini Mission-II is dedicated to oil palm area expansion, is being implemented in 12 states during 2016-17.

6.17 Under the Mission, financial assistance are being provided to the farmers @ 85% cost of the planting material and @ 50% cost of the other components like maintenance cost of new plantations for four years, installation of drip-irrigation systems, diesel/electric pump-sets, bore-well/water harvesting structures/ponds, inputs for inter-cropping in oil palm (during gestation period), construction of vermi-compost units and purchasing of machinery & tools etc.

6.18 100% support is being provided to Indian Institute of Oil Palm Research (IIOPR), Pedavegi, Andhra Pradesh for Research & Development on oil palm.

6.19 All these developmental efforts have resulted in area expansion under oil palm from 8585 ha in 1991-92 to 3.00 lakh ha by the end of 2015-16. Similarly, the Fresh Fruit Bunches (FFBs) production and Crude Palm Oil (CPO) have increased from 21,233 MT and 1,134 MT respectively (1992-93) to 12.83 lakh and 2.17 lakh MT respectively during the year 2015-16. At present, Andhra Pradesh, Karnataka, Tamil Nadu, Mizoram and Odisha are major oil palm growing states. The State-wise details of area achieved under oil palm cultivation and production of FFBs and CPO up-to the year 2015-16 are given below:

S. No.	State	Total Area Coverage (in ha.) up-to March, 2016	Production (in MT)	
			FFBs	CPO
1.	Andhra Pradesh	1,50,530	11,44,092	1,93,562
2.	Telangana	16,239	63,508	11,289
3.	Karnataka	41,431	14,740	2,538
4.	Tamil Nadu	29,510	7,810	1,222
5.	Gujarat	5,054	523	0
6.	Goa	953	3,217	581
7.	Odisha	18,484	4,569	618
8.	Tripura	530	0	0
9.	Assam	570	0	0
10.	Kerala	5,769	40,611	7,016
11.	Maharashtra	1,474	0	0
12.	Mizoram	25,741	3,753	432
13.	Chhattisgarh	2,162	0	0
14.	Andaman& Nicobar	1,593	0	0
15.	Arunachal Pradesh	330	0	0
16.	Nagaland	140	0	0
	**Total**	**3,00,510**	**12,82,823**	**2,17,258**

Mini Mission-III - Tree Borne Oilseeds (TBOs)

6.20 Mini Mission-III covers Tree Borne Oilseeds (TBOs) programme with the objectives of enhancing seed collection of TBOs and augmenting elite planting materials. Assistance Under MM-III, is provided to promote 11 TBOs namely Simarouba, Neem, Jojoba, Karanja, Mahua, Wild apricot, Cheura, Kokum, Jatropha, Tung and Olive having capability to grow and establish in varied agro-climatic conditions as well in the waste land of the country.

6.21 The interventions under MM-III are (i.) integrated development of nurseries & plantation on the wasteland, (ii). maintenance of TBOs plantation, (iii) incentives for undertaking intercropping with TBOs, (iv) research and development, (v) distribution of pre-processing, processing and oil extraction equipment, (vi) support to TRIFED, (vii) training of farmers, (viii) training of extension workers and (ix) local initiatives/contingency.

6.22 During 2016-17, MM-III programme on Tree Borne Oilseeds is implemented in the states of Arunachal Pradesh, Chhattisgarh, Jammu & Kashmir, Madhya Pradesh, Maharashtra, Mizoram, Rajasthan, Tamil Nadu, Tripura and Uttar Pradesh.

R& D Projects under NMOOP

6.23 The R&D projects on oilseeds, oil palm and Tree Borne Oilseeds programme are also being supported under MM-III of NMOOP. The list of R&D projects supported under NMOOP during 2016-17 are as under:

S. No.	Institutions/ Agencies	Activities
1.	GBPUA&T, Pant Nagar, Uttrakhand	Productivity enhancement of R&M in Uttarakhand (MM-I)
2.	IGKVV, Raipur, Chattisgarh	Block demonstration of soybean in Chhattisgarh (MM-I)
3.	ICAR-IIOR, Hyderabad	Briding yield gap of sunflower and sesame (MM-I)
4.	ICRISAT, Hyderabad	Release of high oil and oleic groundnut varieties (MM-I)
5.	ICAR-IIOPR, Pedavagi, A.P	Empowerment of stakeholders on Oil Palm Technologies (MM-II)
6.	ICAR-CRIDA, Hyderabad	Evaluation and upscalling of karanja and simaroba (MM-III)
7.	CRDT- IIT, Delhi	Design and development of post-harvest equipment for simarouba (MM-III)
8.	SKUAST - Sri Nagar, J&K	Evaluation of wild apricot for oil yield (MM-III)
9.	ICAR-CAFRI, Jhansi, U.P	Integrated development of jatropha and karanja (MM-III)
10.	Kumaun University, Nainital, Uttrakhand	Promotion of cheura and wild apricot (MM-III)

Central Sector Programme

Seed production, distribution, minikits and FLDs on Oilseeds during 2016-17

6.24 In order to encourage the adoption of newly released varieties and improved agro-techniques in oilseed crops support is provided under NMOOP to Central Agencies viz. NSC, KRIBHCO, HIL, NAFED and IFFDC for procurement of breeder seed (B/S), production of foundation seed (F/S) and production certified seed (C/S), distribution of certified seed (D/S) and supply of seed minikits. In addition, support is also provided for conduct of Front Line Demonstrations (FLDs) through ICAR/SAUs network. During kharif-2016 and rabi 2016-17, a total of 2,80,269.64 qtls and 88358.41 qtls of various categories of seeds (B/S,F/S,C/S) respectively, were produced and 70,991.70

qtls seeds were distributed. Seed minikits of 7 oilseeds crops totalling 1658530 nos. and of 7 oilseeds crops totalling 1427520 nos. were supplied during kharif-2016 and rabi 2016-17 respectively, through Central Seed Producing Agencies.

6.25 A total of 2616 FLDs on improved varieties and agro-techniques of five oilseeds and 4664 FLDs on seven oilseeds during kharif-2016 and rabi 2016-16 respectively, were conducted through ICAR-AICRP network. During rabi 2016-17 a total of 51665 cluster FLDs have been planned through 423 KVKs of ICAR in different states covering (groundnut, soybean, rapeseed-mustard, sunflower, safflower, linseed, sesame, niger and castor) kharif/rabi/summer 2016-17 oilseeds.

National Oilseeds and Vegetable Oils Development (NOVOD) Board:

6.26 Due to restructuring of Tree Borne Oilseeds (TBOs) programme into National Mission on Oilseeds and Oil Palm (NMOOP) and in view of the ongoing wind-up process of the Board, no developmental activity was under taken by the Board during the year. Subsequently, the Repealing and Amending Act, 2016 (No. 23 of 2016) dated 6th May, 2016 has repealed the National Oilseeds and Vegetable Oils Development (NOVOD) Board Act, 1983 and accordingly the Board has been wind-up vide Ministry of Agriculture and Farmers Welfare notification no. S.O. 2925(E) dated 8th September 2016. A Surplus Staff Establishment (SSE) has been created under the Oilseeds division of Department of Agriculture, Cooperation and Farmers Welfare vide order F.No 2-2/2013-C.A VI, dated 9th September 2016. As per the said order all the employees of erstwhile NOVOD Board have been transferred to the SSE w.e.f. 9th September 2016 and the services of the employees of erstwhile NOVOD Board are being utilized in the various sections/ divisions of this Ministry and in National Horticulture Board.

6.27 An allocation of RS. 3.00 crore has been made under MM-III component of NMOOP to meet the salary of the employees and the administrative expenses of the NOVOD Board, during 2016-17.

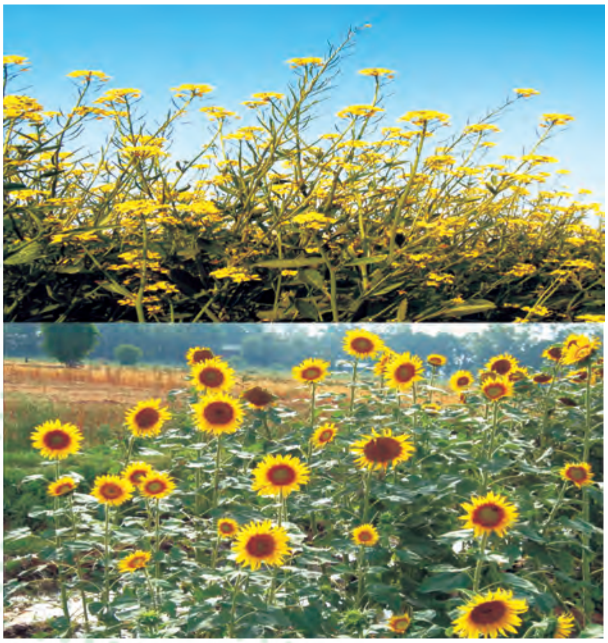

******

Chapter 7

National Mission for Sustainable Agriculture (NMSA)

7.1 National Mission for Sustainable Agriculture (NMSA) is one of the eight Missions outlined under National Action Plan on Climate Change (NAPCC). The Mission aims at promoting sustainable agriculture through seventeen deliverables focusing on ten key dimensions of Indian Agriculture. During 12th Five Year Plan, these dimensions have been embedded and mainstreamed into Missions/Progammes/ Schemes of Department of Agriculture, Cooperation & Farmers Welfare (DAC&FW) through a process of restructuring and convergence. NMSA as a programmatic intervention made operational from the year 2014-15 aims at making agriculture more productive, sustainable, and remunerative and climate resilient by promoting location specific integrated/composite farming systems; soil and moisture conservation measures; comprehensive soil health management; efficient water management practices and mainstreaming rainfed technologies.

Major Components of NMSA are :

- **Rainfed Area Development (RAD):** RAD focuses on Integrated Farming System (IFS) for enhancing productivity and minimizing risks associated with climatic variabilities. Under this system, crops/cropping system is integrated with activities like horticulture, livestock, fishery, agro-forestry, apiculture etc. to enable farmers not only in maximizing farm returns for sustaining livelihood, but also to mitigate the impacts of drought, flood or other extreme weather events with the income opportunity from allied activities during crop damage.
- **Soil Health Management (SHM):** SHM is aimed at promoting location as well as crop specific sustainable soil health management including residue management, organic farming practices by way of creating and linking soil fertility maps with macro-micro nutrient management, appropriate land use based on land type.

Rainfed Area Development (RAD):

7.2 During 2016-17, RAD is being implemented in 27 States of the Country. Integrated Farming System (IFS) is being promoted under RAD in which activities like horticulture, livestock, fishery, agroforestry, value addition are to be taken up along with crops/cropping system. As on 31.12.2016, IFS activities of about 32740 ha have been covered comprising 4245 ha of livestock based farming system, 9727 ha of dairy based farming system, 1609 ha of fishery based farming system, 7408 ha of horticulture based farming system, 5105 ha of agro-forestry based farming system, 472 ha of silvi-pasture based farming system & 4174 ha of cropping system with peripheral plantations. Rs.157.14 crore has been released to the implementing states till 31st December, 2016.

Details of Rainfed Area Development (RAD) programmes being executed in North- Eastern States during 2016-17 (BE) are given below:

Schemes/ Programmes	Budget Allocation (Rs. In lakh)	Amount Released (Rs. In lakh) as on 31.12.2016	IFS activities covered (Area in ha) as on 31.12.2016
Rainfed Area Development (RAD)	3200.00	2517.02	5108

New Initiatives:

7.3 A dedicated website of National Mission for Sustainable Agriculture (NMSA) has been operationalized (http://nmsa. dac. gov. in/) to keep all information of the scheme in the public domain for access and use of all stakeholders.

Pradhan Mantri Krishi Sinchayee Yojana (PMKSY)

7.4 Pradhan Mantri Krishi Sinchayee Yojana (PMKSY) is being implemented with the objective of developing a long term solution for mitigating the effect of drought and increasing area under irrigation with Motto of Har Khet Ko Pani. This programme is being implemented in mission mode by three ministries with Ministry of Water Resources, RD&GR leading the mission.

7.5 With an outlay of Rs. 50,000 crore for a period of 5 years (2015-16 to 2019-20), the scheme has been conceived amalgamating ongoing schemes viz. Accelerated Irrigation Benefit Programme (AIBP) of the Ministry of Water Resources, River Development & Ganga Rejuvenation (MoWR, RD&GR), Integrated Watershed Management Programme (IWMP) of Department of Land Resources (DoLR) and the On Farm Water Management (OFWM) of Department of Agriculture, Cooperation and Farmers Welfare. The major objective of PMKSY is to achieve convergence of investments in irrigation at the field level, expand cultivable area under assured irrigation, improve on-farm water use efficiency to reduce wastage of water, enhance the adoption of precision-irrigation and other water saving technologies (More crop per drop), promote sustainable water conservation practices etc. The aim of PMKSY is not only the creation of assured irrigation but to create protected irrigation by using rainwater by "Jal Sanchay" and "Jal Sinchan".

7.6 Various committees, as envisaged in the operational guidelines of the scheme, viz. National Steering Committee under the Chairmanship of Hon'ble Prime Minister and National Executive Committee under the chairmanship of Vice chairman of NITI Aayog have been constituted. Department of Agriculture, Cooperation & Farmers Welfare has been assigned to implement Per Drop More Crop component of PMKSY to focus on water use efficiency enhancement and supplementary drought proofing measures. It has two sub components viz. Micro-irrigation and Other Interventions.

7.7 Union Cabinet in its meeting held on 27th July, 2016 approved implementation of PMKSY in a mission mode approach. The mission objective is to complete 99 major and medium irrigation projects having potential of 76.03 lakh ha in phases by Dec., 2019, including command area development. For completion of these projects in a mission mode, innovative funding mechanism through NABARD is envisaged. Mission is also responsible for overall coordination and outcome focused monitoring of all components of PMKSY for achieving its target. The Mission is administered by Ministry of Water Resources, River Development and Ganga Rejuvenation.

7.8 During 2016-17, as on 31.12.2016, an amount of Rs. 1211.75 cr and Rs.430.3 crore

has been released to states for implementation of Micro-irrigation activities and for Other Interventions activities respectively. Till 31.12.2016, an area of about 3.54 lakh ha. has been covered under Micro Irrigation.

New Initiatives

7.9 The website of PMKSY (http://www.pmksy.gov.in) with static features and Management Information System (MIS) with multi-nodal access features has been operationalized with integrating and capturing information of various Departments/ Ministries for PMKSY. District Irrigation Plans are cornerstone of planning and implementation of the scheme. 201 officers of All India Services have been trained so far at Premier National Institutes. So far 584 district irrigation plans have been prepared and preparation for other districts is underway.

Sub-Mission on Agro-forestry

7.10 To give specific focus on development of Agroforestry, Ministry of Agriculture & Farmers Welfare, Government of India, brought out the National Agroforestry Policy in 2014 to bring about coordination, convergence and synergy among various elements of agro-forestry scattered in various missions, programmes, schemes and agencies.

7.11 To take forward the recommendations of National Agroforestry Policy, a dedicated Sub-Mission on Agroforestry (SMAF) under the framework of National Mission for Sustainable Agriculture (NMSA) has been approved by Govt. of India with an outlay of Rs. 935.00 crore for a period of 4 years starting from 2016-17. The objectives of SMAF are:

- To encourage and expand tree plantation in complementary and integrated manner with crops and livestock to improve productivity, employment opportunities, income generation and livelihoods of rural households, especially the small farmers.
- To ensure availability of quality planting material like seeds, seedlings, clones, hybrids, improved varieties, etc.
- To popularize various Agroforestry practices/models suitable to different agro ecological regions and land use conditions.
- To create database, information and knowledge support in the area of agroforestry.
- To provide extension and capacity building support to agroforestry sector.

7.12 Liberal transit rules will be a precondition for availing the benefit of the Programme. During 2016-17, an amount of Rs.22.50 crore has been released to 8 States till 31.12.2016 for implementation of programme.

Other Programmes

7.13 Indo German Bilateral Cooperation (GIZ): Effective information exchange is a prerequisite to promptly respond to the needs of the farmers' for authentic knowledge to enable better adaptation to climate changes. Although a number of Agricultural Knowledge and Information Networks (AKINs) exist, there is lack of effectiveness of interventions at farm level i.e. transfer of climate resilient technologies, lack of availability of effective, timely/ dynamic/authentic and farmer-friendly sustainable agricultural knowledge. With this background, under Indo-German Bilateral Cooperation a technical project titled Climate Change Knowledge Network in Indian Agriculture (CCKN-IA) was launched in September, 2013 in three states of Maharashtra, Jharkhand and Odisha.

CCKN-IA aims to promote knowledge exchange among different stakeholders to establish and enhance linkages between climate change and sustainable development in agriculture.

7.14 Under programme, an open source platform called Network for Information on Climate (Ex) change (NICE) has been developed. This platform facilitates effective and efficient knowledge exchange at all levels. NICE has been developed based on open source web solution using semantic web technologies, that can be scaled and adapted to various knowledge needs - from agriculture, soil and water conservation, social security and others.

7.15 NICE integrates and allows existing multiple knowledge stakeholders from domains like meteorology, agriculture science, extension systems and others to share and adapt knowledge across multiple subject domains, to address local climate change adaptation needs. The system is iterative and enables two-way communication to link farmers' needs and knowledge providers, on a real time basis. The project uses multi-modal approach to exchange knowledge to farmers that includes simple one-pager documents, tablet applications, posters, videos, SMS and social media. In addition, project revitalizes existing extension systems, capacities and monitoring systems for effective development, dissemination as well as support adoption by farmers. By end of 31.12.2016 about 23,042 farmers and about 100 extension service providers in selected State have received the climate change adaptation knowledge information through NICE. Further, various trainings for experts, extension agents and farmers have been carried out on NICE, Climate Change adaptation and use of ICT, plant pest and disease management , cross sector knowledge like fishery, animal husbandry etc.

Integrated Nutrient Management

Assessment of Fertilizers:-

7.16 To ensure adequate availability of fertilizers, Department of Agriculture, Cooperation and Farmers Welfare conducts Zonal Conferences with all the States for every Kharif and Rabi season in order to assess the requirement of fertilizers in all the States. After consultation with States, Department of Fertilizers and Lead Fertilisers suppliers etc., the total requirement for each State is assessed for the season.

7.17 Therefore, the States prepare month-wise requirement and the same is forwarded to the Department of Fertilisers. A monthly supply plan based on the month-wise requirement is made by Department of Fertilisers for all States. The supply movement is jointly monitored by Department of Agricultur, Cooperation & Farmers Welfare and Department of Fertilisers with the States through weekly Video Conference

(a) **Kharif 2016 Season:** Requirement of major fertilizers viz, Urea, DAP, MOP, Complexes and SSP for Kharif 2016 season was assessed at 155.13 Lakh Metric Tones (LMT), 50.82 LMT, 17.42 LMT, 53.68 LMT and 30.60 LMT respectively.

(b) **Rabi 2016 Season:** Requirement of major fertilizers viz, Urea, DAP, MOP, Complexes and SSP for Rabi 2016-17 season was assessed at 161.34 Lakh Metric Toones (LMT), 49.77 LMT, 17.42 LMT, 49.87 LMT and 33.38 LMT respectively.

7.18 **Consumption of Chemical Fertilizers:-** Consumption of major chemical fertilizers along with N,P,K nutrients since 2001-02 is given below:-

(lakh tonnes)

Year	Urea	DAP	MOP	Complex	Nitrogen (N)	Phosphate (P)	Potash (K)	Total (N+P+K)
2001–02	199.17	61.81	19.93	49.63	113.10	43.82	16.67	173.60
2002–03	184.93	54.73	19.12	48.10	104.74	40.19	16.01	160.94
2003–04	197.67	56.24	18.41	47.57	110.77	41.24	15.98	167.99
2004–05	206.65	62.56	24.06	55.08	117.13	46.24	20.61	183.98
2005–06	222.97	67.64	27.31	66.94	127.23	52.04	24.13	203.40
2006–07	243.37	73.81	25.86	67.99	137.73	55.43	23.35	216.51
2007-08	259.63	74.97	28.80	65.70	144.19	55.15	26.36	225.70
2008-09	266.49	92.31	40.78	68.05	150.90	65.06	33.13	249.09
2009-10	266.74	104.92	46.34	80.25	155.80	72.74	36.32	264.86
2010-11	281.12	108.70	39.31	97.64	165.58	80.50	35.14	281.22
2011-12	295.65	101.91	30.29	103.95	173.00	79.14	25.75	277.90
2012-13	300.02	91.54	22.11	75.27	168.21	66.53	20.62	255.36
2013-14	306.00	73.57	22.80	72.64	167.50	56.33	20.99	244.82
2014-15	306.10	76.26	28.53	82.78	169.46	60.98	25.32	255.76
2015-16	306.35	91.07	24.67	88.21	173.72	69.79	24.02	267.53

Fertilizer Control Order (FCO), 1985:

7.19 In order to make available large variety of fertilizers to the farmers as per their soil requirement, different grades of fertilizers are notified under FCO Schedule-I (Part-A). At present 13 Straight Nitrogenous Fertilizers, 8 Straight Phosphatic Fertilizers, 5 Straight Potassic Fertilizers, 2 Sulphur Fertilizers, 19 NPK Complex Fertilizers and 18 NP Complex Fertilizers, 29 customized fertilizers, 21 Fortified Fertilizers, 18 Water Soluble Fertilizers and 17 Micronutrients are notified under FCO. The provision of tolerance limit in plant nutrient and physical parameters for various fertilizers is given in FCO Schedule-I (Part B).

7.20 To encourage use of organic and bio-fertilizers, bio fertilizers namely; Rhizobium, Azotobacter, Azospirillum, Phosphate Solubilizing Bacteria, Potash mobilizing Bacteria (KMB), Zinc Solubilizing Bacteria (ZnSB), Mycorrhizae, Acetobacter and Consortia of bio-fertilizers have been incorporated in FCO, 1985. Generalized specifications of organic manures and other organic fertilizers, namely, City compost, Vermi compost, Phosphate Rich Organic manure (PROM) and Phosphate Solubilizing Bacteria enriched Organic Manure are notified under FCO Schedule IV. Besides this, specification of Non-Edible De-Oiled cake/Caster-Oiled cake fertilizers is notified under FCO Schedule-V.

Quality Control of Fertilizers:

7.21 To ensure availability of standard quality to farmers, Fertilizer was declared as an Essential Commodity and Fertilizer Control Order, 1985 was promulgated under section 3 of Essential Commodities Act, 1955 to regulate trade, price, quality and distribution of fertilizers in India. Responsibility of enforcement of this Order has primarily been entrusted to State Governments and Central Government provides training facilities and technical guidance to States and supplements their efforts through random inspection of manufacturing units and their distribution network. Presently there are 81 laboratories in the country including 4 Central Government Laboratories. Analytical capacity and number of samples analysed and found Non Standard during last 5 years is shown in Table below:

Year	Number of Labs.	Annual Analytical Capacity	No. of Samples Analysed	% Non Standard Samples
2011-12	74	1,30,450	1,31,970	4.9
2012-13	75	1,42,621	1,33,872	5.1
2013-14	78	1,52,470	1,38,961	5.4
2014-15	78	1,68,536	1,35,522	5.0
2015-16	81	1,67,190	1,28,041	5.3

Soil Health Management (SHM)

7.22 Soil Health Management (SHM) is one of the most important interventions under National Mission for Sustainable Agriculture (NMSA). SHM aims at promoting Integrated Nutrient Management (INM) through judicious use of chemical fertilizers including secondary and micro nutrients in conjunction with organic manures and bio-fertilizers for improving soil health and its productivity; strengthening of soil and fertilizer testing facilities to provide soil test based recommendations to farmers for improving soil fertility; ensuring quality control requirements of fertilizers , bio-fertilizers and organic fertilizers under Fertilizer Control Order, 1985; up-gradation of skill and knowledge of soil testing laboratory staff, extension staff and farmers through training and demonstrations; promoting organic farming practices etc.

7.23 The components under Soil Health include trainings for fertilizer dealers, foreign nationals, fertilizer inspectors and fertilizer laboratory staff, setting up of new static Soil Testing Laboratories (STLs), setting up of new Mobile STLs, strengthening of existing STLs, setting up of new Fertilizer Quality Control Laboratory (FQCL), strengthening of FQCL apart from trainings and demonstrations on balanced use of fertilizers.

7.24 The components under organic farming include trainings for certification course on organic farming, refresher courses for analysts, field functionaries, trainers training, publishing of News Letters on organic farming and bio-fertilizers, setting up of fruit/vegetable/agro waste compost production units, setting up of bio-fertilizer production units, setting up of bio-fertilizer & Organic Fertilizer Quality Control Laboratories, promotion of organic inputs, training on organic farming, etc.

7.25 Under the scheme, setting up of 277 Soil Testing Laboratories (STLs), 3 Mobile STLs, Strengthening of 38 STLs, Strengthening of 8 FQCLs, 709 trainings, setting up of 1 Liquid Carrier based Biofertilizers productions unit, promotion of Micronutrients in 1000 ha and setting up 6308 Mini Soil Testing Labs have been approved during 2016-17 under SHM component.

7.26 A new scheme "Soil Health Card" has been approved for implementation during the remaining period of 12th Plan to provide 14 crore Soil Health Cards to the farmers in the country. Soil Health Card will provide information to farmers on soil nutrient status of their soil and recommendation on appropriate dosage of nutrients to be applied for improving soil health and its fertility. Soil health card will be issued every 2 years for all land holdings in the country so as to promote balanced and integrated use of plant nutrients. Under the scheme 239.73 lakh soil samples collected and 444.58 lakh soil Health Cards issued by States, as on 03.01.2017.

7.27 Balanced Use of Fertilisers: Ministry of Agriculture, Cooperation and Farmers Welfare, Department of Agriculture, Cooperation & Farmers Welfare is promoting soil test based balanced & judicious use of chemical fertilisers, biofertilizers and locally

available organic manures, like Farm Yard Manure (FYM), vermi-compost and green manure to maintain soil health and its productivity. In order to promote balanced fertiliser application, Government is providing grant for setting up / strengthening of soil testing laboratories, trainings and demonstrations on balanced use of fertilisers and promotion of micro-nutrients across various Plan periods. At present, there are 1414 Soil Testing Laboratories with analysing capacity of 1.95 crore samples per annum.

7.28 Soil Health Management (SHM): Ministry of Agriculture, Cooperation and Farmers Welfare, Department of Agriculture and Cooperation & Farmers Welfare is implementing Soil Health Management component under National Mission for Sustainable Agriculture. The financial assistance on various components as below is provided under SHM:

- Setting up of new Soil Testing laboratories (STL)(static/mobile/mini labs) and strengthening of existing Soil Testing laboratories
- Training of STL staff/extension officers/ farmers/field functionaries and field demonstrations on balanced use of fertilisers etc
- Promotion and distribution of micronutrients
- Setting up of new Fertilizer Quality Control Laboratories (FQCL) and strengthening of existing state Fertilizer Quality Control Laboratories

7.29 Funds amounting to Rs 44.39 crore have been released till 11th November, 2016 under Soil Health Management component. Comparison of funds released during the current year (till October) with that of previous years is shown below. (Rs. in crore):

Year	2012-13	2013-14	2014-15	2015-16	2016-17 till 15.11.16
Amount	8.51	19.24	63.98	44.39	60.31

7.30 Soil Health Card Scheme: Soil Health Card Scheme has been recently approved for implementation during 12th Plan period. The Scheme will provide farmers with information on soil analysis and recommendation on appropriate dosage of nutrients to be applied for cultivation. Soil analysis will be done in accordance with uniform sampling techniques and procedure to provide information to the farmers. Soil Health Card will be issued at 2 years intervals in respect of all the 14 crore land holdings in the country.

7.31 INM & Organic Farming: Ministry of Agriculture, Cooperation and Farmers Welfare, Department of Agriculture, Cooperation & Farmers Welfare is implementing INM & Organic Farming component under National Mission for Sustainable Agriculture. The financial assistance on various components as below is provided under the said components:

- Setting up of mechanized Fruit/ Vegetable market waste/Agro waste compost production units.
- Setting up of State of art liquid/carrier
- Setting up of Bio-fertiliser and Organic fertiliser testing laboratory or strengthening of existing laboratory under FCO.
- Promotion of Organic Inputs on farmer's fields.
- Support to research for development of organic package of practices specific to State and cropping system.
- Setting up of separate Organic Agriculture Research and Teaching Institute (against specific proposal).

Paramparagat Krishi Vikas Yojana (PKVY)

7.32 Paramparagat Krishi Vikas Yojana (PKVY) **is one of the schemes under** National

Mission for Sustainable Agriculture (NMSA) **to promote** certified organic cultivation in 2 lakh ha covering 10,000 clusters. Financial assistance of Rs 50,000 per ha per farmer is provided in 3 years**.**

7.33 Participatory Guarantee System (PGS) of certification is promoted in PKVY clusters. The financial assistance is provided to clusters on different sub components for mobilization of farmers, organic seeds, to harvest biological nitrogen etc. It includes different components such as:

I. Mobilization of farmers: training of farmers and exposure visit by farmers.

II. Quality control: soil sample analysis, process documentation, inspection of fields of cluster members, residue analysis, certification charges and administrative expenses for certification.

III. Conversion practices: transition from current practices to organic farming, which includes procurement of organic inputs, organic seeds and traditional organic input production units and biological nitrogen harvest planting etc.

IV. Integrated manure management: procurement of Liquid Bio fertilizer consortia/Bio pesticides, Neem cake, Phosphate Rich Organic Manure and Vermicompost.

V. Custom hiring centre charges: to hire agricultural implements as per SMAM guidelines.

VI. Labelling and Packaging assistance & Transport assistance.

VII. Marketing through organic fairs.

The funds released in 2015-16 is given in **Annexure 7.1**.

B. Participatory Guarantee System (PGS) – India

- An alternative to Third party certification needed for export market- cumbersome for farmers
- PGS India is cost effective, farmer-friendly and hassle-free – caters to domestic market
- Promotes decentralized and participatory approach
- Prepares farmers to maintain process and product documentation to win market credibility.

Mission Organic Value Chain Development for North Eastern Region (MOVCDNER)

7.34 Realizing the potential of organic farming in the North Eastern Region of the country, Ministry of Agriculture and Farmers Welfare has launched a **Central Sector Scheme** entitled **"Mission Organic Value Chain Development for North Eastern Region"** for implementation in the states of Arunachal Pradesh, Assam, Manipur, Meghalaya, Mizoram, Nagaland, Sikkim and Tripura, during 2015-16 to 2017-18. The scheme aims at development of certified organic production in a value chain mode to link growers with consumers and to support the development of entire value chain starting from inputs, seeds, certification, to the creation of facilities for collection, aggregation, processing, marketing and brand building initiative. The scheme was approved with an outlay of Rs. 400 crore for three years.

The assistance is provided for cluster development, on/off farm input production, supply of seeds/ planting materials, setting up of functional infrastructure, establishment of integrated processing unit, refrigerated transportation, pre-cooling /cold stores chamber, branding labeling and packaging,

hiring of space, hand holdings, organic certification through third party, mobilization of farmers/processors etc. Under this scheme, areas of 0.50 lakh ha have been targeted to be covered under organic farming in North Eastern Region of the country during 2015-16 to 2017-18.

Status of Scheme:

7.35 An amount of Rs. 158.87 crore was allocated to the north eastern states during 2015-16 andduring 2016-17 the allocation was made as Rs. 100.00 crore. During 2015-16 an amount of Rs. 112.11 crore has been released to the states. During current year 2016-17, total expenditure incurred is Rs. 31.22 crore out of which Rs. 31.02 crore have been released to the states.

Natural Resource Management

7.36 Agriculture growth can be sustained by promoting conservation and sustainable use of the natural resources through adoption of appropriate location specific measures. Conservation of natural resources in conjunction with development of rainfed agriculture holds the key to meet burgeoning demands for foodgrain requirement of increasing population of the country.

7.37 Land and Water are two important natural resources having implications on sustainable food production. Besides, frequent droughts, floods and climatic variabilities also impact soil fertility and cause land degradation, thereby, affecting food grain production across the country. As per available estimates of Indian Council of Agricultural Research (ICAR- 2010), out of total geographical area of 328.7 million hectare (m.ha), about 120.4 m.ha(37%) is affected by various kind of land degradation. This includes water and wind erosion (94.9 m.ha), water logging (0.9 m.ha), soil alkalinity/sodicity (3.7 m.ha), soil acidity (17.9 m.ha), soil salinity (2.7 m.ha) and mining and industrial waste (0.3 m.ha).

7.38 Natural Resource Management (NRM) Division is implementing mainly two Central Sector Schemes namely, Soil and Land Use Survey of India (SLUSI) and Soil Conservation Training Centre, Damodar Valley Corporation, Hazaribagh. The major mandates of Division are as under:-

- Technical input on soil & water conservation interventions for prevention of soil erosion & land degradation under various programmes/schemes of DAC&FW;
- Formulation of strategies, policies and programmes to address issues of degraded land, appropriate measures to combat adverse impact of such degradation on agricultural production;
- Reclamation & development of problem lands affected by alkalinity, salinity and acidity;
- Conducting various types of soil survey to provide scientific database on soil and land uses for planning & implementation of various land based interventions;
- Development of training and capacity of officials working in various States for soil and water conservation & management.

7.39 Schemes/Programmes: The details of the Schemes and Externally Aided Programmes being implemented by NRM Division are given in the succeeding paragraphs.

(A) Central Sector Schemes:

a) Central Sector Scheme of Soil and Land Use Survey of India (SLUSI):

7.40 Soil and Land Use Survey of India (SLUSI) governs and monitors all its activities from Head Quarter office situated at Indian Agricultural Research Institute

(IARI) campus, New Delhi. After its inception in 1958, it carried out various types of soil survey at different scales to provide scientific input to policy makers, planners and implementing agencies for various agricultural developmental programs. Watershed being the main hydrological entity for all soil and water conservation activities, SLUSI has generated microwatershed ATLAS of most of the States/Union Territories of the country. An advance technology of Remote Sensing, Geographical Information System (GIS) and Global Positioning System (GPS) are being used to generate site specific soil and land information by its seven regional centres located at Noida, Ranchi, Kolkata, Nagpur, Bengaluru, Hyderabad and Ahmedabad. It has also established a Remote Sensing Centre (RSC) for leveraging applications of advanced technologies in soil survey programmes. The principal activity is the characterization of soils, soil properties, soil suitability for agriculture and presenting the locational information with their spatial extent in map form within the suitable framework i.e. watershed of designated area/district etc. The generated information is instrumental in outlining the potential agricultural areas for the development, planning and management of that area. It also provides scientifically based technical advice on sustainable agricultural development and organises trainings for the officials state user departments on mapping & management of microwatersheds using **Space Technology** along with uses of database generated by SLUSI.

7.41 SLUSI has developed a methodology for prioritization of micro-watersheds in the catchment areas by conducting Rapid Reconnaissance Survey to facilitate planning of soil and water conservation in a selective mode and phased manner. The output of the survey is utilized both by Central and State Governments towards formulation of soil and water conservation working plans. It has generated database at Rapid Reconnaissance level for 224.76 million hectare in the country.

7.42 In view of launching of National Mission for Sustainable Agriculture (NMSA) by Department of Agriculture & Cooperation during 12th Five Year Plan, SLUSI has revised its mandate with special reference to Soil Health Management activities by generating detailed scientific database for planning and implementation of soil and water conservation activities and for sustainable landuse plan on micro-watershed basis on 1:10 K scale.

7.43 SLUSI is conducting Detailed Soil Survey (DSS) and generating database on soil resources through soil survey, profile study and soil analysis in GIS to facilitate adoption of location and soil specific crop management practices for sustainable landuse plan using real time **High Resolution Satellite Data**. The utility of data is enhanced by interpreting area for soil and land capability, irrigability and hydrological soil grouping etc. The database on land and soil characteristics through extensive field level scientific surveys is also helpful in Soil Health Management (SHM) through identification and mapping of degraded lands either due to Salinity/Alkalinity/ Acidity or soil erosion with their intensity by suggesting reclamation / management plan.

7.44 SLUSI also provides technical support to National Mission for Sustainable Agriculture (NMSA) by making available generated maps and reports essentially required for integrated watershed development planning, management and monitoring of natural resources to State Governments.

7.45 Training programs are organized by various centres of SLUSI in the states under their jurisdiction for capacity building and updating knowledge of officers of State Governments involved in different

developmental programs viz. Integrated Watershed Management Program (IWMP), agriculture, horticulture, forestry, soil & water conservations and rural development etc.

2. Development of MIS (Management Information System):

7.46 SLUSI has developed a GIS-based web services for "Detailed Soil Survey Information System" under G2G domain over its Website http://slusi.dacnet.nic.in. Users without GIS knowledge can also intuitively explore the data. GIS maps can visually enhance the spatial and temporal understanding of phenomena and improve the interpretation of soil-landscapes and ecosystems. The data uploaded would be highly useful in recommending location specific crops and soil & water conservation activities in the area.

3. Activities during 2016-17:

- **Digital Initiative:** On the line with the government's Digital initiative, SLUSI has brought a paradigm shift in all Technical & cartographic mapping services from manual drawing and visual interpretation to direct **'ON-SCREEN'** satellite data interpretation and digitization. It has saved the time, mandays and stationery materials required in conventional exercises. These mandays are being used to convert old hard copy maps and reports into digital format. All the centres of SLUSI have started working in this direction by initiating **'ON-SCREEN'** pre-field and post-field **interpretation of satellite data.**
- **Consultancy Projects:** District wise desertification status mapping of Pouri Garhwal & Chamoli districts of Uttrakhand and Hoshiyarpur & Pathankot districts of Punjab State has been carried out on 1:50,000 scale in collaboration with **Space Application Centre, Ahmedabad, Department of Space, Govt. of India.** Multi-temporal LISS-III satellite data was used for desertification change detection analysis. This spatial data was integrated with Soil resource, land capability and land use/land cover data in GIS to generate **"Desertification Vulnerability Index"** (DVI) for chalking out strategies to combat desertification.

  SLUSI has also taken up Soil Resource Mapping of Alppuzha, Kasaragod and Pathanamthitta districts of Kerala on 1:50,000 scale funded by **Kerala State Land Use board (KSLUB)** for Land capability and irrigability classification in these districts during 2016-17.
- **Generation of Digital Micro-watersheds with Unique Nomenclature:** SLUSI is engaged in development of digital version of Micro-watershed atlas of the country with unique code which will provide platform for the planners in designing location specific development plans under various agricultural and rural development programs in the country. The Micro-watershed atlas aims at identifying and recognizing each micro-watershed with distinct spatial extent and Unique National code in the country. This will also help in prevention of overlapping of planning and implementation of development activities by various agencies by adoption of web based transparent monitoring system. Micro-watershed Atlas of all states/Union Territories have been completed except Odisha. The Micro-watershed Atlas of Chattisgarh, Goa, Gujarat, Harayana, Karnataka, Kerala, Madhya Pradesh, Puducherry, Punjab, Sikkim, Tamil Nadu, Tripura and Uttrakhand States

have been uploaded on website and for other completed States is about to be uploaded. The digital spatial databases in respect of Micro-watershed Atlas of Odisha States would be completed during the current financial year.

- **Soil Surveys:** SLUSI has fixed the target to complete Detailed Soil Survey (DSS) of **10.00 lakh ha in Rainfed districts** of various states during 2016-17. The DSS of 2.85 lakh ha has been completed and survey of the rest of the area is under progress and will be completed within this financial year. Similarly, Soil Resource Mapping (SRM) of nine districts of Bihar having **23.60 lakh ha area** has been completed and survey is to be taken up shortly in **Alppuzha, Kasaragod and Pathanamthitta districts of Kerala** State after receiving the funds from Kerala State Land Use board (KSLUB) and would be completed during 2016-17.
- **Priority distribution and Land Capability Classification:** Priority distribution of the areas under various schemes was done and **'Very High Priority'** and **'High Priority'** areas were identified and demarcated in various catchments for soil and water conservation activities. Distribution of area on priority basis under various catchments are as under:
  - o **Soil Survey:** During 2015-16, DSS of 9.01 lakh ha and SRM of 10.34 lakh ha has been completed. Similarly against target of 10.0 lakh under DSS , an area of 6.08 lakh ha and under SRM 24.70 lakh ha against the target of 29.64 lakh ha., has been completed up to January, 2017.
  - o During 2016-17, an amount of Rs. 20.46 crore(RE) is allocated under Non-Plan against which Rs. 16.36 crore has been utilized for various activities upto January, 2017.
- **Short Courses/ Training Programs:** SLUSI has planned to organise **seven short courses/ trainings** during 2016-17 in different parts of the country for the officials of user departments for capacity building and updating their knowledge towards application of data base on soil and land use under various development programs viz. Integrated Watershed Management Program (IWMP), agriculture, horticulture, forestry, soil & water conservations and rural development. Out of seven trainings to be organized, four has been conducted till January, 2017 and rest of the trainings would be completed within this financial year.

b) Central Sector Scheme of Soil Conservation Training Centre-DVC, Hazaribag (Non Plan):

7.47 This Central Sector Scheme was created under Non-Plan for conducting training and capacity building including short orientation courses for soil & water conservation, land degradation, crop

(Area in lakh ha)

Projects	Surveyed Area	Very High Priority	High Priority	Total Priority
RVP	833.9	101.72	120.90	222.62
FPR	489.5	67.00	87.25	154.25
Non RVP/FPR	875.6	78.24	98.91	177.15
Consultancy	48.6	2.34	3.27	5.61
**Total**	**2247.6**	**249.3**	**310.3**	**559.63**

RVP- River Valley Project

FPR- Flood Prone River

management, livelihood support through off farm activities, agro forestry, Integrated Farming System, Soil Health Management and Climate change adaptation and mitigation in agriculture and allied sectors which are essential for strengthening capabilities of field functionaries. During 2016-17, 11 training courses have been planned with an estimated cost of Rs. 50 lakh, out of which 9 training courses have been completed and 169 numbers of officials have been trained with expenditure of Rs 40.00 lakh till January, 2017.

Other NRM Related Interventions:

a) Watershed Development Fund (WDF):

7.48 The Union Government has established a Watershed Development Fund WDF during 2000 with a corpus of Rs.200 crore which includes Rs.100 crore by DAC and Rs.100 crore by National Bank for Agriculture and Rural Development (NABARD) as matching contribution. The total corpus and its management is vested with NABARD. Objective of WDF is to promote participatory watershed development involving Watershed Community, State Government Departments, Banks and NGOs. Presently, WDF scheme is being implemented in Gujarat, Maharashtra, Uttar Pradesh, Uttarakhand, Karnataka, Tamil Nadu, Rajasthan, Chhattisgarh, Odisha, Jharkhand, Madhya Pradesh, Himachal Pradesh and West Bengal. During 2006, after announcement of Prime Minister's Rehabilitation Package in 31 distressed districts in States of Andhra Pradesh, Karnataka, Kerala and Maharashtra, it was decided to implement participatory watershed development programme in all these distressed districts through WDF. As per information reported by the NABARD, Mumbai, **an amount of Rs 241.98 crore (till March, 2015),** had been released for the development of 506 Watershed projects under non distressed districts of 16 States. In case of Prime Minister's Rehabilitation Package, **an amount of Rs. 791.29 crore (up to October, 2015)** had also been released to develop 764 Watershed projects under distressed districts of 4 States.

b) Development of Model Watersheds through ICRISAT, Hyderabad & CSWCRTI, Dehradun:

7.49 To address bio-physical and socio-economic dimensions of specific agro climatic conditions and to develop suitable technologies for maximizing development process under watershed programmes, 18 Model Watershed Projects covering different agro ecological regions of the country have been assigned to Central Soil & Water Conservation Research and Training Institute (CSWCRTI), Dehradun (9 projects) and International Crop Research Institute for Semi Arid Tropics (ICRISAT), Hyderabad (9 projects). These watersheds would serve as model for replicating successful technologies in other National/ State watershed projects. Rs 3.97 crore and Rs **9.25 crore** have so far been released to CSWCRTI and ICRISAT respectively and summary of the recommendation have been circulated to concerned Department for adoption.

Externally Aided Projects:

(A) World Bank assisted Projects:

7.50 World Bank assisted Projects in the states of Himachal Pradesh, Assam, Rajasthan and Uttar Pradesh are being implemented. DAC&FW is involved in supervision, coordination and monitoring of these projects. World Bank provides assistance for these projects directly to the State Governments. The details of the projects are given below:

a) Himachal Pradesh Mid-Himalayan Watershed Development Project:

The Himachal Pradesh Mid-Himalayan Watershed Development Project (Credit No. 4,133) became operational in February, 2006. This project aims at preventing degradation and protection of bio-diversity, improving accessibility to rural areas and productivity of livestock etc. and envisages institutional strengthening, watershed development and management, enhancing livelihood opportunities, project management and coordination. The cost of this project is about Rs.510.00 crore and area to be covered includes 602 Gram Panchayats in 10 districts, namely, Sirmour, Solan, Bilaspur, Shimla, Kullu, Mandi, Hamirpur, Kangra, Chamba and Una. **The work is in progress in all selected Gram Panchayats and a total of Rs. 105 crore have been spent during 2015- 2016. During 2016-17, Rs. 8 crore has been utilized till January, 2017.**

b) Rajasthan Agricultural Competitiveness Project:

Rajasthan Agricultural Competitiveness Project (Credit No 5085) became operational in April 2013 and will close in March 2020. The objective of the project is to demonstrate at scale the feasibility of a range of distinct agricultural development approaches integrating technology, organization, institution and market innovations across selected regions of Rajasthan, each marked by different agro-ecological, climatic, water resource and social conditions. The total project cost is Rs 832.5 crore. Total 20 clusters are targeted to be developed in selected locations. So far 12 clusters viz. Mokhampura in Jaipur, Ladnu in Nagaur and Bansur in Alwar, Pisangan in Ajmer, Gudha in Bundi and Z-minor in Sri Ganganagar, Bonli in Sawai Madhopur, Sangod in Kota, Phoolasar in Bikaner, Kheruwala in Jaisalmer, Orai & Bassi in Chittorgarh and Jakham in Pratapgarh have been selected. **Under this project, Rs. 19.278 crores** have been incurred for preparatory works upto January, 2017.

c) Sodic Land Reclamation & Development Project with World Bank Assistance:

Project proposal on "Uttar Pradesh Land Reclamation and Development Project" at an estimated cost of Rs.2,000 crore for reclamation and development of 3.10 lakh ha area was proposed for seeking financial assistance from World Bank. Technical and Financial agreement was signed in June, 2009 for development of 1.35 lakh ha of degraded land comprising 1.30 lakh ha of Sodic lands and 5,000 ha of Ravine lands at an estimated cost of Rs 1,224 crore in 6 years. **During the year 2015-16, an amount of Rs 165.68 crores have been utilized and during 2016-17 (up to 30th September 2016), Rs. 49.10 have been utilized for the project making the total expenditure on the project to Rs 1000.84 crores.**

(B) Crop Diversification in Himachal Pradesh with Japan International Cooperation Agency (JICA):

7.51 Project for implementation of various interventions for Crop Diversification in Himachal Pradesh with JICA assistance at an estimated cost of Rs.321 crore for 7 years was signed on 1st October, 2010. After detailed deliberations and visit of experts to identified districts of Himachal Pradesh, Overseas Development Assistance (ODA) agreement was signed on 17th February, 2011 and agreed interventions are being undertaken in 5 selected districts namely; Kangra, Hamirpur, Bilaspur, Mandi and Una. **Up to January, 2017, an amount of Rs 161.52 crore has been utilized for various approved interventions.**

National Rainfed Area Authority (NRAA)

7.52 National Rainfed Area Authority (NRAA) was established as an attached office of DAC &FW on 3rd November, 2006. As per approval of Cabinet, NRAA has been again placed under Department of Agriculture & Farmers Welfare (DAC&FW) with effect from 21st March 2015.

7.53 NRAA is an 'Advisory Body' for policy and programme formulation and monitoring of programmes relating to integrated development of degraded/rainfed areas. Cabinet Committee of Economics Affairs (CCEA) also approved on 1st July, 2015 the involvement of NRAA in providing technical inputs in planning and implementation of PMKSY in the Rainfed areas for rain water conservation, watershed development and its management.

7.54 Further, all six long pending studies relating to Watershed, Water management & Animal Husbandry etc. have been pursued and completed. Final reports of these project/studies have been accepted and summary recommendations of all projects have been uploaded on NRAA website (www.nraa.gov.in) for all viewers. These studies are:

a. Preparation of State Specific Technology Manual for Watershed Development in Rajasthan by Maharana Pratap University of Agriculture & Technology, Udaipur (Rajasthan) through Directorate of Watershed Development & Soil Conservation, Government of Rajasthan.

b. Preparation of State Specific Manual for Watershed Development by Tamil Nadu Watershed Development Agency (TAWDEVA, SLNA, Chennai through NABARD Consultancy Services Pvt. Ltd., Chennai.

c. Pilot Study on Capitalizing Opportunities of Rice Fallow for Sustainable Livelihood Development in the State of Jharkhand by Directorate of Soil Conservation, Government of Jharkhand through Birsa Agriculture University, Ranchi.

d. Pilot Study on Capitalizing Opportunities of Rice Fallow for Sustainable Livelihood Development in the State of Chhattisgarh by State Agriculture Management and Extension Training Institute (SAMETI), Government of Chhattisgarh, Raipur through Indira Gandhi Krishi Vishwavidyalaya, Raipur.

e. Inter institutional, Livestock Centric Intervention for livelihood improvement in arid regions in Nagore Districts of Rajasthan (Consortia mode through M/s Rajasthan Livestock Development Board Jaipur, Central Arid Zone Research Institute, Jodhpur and Gramin Vikas Trust, Noida).

f. Agro-forestry Study on Identification of extent of forest land based on the qualitative and quantitative assessment of the fringe forest lands and their productive status in 275 districts of the country by Forest Research Institute (ICFRE), Dehradun(to be uploaded by March, 2017).

7.55 NRAA has provided financial support of Rs. 1.16 crore to National Institute of Rural Development and Panchayti Raj, Ministry of Rural Development (NIRD & PR) for training of IAS/IFS officers for formulation of District Irrigation Plan (DIP) of PMKSY. A total of eight training programmes have been organized and 203 officers of different states have been trained.

7.56 During 2016-17, against allocation of Rs 1.99 crore (Revised Estimate), an amount of Rs. 1.35 crore has been utilized for various mandated activities up to January, 2017.

Reclamation of Problem Soils (RPS) as Sub Scheme of RKVY:-

7.57 As per available estimates (ICAR-2010), the area under problem soils is about 243 lakh hectare., comprising alkali soil(37.00lakh ha.), saline soil(27.3 lakh ha.) and acid soil (179.3 lakh ha.). Accordingly, with a view to sustain soil fertility and productivity of such problem soil Department of Agriculture, Cooperation & Farmers Welfare (DAC&FW) has launched **"Reclamation of Problem Soils (Alkali/Saline & Acid)"** as a sub Scheme of Rashtriya Krishi Vikash Yojana (RKVY) on Pilot basis in 15 States having higher extent of problem soils with central share of Rs 50.00 crore and an amount of Rs. 15.68 crore has been utilized upto January, 2017.

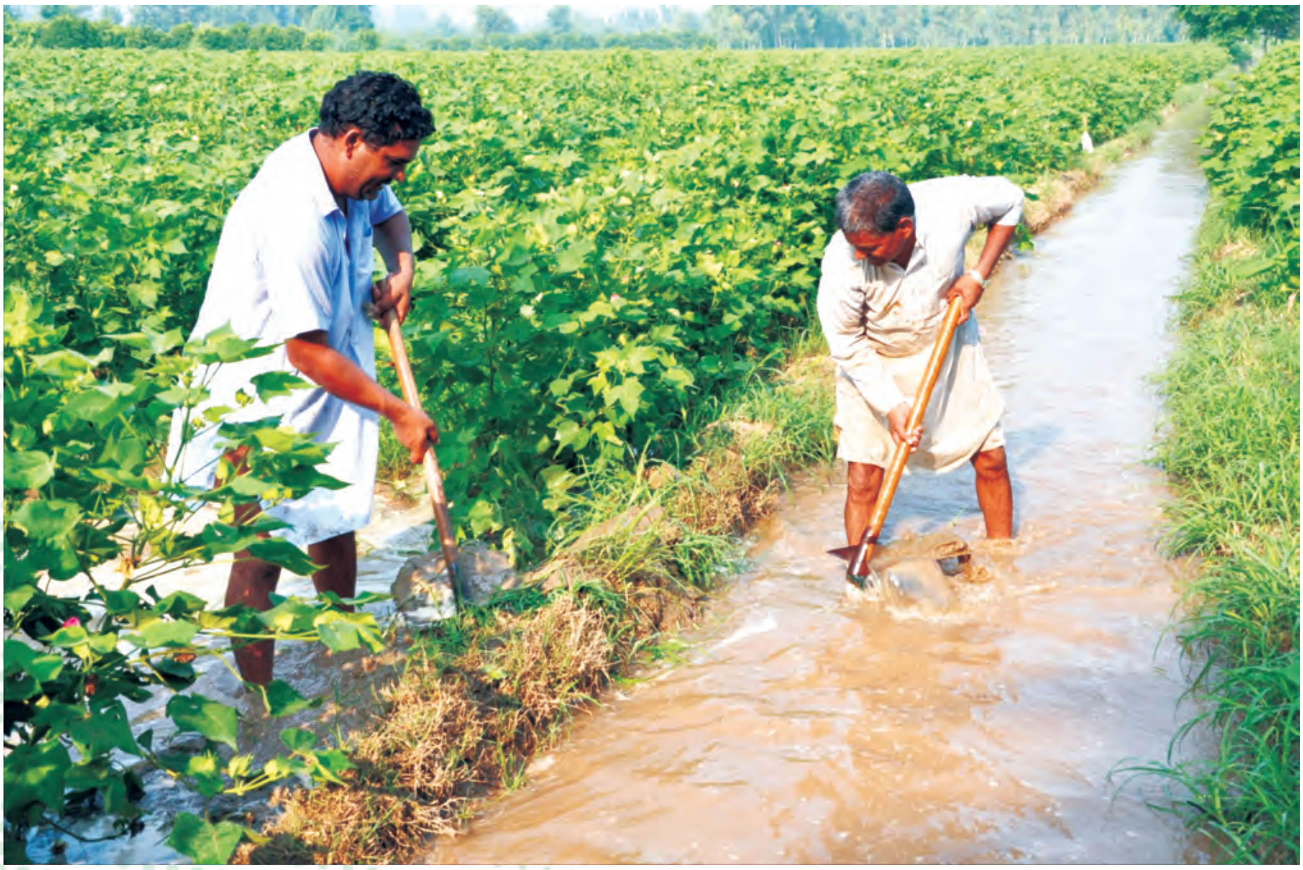

******

Chapter 8

National Mission on Agricultural Extension & Technology (NMAET)

8.1 The Sub Mission on Agricultural Extension (SMAE) under the National Mission on Agricultural Extension and Technology (NMAET) being implemented during the 12th Plan with an objective to restructure and strengthen the agricultural extension machinery with a judicious mix of extensive physical outreach of personnel, enhancement in quality through domain experts & regular capacity building, interactive methods of information dissemination, Public Private Partnership, pervasive & innovative use of Information & Communication Technology (ICT) / Mass Media, Federation of groups and convergence of extension related efforts under various schemes and programmes of Government of India and the State Governments. The SMAE aims to appropriately strengthen, expand and upscale existing Extension & Information Technology (IT) Schemes. The ongoing Extension Schemes include the Central Sector and Centrally Sponsored Schemes being implemented by the Extension Division/Directorate of Extension. Even in the case of Central Sector Schemes which have been subsumed within the Mission, a greater role has been envisaged for the States through their active involvement in planning, implementation and monitoring.

I. Support To State Extension Programmes For Extension Reforms Scheme (ATMA Scheme)

8.2 The erstwhile Scheme **'Support to State Extension Programmes for Extension Reforms (ATMA)'** implemented since 2005 has now been included as a component of the Sub-Mission on Agriculture Extension (SMAE) under NMAET with some cost revisions and additional components. It is now under implementation in **652 districts of 29 states & 3 UTs** of the country. The scheme promotes decentralized farmer-driven and farmer accountable extension system through an institutional arrangement for technology dissemination in the form of an Agricultural Technology Management Agency (ATMA) at district level. Under the scheme grants-in-aid is released to states with an objective to support State Governments efforts of revitalization of the extension system and making available the latest agricultural technologies in different thematic areas to increase agricultural production through extension activities viz. Farmers Training, Demonstrations, Exposure Visits, Kisan Mela, Mobilization of Farmers Groups and Setting up of Farm Schools. Through these activities, latest agriculture technologies are disseminated to farmers of the country.

1. In order to promote key reforms under the scheme, ATMA Cafeteria 2014 continues to support activities in line with the following policy parameters:

- **Multi-agency extension strategies: Atleast 10%** of allocation on recurring activities at district level is to be used through non-governmental sector viz. NGOs, Farmers' Organization (FOs), Panchayati Raj Institutions (PRIs), para-extension workers, agri-preneurs, input suppliers, corporate sector, etc.
- **Farming system approach:** The

activities specified in the cafeteria are broad enough to promote extension delivery consistent with farming systems approach and extension needs emerging through Strategic Research and Extension Plan (SREP).

- **Farmer centric extension services:** The cafeteria provides for group-based extension and it has necessary allocation for activities related to organizing and supporting farmer groups. In order to supplement these efforts, a provision for rewards and incentives to the best organized farmer groups has also been provided.
- **Convergence:** The Strategic Research and Extension Plan (SREP) and State Extension Work Plans (SEWP) would also be mechanisms for ensuring convergence of all activities for extension. At present, resources for extension activities are being provided under different schemes of Centre/ State Governments. It is mandated that the SEWP, submitted by the State Governments for funding under the scheme shall explicitly specify the activities to be supported from within the resources of other ongoing schemes as well as from this scheme.
- **Mainstreaming gender concerns:** It is mandated that **atleast 30%** of resources on programmes and activities are utilized for women farmers and women extension functionaries.

2. The 'important features' of the 'Cafeteria of Activities' supported under Modified ATMA Scheme are as follows:-

- Support for specialists and functionaries at State, District and Block Level
- Innovative support through a 'Farmer Friend' at Village Level @ 1 Farmer Friend per two villages
- Farmers Advisory Committees at State, District and Block levels
- Farm Schools in the field of outstanding farmers being promoted at Block/ Gram Panchayat level by integrating the Progressive farmers into Agricultural Extension System (AES) (3-5 Farm Schools/ block).
- Farmer-to-farmer extension support at the village level to be promoted through Farmers' Group.
- Funding window provided at both State and District levels for implementing innovative extension activities are not specifically covered under the Programme.
- Farmers' Awards instituted at Block, District and State levels.
- Community Radio Stations (CRS) to be set up by KVKs being promoted under the Programme.
- For Non-Governmental implementing agencies, States have been given the flexibility of having State Extension Work Plans prepared and approved at the State level. Atleast 10 per cent of outlay of the Programme is to be utilized through involvement of non-governmental sector. Non-governmental implementing agencies (excluding the corporate sector) are also eligible for service charge up to a maximum of 10 percent of the cost of the extension activities implemented through them. Apart from other NGOs financial assistance is also available for implementation of extension activities through agri-preneurs.
- Re-designating the Subject Matter Specialists (SMSs) as Assistant Technology Managers (ATMs).
- Increasing number of ATMs from existing two to average three ATMs per Block.
- Public extension functionaries are being made more effective through trainings and exposure visits. (MANAGE, Hyderabad offering PG Diploma in

Agriculture Extension Management for public extension functionaries which is fully funded under the ATMA Programme).

- Use of interactive and innovative methods of information dissemination like pico-projectors, low cost films, hand held devices and mobile based service, etc.

3. Implementation Status

- Total 652 ATMAs have been constituted in 29 States and 3 UTs.
- Institutional arrangements viz. Inter Departmental Working Group (IDWG) in 29 States and 3 UTs, ATMA Core Committees – Governing Board (GB) & ATMA Managing Committee in 652 Districts; Block Technology Team (BTT) in 5990 Blocks & Block Farmer Advisory Committees (BFACs) reconstituted in 5544 Blocks; District FACs have been constituted in 561 districts and State Level FACs in 19 States.
- SEWPs of 31 States/ UTs have been prepared and approved based on District Agriculture Action Plans of 651 ATMA districts. As against the B.E. of Rs.450.00 crores, an amount of **Rs. 287.64 crores has been released** to the States up to 2nd December, 2016 for implementation of the Scheme and total release since the inception of the scheme in 2005-06 to 2nd December, 2016 has been to the tune of **Rs. 3499.75 crore.**
- Physical performance of the Scheme since its inception in 2005-06 (April 2005 to September 2016) is as below:
    - o Over 36231269 farmers including 9637720 farm women (26.60%) have participated in farmer oriented activities like Exposure Visits, Trainings, Demonstrations, Farm Schools & Kisan Melas.
    - o Over 215964 Commodity based Farmer Interest Group (CIGs)/ FIGs have so far been mobilized under the scheme.
    - o Over 97803 Farm Schools have been organized on the fields of outstanding farmers.
- Progress of implementation during current financial year (up to September, 2016):
    - o Over 12 lakh farmers including 6 lakh farm women (50.00%) have been reportedly participated in farmer oriented activities like exposure visits, trainings, demonstrations & kisan melas. The data received from Maharashtra is however being confirmed from State Government as they have reflected huge number of beneficiaries under Demonstration activity.
    - o 14045 CIGs/ FIGs organized.
    - o 4566 Farm Schools organized.
    - o 12906 specialists & functionaries have been reported as deployed under ATMA as on 31st August, 2016.

II. "Mass Media Support To Agricultural Extensioin"

8.3 This scheme is utilizing countrywide infrastructure and networks of All India Radio and Doordarshan and focusing dissemination of latest farm practices through Radio and Television networks. The Prasar Bharati, a 'National Public Service Broadcaster' is implementing this scheme. The objective of the scheme is to enhance and boost the Agriculture Extension system in the present scenario. At present the farmers need technology, investment, better quality inputs, real time information and most of all the latest know-how for sustaining commercial and cost effective

sustainable agriculture. A major shift in the methodology of delivering knowledge to the farm has taken place. Radio and TV have the advantage of reaching a wide audience at a very low cost.

1. Telecast of Krishi Darshan Programmes on Doordarshan:-

8.4 Under this scheme, a 30 minute programme is being telecast 5-6 days a week through 01 National, 18 Regional Kendras and 180 High Power/Low Power Transmitters of Doordarshan. For telecasting success stories, innovations and for popularization change-setting technology and farming practices through the Saturday slot of Doordarshan's National Channel, DAC&FW is producing films, which would consciously project inter-alia positive aspects in agriculture and allied sector in India. During the period, 14 films (4 films on various horticulture practices and 10 success stories of the farmers) have been produced. With the view to disseminate service oriented real time information to the farming community, Dooradrashan is telecasting regular Krishi Darshan Programme on 1 Nation Channel in Hindi language, 18 state level regional channels in respective regional languages five days a week and 36 Narrowcasting Production Kendras producing two programmes a week and the same are being telecast through 180 High Power and Low Power Relay Transmitters. Keeping in view the importance of real time farm advisories, weather reports, early warning information, agriculture news and agriculture market trends, all Kendra of DD and AIR station are being telecasting/ broadcasting regular programmes on these subjects under respective segment of the programme.

2. Broadcast of Kisan Vani Programme on All India Radio:

8.5 Under this component, 96 FM/AM stations of All India Radio are broadcasting 30-minute programme six days a week from 6.30-7.00 PM. Each station broadcast separate programme in respective dialects/ languages. With easy availability of FM Radio on mobile phones, FM Stations can have wider outreach than before. However, depending on cost-benefit ratio, it will be endeavour of the Department to go in for alternative technologies such as Amplitude Modulations (AM), Digital Radio etc.

3. Telecast/ Broadcast of spots/ jingles advisories under 'Free Commercial Time (FCT)' on AIR and DD:

8.6 In addition to above regular programmes, the Free Commercial Time (FCT) available under Krishi Darshan and Kisanvani programme is being utilized for dissemination of Advisories on Rabi / Kharif season, Jingles on Cooperatives spots on Kisan Call Centers, Judicious use of fertilizers, safe use of Pesticides, Machinery and Technology, Farm School, National Food Security Mission (NFSM), Kisan Credit Card and Agri -Clinic and Agri -Business Centers (ACABC), package of practices available to the farmers under National Food Security Mission (NFSM), and other important flagship programmes like Neem Coated urea, Pradhan Mantri Krishi Sichai Yajana, Crop Insurance Scheme, BGREI , National Agriculture Market, Siol health Card, Bee Keeping, NHM, Parampagat Krishi Vikas Yojana and Organic Farming etc and , contingency plan developed by State Governments and emergent issues like Drought, Flood etc.

4. Focused Publicity & Awareness Campaign through other media platforms:

8.7 Besides above, the **'Focused Publicity & Awareness Campaign** which would cut across all the Divisions of the Ministry was launched on July 5, 2010 to create awareness

about the assistance available under various schemes of the Department of Agriculture, Cooperation & Farmers Welfare. This campaign will be continued during the 12th Plan, also in an aesthetic, professional and politically neutral manner. Video Spots and Audio spots are being broadcast/telecast through AIR, DD, Lok Sabha TV and also on Private TV Channels. The above spots are being broadcast/telecast through AIR/ DD and private national and regional TV channels during news and entertainment programmes. In addition to this, Ministry is using various multimedia platforms i.e. railway panels/ stations, bus panels, exhibitions through Directorate of Filed Publicity, web based digital platforms, hoardings etc. for massive media campaign on above flagship programmes.

5. Technical Support to DD Kisan Channel:

8.8 Recently launched DD Kisan, a 24 hour agriculture based channel is catering the requirements of the farming community inclusive of research updates, extension advisories, market rates and weather updates and is also utilized extensively under the MMSAE for dissemination of information.

6. Support to Community Radio Stations (CRS):-

8.9 To promote agriculture extension through mass media at community level, the Ministry of Agriculture is also providing support for setting up of Community Radio Stations (CRS), which would make a major contribution to agricultural extension by utilizing the reach of radio transmitter and disseminating information and knowledge, produced locally and having relevance for a specific area in local dialects/languages. As on date, 8 CRs are operational in KVKs and NGOs under this scheme and broadcasting agriculture programmes.

7. Print Media:

8.10 Curiosity of the stake-holders (particularly farmers) is expected to get whetted by the video and audio spots. More detailed inputs – preferably information having region specificity – will continue to be given through print advertisements and write-ups in regional languages. The national and regional newspapers are being utilized based on their circulation figures.

8. Mass Media Scheme in NE Region:

8.11 The programmes under the scheme are being disseminated throughout the country including NE region benefiting the farmers of the area. In the NE Region the programmes are being disseminated in regional languages & local dialects through 1 Regional Kendra at Guwahati, 6 NC Programme Producing Kendras and 22 HPT/LPT along with 8 FM radio stations. The Regional and NC Kendras are producing programmes as per area based requirement and local dialects.

8 Financial Provisions:-

8.12 During the year 2016-17, the amount of Rs 131.18 Cores has been earmarked under Central Sector Scheme 'Mass Media Support to Agriculture Extension' for Krishi Darshan and Kisan Vani Programmes being implemented through Prasar Bharati including 'Focused Publicity and Awareness Campaign' through Radio/ TV /Print and other outdoor media platforms.

III. Establishment of the Agri-Clinics and Agri-Business Centres (ACABC)

8.13 The ACABC scheme is under implementation since April, 2002. The scheme aims at creating gainful self-employment opportunities to unemployed agricultural graduates, agricultural diploma holders, and intermediate in agriculture apart from science graduates with post-graduation in

agriculture related courses for supporting agriculture development and supplementing the efforts of public extension.

8.14 MANAGE is the implementing agency for training component under the scheme through a network of identified Nodal Training Institutes (NTIs) in various states. **NABARD** is implementing subsidy component under the scheme on the behalf of Government of India and is monitoring credit support to Agri-Clinics through Commercial Banks.

8.15 The credit linked back-ended subsidy @ 36% of the Total Financial Outlay (TFO) capital cost of the project funded through bank loan is available under the scheme. This subsidy is 44% in respect of candidates belonging to SC/ST, Women and all categories of candidates from North-Eastern and Hill States.

8.**16** So far, 51413 candidates have been trained and 21077 agri-ventures have been established in the country during the period of implementation of the scheme (since inception till December, 2016). During the year 2016-17, starting from April, 3597 candidates have been trained and 640 agri-ventures have been established till November, 2016.

8.**17** The ACABC scheme has been revised during 2010-11 with changes in relevant operational aspects, aiming to provide better services to farmers, improvements in the quality of training and simplify the process of subsidy disbursement. Provision of extension service to farmers by these agri-preneurs has been made a mandatory component of the scheme. Details of the scheme may be seen at www.agriclinics.net.

IV. Pre-Seasonal DAC-ICAR Interfaces

8.18 Pre-seasonal DAC&FW-ICAR Interfaces (Subject Matter Group Meetings between ICAR and Subject Matter Divisions of DAC&FW) are organized to evolve joint strategies on the emerging issues of agriculture and allied sector. The recommended action by these group meetings are further discussed during an interface meeting with active participation of officers from DAC&FW, ICAR and Department of Animal Husbandry Dairying and Fisheries (AHD&F). The Pre-Kharif 2016 DAC&FW-ICAR Interface was organized during March, 2016 and the group recommendations of the said interface were shared with senior officer of State Agriculture Department during National Conference on Agriculture for Kharif Campaign held during April, 2016. The Pre-Rabi 2016-17 DAC&FW Interface was not organized due to some administrative inconveniences.

V. Kisan Call Centers (KCC):

8.19 The KCC Scheme was launched on 21st January 2004 to provide answer to farmers' queries on agriculture and allied sectors through toll free telephone lines. A country wide common eleven digit number **'1800-180-1551'** has been allocated for KCC. The replies to the queries of the farming community are being given in 22 local languages. KCCs operate from 14 locations in the country covering all the Sates and UTs. Calls are attended from 6.00 am to 10.00 pm on all 7 days of a week. Since inception of the scheme till 30th November, 2016 over 300.63 lakh calls have been registered in the KCCs. During the current year around 44 lakh calls have been received upto 30th November, 2016. In order to make farmers aware of this facility, audio and video spots on Kisan Call Centres have been broadcast/telecast through All India Radio, Doordarshan and private television channels. A Kisan Knowledge Management system (KKMS) has been created at the backend to capture details of the farmers calling KCCs. Modified call Escalation Matrix has also been put in place. If the queries are not answered by FTA is escalated

to concerned Block Level Officer for replying the query through KKMS interface within given time frame.

8.20 The Kisan Call Centre scheme has been recently restructured and strengthened, with a good number of unique features viz. provision of IPPBX, 100% call recording; call barging; voice mail service; customized IVRS; call conferencing through the experts; playing state specific advisories during call wait time and SMS to caller farmers giving a gist of answers given by the KCC Agent now known as Farm Tele Advisor. The farmer calling KCC can also register for receiving SMSs from experts on the subject area and their providing and also for receiving regular updates on mandi price of selected mandis and crops.

VI. HRD Support

8.21 DAC has strengthened a network of training institutions in the country by supporting the National Institute of Agricultural Extension Management (MANAGE) at Hyderabad; four Regional Extension Education Institutes (EEIs) at the regional level and the State Agricultural Management & Extension Training Institutes (SAMETIs) at the State level.

1. MANAGE:

8.22 MANAGE is providing training support to senior and middle level functionaries of the State Governments. It is also providing necessary support to the states in implementation of Extension Reforms (ATMA) scheme. Against 190 training courses planned for 2016-17, 96 training courses have been organized by MANAGE up to October, 2016. Against the budget estimate of Rs.1260.00 lakh, an amount of **Rs.630.00 lakh has been released** till November, 2016.

The implementation of self-financing professional courses viz. two-year Post Graduate Program in Agri-Business Management, one-year 'Post Graduate Diploma in Agricultural Extension Management' in distance learning mode and one year Diploma in Agricultural Extension Services for Input Dealers (DAESI) by MANAGE (organized on weekends/weekly holidays) are being continued during the year 2016-17.

2. Extension Education Institutes (EEIs):

8.23 Four Extension Education Institutes namely, Nilokeri (Haryana), Rajendranagar, Hyderabad (Telangana), Anand (Gujarat), Jorhat (Assam) are functioning at the Regional Level. The objectives of EEIs are to improve the skills and professional competence of middle level Extension field **functionaries of Agriculture and Allied Departments of the State Governments in the areas of (a) Extension Education; (b) Extension Methodology; (c) Information and C-ommunication Technology; (d) Training Methodology; (e) Communication; (f) Market led Extension; etc.**

8.24 During 2016-17, a total of 197 training programmes have been approved by the Directorate of Extension, consisting of 96 On-Campus and 101 Off-Campus trainings. Against this, 128 training programmes with 3002 participants (67 on campus and 48 off campus) have already been conducted (till December, 2016). An amount of Rs. 549.09 lakh has been released to the EEIs till December, 2016.

3. Model Training Courses (MTCs):

8.25 Model Training Courses of eight days duration on thrust areas of Agriculture, Horticulture, Animal Husbandry, Fisheries Extension are supported by the Directorate of Extension (DOE) with the objective of improving the professional competence and upgrading the knowledge and developing

technical skills of Subject Matter Specialists/ Extension workers of Agriculture and allied departments of the State Governments. During the current, financial year 60 Model Training Courses have been planned, out of which 39 training courses have been organized till December, 2016. Against the budget grant of Rs. 177.00 lakh an amount of Rs. 92.00 lakh has been released till December, 2016.

4. Diploma in Agricultural Extension Services for Input Dealers (DAESI):

8.26 DAESI is of one year (expanded to 48 weeks) regular course launched in 2003 with an objective to impart education in agriculture and other allied areas to the Input Dealers so that they can establish linkage to their business with extension services, besides discharging regulatory responsibilities enjoined on them.

8.27 This programme was earlier implemented through MANAGE @Rs.20,000 per candidate in self-financial mode in Andhra Pradesh, Maharashtra, Tamil Nadu, Odisha, Jharkhand & West Bengal, and so far 4107 candidates have been trained till March, 2016. It has now been decided to implement DAESI programme across the country through SAMETIs with involving Agribusiness Companies, ATMAs, KVKs, Agril. Collages & NGOs. During the current year 2016-17, 62 programmes are under process which will be completed by March, 2017. As on 31.12.2016, 1095 candidates have been trained during the current year.

VII. National Gender Resource Centre in Agriculture (NGRCA)

8.28 NGRCA has been set up as a unit of Directorate of Extension (DOE) of the DAC&FW under the Scheme of Extension Support to Central Institutes/DOE. NGRCA reflects the national commitment of empowerment of women through 'strategy of mainstreaming and agenda setting'. The Centre acts as a focal point for the convergence of gender related activities and issues in agriculture and allied sectors within and outside DAC&FW; addressing gender dimension to agriculture policies and programmes; rendering advocacy/ advisory services to the States /UTs to internalize gender specific interventions for bringing the farm women in the mainstream of agriculture development.

8.29 Being a Nodal Agency for women in agriculture in DAC&FW, Ministry of Agriculture, the Centre is actively involved in the revision of guidelines of various Beneficiary oriented scheme/programmes of DAC&FW to ensure allocation of resources and flow of benefits to the women farmers in proportion to their participation in agricultural activities. The following activities have been taken up during the year.

1. The following Macro/ Micro level and Action Research Studies have been initiated by the Centre.

- Study on "Adoption of Gender Friendly Tools by Women Farmers and its impact on their lives" – selection of the agency has been completed.
- Study on "Schemes for Improving Women Farmers Access to Extension Services and Gender Mainstreaming in Agriculture'' – Selection of the agency has been completed.

2. Publications brought out by NGRCA are as follow:

- Compendium on Gender Friendly Tools/Equipments.
- Farm Women Friendly Handbook (both in English & Hindi)

3. Revision of guidelines of various Beneficiary Oriented Schemes/

Programmes /Missions of DAC&FW to ensure allocation of resources and flow of benefits to the women farmer in proportion to their participation in agricultural activities.

4. Preparation of separate chapter on 'Gender Perspective in Agriculture' for the Annual Report and Gender Related Write-up for the Out Come Budget of DAC&FW, Ministry of Agriculture & Farmers Welfare.

5. Collation of special provisions and package of assistance available for women farmers under various on-going Missions/ Sub-Missions/ Schemes of DAC&FW, Ministry of Agriculture & Famers Welfare and even those of other Ministries/Departments.

6. Joint circular for Convergence of National Rural Livelihoods Mission (NRLM), MORD and the Schemes / Programmes /Missions of Department of Agriculture, Cooperation and Farmers Welfare, MoA&FW issued to the States/UTs.

VIII. Monitoring And Evaluation:

8.30 Support to State Extension Programme for Extension Reforms launched has a provision of third party monitoring termed as Concurrent Evaluation at State level. The Extension Division had also conducted the centralized evaluation of the scheme through an independent agency, Agriculture Finance Corporation (AFC). Important suggestions and issues for further improvements in ATMA schemes have already been dully addressed in the modified ATMA guidelines.

IX. Exhibitions

8.31 Agricultural exhibitions / Fairs are an effective means of reaching to a large number of farmers. The Department has sponsored / organized about 08 Kisan Melas in different parts of the country including participation in India International Trade Fair 2016 by setting up Agriculture Pavilion from 14th - 27th November, 2016 at Pragati Maidan, New Delhi, to disseminate relevant information and promote appropriate technologies and improved agronomic practices among the farmers and other stakeholders in agriculture and allied sectors. Financial assistance is provided for organization of Regional Agriculture Fairs (RAFs) to State Agriculture Universities and Indian Council of Agricultural Research (ICAR) institutes. Five (5) Regional Agriculture Fairs @ one in each region, namely, North, South, East, West and North East organised through State Agriculture Universities/ ICAR Institutes during a year are supported with a maximum funding of Rs.15 lakh each. The Ministry of Agriculture and Farmers' Welfare also provides financial assistance to other institutions for organization of farmer-centric International/ National/ State level events.

8.32 Various steps like direct participation of farmers, Farmer-Scientist interactions in regional languages, demonstrating appropriate technologies directly and by way of organizing crop/ livestock demonstrations, web-casting of Agriculture Pavilion during IITF 2016 are being done to insure that benefit of melas /agri shows reaches more no. of farmers. Suitable infotainment shows like Skit shows with some technical message are also included a part of the fair. Pre-event publicity through press releases, AIR, DD, KIsan Call Centre, Kisan Vani programme through FM Radio Station are also done during the year. DAC has participated in following events during the the year 2016-17

Sl. No	Fairs/Exhibitions/Events	State	Exp. Incurred (Rs.)
1.	Participation in *"Gram Uday se Bharat Uday Abhiyan"* at Jamshedpur from 23-24 April, 2016	Jharkhand	10,78,541
2.	Participation in DoNER Day Celebration at Shillong from 27-28 May, 2016	Meghalaya	60,000
3.	Participation in 7th Krishi Fair at Puri, Odisha from 4-8 June, 2016	Odisha	1,48,990
4.	Participation in 20th National Exhibition at Kolkata from 10-14 August, 2016	WB	1,71,650
5.	Participation in Agri-Asia at Mahatma Mandir, Gandhinagar, Gujarat from 2nd to 4th September, 2016	Gujrat	1,91,500
6.	Organization of National level Krishi Unnati Mela from 25th to 29th September, 2016 at Mathura	UP	52,850
7.	Participation in Exhibition at Grakhpur, U.P. from 23rd to 24th October, 2016	UP	9,490
8.	IITF from 14th to 27th November, 2016 at Pragati Maidan	NCT of Delhi	Yet to be finalized

New Initiatives:

8.33 The Division has introduced a quarterly e-bulletin; monthly e-book and Farmer Friendly Hand Book on yearly basis. These documents are available on Department's website www.agricoop.nic.in.

Programmes of North-Eastern States

8.34 The status of implementation of Schemes in the North Eastern Region is as follows:

a. Support to State Extension Programme for Extension Reforms

S. No.	Name of the State	No. of ATMA Registered
1.	Arunachal Pradesh	22
2.	Assam	26
3.	Manipur	9
4.	Mizoram	8
5.	Meghalaya	11
6.	Nagaland	11
7.	Sikkim	4
8.	Tripura	8
**Total:**		**99**

8.35 During the current financial year 2015-16, Budget estimate of Rs. 4500 lakh was earmarked for North Eastern States for implementing the Scheme. Out of which, an amount of Rs. 2660.79 lakh has been released to Assam (Rs. 583.020 lakh), Arunachal Pradesh (Rs. 467.340 lakh), Manipur (Rs. 304.890 lakh), Meghalaya (Rs. 120.630 lakh), Mizoram (Rs. 393.880 lakh), Nagaland (Rs. 581.580 lakh), Tripura (Rs. 77.220 lakh) and Sikkim (Rs. 132.230 lakh) till November, 2016

b. Mass Media Support to Agricultural Extension:

8.36 List of Narrowcasting and FM Kisan Vani Stations in the North Eastern Region is given below:

Sl. No	Name of the State	Name of Narrowcasting Station	Name of the FM Kisan Vani Station
1	Arunachal Pradesh	-	Itanagar
2	Assam	Tinsukhia, Margheretia,Jorhat, Sonari, Nazira, Dibrugarh	Jorhat, Dhubri, Hafflong, Nowgong
3	Manipur	Imphal, Churachandpur, Ukhrul	--
4	Meghalaya	Shillong, Nongstoin, Jowai, Cherrapunjee	Jowai
5	Mizoram	AizwalLawangtlal Lunglei	Lunglei
6	Nagaland	Gangtok	Mokokchung
7	Tripura	AgartalaKailasahr Tellamura, Amarpur Jolaibari	Kailasahr, Belonia

These stations are producing/ broadcasting 30 minutes agricultural programmes, 5/6 days a week.

c. KisanCall Centers (KCCs):

8.37 The KCC located at Guwahati caters to the needs of the North Eastern Region except Sikkim which is located at Kolkata (West Bengal). Queries are replied in different languages depending upon the area from where the query is received. Since the beginning of scheme, the calls registered from various states of North-Eastern Region upto 31st October are, Arunachal Pradesh (5669), Assam (223808), Manipur (28017), Meghalaya (14191), Mizoram (7650), Nagaland (2925), Sikkim (10040) and Tripura (36341). The calls registered from these states during current year up to 31st October, 2016 are Arunachal Pradesh (582), Assam (20486), Manipur (1257), Meghalaya (729), Mizoram (66), Nagaland (211), Sikkim (657) & Tripura (3257).

d. Extension Education Institute (EEI):

8.38 EEI set up at Jorhat (Assam) in 1987 has been providing training support at the regional level to the middle level functionaries of in 8 States of North Eastern Region and West Bengal. During the year 2016-17 (till October, 2016) 15 courses have been organized and 468 officers trained. An amount of Rs. 89.50 lakhs has been released to EEI Jorhat till October, 2016 as against the budget estimate of Rs. 150.00 lakh.

Gender Specific Interventions under ATMA Programme:

8.39 ATMA programme provides that minimum 30% resources are to be utilized for women farmers. To improve participation of women in planning & decision making process, the scheme provides for representation and active involvement of women at various decision making platforms viz. ATMA-Governing Body and ATMA-Management Committee at district level & Farmer Advisory Committees (FACs) at block, district and state level. Besides, scheme provides for enhanced involvement of women as 'Farmer Friend' in a mechanism promoted under the scheme for Farmer-to-Farmer extension. Farm Women's Food and nutritional Security Groups (FIGs) @ at least 3 per block to be formed annually for ensuring household food and nutritional security providing assistance of Rs.10,000/ per group.

8.40 Since inception of the scheme in 2005-06, total **96,37,720** farm women (26.60% of the total benefited farmers) have participated in farmer oriented activities like Exposure Visits,

Training, Demonstrations & Kisan Melas including **6,31,572** women farmers benefited during 2016-17(up to 30th November, 2016).

8.41 In addition, ATMA supports organizing atleast 2 women Food Security Groups/ block to ensure food and nutritional security at the household and community level.

8.42 For the first time, in the team of committed '**extension functionaries**' engaged with Government of India, support (90%) one '**Gender Coordinator**' at the State level has been included.

Gender Specific Interventions under ATMA Programme:

Sl. No.	Activity/ Program	Details of Activities/ Programs in which women are involved	Tar-get	Achieve-ments	Budget Allocation	Amount Utilized	Remarks
1	ATMA Program	Minimum 30% of resources meant for programmatic activities are to be utilized for women farmers.	-	6,31,572 women farmers have participated in farmers oriented activities during 2016-17 (till 30th November, 2016).	30% of the budget earmarked for farmer oriented filed activities.	26599.20 lakh (Up to 30th November, 2016	To improve participation of women in planning & decision making process, the scheme provides for representation and active involvement of women at various decision making platforms viz. ATMA-GB & ATMA-MC at district level & Farmer Advisory Committees at block, district & state level. Farm Women's Food and nutritional Security Groups (FGGs) @ at least 3 per block to be formed annually for ensuring household food and nutritional security providing assistance of Rs.10,000/ per group.

Development of Portals

8.43 DAC&FW has developed 80 portals, applications and websites (primarily in collaboration with National Informatics Centre) covering both the headquarters and its field offices. The important portals include Soil Health Card, National Agriculture Market, Pradhan Mantri Fasal Bima Yojana (PMFBY), PMKSY, AGMARKNET etc.

National e-Governance Plan in Agriculture (NeGP-A):

8.44 The Government is implementing a state sector plan scheme (erstwhile centrally sponsored) Mission Mode Project (MMP) **National e-Governance Plan in Agriculture (NeGP-A)** for helping farmers to access information related to latest technology. This project has been approved at a total cost of Rs. 858.79 crore for implementation of this scheme in entire country. Dissemination of information to the farmers has been aimed through various delivery channels including Common Service Centres, Web Portals, SMSs and Kisan Call Centres, Mobile apps etc. twelve identified clusters of services under the project is under implementation. The services include Information on Pesticides,

Fertilizers and Seeds; Soil Health; Information on crops, farm machinery, training and Good Agricultural Practices (GAPs); Weather advisories; Information on prices, arrivals, procurement points, and providing interaction platform; Electronic certification for exports and import; Information on marketing infrastructure; Monitoring implementation / evaluation of schemes and programmes; Information on fishery inputs; Information on irrigation infrastructure; Drought Relief and Management; Livestock Management.

mKisan Portal:

8.45 This Portal subsumes all mobile based initiatives in the field of Agriculture & Allied sectors. It brings together SMS (both Push and Pull), Interactive Voice Response System, Mobile Apps and Services. Officers, Scientists and Experts from all Organizations and Departments of the GoI and State Governments (including State Agriculture Universities(SAUs) , Krishi Vigyan Kendras(KVKs) and Agro –Meteorological Field Units (AMFUs) are using this Portal all over the country are using this Portal for disseminating information(giving topical & seasonal advisories and providing services through SMSs to farmers in their local languages) on various agricultural activities to registered farmers, during FY 2015-16, more than **780 crore** SMSs and during FY 2016-17 more than 480 crore SMSs have been sent till 31.12.2016 to farmers by all agencies/ organisation/departments in agriculture and allied sectors down to Block level throughout the country since its inception July-2013. The content may include information about the Schemes, Advisories from Experts, Market Prices, Weather Reports, Soil Test Reports etc. The farmers can register for this service by calling Kisan Call Center on the toll free number 1800-180-1551 or through the Web Portal/SMS.

Mobile Apps.

8.46 As part of multiple channels of delivery of information to the farmers, DAC&FW has already launched five mobile apps (Kisan Suvidha, Pusa Krishi, Crop Insurance, Agrimarket & Bhuwan Hail storm). It has also been decided to make complete information and services available to the farmers through suitable Mobile Apps. Few important Mobile Apps developed by Department are:

(a) **Hail Storm application** :- Farmer or other stakeholder may upload hail storm photograph with GPS tagging.

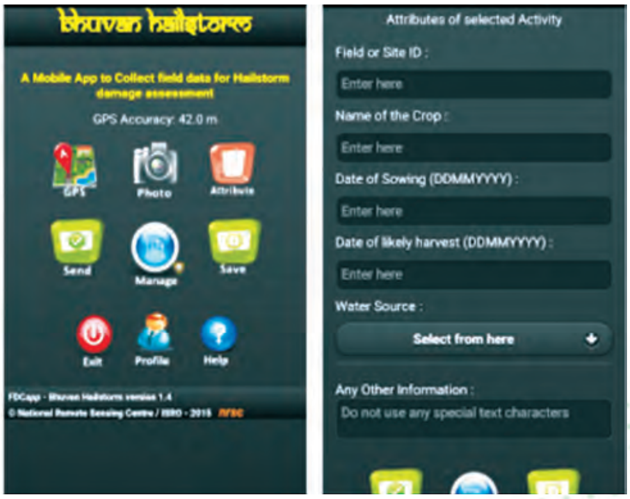

(b) **Crop Insurance**:- Famer can know insurance premium, notified area etc. on the mobile.

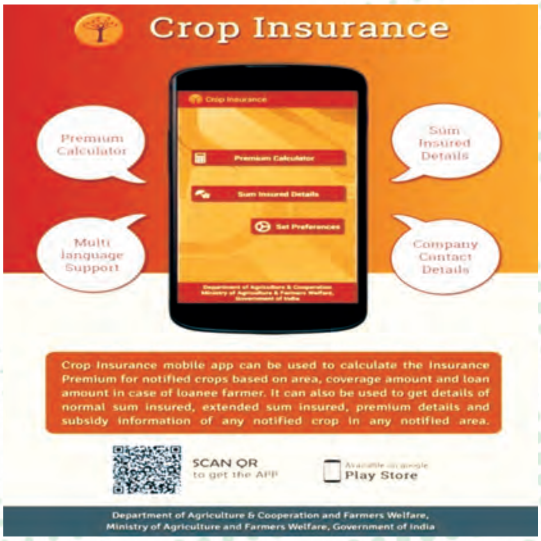

(c) **Agri Market**:- Farmer can know the prices of various crops in the mandis near him.

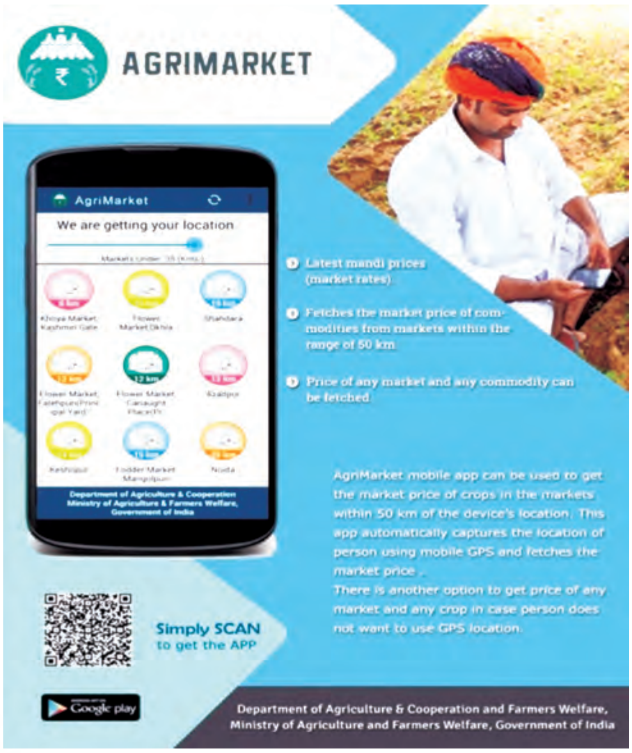

(d) **Kisan Suvidha** has a simple interface and provides information on **five critical parameters** – weather, input dealers, market price, plant protection and expert advisories. An additional tab directly connects the farmer with the Kisan Call Centre where agriculture experts answer their queries. Unique features like extreme weather alerts and market prices of commodity in nearest area and the maximum price in State as well as India have been added to empower farmers in the best possible manner. Kisan Suvidha mobile app is presently functioning in English & Hindi with a facility of language translation. States have been given a facility of translating the app in their own language. This translation facility will help in extending the outreach of this app and thus more and more farmers can be benefited throughout the length and breadth of the nation. It is available in Google Play Store and on mKisan – Mobile Apps section (mkisan.gov.in). Till December 2016 more than 3.7 Lakh users have downloaded the App.

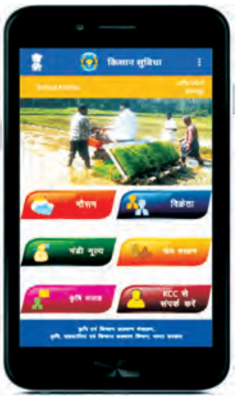

(e) **Pusa Krishi- Mobile App:** Pusa Krishi Mobile App has been launched recently by the Government. The objective of this app is to help farmers to get information about technologies developed by IARI, which will eventually help in increasing returns to farmers.

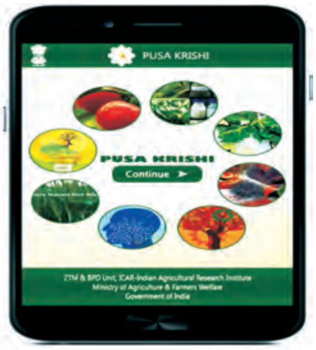

(f) **CCE App:** for undertaking crop cutting experiment.

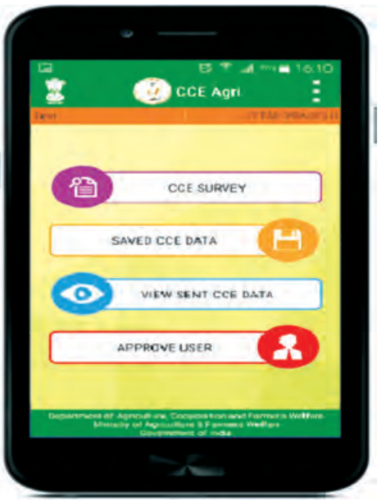

Strengthening of IT Apparatus in Agriculture and Cooperation in the States and Union Territories (AGRISNET):

8.47 The Government is implementing a Central Sector Plan Scheme "Strengthening/ Promoting Agricultural Informatics & Communications", of which one of the components is AGRISNET. The objective of AGRISNET is to provide improved services to the farming community through use of ICT. Under the AGRISNET Scheme funds are released to the State Government concerned for provisioning of software and hardware systems including networking, data digitization, manpower training to ensure computerization upto Block level 26 States have so far availed funds released by the Government under AGRISNET. However, under the revised guidelines under NeGP-A, this scheme has been subsumed under NeGP-A.

SEEDS:

8.48 Sub-Mission on Seed and Planting Material (SMSP):- Central Sector Scheme "Development and Strengthening of Infrastructure Facilities for the Production and Distribution of Quality Seeds" was under implementation on all India basis since 2005-06 with effect from 01.04.2014. This scheme was subsumed in "Sub Mission on Seed and Planting Material (SMSP)" under centrally Sponsored Scheme "National Mission on Agricultural Extension and Technology (NMAET)" with the objective to develop and strengthen the existing infrastructure for production and distribution of certified / quality seeds with a focused, time bound and integrated agenda to improve the availability of quality seeds for the farmers and to encourage the development of new varieties of plants. The existing components of the scheme are as under:

Sl. No.	Intervention
1.	Strengthening for Seed Quality Control
2.	Strengthening of Grow Out Test(GOT) Facilities
3.	Support to Seed Certification Agencies
4.	Seed Village programme
5.	Certified seed production through seed villages
6.	Seed Processing Facilities
7.	Seed Storage Facilities
8.	Transport Subsidy on Movement of seeds to NE States etc
9.	National Seed Reserve
10.	Application of Bio-technology in Agriculture
11.	Public Private Partnership in Seed Sector
12.	Assistance for Boosting Seed Production in Private Sector
13.	Support to Sub-Mission Director and Survey / Studies
14.	PPV&FRA

8.49. Production of Breeder, Foundation and Certified/Quality Seeds:- Seeds are the basic and critical input for agricultural production. The Indian Seeds programme recognizes three generations of seeds, namely, breeder, foundation and certified seeds. The details of production of breeder and foundation seeds as well as production of certified seeds from 2005-06 to 2016-17 are shown in the following table:-

YEAR	Production/Availability of Seed (Metric Tonnes)		
	Breeder Seed (Production)	Foundation Seed	Certified / Quality seed
2005-06	6823	74800	1405000
2006-07	7382	79654	1481800
2007-08	9196	85254	1943100
2008-09	9441	96274	2503500
2009-10	10683	114638	2797200
2010-11	11921	180640	3213592
2011-12	12338	222681	3536200
2012-13	11020	161700	3285800
2013-14	8229	174307	3473130*
2014-15	8621	157616	3517664
2015-16	9036	149542	3435248
2016-17	11221.8 (Target)	220907	3802904

8.50 Strengthening of Seeds Quality Control Organizations (State Seed Certification Agencies and State Seed Testing Laboratories):- The responsibility of seed law enforcement is vested with the State Governments. Seed Inspectors, notified under the relevant provisions of the Seeds Act, 1966 and the Seeds (Control) Order, 1983, inspect the premises of seed distribution agencies to draw samples for testing. Appropriate action is taken against sellers of sub-standard seeds as per the provisions of the Seeds Act/Rules and Orders. These Inspectors are also authorized to stop the sale of sub-standard seeds and to seize their stocks. Seed Inspectors have also been provided with powers of enforcement under the Environment (Protection) Act, 1986 to regulate the quality of genetically modified (GM) Crops.

8.51 Policy on Export/Import of Seeds and Planting Materials:- The export/ import of seeds have increased with rationalization and simplification of the export/import regime. This has benefited Indian farmers, the seed industry and entrepreneurs. 110 cases were recommended for issue of export and 163 cases were recommended for import during the year 2015-16). During the year 2016-17, 112 cases of export and 103 cases of import have been recommended.

8.52 Implementation of Protection of Plant Varieties and Farmers' Rights:- Legislation for Protection of Plant Varieties and Farmers Rights was enacted in year 2001 which provides for the establishment of an effective system for protection of plant varieties rights of farmers and plant breeders and to encourage the development of new varieties of plants. Central Sector Scheme for the Protection of Plant Varieties and Farmers Rights Authority (PPV&FRA) provides necessary back-up support for implementation of this legislation. The major achievements are detailed below:

- Distinctiveness, Uniformity and Stability (DUS) guidelines of 5 new crops/species published in the Gazette of India vide S.No.1444(E) dated 19th April, 2016 Vegetable Amaranth (Amaranthus tricolor L.), Ridge gourd (Luffa acutangula (L.) Roxb.) Palak / Spinach beet (Beta vulgaris var. bengalensisRoxb.)Carnation (Dianthus caryophyllus L.) and Orchid (PaphiopedilumPfitz.). In addition 7 new crops/species guidelines have been published in the Gazette of India vide S.No.2394(E) dated 13th July, 2016namely Noni (Morindacitrifolia L.), Nutmeg (MyristicafragransHoutt.), Bael (Aegle marmelosCorrea), Jamun (SyzygiumcuminiSkeels), Custard Apple (*Annona squamosa*L.), Jasmine (*Jasminumsambac*L.), Kalmegh (*Andrographispaniculata*L.)
- DUS tests guideline of 63 crops/ species areundervariousstagesofdevelopment. Out of these 21 Crops/ species (aonla, neem, karanj, guava, litchi, marigold, betelvine, Deodar, Chir pine, Mulberry, Buckwheat, jasmine, Grain amaranth, faba bean, Proso millet, Kodo millet, Little millet, Barnyard millet, Elephant foot yam, Taro& Jatropha) have already been finalized and published in Plant Variety Journal of India.
- The Authority has organized 334 awareness programme for training, awareness, and capacity building which includes five National seminar, three state level seminar cum agro biodiversity fair and exhibition and three workshop as per the provision of the PPV&FR Act, 2001 to make aware the participants on farmers Rights, Breeders Rights Researcher Right etc.
- Two branch offices have already been established at Ranchi and Guwahati to facilitate the registration of farmer's varieties and other varieties. In addition

to above, it has been decided to establish three new branch offices of the Authority at Palampur (HP), Pune (Maharashtra) and Shivamogga (Karnataka) has been solicited. These branch offices will also monitor DUS centres within their territorial jurisdiction, provide support in the development of DUS tests guidelines, support to organize training cum-awareness programme, besides facilitating farmers for the registration of farmers varieties and other varieties.

- In order to support, reward & recognize farmers, communities of farmers particularly the tribal, rural communities engaged in conservation, improvement and preservation of genetic resources of economic plants and other wild relatives in biodiversity hotspots, Plant Genome Saviour Farmers Reward & Recognition and Plant Genome Saviour Community Award are given each year. This year i.e. on 24th August, 2016 five Farming/ Tribal Communities have also been conferred Plant Genome Saviour Community Award consisting of cash of Rs.10.00 lakhs each, a citation and a memento. 10 Individual Farmers have been conferred Plant Genome Saviour Rewards consisting of cash of Rs. 1.50 lakh each, citation and memento and 4 farmers for Plant Genome Saviour Recognition comprising of Rs. 1.00 lakh each along with citation and memento.
- During the year 2015-16, PPV&FRA received about 2174 applications for registration under the PPV&FR Act and authority issued 419 certificates.
- 78 DUS test centres are being supported by the Authority, which are located in various institution of ICAR, ICFRE, CSIR, other reputed research organization and State Agriculture Universities.

8.53 National Seed Research and Training Centre (NSRTC) at Varanasi, Uttar Pradesh:- The National Seed Research and Training Centre (NSRTC) Varanasi (Uttar Pradesh) has been functioning since Oct.,2005 and has been notified as Central Seed Testing and Referral Laboratory (CSTL) with effect from 01.04.2007. The mandate of NSRTC is to have a separate National Seed Quality Control Laboratory to maintain uniformity in Seed Testing and to assure supply of quality seeds at National Level. It also acts as Referral Laboratory under Court of Law for seed related issues. It is a premiere Institute for capacity building in relation to maintaining Seed Quality assurance by offering HRD activities round the year. The CSTL working under ambit of NSRTC is also a member Laboratory of International Seed Testing Association (ISTA), since 2007. As per National programme to maintain uniformity in seed testing, NSRTC has analysed approximately 19,132 nos. of seed sample during 2015-16 under 5% re-testing programme. Besides, a number of 97 Court Referred Seed Samples have also been analysed in the CSTL as and when received from the respective Court.

8.54 The details of the seed sample received and analyzed during last five years and current year are given as under :

Sl. No.	Year	Total Nos. of Seed Samples received and analyzed	Court Seed Samples received and analyzed
1.	2010-11	13859	66
2.	2011-12	15978	66
3.	2012-13	17808	89
4.	2013-14	18951	106
5.	2014-15	19068	213
6.	2015-16	19132	97
7.	2016-17 (as on 31.10.2016)	14851	62
**Total (Nos.)**		**119647**	**699**

8.55 During 2015-16, NSRTC had organized 10 National Training programmes on various seed related issues, for the benefits of various stakeholders of Public and Private Sector. In addition NSRTC has also organized National Seed Congress at Hyderabad, Andhra Pradesh in 2015-16.

8.56 Department intends to establish Indian International Rice Research Institute (IRRI)'s South Asia Regional Center at campus of NSRTC, Varanasi (UP). The proposed centre of IRRI would help in research and rice value addition in the country.

8.57 National Seeds Corporation: The Department of Agriculture, Cooperation and Farmers Welfare had two Central Public Sector Undertakings namely National Seeds Corporation (NSC) and the State Farms Corporation of India (SFCI), after soliciting the approval of the Cabinet and completion of codal formalities of the Ministry of Corporate Affairs, SFCI has been amalgamated with the NSC w.e.f 1.4.2014 to bring about greater synergy in production and distribution of quality seeds. NSC is scheduled 'B' Central Public Sector Enterprise (CPSE) and also enjoys "Mini Ratna" (Category-I) status. The performance of NSC during 2015-16 is as under: -

- During 2015-16 NSC earned profit after tax (PAT) of Rs. 43.41 crore as compared to Rs. 38.84 crore during 2014-15. NSC declared dividend @ 20% of Rs. 13.79 crore in 2015-16 (including dividend distribution tax) against Rs.8.13 crore @ 15% in 2014-15 on the paid up capital of the corporation. 23.17 lakh Qtl seeds have been produced/procured during the financial year 2015-16. NSC is undertaking seed production of more than 600 varieties/hybrids/ lines including parental lines of about 60 crops consisting of cereals, millets, oilseeds, pulses, fodder, fibers, green manure, potato and wide range of vegetables crops. During the financial year 2015-16 NSC has distributed 13.98 lakh qtls of seed in the country. During the current financial year 2016-17 NSC has distributed 5.03 lakh qtls of seed in Kharif, 2016 season.

8.58 Use of Bt. Cotton Hybrid Seeds:- Bt. Cotton is the only transgenic crop approved in the country for commercial cultivation. The Genetic Engineering Appraisal Committee (GEAC) of the Ministry of Environment, Forests and Climate Change is the nodal agency for grant of permission for environmental release of Bt. Cotton hybrids under the Environment Protection Act, 1986 in the country. At present, about 1128 Bt. Cotton hybrid seeds are available for cultivation in the country. These Bt. Cotton hybrids are grown in ten (10) States i.e., Gujarat, Madhya Pradesh, Maharashtra, Andhra Pradesh, Telangana, Karnataka, Tamil Nadu, Haryana, Punjab and Rajasthan. The area under Bt. Cotton has increased from 29073 ha in Kharif 2002 to 85.29 lakh ha. in 2016-17 (81% of total cotton area)

8.59 National Seed Reserve:- The Establishment & Maintenance of Seed Bank Programme has been re-structured as National Seed Reserve for implementation during the remaining period of 12th Plan w.e.f. 2014-15 – 2016-17. The basic objectives of the scheme are to meet the requirement of seeds of short and medium duration crops varieties to farmers during natural calamities and unforeseen conditions. The National Seed Reserve (NSR) programme is implemented by about 22 implementing agencies in the country namely NSC, State Seeds Corporations and State Department of Agriculture of Tamil Nadu, Himachal Pradesh, Jammu & Kashmir and Jharkhand. Under the programme, one time financial assistance for procurement of seeds called Revolving fund and cost of material handling

equipment is provided to the implementing agency. Assistance is also provided for maintenance of certified and foundation seeds of identified crops, construction of seed godown, establishment of seed processing plant, Cost of Material handling equipment, Cost of service out sourced and computerization & networking facilities, besides price differential cost for left over stock of seeds as per norms of the scheme. The quantity of certified and foundation seeds maintained under NSR and amount released to the participating agencies during 2015-16 and 2016-17 are as under:-

Year	Targets of Physical quantity in lakh quintals	Physical Achievement in lakh quintals	Amount released to implementing agencies (Rs. in lakhs)
2015-16	3.65	2.76	2101.25
2016-17	3.65	3.00 (Anti.)	1281.11 (As on 04.01.17)

8.60 SAARC Seed Bank : India signed the Agreement on establishment and maintenance of SAARC Regional Seed Bank in November, 2011 at the XVII SAARC summit held at Maldives in 2011 with the objective to provide regional support to national seed security efforts; address regional seed shortage through collective actions and foster inter-country partnerships, to promote increase of Seed Replacement Rate (SRR) with appropriate varieties at a faster rate as far as possible so that the use of quality seed for crop production can be ensure; and to act as a regional seed security reserve for the Member States.

8.61 Accordingly, National Seeds Corporation Limited (NSC), New Delhi has been declared as National Designated Agency to coordinate for establishment and maintenance of SAARC Regional Seed Bank in India. The National Designated Agency (NDA) would be entrusted the task of establishing SAARC Seed Bank as per provisions contain in the agreement. It will work as the National Focal Point also. National Seeds Corporation Limited, State Seeds Corporations and State Department of Agriculture are declared as implementing agency to establishment and maintenance of the SAARC Regional Seed Bank in the Country.

8.62 In addition, Government of India constituted a National Technical Committee to coordinate all related activities i.e working out modalities, technicalities of participation to the SAARC Regional Seed Bank in the Country.

8.63 Joint Secretary (Seeds) attended the Inception meeting of the SAARC Seed Bank Board held at Dhaka, Bangladesh from 27th to 28th September, 2016 and necessary steps are being taken to implement the SAARC Seed Bank in the future.

8.64 Seed Village Programme : - In order to upgrade the quality of farmer saved seeds which is about 60-65% of the total seeds used for crop production programme, following interventions are made :

(a) 50% assistance for the seeds for *cereal crops* and *60% for oilseeds, pulses, fodder and green manure crops* is provided for distribution of foundation/certified seeds required for one acre area per farmer.

(b) Farmers' Trainings: Financial assistance of Rs.15000 per group (50-150 farmers each group) is provided for farmers training on seed production and post harvest seed technology (Rs.0.15 lakh)

(c) Seed treating/dressing drums : Financial assistance for treating seeds

produced in the Seed Village is available @ 3500 per seed treating drum of 20Kg capacity and Rs. 5000 per drum of 40Kg capacity.

(d) Seed Storage bins: To encourage farmers to develop storage capacity of appropriate quality, financial assistance will be given to farmers for purchasing Seed Storage bins. The rate of assistance is as under.

@33% for SC/ST farmers for 10 qtls. capacity upto maximum of Rs.1500
@33% for SC/ST farmers for 20 qtls. capacity upto maximum of Rs.3000
@25% for General farmers for 10 qtls. capacity upto maximum of Rs.1000
@25% for General farmers for 20 qtls. capacity upto maximum of Rs.2000

Assistance for purchase of only one seed bin for each identified farmer is available in the seed village programme. The implementing agency may also distribute smaller size of seed bins as per demand of farmer and financial assistance is reduced accordingly.

The year-wise physical progress of the programme is as under : -

Year	Number of Seed Village Organized	Quantity of Seed Produced (In Lakh Quintals)
2006-07	10,778	22.961
2007-08	18,121	40.070
2008-09	35,212	58.009
2009-10	69,127	79.564
2010-11	1,01,067	161.553
2011-12	89,244	199.28
2012-13	78,943	116.708
2013-14	68455	145.14
2014-15	48004	193.71
2015-16	29249	91.82
2016-17	9115	3.41*

* The progress from most implementing agencies is yet to be received after Rabi crops harvest.

8.65 Boosting Seed Production In Private Sector: - Under this component of the Credit linked back ended subsidy @40% of the capital cost of the project in general areas and 50% in case of hilly and scheduled areas subject to an upper limit of Rs 150 lakhs per project is funded. Two percent (2%) of the total fund utilized under the component will be allowed as administrative charges to the Nodal agency. So far, 527 such projects have been sanctioned for the small entrepreneurs in 16 States with 131.37 lakh qtls. seed processing capacity and storage capacity of 41.78 lakh qtls as on 31.10.2016.

8.66 Programme being implemented in the North-Eastern States: - Details of the programmes being implemented in the NE region is at **Annexure 8.1.**

8.67 Modifications in New Policy on Seed Development: The National Seed Policy, 2002 provides that all imports of seeds and planting materials, etc. will be allowed freely subject to EXIM Policy Guidelines and the requirements of the Plants, Fruits and Seeds (Regulation of import into India) Order, 1989 as amended from time to time. Import of parental lines of newly developed varieties will also be encouraged. The Policy also provides that seed and planting materials imported for sale into the country will have to meet minimum seed standards of seed health, germination, genetic and physical purity as prescribed. All importers will make available a small sample of the imported seed to the Gene Bank maintained by National Bureau of Plant Genetic Resources (NBPGR). In order to harmonize New Policy on Seed Development, 1988 with the National Seed Policy, 2002, following two modifications have been made in the New Policy on Seed Development, 1988:

(i) Seeds of wheat and paddy – In order to provide to the Indian farmer the best planting material available in the world to increase productivity, the import of seeds of wheat and paddy

may also be allowed as per provisions of the Plant Quarantine Order, 2003 as amended from time to time for a period not exceeding two years by companies, which have technical/ financial collaboration agreement for production of seeds with companies abroad, provided the foreign supplier agrees to supply parent line seeds/ nucleus or breeder seeds/technology to the Indian company within a period of two years from the date of import of the first commercial consignment after its import has been recommended by DAC. For trial and evaluation of the variety sought to be imported by eligible importers, 16 kg. seed in case of wheat and 5 kg. in case of paddy will be given to ICAR or farms accredited by ICAR for sowing. After receipt of the satisfactory results of trial/evaluation, an eligible importer may apply for the bulk import of such seeds to the DAC.

(ii) Similar procedure of trial/evaluation has been recommended for import of the seeds of coarse cereals, pulses and oil seeds.

Further, Seeds Division has simplified the forms for export and import of seeds and planting materials.

Implementation of OECD Seed Schemes in India

8.68 The objective of the Organisation for Economic Co-operation and Development (OECD) Seed Schemes is to encourage use of seeds of consistently high quality in participating countries. These schemes authorize the use of labels and certificates for seed produced and processed for international trade according to OECD guidelines. India's participation in OECD Seed Schemes aims to enhance its seed export capabilities and probabilities.

8.69 The Department of Agriculture, Cooperation & Farmers Welfare has become a member of OECD Seed Scheme from 23rd October, 2008 and 109 varieties in 20 crops have been entered in OECD list of varieties and 74 varieties of agricultural crops sent to OECD Secretariat for enlisting in the OECD list of varieties. Awareness workshops and hands-on-training and gearing up State Seed Certification Agencies for registration of OECD listed varieties have also been taken up for implementation of OECD Varietal Certification in India. In addition, a Book on OECD varietal certification in India has been published.

8.70 Gender Perspective in the Implementation of SMSP: There is no separate allocation of funds under the schemes of Seeds Division for women farmers. Implementing agencies/ States have been requested to allocate adequate funds for participation of women farmers in Seed Village Programme and also certified Seed production of oilseeds, pulses, fodder and green manure crops through Seed Village.

Sub Mission on Agricultural Mechanization (SMAM)

8.71 Agriculture Mechanization is an essential input to modern agriculture to increase the productivity and for making judicious use of other inputs like seeds, fertilizers, chemicals & pesticides and natural resources like water, soil nutrients etc. besides reducing the human drudgery and cost of cultivation. Agriculture Mechanization also helps in improving safety and comfort of the agricultural worker, improvements in the quality and value addition of the farm produce and also enabling the farmers to take second and subsequent crops making Indian agriculture more attractive and profitable. It also helps the Indian farming to become commercial instead of subsistence. There is a linear relationship between availability of

farm power and farm yield. Therefore, there is a need to increase the availability of farm power from 1.841 kW per ha (2012-13) to 2.0 kW per ha by the end of 12th FY Plan to cope up with increasing demand of food grains.

8.72 As per the Agriculture Census 2010-11, the average size of all land holding is 1.15 ha which was 1.23 ha in the last Agriculture Census of 2005-06. About 85 % of the total land holdings are in small and marginal size groups which need special efforts for its mechanization.

8**.73** Subsequently, recognizing the need to mechanize the marginal and small farmers, and for inclusive growth of Farm mechanization Sector in the country a Sub Mission on Agricultural Mechanization (SMAM) was launched in the year 2014-15 with the following objectives:

- Increasing the reach of farm mechanization to small and marginal farmers and to the regions where availability of farm power is low;
- Promoting 'Custom Hiring Centres' to offset the adverse economies of scale arising due to small land holding and high cost of individual ownership;
- Creating hubs for hi-tech & high value farm equipments;
- Creating awareness among stakeholders through demonstration and capacity building activities;
- Ensuring performance testing and certification at designated testing centers located all over the country.

Financial assistance in SMAM under its various components:

8.74 The financial assistance as cost subsidy to the tune of 25-40% is being provided for the individual ownership of the farm machinery which is also applicable for farm machinery component under RKVY, NFSM, NHM & NMOOP schemes for different categories of Machinery & Equipment. The financial assistance @40% is provided for establishment of farm machinery banks to provide the custom hiring services for the benefits of small and marginal farmers. For installing solar photovoltaic water pumping system, financial assistance of Rs 43200/ horse power to Rs 63,360/Horsepower is provided. To promote the mechanization in selected village with low level of farm mechanization, financial assistance @80% of the project cost for farm machinery banks is given to the group of minimum 8 farmers. Hiring assistance for various farm operations carried out through the farm machinery banks set up under financial assistance is also provided @ 50% of the cost of operation/ ha limited to Rs. 2000 per ha. to farmers.

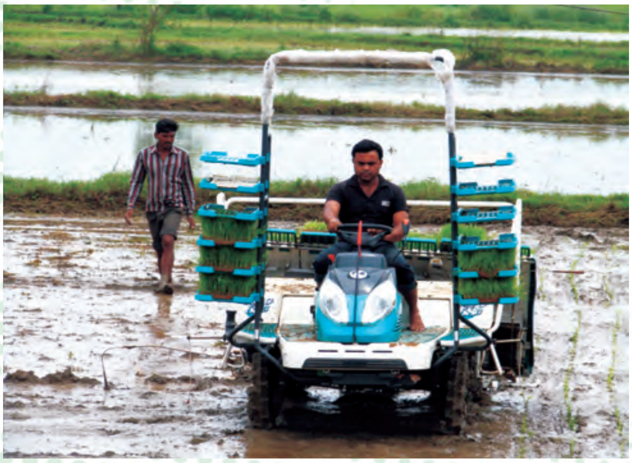

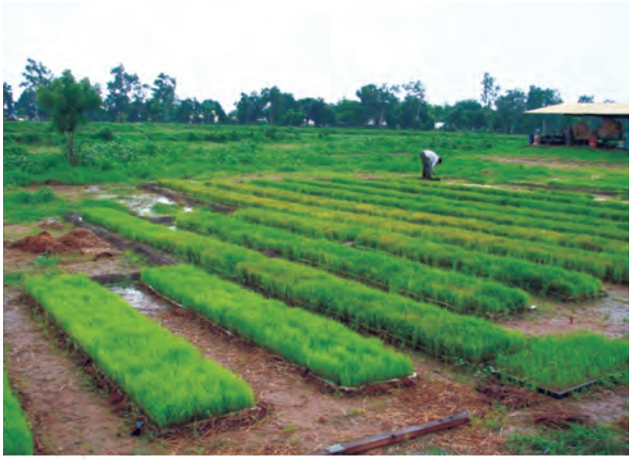

8.75 As a result of different programmes implemented by the Government of India over the years and equal participation from Private Sector, the farm mechanization has been increasing steadily over the years. This is evident from the sale of tractors and power tillers, taken as indicator of the adoption of the mechanized means of farming, during the last five years is given below

Year wise sale of tractors and power tillers.

Year	Tractor Sales (NOS.)	Power tiller Sale (Nos.)
2004-05	2,47,531	17,481
2005-06	2,96,080	22,303
2006-07	3,52,835	24,791
2007-08	3,46,501	26,135
2008-09	3,42,836	35,294
2009-10	3,93,836	38,794
2010-11	5,45,109	55,100
2011-12	5,35,210	60,000
2012-13	5,90,672	47,000
2013-14	6,96,828	56,000
2014-15	5,51,463	46,000
2015-16	5,71,249	48,882

8.76 Average farm power availability for the cultivated areas of the country has been increased from 0.48 kW/ha in 1975-76 to 1.84 kW/ha in 2012-13 and expected to cross 2.0 kW/ha by 2016-17.

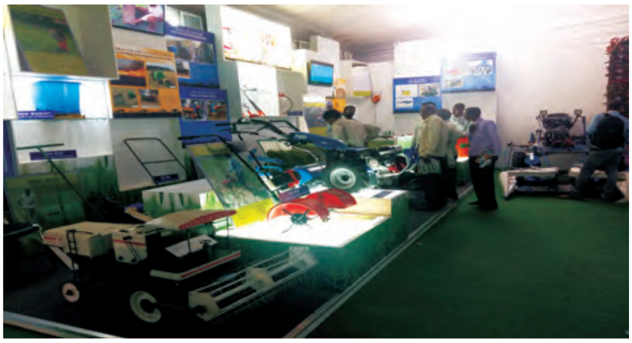

Training of Farmers & Technicians

8.77 The Farm Machinery Training & Testing Institutes (FMTTIs) located at Budni (Madhya Pradesh), Hissar (Haryana), Garladinne (Andhra Pradesh), and Biswanath Chariali (Assam), are imparting training to different categories of beneficiaries like farmers, technicians, under graduate engineers, entrepreneurs and the foreign nationals nominated under international exchange treaty on selection, operation, maintenance, energy conservation and management of agricultural equipments. During the year 2015-16, 7545 persons were trained till 31st March, 2016 against the annual target of 6000 in different courses. During 2016-17, 7801 persons were trained till 31st December 2016 against the annual target of 6000 in different courses. To supplement the efforts of the FMTTIs, outsourcing of the training through the identified Institutions, ICAR Institutions, ATMA Institutions, National innovation Foundation, training programmes are funded as per norms prescribed by Ministry of Skill Development and Entrepreneurship.

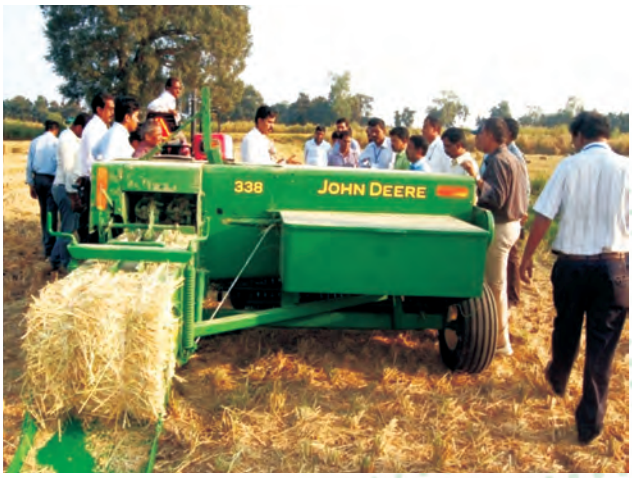

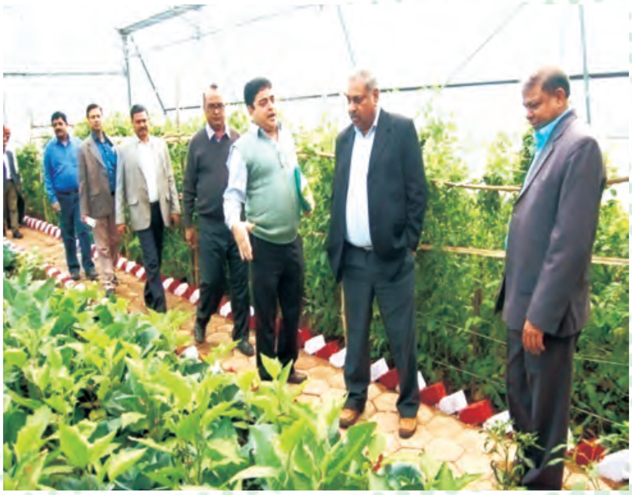

Testing and Evaluation of Farm Machinery & Equipment

8.78 The Institute at Budni is authorized to conduct commercial tests on tractors and other agricultural machines as per relevant BIS test codes besides conducting the testing of tractors and power tillers under CMV Rules 1989. CFMTTI is also National designated authority for testing of tractors as per OECD codes. The institute at Hisar conducts commercial tests on self-propelled combine harvesters, irrigation pumps, plant protection equipment, agricultural implements and other machines as per relevant BIS codes and authorized to issue the certificate under CMVR 1989 in respect of the combined harvesters. The Institute at Garladinne is authorized to test power tillers and also conduct tests on various agricultural implements / equipment components. The institute at Biswanath Chariali (Assam) tests bullock drawn implements, manually operated equipment, tractor drawn implements, self propelled machines and small hand tools. The four FMTTIs altogether have tested 349 machines of various categories, including tractors, power-tillers, combine harvesters, and other machinery and equipments, till 31st March, 2016 against the target of 165 for the year 2015-16. During 2016-17, total 283 farm machines have been tested till 31st December 2016 against the annual target of 165 machineries.

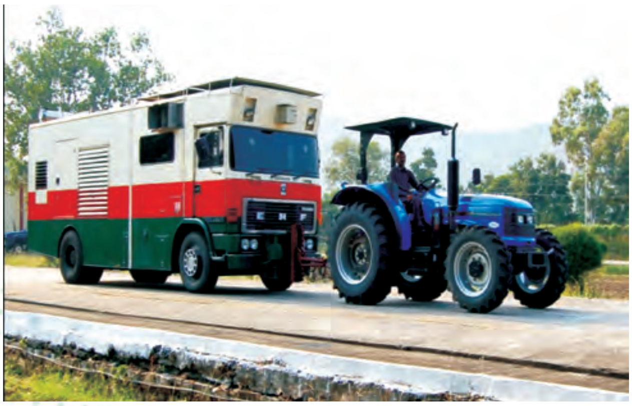

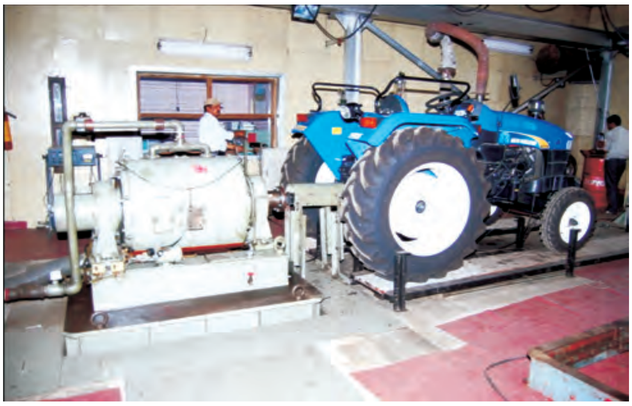

8.79 To cope up with the ever increasing demand of testing of agricultural machines and equipments, DAC & FW has designated 30 testing centers at State Agricultural Universities (SAUs), ICAR Institutions and State Agricultural Departments as Authorized Testing Centers to test selected type of agricultural machinery and equipments under different categories of farm machinery. Relevant information has been made available on the departmental website http://farmech.gov.in for wider publicity among the users/manufacturers and other stake holders. Central Institute of Agricultural Engineering (CIAE), Bhopal and Punjab Agricultural University (PAU), Ludhiana has been designated as authorized testing centers in addition to FMTTI, Hisar and Budni to test tractor mounted combine harvesters. Central Institute of Agricultural Engineering (CIAE), Bhopal and Junagarh Agricultural University (JAU), Junagarh, Farm Machinery Testing, Training and Production Centre, Department of FM&P, Dr. PDKV, Akola, Maharashtra, College of Agricultural Engineering and Technology, Dr. Balasaheb Sawant Konkan Krishi Vidyapeeth, Dapoli Maharashtra, has been designated as authorized testing centers in addition to FMTTI, Hisar to test plant protection equipments. Central Institute of Post harvest Engineering and Technology (CIPHET) Ludhiana has been designated as authorized testing center for testing all types

of post harvest technology equipments and machinery.

8.80 A workshop was held on held on 4-5th April, 2016 at CFMT&TI, Budni (M.P.) where the heads of the designated testing centers have participated to discuss issues related to strengthening of testing centers in terms of infrastructure and man power and transfer of testing fee to testing centers for meeting the recurring expenses etc and other related issues to testing procedure, functioning and documentations.

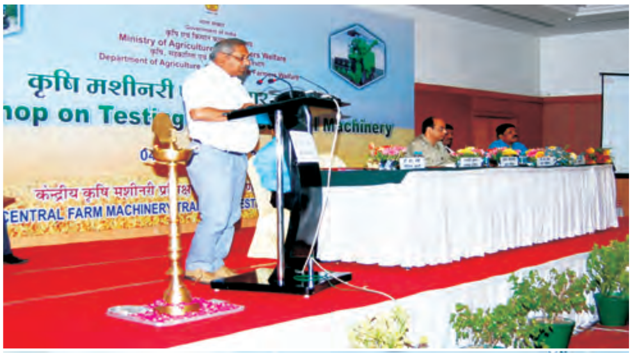

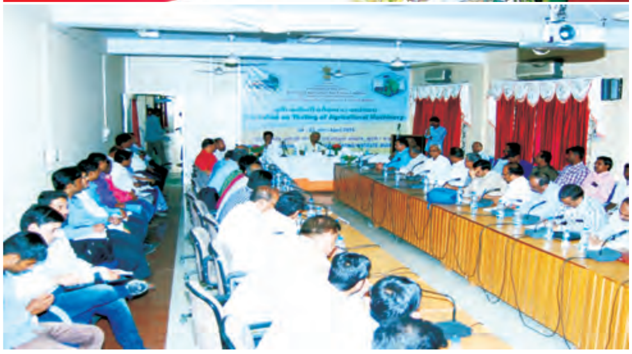

8.81 A Conference on "Innovations in Agricultural Mechanization – Development of linkage among R&D Institutes - Industry – Farmers" was organized at Vigyan Bhavan New Delhi on 7-8th July 2016. More than 800 participants including Scientists, Engineers, Central and state Government officers, Extension workers, Agricultural machinery manufacturers, Progressive farmers Participated in the conference. The agenda and object for the Conference was, aims to linkage between the Agricultural machinery manufacturers, Scientist involved in R&D institutions, Extension functionaries in Agricultural Mechanisation sectors and farmers to promote the new research and innovations for its commercialization so that it can reach to farmers and to identify strategies and outline action areas for development of farm equipment sector. The conference was inaugurated by the Hon'ble Union Minister of Agriculture & Farmers Welfare Shri Radha Mohan Singh. He has released the book namely "Farm Mechanization in India – The Custom Hiring Perspective" on this occasion.

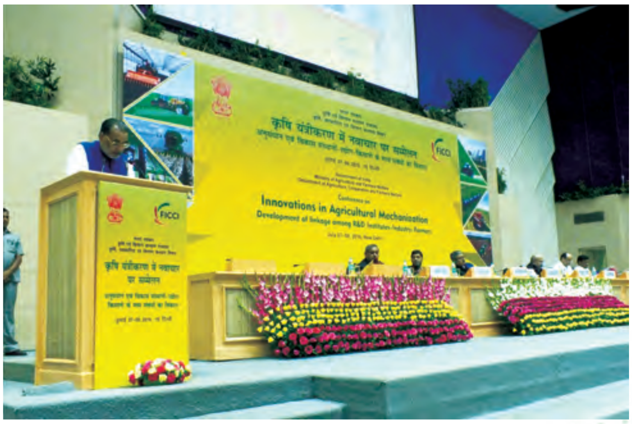

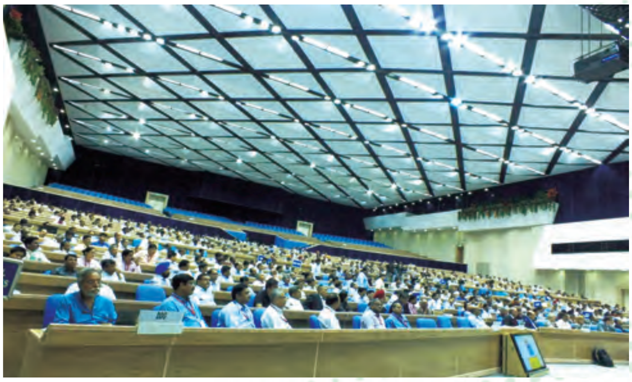

Demonstration of Newly Developed Agricultural/Horticultural Equipment

8.82 To support and encourage the Agriculture mechanization at field level and to bring the awareness of new technologies among the farmers, demonstrations of newly developed agricultural equipment including Post harvesting, horticultural equipment have been included under component 1 & 2 of the SMAM scheme. Under these

components, 100% financial assistance as Center Sector Scheme is provided @Rs 4000 per ha up to 100 ha per season for the field demonstration of farm machinery and post harvesting technology/equipments on farmer's field.

8.83 Custom Hiring Centres: To promote the mechanization of small and marginal farm holdings and farm holding in difficult area with low level of mechanization, the efforts has been made to concentrate on establishment of farm Machinery Bank & Hi tech, high productive farm machinery hubs for custom hiring services. Under the components 4, 5 & 6 of the SMAM total Rs 91.44 crores as cost subsidy, has already been released to States in the first installment to establish 1662 Farm machinery Banks for providing the custom hiring services in the country till December 2016.

8.84 Farm Mechanization Programmes under various schemes of Agriculture: Financial Assistance in the form of subsidy at the rate of 25-40 percent under RKVY, NFSM & NHM, NMOOP is applicable as per guidelines of SMAM to the farmers for individual ownership of agricultural equipment including hand tools, bullock-drawn/ power-driven implements, planting, reaping, harvesting and threshing equipment, tractors, power-tillers and other specialized agricultural machines

8.85 Activities in the North-Eastern States: A FMTTI has been established at Biswanath Chariali in the Sonitpur district of Assam, to cater to the needs of human resource development in the field of agricultural mechanization and also to assess the quality and performance characteristics of different agricultural implements and machines in the region. During 2015-16 total 934 trainees were trained at this institute against the target of 800 and tested 36 nos. of agricultural implements and machinery against the annual target of 25. During 2016-17 (till December 2016) total 788 trainees were trained against the target of 800 and tested 22 nos. of agricultural implements and machinery against the annual target of 25. To support the agricultural Mechanization in North Eastern States where the Mechanization level is very low, the maximum permissible subsidy per machine per beneficiary is provided @ 50% limited to Rs. 1.25 lakhs/beneficiary for procurement of various agricultural machinery and equipments for individual ownership, and 95% of the cost of the machinery/Implement up to Rs 10 lakhs per farm Machinery bank for the establishment of Farm Machinery Banks with a minimum of 8-10 farmers respectively. During the year 2016-17, cost subsidy of Rs. 10.74 crores has already been released in the first installment to NE States under the different components of SMAM.

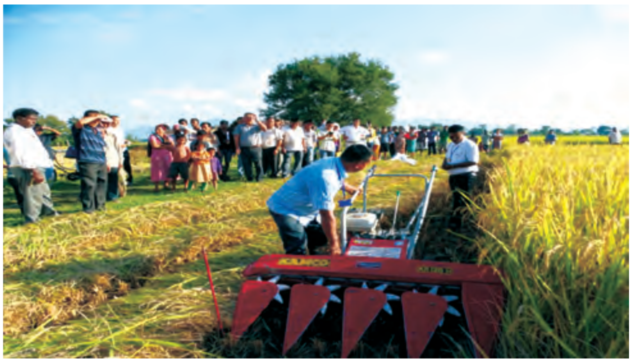

8.86 Gender Friendly Equipment for Women: Under the component 1 of SMAM, Agricultural Mechanization through Training, Testing, and Demonstration, a total 2267 women were trained during the current Financial Year 2016-17, (till December 2016) .A list of about 30 identified gender-friendly tools and equipment developed by the Research and Development Organization for use in different farm operations has already been sent to all states and UTs. for popularizing them. State governments have been directed to earmark 30 per cent of total funds allocated under SMAM for women beneficiaries.

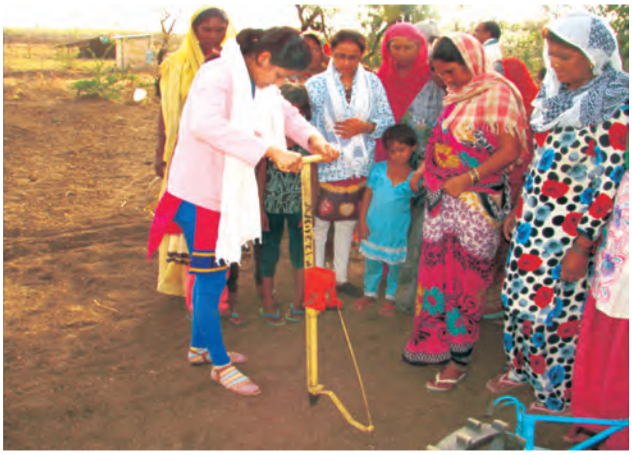

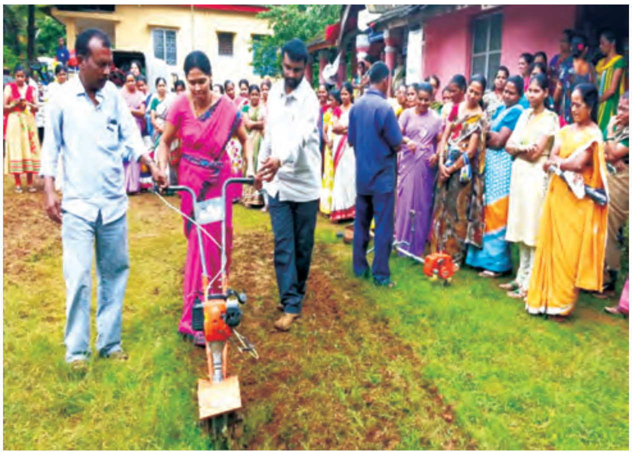

8.87 State Agro Industries Corporations: The Government of India had advised the State Governments in the year 1964, to set up State Agro Industries Corporations (SAICs) in the public sector to act as catalysts in providing access to industrial inputs to farmers, for their use in agriculture. Thus, 17 SAICs were set up in the joint sector with equity participation of the Government of India and the respective State Governments of Andhra Pradesh, Assam, Bihar, Gujarat, Haryana, Himachal Pradesh, Jammu and Kashmir, Karnataka, Kerala, Madhya Pradesh, Maharashtra, Orissa, Punjab, Rajasthan, Uttar Pradesh, Tamil Nadu and West Bengal during 1965 to 1970. Many of the State Governments have increased their equity participation as a result of which the Government of India, at present, is a minority shareholder. SAICs have since expanded their basic functions by commencing manufacture and marketing of agricultural inputs, implements, machines, after-sales service, promotion and development of agro-based units/industries. The Government of India is implementing a policy of disinvestment of its shares in SAICs with a view to give greater decision making power to the State governments by allowing transfer of its shares to state governments.

Sub Mission on Plant Protection and Plant Quarantine (SMPP):

8.88 The primary aim of this Sub mission is to minimize loss to quality and yield of agricultural crops from the ravage of insect pests, diseases, weeds, nematodes, rodents etc. and to shield our bio-security from the incursions and spread of alien species. The sub mission also seeks to facilitate exports of Indian Agricultural commodities to global markets and to promote good agricultural practices, particularly with respect to plant protection strategies and techniques. The destructive Insect and pests Act, 1914 and the Insecticides Act, 1968 provide the legal framework for the regulatory function in this regard. The Sub Mission has 4 important components, (i) Strengthening and Modernization of Plant Quarantine Facilities (SMPQF), (ii) Strengthening and Modernization of Pest Management Approach (SMPMA), (iii) Monitoring of Pesticide Residues at National Level (MPRNL) and the (iv) National Institute of Plant Health Management (NIPHM).

I) Strengthening and Modernization of Plant Quarantine Facilities (SMPQF)

8.89 The objectives of Plant Quarantine is to prevent introduction of exotic pests, diseases and weeds which are likely to be introduced through import of agricultural commodities or plants material into India. Conversely, India is also required to fulfill its obligation under the International Plant Protection Convention (IPPC) with regard to safety

of its agri exports from the Phytosanitary angle.

8.90 Plant Quarantine (Regulation Import into India) Order, 2003 notified under provisions of Destructive Insect and Pest Act, 1914 (DIP Act) provides regulatory framework for imports of Plant and Plant Products. Post entry quarantine inspection is carried out, where needed, in imports of seeds and plant material for propagation. Phytosanitary Certificates (PSCs) are issued for exports as per International Plant Protection Convention (IPPC), 1951 of the Food and Agricultural Organization (FAO). These functions are being discharged through 57 Plant Quarantine Stations (PQS) functioning under Directorate of Plant Protection, Quarantine & Storage (DPPQ&S), Faridabad at various international airports, seaports and land customs stations across the country to facilitate international trade of agricultural products. Technical dossiers for Pest Risk Assessment (PRA) of potential exportable agri- commodities are prepared under this scheme to gain market access in foreign markets. Similarly, market access proposals of our international trading partners are also appraised on technical grounds.

8.91 The funds under SMPQF are utilized for supporting staff, infrastructure, capacity building and laboratories analysis associated with quarantine and phytosanitary responsibilities.

8.92 PQS at Bongaon, Cochin, Kandla, Visakhapatnam, Trivendrum, Mangalore, Tuticorin, Hyderabad, Bangalore & Kakinada, have acquired ISO 9001:2000 certification. In addition to ISO 9001:2008 certification, three major PQS namely Chennai, Mumbai and New Delhi have achieved NABL accreditation; ISO/IEC: 17025:2005 for the Scheme; "Monitoring of Pesticide Residues at National Level". Five major Plant Quarantine Stations namely Amritsar, Chennai, Kolkata, New Delhi, Bangalore and Mumbai have been equipped with modern diagnostic facilities for screening plant material to detect fungal, bacterial and viral diseases and upgraded and notified to the status of Regional Plant Quartine stations w.e.f 29.06.16. Plant Quarantine Station, Goa is started functioning at Goa International Airport, Goa.

8.93 During 2016-17 (01.04.2016 to 31.12.2016) Pest Risk Analysis (PRA) of 10 agricultural commodities were carried out and notified for import. In addition, technical information was provided to the 20 concerned National Plant Protection Organizations for the export of 60 agricultural commodities. **21,857** import permits (IP) were issued for seed and plant material and screening of **140** lakh metric tons of imported agricultural commodities were undertaken. Phytosanitary certification of **171.42** lakh metric tons of plant and Plant products was conducted for export and **2,89,446** number of Phytosanitary Certificates (PSC) were issued. 565 Pest Control Agencies were accredited for undertaking fumigation with methyl bromide (NSPM-12) as on 31.12.2016 including 25 new agencies and 408 agencies have been accredited for Forced Hot Air Treatment (FHAT) under NSPM-09 for wood and wood packing material including 42 new agencies.

8.94 Further, 400 Pest Control Agencies were accredited for undertaking fumigation with Aluminum Phosphide under NSPM-22 including 65 new Agencies. In addition to above, 86 Rice Processing Mills for export of Rice to USA and 30 for China & 52 units for Peanut processing and 42 pack houses for fresh fruit and vegetables have been registered to ensure pest free export of agricultural commodities to the foreign countries.

Ease of Doing Business

8.95 The web based **Plant Quarantine Information System (PQIS)** provides plant quarantine services related to the online issuance of import permits (IP), Phytosanitary Certificates (PSC) and Import Release Orders (IRO) to importers and exporters (traders) which is being Integrated with customs Gateway (ICEGATE) for seamless integration and single window facilitation to importers and exporters with a view to improve the environment for doing business.

8.96 Further, the PQ import procedures have been simplified and elaborated for Phytosanitary inspection and Plant Quarantine import clearance of plants/plant products & other regulated articles providing stipulated timelines to facilitate trade.

(II). Strengthening and Modernization of Pest Management Approach (SMPMA)

8.97 Integrated Pest Management (IPM): The Government is committed to popularize Integrated Pest Management (IPM) under a Central sector Schemes "**Strengthening and Modernization of Pest Management Approach (SMPMA) in India". The scheme seeks to promote** cultural, mechanical and biological methods of pest control and recommends use of chemical pesticides as a measure of last resort. The Central Government has established 35 Central Integrated Pest Management Centers (CIPMCs) in 29 States and 01 Union Territory. The mandate of these Centers is to conduct pest /disease monitoring, production and release of bio-control agents/bio-pesticides, conservation of bio-control agents and human Resource Development in IPM approach. Training is imparted to Agriculture/ horticulture Extension Officers and farmers at grass root level through season long training programmes, Human Resources Development Programmes and Farmers Field Schools (FFSs). The FFS provides practical training to farmers on the principal of IPM: survey and surveillance for pests and friendly insects ,use of locally available bio-control agents, cultural, physical, mechanical methods of pest control, use of bio-pestcides, effects of pesticides on natural enemies of pests and safe and judicious use of pesticides.These schools are conducted separately for the Kharif and Rabi seasons, each FFS lasting for 14 weeks. During **2016-2017(upto December, 2016)**, **376 FFSs** were conducted in which **11,280** farmers were trained.

8.98 Apart from conducting FFS, the CIPMCs carry out pest/disease monitoring and conservation, production and release of bio-control agents. Pest and disease situation was surveyed by covering an area of **06.53 lakh** hectares. A total of **14.24 crore** bio-control agents were mass produced in laboratories and released against targeted insect-pests in various crops. Augmentation and conservation was taken up in **682.42 thousands hectares**. Under short duration HRD programme a total of **58**(two days) and **08**(five days) programmes were also organized. **02** number of Season Long Training Programmes (SLTPs) were also organized in Cotton & Soyabean crops through which **80 Master Trainers** were trained. **Eighty four (84 Nos)** IPM Packages of practices for pest/diseases management in major crops have been developed. The revised IPM Packages of practice have also been circulated to State Department of Agriculture/horticulture/ICAR Institutions/ State Agriculture Universities & all states/ UTs. A national plan on rodent pest management is under implementation to build trained manpower, create awareness, involve community based organizations and launch rodent control campaigns at village level at identified endemic areas.

8.99 Seed treatment Campaign: Seed treatment and Grow Safe Food were also taken up. Seed treatment is the application of chemical and biological agents on seeds to control primary soil and seed borne infestation of insects and diseases, which are serious threats to crop production. Since 2006, seed treatment campaign is taken up every year by Department of Agriculture Co-operation and Farmers Welfare (DAC&FW) during Kharif and Rabi seasons involving State Agriculture Departments and CIPMCs.

8.100 Grow Safe Food (GSF) Campaign: Pesticides are critical inputs in agriculture as, protect crops from depredations from pests and diseases. However, pesticides are toxic substances and are likely to leave behind traces in crops which may be in excess of the Maximum Residue Limits, if applied in an indiscriminate and unapproved manner. Pesticides are, therefore, subjected to rigorous evaluation at the time of registration and specific conditions for application are clearly spelt out. If the methodology for pesticide application on crops is followed in accordance with the instructions on labels and leaflets of pesticide containers, the likelihood of discovery of pesticide residue in crops above the safe limit become virtually nonexistent.

8.101 In recent times, reports are being received from some parts of the country regarding indiscriminate use of pesticides in disregard of approved methods of application. As a consequence, residues are being discovered, particularly in crops like fruits, vegetables, spices etc. which are sometimes in breach of the safety limit. Such residues can have a deleterious effect on human health and is therefore, a cause for public concern. The Department of Agriculture, Cooperation and Farmers Welfare has been working in tandem with State Government, pesticides manufactures and farmers to create an all round awareness on the need to grow safe food.

8.102 Pesticide residue levels are being monitored on an all India basis and State Governments are being apprised whenever instances of pesticide over-use come to light. Advisories are being sent to pesticides dealers to build awareness on the importance of instruction on pesticide labels and leaflets for production safe food and other agricultural produce. Dealers are being exhorted to disseminate this vital message among their farmer customers.

8.103 A ‘Grow Safe Food’ campaign has been initiated to carry the message of safe and judicious use of pesticides to farmers and other stakeholders. A simple message on the five essential principles of judicious pesticides use – application of pesticides on the right crop, against pests for which the pesticide has been approved, at the right time, in approved doses, and as per approved method of application - is sought to be conveyed through hoardings, banners etc. in regional languages in Gram Panchayats and rural areas. A logo has been developed to signify the importance of the endeavor.

Implementation of Insecticides Act, 1968:

8.104 The insecticides Act, 1968 regulate import, manufacture, sale, transportation, distribution and use of insecticides with a view to prevent risk to humans or animals, and for matters connected therewith. Registration Committee, constituted under Section 5 of the Act, is empowered to register the pesticides/insecticides under Section 9 of the Act after verifying that it is efficacious and safe for use by farmer. In order to bring about greater transparency and efficiency in the process of registration of pesticides, on-line registration of insecticides has been partially operationalised. The system enables partial on-line filing of applications for registration in all categories viz under section (4)/9(3B)/9(3)/Export/Endorsements. The Certificates of Registration under section

9(4) along with label and leaflets are being generated from the data base of 9(3) created in the Sectt. of CIB&RC in Computerized Registration of Pesticides **(CROP)** Software. Label/leaflets of pesticides containers have been revised to facilitate farmers in the safe use of pesticide.

Central Insecticide Laboratory (CIL)

8.105 The quality of pesticides is monitored by Central and State Insecticide Inspectors who draw samples of insecticides from pesticide manufactures/ dealers for analysis in 68 State pesticide testing laboratories (SPTLs) spread across 23 States and one Union Territory. For the States which do not have facilities for testing pesticides, Regional Pesticide Testing Laboratories (RPTLs) have been set up by Central Government at Chandigarh and Kanpur. In case of dispute, samples are referred to Central Insecticides Laboratory (CIL), Faridabad. For quality assurance, the CIL has obtains accreditation from National Accreditation Board for Testing and Calibration Laboratories (NABL) for biological and chemical testing as per ISO/ IEC17025:2005. The RPTLs have also obtained NABL accreditation for chemical testing. Keeping in view the growth in consumption of bio-pesticides and need to regulate their quality, assistance for setting up bio-pesticides testing facilities is being provided to states. Eight labs and NIPHM have been notified for testing bio-pesticides which are at par with CIL for bio pesticides testing. Funds are provided to State Government for setting up/ strengthening pesticide testing labs.

8.106 Creation of necessary infrastructure is under process at National Pesticide Reference Repository (NPRR) and National Pesticide Investigational Laboratory (NPIL). NPRR will store and supply reference standards etc. necessary for analysis of pesticide samples while NPIL will help detect presence of chemical pesticides in microbial bio-pesticide products. This laboratory will also house a technical audit division for pesticide testing laboratories. NPRR and NPIL are expected to bring about a qualitative change in standards of quality testing of pesticides.

Techno-Legal Cell (TLC)

8.107 Techno-Legal Cell **(**TLC) co-ordinates between Directorate and RPTLs and DAC and States for various purposes like NABL accreditation of the laboratories, grant-in-aid to the states and UTs for setting up of new state pesticides Testing Laboratories(SPTLs) and strengthening of existing SPTLs and Bio-pesticides Testing Laboratories in the states. Techno-legal cell also provides support to states pertaining to various technical issues like supply of reference standards and methods of analysis etc. NABL accreditation has been achieved for both the Regional Pesticides Testing Laboratories i.e. RPTL Chandigarh & RPTL Kanpur and State Pesticides Testing Laboratories (SPTL) in Punjab (03 Nos), Maharashtra (03 Nos), and in Karnataka state (04 Nos).

8.108 The cell is also involved in notification of Central Insecticide Inspectors (CIIs) in official Gazette of Govt. of India. Sincere efforts are also made by TLC to prevent manufacturing and sale of spurious pesticides with the help of these inspectors and with co-operation of all State Govt. For this purpose, CIIs are notified for conducting raids/ inspection in manufacturing, distribution and trading premises etc. in routine and on specific complaints received from various sources.

8.109 A total grant-in-aid of Rs 239 lakh was provided for setting and strengthening up of State Pesticide/ Bio-pesticide testing laboratories in the States of Arunachal Pradesh ,Nagaland, Jharkhand, Rajasthan and Manipur during 2015-16.Similarly,a grant in aid of Rs 60 lakh has also been provided to the states of Andhra Pradesh for strengthening up of Bio-pesticides

testing laboratories in the state during 2016-17.Teams were deputed for conducting inspection and verification of complaints received in TLC through different sources in various states like Maharashtra, West Bengal, Rajasthan, Karnataka and Gujarat. Efforts have been made by TLC to prevent activities related to illegal import of pesticides in the country. Few Consignment of pesticides imported illegally by Indian firms were detained by the custom authorities on the directions of TLC. Necessary action as per the provisions of the Insecticide Act, 1968 is being taken against all those firms which have been found to be involved in illegal import of pesticides. A total of 486 samples of Chemical pesticides,09 samples of Bio-products and Bio-pesticides and 08 samples of pesticides from imported consignments have been drawn during the year 2015-16. Prosecution against firms which have been found to be involved in illegal import and manufacturing of pesticides during 2015-16 have either being launched or in progress. A total of 108 samples of pesticides have been drawn by Central insecticide inspectors during the year 2016-17, till date and 05 numbers of cases have also been launched. Further, launching of prosecution is in progress in 27 other cases of misbranded and illegal import found in the year 2015-16.

8.110 National Pesticide Reference Repository (NPRR) to address issues of supply of reference standards and variation in analysis of pesticide samples has been established for detecting presence of chemical pesticides in microbial bio-pesticides products. **National Pesticides Investigational laboratory (NPIL)** has also been established. Procurement of equipment is underway. NPRR and NPIL would be fully functional shortly.

Locust Control & Research

8.111 The scheme Locust Control and Research is being implemented through an Organisation known as Locust warning Organisation (LWO) established during 1939 and later merged with directorate of PPQ&S in 1946. The locust Warning organisation (LWO) monitors locust development and its activities over an area of 2.00 lakh Sq Km of the Scheduled Desert Area(SDA) mainly in the States of Rajasthan and Gujarat and partly in the States of Punjab and Haryana.

8.112 The scheme has 10 Locust Circle Offices (LCOs) located at Bikaner, Jaisalmer, Barmer, Jalore, Phalodi, Nagaur, Suratgarh, Churu in Rajasthan and Palanpur & Bhuj in Gujarat with its field Headquarters at Jodhpur and Central Headquarter at Faridabad. Besides, there is one Field Station for Investigation on Locust (FSIL) situated at Bikaner. To strengthen the locust monitoring and forecasting, an advanced device viz.e-locust 3 to monitor the Desert Locust activities in the fields and software RAMSES to prepare vegetation maps and collect survey data based on satellite imageries for locust forecasting has been adopted. LWO maintains its own wireless network for exchanging of information on locust survey and control between various field offices and Hqrs. Faridabad. Satisfactory locust control potential is being maintained in the form of pesticides, plant protection equipments, wireless sets, GPS, eLocust3 and trained technical and mechanical staff. Presently, the total staff strengthen in the Scheme LC&R at various levels is 247 (Central Hqr-5, Field Hqrs Jodhpur-46, Field Circle Offices-175 and FSIL Bikaner-26).

National Desert Locust Situation and Control Campaign

8.113 India continued to remain free from any Solitary /gregarious phase of desert locust activities during the period up to October 2016. A close liaison was maintained throughout the period under report with FAO Hqrs Rome, regional and international agencies/organisations associated with

the locust control through exchange of fortnightly Locust situation Bulletins.

8.114 The Pesticide Management Bill 2008, which is intended to replace Insecticide Act, 1968 to provide for a more effective regulatory framework for introduction and use of pesticides in the country was in the Parliament (Rajya Sabha) on 21.10.2008 .The Parliament Standing Committee on Agriculture laid its report on the Bill in Parliament on 18.02.2009. The recommendations of the Standing Committee have been examined and taking into account these recommendations, the official amendments to the Pesticides Management Bill have been finalized for introduction into Parliament.

(III) Monitoring of Pesticide Residues at the National Level:

8.115 This Scheme was launched in 2005, as a Central Sector Scheme to collect, collate and analyze data and information on a centralized basis, on prevalence of pesticide residues in agricultural products at farm-gate and market yards. Samples of agricultural commodities and food commodities including animal produce are drawn and analyzed in 25 participating laboratories of Central Government and Agricultural Universities. All the participating laboratories are accredited by National Accreditation Board for Testing and Calibration of Laboratories (NABL) in the field of pesticide residue analysis as per ISO/IEC 17025:2005. The pesticide residue data generated is shared with concerned State Governments for corrective action for judicious and proper use of pesticides on Crops with an integrated pest management approach.

(iv) Capacity Building in Plant Protection:

8.116 National Institute of Plant Health Management (NIPHM) is an institute for plant health under Ministry of Agriculture and Farmers Welfare. NIPHM has entered into MOUs with select institutions and started new courses, including training courses for officers of DPPQ&S. During 2016-17 (up to December, 2016) 115 regular training courses were conducted at NIPHM wherein 1544 officers from various States and Organizations were trained. NIPHM has started regular PGDPHM one year course for students during the current year. NIPHM has conducted a training programme on Pest Detection and Identification (QPDI), which was conducted during the above period with 7 international and 18 National participants. International participants participated for the first time in this program.

8.117 For the first time, NIPHM has conducted the Regional Health System Analysis (RPHSA) program with 10 confirmed international and 11 national participants, which is the 4th in the series, the first three were with USDA Collaboration.

Women in Plant Protection:

8.118 Indian women folk have been playing significant role in agriculture since times immemorial and are the major partners in all the agricultural activities and operations, be it sowing, transplanting, weeding, rouging, picking, plucking, harvesting or threshing. As far as plant protection is concerned, women have played a vital role in weeding, rouging (of pest infested plants/parts) and also in the pesticide spray operations etc. in every part of the country. Women are imparted plant protection skills and methods through Farmers Field Schools (FFSs) organized by CIPMCs.

National Agricultural Bio-Security System:

8.119 In view of threat perception to bio-security of country on account of increasing international trade, emergence of trans-boundary diseases of plants and animals (such as Ug-99 wheat stem rust and avian

influenza), introduction of genetically modified organism,climate change and bioterrorism. Ministry of Agriculture & Farmers Welfare has prepared a proposal for putting in place a National Agricultural Bio-security System (NABS). The proposal is based on recommendation of National Commission on Farmers headed by Prof. M.S. Swaminathan and National Policy on Farmers 2007.The proposal envisages creation of an autonomous National Agricultural Bio-security Authority (NABA) for which an Agricultural Bio-security Bill, 2013 was introduced in the Lok Sabha on 11.03.2014 but lapsed with dissolution of the Lok Sabha. In order to re-introduce the Bill in the Lok Sabha, the draft Cabined Note was prepared and sent to Cabinet Secretariat and Prime Minister's Office (PMO). As per the advice of PMO, the Note was placed before an Informal Group of Ministers headed by Home Minister on 01.06.2016. The meeting adjourned with the directions that assessment of comparative merits of other options, including strengthening of the existing system, visa us that of the Agricultural Bio-security Authority be carried out.

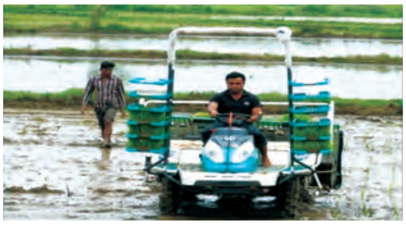
Self Propelled Paddy Trans-planter

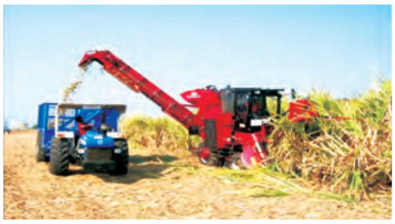
Sugarcane Harvester

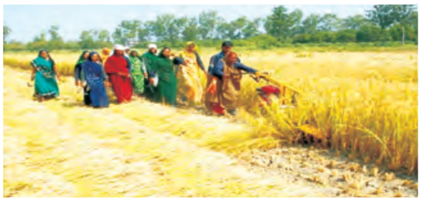
Self propelled Vertical conveyor reaper

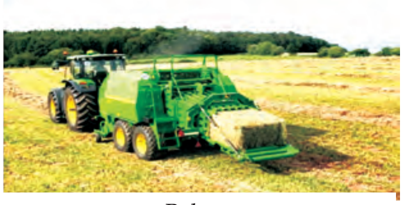
Baler

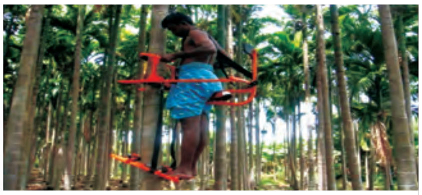
Coconut tree Climber

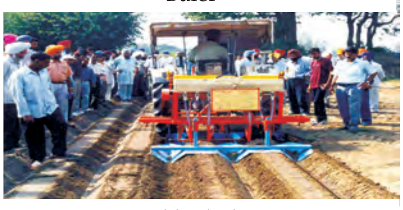
Raised bed Planter

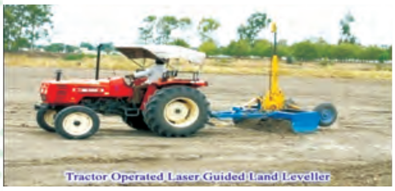

Laser land leveler

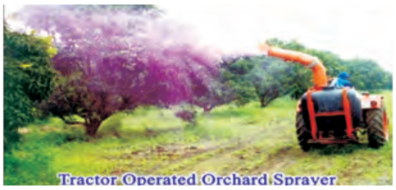

Aero Blast Sprayer for tall crops, and trees

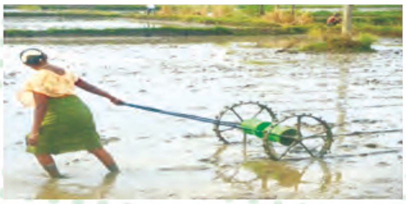
Pre germinated paddy seeder

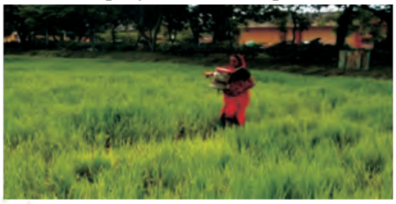
Manual broadcaster

******

Chapter 9

National Crop Insurance Programme (NCIP)

9.1 Keeping in view the risks involved in agriculture and to insure the farming community against various risks, Ministry of agriculture introduced a crop insurance scheme in 1985 and thereafter brought improvements in the erstwhile scheme(s) from time to time based on the experience gained and views of the stakeholders, States, farming community etc. To enlarge the coverage in terms of farmers, crops and risks, National Agricultural Insurance Scheme (NAIS) was notified/ implemented with effect from 1999. To make the crop insurance schemes more farmers' friendly, a re-structured Central Sector crop insurance scheme namely, "National Crop Insurance Programme (NCIP)" was implemented by merging erstwhile Pilot schemes of Modified National Agricultural Insurance Scheme (MNAIS), Weather Based Crop Insurance Scheme (WBCIS) and Coconut Palm Insurance Scheme (CPIS) (as its components) with some improvements for its full-fledged implementation from Rabi 2013-14 season throughout the country. National Agricultural Insurance Scheme (NAIS) was to be discontinued after implementation of NCIP from Rabi 2013-14 season. However, on the representations and at the option of States, NAIS was also allowed for implementation upto 2015-16. The erstwhile crop insurance schemes have further been reviewed in consultation with various stakeholders including States/UTs. and new scheme namely, **Pradhan Mantri Fasal Bima Yojana (PMFBY)** has been approved for implementation from Kharif 2016 along with pilot Unified Package Insurance Scheme (UPIS) and Restructured Weather Based Crop Insurance Scheme (RWBCIS).

9.2 The Crop Insurance schemes remain optional for State Governments and they may notify crops and areas according to provisions of scheme like earlier schemes. The existing Schemes of PMFBY & RWBCIS are being implemented in the country on 'Area Approach' basis where yield of notified areas under PMFBY and weather data of notified Reference Automatic Weather Stations (AWSs) under RWBCIS are taken as one unit for assessment/ payment of claims for widespread calamities. However, claims are also being assessed & paid on the basis of losses at individual farms due to localized calamities of hailstorm, landslides and inundation under PMFBY and add-on/ index-plus coverage for hailstorm and cloudburst under RWBCIS. The penetration/ coverage of these schemes in terms of number of farmers has reached about 30% of the total number of land-holdings in the country. PMFBY targets coverage of 50% of the total cropped area of the country during next two to three years.

9.3 The total funds released by Government of India under various schemes for crop insurance are as under:

(Rs. crore)

Plan/ Year	NAIS (since Rabi 1999-2000)	WBCIS (since Kharif 2007)	MNAIS (since Rabi 2010-11)	CPIS (since 2009-10)	PMFBY (since Kharif 2016)	Total
IX Plan (1997-2002)	811.49	-	-	-	-	811.49
X Plan (2002-07)	2626.84	-	-	-	-	2626.84
XI Plan (2007-12)	5851.88	1370.37	87.15	1.95	-	7311.35
XII Plan (2012-17)					-	
2012-13	700.00	655.00	194.18	0.50	-	1549.68
2013-14	1600.00	700.00	251.02	0.50	-	2551.52
2014-15	1543.56	470.00	584.79	Nil	-	2598.35
2015-16	1937.79	630.79	413.89	Nil	-	2982.47
2016-17	6201.79	-	-	Nil	1753.41	7955.21
**Total**	**21273.35**	**3826.16**	**1531.03**	**2.95**		**28386.91**

* as on 31.12.2016

9.4 During the last 33 crop seasons (i.e from Rabi 1999-2000 to Rabi 2015-16), 3705 lakh farmers were covered over an area of 5156 lakh hectares insuring a sum amounting to Rs.653134 crore. Total premium of Rs.31364 crore were collected against the claims of Rs.61503 crore benefiting 1438 lakh farmers.

9.5 Brief details of the current schemes and erstwhile schemes are given below:

Pradhan Mantri Fasal Bima Yojana (PMFBY)

9.6 The erstwhile crop insurance schemes have recently been reviewed in consultation with various stakeholders including States/ UTs. As a result of the review, a new scheme **"Pradhan Mantri Fasal Bima Yojana (PMFBY)** has been approved for implementation from Kharif 2016 along with pilot Unified Package Insurance Scheme (UPIS) and restructured Weather Based Crop Insurance Scheme (WBCIS). Under the PMFBY, a uniform maximum premium of only 2% will be paid by farmers for all Kharif crops and 1.5% for all Rabi crops. In case of annual commercial and horticultural crops, the maximum premium to be paid by farmers will be only 5%. The premium rates to be paid by farmers are very low and balance premium will be paid by the Government, to be shared equally by State & Central Government, to provide full insured amount to the farmers against crop loss on account of natural calamities. There is no upper limit on Government subsidy.

9.7 Earlier, there was a provision of capping the premium rate which resulted in low claims being paid to farmers. This capping was done to limit Government outgo on the premium subsidy. This capping has now been removed and farmers will get claim against full sum insured without any reduction. The use of technology will be encouraged to a great extent. Smart phones will be used to capture and upload data of crop cutting experiments to reduce the delays in claim payment to farmers. Remote sensing will be used to reduce the number of crop cutting experiments. The salient features of PMFBY are as under:-

- PMFBY provides a comprehensive insurance cover against failure of the crop thus helping in stabilising the income of the farmers and encouraging them to adopt innovative practices.
- The Scheme envisages coverage of all Food & Oilseeds crops and Annual Commercial/Horticultural Crops for

which past yield data is available and for which requisite number of Crop Cutting Experiments (CCEs) will be conducted being a part of the General Crop Estimation Survey (GCES).

- The scheme is compulsory for loanee farmer obtaining Crop Loan /KCC account for notified crops. However, voluntary for Other/non loanee farmers who have insurable interest in the insured crop(s).
- The Maximum Premium payable by the farmers is 2% for all Kharif Food & Oilseeds crops, 1.5% for Rabi Food & Oilseeds crops and 5% for Annual Commercial/Horticultural Crops.
- The difference between premium and the rate of Insurance charges payable by farmers is shared equally by the Centre and State.
- The seasonality discipline is same for both loanee and non-loanee farmers.
- The scheme is implemented by AIC, all companies of General Insurers' (Public Sector) Association (GIPSA) and other empanelled private general insurance companies. Selection of Implementing Agency (IA) will be done by the concerned State Government through bidding.
- The existing State Level Co-ordination Committee on Crop Insurance (SLCCCI), Sub-Committee to SLCCCI, District Level Monitoring Committee (DLMC) is responsible for proper management of the Scheme.
- The Scheme is implemented on an 'Area Approach basis'. The unit of insurance shall be Village/Village Panchayat level for major crops and for other crops it may be a unit of size above the level of Village/Village Panchayat.
- The Threshold Yield (TY) shall be the benchmark yield level at which Insurance protection shall be given to all the insured farmers in an Insurance Unit Threshold of the notified crop will be moving average of yield of last seven years excluding yield upto two notified calamity years multiplied by Indemnity level.
- Three levels of Indemnity, viz., 70%, 80% and 90% corresponding to crop Risk in the areas is available for all crops.
- The Loss assessment for crop losses due to non-preventable natural risks will be on Area approach.
- However losses due to localised perils (Hailstorm, landslide & inundation) and Post-Harvest losses due to specified perils, (Cyclone/Cyclonic rain & Unseasonal rains) shall be assessed at the affected insured field of the individual insured farmer.
- In case of majority of insured crops of a notified area are prevented from sowing/planting the insured crops due to adverse weather conditions that will be eligible for indemnity claims upto maximum of 25% of the sum-insured.
- There is also a provision on account of claims in case of adverse seasonal conditions during crop season viz. floods, prolonged dry spells, severe drought, and unseasonal rains. On account payment upto 25% of likely claims will be provided, if the expected yield during the season is likely to be less than 50% of normal yield
- In case of smaller States, the whole State shall be assigned to one IA (2-3 for comparatively big States). Selection of IA may be made for at least 3 years.
- The designated/empanelled companies participating in bidding have to bid the premium rates for all the crops notified / to be notified by the State Govt. and

non-compliance will lead to rejection of company's bid

- Crop Cutting Experiments (CCE) shall be undertaken per unit area /per crop, on a sliding scale, as prescribed under the scheme outline and operational guidelines. Improved Technology like Remote Sensing. Drone etc will be utilised for estimation of yield losses.
- State governments should use Smart phone apps for video/image capturing CCEs process and transmission thereof with CCE data on a real time basis for timely, reliable and transparent estimation of yield data
- The cost of using technology etc. for conduct of CCEs etc will be shared between Central Government and State/U.T. Governments on 50:50 basis.
- .The claim amount will be credited electronically to the individual Insured Bank Account.
- Adequate publicity needs to be given in all the villages of the notified districts/ areas
- A crop insurance Portal www.agri-insurance.gov.in has been developed for better administration, coordination amongst Stakeholders, transmission/ dissemination of information and transparency.

9.8 During first season i.e. Kharif 2016 about 374 lakh farmers for a sum insured of Rs. 141487 crore have been covered under PMFBY & WBCIS.

Restructured Weather Based Crop Insurance Scheme (RWBCIS)

9.9 With the objective to bring more farmers under the fold of crop insurance and to overcome the shortcoming regarding delay in settlement of claims etc under erstwhile NAIS, a pilot Weather Based Crop Insurance Scheme (WBCIS) was launched in 20 States (as announced in the Union Budget 2007-08). However WBCIS was implemented as a full-fledged component scheme of NCIP from Rabi 2013-14 season to Rabi 2015-16. WBCIS intends to provide insurance protection to the farmers against adverse weather incidence, such as deficit and excess rainfall, high or low temperature, humidity etc. which are deemed to impact adversely the crop production. It has the advantage to settle the claims within shortest possible time. Under WBCIS, actuarial rates of premium were charged. Recently the scheme has further been restructured on the basis of premium structure and administrative lines of PMFBY and available in the country from Kharif 2016 as Restructured WBCIS. State-wise cumulative progress achieved is given in **Annexure 9.1.**

Coconut Palm Insurance Scheme (CPIS)

9.10 The Coconut Palm Insurance Scheme (CPIS) is implemented since the year 2009-10 in the selected areas of Andhra Pradesh, Goa, Karnataka, Kerala, Maharashtra, Orissa and Tamil Nadu. The scheme is implemented as a full-fledged component scheme of NCIP from Rabi 2013-14 season and is still under implementation in all coconut growing States. The scheme is administered by Coconut Development Board. Insurance coverage is extended to the total loss of the individual palm in the age group of 4 to 60 years for dwarf and hybrid and 7 to 60 years for tall variety, leading to either death of palm or the palm becoming useless due to various natural and other perils. The farmers should have at least 5 healthy nut bearing palms in the age group of 4 to 60 years in contiguous area/ plot and should have been enrolled by State Agriculture/ Horticulture Department or Coconut Development Board or any other such agency under rehabilitation/ development/ expansion scheme. The Sum

Insured is based on the average input cost of the plantation and the age of the specific plant. The Sum Insured varies from Rs. 900 per palm (in the age group of 4-15 years) to Rs. 1725 per palm (in the age group of 16-60 years). The premium rate per palm ranges from Rs. 9.00 (in the age group of 4 to 15 years) to Rs. 14.00 (in the age group of 16 to 60 years) and it varies on sum insured per year, considering the age of specific palm. Fifty per cent of premium is contributed by GOI; 25% by the concerned State Government and the remaining 25% by the farmer. The Insurance Company i.e. Agriculture Insurance Company (AIC) of India Ltd. is the implementing agency of the scheme. The CPIS is being administered/ implemented by the Coconut Development Board (CDB).

9.11 The details of coverage under CPIS as on 31.12.2016 are as under:

Coconut Palm Insurance Scheme (CPIS)-cumulative (from 2009-10 to 2015-16)

(₹ in Lakh)

State	No. of farmers	No. of Palms	Sum Insured	Premium				Claims	Farmers benefitted
				Farmer	State	CDB	Total		
Andhra Pradesh	767	83618	1034.21	3.02	2.99	6.01	12.03	0.00	0
Goa	240	59643	692.50	0.91	0.91	1.83	3.66	1.63	14
Karnataka	704	96422	1067.64	1.83	1.52	3.05	6.09	3.61	73
Kerala	52063	2081126	22469.28	29.60	29.60	59.20	118.40	357.41	5877
Maharashtra	14659	1066749	12844.84	22.56	22.56	45.12	90.23	36.09	396
Odisha	687	21558	353.61	0.68	0.68	1.35	2.70	0.16	3
Tamil Nadu	3781	741974	6527.50	11.22	11.22	22.44	44.89	112.25	384
West Bengal	692	28558	328.42	0.82	0.00	0.82	1.64	0.00	0
**TOTAL**	**73593**	**4179648**	**45318.00**	**70.65**	**69.49**	**139.82**	**279.63**	**511.15**	**6747**

Source: Department of Agriculture, Cooperation & Farmers Welfare(Credit Division)

Unified Package Insurance Scheme (UPIS)

9.12 Unified Package Insurance Scheme has also been approved for implementation in selected 45 districts on pilot basis from Kharif 2016 to provide financial protection & comprehensive risk coverage of crops, assets, life, and student safety to farmers. Pilot will include seven section Viz., crop Insurance (PMFBY/WBCIS), Loss of Life (PMJJBY), Accidental Death & Disability (PMSBY), Student Safety, Household, Agriculture implements & Tractor. Crop Insurance Section is compulsory. However, farmers can choose atleast two section from remaining. Farmers may be able to get all requisite insurance products for farmers through one simple proposal/ application Form. Two flagship schemes of the Government viz PMSBY & PMJJBY have been included apart from insurance of assets. Pilot scheme will be implemented through single window. Premium of PMSBY & PMJJBY is to be transferred to insurance companies which have tied up with the concerned banks. Processing of claims (other than Crop Insurance) on the basis of individual claim report.

9.13 Erstwhile Schemes: NAIS & MNAIS

National Agricultural Insurance Scheme (NAIS)

9.14 With a view to provide financial support to farmers in the event of loss/ failure of any of the notified crop in the notified areas as a result of natural calamities (flood, drought etc), pests and diseases, National Agricultural Insurance Scheme (NAIS) was introduced in the country from Rabi 1999-2000 season in place of erstwhile Comprehensive Crop Insurance Scheme (CCIS). NAIS was to be discontinued after implementation of National Crop Insurance Programme (NCIP) from Rabi 2013-14 season. However, on the representations from States, NAIS has been allowed to continue since 2013-14. The scheme has been discontinued after Rabi 2015-16 on implementation of PMFBY from Kharif 2016 season.

9.15 The scheme was optional for States/ Union Territories (UTs). The scheme is implemented by 26 States and 2 UTs in one or more seasons. The scheme is demand-driven. However, the progress of the scheme can be measured in terms of farmers/ area covered, sum insured, premium collected, claims paid and farmers benefited. State-wise details of farmers covered, area covered, sum insured etc under NAIS since its inception i.e Rabi 1999-2000 to Rabi 2015-16 are given in **Annexure 9.2.**

Modified National Agricultural Insurance Scheme (MNAIS)

9.16 The scheme was introduced as pilot scheme in Rabi 2010-11 and thereafter implemented as a full-fledged component of NCIP from Rabi 2013-14 to Rabi 2015-16. The scheme has been withdrawn after Rabi 2015-16 on implementation of PMFBY from Kharif 2016. State-wise cumulative details of coverage till Rabi 2015-16 under the scheme are given in the **Annexure 9.3.**

******

Chapter 10

Integrated Scheme on Agriculture Census & Statistics

10.1 Recognizing the predominance of agriculture sector in the economy, collection and maintenance of data relating to agricultural holdings assumes vital importance. Agriculture Census is conducted on quinquennial basis to collect information related to structural characteristics of operational holdings in the country. As part of World Agriculture Census (WCA) programme, first comprehensive Agriculture Census was carried out in the country with agricultural year 1970-71 as the reference period. The current Agriculture Census 2015-16 is tenth in the series.

10.2 The Agriculture Census provides crucial information on the structural aspects of Indian Agriculture which continues to be the main stay of the Indian Economy. The concepts and definitions used in the Indian Agriculture Census are broadly in conformity with those adopted in the World Census of Agriculture. The basic statistical unit for data collection in Agriculture Census is 'Operational Holding' rather than 'Ownership Holding', as the farm level decisions are taken by persons who operate land and not by those who own it. The reference period for Agriculture Census is the Agricultural year (July- June).

10.3 The Agriculture Census is conducted in three phases. Periodic Agriculture Census is the main source of information on number, area and other basic characteristics of operational holdings such as land use, cropping pattern, irrigation & tenancy status, pattern of input use etc. The results of Agriculture Census are published in the form of reports and are placed on the website of the Department at http://agcensus.nic.in for public use.

10.4 Agriculture Census Scheme, now a component of the Integrated Scheme on Agriculture Census, Economics and Statistics, was converted from a Centrally-Sponsored Scheme to a Central Sector Plan Scheme during 2007-08. Accordingly, 100 per cent financial assistance is provided to States/ UTs for payment of salaries, office expenses, honoraria, tabulation and printing of schedules, etc.

10.5 Agriculture Census Division also coordinated the Situation Assessment Survey (SAS) of Agricultural Households, 2013 conducted by the National Sample Survey Office (NSSO), Ministry of Statistics & Programme Implementation. This survey was a comprehensive socio-economic study covering farming practices, possession of productive assets, awareness and access to modern technology, resource availability, indebtedness etc. of farmers in the country. Funds for the survey were provided by Department of Agriculture, Cooperation & Farmers Welfare. The reports of the survey were released by the NSSO and made available on the website http://www.mospi.gov.in/ for public use.

Agriculture Census 2015-16

10.6 The tenth Agriculture Census with reference year 2015-16 has been launched in the country during the State Agriculture

Census Commissioner's meeting held on 3rd February, 2016 at New Delhi. Towards conduct of constituent activities of Agriculture Census 2015-16, the following steps were taken.

- After consulting with various stakeholders, the data collection/ compilation instruments were prepared and circulated to the States.
- The detail work programme specifying timeline for related activities of the Census programme has been finalized.
- The training of associated staff of various States/UTs was organized.
- As scheduled, the field work of Phase-I of Agriculture Census 2015-16 has been started from July, 2016.
- Using the computerized database of land records, data for first Phase of Agriculture Census 2015-16 are being extracted in the States of Maharashtra, Gujarat, Andhra Pradesh and Telengana on experimental basis.
- The first review meeting on Agriculture Census 2015-16 of State Agriculture Census Commissioners and Technical Officers was organized on 16th September, 2016. During this meeting, the data entry software developed by the Agriculture Census Division was also demonstrated to the States/UTs.
- At the State / UT level, the State Level Coordination Committee (SLCC) under the Chairmanship of State Agriculture Census Commissioner reviews the work of Agriculture Census for timely completion.
- Other measures taken for monitoring the work of Agriculture Census include field visits, regular interaction with implementing agencies, fortnightly and monthly reports on physical and financial progress etc.

Gender Perspective in Agriculture Census

10.7 Since 1995-96, following recommendations of the Central Statistics Office, Ministry of Statistics and Programme Implementation, gender based data in Agriculture Census have been collected. The scope of collection of gender based data have been restricted to number of operational holdings, corresponding operated area by different size classes of holdings, social group (SC, ST and others), and types of holdings (individual, joint and institutional).

10.8 Percentage of female operational holdings as per results of latest Agriculture Census 2010-11 and Agriculture Census 2005-06 is given in the following table.

Sr. No.	Size Group	2005-06*	2010-11
1	Marginal (Below 1.00 ha.)	12.60	13.63
2	Small (1.00-2.00 ha.)	11.10	12.15
3	Semi-Medium (2.00-4.00 ha.)	9.61	10.45
4	Medium (4.00-10.00 ha.)	7.77	8.49
5	Large (Above10.00 ha.)	6.00	6.78
	**All Size Groups**	**11.70**	**12.78**

* Excludes Jharkhand

10.9 Increase in percentage of female operational holders during different Agriculture Censuses indicates participation of more and more women in operation and management of agricultural holdings in the country.

******

Chapter 11

Agricultural Marketing

11.1 The Government is playing an important role in developing Agriculture Marketing system in the country. The Marketing Division of Department of Agriculture, Cooperation & Farmers Welfare (DAC&FW) is concerned with policy and programme implementation for Agricultural Marketing Reforms and Schemes of Integrated Scheme for Agricultural Marketing (ISAM), and National Agricultural Market (NAM) through Agri-Tech Infrastructure Fund (ATIF).

11.2 Agriculture sector needs competitive and well- functioning markets for farmers to sell their produce. In order to remove restrictive and monopolistic practices of present marketing system, reduce the intermediaries in supply chain and enhance private sector investment in development of post-harvest marketing infrastructure to reduce wastages and to benefit farmers through access to global markets, reforms in agricultural markets have to be an ongoing process. Accordingly, the Ministry of Agriculture formulated a model APMC Act in consultation with the States/UTs, and circulated the same during 2003 and its rules in 2007 for adoption by the States/UTs.

11.3 In order to have more focussed and result oriented efforts on marketing reforms, the Ministry further identified 7 essential areas of market reforms which could be pursued with the States in a focused manner.

(i) Establishment of private market yards / private markets managed by a person other than a Market Committee;

(ii) Establishment of farmer/consumer market by a person other than Market Committee (Direct sale in retail by the farmers to the consumers);

(iii) Direct wholesale purchase of agricultural produce by processors/ exporters/ bulk buyers, etc at the farm gate

(iv) Provision for Contract Farming;

(v) Unified single license/ registration for trade transaction in more than one market;

(vi) Provision for e-trading; and

(vii) Single point levy of market fee across the State.

11.4 The ongoing Central Sector Schemes implemented by the Division during XII Plan were integrated into a new scheme viz. the Integrated Scheme for Agricultural Marketing (ISAM) w.e.f. 01.04.2014. The ISAM has five sub schemes namely (i) Agricultural Marketing Infrastructure (AMI) {the erstwhile schemes of Grameen Bhandaran Yojana (GBY) and the Scheme for Development/Strengthening of Agricultural Marketing Infrastructure, Grading and Standardisation (AMIGS) have been subsumed into AMI sub scheme}, (ii) Marketing Research and Information Network (MRIN), (iii) Strengthening of Agmark Grading Facilities (SAGF) (iv) Agri-business Development (ABD) through Venture Capital Assistance (VCA) and Project Development Facility (PDF) and (v) Ch. Charan Singh National Institute of Agricultural Marketing (NIAM), Jaipur.

The first three sub schemes are implemented by Directorate of Marketing & Inspection (DMI), the fourth sub scheme by Small Farmers Agri-Business Consortium (SFAC), New Delhi and fifth sub scheme by NIAM, Jaipur.

11.5 Directorate of Marketing and Inspection (DMI): The Directorate, set up in the year 1935 as an attached office of the Ministry of Agriculture, is responsible for integrated development of marketing of agricultural and allied produce in the country with a view to safeguard the interests primarily of the producer-sellers as well as of the consumers. It maintains a close liaison between the Central and the State Governments in implementation of agricultural marketing policies in the country.

11.6 The Directorate is headed by the Agricultural Marketing Adviser to the Government of India (AMA). It has its Head Office at Faridabad (Haryana), Branch Head Office at Nagpur (Maharashtra), 11 Regional Offices located at Delhi, Kolkata Mumbai, Bhopal, Chennai, Kochi, Hyderabad, Guwahati, Lucknow, Jaipur and Chandigarh, 26 Sub offices spread all over the country, the Central Agmark laboratory at Nagpur and 11 Regional Agmark Laboratories (RALs) at Delhi, Kolkata, Mumbai, Rajkot, Bhopal, Chennai, Kochi, Guntur, Kanpur, Jaipur and Amritsar.

11.7 Major functions of DMI:

- To guide States/UTs on reforms in their Agricultural Marketing Regulations and for development and management of agricultural produce markets.
- Implementation of sub schemes of Integrated Scheme for Agricultural Marketing (ISAM) viz. (i) Agricultural Marketing Infrastructure (AMI) (The schemes of Grameen Bhandaran Yojana (GBY) implemented since 01.4.2001 and Scheme for Development/ Strengthening of Agricultural Marketing Infrastructure, Grading and Standardization (AMIGS) implemented from 20.10.2004 have been subsumed into Agricultural Marketing Infrastructure (AMI) sub scheme of ISAM w.e.f. 01.04.2014). (ii) Marketing Research and Information Network (MRIN); (iii) Strengthening of Agmark Grading Facilities (SAGF).
- Promotion of Standardization and Grading of agricultural and allied produce under the Agricultural produce (Grading & Marking) Act, 1937 as amended in 1986.
- Marketing Extension including the AGMARKNET portal.
- Training of Personnel in Agricultural Marketing.

Agricultural Marketing Infrastructure (AMI):

11.8 Under the AMI sub-scheme, there are two components (i) Storage Infrastructure (ii) Marketing Infrastructure other than Storage. The main objectives of the AMI schemes are to develop agricultural marketing infrastructure for effectively managing marketable surplus of agriculture including horticulture and of allied sectors including dairy, poultry, fishery, livestock and minor forest produce, promoting innovative and latest technologies and competitive alternative agricultural marketing infrastructure by encouraging private and cooperative sector investments, direct marketing, creation of scientific storage capacity, Integrated value chains (confined up to primary processing stage only) and to provide Infrastructure facilities for grading, standardization and

quality certification of agricultural produce. In addition the scheme aims to create general awareness and provide training to farmers, entrepreneurs, market functionaries and other stakeholders on various aspects of agricultural marketing including grading, standardization and quality certification.

11.9 The scheme is reform linked and State agency projects of those States/Union Territories that have undertaken reforms in their respective APMC Acts to allow/permit (i) 'Direct Marketing', (ii) 'Contract Farming' and (iii) agricultural produce markets in private and cooperative sectors will be eligible for assistance under the sub-scheme. Notwithstanding the reform status, state agencies in all States/UTs will be eligible to avail assistance for storage infrastructure projects.

However, Projects promoted by private entrepreneurs other than State agencies are eligible to avail assistance under the sub-scheme, irrespective of the reforms undertaken by the State Government/UTs in their respective APMC Acts.

11.10 The assistance under the sub-scheme is available to Individuals, Group of farmers / growers, Registered Farmer Producer Organisations (FPOs), Partnership/ Proprietary firms, Companies, Corporations, Non-Government Organizations (NGOs), Self Help Groups (SHGs), Cooperatives, Cooperative Marketing Federations, Autonomous Bodies of the Government, Local Bodies (excluding Municipal Corporations for storage infrastructure projects), Panchayats, State agencies including State Government Departments and autonomous organization / State owned corporations such as Agricultural Produce Market Committees & Marketing Boards, State Warehousing Corporations, State Civil Supplies Corporations etc.

11.11 Under the Storage Infrastructure component of AMI sub scheme for the projects in special category areas of North Eastern States, Sikkim, UTs of Andaman & Nicobar and Lakshadweep Islands & Hilly areas, subsidy @33.33% with a subsidy ceiling of Rs. 1333.2 per tonne with a maximum subsidy ceiling of Rs. 400 Lakhs is extended. For the projects of Registered FPOs, Panchayats, Women, SC & ST beneficiaries or their cooperatives/Self Help Groups, in other areas, subsidy @33.33% with a subsidy ceiling of Rs. 1166.55 per tonne up to 1000 MT and Rs. 1000 per tonne for more than 1000 MT, with a maximum subsidy ceiling of Rs. 300 Lakhs is extended. For all other categories of beneficiaries in other areas, subsidy @25% with a subsidy ceiling of Rs. 875 per tonne up to 1000 MT and Rs. 750 per tonne for more than 1000 MT, with a maximum subsidy ceiling of Rs. 225 Lakhs is extended.

11.12 Since inspection of the GBY from 01.04.2001 and up to 31.12.2016, a total number of 37,574 godowns having a capacity of 630.94 lakh MT have been sanctioned and subsidy of Rs. 2,405.78 crore has been released. The target for the XII plan is 230.00 lakh MT and the achievement during the XII Plan up to December, 2016 is 313.77 lakh MT.

11.13 Under the Marketing Infrastructure Projects other than Storage component of AMI sub scheme in special category areas of North Eastern States, Sikkim, Stats of Uttrakhand, State of Himachal Pradesh, Jammu & Kashmir, UTs of Andaman & Nicobar and Lakshadweep Islands, Hilly and tribal areas, subsidy @ 33.33% with a subsidy ceiling of Rs. 500 lakhs is extended. For the projects of Registered FPOs, Women, SC & ST beneficiaries or their cooperatives, in other areas, subsidy @33.33% with a

maximum subsidy ceiling of Rs. 500 lakhs with is extended. For all other categories of beneficiaries in other areas, subsidy @25% with a maximum subsidy ceiling of Rs. 400 lakhs is extended.

11.14 Since inception of AMIGS from 20.10.2004 upto 31.12.2016 a total of 18,331 projects have been sanctioned and subsidy of Rs. 1,621.02 crores has been released. The target for the XII plan is 4000 projects and the achievement during XII plan upto December, 2016 is 9499 projects. Presently the AMI scheme is not available for the promoters of General Category other than North Eastern Region from 05.08.2014 due to paucity of funds. For promoters of SC, ST categories and north eastern regions, the scheme is not available from 31.12.2016.

11.15 Marketing Research and Information Network (MRIN) Sub Scheme: This scheme was launched as Central Sector Scheme in March, 2000 with objective to collect and disseminate information on price, arrival and other market related data for the benefit of farmers and other market users. DMI has been implementing the scheme in collaboration with Agricultural Marketing Boards/Directorates, APMCs and NIC.

11.16 Wholesale prices and arrivals information in respect of more than 300 commodities and 2000 varieties are being disseminated through the portal on daily basis. 3288 wholesale markets have been linked to the AGMARKNET portal (http://agmarknet.gov.in) and more than 2700 markets have been reporting the data. In addition to price, several other market related information like accepted standards/grades, labelling, sanitary and phyto-sanitary requirement, physical infrastructure of storage and warehousing, marketing laws, fees payable etc. are being provided.

11.17 Mandi price information is disseminated through DD Kisan channel and Kisan Call Centres. New innovations have been introduced for dissemination of market data through Agrimarket and Kisan Suvidha Mobile APPs and through SMS by IFFCO Kisan Sanchar Ltd. etc. at grass root level. The portal also has linkages with various organizations concerned with agricultural marketing. Besides spot price, the portal also provides access to future price, MSP and international commodity price, e-directory of markets, CODEX Standards etc.The Agmarknet portal and National Agricultural Market Atlas portals have been revamped and hosted with better look and user friendly format.

11.18 Marketing Extension: Quality control programme under Agmark as well as different Plan schemes of the Directorate are given wide publicity through mass media. The information is disseminated through documents, video spots, printed literature, exhibition, conferences, seminars and workshops. The Directorate is conducting National Consumer day Celebrations on 24th December and World Consumer Day Celebrations on 15th March by the Field Offices, participating in IITF from 14-27 November, at Pragati Maidan, New Delhi, and AAHAR at New Delhi from 10-14 March. The Directorate is also organizing National Agmark Exhibition every year in important cities. The Directorate is also participated in Krishi Unnati Mela in March, 2016.

11.19 Strengthening of Agmark Grading Facilities (SAGF) Sub Scheme: The Agricultural Produce (Grading and Marking) Act, 1937 provides for the grading and marking of agricultural produce. It involves framing of grades, standards and certification of agricultural commodities included in the scheduled appended to the Act. This

programme requires analysis of check samples and research samples in Agmark Laboratories. SAGF scheme aims to meet the expenditure for the purchase of equipment, chemicals, glassware and apparatus, AMC of the equipments as well as renovation and repair works in the Agmark Laboratories/ Regional and Sub offices. With this support, 11 Regional Agmark Laboratories and a Central Agmark Laboratory, Nagpur are carrying out analysis of check samples and research samples for developing and promoting grading and standardization of agricultural commodities under Agmark.

11.20 As on date, 105 grading and Marking Rules covering 213 commodities, have been notified under the provisions of AP (G&M) Act, 1937. These include fruit and vegetables, cereals, pulses, oil seeds, vegetable oils, ghee, spices, honey etc. Grading and marking of agricultural commodities is voluntary as per provisions of the Act.

Agricultural Marketing Reforms (MDRC):

11.21 With the coordinated and integrated strides made by policy makers, agricultural scientists and more so by farmers, the foodgrain production has now gone up more than five times since beginning of first five year plan in 1951 and growth graph of horticultural crops has even exhibited better picture. Even after this considerable increase in production and productivity in agriculture sector, agrarian economy is still under stress and it is frequently heard that farmers are committing suicides here and there, which indicates that farmers are still not getting their due for their hard work.

11.22 Therefore, in order to achieve the goal of doubling farmers' income in time bound manner, agriculture sector, for inclusive growth, needs to have a well-functioning, competitive and transparent markets in addition to focus on increasing the production & productivity and simultaneously reducing the cost of cultivation. Reforms in agricultural marketing sector is considerate tool to not only offer remunerative prices to the farmers, reduce marketing cost & margin and put tab on food inflation but also augment in doubling the farm income and consequently releasing farmers' from economic stress.

11.23 Department during the year revitalised the reform agenda with the objective to actualise it at the ground level and favourably affect farmers' income. As a part of reform agenda, Department, with the objective to create barrier free market, enhance competition & transparency in transactions and widen choices to the farmers for sale of their produce, launched National Agriculture Market (e-NAM) on 14th April, 2016. Under this ambitious project, 585 wholesale regulated markets are proposed to be linked to e-NAM portal by 2018. Till now, Department has succeeded to link 250 markets to the portal against the set target of 200 markets. To backup this project through facilitative legal framework & policy and revamp other reform agenda in the sector, Department is in the process of formulation of new Model APMC Act.

11.24 Only those States/ UTs are eligible to link their markets to e-NAM portal, which have undertaken reforms in their APMC Acts in respect of (i) e-trading; (ii) single point levy of market fee across the State; and (iii) single unified trading license valid across the State.

11.25 Status of progress of reforms with respect to 7 vital areas identified by the Department is given in the table below-

Table- Reform status as on 31/12/2016

Sl. No	Area of Reforms	States adopted the suggested area of market reforms
1.	Establishment of private market yards/ private markets managed by a person other than a market committee.	Andhra Pradesh, Arunachal Pradesh, Assam, Chhattisgarh, Gujarat, Goa, Himachal Pradesh, Karnataka, Maharashtra, Mizoram, Nagaland, Orissa (excluding for paddy / rice), Rajasthan, Sikkim, Telangana, Tripura, Punjab, UT of Chandigarh, Jharkhand, Uttarakhand, West Bengal.
2.	Establishment of direct purchase of agricultural produce from agriculturist (Direct Purchasing from producer)	Andhra Pradesh, Arunachal Pradesh, Assam, Chhattisgarh, Gujarat, Goa, Haryana (for specified crop through establishment of Collection Centres) Himachal Pradesh, Karnataka, Madhya Pradesh, Maharashtra, Mizoram, Nagaland, Rajasthan, Sikkim, Telangana, Tripura, Punjab (only in Rule), UT of Chandigarh (only in Rule),Jharkhand, Uttarakhand and West Bengal.
3.	Establishmentoffarmers/consumers market managed by a person other than a market committee (Direct sale by the producer)	Arunachal Pradesh, Assam, Chhattisgarh, Gujarat, Goa, Himachal Pradesh, Karnataka, Maharashtra, Mizoram, Nagaland, Rajasthan, Sikkim, Tripura, Jharkhand, Uttarakhand and West Bengal.
4.	Contract Farming Sponsor shall register himself with the Marketing Committee or with a prescribed officer in such a manner as may be prescribed.	Andhra Pradesh, Arunachal Pradesh, Assam, Chhattisgarh, Goa, Gujarat, Haryana Himachal Pradesh, Jharkhand, Karnataka, Maharashtra, Madhya Pradesh, Mizoram, Nagaland, Orissa, Punjab (separate Act), Rajasthan, Sikkim, Telangana, Tripura and Uttarakhand.
5.	To promote and permit e-trading	Andhra Pradesh, Chhattisgarh, Gujarat, Jharkhand , Haryana, H.P., Karnataka, Rajasthan, Sikkim, Goa, Madhya Pradesh, Maharashtra, Mizoram, Telangana, Uttarakhand and Uttar Pradesh .
6.	Single point levy of market fee across the State	Andhra Pradesh, Rajasthan, Gujarat, Goa, Haryana, Himachal Pradesh, Chhattisgarh, Jharkhand, Karnataka, Madhya Pradesh, Maharashtra, Mizoram, Nagaland, Sikkim, UT of Chandigarh, Punjab, Telangana , Uttar Pradesh and Uttarakhand
7.	Single trading license valid across the State	Andhra Pradesh, Chhattisgarh, Goa, Gujarat, Haryana, Himachal Pradesh, Karnataka, Rajasthan, Madhya Pradesh, Maharashtra, Mizoram, Nagaland, Telangana, Sikkim and Uttar Pradesh.

11.26 Deregulation of Marketing of Fruits & Vegetables

Fruits & vegetables being perishable crops need different set of markets and marketing system with reduced supply chain and adequate marketing infrastructures including cold storages & cold chain. Existing organised system of marketing through network of APMC markets used to be attributed for high post-harvest losses, long intermediation in supply chain and low return to the farmers. In order to address this issue, Department has been advocating with the States to provide complete freedom to the farmers as well as buyers and investors to invest in development of marketing infrastructure outside the market yard so as to facilitate emergence of various

channels of marketing. In addition to these innovative channels, APMC Market should also be available as one of the alternative choices to the farmers. Till now, 14 States (Madya Pradesh, Himachal Pradesh, Delhi, West Bengal, Odisha, Rajsthan, Chattisgarh, Gujrat, Maharashtra, Haryana, Karnatka, Assam, Nagaland, Meghalya) have moved in this direction, though in varied forms. Using the every platform, this Department during the year tried to convince the States to adopt ideal model suggested by this Department.

11.27 De-linking of Provisions of Compulsory Requirement of Shop/ Space for Registration of Traders / Market Functionaries-

Linking of provision of compulsory requirement of shop/ space for registration/ licensing to traders restricts entry of new traders (buyers) in the trade transaction, which in turn, reduces the competition among buyers resulting into offer of less competitive price to the farmers. In view of this, there should not be any such compulsory requirement of shop/ premises while granting the licence to the traders. As per information available from States, State-wise status is as under -

Provisions	Name of States where such provision existed
- For Traders	Uttrakhand, Arunachal Pradesh
- For Commission agents	Maharashtra, Andhra Pradesh, Haryana, Karnataka, Rajasthan, Telangana
- For Traders and Commission agents both	Chhattisgarh, J&K, Tamil Nadu

CCS National Institute of Agricultural Marketing (NIAM):

11.28 NIAM is an Institute of excellence set up by the Government of India in August 1988 with aim to enhance the efficiency and effectiveness of agricultural marketing systems that is inclusive and empowers the primary producer by building capacity of various stakeholders through teaching, training applied research, policy advocacy and consultancy services. The Institute since inception has been continuously engaged in cutting edge applied research and knowledge dissemination for strengthening the agricultural marketing system and activities to enable primary producers, particularly small and marginal farmers to enhance their income by realizing better price. In order to carry out the mandate of training, research, consultancy, education and policy advocacy, three centres have been formulated: (1) Centre for Design and Management of Agriculture Markets and Business Planning and Project formulation. (2) Centre for Entrepreneurship and Skilling (3) Centre for Information and Agri-value Chain Analysis.

11.29 In order to encourage desired changes at the ground, Institute has aligned its activities with the *Kisan Vikas Vriksha* of Ministry of Agriculture and Farmers Welfare, like promoting National Agricultural Market, development of integrated value chains mainly for horticulture and organic crops and developing marketing strategies particularly for NER States.

11.30 The Institute has been empanelled as National Level Agency by MIDH for developing marketing strategies for northeastern states namely Meghalaya, Assam and Tripura. Value chains of horticultural crops like Makhana, Tomato and Pea and leading medicanl crops are also being assessed with the support of agencies like MIDH, NABARD and National Medicinal Plant Board for enhancing farmers' participation in these value chains.

11.31 Aggregation through FPOs has been studied and a three month residential

certificate course has been designed for CEO's and other management of Farmer Producer Organizations (FPOs) by the Institute in association with Bankers Institute of Rural Development (BIRD), Lucknow to implement business plans and bring sustainability to their operations.

11.32 Farmer Business School is an initiative of the Institute to help farmers enhance their income called. The initiative is based on the concept developed by FAO and focuses on bringing a paradigm change in trainings primary producers mainly smallholders to enable them become efficient managers and entrepreneurs. In another initiate under Pradhan Mantri Fasal Bima Yojna, NIAM has developed a Board Game for farmers to make them aware on various aspects of the Scheme.

11.33 Institute has completed an assignment for developing 'Market Strategies for Organic Produce of Sikkim', wherein a comprehensive and feasible market plan has been suggested to yield better returns and enhance linkage of producers to high-end markets. Institute in its commitment to Northeastern regional has organized an exposer visit of market owners which aims at exposing community market owners from the state to various models of operation and management of markets of the country.

11.34 Warehousing and cold storage have become important components in development of integrated supply chain mainly for perishables. Accordingly NIAM has completed numerous consultancy assignments on warehousing like capacity building of officers of APSWC on operation and management of commercial warehouses, accreditation of Warehouses across the country, capacity building of warehousemen and Farmers Awareness Programme on negotiability of warehouse receipt. A training module on cold chain management is being developed by the Institute.

11.35 NIAM is evolving as a key organization in the field of agricultural marketing for south-east Asian and African countries. NIAM has been proposed to be the Nodal Resource Center for conducting trainings, workshop/conferences and consultancy for SAARC nations, FTF programme of USAID and members of Agriculture and Food Marketing Association for Asia Pacific (AFMA). The Institute is organizing a two-week training programme during 16-30 November 2016 on "Emerging trends in marketing of fresh fruits and vegetables" for participants from nine African countries under "Feed the Future" programme. An international conference on barrier free trade in agriculture for policy makers is being organized during the month of February 2016 for SAARC nation countries.

11.36 NIAM also offers two years PGDABM approved by the All India Council for Technical Education (AICTE). The programme with 100 percent placement is one of the most sought after programme by the Industry. The Institute is in the process of getting the programme accredited by NBA so that successful candidates may be awarded with Masters Degree and their acceptability get enhanced in Industry and academics.

Small Farmers' Agribusiness Consortium (SFAC):

11.37 SFAC was set up as a registered society on 18th January, 1994. Currently, the members of SFAC include RBI, SB1, IDB1, EX1M Bank, Oriental Bank of Commerce, NABARD, Canara Bank, NAFED etc. SFAC is implementing a Central Sector Scheme for Agribusiness Development through Venture Capital Assistance (VCA) to qualifying projects, which promotes linkages with farmers for procurement of their produce as raw material and provides employment in rural areas and has term-loan sanctioned by any Notified Financial Institution such as nationalized banks, SBI

and its subsidiaries, IDBI, SIDBI, NABARD, NCDC, NEDFI, Exim Bank, RRBs and State Financial Corporations. The mission of the Society is to support innovative ideas for generating income and employment in rural areas by promoting private investment in agri-business projects.

11.38 SFAC is implementing the Central Sector Scheme for agribusiness development in association with Notified Financial Institutions providing (i) Venture Capital to agribusiness projects, and (ii) assistance to farmers / products groups for preparing bankable Detailed Project Reports (DPR). During 2016-17 amounting to Rs. 29.12 crores upto 31.12.2016, SFAC achieved the physical target of 116 projects against an annual target of 200. SFAC will be able to surpass physical target as well as financial target during January-March, 2017. The projection or estimates for the period January –March, 2017 100 agri-business projects amounting to Rs. 23.00 Crores. Since the inception of the Scheme, DAC released an amount of Rs. 481.91 crore till 31.12.2016, against which SFAC has assisted 1463 agribusiness projects and sanctioned/ released Venture Capital Assistance of Rs. 433.78 Crores with total private and institutional investment of Rs. 5238.74 Crores. This has created 73317 directed employment and linked approximately 1.43 lakh farmers to these units for procurement of their produce.

11.39 Equity Grant and Credit Guarantee Fund Scheme for Farmer Producer Companies: SFAC is also taking up the implementation of the Equity Grant and Credit Guarantee Fund Scheme for Farmer Producer Companies which was approved by the EFC during 2013-14. This scheme will enable registered farmer producer companies to access equity grant to double member equity upto a maximum limit of Rs.10.00 lakhs. The scheme also provides a credit guarantee to financial institutions which extend loans to producer companies without collateral upto Rs.1.00 crore. An outlay of Rs.1 50.00 crores has been sanctioned during 2013-14 for the Scheme (Rs. 50.00 crore for Equity Grant and Rs. 100.00 crores for Credit Guarantee Scheme). This is likely to benefit hundreds of newly formed producer companies across the country to increase their equity capital and leverage institutional borrowing from banks. SFAC has sanctioned so far 61 projects amounting to Rs. 396.80 lakh under Equity Grant Fund.

11.40 SFAC is organizing awareness camps to publicize the Equity Grant and Credit Guarantee Fund Scheme all over India. 13 Banks have signed MoU with SFAC under Credit Guarantee Fund Scheme for FPCs. (Canara Bank, State Bank of India, NABARD, IDBI, NABARD Financial Services, PNB, Indian Bank, Bank of India, NABKISAN Finance Ltd., RBL Bank, HDFC Bank, Yes Bank & Sarva Haryana Gramin Bank).

11.41 Credit Guarantee Fund Scheme for Farmers Producer Companies - Credit Guarantee Fund Scheme is a new Central Sector Scheme, The Credit Guarantee Fund has been set up with the primary objective of providing a Credit Guarantee Cover to Eligible Lending Institutes (ELIs) providing loans to Farmer Producer Companies (FPCs). Under this scheme FPCs are will be given collateral free credit by ELIs. The CGF shall be operated by Small Farmers' Agri Business Consortium (SFAC) through lending institutions. The amount has been kept as FDRs as per the guidelines of the Scheme. As per the EFC, the expenses of the scheme will be met out from the interest earned on these FDRs. The FDRs will continue till the schemes will be implemented by SFAC, thereafter the amount will be spent as per the Government direction.

SFAC has sanctioned Credit Guarantee cover in favour of 16 banks amounting 684.76 lakhs under the Credit Guarantee Fund Scheme.

State-wise sanctioned project-Credit Guarantee fund scheme from 2014-15 to 2016-17

SI. No.	Bank-wise	No. of cases	C.G. cover sanctioned (Rs. in lakhs)
1.	Haryana	I	42.50
2.	Rajasthan	2	39.10
3.	Madhya Pradesh	7	382.36
4.	Tamil Nadu	6	220.80
	**Total**	**16**	**684.76**

Bank-wise sanctioned project-Credit Guarantee fund scheme from 2014-15 to 2016-17

Sl. No.	Bank-wise	No. of cases	C.G. cover sanctioned (Rs. in lakhs)
1.	IDBI Bank Limited	4	261.66
2.	NABARD	4	152.80
3.	State Bank of India	2	62.90
4.	Canara Bank	2	63.75
5.	Yes Bank	1	21.25
6.	NABKISAN Finance Ltd	1	37.40
7.	Indian Bank	2	85.00
	**Total**	**16**	**684.76**

The projection or estimates for the period January–March, 2017: SFAC will be able to sanction Credit Guarantee Cover to 10 banks under Credit Guarantee Fund Scheme amounting to Rs. 3.00 Crores.

11.42 Farmers Producer Organizations - SFAC was mandated by Department of Agriculture, Cooperation and Farmers Welfare, Ministry of Agriculture and Farmers Welfare, Govt. of India, to support the State Governments in the formation of Farmer Producer Organizations (FP0s). The initiative which started in 2011-12 under the two Central Sector Schemes for Vegetable Initiative for Urban Clusters (VIUC) and Integrated Development of 60,000 Pulse Villages in Rainfed Areas has expanded in its scope and covers special FPO projects being taken up by some State Governments under general RKVY funds as well as under the National Demonstration Project under the National Food Security Mission (NFSM) and Mission for Integrated Development of Horticulture (MIDH).

11.43 As on 31st December, 2016, against a target of 7.01 lakh farmers to be mobilized 5.83 lakh farmers have been identified and formed into 34,345 Farmer Interest Groups (FIGs). These FIGs further have been federated into FPOs and so far 569 FPOs have been registered and 131 are under the process of registration.

11.44 National Aericulture Market (NAM):- Ministry of Agriculture, Department of Agriculture, Cooperation & Farmers Welfare (DAC&FW) has mandated Small Farmers' Agribusiness Consortium (SFAC) to act as the Lead Promoter of NAM. Under the scheme, a pan India electronic trading portal (e-NAM) is being deployed in selected regulated wholesale markets in States across the country and 585 markets are proposed to be integrated with e-NAM by March, 2018. E-NAM is reformed linked scheme and States are required to carry out pre-requisite reforms to enable (i) a Single licence to be valid across the State, (ii) Single point levy of market fee, and (iii) Provision for electronic auction as a mode of price discovery.

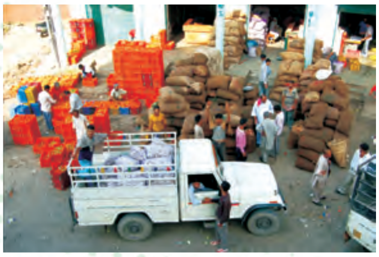

Chapter 12

Agricultural Cooperation

Amendment to the Constitution in respect of Cooperatives:

12.1 For the purpose of ensuring the democratic, autonomous and professional functioning of cooperatives, it was decided to amend the Constitution of India. Accordingly, the Constitution (One Hundred and Eleventh Amendment) Bill, 2009 was introduced in Lok Sabha on 20.11.2009.The Bill was passed in Lok Sabha on 21.12.2011 and in Rajya Sabha on 28.12.2011 as "The Constitution (Ninety Seventh Amendment) Act, 2011" and Hon'ble President of India gave her assent to the aforesaid Act on 12.01.2012. The Act came into force w.e.f. 15.02.2012 vide Gazette Notification dated 08.02.2012.

12.2 The amendment in the Constitution, inter alia, seeks to empower the Parliament in respect of multi-State Cooperative Societies and the State Legislatures in case of registered under the Sate co-operative societies Act to make appropriate law, laying down the following matters:

- Right to form cooperative societies as a Fundamental Right by insertion of the words "cooperative societies" in sub clause (c) of clause (1) of Article 19.
- Provisions for incorporation, regulation and winding up of co-operative Societies based on the principles of democratic member control, member economic participation and autonomous functioning.
- Insertion of Article 43B in part IV of the Constitution as Directive Principle of State Policy for Voluntary formation of cooperative societies.
- Specifying the maximum number of director of a co-operative society to be not exceeding twenty-one members.
- Providing for a fixed term of five years from the date of election in respect of the elected members of the board and its office bearers; and formation of an authority or body for the conduct of elections to a cooperative society.
- Providing for a maximum time limit of six months during which a board of Directors of co-operative society could be kept under supersession or suspension.
- Providing for independent professional audit.
- Providing for right of information to the members of the co-operative societies.
- Empowering the State Governments to obtain periodic reports of activities and accounts of co-operative societies.
- Providing the reservation of one seat for the Scheduled Castes or the Scheduled Tribes and two seats for women on the board of every cooperative society, which have individuals as members from such categories.
- Providing for offences relating to cooperative societies and penalties in respect of such offences.

12.3 Amending the State Cooperative Societies Acts in tune with the provisions of the above amendments in the Constitution will not only ensure autonomous and democratic functioning of the cooperatives, but also ensure accountability of management to the members & other stakeholders and also enhance public faith in these institutions.

The Constitutional amendment provides for a maximum period of one year from the date of its commencement to amend the state laws relating to cooperative societies, if required, to make them consistent with the provisions of the amendment. So far 17 States, viz. Arunachal Pradesh, Assam, Bihar, Chhattisgarh, Gujarat, Haryana, Karnataka, Kerala, Madhya Pradesh, Mizoram, Odisha, Rajasthan, Tripura, Uttar Pradesh and West Bengal ,Tamil Nadu and Maharashtra have amended their State Cooperative Societies Acts in consonance with the constitution(97th Amendment) Act, 2011. However, in the meantime certain provisions of the Constitution (97th Amendment) Act, 2011 have been struck down by the Hon'ble High Court of Gujarat at Ahmedabad vide order dated 22.4.2013 in WP (PIL) No.166 of 2012. The union of India has filed SLP No. 25266-25267 on 12.7.2013 before the Hon'ble Supreme Court against the aforesaid order and the case is pending before the Supreme Court for its disposal.

12.4 National Cooperative Development Corporation (NCDC):

(i) Assistance to NCDC Programmes for Development of Cooperatives: The Department of Agriculture, Cooperation & Farmers Welfare is implementing cooperative programmes through National Cooperative Development Corporation (NCDC). The Central Sector Integrated Scheme on Agricultural Cooperation (CSISAC), for the remaining period of the 12th Five Year Plan (2013-2017) was approved and commenced its implementations during the year 2014-15. The CSISAC is the result of merger of two erstwhile schemes of 11th Five Year Plan i.e Restructured Central Sector Scheme for Assistance to National Cooperative Development Corporation (NCDC) Programmes for Development of Cooperatives and Central Sector Scheme for Cooperative Education & Training. The programmes/ schemes being implemented through NCDC are (i) Assistance for Marketing, Processing, Storage etc. programmes in the Cooperatively Least & Under developed States & for weaker section programmes in developed States; (ii) Assistance for cotton development including ginning and pressing and establishment of new cooperative spinning mills and modernization / expansion/ rehabilitation of existing cooperative spinning mills; and (iii) Integrated Cooperative Development Projects in selected districts (ICDP). While the loan component (both Term Loan & Investment Loan) is provided by NCDC from its own resources, subsidy is provided by Government of India under the CSISAC Schemes.

12.5 NCDC is a statutory corporation set by an Act of Parliament. It is a non-equity development financing institution for the cooperative sector in the country and provides assistance for economic development of agriculture & rural sector through cooperative societies. The Corporation's focus is on programmes of agricultural inputs, processing, storage and marketing of agriculture produce and supply of consumer goods in rural areas. In the non-farm sector, the Corporation's endeavour is to equip cooperatives with facilities to promote income generating activities, with special focus on weaker sections such as handlooms, sericulture, poultry, fishery, scheduled caste & scheduled tribe cooperatives etc. It also provides assistance for capacity building and upgradation of skills of personnel involved in the cooperatives.

12.6 Against the approved outlay of Rs.114.50 crore subsidy under CSISAC Scheme for 2016-17, DAC&FW has provided subsidy of ₹106.65 crore. Of this, ₹78.69 crore, received in June, 2016 was utilised immediately and out of ₹29.96 crore received lately in December 2016, ₹7.88 crore has been

utilised. The remaining subsidy amount of ₹20.08 crore available with NCDC is under process for disbursement.

12.7 Besides the above, the scheme NCDC has dovetailed its programmes with schemes of Government of India e.g. (i) Agricultural Marketing Infrastructure (AMI) a sub-scheme of Integrated Scheme for Agricultural Marketing (ISAM) for Storage & Infrastructure; (ii) Rashtriya Krishi Vikas Yojana (Training) (iii) Mission for Integrated Development of Horticulture (MIDH); (iv) Scheme for Cold Chain, Value Addition and Preservation Infrastructure; (v) Interest rebate under Technology Up gradation Fund - Ministry of Textile; (vi) Sugar Development Fund (vii) Assistance for Boosting Seed Production component under Sub-Mission for Seed and Planting Material (SMSP) of National Mission on Agricultural Extension and Technology (NMAET).

12.8 Against the approved annual action plan outlay of ₹6,000.00 crore for 2016-17, NCDChas disbursed ₹7883.27 crore (till 15.12.2016) for various activities as under:

(₹ in crore)

Sl. No.	Activity	Disbursements upto 15.12.2016
1	Marketing & Inputs	5035.31
2	Agro-Processing (Sugar, Textile & other processing units)	1424.29
3	Storage & Cold Storage	45.51
4	Weaker Section Programme (Fisheries/ Dairy & Livestock/ Poultry/ Handloom/ SC & ST/ Women & Labour Cooperatives)	297.51
5	Computerisation of Cooperatives	3.41
6	Consumer Cooperatives	3.88
7	Integrated Cooperative Development Projects (ICDP)	172.27
8	Industrial & Service	900.95
9	Promotional & Development	0.14
	**Grand Total (1 to 9)**	**7883.27**

12.9 During the year 2016-17, (upto 15.12.2016), NCDC has sanctioned assistance of ₹7542.54 crore for marketing & input activities. Major portion of the sanctioned assistance i.e. ₹6470.00 crore is in the form of short term working capital to Chhattisgarh State Coop. Marketing Federation (₹5420 crore), NAFED (₹300 crore), West Bengal State Cooperative Marketing Federation (₹300 crore), and Andhra Pradesh Cooperative Oil Seeds growers' Federation (₹450 crore) for procurement of Paddy, Ground Nut Pod, Sunflower Seeds, Moong, Copra under Price support operations.

12.10 Out of ₹5035.31 crore disbursed during the same period, ₹3500 crore was exclusively for Price Support Operations. These have a long way in ensuring smooth procurement operations from farmers. The timely assistance from NCDC has helped prevent distress sale by more than 15 lakh farmers in these states.

(ii) Cooperative Sugar Mills:

12.11 The number of installed cooperative sugar factories increased from 2 in 1950-51 to 327 in 2015-16. The installed sugar production capacity of cooperative sector was ₹128.59 lakh MT at the end of year 2015-16 accounting for about 39% of the total production capacity of ₹332.31 lakh MT capacity in the country. During 2016-

17, (as on 15.12.2016), NCDC sanctioned and disbursed ₹1824.27crore and ₹1146.17 crore respectively benefitting 5,32,930 sugarcane farmer members and is likely to further sanction and release additional amount of ₹200.00 crores and 550.00 crores respectively, by 31.03.2017. During the year under review, 13 sugar cooperative societies have been further brought under the fold of NCDC funding. The financial assistance for establishment of a new sugar mill in Navsari district, Gujarat has helped the sugarcane grower farmers, especially the pre-dominant population of tribal farmers in the district. NCDC's financial assistance for zero liquid discharge mechanism (ZLD) has also been instrumental in resuscitating 7 distilleries of Uttar Pradesh Cooperative sugar mills as they were on the verge of closure due to non confirmatory Pollution norms. In addition, assistance has been sanctioned for modernisation cum expansion (3 mills), establishment of Cogeneration plant for green energy (2 units) and an Ethanol plant.

(iii) Cooperative Spinning Mills:

12.12 In order to improve economic condition of the cotton growers as well as handloom & power loom weavers and to consolidate the gains achieved so far, the DAC & FW, through NCDC, continued to provide financial assistance to the spinning mills & ginning and pressing units in the cooperative sector. During the year 2016-17, (as on 15.12.2016), NCDC has so far sanctioned ₹329.16 crore (comprising loan of ₹289.62 crore and subsidy of ₹39.54 crore under CSISAC scheme) to 11 Cooperative Ginning & Spinning Mills for modernisation of 12480 ring spindles, expansion of 14112 spindles, installation of 66048 ring spindles & 48 Double Roller Gins and also for meeting working capital requirements. This will benefit 55325 members including 50394 cotton growers and 3998 power loom weavers. NCDC has so far disbursed ₹203.01 crore (comprising loan of ₹194.98 crore and subsidy of ₹8.03 crore under CSISAC scheme). Of this, ₹2.38 crore would benefit SC/ST members. During 2016-17, (as on 15.12.2016), NCDC sanctioned assistance of ₹22.23 crore (including subsidy of ₹4.70 crore) for other processing units (Oilseed, Plantation crop & Foodgrains). Of this assistance, ₹14.87 crore has been sanctioned for expansion of the Palm Oil processing unit of Telangana State Cooperative Oilseeds Federation and working capital loan of `50.00 crore to Andhra Pradesh Oilseeds Federation for procurement of Oilseeds. Further, an amount of ₹78.02 crore has been disbursed for the above mentioned other processing units.

(iv) Cooperative Storage and Cold Storage:

12.13 DAC&FW through NCDC has been making systematic and sustained efforts to assist cooperatives in creating additional storage capacity aimed at facilitating expanded operations of cooperative marketing of agriculture produce, distribution of inputs and sale of consumer articles. Storage capacity assisted by NCDC stood at 160.07 lakh MT as on 31.03.2016. During the year 2016-17, (as on 15.12.2016), financial assistance of `30.93 crore was sanctioned and `40.93 crore was disbursed for storage programmes. In addition, ₹4.57 crore was released for cold storage programmes.

(v) Weaker Section Programmes:

12.14 Towards promoting weaker sections, NCDC is assisting different types of projects for cooperative societies involved in fishery, poultry, dairy, livestock, handloom, coir, jute, sericulture and also activities taken up cooperative societies comprising of Scheduled Castes, Scheduled Tribes, labour & women members. During 2016-17, (as on 15.12.2016), an amount of ₹464.78 crore

was sanctioned for various weaker section programme. This included (i) ₹275.00 crore comprising loan of ₹165.00 crore and CSISAC subsidy of ₹55.00 crore to Government of Andhra Pradesh for development of Sheep and Goat Cooperatives in 11 districts of the State and would benefit 2835 SC and 1950 ST members; (ii) an assistance of ₹174.33 crore for Dairy Cooperatives in Gujarat, Maharashtra, Punjab and Rajasthan is likely to benefit producer members of whom 77462 are SC, 180237 ST and 202397 are women members; (iii) ₹12.52 crore for fisheries programme benefiting 4472 members majority of which belong to SC & ST; and (iv) ₹2.93 crore for 3 handloom programmes in benefiting 913 weaver members. During the same period, an amount of ₹297.51 crore was disbursed for various weaker section programme.

(vi) Integrated Cooperative Development Projects (ICDP):

12.15 NCDC is implementing Integrated Cooperative Development Project (ICDP) in selected districts. During the year 2016-17 (as on 15.12.2016), NCDC has disbursed loan assistance of ₹121.91 crore and subsidy of ₹50.36 crore, totaling to ₹172.27 crore. The subsidy of ₹50.36 crores includes ₹9.89 crore towards Manpower Development and Training, managerial assistance of Project Implementing Agency and Monitoring Cell. Further, 6 projects in Uttar Pradesh with a project cost of ₹156.60 crore are being sanctioned, which involve NCDC share of assistance of ₹148.26 crore (₹119.65 crore as loan and ₹28.61 crore as subsidy).

(vii) Service Cooperatives:

12.16 During the year 2016-17, (as on 15.12.2016), NCDC has sanctioned and released ₹1537.65 crore and ₹900.94 crore respectively to District Central Cooperative Banks and other cooperatives in the States of Madhya Pradesh, Kerala, Andhra Pradesh, Karnataka and Rajasthan to supplement their resources for providing crop loans to farmers and agriculture and allied activities.

(viii) Assistance to Cooperatives in Cooperatively Least & Under Developed States:

12.17 In order to reduce the regional disparities, NCDC funds programmes in various States which are categorised as under:

- Cooperatively Least Developed States / Union Territories. {Arunachal Pradesh, Assam, Bihar, Jharkhand, Jammu & Kashmir, Manipur, Meghalaya, Mizoram, Nagaland, Sikkim, and Tripura};
- Cooperatively Under Developed States/ Union Territories. {Andhra Pradesh, Chhattisgarh, Goa, Himachal Pradesh, Madhya Pradesh, Odisha, Rajasthan, Telangana, Uttar Pradesh, Uttarakhand, West Bengal, Andaman & Nicobar Islands (UT) and Lakshadweep (UT)};
- Cooperatively Developed States/ Union Territories. {Gujarat, Haryana, Karnataka, Kerala, Maharashtra, Punjab, Tamilnadu, Chandigarh (UT), Dadra & Nagar Haveli (UT), Daman & Diu (UT), Puducherry (UT), Delhi }

12.18 The Cooperatives in these categories are eligible for different slabs of subsidies available under the CSISAC Scheme. During the year 2016-17 (as on 15.12.2015), financial assistance of ₹6844.38 crore & ₹4549.10 crore has been sanctioned and disbursed respectively by the NCDC to cooperatives in cooperatively least/under-developed States/ UTs respectively. The disbursements in the UD/LD States account for 57.71% of total releases of ₹7883.27 crore by NCDC during 2016-17 (upto 15.12.2016).

(ix) Development of Women Cooperatives:

12.19 NCDC encourages women cooperatives to avail assistance under its various schemes. A large number of women members are engaged & involved in cooperatives dealing with activities related to Foodgrain Processing, Plantation Crops, Oilseed Processing, Fisheries, Dairy & Livestock, Spinning Mills, Handloom & Powerloom Weaving and under Integrated Cooperative Development Projects etc. Women Cooperatives are now covered under Weaker Section Programmes for the purpose of availing subsidy and concessional funding during the remaining period of the 12th Five Year Plan programme under Central Sector Integrated Scheme on Agricultural Cooperation.

(x) Efforts towards "Doubling Farmers Income":

12.20 Towards doubling the farmers' income, NCDC has sanctioned 4 pilot projects on organic farming - one each in the districts of Jhalawar (Rajasthan) and Ranchi (Jharkhand) and two in Varanasi (Uttar Pradesh). The total cost involved, spread over a period of three years, is estimated at `1.02 crore which would benefit around 700 farmers and their families located in 26 clusters.

(xi) ISO Certification:

12.21 NCDC, an ISO 9001:2008 compliant organization has upgraded its Quality Management System in accordance with the new standards and has been certified ISO 9001:2015 w.e.f. 10.12.2016 reflecting the organization's commitment for continual improvement and better service delivery to its clients.

Assistance for Cooperative Education and Training:

12.22 The Government of India has been implementing a Central Sector Scheme for Cooperative Education and training through the National Cooperative Union of India (NCUI) and National Council for Cooperative Training (NCCT) since the 3rd Five Year Plan as a continuing scheme. From the 12th Five Year plan, the scheme of "Cooperative Education and Training" and "Ässistance to NCDC for development of Cooperatives" has been merged into a single scheme namely "İntegrated Scheme on Agricultural Cooperation". As per the new scheme upto 50% of the expenditure is given as grant-in-aid to NCUI by Government of India for Cooperative Education in cooperatively under-developed states/ under-developed areas of developed states (UDS) for approved activities. The balance portion is met by NCUI from the Cooperative Education Fund created under MSCS Act, 2002 and own sources. Government of India through NCUI also grants 50% assistance to Junior Cooperative Training Centres' which are run by the State Cooperative Unions/ State Governments. At present, NCUI is running 43 Cooperative Education Field projects spread over 22 States/UTs.

12.23 During the year 2015-16, NCUI Cooperative Education Field Projects including north-east and women Projects conducted wide ranging programmes on various issues of cooperative development. Under UDS projects, new societies registered during the year were 26 and the number of persons benefitting from income generating programmes were 8317. In northern states, 320 SHGs were formed with the membership of 3135 by the project personnel. NCUI organized 24599 events and imparted education to 299231 persons during the year 2015-16. Under approved activities, the National Centre for Cooperative Education (NCCE), organized 89 training programmes against the target of 60 programmes during the year including 12 weeks Diploma programme in Cooperative Education and Development. 3698 persons from various

sectors of the cooperative movement in different states were trained. Out of 109 training prgorammes, 38 programmes were organized for weaker sectors like labour, fisheries and tribal cooperatives and exclusively for SC/ST cooperative in which 1398 persons participated. 20 programmes were organized exclusively for women cooperatives which were attended by 708 participants. During the year 2015-16, an amount of Rs. 528.52 Lakhs was released as grants-in-aid to NCUI including Rs. 88.68 Lakhs for NE regions.

12.24 The Cooperative Training programmes are being conducted by the National Council for Cooperative Training (NCCT) through its 5 Regional Institutes of Cooperative Management, 14 Institutes of Cooperative Management located in different States and Vaikunth Mehta National Institute of Cooperative Management, Pune. NCCT which had been received 100% grants-in-aid till the 12th Plan is to meet its requirement from the interest income of Corpus Fund (the fund which was created during the 10th Plan by equal contribution of Rs. 100 by GOI and cooperative movement) and the Government shall meet the gap in the resources as per the budget estimates for the concerned year. An amount of Rs. 548.25 was released to NCCT for VAMNICOM component. Besides, during the financial year 2015-16, a sum of Rs. 1430.16 lakhs was released to NCCT towards gap in resources for the year 2013-14 and 2014-15.

12.25 During the year 2015-16 the National Council for Cooperative Training (NCCT) has conducted 2170 number of training programmes and trained 70687 participants against the target to conduct 1600 number of training programmes and to train 40,000 participants which includes 17449 participants from SC/ST categories.

12.26 NE Region : The Government of India is providing separate assistance for intensification of cooperative education in the north-eastern region through NCUI. NCUI has established 7 field projects namely Aizawl (Mizoram), Thoubal (Manipur), Mangalwaria(Sikkim), Shillong(Meghalaya), Kohima(Nagaland), Morigaon and Jorhat (Assam) in the North Eastern Region.

Price support scheme (PSS)

12.27 The Department of Agriculture, Cooperation and Farmers Welfare implements Price Support Scheme (PSS) for procurement of oil seeds, pulses and cotton through Central Nodal Agencies at the Minimum Support Price (MSP) declared by the Government. This scheme is implemented at the request of the concerned state government which agrees to exempt the procured commodities from levy of mandi tax and assist central nodal agencies in logistic arrangements including gunny bags, provide working capital for state agencies, creation of revolving fund for PSS operations etc. as required under the Scheme guidelines. The basic objectives of PSS are to provide remunerative prices to the growers for their produce with a view to encourage higher investment and production and to safeguard the interest of consumers by making available supplies at reasonable prices with low cost of intermediation.

12.28 Achievement under Price Support Scheme (PSS) : During the season 2015-16, the prices of various oilseeds and pulses ruled below respective MSP fixed for the relevant marketing season. During the year 2015–16 and 2016-17, NAFED procured agricultural commodities as per the details given below-

Procurement made by NAFED (as on 09.01.2017)

Sl. No.	Year/Season	Commodity	Procured quantity (in MTs)	Minimum Support Price (MSP) for FAQ (in Rs. Per MTs)	Procurement value (in lack Rs.)
1	SUNFLOWER SEED RABI 2016	ODISHA	164.56	38,000.00	62.53
		HARYANA	4784.708		1818.21
		**TOTAL**	**4949.268**		**1880.74**
2	Milling Copra 2016	TAMIL NADU	1170.443	59500.00	696.41
		ANDHRA PRADESH	3296.75		1961.57
		**TOTAL**	**4467.193**		2657.984
3	Moong K-2016	KARNATAKA	1837.30	52250.00	959.99
		MAHARASHTRA	6430.28		3359.82
		**TOTAL**	**8267.58**		**4319.81**
4	Groundnut K-2016	GUJARAT	146870.03	42200	61979.15
5	Soyabean K-2016	MAHARASHTRA	163.81	27750	45.46

Procurement made by SFAC

Sl. No	Year/Season	Commodity	Procured quantity (in MTs)	Minimum Support Price (MSP) for FAQ (in Rs. Per MTs)	Procurement value (in lack Rs.)
1	Moong K-2016	KARNATAKA	679.90	52,250.00	355.25
		MAHARASHTRA	548.00		286.35
		**TOTAL**	**1227.90**		**641.6**

12.29 Market Intervention Scheme (MIS)

The Department of Agriculture, Cooperation and Farmers Welfare implements the MIS for procurement of agricultural and horticultural commodities which are perishable in nature and are not covered under the PSS. The objective of intervention is to protect the growers of these commodities from making distress sale in the event of a bumper crop during the peak arrival period when the prices tend to fall below economic levels and cost of production. The condition is that there should be either at least a 10 percent increase in production or a 10 percent decrease in the ruling market prices over the previous normal year. The scheme is implemented at the request of a State/UT government which is ready to bear 50 percent of the loss (25 percent in case of North-Eastern States), if any, incurred on its implementation. The extent of total amount of loss to be shared on a 50:50 basis between the Central Government and the State Government is restricted to 25 percent of the total procurement value which includes cost of the commodity procured plus permitted overhead expenses. Under the scheme, in accordance with MIS guidelines, a pre-determined quantity at the fixed Market

Intervention Price (MIP) is procured by the agencies designated by the state government for a fixed period or till the prices are stabilized above the MIP whichever is earlier. The area of operation is restricted to the concerned state only. The details of MIS implemented during the year 2015-16 and 2016-17 as on 31.12.2016.

Sl. No.	Year	Commodity	MIP (Rs. Per MTs)	State	Sanctioned Qty. (in MTs)
1	2015-16<br>20.05.2015 to 20.06.2015	Potato	4,250	Uttar Pradesh	1,00,000
2	15.12.2015 to 14.03.2016	Oil Palm FFB	7,888/-	Andhra Pradesh	1,14,963
3	01.09.2016 to 30.09.2016	Grapes	42,000/-	Mizoram	3,800
4	01.11.2016 to 30.11.2016	Onion	6240/-	Karnataka	1,00,000
5	2016-17<br>01.12.2016 to 30.12.2016	Onion	7,070/-	Telangana	5,000
6	2016-17<br>14.12.2016 to 12.01.2017	Palm-Oil	7,650/-	Tamil Nadu	1,000

******

Chapter 13

Rashtriya Krishi Vikas Yojana (RKVY)

13.1 Background: National Development Council (NDC), in its meeting held on 29th May, 2007 resolved that a special Additional Central Assistance (ACA) Scheme be introduced to incentivize States to draw up comprehensive agriculture development plans, taking into account agro-climatic conditions, natural resources and technology for ensuring more inclusive and integrated development of agriculture and allied sectors. Accordingly, Department of Agriculture & Cooperation (DAC), Ministry of Agriculture, in consultation with the Planning Commission, launched Rashtriya Krishi Vikas Yojana (RKVY) in 2007-2008.

13.2 During XI Plan, Rs. 22,408.77 crore was released for implementation of the scheme in the States & Union Territories (UTs) of which Rs. 22,332.97 crore was utilized in implementing projects relating to crop development, horticulture, agricultural mechanization, natural resource management, marketing & post-harvest management, animal husbandry, dairy development, fisheries, extension etc. During XII Five Year Plan, an outlay of Rs. 63,246 crore has been earmarked for implementing RKVY, out of which Rs. 8400 crore, Rs. 7052.51crore, Rs. 8443.20 crore and Rs.3942.38 crore had been released under the scheme during 2012-13, 2013-14, 2014-15 and 2015-16 respectively.

13.3 In sync with Ministry of Finance directions, funding pattern of the scheme from 2015-16 has been shared between centre and states in the ratio of 60:40 (90:10 for 8 North-Eastern and 3 Himalayan States) against 100% funding under RKVY by Central Government till the end of financial year 2014-15. The allocation provided for implementation of the scheme during 2016-17 which was Rs. 5400 crore at BE stage has been reduced to Rs.3800 crore in RE.

13.4 Based on the feedback received from States, experiences garnered during implementation in 11th Plan and inputs provided by Stakeholders, Operational Guidelines of RKVY have been revised for implementation from 2014-15 in order to enhance efficiency and efficacy of the programme and its inclusiveness during 12th Plan period. The revised Operational Guidelines of the scheme mandated that at least 35% of Normal RKVY allocation should be utilized by the States for implementing Infrastructure and Assets development projects.

13.5 Objectives: The main objectives of RKVY Scheme are as under:

i. To incentivize the States so as to increase public investment in agriculture and allied sectors.

ii. To provide flexibility and autonomy to States in the process of planning and executing agriculture and allied sector schemes.

iii. To ensure the preparation of Agriculture Plans for the districts and the States based on agro-climatic conditions, availability of technology and natural resources.

iv. To ensure that the local needs/crops/ priorities are better reflected in the agricultural plans of the States.

v. To achieve the goal of reducing the yield gaps in important crops through focused interventions.

vi. To maximize returns to the farmers in agriculture and allied sectors;

vii. To bring about quantifiable changes in the production and productivity of various components of agriculture and allied sectors by addressing them in a holistic manner.

13.6 Implementation Strategy: RKVY accords flexibility and autonomy to States in planning and executing projects related to agriculture and allied sectors. States are empowered to formulate strategies for development of the agriculture and allied sectors in a holistic way taking into account their agro-climatic conditions so as to effectively address their local needs and priorities, rather than continuing with business as usual mode of "one size fits all" formula. Projects under RKVY cover the entire gamut of activities in the agriculture and allied sectors supplementing ongoing Centrally Sponsored Schemes in these sectors. RKVY also emphasizes on convergence through District Agriculture Plans (DAPs) and State Agriculture Plan (SAP) for eliminating overlap of resources & efforts for optimal utilization of funds available through various schemes. The requirements of the Gram Panchayats selected under Saansad Adarsh Gram Yojana (SAGY) would be kept in mind by SLSC while sanctioning projects.

13.7 Besides planning and execution of projects, RKVY provides complete flexibility and autonomy to States to customize interventions as per local requirements. For a vast country like India with diverse soil & agro-climatic conditions, varying technological capabilities and agronomic practices, RKVY framework has enabled States to plan and chart their own developmental trajectory. At national level, it enables the country to achieve the desired annual growth rate in agriculture and allied sectors.

13.8 Over the years, RKVY has also facilitated in addressing national priorities, without affecting the autonomy and flexibility of States, through special programmes as sub-schemes. Towards this endeavour, sub-schemes such as Bringing Green Revolution to Eastern India (BGREI), Crop Diversification Programme (CDP), Reclamation of Problem Soil, Foot and Mouth Diseases Control Programme, Rice Fallow Areas in Eastern India for Pulses and Oil Seeds etc are being implemented during 2016-17.

13.9 Outcome: One of the basic objectives of RKVY is to incentivize investments in agriculture and allied sectors by linking State wise allocation of RKVY funds to the increased share of State Plan Expenditure in agriculture and allied sectors. This has helped in stepping up allocation to agriculture and allied sectors as a percentage of total State Plan Expenditure from 4.88% (Rs 8,770 crore) in 2006-07 to 8.1% (Rs.75012.20 crore) during 2014-15. Leveraging higher investments, States have been able to enhance production and productivity of crops and animal husbandry sector. During XI Plan, States have taken up over 5700 projects across the entire spectrum of agriculture & allied sectors such as crop development, horticulture, agricultural mechanization, marketing & post-harvest management, animal husbandry, dairy development, fisheries, extension, etc. Concerted efforts by Centre and States has helped Agriculture and allied sector to achieve an annual growth rate of 4.1%(at 2004-05 prices) during the XI Plan against 2.46% per annum during X Plan period.

13.10 Allocation of Funds to States: RKVY is administered by the Union Ministry of Agriculture & Farmers Welfare. Funds under this scheme are provided to the States as grant-in-aid by the Central Government.

13.11 Eligibility Criteria for Access to RKVY funds: Following two eligibility conditions

are stipulated for States to become and remain eligible for receiving assistance under RKVY:

a) The base line share of agriculture and allied sectors in its total State Plan (excluding RKVY funds) expenditure is at least maintained; and

b) District Agriculture Plans (DAPs) and State Agriculture Plans (SAPs) have been formulated.

13.12 Criteria for Allocation of Funds to States: Allocation of RKVY fund to each of the eligible States is based on the six parameters as per formula given below:

Sl. No.	Criteria/Parameter	Weightage
1.	Percentage share of net un irrigated area in a state to the net un irrigated area of all eligible States.	15%
2.	Last three (3) years average area under oil seeds and pulses.	5%
3.	State's highest GSDP for agriculture and allied sectors for the past five years.	30%
4.	Increase in expenditure in Agriculture and allied sectors in the previous year over the year prior to that year. (For example, previous year for allocating State's share for 2014-15 would be the year 2012-13 and the year prior to that would be 2011-12).	30%
5.	Increase in Plan and non-plan expenditure made by the States from the State Budgets on Animal Husbandry, Fisheries, and Agricultural Research & Education in the previous years over the year prior to that year.	10%
6.	Inverse of Yield gap between state average yield and potential yields as indicated in the frontline demonstration data.	10%

13.13 Streams of Funding in RKVY: RKVY funds would be provided to the States as grant by the Central Government in following streams.

(a) RKVY (Production Growth) with 35% of annual outlay,

(b) RKVY (Infrastructure and Assets) with 35% of annual outlay;

(c) RKVY (Special Schemes) with 20% of annual outlay; and

(d) RKVY (Flexi Fund) with 10% of annual outlay (States can undertake either production Growth or Infrastructure & Assets projects with this allocation depending upon State specific needs/ priorities).

13.14 The requirement of minimum allocation of RKVY fund (35% at Central Level or 43.75% at State Level) to "Production Growth Stream" has been waived off subsequently. Thus, State can allocate their entire RKVY Normal allocation to Infrastructure &Assets stream with effect from financial year 2014-15.

13.15 Further all Government (Centre/ State) and quasi- Govt. agencies (fully funded organizations, State Agriculture Universities, Panchayati Raj Institutions, Primary Agriculture Credit Societies (PACS)) etc. may be entitled to 100% funding of project cost under RKVY for creation of assets / infrastructure to promote activities in agriculture and allied sectors against capping of subsidy to 25% of the project cost in the case of non state actors.

13.16 State Level Sanctioning Committee (SLSC): State Level Sanctioning Committees (SLSCs) have been constituted under the Chairmanship of Chief Secretary with Secretary (Agriculture) of the concerned State/UT as Member Secretary along with other members from allied departments & State Agriculture Universities, Department of Agriculture, Cooperation & Farmers Welfare (GOI), Department of Animal Husbandry, Dairying & Fisheries (GOI), and NITI Aayog (erstwhile Planning Commission). SLSC appraises and approves the projects under

RKVY. State Agriculture Department is the nodal Department for implementation of the scheme.

13.17 District and State Agricultural Plans: The RKVY guidelines recognize the need for convergence and integration of the various programmes implemented at district / State level through Comprehensive District Agriculture Plan(C-DAPs) and State Agriculture Plan (SAP). Several State/UTs have prepared comprehensive District and State Agriculture Plan for 11th Plan, which are being revised and updated appropriately by the states for implementing RKVY during 12th Plan keeping in view of modification proposed for the plan period and emerging needs of the State.

13.18 Each district is required to formulate a C-DAP taking into account resources available from other ongoing Central or State Plan schemes such as Backward Region Grant Fund (BRGF), Swaranjayanti Gram Swarozgar Yojana (SGSY), National Rural Employment Guarantee Scheme (NREGS) and Bharat Nirman, etc. The requirements of the Gram Panchayats selected under Saansad Adarsh Gram Yojana (SAGY) would be also kept in mind while formulating the District Agriculture Plans (DAPs).

13.19 C-DAPs reflect the financial requirements and the sources of financing the agriculture development plans in a more holistic manner by including animal husbandry and fishery, minor irrigation projects, rural development works, agricultural marketing schemes and schemes for water harvesting and conservation taking into account natural resources and technological possibilities in each district. The DAPs are thereafter integrated to formulate the State Agriculture Plans (SAPs).

13.20 State Agriculture Infrastructure Development Programme (SAIDP): Revised Operational Guidelines of RKVY also mandated that each States to prepare SAIDP in a similar manner to that of DAPs and SAPs for identifying shelf of projects for RKVY (Infrastructure & Assets) stream. SAIDP should ideally be consolidation of requirement of infrastructure identified in DAPs and SAP.

13.21 Status of Preparation of District Agriculture Plans: Preparation of the District Agriculture Plans (DAPs) and the State Agriculture Plan (SAP) is the cornerstone of the strategy of implementation of RKVY. Most of the State Governments had prepared the DAPs (604 districts out of 617 districts in the country) in XI Plan. Infact, 27 States had completed SAP for the XI Plan period. Though the progresses of preparation of DAPs and SAP have been tardy during XII Five Year Plan, however, 247 DAPs out of 652 districts and 12 SAPs have been prepared.

13.22 Projects undertaken by States under RKVY: RKVY is designed to focus funds allocation and States' attention to selection of strategy and projects which will best help to generate growth in agriculture and allied sectors. State Governments, keeping in view their priorities, have approved project proposals for implementation under RKVY in wide ranging sectors which include crops, horticulture, organic farming, agriculture/farm mechanization, micro/minor irrigation, watershed development, agriculture marketing and storage, seed farms and soil/fertilizer testing laboratories, animal husbandry, dairy development, fisheries, extension and research, etc. Critical infrastructure such as State Seed farms, Soil and Fertilizer testing laboratories, starved over the years due to paucity of funds, got a much needed dose of assistance under RKVY across the States. The sector-wise cost of projects approved by States during 2012-13 to 2015-16 of XII Plan is given in **Annexure-13.1.** While, growth in agriculture and allied sector in States cannot be attributed entirely to RKVY projects as there are many other programmes and policies contributing for it,

RKVY has emerged as a principal instrument of development of agriculture and allied sector in States and has indeed accelerated revival of agriculture.

13.23 Sub-Schemes launched under RKVY: RKVY has enabled launching of new schemes/programmes keeping States' flexibility and authority intact. Since 2010-11 several sub-schemes have been introduced under RKVY with focused objectives. Following special Programmes/schemes are being implemented as sub-schemes of RKVY, which also include some new sub- schemes launched during the current financial year, with a total allocation of Rs.1069.20 crore for 2016-17.

i. **Bringing Green Revolution to Eastern India (BGREI):** Initiated in 2010-11, BGREI has been targeting productivity improvement in the rice based cropping system of Assam, West Bengal, Odisha, Bihar, Jharkhand, Eastern Uttar Pradesh and Chhattisgarh. Allocation for this scheme in 2010-11 & 2011-12 was Rs. 400 crore each, which had been enhanced to Rs. 1000 crore during 2012-13, 2013-14 & 2014-15. The allocation for this programme during 2015-16 was Rs.500 crore and for 2016-17 is Rs.630 crore.

ii. **Crop Diversification Programme (CDP):** Pursuant to announcement in Union Budget (2013-14), this scheme had been launched in 2013-14 with an allocation of Rs. 500 crore to promote technological innovation to encourage farmers to choose crop alternatives. The allocation for this programme for 2014-15 & 2015-16 are Rs.250 crore & Rs.150 crore respectively. The allocation for this programme for 2016-17 is Rs.180.00 crore.(including Rs. 30 crore earmarked for Crop Diversification from tobacco farming).

iii. **Reclamation of Problem Soil:** This programme is initiated as a sub-scheme of RKVY during 2016-17 for enhancing soil fertility & productivity for meeting demand of food grain of the country. The allocation for the scheme for the current financial year is Rs.50 crore.

iv. **Rice Fallow Areas in Eastern India for Pulses and Oil Seeds:** This is a new initiative launched under RKVY as a sub-scheme to bring area of rice fallow in Eastern India under pulses and oilseed cultivation. The allocation for the scheme for the current financial year is Rs.50 crore.

v. **Foot and Mouth Disease Control Programme**: This programme is initiated as a sub-scheme of RKVY during 2016-17 for supplementing the effort of the State Governments for prevention, control and containment of animal disease. The allocation for the scheme for the current financial year is Rs.100.65 crore.

13.24 Monitoring of RKVY: A web-based Management Information System for RKVY [Relational Database and Management Information System (RDMIS)] has been established to collect and disseminate relevant information and data related to each RKVY project and also to monitor progress and completion details of these projects over their life cycle. States are entrusted responsibilities' to enter data on approval & implementation of projects under RKVY online in the RKVY website (http.//www.rkvy.nic.in). RDMIS has been able to provide current and authenticated data on outputs, outcomes and growth impact of projects taken up under RKVY.

13.25 Flagship/Innovative Schemes: States have also taken up several flagship and innovative projects under RKVY, which reflect best practices, innovative approaches and achievements under RKVY. An illustrative list of these projects is at **Annexure-13.2.**

13.26 States have been reasonably prompt in approving projects & incurring expenditure

under RKVY. Status of year-wise funds released to the States & their expenditure & utilization status as on **31.12.2016** is given below:

(Rs. in crore)

Year	RE	Fund released	Expenditure Reported	%of utilization over release
2007-08	1263.00	1246.89	1246.79	99.99%
2008-09	2891.70	2886.80	2882.39	99.85%
2009-10	3707.07	3760.93	3756.53	99.88%
2010-11	6722.00	6720.06	6719.02	99.98%
2011-12	7810.87	7794.09	7728.24	99.16%
2012-13	8400.00	8400.00	8384.37	99.81%
2013-14	7089.00	7052.51	6962.13	98.72%
2014-15	8444.00	8443.20	8187.54	96.97%
2015-16	3900.00	3942.38	3418.36	86.71%
2016-17	3800.00	2610.30	884.04	33.87%

State-wise status of funds released for the years up to 2016-17 and expenditure/UC status as on 31.12.2016 is given in **Annexure 13.3.**

13.27 Conclusion: RKVY is a transformational jump in evolution of agricultural development schemes from variegated schematic approach followed so far through diverse but strait-jacketed schemes to a completely new approach in agriculture planning by allowing the States to plan their strategy and design appropriate schemes to implement that strategy with complete flexibility in scheme designs and autonomy in its implementation. RKVY has imparted definite momentum to the agriculture sector. Regular activities starved of resources, promising strategies languishing for lack of support, and limited opportunities for experimentation and innovation, have found a new hope in RKVY. States are allocating more funds to agriculture to take up schemes of agriculture development. This is reflected in additional plan allocation to agriculture sector. There is a sense of hope and optimism. Agriculture, which had slid back stage, is making a comeback. One timely policy instrument through RKVY has accelerated revival of agriculture and put it on growth path.

13.28 Audit Para: There is no Audit para pertaining to RKVY. However, there is a Performance Audit of Rashtriya Krishi Vikas Yojana (RKVY) done for the period 2007-08 to 2012-13 (ending March 2013) by C&AG who submitted the report in both the Houses of Parliament on 5th May 2015. The status of performance Audit of RKVY in the prescribed format is given under:

Sl. No	Year	No. of Paras/PA reports on which ATNs have been submitted to PAC after vetting by Audit	Details of the Paras/PA reports on which ATNs are pending		
			No. of ATNs not sent by the Ministry even for the first time.	No. of ATNs sent but returned with observations and Audit is awaiting their resubmission By the Ministry	No. of ATNs which have been finally vetted by Audit but have not been submitted by the Ministry to PAC.
1	2015	-	-	Performance Audit of RKVY has been done by C&AG for the period 2007-08 to 2012-13 (ending March 2013) who submitted the report in both the Houses of Parliament on 5th May 2015. The Draft ATN on the observations made in the report were furnished to Audit on 27.06.2016 on which vetting comments of Audit have been received. Efforts are being made to send the revised ATN at the earliest.	

Chapter 14

Drought Management

14.1. The Department of Agriculture, Cooperation & Farmers Welfare (DAC&FW) is mandated to coordinate relief measures necessitated by drought, hailstorm, pest attack, frost/cold wave. Spatial distribution and quantum of rainfall during South-West Monsoon (June- September) mainly determines the incidence of drought in the country, as South West Monsoon accounts for more than 70% of annual rainfall. Department of Agriculture, Cooperation & Farmers Welfare closely monitors progress of South-West Monsoon in the country, in close coordination with India Meteorolgical Department (IMD) and keeps a watch over scanty/deficient rainfall conditions.

14.2. The Department has reviewed and updated the Crisis Management Plan (CMP) for Drought 2016 (National) during the current year. The Plan defines roles and responsibilities of various agencies involved in crisis management including media management during drought. CMP 2016 (National) was circulated to State/UT Governments for preparing their own CMPs.

14.3. State Governments initiate necessary relief measures in the wake of natural calamities including drought from State Disaster Response Fund (SDRF) which is readily available with them. Contribution to SDRF is made by Central and State Governments in the ratio of 3:1 for general category States (18 out of 29 namely, Andhra Pradesh, Telangana, Bihar, Chhattisgarh, Goa, Gujarat, Haryana, Jharkhand, Karnataka, Kerala, Madhya Pradesh, Maharasthra, Odisha, Punjab, Rajasthan, Tamil Nadu, Uttar Pradesh and West Bengal) and in the ratio of 9:1 for special category States (11 out of 29 i.e. 8 North East States namely, Arunachal Pradesh, Assam, Meghalaya, Manipur, Mizoram, Nagaland, Tripura, Sikkim and 3 hilly States of Himachal Pradesh, Jammu & Kashmir and Uttarakhand). Government of India supplements the efforts of the State Governments by providing requisite financial and logistic support in the wake of calamities. Additional financial assistance, over and above SDRF, is also provided from (National Disaster Response Fund (NDRF) for natural calamities of severe nature as per established procedure and extant norms. Allocation under SDRF has been made on the basis of recommendations of the 14th Finance Commission for a period of 5 years from 2015-16 to 2019-20.

14.4. During the year 2016-17, as per information available till 31st December, 2016, Karnataka, Andhra Pradesh and Kerala had declared drought. Memorandum seeking central financial assistance from NDRF had been received from Karnataka and Andhra Pradesh. Inter-Ministerial Central Team (IMCT) was constituted to visit the affected areas of the State to assess the loss/damages to crops and recommend appropriate central financial assistance from NDRF.

14.5. Central Research Institute for Dryland Agriculture (CRIDA), Indian Council of Agricultural Research (ICAR) has developed detailed District-wise contingency plans to provide a broad advisory to farmers at the district level, prescribing alternate strategies in the event of climate variability, by factoring in crops/livestock/ aquaculture practices/pattern, soil characteristics, infrastructural facilities, etc. These plans are developed based on certain simulated models for different weather conditions like occurrence of drought, flood, cyclones, frost/cold wave etc. CRIDA has prepared Contingency Plans for 619 districts.

Chapter 15

International Cooperation

15.1 The mandate of International Cooperation is to foster mutually beneficial partnerships with other countries of the world in a multilateral as well as bilateral format. Department of Agriculture, Cooperation & Farmers Welfare is the Nodal contact point in Government of India for Food & Agriculture Organization (FAO) and World Food Programme (WFP) of the United Nations. Bilateral Agreement, Memorandum of Understanding (MoU), Protocols and Work plans with the countries of strategic interest are signed and implemented for furthering cooperation in the field of Agriculture & Allied sectors in coordination with the Ministry of External Affairs and other concerned Ministries and Departments.

Multilateral Cooperation

15.2 Food & Agriculture Organization (FAO): India is a founder member of the FAO and has been taking part in all its activities. India makes payment of the annual membership contribution to FAO, and has paid the contribution to FAO for the year 2016. India has been availing services from the FAO from time to time in the form of training, consultancy services, equipments and material in the field of agriculture and allied sectors under its technical cooperation programme (TCP). The major projects which are currently under implementation with FAO assistance are as under:

(i) Development of Extension and Outreach organizational and managerial capacities by state and public institutions in Mizoram: This project was signed on 22nd June, 2015 for a period of two years with the FAO assistance of US $ 496.000. Project will contribute to the State of Mizoram's development plans by supporting Departments to improve their outreach and extension support using current key production constraints, especially in the areas of managing an introduced pig disease - Porcine Reproductive & Respiratory Syndrome (PRRS), sustainable cropping on sloping areas and community forest policy, to develop new and innovative solutions to service delivery.

(ii) Global Environment Facility (GEF) 6 Formulation in India: This project was signed on 26th June, 2015 for a period of 18 months with the FAO assistance of US $ 110.787. Under this project, a GEF proposal covering four focal areas : i) Climate Change Mitigation/Programme 4; ii) Biodiversity/ Programme 7; iii) Land degradation/Entire Focal Area; and iv) Special Climate Change fund for Adaptation is being prepared by FAO India.

(iii) Strengthening National Forest Inventory and Monitoring Protocols and Capacities in India: This project was signed on 1st May, 2016 for a period of 18 months with the FAO assistance of US $ 397.000. Under this project, Government, Industry and relevant stakeholders actively promote more environmentally sustainable development; and resilience of communities is enhanced in the face of challenges posed by Climate Change, disaster risk and natural resource depletion.

(iv) Technical Cooperation Program Facility (TCPF): Strengthening institutional capacities for sustainable mountain development in the Indian Himalayan Region: This project was signed on 11th July, 2016 for duration of 2 years with the FAO assistance of US $ 99.955. Project will contribute to enhanced institutional capacities for sustainable mountain development in the Indian Himalayan Region (IHR), with a focus on agriculture and allied sectors through targeted support for institutional development and policy advocacy. The initiative will further contribute to informed and evidence-based decision and policy making on mountain agriculture and allied sectors (including animal husbandry, fisheries, forestry, biodiversity, land degradation and climate change).

(v) Technical Cooperation Program Facility (TCPF): Promoting Nutrition Education and Communication in India: This project was signed on 1st August, 2016 for a period of 17 months with the FAO assistance of US $ 99.821. The project aims to demonstrate the application of a communication intervention to improve behaviours at the intersection of agriculture and nutrition in India.

(vi) Technical Cooperation Program Facility (TCPF): Supporting Project Preparation of FAO India GEF 6 Full Scale Project: This project was signed on 1st August, 2016 for a period of 17 months with the FAO assistance of US $ 99.821. Project will support a range of project preparation activities to design the Full Size Global Environment Facility (GEF) 6 Project (USD 36.9 million) Green-Agriculture: Transforming Indian agriculture for global environmental benefits and the conservation of critical biodiversity and forest landscapes, which aims to catalyze transformative change for India's agricultural sector to support achievement of national and global environmental benefits and conserve critical biodiversity and forest landscapes. The project will be implemented at the National level and in five states viz. Rajasthan, Madhya Pradesh, Odisha, Uttarakhand, and Mizoram.

(vii) Strengthening Agricultural Market Information Systems in India using innovative methods and digital technology (Baby Project) : This project was signed on 14th September, 2015 for a period of 21 months with the FAO assistance of US $ 884.374. The project supports national efforts to produce quality statistics and data on crop production forecasts, market food prices and stocks for the four AMIS commodities (maize, rice, soybeans and wheat), by incorporating international best practices and experiences.

(viii) Programme Support to Nationally Executed (NEX) Land and Water Programme in India: This project was signed on 1st January, 2004 with the FAO assistance of US $ 4652.611. The objectives of the project is to ensure high quality results and impact of the Andhra Pradesh Land and Water Management Programme, in accordance with FAO's role and responsibilities vis-à-vis the three NEX projects; to facilitate synergy between these projects and other normative activities of the Organization and to promote further programme development in this area in India. The project is going to be expired on 31st December, 2016.

(ix) Green-Agriculture: Transforming Indian agriculture for global environmental benefits (PPG): This project was signed on 16th August, 2016 for a period of 16 months with the FAO assistance of US $ 300.000. The objective of the PPG is to support the preparation of the full project document which will consist of relevant baseline data and information collected during the preparation period, workshop reports, consultant's analysis and cost/efficient proposals, financing plan, risk analysis and

mitigation measures and realistic targets and indicators.

(x) Technical Assistance to Farmer Water School Programme and Agricultural Activities under the Uttar Pradesh Water Sector Restructuring Project (UPWSRP) Phase II : This project was signed on 9th March, 2016 with the FAO assistance of US $ 1847.539. The objective of the project is to strengthen the institutional and policy framework for integrated water resources management for the entire State and to increase agricultural productivity and water productivity by supporting farmers in targeted irrigation areas. The project is going to be expired on 30th October, 2020.

(xi) Incorporating International Best Practices in the Preparation of Agricultural Outlook and Situation Analysis Reports for India - Phase II : This project was signed on 3rd March, 2014 for a period of 26 months with the FAO assistance of US $ 461.139. The project is designed for supplementary funding to MoA project for carrying out critical tasks that will lead to better availability of information for policy makers.

World Food Programme (WFP)

15.3 The World Food Programme (WFP) was set up in 1963 jointly by the United Nations and Food & Agriculture Organization (UN/FAO). India is the member of WFP since its inception. It seeks to provide emergency feeding in places facing acute food insecurity due to natural calamities and man-made causes. Present share of Government of India for a biennium is US $ 1.92 million towards WFP Pledge Contribution which is used by them to support WFP India Country Programme. India has made full payment to UNWFP for biennium 2015-2016. Besides, GoI makes an annual payment of Rs. 30 lakh to UNWFP 'Country Office towards their Local Operating Cost (LOC).

15.4 Contribution for the year 2016 has been released as requested by WFP. Government of India (GoI) has recently decided that the amount of US $ 1.92 million (Biennium) towards GoI's pledge contribution to UNWFP will remain the same for the year 2017 as well.

15.5 A Country Strategic Programme (CSP) 2015-2018 has been signed between WFP and GoI and meeting of the Country Advisory Committee (CPAC) of WFP India was held on 18th April, 2016 to discuss CSP 2015-18 and Annual Work Plan thereof. Recently a 'Letter of Intent' has also been signed for establishment of Center of Excellence (CENEX) under strategic outcome of CSP 2015-2018.

15.6 World Food Day (WFD): World Food Day (WFD) was celebrated throughout the Country on 16th October, 2016 to commemorate the founding day of the FAO and to create public awareness about the plight of the hungry and malnourished people and to take concrete action to tackle and overcome the menace of hunger. This year's theme of WFD was **"Climate is changing. Food and Agriculture must too".**

15.7 Office of Minister (Agriculture), Embassy of India, Rome: The Government of India for the purpose of maintaining liaison and coordination with the FAO has nominated the Indian Ambassador in Rome as its permanent representative to the FAO. Keeping in view, the specialized nature and the increasing volume of interaction with the UN agencies and their associated bodies, an agricultural wing headed by a senior officer of the rank of Joint Secretary to the Government of India with the designation of Minister (Agriculture), EOI, Rome has been set up in the Indian Embassy, Rome. The Minister (Agriculture), EOI, Rome has been

designated as India's Alternate Permanent Representative (APR) to the FAO, the World Food Programme (WFP) and the International Fund for Agricultural Development (IFAD). The Minister (Agriculture), EoI, Rome represents Indian interests, particularly in the field of agriculture and allied sectors, at the meeting of various UN Agencies. The Office of Minister (Agriculture), EoI, Rome is a Subordinate Office under the administrative control of this Department.

Bilateral Cooperation

15.8 MoU/MoC/Agreements/Work Plans: Ministry of Agriculture and Farmers Welfare has entered into 58 MoUs/MoCs/ Agreements with 56 countries in the field of agriculture. During the year 2016, MoU/ MoC/Agreement/Work Plan/Declaration etc. were signed by the Department of Agriculture, Cooperation and Farmers Welfare with corresponding Ministry of Armenia on 19.02.2016, Madagascar on 29.02.2016, Lithuania on 19.07.2016, Japan on 11.11.2016 and Kyrgyzstan on 20.12.2016.

15.9 In addition to these, a Memorandum of Understanding (MoU) between India-Taipei Association in Taipei and Taipei Economic and Cultural Centre in India was signed on 12.09.2016 and an MoU between Government of India and the Global Crop Diversity Trust (GCDT), Bonn, Germany was signed on 07.11.2016. With a view to develop better understanding on issues of mutual interest, a one day workshop was organized on 26th April 2016 at New Delhi, with participation of experts and officials from USDA, in which issues relating to four areas: crop insurance; global agriculture situations and trends; US farm bill and issues under WTO, were discussed.

15.10 Joint Working Group (JWG) Meetings : During the year 2016, following JWG meetings were held :

(i) Meeting of India-Netherlands Joint Working Group (JWG) on Agriculture was held on 20.05.2016 in Netherlands.

(ii) 1st meeting of India-Armenia Joint Working Group (JWG) on Agriculture was held during 14-15 October, 2016 in Armenia.

(iii) 6th meeting of India-Nepal Joint Working Group (JWG) on Agriculture was held on 8-9 September, 2016 in India.

(iv) 1st meeting of India-Serbia Joint Working Group (JWG) on Agriculture was held on 15.11.2016 in India, through video conference.

(v) 5th meeting of India-Germany Joint Working Group (JWG) on Agriculture was held on 16.11.2016 in Germany.

(vi) 8th meeting of India-France Joint Working group (JWG) on Agriculture was held on 6-7 December, 2016 in France.

15.11 Indo-US Cooperation: National Institute of Plant Health Management (NIPHM), Hyderabad has been collaborating with United States Agency for International Development (USAID) for training purposes and capacity building programmes. National Institute of Agriculture Extension Management (MANAGE), Hyderabad & National Institute of Agriculture Marketing (NIAM), Jaipur are also collaborating with USAID to provide training for national of three African Countries viz. Liberia, Kenya and Malawi under trilateral Cooperation envisaged under MoU with USA. Ten more countries have been included under India-USAID Trilateral Cooperation. A process for signing of a new MoU between India and USA in agriculture and allied sector is in pipeline.

Strategic Groups

15.12 India is a member of multilateral groupings such as **G-20** – a forum for global

cooperation on international economic and financial issues; **IBSA** (India, Brazil and South Africa); **BRICS** (Brazil, Russia, India, China and South Africa); **SAARC** (South Asian Association for Regional Cooperation); **ASEAN** (Association of South East Asian Nations); **BIMSTEC** (Bay of Bengal Initiative for Multi-sectoral Economic & Technical Co-operation) etc.

15.13 G-20: This year, G-20 Agriculture Deputies meeting was held on 1-2 June, 2016 at Xi'an China, followed by G-20 Agriculture Ministers meeting on 3rd June, 2016 at the same venue. Final communiqué was issued from G-20 Agriculture Ministers on the occasion.

15.14 BRICS : The Department successfully organized the 6th meeting of Agriculture Ministers of the BRICS countries on 23rd September, 2016 in New Delhi. A joint declaration on the way ahead for our future initiatives and continued cooperation was adopted unanimously. The declaration envisages BRICS as an important agriculture platform for developing, testing and sharing models of sustainable technology in the face of climate change; the importance of deploying ICT in agriculture for giving farmers access to inputs, technology and financial services; promotion of agriculture sustainability as a key component of the 2030 agenda for sustainable development; the need to prioritize and strengthen support for small holder farmers; improved water management given the dependence of agriculture on water and giving protection and preservation of cultivable land. The declaration also aims to promote production of pulses in the BRICS countries given the crops' beneficial influence on soil fertility and in ameliorating malnutrition.

OTHERS

15.15 India-Africa Agribusiness Forum: This Ministry, in collaboration with FCCI successfully organized the India-Africa Agribusiness Forum at New Delhi on 10-11th February, 2016 in which several Ministries, government officials and company representatives participated.

15.16 Cooperation with International Organizations: India is a member of many International Organizations and the Annual contributions to these International Organizations are being made from time to time as per agreed commitment/agreement by the IC Division. These International Organizations are as under :-

i) Food and Agriculture Organization of United Nations (FAO).
ii) World Food Programme (WFP).
iii) Trust Fund of FAO for Desert Locust in Eastern Region.
iv) Trust Fund for International Desert Locust.
v) Organization for Economic Cooperation & Development (OECD).
vi) Asia and Pacific Coconut Community (APCC).
vii) Asia-Pacific Plant Protection Convention (APPPC).
viii) Global Crops Diversity Trust Fund (GCDT)

15.17 International Seminars/Workshops/ Conferences etc. : During 2016-17 (upto December, 2016), this Department took part in 110 International Conferences/Meetings/ Seminars/Training etc. dealing with various aspects of agricultural development organized by the FAO and other International Organizations.

15.18 External Assistance: There are various Projects which are being implemented in different States of India with the help of external assistance provided by various foreign Agencies/Countries such as World Bank, International Fund for Agriculture Development (IFAD) and Asian Development Bank (ADB) etc.

15.19 World Bank Assisted Projects: Currently there are 10 World Bank assisted Projects which are being implemented through this Department. These projects are as under:-

(i) Assam Agricultural Competitiveness Project (AACP);

(ii) Additional financing for Assam Agricultural Competitiveness Project;

(iii) National Dairy Support Project;

(iv) Himachal Pradesh Mid-Himalayan Watershed Development;

(v) Additional financing for Himachal Pradesh Mid-Himalayan Watershed Development;

(vi) Uttar Pradesh Sodic Land Reclamation Project-III;

(vii) Maharashtra Agricultural Competiveness Project (MACP);

(viii) Rajasthan Agricultural Competiveness Project (RACP);

(ix) Uttaranchal Decentralised Watershed Development Project;

(x) Additional financing for Uttarakhand Decentralised Watershed Development Project.

15.20 International Fund for Agriculture Development (IFAD) assisted Projects: At present there is only one IFAD assisted project viz. Convergence of Agricultural Interventions in Maharashtra's Distressed Districts Programme.

15.21 Asian Development Bank (ADB) assisted Projects : Currently there are two ADB assisted Projects viz. (i) Agribusiness Infrastructure Development Investment Programme–1 (Bihar) (ii) Agribusiness Infrastructure Development Investment Programme – 2 (Maharashtra).

******

Chapter 16

Agricultural Trade

India's Agriculture Trade

16.1 India has emerged as a significant agri-exporter in a few crops viz. rice, cotton, sugarcane, cashew nut, castor seed and groundnut. As per WTO's Trade Statistics, the share of India's agricultural exports and imports in the world agriculture trade in 2015 were 2.26% and 1.74%, respectively.

16.2 Agricultural exports as a percentage of agricultural GDP has increased from 12.14% in 2011-12 to 12.19 % in 2014-15. During the same period, Agricultural imports as a percentage of agricultural GDP also increased from 4.66% to 5.88%.

Agricultural Exports and Imports

16.3 Export of agricultural commodities has helped producers to take advantage of wider international market which in turn has incentivized their domestic production. Crops exported in large quantities viz. rice, cotton, and maize have witnessed significant increase in area coverage and growth rate of production.

16.4 Agricultural exports decreased from Rs. 2,62,778 crore in 2013-14 to Rs. 2,13,556 crore in financial year 2015-16 registering a decline of nearly 18.73%. Decrease in the value of agricultural exports during 2015-16 was primarily on account of lower exports of wheat, oil meals, guargum meals, cotton, basmati & non-basmati rice. The share of agricultural exports in India's total exports decreased from 13.79% in 2013-14 to 12.46% in 2015-16.

16.5 India's top 10 agricultural export commodities in terms of quantity and value for the year 2013-14, 2014-15, 2015-16 & 2016-17 (April to September) are given in the table 1 below:

Table 1: India's top 10 agricultural export commodities

[Quantity in '000' tons; Value in Rs. Crores]

	**Commodity**	2013-14		2014-15		2015-16		2016-17 (Apr-Sept)	
		**Qty**	**Value**	**Qty**	**Value**	**Qty**	**Value**	**Qty**	**Value**
(1)	(2)	(3)	(4)	(5)	(6)	(7)	(8)	(9)	(10)
1	MARINE PRODUCTS	1,001	30,627	1,073	33,685	976	31183	493	18686
2	BUFFALO MEAT	1,366	26,458	1,476	29,283	1314	26682	629	12332
3	RICE -BASMATI	3,754	29,292	3,702	27,599	4045	22714	2067	10924
4	SPICES	897	15,146	923	14,842	821	16374	475	9416
5	RICE(OTHER THAN BASMATI)	7,148	17,795	8,226	20,336	6374	15086	3369	8616
6	COTTON RAW INCLD. WASTE	1,948	22,338	1,143	11,643	1346	12816	210	2061
7	SUGAR	2,478	7,179	1,954	5,327	3826	9772	1392	4318
8	CASHEW NUT	121	5,095	135	5,566	103	5025	37	2158
9	OIL MEALS	6,577	17,070	3,904	8,129	2056	3599	814	1380
10	GUERGAM MEAL	602	11,735	665	9,480	325	3233	164	1208

Source: Deptt of Commerce

16.6 India's agricultural imports increased from Rs. 85,727 crore in 2013-14 to Rs 1,39,933 crore in 2015-16 registering a growth of nearly 63.2 %. Increase in value of agricultural imports during this period was primarily on account of imports of vegetable oils, pulses, fruits, cashew nuts, spices, sugar and cotton. Share of agricultural imports in the total imports increased from 3.16 % in 2013-14 to 5.63 % in 2015-16.

16.7 India's top 10 agriculture import commodities for the year 2013-14, 2014-15, 2015-16 and 2016-17(April to September) are given in the table 2 below:

Table 2: India's top 10 agriculture import commodities

[Quantity in 000 tons, Value in Rs crores]

	Commodity	2013-14		2014-15		2015-16		2016-17 (Apr-Sept)	
		Qty	Value	Qty	Value	Qty	Value	Qty	Value
(1)	(2)	(3)	(4)	(5)	(6)	(7)	(8)	(9)	(10)
1	VEGETABLE OILS	7,943	44,038	11,548	59,094	15639	68630	6996	35151
2	PULSES	3,178	11,037	4,585	17,063	5798	25619	2014	10501
3	FRESH FRUITS	769	7,716	858	9,544	836	11013	451	4800
4	OTHER WOOD AND WOOD PRODUCTS	–	12,500	–	11,888	--	10194	--	4381
5	CASHEW NUT	776	4,668	941	6,600	962	8701	506	5449
6	SPICES	156	3,452	161	4,392	191	5382	140	2778
7	SUGAR	881	2,287	1,539	3,668	1943	4038	661	1904
8	COTTON RAW INCLD. WASTE	181	2,376	259	3,101	232	2563	333	4003
9	MISC PROCESSED ITEMS	–	1,474	–	1,749	--	1799	--	1030
10	COFFEE	60	729	75	930	66	802	37	415

Source: Department of Commerce

16.8 Share of top 10 exported and imported agri-commodities during 2014-15 is as follows:

Export Share in 2015-16

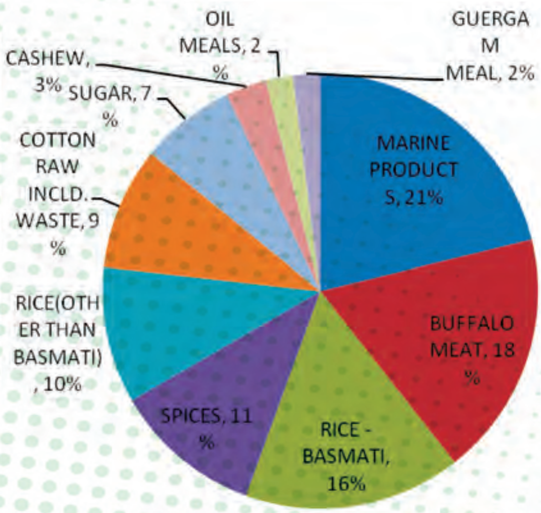

Import Share in 2015-16

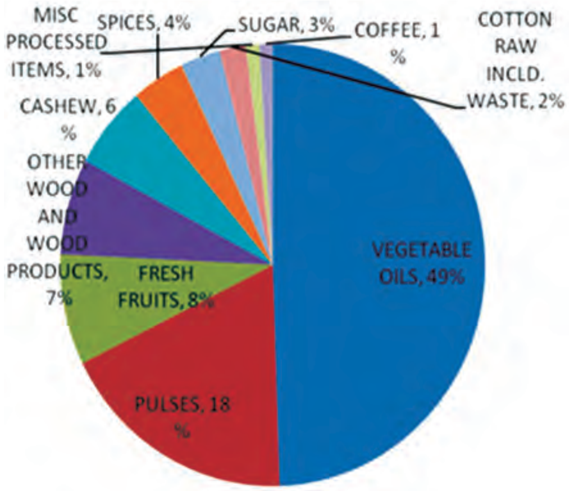

Source: Department of Commerce

Foreign Trade Policy

16.9 India, with a large and diverse agriculture, is among the world's leading producers of rice, wheat, sugarcane, castor seeds, groundnut, millet, sesame seed, fruits and vegetables. Therefore, changes in its balance sheets for key commodities will have a potentially large impact on world markets. Over the years, India has developed export competitiveness in certain specialized agriculture products viz. basmati rice, guar gum and castor.

16.10 Trade Policy has been amended from time to time on various agricultural commodities in response to domestic availability and price situation. The basic customs duty (BCD) in some agri products was reduced/removed to encourage domestic manufacture of value added products, to generate employment, and to make exports competitive. To combat undervaluation and protect the interests of domestic farmers and industry, the BCD of some agri products like sugar, vegetable edible oil and wheat was raised. Import duty on sugar was increased from 25% to 40% vide Customs Notification dated 30th April, 2015. Import duty on crude and refined edible oils has been raised from 7.5% to 12.5% and 15% to 20% respectively vide Custom notification dated 17.09.2015. Current Applied Duty on Crude Palm has been decreased from 12.5% to 7.5% and on refined palm oil from 20% to 15% by Department of Revenue vide Notification No. 51/2016-Customs dated 23.09.2016. Further, import duty on wheat was first raised from "zero" 10% in August 2015 and from 10% to 25% in October 2015. However, duty on wheat was brought down to 10% on 23rd Sept 2016. Recently, the Import duty on wheat was brought down to Zero vide Notification No. 60/2016- Customs dated 8th December, 2016. Minimum Export Price of potato has been removed vide notification dated 27th December, 2016.

Foreign Direct Investment (FDI) Policy in Agriculture

16.11 Hundred percent FDI has been allowed in development and production of seeds and planting material. In addition, 100% FDI is allowed for floriculture, horticulture and cultivation of vegetables and mushrooms under controlled conditions. Also, hundred percent FDI is allowed in animal husbandry (including breeding of dogs), pisciculture, aquaculture and services related to agro and allied sectors. Similarly, hundred percent FDI is allowed in the planation sector namely tea, coffee, rubber, cardamom, palm oil tree and olive oil tree.

16.12 From April 2000 to September 2016, FDI inflows of Rs. 11,679.38 crores have been received in agriculture sector (i.e. agriculture services including agriculture machinery). The investments were made in development and production of seed and planting material, horticulture and nursery services, agriculture machinery, plant protection services, cattle breeding and livestock rearing, cold storage and warehousing.

Institutional Mechanism for in-house knowledge management Database on Agricultural Trade

16.13 In order to create a database of India's trade flows (global export and import in value and quantity terms) for all agricultural products, this Department has hired consultancy services of Consumer Unity & Trust Society (CUTS), Jaipur in October 2014.

16.14 The CUTS, *inter alia,* is required to:

- Analyse database to capture broad export trends, conduct competitiveness analysis of India's agriculture products for exports and identify the tariff lines with (i) high export growth and (ii) other products having export potential.

- Analyse the database to capture broad import trends, conduct an analysis of vulnerability of India's agriculture products from imports and identify the products/tariff lines with imports threat that would require duty protection on account of livelihood and food security.
- Provide inputs on issues relating to agriculture in World Trade Organization (WTO), other bilateral negotiations relating to trade and any other trade related issue.

Commodity Profile

16.15 Trade Division has been regularly publishing commodity profiles of six prime agricultural products viz. Rice, Wheat, Pulses, Vegetable Edible Oil, Sugar and Cotton. The profile gives a snapshot on the trends in production, export, import, price movements and explains trade policy in brief for the commodity concerned.

16.16 The commodity profiles are placed in the public domain and are updated once in each month. These profiles can be viewed by clicking 'Trade' at http://agricoop.nic.in/divisions.html

16.17 Chapter-wise export and import data of 352 agriculture tariff lines at six digit HS level indicating bound rate and applied rate from 2009-10 to 2015-2016 is hosted on the website. (http://agricoop.nic.in/divisions.html)

Audit Para

16.18 No audit para/ observation are pending in Trade Division of this Department.

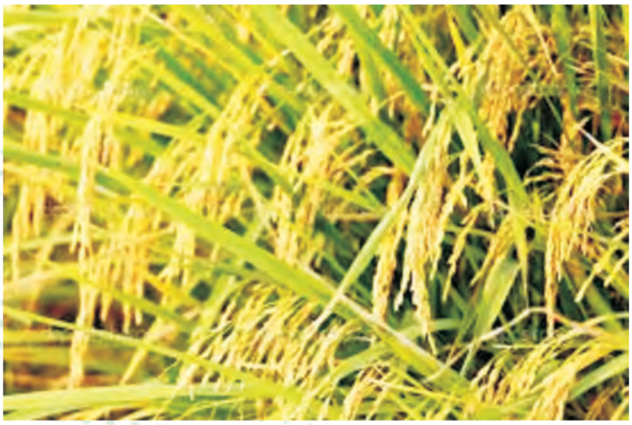
Rice

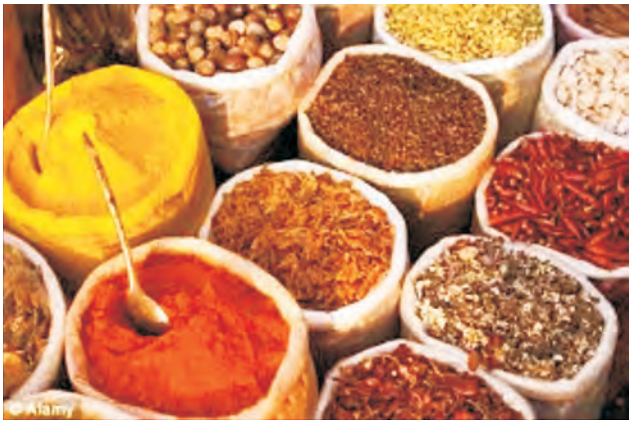

Indian Spices

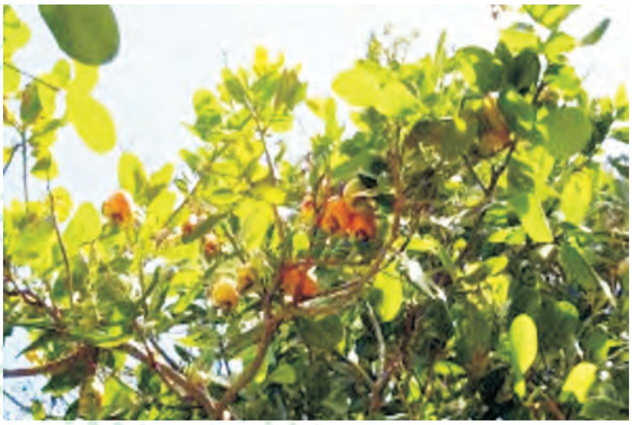
Cashew Plant

Chapter 17

Agricultural Credit

17.1 The Government has taken many policy initiatives for strengthening of farm credit delivery system for providing credit at lower rates of interest to support the resource requirements of the agricultural sector. The emphasis of these policies has been on providing timely and adequate credit support to farmers with particular focus on small and marginal farmers and weaker sections of society to enable them to adopt modern technology and improved agricultural practices for increasing agricultural production and productivity. The policy essentially lays emphasis on augmenting credit flow at the ground level through credit planning, adoption of region specific strategies and rationalization of lending policies and procedures and bringing down the rate of interest on farm loan.

Policy initiatives for increasing the flow of credit

17.2 The Government has initiated several measures to galvanize the institutional credit system to make them more responsive to the needs of farmers. Some of the important measures initiated in this regard are as under:-

Agriculture Credit

17.3 Agricultural credit flow has increased consistently over the years and it reached Rs.877,527 crore against the target of Rs.850,000 crore during 2015-16. Target for the year 2016-17 has been fixed at Rs.900,000 crore and the achievement is Rs.755,995 crore (upto September, 2016). The target of Rs. 9,00,000 crore is likely to be surpassed by the end of the financial year 2016-17.

Kisan Credit Card

17.4 In order to ensure that all eligible farmers are provided with hassle free and timely credit for their agricultural operation, Kisan Credit Card (KCC) scheme was introduced in 1998-99. Marginal farmers, share croppers, oral lessee and tenant farmers are eligible to be covered under the Scheme. The main objectives of the Scheme are to meet the short term credit requirements for cultivation of crops, post harvest expenses, produce marketing loan, consumption requirements of farmer household, working capital for maintenance of farm assets and activities allied to agriculture, like dairy animals, inland fishery etc. investment credit requirement for agriculture and allied activities like pump sets, sprayers, dairy animals etc. The State Governments have been advised to launch an intensive branch/ village level campaign to provide Kisan Credit Card to all the eligible and willing farmers in a time bound manner. KCCs have now been converted into Smart Card cum Debit Cards to facilitate its operation through ATMs. The cumulative number of live KCCs issued by Commercial Banks, Cooperative Banks & Regional Rural Banks as on 31 March, 2016 was 754.64 lakh with outstanding loan amount of Rs.530034.58 crore. The cumulative number of live KCCs is estimated to increase to 756.64 lakh with outstanding loan amount of Rs. 5,30,134.58 crore by 31.03.2017. Some of the major features of revised KCC Scheme are:

- Assessment of crop loan component based on the scale of finance for the crop plus insurance premium x Extent of area cultivated + 10% of the limit towards post-harvest/ household/ consumption requirements + 20% of limit towards maintenance expenses of farm assets.
- Flexi KCC with simple assessment prescribed for marginal farmers.
- Validity of KCC for 5 years.
- For crop loans, no separate margin need to be insisted as the margin is in-built in scale of finance.
- No withdrawal in the account to remain outstanding for more than 12 months; no need to bring the debit balance in the account to zero at any point of time.
- Interest subvention /incentive for prompt repayment to be available as per the Government of India and / or State Government norms.
- No processing fee up to a limit of Rs.3.00 lakh.
- One time documentation at the time of first availment and thereafter simple declaration (about crops raised/ proposed) by farmer.
- KCC cum SB account instead of farmers having two separate accounts. The credit balance in KCC cum SB account to be allowed to fetch interest at saving bank rate.
- Disbursement through various delivery channels, including ICT driven channels like ATM/ PoS/ Mobile handsets.

Interest Subvention Scheme

17.5 The Government has been implementing Interest Subvention Scheme since 2006-07. Under the Scheme, interest subvention of 2% per annum is provided to Public Sector Banks, Private Sector Scheduled Commercial Banks, Cooperative Banks and Regional Rural Banks on their own funds used for short term crop loans upto Rs.3.00 lakh per farmer provided the lending institutions make available short term credit at the ground level at 7% per annum to farmers. Farmers are provided with 3% additional interest subvention for the short term crop loan of upto Rs.3.00 lakh for a maximum period of one year for prompt repayment on or before the due date. Thus, farmers, who promptly repay their crop loans as per the repayment schedule fixed by the banks, are extended loans at an effective interest rate of 4% per annum. Government has extended the scheme to crop loans borrowed from private sector scheduled commercial banks in respect of loans given within the service area of the branch concerned.

17.6 Further, in order to discourage distress sale by farmers and to encourage them to store their produce in warehouses against warehouse receipts, the benefit of interest subvention scheme has been extended to small and marginal farmers having Kisan Credit Card for a further period upto six months post harvest on the same rate as available to crop loan against negotiable warehouse receipt for keeping their produce in warehouses.

17.7 To provide relief to farmers affected by natural calamities, the interest subvention of two percent continues to be available to banks for the first year on the restructured amount. Such restructured loans may attract normal rate of interest from the second year onwards as per the policy laid down by the RBI.

Release of funds to RBI/NABARD for settling the claims under Interest Subvention Scheme

17.8 The Interest Subvention Scheme was earlier implemented by Department of Financial Services. During the financial year 2015-16, the Government allocated a

sum of Rs.13,000 crore to Department of Financial Services for settling the claims under Interest Subvention Scheme and the entire sum was released to RBI/NABARD, the implementing agencies. The Interest Subvention Scheme has been transferred to the Department of Agriculture, Cooperation and Farmers Welfare from the current financial year 2016-17. The Government has allocated a sum of Rs.15,000 crore to Department of Agriculture, Cooperation and Farmers Welfare for settling the claims under Interest Subvention Scheme. A total sum of Rs.12,558.63 crore has been released to RBI/NABARD as on 31.10.2016 for settlement of audited claims under Interest Subvention Scheme. Details of funds released during the last five years are as under:

(Rs. In crore)

2012-13	2013-14	2014-15	2015-16	2016-17
5,400	6,000	6,000	13,000	12,558.63 (As on 31.10.2016)

Joint Liability Group (JLG)

17.9 Joint Liability Group is an informal group comprising 4 to 10 individuals coming together for the purpose of availing bank loan on individual basis or through group mechanism against mutual guarantee. The JLG mode of financing serves as collateral substitute for loans to be provided to the target group i.e. small, marginal, tenant farmers, oral lessees, share croppers, etc. It builds mutual trust and confidence between the bank and the target group and minimizes the risks in the loan portfolio for the banks through group dynamics, cluster approach, peer education and credit discipline. The objective of the JLG mode of financing is to provide food security to vulnerable section by enhanced agriculture production, productivity and livelihood promotion. JLGs can also easily serve as a conduit for technology transfer, facilitating common access to market information, training and technology dissemination in activities like soil testing, training and assessing input requirements, etc.

17.10 The Scheme for financing of Joint Liability Groups of Tenant Farmers was started by NABARD in 2005-06. The scheme was extended to non-farm sector from 2009 onwards. Thus, JLGs consists of those of farmers and also of non-farmers. The exclusive scheme for Bhoomi Heen Kisan was launched by Government of India during the year 2014-15 with a target for financing 5 lakh Joint Farming Groups of "Bhoomi Heen Kisan" through NABARD. The total number of JLGs and total loan amount provided (cumulative) as on 31.12.2016 are as under:

No. JLGs financed as on 31.12.2016	
No.	Amount
18,23,507	Rs.18,28,232.68 lakh

The number of JLGs and loan amount are estimated to increase by 5,45,215 JLG and Rs. 6,26,997.30 lakh by 31.03.2017.

Investment in Debentures of State Cooperative Agriculture & Rural Development Banks (SCARDB)/State Cooperative Land Development Banks (SCLDB)

17.11 The Scheme for "Investment in the Debentures of State Land Development Banks" is a continuing plan scheme and has been in existence since 1966-67 (Third Five Year Plan). The State Land Development Banks (SLDBs)/State Cooperative Agriculture & Rural Development Banks (SCARDBs) raise resources for long term lending to cultivators by floating debentures which are trustee securities. The debentures floated by SLDBs/SCARDBs are subscribed by concerned State Governments, Government of India. The Special Development Debentures are floated for minor irrigation; horticulture/plantation, farm mechanization, land development,

wasteland, rural housing, Rural go-downs, non-farm sector and animal husbandry etc. While switching over to loan system, NABARD has decided to limit the quantum of refinance to SCARDBs uniformly at 90% of total loans issued and the balance of 10% refinance support from Central & State Governments on 50:50 basis by way of investment in the debentures of SCARDBs. The share of Government of India is 5% in respect of Special Development Debentures. The scheme is demand driven and no State-wise allocation is made under the scheme.

17.12 Position of funds invested by Government of India in the Debentures of State Land Development Banks/ State Cooperative Agriculture & Rural Development Banks during last five years is as under:-

(Rs. in crore)

2011-12	2012-13	2013-14	2014-15	2015-16
30.24	25.00	24.66	10.89	12.30

For the year 2016-17, Rs.25.00 crore has been provided at RE stage.

Relief measures for natural calamities

17.13 Reserve Bank of India (RBI) has issued guidelines for relief measures by banks in areas affected by natural calamities vide their circular dated 21.08.2015 that State Level Bankers' Committee/District Level Consultative Committees/Banks are directed to take a view on rescheduling of loans if the crop loss is 33% or more. Banks have been advised to allow maximum period of repayment of upto 2 years (including the moratorium period of 1 year) if the crop loss is between 33% and 50%. If the crop loss is 50% or more, the restructured period for repayment is extended to a maximum of 5 years (including the moratorium period of 1 year). The Banks have further been advised that:

- All short-term loans are eligible for restructuring. The principal amount of the short-term loan as well as interest due for repayment is converted into term loan.
- In all cases of restructuring, moratorium period of at least one year is granted. Further, the banks should not insist for additional collateral security for such restructured loans.
- The existing term loan instalments are rescheduled. The banks have been directed to reschedule the payment of instalment during the year of natural calamity and extend the loan period by one year. The banks may also have to postpone payment of interest by borrowers.
- Fresh crop loans are granted to the affected farmers which will be based on the scale of finance for the particular crop and the cultivation area, as per the extant guidelines.
- A crop loan continues at concessional rate of interest viz. 7% till completion of one year. Thereafter the interest at normal rate of interest is charged.

Sarangi Committee

17.14 Following a Cabinet Decision the Sarangi Committee was set up under the Chairmanship of Shri U. C. Sarangi, former Chairman, NABARD, in October, 2015 to suggest feasible measures/options for improving targeted lending to small and marginal farmers. The Committee has since in its report made several recommendations which are under consideration with the Government.

******

Chapter 18

Gender Perspective in Agriculture

18.1 National Policy on Farmers 2007 has included "mainstreaming the human and gender dimensions in all farm policies and programmes as one of the major policy goals". In line with the Policy directives, appropriate structural, functional & institutional measures are being taken to empower women in agriculture and allied sectors by building their capacities and improving their access to inputs, technologies and other farming resources. While the main objective of the Department of Agriculture, Cooperation & Farmers Welfare is to enhance the agricultural production and productivity, Department is also promoting mainstreaming of gender concerns in a big way by incorporating 'pro-women initiatives' and earmarking at least 30% of benefits and resources for women under all its major beneficiaries oriented Schemes and Programmes. Focus is also being given on formation of women Self Help Groups (SHGs), capacity building interventions, linking them to micro credit, enhancing their access to information and ensuring their representation in decision making bodies at various levels.

18.2 The National Gender Resource Centre in Agriculture (NGRCA) set up in the Department of Agriculture, Cooperation & Farmers Welfare, Ministry of Agriculture & Farmers Welfare and supported under the Central Sector Component of Sub-Mission on Agricultural Extension (SMAE) acts as a focal point for convergence of all gender related activities & issues in agriculture & allied sectors within and outside the Department. The Centre is also contributing towards adding gender dimension to agriculture policies & programmes and rendering advocacy / advisory services to the States/ UTs to internalize gender specific interventions in policies and programmes of agriculture sector.

18.3 Besides undertaking and supporting training, research and advocacy to mainstreaming gender issues in agriculture and natural resource management, NGRCA aims at forging effective functional linkages with other related departments, agencies and institutions & is mandated to ensure that the policies and programmes in agriculture are fully engendered & reflect the national commitment to empowerment of women.

18.4 Gender Budgeting Cell (GBC) has been constituted in the Department of Agriculture, Cooperation & Farmers Welfare for looking into the budgetary commitments of various schemes of DAC & FW, bringing gender concerns on to the centre stage in all aspects of public expenditure and policy and ensuring a proportionate flow of the public expenditure benefiting women farmers. Nodal officers/ Gender Coordinators in various Divisions have been sensitized about the concept of gender budgeting. Formats of all the beneficiary oriented schemes of the Ministry are being revised to generate gender disaggregated data.

18.5 Current Initiatives of NGRCA

- Realizing the need to provide useful information on Gender Friendly Tools (GFT)/ Equipment used in crop production, crop processing, post-harvest management and other allied sectors generated by various research and development organizations at one

source, an attempt has been made to collect requisite details from various institutes viz. Central Institute for Agricultural Engineering (CIAE), Bhopal; Central Institute for Women in Agriculture (CIWA), Bhubaneshwar, Odisha; AICRP Centers on Home Science; College of Home Science and State Agricultural Universities and collate it. Accordingly, "Compendium on Gender Friendly Tools/Equipments" carrying information on 69 tools/ equipment has been prepared in Hindi and English and is under circulation among all States/UTs through SAMETIs, EEIs, ICAR Institutes, SAUs, ATMA, KVKs, etc.

- A 'Handbook for Women Farmers' has been developed both in English and Hindi by collating special provisions and package of assistance which women farmers can claim under various on-going Missions/ Sub Missions/ Schemes of DAC & FW, Ministry of Agriculture & Farmers Welfare and other Ministries /Departments . The said Handbook has also been circulated to all States/UTs; State Nodal Officers, ATMA; Extension Education Institutes (EEIs) and SAMETIs of all States. It is expected that the team of committed extension functionaries available at State, District and Block Level under ATMA/Schemes/ Programmes of DAC & FW set up will not only make women aware of such interventions but would also facilitate them to derive full benefits of the tailor made provisions for them.
- Gender Sensitization Modules (GSM) developed for sensitization of extension functionaries at Senior, Middle and at Cutting edge level has been condensed to half a day module for its delivery through all ongoing programmes being organized at various Training institutes. MANAGE has developed this 'Capsule Module on Gender Learning' focusing on the practical tips of Gender Budgeting and Accounting duly roping in the recommendations and the outcome of several Action Research Studies undertaken by NGRCA as well as policy decisions of Ministry of Finance, Ministry of Women and Child Development and Ministry of Agriculture & Farmers Welfare.
- The Intermediate Status Report for the Study on "Adoption of Gender Friendly Tools by women farmers and its impact on their lives" and Study on 'Scheme for Improving Women Farmers Access to Extension Services and Gender Mainstreaming in Agriculture' has been submitted by the agencies.
- Revision of guidelines of various Beneficiary Oriented Schemes/ Programmes/Missions of DAC & FW to ensure allocation of resources and flow of benefits to the women farmers in proportion to their participation in agricultural activities.
- Preparation of separate chapter on 'Gender Perspective in Agriculture' for the Annual Report and Gender Related Write-up for the Out Come Budget of DAC & FW, Ministry of Agriculture & Farmers Welfare.
- Collation of special provisions and package of assistance available for women farmers under various on-going Missions/ Sub-Missions/ Schemes of DAC & FW, Ministry of Agriculture & Farmers Welfare and even those of other Ministries /Departments.
- Joint circular for Convergence of National Rural Livelihoods Mission (NRLM), MORD and the Schemes / Programmes / Missions of Department of Agriculture, Cooperation and Farmers Welfare, MoA&FW issued to the States/UTs
- Gender desegregated Data is being maintained under all activities of Sub Mission on Agricultural Extension

(SMAE) of National Mission on Agricultural Extension and Technology (NMAET)

- Coordination with various divisions of Ministry from time to time to collate gender disaggregated data on flow of benefits (physical as well as financial) to women farmers, SC/ST farm women under various schemes and strategies being adopted by the divisions for empowerment of women.

Mainstreaming efforts of various Divisions of DAC & FW:

18.6 The important mainstreaming and gender budgeting efforts within various subject matter divisions are briefly given in the succeeding paragraphs.

(A) Horticulture

18.7 Under Mission for Integrated Development of Horticulture (MIDH), an umbrella scheme on horticulture by subsuming other ongoing schemes on horticulture i.e. National Horticulture Mission(NHM), Horticulture Mission for North East & Himalayan States (HMNEH), National Bamboo Mission (NBM) now re-named as National Agro Forestry & Bamboo Mission, Central Sector Scheme of National Horticulture Board (NHB), Coconut Development Board (CDB) and Central Institute for Horticulture (CIH), Nagaland, all categories of farmers including women beneficiaries are covered in components like floriculture, beekeeping etc.

18.8 MIDH aims at promoting holistic growth of the horticulture sector through area based regionally differentiated strategies. Keeping in view the overall objectives and as per directives of Planning Commission, all the implementing agencies viz. State Horticulture Missions and National Level Agencies have been directed to ensure that at least 30% of budget allocation is earmarked for women beneficiaries while giving subsidies as well as for imparting training for skills development to women farmers for growing horticulture crops and post-harvest management. Women beneficiaries such as women farmers, women Self Help Groups, women entrepreneurs are encouraged to obtain the benefits of the schemes under the Mission.

Directorate of Cashewnut and Cocoa Development, Kochi

18.9 The Directorate of Cashewnut and Cocoa Development is implementing cashew & cocoa development programmes viz. New plantation development, replanting / rejuvenation of cashew plantations, front line technology demonstrations, upgradation of nursery infrastructure, strengthening of data base, human resource development, publicity for crop promotion under MIDH for the development of these two crops during 2016-17. The DCCD acts as a nodal agency for accreditation of existing cashew / cocoa nurseries. The Salient achievements under the scheme during 2016-17 are as follows:

- Under the scheme for new plantation development, an additional area of 1200 ha under cashew and 1700 ha under cocoa with clones of high yielding varieties has been covered. Around 3000 farmers benefitted under the scheme out of which 30% are to be women beneficiaries.
- Under the HRD programme, 1000 unemployed women are proposed to be given training on preparation of value added edible products from cashew apple.
- The Directorate has prepared citizen / client charter in line with performance monitoring and evaluation system and sevottam guidelines to place in the website.

Directorate of Arecanut and Spices Development, Calicut

18.10 The Directorate of Arecanut and Spices Development (DASD) is a subordinate office under Ministry of Agriculture & Farmers Welfare, Government of India, to look after the development of Spices, Arecanut, Betel vine and Aromatic plants at National level. The Directorate monitors the development programmes implemented by the States in spices under MIDH. The Directorate is directly involved in the production and distribution of quality planting material of high yielding varieties of spices and technology transfer programmes in association with State Agricultural Universities and Central Institutes across the country. During the year 2016-17, the Directorate had an outlay of Rs. 11 crores under MIDH. DASD has launched a website www.spicenurseries.in to create a platform where both the nursery owners and farmers including women farmers are brought together to share the information on availability of planting material. To improve the quality of planting material disbursed through various nurseries, the Directorate is also engaged in accreditation of nurseries.

Central Institute of Horticulture, Medziphema, Nagaland

18.11 Central Institute of Horticulture was established in the year 2006 for the holistic development of horticulture sector in the North East Region. The main thrust areas of the Institute are refinement and demonstration of identified technologies pertaining to the region; production and supply of quality seed and planting material; training and capacity building of state govt. officials, field functionaries and farmers on different aspects of horticulture development including organic farming, monitoring of centrally sponsored programmes in the area of horticulture, post harvest management, processing, value addition, marketing and agribusiness promotion. The salient achievements of the institute during 2016-17 are as below-

03 numbers of the courses were undertaken during the period on following subjects for rural youth and women to promote the entrepreneurship development in the field of horticulture in NE region.

- Certificate Course on Protected Cultivation of Horticulture Crops.
- Certificate course on Organic Farming and Certification.
- Certificate course on Nursery Management

(B) Agricultural Extension

- The Sub-Mission on Agricultural Extension (SMAE) which encompasses both- the Centrally Sponsored and Central Sector extension outreach and ICT initiatives, under its "Support to State Extension Programmes for Extension Reforms" (ATMA intervention) supports gender concern to a great extent. This initiative is under implementation in 652 districts of 29 States and 3 UTs. ATMA programme provides that minimum 30% resources are to be utilized for women farmers. To improve participation of women in planning & decision making process, the scheme provides for representation and active involvement of women at various decision making platforms viz. ATMA-Governing Body and ATMA-Management Committee at district level & Farmer Advisory Committees (FACs) at Block, District and State level. Besides, scheme provides for enhanced involvement of women as 'Farmer Friend' in a mechanism promoted under the scheme for Farmer-to-Farmer extension. Farm Women's Food and nutritional Security Groups (FIGs)

@ at least 3 per block are to be formed annually for ensuring household food and nutritional security providing assistance of Rs.10,000/ per group.

- Inclusion of one *'Gender Coordinator'* in every State in the team of committed extension personnel being supported under the Scheme. The role of Gender Coordinator is to ensure flow of support viz. training/ capacity building and extension support as per the specific requirements of women farmers through a strategy suited to their needs.
- Since inception of the Scheme in 2005-06, total **96,37,720 farm women** (**26.60%** of the total benefited farmers) have participated in farmer oriented activities like Exposure Visits, Training, Demonstrations & Kisan Melas including 6,31,572 women farmers benefited during 2016-17 (up to 30th November, 2016).

18.12 The Central Sector Scheme **"Establishment of Agri-Clinics & Agri-Business Centres (ACABC)"** was implemented since April, 2002 with the aim to supplement the efforts of public extension, support agricultural development and create gainful self-employment opportunities to unemployed youths (including women) with qualification in agriculture and allied sectors.

18.13 The scheme promotes involvement of agri-preneurs (including women) trained under the ACABC scheme in providing advisory and extension services to the farmers in agriculture and allied areas. The agri-preneurs trained under ACABC scheme are actively engaged in providing advisory and extension services to the farmers on various technologies.

18.14 The National Institute of Agricultural Extension Management (MANAGE), Hyderabad is the implementing agency for training component under the scheme through a network of identified Nodal Training Institutes (NTIs) in various States and NABARD is implementing the subsidy component on behalf of Government of India and is monitoring credit support to agri-preneurs through commercial banks.

18.15 Under ACABC scheme, the training is imparted to unemployed candidates who possess degree/ diploma in agriculture and allied subjects, intermediate in agriculture and science graduates with PG in agri related courses through selected Nodal Training Institutes (NTIs) in various parts of the country. The NTIs also provide hand-holding to the trained candidates for establishment of agri-ventures in agriculture and allied areas and facilitates in providing loan assistance from banks and subsidy support from NABARD.

18.16 There is a provision of credit linked back-ended upfront composite subsidy on the bank loan availed by trained candidates under the Scheme. The subsidy is 44% in respect of women, SC/ST and all categories of candidates from North-Eastern and Hill States and 36% in respect of other categories. The subsidy is admissible for loans upto Rs.20 lakh in case of individual and Rs.100 lakh in case of Group Projects (for ventures set up by a group of 5 trained candidates).

18.17 During the current year 1930 candidates were trained and 561 have established their ventures of which 188 and 29 are women. Since inception of the scheme, 49745 candidates have been trained and 21010, agri-ventures have been established in the country till 31.10.2016. Out of these 3706 and 1150 are women.These ventures are acting as active supplementary institutions

for Government Extension mechanism in the states.

Details of the scheme may be seen at website www.agriclinics.net

(C) Crops

18.18 The scheme 'National Food Security Mission (NFSM)' was launched in October 2007-08, targeting three important food grain crops viz. rice, wheat and pulses. Cabinet Committee of Economic Affairs has cleared the continuation of NFSM programme in the XII Plan with addition of coarse cereals and commercial crops viz. cotton, jute and sugarcane. At the stage of Administrative approval, 30% of budgetary allocation is earmarked for women beneficiaries/farmers. Concerned Implementing Agencies are responsible for monitoring implementation of these components, i.e., allocation of resources for SC/ST/Women beneficiaries.

18.19 Component wise financial provision for promotion of farmer producer organizations (FPOs) and marketing support for value chain integration includes marketing support to unregistered farmer groups, SHGs, SHG federation etc. for local marketing of pulses and millets (@Rs.2.00 lakh per group of 15 farmers, for one time support only) . This provision enables unregistered farmer groups, SHGs of women and others who wish to collaborate informally for direct marketing pulses in local haats, townships and region.

(D) Agricultural Marketing

18.20 In order to provide single window approach and user & investment friendly atmosphere, all the ongoing six Plan Schemes implemented during the XI Plan period have been put under one umbrella **"Integrated Scheme for Agricultural Marketing (ISAM).** It aims to (i) promote agri-marketing through creation of marketing and agribusiness infrastructure including storage, (ii)incentivize agri-market reforms, (iii) provide market linkages to farmers, (iv) provide access to agri-market information and (v) support quality certification of agriculture commodities. ISAM scheme is having five components, namely: (i) Agricultural Marketing Infrastructure (AMI), the objective of this component is to create market infrastructure including Storage Infrastructure and Integrated Value Chain Projects (IVC) [the schemes of Grameen Bhandaran Yojana (GBY) and Development/ Strengthening of Agricultural Marketing Infrastructure, Grading and Standardization (AMIGS) has been merged as AMI] (ii) Marketing Research and Information Network (MRIN) (iii) Strengthening of Agmark Grading Facilities (SAGF), (iv) Agri-Business Development (ABD) through Venture Capital Assistance (VCA) and Project Development Facility (PDF) and (v) Training, Research and Consultancy through Choudhary Charan Singh National Institute of Agriculture Marketing (NIAM).

18.21 Under Agricultural Marketing Infrastructure (AMI) components, Women are eligible for subsidy @ 33.33% as against 25% for others.

(E) Mechanization and Technology

18.22 Taking into consideration all the factors and challenges coming in the progress of Farm Mechanization, Department of Agriculture, Cooperation & Farmers Welfare, Mechanization and Technology Division has formulated a Sub Mission on Agricultural Mechanization (SMAM) under the main Mission i.e. National Mission on Agricultural Extension and Technology (NMAET) which is under implementation w.e.f. 2014-15. The components of SMAM are as follows:

i. Promotion and Strengthening of Agricultural Mechanization

through Training, Testing and Demonstration

ii. Demonstration, Training and distribution of Post Harvest Technology and Management (PHTM)

iii. Financial Assistance for Procurement of Agriculture Machinery and Equipment: Promotes ownership of various agricultural machinery & equipments

iv. Establish Farm Machinery Banks for Custom Hiring

v. Promotion of farm mechanisation in selected villages

vi. Financial Assistance for promotion of Mechanized operations/hectare carried out through custom hiring Centres

vii. Promotion of farm machinery and equipment in north eastern region

18.23 The SMAM provides a suitable platform for converging all activities for inclusive growth of agricultural mechanization by providing a 'single window' approach for implementation with a special focus on women farmers with the following provisions:

18.24 State Governments have been advised to ensure 30% of allocation under the scheme belongs to women beneficiary and also to furnish reports separately in SMAM guidelines.

- 10% more assistance for women beneficiary to procure Agricultural Machinery, implements and equipment including PHT under component 2 and 3.

- In order to reduce the drudgery and increasing efficiency in farm operations, number of agricultural implements and hand tools suitable for farm women has been developed by Research & Development organizations under ICAR. The list of gender friendly equipment has been sent to all States/ UTs for popularizing them through various schemes of Government.

18.25 Gender Friendly Equipment for Women: Under the component 1 of SMAM, Agricultural Mechanization through Training, Testing, and Demonstration, a total 1949 women were trained during the current Financial Year 2016-17 (till October. 2016). A list of about 30 identified gender-friendly tools and equipment developed by the Research and Development Organization for use in different farm operations has already been sent to all States and UTs for popularizing them. State Governments have been directed to earmark 30 per cent of total funds allocated under SMAM for women beneficiaries.

Institute wise women's trainees trained

Sl. No.	Institute	Women's trainees trained
1	CFMT&TI Budni	724
2	NRFMT&TI Hisar	255
3	SRFMT&TI Garladinne	780
4	NERFMT&TI BiswanathCharialli	190
	**Total**	**1949**

(F) Seeds

18.26 Sub-Mission for Seeds and Planting Material (SMSP) under National Mission on Agricultural Extension & Technology (NMAET) which is being implemented w.e.f. 01.04.2014 with the view to develop/ strengthen seed sector and to enhance production and multiplication of high yielding certified/ qualify seeds of all agricultural crops and making it available to the farmers at affordable prices and also place an effective system for protection of plant varieties, rights of farmers and plant breeders to encourage development of new varieties of plants. Scheme is also for **Women farmers**. States/implementing agencies are requested to allocate adequate funds and

ensure participation of women. However, no special budgetary provisions for women have been made in the Sub-Mission.

(G) Cooperation

18.27 NCDC encourages women cooperatives to avail assistance under its various schemes. Prominent among the women's co-operatives assisted by NCDC are in the sectors of Food Grain Processing, Oil Seeds Processing, Fisheries, Dairy & Livestock, Spinning Mills, Plantation Crops, etc.

18.28 Women cooperatives are covered under Weaker Section Programme for the purpose of availing subsidy and concessional funding during the remaining period of the 12th Five Year Plan programme under **Central Sector Integrated Scheme on Agricultural Cooperation.**

18.29 Cumulatively, as on 31.03.2016 NCDC has sanctioned and released financial assistance of Rs. 193.54 crores and Rs. 96.41 crores respectively for the development of cooperative societies exclusively promoted by women. During the year 2015-16, NCDC sanctioned RS. 2.04 crores to 75 Unit under Consumer and ICDP programmes and released Rs. 3.55 crores to women co-operative societies under ICDP programme. In the 2815 projects/units sanctioned by NCDC in the year 2015-16, 11.98 lakh women are enrolled as members, out of which 369 women members are on the Board of Directors of different Cooperatives.

(H) Policy

18.30 The National Policy for Farmers 2007 announced by the Government envisages the following measures aimed at women's empowerment:

- Asset reforms under land, water and livestock for an equitable share to women farmers.
- Better access to inputs and services, science and technology, implements, credit and support services like creches, child care centres, nutrition, health and training.
- Encouragement to women for participating in group activities aimed at achieving economies of scale through farming groups.
- Involvement of women in conservation and development of bio-resources.

18.31 The Policy provisions, inter alia, include asset reforms in respect of land, water, livestock, fisheries, and bio-resources; support services and inputs like application of frontier technologies; agricultural bio-security systems; supply of good quality seeds and disease-free planting material, improving soil fertility and health, and integrated pest management systems; support services for women like crèches, child care centers, nutrition, health and training; timely, adequate, and easy reach of institutional credit at reasonable interest rates, and farmer-friendly insurance instruments; use of Information and Communication Technology (ICT) and setting up of farmers' schools to revitalize agricultural extension; effective implementation of MSP across the country, development of agricultural market infrastructure, and rural non-farm employment initiatives for farm households; integrated approach for rural energy, etc.

18.32 Many of the provisions of the NPF are being operationalised through various schemes and programmes which are being implemented by different Central Government Departments and Ministries. For the operationalisation of the remaining provisions of the Policy, an Action Plan has been finalized and circulated to the Ministries and Department concerned, as well as to all States and UTs for necessary follow up action. An Inter-Ministerial Committee constituted

for the purpose also monitors the progress of the Plan of Action for the operationalisation of the NPF.

(I) Agriculture Census

18.33 Recognizing the predominance of agriculture sector in the economy, collection and maintenance of data relating to agricultural holdings assumes vital importance. Agriculture Census is conducted on quinquennial basis to collect information related to structural characteristics of operational holdings in the country. As part of World Agriculture Census (WCA) programme, first comprehensive Agriculture Census was carried out in the country with agricultural year 1970-71 as the reference period. The current Agriculture Census 2015-16 is tenth in the series.

18.34 Gender based data in Agriculture Census is being collected since 1995-96 following recommendations of the Central Statistics Office, Ministry of Statistics and Programme Implementation, New Delhi. The scope of collection of gender based data has been restricted to number of operational holdings, corresponding operated area by different size classes of holdings, social group (SC, ST and others), and types of holdings (individual, joint and institutional).

Percentage of female operational holdings as per results of various Agriculture Censuses is given in the following table.

Sr. No.	Size Group	2000-01*	2005-06*	2010-11
1	Marginal (Below 1.00 ha.)	11.84	12.60	13.63
2	Small (1.00-2.00 ha.)	10.27	11.10	12.15
3	Semi-Medium (2.00-4.00 ha.)	8.67	9.61	10.45
4	Medium (4.00-10.00 ha.)	6.863	7.77	8.49
5	Large (Above 10.00 ha.)	5.22	6.00	6.78
	**All Size Groups**	**10.83**	**11.70**	**12.78**

* Excludes Jharkhand

18.35 Increase in percentage of female operational holders during different Agriculture Censuses indicates participation of more and more women in operation and management of agricultural holdings in the country. A brief summary of flow of benefits to women under various schemes/ programmes of the DAC&FW is given in **Annexure- 18.1**.

The Way Forward:

18.36 Realizing that it is 'Gender' that differentiates the roles, responsibilities, resources, constraints and opportunities of women and men in agriculture, precise gender information is the need of the hour. Incorporating gender into agricultural development will lead to:

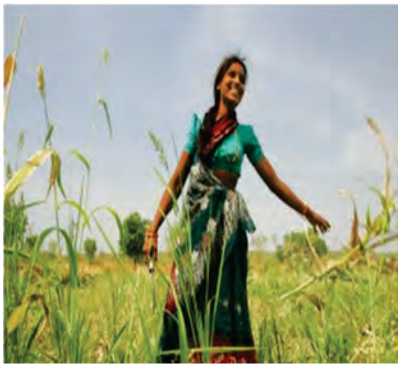

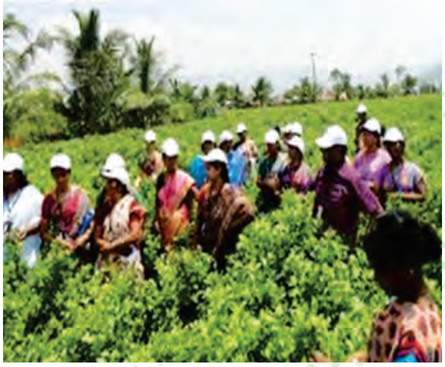

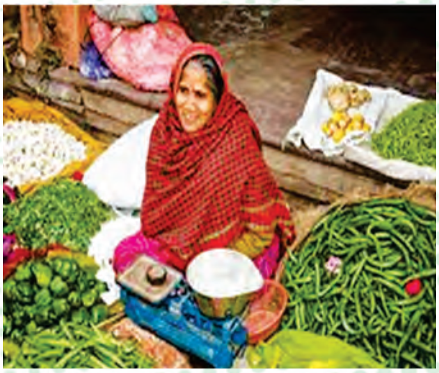

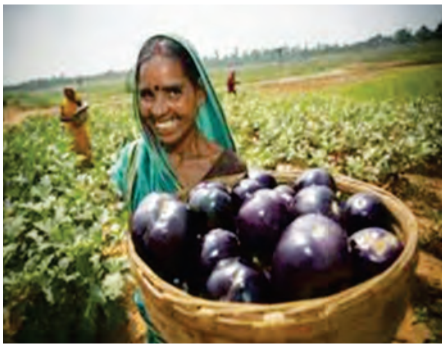
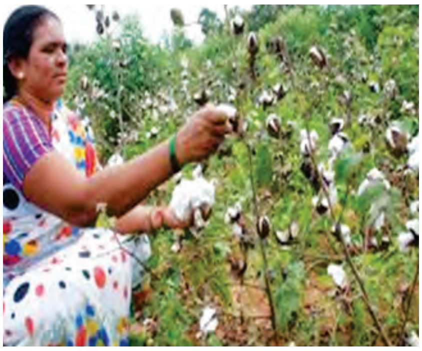
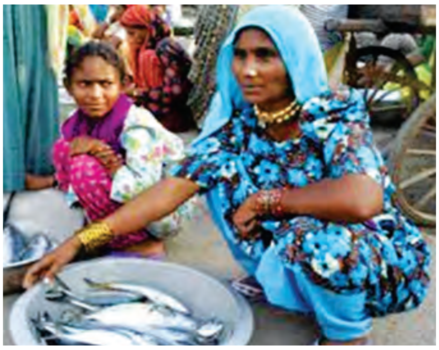

- Building inherent strength of women and men to mutually learn;
- Overcoming gender based prejudices; and
- Articulating gender perspectives in development activities

18.37 In line with the National Agricultural Policy-2000 and provisions under National Policy for Farmers-2007, the strategy of the Government is to focus on farmers' welfare by making farming viable both for men and women. This may improve farm women's access to productive resources including agricultural extension services thereby bringing overall improvement in the lives of rural women.This may not only enhance the production and productivity of agricultural sector and improve overall national food security but would also smoothen the transition of women from being beneficiaries of the programmes& Schemes to their active participation in shaping the empowerment.

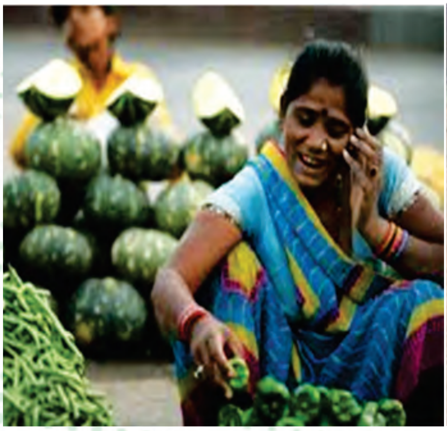
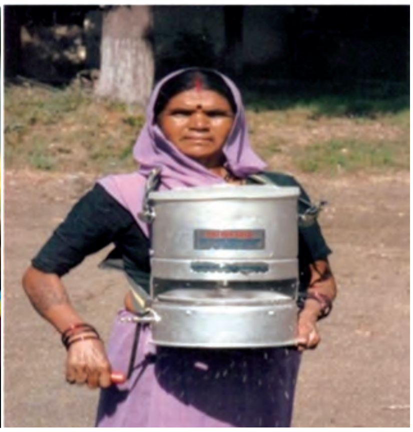
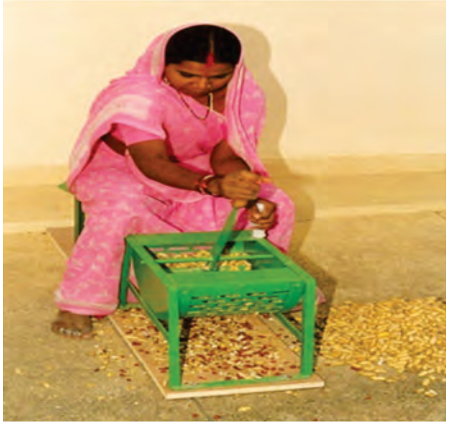

Annexure – 2.1

CHAPTER - 2
Functions and Organizational Structure

List of Functional Divisions in the Department Of Agriculture, Cooperation and Farmers Welfare

Divisions:

1. Administration
2. Agricultural Census
3. Agricultural Marketing
4. Budget, Finance and Accounts
5. Credit
6. Cooperation
7. Crops
8. Drought Management
9. Economic Administration
10. Extension
11. General Coordination
12. Official Language
13. Horticulture
14. Information Technology
15. Integrated Nutrients Management
16. International Cooperation
17. Mechanization and Technology
18. Natural Resource Management
19. Plant Protection
20. Policy
21. Plan Coordination
22. Rashtriya Krishi Vikash Yojna (RKVY)
23. Rainfed Farming System
24. Seeds
25. Oil Seeds
26. Trade
27. Vigilance

Annexure – 2.2

CHAPTER - 2
Functions and Organizational Structure

Inventory of Field Formations

I. Attached Offices

1. Directorate of Economics & Statistics, Shastri Bhavan, 'B' Wing, New Delhi.
2. Commission for Agricultural Costs and Prices, Shastri Bhavan, 'F' Wing, Second Floor, New Delhi.
3. Directorate of Plant Protection, Quarantine and Storage, N.H.IV, Faridabad (Haryana).
4. Directorate of Marketing & Inspection, N.H.IV, Faridabad (Haryana).
5. Mahalanobis National Crop Forecast Centre, New Delhi.

II. Sub-Ordinate Offices

1. Central Farm Machinery Training & Testing Institute, Budni (Madhya Pradesh).
2. Northern Region Farm Machinery Training & Testing Institute, Hissar (Haryana).
3. Southern Region Farm Machinery Training & Testing Institute, Garladinne, Distt. Anantapur (A.P).
4. North Eastern Region Farm Machinery Training & Testing Institute, Biswnath Chariali, Distt. Sonitpur (Assam).
5. Directorate of Cotton Development, Bhoomi Sarvekshan Bhawan, Near Centre Point School, Seminary Hills, Katol Road, Nagpur, Maharashtra-440013.
6. Directorate of Jute Development, 234/4, Acharya Jagdish Bose Road, Nizam Palace Campus, Kolkata- 700020 (West Bengal).
7. Directorate of Millets Development, Mini Secretariat Building, Room No. 710, 6th Floor, Bani Park, Jaipur (Rajasthan).
8. Directorate of Sugarcane Development, 8th Floor, Hall No.3, Kendriya Bhavan, Aliganj, Lucknow - 226024 (U.P).
9. Directorate of Rice Development, 191, Patliputra Colony, Patna-800013 (Bihar).
10. Directorate of Wheat Development, CGO Building, Hapur Road Chauraha, Kamla Nehru Nagar, Ghaziabad (U.P).
11. Directorate of Extension, Krishi Vistar Bhavan, Dr. K.S. Krishna Marg, IARI Campus, Pusa, New Delhi-110 012.
12. Directorate of Oilseeds Development, Telhan Bhavan, Himayat Nagar, Hyderabad (A.P).
13. Directorate of Pulses Development, 8th Floor, Vindhyachal Bhavan, Bhopal-462004 (M.P).
14. Central Fertiliser Quality Control & Training Institute, N.H.IV, Faridabad (Haryana).
15. National Centre of Organic Farming, C.G.O. Complex, Kamla Nehru Nagar, Hapur Road Chungi, Ghaziabad (U.P).
16. Directorate of Cashewnut and Cocoa Development, M.G. Road, Kochi-682011 (Kerala).

17. Directorate of Arecanut and Spices Development, Cannanore Road, Kozhikode-673005 (Kerala).
18. Office of the Minister (Agriculture), Embassy of India, ROME (ITALY).
19. All India Soil and Land Use Survey, IARI Campus, Pusa, New Delhi-110 012.
20. National Seed Research & Training Centre (NSRTC) VARANASI (U.P.).
21. Central Institute of Horticulture, Medziphema, Nagaland.

Annexure – 2.3

1. Public Sector Undertaking

1. National Seeds Corporation, New Delhi.

2. Autonomous Bodies

1. Coconut Development Board, Kochi (Kerala).
2. National Horticulture Board, Gurgaon (Haryana).
3. National Cooperative Development Corporation, New Delhi.
4. Small Farmers' Agri Business Consortium, New Delhi.
5. National Institute for Agricultural Extension Management, Hyderabad (A.P).
6. National Institute of Agricultural Marketing, Jaipur (Rajasthan).
7. National Institute of Plant Health Management, Hyderabad (A.P.)
8. National Centre for Cold Chain Development, Nirman Bhawan, New Delhi.

3. National Level Cooperative Organisations

1. National Cooperative Union of India, New Delhi.
2. National Agricultural Cooperative Marketing Federation of India Limited, New Delhi.
3. National Federation of Cooperative Sugar Factories Ltd., New Delhi.
4. National Heavy Engineering Cooperative Ltd., Pune (Maharashtra).
5. National Federation of Urban Cooperative Banks and Credit Societies Ltd., New Delhi.
6. The All India Federation of Cooperative Spinning Mills Ltd., Mumbai (Maharashtra).
7. National Cooperative Agriculture and Rural Development Banks Federation Ltd., Mumbai (Maharashtra).
8. National Federation of State Cooperative Banks Ltd., Navi Mumbai (Maharashtra).
9. National Federation of Labour Cooperatives Ltd., New Delhi.
10. National Cooperative Tobacco Growers' Federation Ltd., Anand (Gujarat).

4. Auhtorities

1. Protection of Plant Varieties and Farmers' Rights Authority, NASC Complex, DPS Marg Opp. Todapur, Delhi-110012.
2. National Rainfed Area Authority, NASC Complex, Dev Prakash Shastri Marg, Pusa, New Delhi- 110012.

Annexure-3.1

As on 2nd August, 2016

Agricultural Statistics Division
Directorate of Economics & Statistics
Department of Agriculture, Cooperation and Farmers welfare
Fourth Advance Estimates of Production of Foodgrains for 2015-16

Million Tonnes

Crop	Season	2003-04	2004-05	2005-06	2006-07	2007-08	2008-09	2009-10	2010-11	2011-12	2012-13	2013-14	2014-15		2015-16	
													4th Advance Estimates	2014-15 (Final)	Targets	4th Advance Estimate s
Rice	Kharif	78.62	72.23	78.27	80.17	82.66	84.91	75.92	80.65	92.78	92.37	91.50	90.86	91.39	92.10	91.31
	Rabi	9.91	10.90	13.52	13.18	14.03	14.27	13.18	15.33	12.52	12.87	15.15	13.94	14.09	14.00	13.01
	Total	88.53	83.13	91.79	93.36	96.69	99.18	89.09	95.98	105.30	105.24	106.65	104.80	105.48	106.10	104.32
Wheat	Rabi	72.16	68.64	69.35	75.81	78.57	80.68	80.80	86.87	94.88	93.51	95.85	88.94	86.53	94.75	93.50
Jowar	Kharif	4.84	4.04	4.07	3.71	4.11	3.05	2.76	3.44	3.29	2.84	2.39	2.01	2.30	3.10	1.71
	Rabi	1.84	3.20	3.56	3.44	3.81	4.19	3.93	3.56	2.69	2.44	3.15	3.04	3.15	2.75	2.70
	Total	6.68	7.24	7.63	7.15	7.93	7.25	6.70	7.00	5.98	5.28	5.54	5.05	5.45	5.85	4.41
Bajra	Kharif	12.11	7.93	7.68	8.42	9.97	8.89	6.51	10.37	10.28	8.74	9.25	9.05	9.18	9.50	8.06
Maize	Kharif	12.73	11.48	12.16	11.56	15.11	14.12	12.29	16.64	16.49	16.19	17.14	16.39	17.01	17.25	15.24
	Rabi	2.25	2.70	2.55	3.54	3.85	5.61	4.43	5.09	5.27	6.06	7.11	7.29	7.16	6.50	6.56
	Total	14.98	14.17	14.71	15.10	18.96	19.73	16.72	21.73	21.76	22.26	24.26	23.67	24.17	23.75	21.81
Ragi	Kharif	1.97	2.43	2.35	1.44	2.15	2.04	1.89	2.19	1.93	1.57	1.98	2.00	2.06	1.80	1.79
Small Millets	Kharif	0.56	0.48	0.47	0.48	0.55	0.44	0.38	0.44	0.45	0.44	0.43	0.37	0.39	0.52	0.37
Barley	Rabi	1.30	1.21	1.22	1.33	1.20	1.69	1.35	1.66	1.62	1.75	1.83	1.60	1.61	1.78	1.51
Coarse Cereals	Kharif	32.22	26.36	26.74	25.61	31.89	28.54	23.83	33.08	32.44	29.79	31.20	29.82	30.94	32.17	27.17
	Rabi	5.39	7.10	7.33	8.31	8.86	11.49	9.72	10.32	9.58	10.25	12.09	11.93	11.92	11.03	10.77
	Total	37.60	33.46	34.07	33.92	40.75	40.04	33.55	43.40	42.01	40.04	43.29	41.75	42.86	43.20	37.94

Crop	Season	2003-04	2004-05	2005-06	2006-07	2007-08	2008-09	2009-10	2010-11	2011-12	2012-13	2013-14	2014-15		2015-16	
													4th Advance Estimates	2014-15 (Final)	Targets	4th Advance Estimate s
Cereals	Kharif	110.84	98.59	105.01	105.78	114.55	113.45	99.75	113.73	125.22	122.16	122.70	120.68	122.34	124.27	118.48
	Rabi	87.45	86.64	90.21	97.30	101.46	106.45	103.70	112.52	116.98	116.63	123.09	114.81	112.53	119.78	117.28
	Total	198.28	185.23	195.22	203.08	216.01	219.90	203.45	226.25	242.20	238.79	245.79	235.49	234.87	244.05	235.76
Tur	Kharif	2.36	2.35	2.74	2.31	3.08	2.27	2.46	2.86	2.65	3.02	3.17	2.78	2.81	3.67	2.46
Gram	Rabi	5.72	5.47	5.60	6.33	5.75	7.06	7.48	8.22	7.70	8.83	9.53	7.17	7.33	9.50	7.17
Urad	Kharif	1.20	0.95	0.90	0.94	1.12	0.84	0.81	1.40	1.23	1.43	1.15	1.27	1.28	1.29	1.39
	Rabi	0.27	0.38	0.35	0.50	0.34	0.33	0.42	0.36	0.53	0.47	0.55	0.60	0.68	0.52	0.81
	Total	1.47	1.33	1.25	1.44	1.46	1.17	1.24	1.76	1.77	1.90	1.70	1.87	1.96	1.81	2.20
Moong	Kharif	1.43	0.81	0.69	0.84	1.25	0.78	0.44	1.53	1.24	0.79	0.96	0.85	0.87	1.10	1.02
	Rabi	0.28	0.25	0.26	0.28	0.27	0.26	0.25	0.27	0.40	0.40	0.65	0.66	0.64	0.61	0.59
	Total	1.70	1.06	0.95	1.12	1.52	1.03	0.69	1.80	1.63	1.19	1.61	1.51	1.50	1.71	1.60
Other Kharif Pulses	Kharif	1.18	0.61	0.54	0.70	0.96	0.80	0.49	1.33	0.93	0.62	0.71	0.73	0.77	0.99	0.67
Other Rabi Pulses	Rabi	2.48	2.32	2.31	2.29	2.00	2.23	2.31	2.27	2.40	2.73	2.53	3.14	2.77	2.37	2.37
Total Pulses	Kharif	6.16	4.72	4.86	4.80	6.40	4.69	4.20	7.12	6.06	5.91	5.99	5.63	5.73	7.05	5.54
	Rabi	8.74	8.41	8.52	9.40	8.36	9.88	10.46	11.12	11.03	12.43	13.25	11.57	11.42	13.00	10.93
	Total	14.91	13.13	13.38	14.20	14.76	14.57	14.66	18.24	17.09	18.34	19.25	17.20	17.15	20.05	16.47
Total Foodgrains	Kharif	117.00	103.31	109.87	110.58	120.96	118.14	103.95	120.85	131.27	128.07	128.69	126.31	128.06	131.32	124.01
	Rabi	96.19	95.05	98.73	106.71	109.82	116.33	114.15	123.64	128.01	129.06	136.35	126.38	123.96	132.78	128.21
	Total	213.19	198.36	208.60	217.28	230.78	234.47	218.11	244.49	259.29	257.13	265.04	252.68	252.02	264.10	252.22

\# Lakh bales of 170 kgs. each

\# # Lakh bales of 180 kgs. each

As on 2nd August, 2016

Agricultural Statistics Division
Directorate of Economics & Statistics
Department of Agriculture, Cooperation and Farmers welfare
Fourth Advance Estimates of Production of Commercial Crops for 2015-16

Lakh Tonnes

Crop	Season	2003-04	2004-05	2005-06	2006-07	2007-08	2008-09	2009-10	2010-11	2011-12	2012-13	2013-14	2014-15		2015-16	
													4th Advance Estimates	2014-15 (Final)	Targets	4th Advance Estimates
Groundnut	Kharif	68.60	52.62	62.98	32.94	73.62	56.17	38.52	66.43	51.27	31.87	80.58	50.75	59.30	62.53	53.40
	Rabi	12.67	15.12	16.95	15.69	18.20	15.51	15.76	16.22	18.37	15.08	16.56	14.82	14.71	18.99	14.30
	Total	81.27	67.74	79.93	48.64	91.83	71.68	54.28	82.65	69.64	46.95	97.14	65.57	74.02	81.52	67.71
Castorseed	Kharif	7.97	7.93	9.91	7.62	10.54	11.71	10.09	13.50	22.95	19.64	17.27	17.33	18.70	20.34	16.50
Sesamum	Kharif	7.82	6.74	6.41	6.18	7.57	6.40	5.88	8.93	8.10	6.85	7.15	8.11	8.28	7.61	8.66
Nigerseed	Kharif	1.09	1.12	1.08	1.21	1.10	1.17	1.00	1.08	0.98	1.02	0.98	0.73	0.76	1.19	0.77
Rapeseed & Mustard	Rabi	62.91	75.93	81.31	74.38	58.34	72.01	66.08	81.79	66.04	80.29	78.77	63.09	62.82	81.09	68.21
Linseed	Rabi	1.97	1.70	1.73	1.68	1.63	1.69	1.54	1.47	1.52	1.49	1.41	1.53	1.55	1.90	1.32
Safflower	Rabi	1.35	1.74	2.29	2.40	2.25	1.89	1.79	1.50	1.45	1.09	1.13	0.96	0.90	1.83	0.64
Sunflower	Kharif	3.06	4.31	4.56	3.66	4.63	3.57	2.14	1.92	1.47	1.87	1.54	1.06	1.11	1.91	0.68
	Rabi	6.24	7.56	9.83	8.62	10.00	8.01	6.36	4.59	3.69	3.57	3.50	3.09	3.23	6.18	2.63
	Total	9.30	11.87	14.39	12.28	14.63	11.58	8.51	6.51	5.17	5.44	5.04	4.15	4.34	8.09	3.31
Soyabean	Kharif	78.18	68.76	82.74	88.51	109.68	99.05	99.64	127.36	122.14	146.66	118.61	105.28	103.74	126.43	85.92

Crop	Season	2003-04	2004-05	2005-06	2006-07	2007-08	2008-09	2009-10	2010-11	2011-12	2012-13	2013-14	2014-15		2015-16	
													4th Advance Estimates	2014-15 (Final)	Targets	4th Advance Estimates
Total Nine	Kharif	166.72	141.49	167.67	140.12	207.13	178.08	157.28	219.22	206.91	207.91	226.12	183.26	191.89	220.01	165.93
Oilseeds																
	Rabi	85.14	102.04	112.11	102.77	90.42	99.11	91.53	105.57	91.08	101.52	101.37	83.49	83.21	109.99	87.10
	Total	251.86	243.54	279.78	242.89	297.55	277.19	248.82	324.79	297.99	309.43	327.49	266.75	275.11	330.00	253.04
Cotton #	Total	137.29	164.29	184.99	226.32	258.84	222.76	240.22	330.00	352.00	342.20	359.02	354.75	348.05	351.50	301.47
Jute # #	Total	102.52	93.99	99.70	103.17	102.20	96.34	112.30	100.09	107.36	103.40	110.83	109.34	106.18	110.00	99.38
Mesta # #	Total	9.21	8.73	8.70	9.56	9.90	7.31	5.87	6.11	6.63	5.90	6.07	5.15	5.08	7.00	5.28
Jute & Mesta # #	Total	111.73	102.72	108.40	112.73	112.11	103.65	118.17	106.20	113.99	109.30	116.90	114.49	111.26	117.00	104.66
Sugarcane	Total	2338.62	2370.88	2811.72	3555.20	3481.88	2850.29	2923.02	3423.82	3610.37	3412.00	3521.42	3593.30	3623.33	3550.00	3521.63

\# Lakh bales of 170 kgs. each

\# # Lakh bales of 180 kgs. each

Annexure-3.2

As on 22 .09. 2016

Agricultural Statistics Division
Directorate of Economics & Statistics
Department of Agriculture, Cooperation and Farmers welfare
First Advance Estimates of Production of Foodgrains for 2016-17

Million Tonnes

Crop	Season	2003-04	2004-05	2005-06	2006-07	2007-08	2008-09	2009-10	2010-11	2011-12	2012-13	2013-14	2014-15	2015-16		2016-17	
														1st Advance Estimates	4th Advance Estimates	Targets	1st Advance
1	2	3	4	5	6	7	8	9	10	11	12	13	14	15	16	17	18
Rice	Kharif	78.62	72.23	78.27	80.17	82.66	84.91	75.92	80.65	92.78	92.37	91.50	91.39	90.61	91.31	93.00	93.88
	Rabi	9.91	10.90	13.52	13.18	14.03	14.27	13.18	15.33	12.52	12.87	15.15	14.09		13.01	15.50	
	Total	88.53	83.13	91.79	93.36	96.69	99.18	89.09	95.98	105.30	105.24	106.65	105.48	90.61	104.32	108.50	93.88
Wheat	Rabi	72.16	68.64	69.35	75.81	78.57	80.68	80.80	86.87	94.88	93.51	95.85	86.53		93.50	96.50	
Jowar	Kharif	4.84	4.04	4.07	3.71	4.11	3.05	2.76	3.44	3.29	2.84	2.39	2.30	1.87	1.71	3.00	2.42
	Rabi	1.84	3.20	3.56	3.44	3.81	4.19	3.93	3.56	2.69	2.44	3.15	3.15		2.70	3.00	
	Total	6.68	7.24	7.63	7.15	7.93	7.25	6.70	7.00	5.98	5.28	5.54	5.45	1.87	4.41	6.00	2.42
Bajra	Kharif	12.11	7.93	7.68	8.42	9.97	8.89	6.51	10.37	10.28	8.74	9.25	9.18	8.64	8.06	9.50	8.55
Maize	Kharif	12.73	11.48	12.16	11.56	15.11	14.12	12.29	16.64	16.49	16.19	17.14	17.01	15.51	15.24	17.50	19.30
	Rabi	2.25	2.70	2.55	3.54	3.85	5.61	4.43	5.09	5.27	6.06	7.11	7.16		6.56	7.00	
	Total	14.98	14.17	14.71	15.10	18.96	19.73	16.72	21.73	21.76	22.26	24.26	24.17	15.51	21.81	24.50	19.30
Ragi	Kharif	1.97	2.43	2.35	1.44	2.15	2.04	1.89	2.19	1.93	1.57	1.98	2.06	1.44	1.79	2.00	1.85
Small Millets	Kharif	0.56	0.48	0.47	0.48	0.55	0.44	0.38	0.44	0.45	0.44	0.43	0.39	0.42	0.37	0.50	0.34
Barley	Rabi	1.30	1.21	1.22	1.33	1.20	1.69	1.35	1.66	1.62	1.75	1.83	1.61		1.51	1.85	
Coarse Cereals	Kharif	32.22	26.36	26.74	25.61	31.89	28.54	23.83	33.08	32.44	29.79	31.20	30.94	27.88	27.17	32.50	32.45
	Rabi	5.39	7.10	7.33	8.31	8.86	11.49	9.72	10.32	9.58	10.25	12.09	11.92		10.77	11.85	
	Total	37.60	33.46	34.07	33.92	40.75	40.04	33.55	43.40	42.01	40.04	43.29	42.86	27.88	37.94	44.35	32.45
Cereals	Kharif	110.84	98.59	105.01	105.78	114.55	113.45	99.75	113.73	125.22	122.16	122.70	122.34	118.49	118.48	125.50	126.33

Crop	Season	2003-04	2004-05	2005-06	2006-07	2007-08	2008-09	2009-10	2010-11	2011-12	2012-13	2013-14	2014-15	2015-16		2016-17	
														1st Advance Estimates	4th Advance Estimates	Targets	1st Advance
1	2	3	4	5	6	7	8	9	10	11	12	13	14	15	16	17	18
	Rabi	87.45	86.64	90.21	97.30	101.46	106.45	103.70	112.52	116.98	116.63	123.09	112.53		117.28	13.50	
	Total	198.28	185.23	195.22	203.08	216.01	219.90	203.45	226.25	242.20	238.79	245.79	234.87	118.49	235.76	139.00	126.33
Tur	Kharif	2.36	2.35	2.74	2.31	3.08	2.27	2.46	2.86	2.65	3.02	3.17	2.81	2.61	2.46	3.62	4.29
Gram	Rabi	5.72	5.47	5.60	6.33	5.75	7.06	7.48	8.22	7.70	8.83	9.53	7.33		7.17	9.60	
Urad	Kharif	1.20	0.95	0.90	0.94	1.12	0.84	0.81	1.40	1.23	1.43	1.15	1.28	1.37	1.39	1.45	2.01
	Rabi	0.27	0.38	0.35	0.50	0.34	0.33	0.42	0.36	0.53	0.47	0.55	0.68		0.81	0.70	
	Total	1.47	1.33	1.25	1.44	1.46	1.17	1.24	1.76	1.77	1.90	1.70	1.96	1.37	2.20	2.15	2.01
Moong	Kharif	1.43	0.81	0.69	0.84	1.25	0.78	0.44	1.53	1.24	0.79	0.96	0.87	0.86	1.02	1.22	1.35
	Rabi	0.28	0.25	0.26	0.28	0.27	0.26	0.25	0.27	0.40	0.40	0.65	0.64		0.59	0.65	
	Total	1.70	1.06	0.95	1.12	1.52	1.03	0.69	1.80	1.63	1.19	1.61	1.50	0.86	1.60	1.87	1.35
	Kharif	1.18	0.61	0.54	0.70	0.96	0.80	0.49	1.33	0.93	0.62	0.71	0.77	0.72	0.67	0.96	1.06
Other Rabi Pulses	Rabi	2.48	2.32	2.31	2.29	2.00	2.23	2.31	2.27	2.40	2.73	2.53	2.77		2.37	2.55	
Total Pulses	Kharif	6.16	4.72	4.86	4.80	6.40	4.69	4.20	7.12	6.06	5.91	5.99	5.73	5.56	5.54	7.25	8.70
	Rabi	8.74	8.41	8.52	9.40	8.36	9.88	10.46	11.12	11.03	12.43	13.25	11.42		10.93	13.50	
	Total	14.91	13.13	13.38	14.20	14.76	14.57	14.66	18.24	17.09	18.34	19.25	17.15	5.56	16.47	20.75	8.70
Total Foodgrains	Kharif	117.00	103.31	109.87	110.58	120.96	118.14	103.95	120.85	131.27	128.07	128.69	128.06	124.05	124.01	132.75	135.03
	Rabi	96.19	95.05	98.73	106.71	109.82	116.33	114.15	123.64	128.01	129.06	136.35	123.96		128.21	137.35	
	Total	213.19	198.36	208.60	217.28	230.78	234.47	218.11	244.49	259.29	257.13	265.04	252.02	124.05	252.22	270.10	135.03

Agricultural Statistics Division
Directorate of Economics & Statistics
Department of Agriculture, Cooperation and Farmers welfare
First Advance Estimates of Production of Commercial Crops for 2016-17

As on 22 .09. 2016

Lakh Tonnes

Crop	Season	2003-04	2004-05	2005-06	2006-07	2007-08	2008-09	2009-10	2010-11	2011-12	2012-13	2013-14	2014-15	2015-16		2016-17	
														1st Advance Estimates	4th Advance Estimates	Targets	1st Advance Estimates
Groundnut	Kharif	68.60	52.62	62.98	32.94	73.62	56.17	38.52	66.43	51.27	31.87	80.58	59.30	51.07	53.40	64.30	64.98
	Rabi	12.67	15.12	16.95	15.69	18.20	15.51	15.76	16.22	18.37	15.08	16.56	14.71		14.30	20.70	
	Total	81.27	67.74	79.93	48.64	91.83	71.68	54.28	82.65	69.64	46.95	97.14	74.02	51.07	67.71	85.00	64.98
Castorseed	Kharif	7.97	7.93	9.91	7.62	10.54	11.71	10.09	13.50	22.95	19.64	17.27	18.70	19.44	16.50	22.00	17.31
Sesamum	Kharif	7.82	6.74	6.41	6.18	7.57	6.40	5.88	8.93	8.10	6.85	7.15	8.28	7.95	8.66	9.00	6.75
Nigerseed	Kharif	1.09	1.12	1.08	1.21	1.10	1.17	1.00	1.08	0.98	1.02	0.98	0.76	0.96	0.77	1.15	1.01
Rapeseed & Mustard	Rabi	62.91	75.93	81.31	74.38	58.34	72.01	66.08	81.79	66.04	80.29	78.77	62.82		68.21	85.00	
Linseed	Rabi	1.97	1.70	1.73	1.68	1.63	1.69	1.54	1.47	1.52	1.49	1.41	1.55		1.32	1.60	
Safflower	Rabi	1.35	1.74	2.29	2.40	2.25	1.89	1.79	1.50	1.45	1.09	1.13	0.90		0.64	1.60	
Sunflower	Kharif	3.06	4.31	4.56	3.66	4.63	3.57	2.14	1.92	1.47	1.87	1.54	1.11	1.16	0.68	2.40	1.34
	Rabi	6.24	7.56	9.83	8.62	10.00	8.01	6.36	4.59	3.69	3.57	3.50	3.23		2.63	6.10	
	Total	9.30	11.87	14.39	12.28	14.63	11.58	8.51	6.51	5.17	5.44	5.04	4.34	1.16	3.31	8.50	1.34
Soyabean	Kharif	78.18	68.76	82.74	88.51	109.68	99.05	99.64	127.36	122.14	146.66	118.61	103.74	118.32	85.92	136.15	142.23
Total Nine Oilseeds	Kharif	166.72	141.49	167.67	140.12	207.13	178.08	157.28	219.22	206.91	207.91	226.12	191.89	198.90	165.93	235.00	233.63
	Rabi	85.14	102.04	112.11	102.77	90.42	99.11	91.53	105.57	91.08	101.52	101.37	83.21		87.10	115.00	
	Total	251.86	243.54	279.78	242.89	297.55	277.19	248.82	324.79	297.99	309.43	327.49	275.11	198.90	253.04	350.00	233.63
Cotton #	Total	137.29	164.29	184.99	226.32	258.84	222.76	240.22	330.00	352.00	342.20	359.02	348.05	335.07	301.47	360.00	321.23
Jute # #	Total	102.52	93.99	99.70	103.17	102.20	96.34	112.30	100.09	107.36	103.40	110.83	106.18	102.84	99.38	110.00	99.05
Mesta # #	Total	9.21	8.73	8.70	9.56	9.90	7.31	5.87	6.11	6.63	5.90	6.07	5.08	5.16	5.28	7.00	5.01
Jute & Mesta # #	Total	111.73	102.72	108.40	112.73	112.11	103.65	118.17	106.20	113.99	109.30	116.90	111.26	108.00	104.66	117.00	104.05
Sugarcane	Total	2338.62	2370.88	2811.72	3555.20	3481.88	2850.29	2923.02	3423.82	3610.37	3412.00	3521.42	3623.33	3414.25	3521.63	3550.00	3052.46

\# Lakh bales of 170 kgs. each

\# # Lakh bales of 180 kgs. each

Annexure-3.3

MINIMUM SUPPORT PRICES

(According To Crop Year)

(As on 31.12.2016)

(Rs. per quintal)

Sl. No.	Commodity	Variety	2012-13	2013-14	(#) increase in MSP 2013-14 over 2012-13	2014-15	(#) increase in MSP 2014-15 over 2013-14	2015-16	(#) increase in MSP 2015-16 over 2014-15	2016-17	(#) increase in MSP 2016-17 over 2015-16
	**KHARIF CROPS**										
1	PADDY	Common	1250	1310	60(4.8)	1360	50(3.8)	1410	50(3.7)	1470	60(4.3)
		Grade 'A'	1280	1345	65(5.1)	1400	55(4.1)	1450	50(3.6)	1510	60(4.1)
2	JOWAR	Hybrid	1500	1500	-	1530	30(2.0)	1570	40(2.6)	1625	55(3.5)
		Maldandi	1520	1520	-	1550	30(2.0)	1590	40(2.6)	1650	60(3.8)
3	BAJRA		1175	1250	75(6.4)	1250	-	1275	25(2.0)	1330	55(4.3)
4	MAIZE		1175	1310	135(11.5)	1310	-	1325	15(1.1)	1365	40(3.0)
5	RAGI		1500	1500	-	1550	50(3.3)	1650	100(6.5)	1725	75(4.5)
6	ARHAR (Tur)		3850	4300	450(11.7)	4350	50(1.2)	4625^	275(6.3)	5050^^	425(9.2)
7	MOONG		4400	4500	100(2.3)	4600	100(2.2)	4850^	250(5.4)	5225^^	375(7.7)
8	URAD		4300	4300	-	4350	50(1.2)	4625^	275(6.3)	5000^^	375(8.1)
9	COTTON	Medium Staple	3600	3700	100(2.8)	3750	50(1.4)	3800	50(1.3)	3860	60(1.6)
		Long Staple	3900	4000	100(2.6)	4050	50(1.3)	4100	50(1.2)	4160	60(1.5)
10	GROUNDNUT IN SHELL		3700	4000	300(8.1)	4000	-	4030	30(0.8)	4220*	190(4.7)
11	SUNFLOWER SEED		3700	3700	-	3750	50(1.4)	3800	50(1.3)	3950*	150(3.9)
12	SOYABEEN	Black	2200	2500	300(13.6)	2500	-	-	-	-	
		Yellow$$	2240	2560	320(14.3)	2560	-	2600	40(1.6)	2775*	175(6.7)
13	SESAMUM		4200	4500	300(7.1)	4600	100(2.2)	4700	100(2.2)	5000^	300(6.4)
14	NIGERSEED		3500	3500	-	3600	100(2.9)	3650	50(1.4)	3825*	175(4.8)
	**RABI CROPS**										
15	WHEAT		1350	1400	50(3.7)	1450	50(3.6)	1525	75(5.2)	1625	100(6.6)
16	BARLEY		980	1100	120(12.2)	1150	50(4.5)	1225	75(6.5)	1325	100(8.2)
17	GRAM		3000	3100	100(3.3)	3175	75(2.4)	3500**	325(10.2)	4000^	500(14.3)
18	MASUR (LENTIL)		2900	2950	50(1.7)	3075	125(4.2)	3400**	325(10.6)	3950!	550(16.2)
19	RAPESEED/MUSTARD		3000	3050	50(1.7)	3100	50(1.6)	3350	250(8.0)	3700*	350(10.4)
20	SAFFLOWER		2800	3000	200(7.1)	3050	50(1.7)	3300	250(8.2)	3700*	400(12.1)
21	TORIA		2970	3020	50(1.7)	3020	-	3290	270(8.9)	3560***	270(8.2)
	**OTHER CROPS**										
22	COPRA	Milling	5100	5250	150(2.94)	5250	-	5550	300(5.7)	5950	400(7.2)
	(Calender Year)	Ball	5350	5500	150(2.80)	5500	-	5830	330(6.0)	6240	410(7.0)
23	DE-HUSKED COCONUT (Calender Year)		1400	1425	25(1.79)	1425	-	1500	75(5.3)	1600	100(6.7)
24	JUTE		2200	2300	100(4.55)	2400	100(4.3)	2700	300(12.5)	3200	500(18.5)
25	SUGARCANE$		170.00	210.00	40(23.5)	220.00	10(4.8)	230.00	10(4.5)	230.00	-

Figures in brackets indicate percentage increase.

$ Fair and remunerative price.

$$ Minimum Support Price of Soyabean yellow is also applicable to black variety during 2015-16 and 2016-17.

* Including Bonus of Rs. 100 per quintal.

** Including Bonus of Rs. 75 per quintal .

^ Including Bonus of Rs. 200 per quintal.

^^ Including Bonus of Rs. 425 per quintal.

! Including Bonus of Rs. 150 per quintal

*** To be marketed in 2017-18

Annexure-6.1

Estimates of Area, Production and Yield of Total Oilseeds

STATES	Area (' 000 Hectares)						Production ('000 Tonnes)						Yield (Kgs./Hect.)					
	2010-11	2011-12	2012-13	2013-14	2014-15	2015-16*	2010-11	2011-12	2012-13	2013-14	2014-15	2015-16*	2010-11	2011-12	2012-13	2013-14	2014-15	2015-16*
Andhra Pradesh	1825.8	1500.2	1439.5	1642.4	1072.0	914.0	1332.3	723.8	928.3	1414.3	597.2	873.0	730	482	645	861	557	955
Assam	266.7	268.3	306.2	304.2	306.9	306.0	153.6	149.5	186.8	186.3	205.7	185.0	576	557	610	613	670	605
Bihar	130.0	133.4	128.0	123.7	116.2	119.2	136.3	139.5	143.3	146.2	127.0	128.2	1048	1046	1120	1182	1093	1076
Chhattisgarh	316.5	308.3	297.5	289.6	291.1	298.9	217.2	169.5	215.0	185.4	174.2	149.6	686	550	723	640	599	501
Gujarat	2893.0	3131.0	2452.0	3079.0	2545.6	2558.6	4896.1	5035.0	2705.0	6870.4	4886.9	4101.8	1692	1608	1103	2231	1920	1603
Haryana	519.5	553.0	580.2	549.1	510.6	528.2	963.8	771.0	993.1	899.0	743.4	849.2	1855	1394	1712	1637	1456	1608
Himachal Pradesh	15.0	14.9	13.5	12.5	12.2	11.7	7.7	8.6	6.9	6.1	6.6	8.6	514	579	514	490	542	738
Jammu & Kashmir	64.6	64.5	64.8	65.7	59.2	59.7	53.0	53.3	51.1	58.8	40.4	40.5	821	826	789	895	682	677
Jharkhand	182.0	228.9	250.6	275.8	267.5	259.6	113.7	155.5	197.2	182.9	177.6	176.9	625	680	787	663	664	681
Karnataka	1624.0	1416.0	1422.0	1410.0	1373.0	1331.0	1270.0	942.0	919.6	1162.0	959.0	867.0	782	665	647	824	698	651
Kerala	2.0	1.9	1.0	1.0	0.7	0.5	2.1	2.3	1.1	1.0	0.8	0.6	1050	1226	1045	980	1054	1175
Madhya Pradesh	7029.9	7201.6	7534.4	7732.0	7066.1	7336.0	8035.4	7727.8	9276.0	6634.9	7724.2	6243.5	1143	1073	1231	858	1093	851
Maharashtra	3615.0	3667.0	3806.0	4148.0	4242.0	4193.0	5040.0	4485.0	5086.8	5293.9	2850.2	2375.0	1394	1223	1337	1276	672	566
Odisha	290.4	250.7	243.3	223.4	212.0	178.3	179.8	165.8	170.3	168.8	141.5	114.5	619	661	700	755	667	642
Punjab	53.5	50.0	51.4	48.1	45.6	46.5	71.5	68.0	69.4	64.2	57.7	61.3	1336	1360	1350	1335	1265	1318
Rajasthan	5488.4	4622.7	4912.2	5274.0	4457.2	4834.8	6604.8	5744.5	6364.6	6033.8	5314.3	5710.6	1203	1243	1296	1144	1192	1181
Tamil Nadu	449.4	449.2	388.5	408.2	415.0	412.2	933.1	1113.7	816.9	964.2	985.3	919.1	2076	2479	2103	2362	2374	2230
Telangana	493.2	444.9	505.5	387.9	496.0	449.0	661.6	540.9	722.8	471.4	630.0	496.0	1341	1216	1430	1215	1270	1105
Uttar Pradesh	1105.0	1129.0	1147.0	1106.0	1127.0	1292.0	919.4	935.0	1030.5	895.8	787.2	863.5	832	828	898	810	698	668
Uttarakhand	27.2	30.0	32.2	31.8	31.6	33.0	27.5	32.5	39.7	34.1	29.6	36.0	1012	1082	1235	1070	938	1091
West Bengal	671.6	676.1	732.1	770.3	776.4	793.0	703.6	672.4	850.7	909.9	901.4	937.4	1048	994	1162	1181	1161	1181
**All India**	**27224.3**	**26308.1**	**26484.4**	**28050.2**	**25596.2**	**26134.5**	**32477.3**	**29798.6**	**30939.8**	**32749.4**	**27510.8**	**25303.7**	**1193**	**1133**	**1168**	**1168**	**1075**	**968**

*4th Advance estimate.

Annexure 7.1

Statement of amount of funds released to the States under PKVY during 2015-16. (60:40 and 90:10 ratio of Central : State Share).

(Rs.in Lakh)

Sl. No.	State	No. of Clusters	Total outlay approved by EC (Rs. in lakhs)	60% or 90% of GOI share	GOI Share released.
1	Assam	220	1552.42	1397.19	576.39
2	Arunachal Pradesh	19	137.08	123.37	51.41
3	Andhra Pradesh	411	2933.75	1760.25	1100.15
4	Bihar	327	2336.15	1401.69	1050.37
5	Chhattisgarh	188	1341.96	805.18	603.88
6.	Goa	04	28.55	17.13	7.14
7.	Gujarat	100	713.81	428.28	178.45
8.	Haryana	20	142.76	85.65	53.53
9	Himachal Pradesh	110	768.52	691.67	395.00
10	Jammu & Kashmir	28	199.87	179.88	74.95
11	Jharkhand	100	713.80	428.28	321.21
12	Karnataka	545	3890.26	2334.15	1945.12
13	Kerala	119	849.38	509.63	382.22
14	Madhya Pradesh	880	6281.50	3768.90	2826.67
15	Maharashtra	932	6929.36	4157.62	2598.51
16	Mizoram	34	237.54	213.79	89.08
17	Manipur	30	214.14	192.73	107.07
18	Nagaland	24	171.31	154.18	153.33
19	Meghalaya	45	321.21	289.09	144.54
20	Odisha	320	2284.19	1370.51	1027.88
21	Punjab	50	356.90	214.14	160.60
22	Rajasthan	755	5274.86	3164.92	2373.69
23	Sikkim	150	818.21	736.39	409.30
24	Tamil Nadu	112	799.46	479.68	399.73
25	Tripura	50	356.90	321.21	133.84
26	Telangana	300	2141.42	1284.85	1070.71
27	Uttar Pradesh	575	4104.39	2462.63	2052.20
28	Uttarakhand	550	3925.94	3533.35	1962.97
29	West Bengal	120	856.57	513.94	214.14
30	Andman & Nicobar	68	485.38	485.38	130.00
	Other administrative charges	-	-	-	24.85
	**Total**	**7186**	**51167.59**	**33505.66**	**22618.96**

Annexure- 8.1

Seeds Programmes in North-Eastern States

S. No.	Schemes/ Programmes Activities Approved	Details of Schemes/ Programmes/ Activities taken	Target for 2015-16	Achievement 2015-16	Target for 2016-17	Achievement 201-17
1.	National Seed Reserve (NSR)	The basic objective of this component is to keep seed available for meeting requirement during natural calamities.	57798 Qtl	57798 Qtl	71387 Qtl	71387 Qtl. likely
2.	Transport Subsidy on movement of Seeds to North-Eastern States including Sikkim, Himachal Pradesh, J&K, Uttaranchal and Hill areas of West Bengal.	The topographical situation and climatic condition being not conducive for the production of seeds in the North-Eastern States.<br>(a) Reimbursement of 100% difference between road and rail transportation cost to implementing States/agency for movement of seeds produced outside the state and movement to identified state capital/district headquarter.<br><br>(b) The actual cost, restricted to maximum limit of Rs. 60/- per quintal for the movement of seed within the State from State Capital/ district headquarters to sale outlets/sale counters is being reimbursed to implementing State/agency.	It is a reimbursement programme. Therefore, targets cannot be pre-fixed as transportation of seeds depends on demand which varies from year to year.	For NE State, for a quantity 87,721 qtls quintals transported exclusively to NE States in 2015-16 an amount of Rs. 3.398 crore has been reimbursed.<br><br>The total quantity including NE States was 192,105 quintals in 2015-16.	As mentioned in Col. 4	For NE States a quantity of 9,719 qtl transported exclusively to NE States. An amount of Rs. 0.522 crore has been reimbursed.<br><br>(The Total quantity of seeds transported total including NE States is 1,43,892 Quintal (upto 03.11.2016)

Annexure- 9.1

State-wise Weather Based Crop Insurance Scheme (WBCIS)-Cumulative (upto Rabi 2015-16)

₹(in lakh)

Sl. No.	State / UT	Farmers Insured (no.)	Area Insured (ha.)	Sum Insured	Farmer Premium	GOI Share in Premium	State Govt Premium (share)	Gross Premium	Claims Payable	Claims Paid	Farmers Benefitted (no.)
(1)	(2)	(3)	(4)	(5)	(6)	(7)	(8)	(9)	(10)	(11)	(12)
1	Andhra Pradesh	3616822	5902076.85	1513346.07	61931.86	44589.08	44589.08	151110.02	106626.79	106616.77	2371998
2	Assam	67575	39212.19	23589.98	1193.96	596.53	596.53	2387.03	1892.79	290.17	46864
3	Bihar	12885954	13114092.98	3043374.59	86904.17	89458.40	89458.40	265820.96	200006.84	149507.27	10713612
4	Chhattisgarh	1287074	2246719.39	443314.00	18905.01	11149.55	12093.90	42148.47	31947.25	31947.25	843828
5	Gujarat	497661	413126.40	22394.75	251.80	839.80	1147.88	2239.48	857.35	857.35	170576
6	Haryana	359388	602022.74	190262.00	3695.45	5600.39	7472.66	16768.49	6565.42	6208.42	250497
7	Himachal Pradesh	390609	4618667.49	141956.95	7784.28	3751.34	3751.34	15287.32	11500.67	9096.27	246360
8	Jharkhand	585356	561878.72	120853.09	4163.24	3248.90	3248.90	10661.02	7750.77	6745.01	526610
9	Jammu & Kashmir	1364	809.23	461.26	23.06	11.53	11.53	46.13	54.18	23.93	1364
10	Karnataka	985183	1219977.56	234493.18	11518.49	7533.72	7533.72	26585.93	18723.72	18639.77	793077
11	Kerala	162894	119924.51	36981.50	1255.63	1013.54	1347.45	3616.63	3324.96	2702.84	128796
12	Madhya Pradesh	1381347	1919796.56	509160.53	18028.63	16041.97	16041.97	50112.56	31616.70	30498.31	1151892
13	Maharashtra	2908194	2932546.26	783273.94	37825.70	26241.18	31185.04	95251.93	111327.56	110007.98	2546864
14	Odisha	315755	456542.27	65780.84	1414.07	2121.10	2121.10	5656.26	3210.36	3210.36	215814
15	Punjab	67	338.20	47.58	1.20	1.80	1.80	4.79	0.67	0.67	50
16	Rajasthan	44125707	58032258.65	4816829.91	168450.20	154340.74	154340.74	477033.57	419552.15	392447.00	28941314
17	Tamil Nadu	133157	179235.54	84705.82	1152.19	1051.40	1051.40	3254.56	2501.27	2333.74	73237
18	Telangana	165399	173376.73	52828.73	2617.15	1308.57	1308.57	5234.30	11640.66	11215.16	143077
19	Uttar Pradesh	1529699	1318113.64	265304.16	12053.71	16714.42	16714.42	45482.57	54403.62	53716.05	1394020
20	Uttarakhand	177887	323710.21	63102.43	3245.01	1478.17	1478.17	6201.36	8755.61	8743.33	114323
21	West Bengal	123314	118031.51	21084.80	642.73	684.21	684.21	2011.16	1457.17	1364.68	66812
	**TOTAL**	**71700406**	**94292457.61**	**12433146.10**	**443057.54**	**387776.35**	**396178.82**	**1226914.54**	**1033716.50**	**946172.31**	**50740985**

Note:Rabi 2015-16 statistics is provisional and claims from few states are yet to be reported

Source: Department of Agriculture, Cooperation & Farmers Welfare(Credit Division)

Annexure 9.2

State-wise National Agricultural Insurance Scheme (NAIS)-Cumulative upto Rabi 2015-16

(₹ in lakh)

Sl. No.	State / UTs	No. of Farmers covered	Area Insured (In Ha)	Sum Insured	Farmers' Premium	State Govt. Premium (Share)	GoI Premium (Share)	Gross Premium	Claims Reported	Claims Paid	No. of Farmers Benefitted
(1)	(2)	(3)	(4)	(5)	(6)	(7)	(8)	(9)	(10)	(11)	(12)
1	Andhra Pradesh	30697873	46529046.05	6581021.59	172286.79	8306.94	8306.94	188900.67	503002.75	487610.41	6974654
2	Assam	422654	309427.45	90821.88	2269.20	130.77	130.77	2530.74	1693.36	1662.41	66123
3	Bihar	10707824	11710269.13	2829879.34	57771.32	2566.75	2566.75	62904.82	418597.03	258432.25	4439428
4	Chhattisgarh	11802512	23539524.40	1504999.34	35314.54	1955.61	906.27	38176.43	108828.20	107729.40	2378860
5	Goa	8211	13440.13	318.12	4.31	0.73	0.60	5.65	2.36	2.36	702
6	Gujarat	15496181	35194678.68	4931852.71	179895.16	15697.38	7080.19	202672.73	880375.31	880375.32	5500924
7	Haryana	635778	769038.32	83496.10	2345.73	34.13	34.13	2413.98	4336.39	4336.40	129424
8	Himachal Pradesh	372427	292221.87	78532.67	900.42	668.81	87.87	1657.10	2252.03	1990.57	114714
9	Jharkhand	6931179	4247333.09	459685.46	10461.03	353.70	353.70	11168.42	75038.95	60945.16	2582643
10	Karnataka	14348056	22575560.72	2011342.57	54238.73	1861.45	1812.38	57912.56	282810.44	282227.32	6093637
11	Kerala	461282	414760.68	87165.63	1629.98	125.80	125.80	1881.59	3062.82	3062.82	85472
12	Madhya Pradesh	43751245	103084080.56	9984461.14	261521.58	3633.49	3633.49	268788.57	1018918.44	994326.91	9857683
13	Maharashtra	51770126	40869932.70	3971360.41	127387.50	36666.43	5342.31	169396.24	983411.17	894024.20	24809085
14	Manipur	35645	57471.71	14812.25	348.42	10.16	10.16	368.73	1226.20	1226.20	29932
15	Meghalaya	36357	36342.74	7473.42	277.09	21.32	21.32	319.72	68.46	52.18	3600
16	Mizoram	121	133.79	23.24	0.53	0.03	0.03	0.58	11.23	11.23	119
17	Odisha	19975543	19460413.26	3623794.66	81722.19	4494.70	4494.70	90711.59	387711.37	387711.37	4408892
18	Rajasthan	15058674	31379980.35	1620309.00	45016.74	368.81	368.81	45754.35	262165.99	262165.99	5200566
19	Sikkim	2062	1445.41	351.95	6.78	0.47	0.47	7.72	1.28	1.28	86
20	Tamil Nadu	8308811	10560119.94	2585150.28	33934.12	30230.98	3150.68	67315.77	300114.93	299341.23	2888885
21	Telangana	1288046	1534585.11	667068.24	17540.79	785.44	785.44	19111.68	4914.60	2204.19	76278
22	Tripura	21248	14086.19	3301.68	80.68	4.86	4.86	90.39	58.40	58.31	3449
23	Uttar Pradesh	23426012	31070385.59	3353782.49	63262.82	2609.59	2609.59	68482.01	116937.52	116937.52	4517617
24	Uttarakhand	399156	372508.94	87097.93	1799.49	89.32	89.32	1978.13	4188.31	4188.31	119370
25	West Bengal	15115995	7428041.85	2111161.32	50270.65	53428.45	5784.69	109483.78	149054.42	133834.36	3270326
26	A & N Islands	4773	7147.86	2300.39	3.25	45.00	15.48	63.73	116.41	115.22	1102
27	Puducherry	43258	59992.72	11669.90	137.23	72.97	17.89	228.10	316.95	313.75	7269
28	Jammu & Kashmir	49065	68994.99	10902.23	197.86	7.97	7.97	213.80	126.42	124.07	4492
	**GRAND TOTAL**	**271170114**	**391600964.24**	**46714135.95**	**1200624.93**	**164172.06**	**47742.61**	**1412539.59**	**5509341.76**	**5185010.75**	**83565332**

Note:Rabi 2015-16 statistics is provisional and claims from few states are yet to be reported

Source: Department of Agriculture, Cooperation & Farmers Welfare(Credit Division)

Annexure 9.3

State-wise Modified National Agricultural Insurance Scheme (MNAIS)-Cumulative (upto Rabi 2015-16)

₹(in lakh)

Sl. No.	States / Uts.	Farmers Insured (no.)	Area Insured (ha.)	Sum Insured	Farmer Premium	GOI Share in Premium	State Govt."s Premium	Premium Collected	Claims Payable	Claims Paid	Farmers Benefitted (no.)
(1)	(2)	(3)	(4)	(5)	(6)	(7)	(8)	(9)	(10)	(11)	(12)
1	Andhra Pradesh	1800231	1881035.28	813718.17	34200.22	21500.27	21500.27	77200.77	85812.33	84733.87	638698
2	Andaman & Nico-bar	783	1230.47	460.32	0.00	3.04	13.24	16.28	5.86	5.86	184
3	Assam	19929	15685.01	6504.53	154.08	47.33	47.33	248.81	101.91	69.88	1837
4	Bihar	1382947	1570614.95	262831.54	15447.12	18889.34	18889.34	53225.80	21320.46	17559.28	269128
5	Chhattisgarh	18	31.76	4.76	0.13	0.04	0.04	0.22	0.00	0.00	0
6	Goa	296	290.64	77.75	1.56	0.19	0.19	1.94	14.49	0.00	82
7	Gujarat	17084	590.66	261.22	10.99	8.62	8.62	28.23	0.00	0.00	0
8	Haryana	262687	446689.58	184769.36	3816.44	1188.17	1188.17	6192.78	5042.13	5021.04	46374
9	Jharkhand	117508	118015.56	26667.49	1175.96	717.79	717.79	2611.55	379.00	376.40	8738
10	Karnataka	1566251	2297977.72	371696.96	16549.34	11428.14	11430.20	39405.50	24553.12	24451.66	457984
11	Kerala	47416	64041.47	23924.50	512.37	284.42	410.51	1207.30	566.42	472.60	4287
12	Madhya Pradesh	218380	359047.89	51088.50	1229.69	380.15	380.15	1990.97	873.30	873.30	12717
13	Maharashtra	51964	49621.11	7621.06	382.17	456.50	523.32	1361.99	0.00	0.00	0
14	Mizoram	512	488.00	99.33	3.44	1.52	1.52	6.48	9.44	9.44	512
15	Odisha	425024	280279.69	103216.84	2185.16	646.75	646.75	3478.66	21363.20	21362.95	198161
16	Rajasthan	12620454	13719000.70	1063428.05	47976.12	34328.86	34328.87	116633.85	154286.53	132231.21	4666254
17	Tamil Nadu	476483	522839.98	134936.23	5708.90	4166.24	4166.24	14041.38	26782.85	26677.68	243958
18	Telangana	918835	1255672.01	534647.66	12079.71	3003.94	3003.94	18087.58	6285.71	47.84	67933
19	Uttar Pradesh	5585861	6076530.80	2012245.84	57391.04	27935.16	27935.15	113261.45	177907.42	177046.74	2425868
20	Uttarakhand	217704	183679.26	66147.04	1139.51	155.86	155.86	1450.22	1515.67	1513.72	36723
21	West Bengal	1943351	961459.29	501798.15	15353.13	12886.85	18334.52	46574.49	28935.36	26664.38	428482
	**Total**	**27673718**	**29804821.84**	**6166145.31**	**215317.06**	**138029.18**	**143682.01**	**497026.26**	**555755.18**	**519117.85**	**9507920**

Note:Rabi 2015-16 statistics is provisional and claims from few states are yet to be reported

Source: Department of Agriculture, Cooperation & Farmers Welfare(Credit Division)

Annexure 13.1

Sector wise Approved Projects Cost 2012-13

₹(in lakh)

SNo	STATE NAME	CROP	HORT	SERI	ANHB	OTHR	FISH	COOP	IPMT	SEED	FINM	AMEC	EXTN	MRKT	NONF	ITEC	AGRE	NRM	IRRI	ORFM	DDEV	TOTAL
1	ANDHRA PRADESH	11.29	210.45	10.00	105.80		21.53			63.68		319.81	4.95	48.67			19.49	19.65		15.00	29.90	880.22
2	ARUNACHAL PRADESH	30.41	17.87	0.35	6.06	0.47	6.05					10.63	1.11	6.50					0.47	2.99	2.92	85.83
3	ASSAM	52.45	27.80		36.70	7.54	20.50			5.38		26.10	132.35	22.63	15.05		13.00		21.60	11.57	10.97	403.64
4	BIHAR	323.97	156.42		27.60	64.87	24.92	50.72				161.45	2.00								26.00	837.95
5	CHATTISHGARH	174.12	102.90		67.43	8.54	28.51	34.34	13.01	13.97	2.10	18.19	48.63	16.83			12.58		16.30		24.17	581.62
6	GOA	4.29	25.16			1.10			1.29	6.52			0.76				1.77				4.01	44.90
7	GUJARAT	156.82	155.93		75.75		25.04	3.28	1.04	24.82		100.83	94.24	27.71	4.05		6.97	149.88	165.89	11.63	1.80	1005.68
8	HARYANA	10.14	16.34		53.79	2.74	2.00		11.81	11.05	14.23	12.00	0.79	3.11			15.65	5.22	79.99	3.38	26.45	268.69
9	HIMACHAL PRADESH	14.25	3.72	1.00	8.59		10.28			1.56			0.10	3.00			0.76		3.35	10.50	8.06	65.17
10	JAMMU AND KASHMIR	1.81	11.44	1.27		0.63		0.43		4.24		0.96			1.27		0.19		10.71	2.37	12.00	47.32
11	JHARKHAND	252.84	12.00		12.17	89.51	15.68		16.56	55.34		140.13	5.42						49.31	4.00	95.85	748.81
12	KARNATAKA	18.67	114.03	13.50	101.21	81.59	15.00		3.50	24.85	5.00	85.00		43.00	5.00	16.00	24.40	25.00		21.00		596.75
13	KERALA	61.96	55.16		44.33	2.71	32.42					0.81	0.25	0.25	1.74		5.03	5.89	9.73	1.44	10.58	232.30
14	MADHYA PRADESH	50.01	100.60		108.16	18.39	12.34	134.00		113.83	25.18	65.74	25.89		1.51		14.35		85.00	5.69		760.69
15	MAHARASHTRA	137.86	173.06		30.16	10.61			252.61	15.00			82.72			1.16		370.00	25.06			1098.24
16	MANIPUR		8.34	1.15	5.57		7.81	0.70		0.60		2.30	12.53	1.60				1.00		0.75		42.35
17	MEGHALAYA		7.94		7.22													4.93	12.14			32.23
18	MIZORAM	29.47	15.77	12.55	14.40		13.91		0.30			3.11	5.77	0.70				55.37	3.47		1.30	156.12
19	NAGALAND	10.50	9.50	2.00	12.40	3.00	12.84	1.50	2.00		2.00	3.30	1.50	2.50	5.21			11.00	6.35	3.00		88.60
20	ORISSA	275.89	61.60		103.49		13.60	4.00	5.14	2.21	6.10	58.73	75.34				10.36		10.00		6.50	632.96
21	PUNJAB	10.00	1228.11		47.33	1.00	7.23		26.57		11.56	18.58			5.50		15.60	60.00			39.46	1470.94
22	RAJASTHAN	4.41	3.88		105.60		16.95	20.60	2.00	15.95		50.75		101.22			37.19		2.00	0.70	165.00	526.25
23	SIKKIM	1.52	8.36					0.91				1.28						6.50		1.20	1.00	20.77
24	TAMILNADU	160.24	87.25		64.16	9.19	40.98	12.25		28.29	1.90	112.20	24.75	32.44	8.74	3.34	3.72		9.20	6.60	27.63	632.88
25	TRIPURA	43.48	4.90		6.13		8.71					0.63		2.02								65.87
26	UTTAR PRADESH	96.74	37.99		26.77	10.34	4.07	176.00	7.65	164.32	2.30	106.62	2.17	2.32		0.86	1.06	52.85	13.11	28.18	34.77	768.12
27	UTTARAKHAND	6.00		82.04	8.91		3.58			0.06	0.55		0.90	10.00			2.04			9.44	15.08	138.60
28	WEST BENGAL		22.12	6.35	32.01	2.45	9.28	6.68		19.96				167.57	4.31		0.15	4.63		6.11		281.62
29	TOTAL	1939.14	2678.64	130.21	1111.74	314.68	353.23	445.41	343.48	571.63	70.92	1299.15	522.17	492.07	52.38	21.36	184.31	771.92	523.68	145.55	543.45	12515.12

Total Cost : Rs. 12515.12

CROP CROP DEVELOPMENT; HORT HORTICULTURE; SERI SERICULTURE; ANHB ANIMAL HUSBANDRY; OTHR INNOVATIVE PROGRAMMES / OTHERS; FISH FISHERIES; COOP COOPERATIVES/COOPERATION;IPMT INTEGRATED PEST MANAGEMENT; SEED SEED; FINM FERTILISERS AND INM; AMEC AGRICULTURE MECHANISATION; EXTN EXTENSION; MRKT MARKETING AND POST HARVEST MANAGEMENT; NONF NON FARM ACTIVITIES; ITEC INFORMATION TECHNOLOGY; AGRE RESEARCH (AGRI/HORTI/ANIMAL HUSBANDRY etc); NRM NATURAL RESOURCE MANAGEMENT; IRRI MICRO/MINOR IRRIGATION; ORFM ORGANIC FARMING / BIO FERTILISER; DDEV DAIRY DEVELOPMENT

Sector wise Approved Projects Cost 2013-14

Unit In Crores

SNo	STATE NAME	CROP	HORT	SERI	ANHB	OTHR	FISH	COOP	IPMT	SEED	FINM	AMEC	EXTN	MRKT	NONF	ITEC	AGRE	NRM	IRRI	ORFM	DDEV	TOTAL
1	ANDHRA PRADESH	3.50	86.66	4.42	50.47	34.43	6.56	28.00		48.34		94.50		16.53			14.96	29.91	1.02	16.00	13.22	448.52
2	ARUNACHAL PRADESH	9.99	5.42	0.99	10.29	0.59	7.32			1.00		1.00	1.00						0.75			38.35
3	ASSAM	108.79	39.72	4.95	29.70	24.50	14.85			28.27		20.41	100.22	14.00					6.50	11.88	4.46	408.25
4	BIHAR	328.35	12.00			23.43	15.40	49.46				44.56		28.55								501.75
5	CHATTISHGARH	193.82	69.66		8.21		28.32	14.20		10.85		12.75					0.25		2.00	1.26	4.96	346.28
6	GOA	6.97			7.71		2.50						1.56	21.78		1.83						42.35
7	GUJARAT		0.26		27.70	1.20		0.18			3.00	103.64		31.66	13.13	1.25		18.80	90.45	39.09	10.50	340.86
8	HARYANA	20.40	20.57		63.70	3.72		2.80	2.00	24.08	5.83	13.93	0.42	109.01			11.00	13.17	89.07	2.00	23.15	404.85
9	HIMACHAL PRADESH	4.16	107.86	1.00	7.56		7.27						4.16	4.00			6.70	6.70	5.76	7.50		162.67
10	JAMMU AND KASHMIR	2.45	104.15	0.65		0.00		0.20		2.34	0.60	1.45		1.22	3.11				5.41	1.64		123.22
11	JHARKHAND	11461.49								10.31			60.81							3.60		11536.21
12	KARNATAKA	58.64	123.58	14.00	51.59	62.20	10.75		1.50	116.10		72.00		40.00	31.00		35.25	92.50		14.40		723.51
13	KERALA	36.83	63.12		72.32		12.78					51.45		0.52			6.98	17.48			12.25	273.73
14	MADHYA PRADESH	109.38	1052.96		99.67	18.50	10.71		1.77	77.91		63.08	26.29		8.57		1.57		4.50	3.00	29.47	1507.38
15	MAHARASHTRA	98.10	135.46	8.67	89.80	2.75			1.83	16.38	2.50	30.00	56.85	15.64				52.58	214.00	4.88	55.76	785.20
16	MANIPUR		3.00	1.00	4.05		5.40	2.60		3.07		1.41	3.34					7.25		0.29	1.60	33.01
17	MEGHALAYA		8.64	2.78	6.55	3.44	11.50	0.94											3.10			36.95
18	MIZORAM	41.59	10.58	3.00	12.06		10.31		0.33			22.35	2.41	2.48				11.37	6.50	0.05	1.33	124.36
19	NAGALAND	5.20	6.07	1.50	6.17	1.18	6.40	1.50	0.80	1.00	0.80	3.20		1.60	0.59			13.30	2.00	0.60		51.91
20	ORISSA	258.92	93.26		40.36	55.88	59.96	92.65	2.88	3.79	0.31	79.08	52.80	19.40				29.81	56.13		67.23	912.46
21	PUNJAB	59.90	348.94	0.72	80.63		1.08										31.42	51.00			29.80	603.49
22	RAJASTHAN				64.29		1.25		3.00	25.06	4.42	4.50	0.40	19.95			4.00	32.52				159.39
23	TAMILNADU	85.51	41.82		19.28		6.96	1.40			6.00	162.10	44.00	19.90		13.02	3.60				25.54	429.13
24	TRIPURA	24.87	9.06		9.67		12.20					2.25						4.54	0.63		2.52	65.74
25	UTTAR PRADESH	348.89	30.44	1.30	47.48	26.11	12.12	25.00	2.03	90.41	14.59	47.40	1.00	17.00		0.64	3.61	40.67	151.01	95.03	34.98	989.71
26	UTTARAKHAND	12.96	7.56		11.66	4.53				7.47	0.35	3.20	1.10	19.10				76.84		19.42	16.80	180.99
27	WEST BENGAL	5.67	31.35	10.72	48.85	14.92	17.67	16.50	0.21	13.90	4.33	60.00		80.11				20.00	5.00		5.74	334.97
28	TOTAL	13286.38	2412.14	55.70	869.77	277.38	261.31	235.43	16.35	480.28	42.73	894.26	356.36	462.45	56.40	16.74	119.34	518.44	643.83	220.64	339.31	21565.24

Total Cost : Rs. 21565.24

CROP CROP DEVELOPMENT; HORT HORTICULTURE; SERI SERICULTURE; ANHB ANIMAL HUSBANDRY; OTHR INNOVATIVE PROGRAMMES / OTHERS; FISH FISHERIES; COOP

COOPERATIVES/COOPERATION;IPMT INTEGRATED PEST MANAGEMENT; SEED SEED; FINM FERTILISERS AND INM; AMEC AGRICULTURE MECHANISATION; EXTN EXTENSION; MRKT MARKETING AND POST HARVEST MANAGEMENT; NONF NON FARM ACTIVITIES; ITEC INFORMATION TECHNOLOGY; AGRE RESEARCH (AGRI/HORTI/ANIMAL HUSBANDRY etc); NRM NATURAL

RESOURCE MANAGEMENT; IRRI MICRO/MINOR IRRIGATION; ORFM ORGANIC FARMING / BIO FERTILISER; DDEV DAIRY DEVELOPMENT

Sector wise Approved Projects Cost 2014-15

Unit In Crores

Sl. No	STATE NAME	CROP	HORT	SERI	ANHB	OTHR	FISH	COOP	IPMT	SEED	FINM	AMEC	EXTN	MRKT	NONF	ITEC	AGRE	NRM	IRRI	ORFM	DDEV	TOTAL
1	ANDHRA PRADESH		44.15	6.44	80.12	2.22	14.70		7.63	29.50	17.45	28.09	2.09	16.96			12.63	3.62		10.97	20.84	297.41
2	ARUNACHAL PRADESH	9695.75	9.14	2.45	12.50	6.28	8.89	2.02				4.40		3.00								9744.43
3	ASSAM	128.36	64.68	3.28	6.05	9.46	5.07			20.65		62.50	4.15	17.00	5.35		3.20		65.60	11.00	7.74	414.09
4	BIHAR	610.19		1.44	23.37	9.95		77.36		2.88	3.07	31.75		15.08					30.20			805.29
5	CHATTISHGARH	179.82	46.00		15.16		28.02	6.07		32.58	18.70						15.36		17.00		8.35	367.06
6	GOA						9.77				0.41		0.45			10.88		15.90				37.41
7	GUJARAT	8.35	172.19		34.40		10.24		2.14	58.20		281.04					0.70	450.00	15.00	0.20	15.89	1048.35
8	HARYANA	125.27	62.34		47.54		6.77	0.34	2.25	29.98		22.75		16.30			21.48	13.51	99.22		14.86	462.61
9	HIMACHAL PRADESH	3.82	18.50	1.00	23.03		1.89						5.44	1.40			1.19	6.70	12.00	8.00	2.40	85.37
10	JAMMU AND KASHMIR	1.74	10.30	0.80	14.72	100.94	0.60	0.40		2.66		3.58	1.01	0.75	2.56		0.71	1.75	4.98	3.02		150.52
11	JHARKHAND	209.59						14.31	7.69				1.85			0.77				7.85		242.06
12	KARNATAKA	11.46	128.13	18.05	131.35	42.50	10.89		5.10	49.00	1.50	72.68	38.94	178.74	25.00		39.15	41.53	51.68	12.45	2.00	860.15
13	KERALA	65.22	74.55		57.72		49.01			0.78		4.00		14.60	46.44		18.08	21.98	4.33	1.40	8.74	366.85
14	MADHYA PRADESH	23.78	23.30	5.04	38.47	49.89	7.32		5.50	105.38		100.08	46.60				3.74		93.29			502.39
15	MAHARASHTRA	105.00	239.14	6.02	88.65	1.44	4.43			13.69	13.95		57.19	34.49			3.77	91.10	600.00	3.10	37.81	1299.78
16	MANIPUR		4.31	3.12	5.83		5.33	1.30		3.42		0.94	2.18		1.71		0.25	5.09		3.67	1.00	38.15
17	MEGHALAYA		4.88	9.90	5.83		21.34		0.69			0.30	0.42						17.28			60.64
18	MIZORAM	9.68	11.65	10.42	17.89	2.75	13.98		2.55	0.11		9.13	6.03	0.66			1.33	17.79	6.80	0.21	2.02	113.00
19	NAGALAND	10.00	10.66	4.00	9.27	4.34	9.50	4.00	2.50	5.50	3.49	11.00	2.60	3.00	1.96			20.84		1.00	1.43	105.09
20	ORISSA	223.21	87.80	25.41	1.06				2.06	11.43	0.85	24.95				3.75	12.85		24.45			417.82
21	PUNJAB	289.76	19.62		177.28	5.00	35.34		0.50			43.72			9.03		41.20		59.36	2.00	81.53	764.34
22	RAJASTHAN	2.32	73.85		159.20	7.52	4.75	38.50	0.15	183.51	16.80	0.87	126.13	13.12	1.99		5.15	47.39	184.39	4.77	3.03	873.44
23	SIKKIM		3.50					1.19		1.20												5.89
24	TAMILNADU	74.00	523.77		7.30	1.72	7.02	5.25		0.28	16.66	57.68	5.81	24.07			6.49	11.75	3.20		32.62	777.62
25	TELANGANA		36.06	1.12	28.79	2.85	3.74	5.66	0.20	36.92	1.00	53.03		2.47	1.03		15.83		1.00	5.00	13.11	207.81
26	TRIPURA	25.38	16.97	1.00	11.00	7.10	10.50					4.77	7.49		0.90			1.60	1.26	1.00		88.97
27	UTTAR PRADESH	263.99	0.11	10.57	4.33	7.16	0.27	100.00		7.07		55.95		14.58		3.16		0.12	37.37	0.42	28.17	533.27
28	UTTARAKHAND	4.79	5.43		14.60	26.22			0.25			29.22	3.75	16.63		76.15	7.88	111.53		42.34		338.79
29	WEST BENGAL	106.06	36.08	9.91	118.75	51.43	98.87	25.45	6.96	32.75		59.25	32.75	148.49	2.49		3.11	26.43	88.13		30.24	877.15
30	TOTAL	12177.54	1727.11	119.97	1134.21	338.77	368.24	281.85	46.17	627.49	93.88	961.68	344.88	521.34	98.46	94.71	214.10	888.63	1416.54	118.40	311.78	21885.75

Total Cost : Rs. 21885.75

CROP CROP DEVELOPMENT; HORT HORTICULTURE; SERI SERICULTURE; ANHB ANIMAL HUSBANDRY; OTHR INNOVATIVE PROGRAMMES / OTHERS; FISH FISHERIES; COOP

COOPERATIVES/COOPERATION;IPMT INTEGRATED PEST MANAGEMENT; SEED SEED; FINM FERTILISERS AND INM; AMEC AGRICULTURE MECHANISATION; EXTN EXTENSION; MRKT MARKETING AND POST HARVEST MANAGEMENT; NONF NON FARM ACTIVITIES; ITEC INFORMATION TECHNOLOGY; AGRE RESEARCH (AGRI/HORTI/ANIMAL HUSBANDRY etc); NRM NATURAL
RESOURCE MANAGEMENT; IRRI MICRO/MINOR IRRIGATION; ORFM ORGANIC FARMING / BIO FERTILISER; DDEV DAIRY DEVELOPMENT

Sector wise Approved Projects Cost (Ongoing and Completed projects) 2015-16

(in Crore)

Sr No	State	Fin Year	Approved Project	CROP	HORT	SERI	ANHB	OTHR	FISH	COOP	IPMT	SEED	FINM	AMEC	EXTN	MRKT	NONF	ITEC	AGRE	NRM	IRRI	ORFM	DDEV	Total
1	ANDHRA PRADESH	2015-16	Completed				6.0200	0.2400															1.8500	8.1100
			Ongoing	14.9724	65.0484	13.0455	10.3600	14.3914	28.0900		5.1935	19.6100	11.6200	34.2300		13.1700	0.6326		16.7484	0.8384		42.2347	16.1000	306.2853
			Total	14.9724	65.0484	13.0455	16.3800	14.6314	28.0900		5.1935	19.6100	11.6200	34.2300		13.1700	0.6326		16.7484	0.8384		42.2347	17.9500	314.3953
2	ARUNACHAL PRADESH	2015-16	Ongoing		7.9200		1.4041																	9.3241
			Total		7.9200		1.4041																	9.3241
3	BIHAR	2015-16	Ongoing	2.6700																				2.6700
			Total	2.6700																				2.6700
4	CHATTISHGARH	2015-16	Completed		11.7700																			11.7700
			Ongoing	162.6470	12.8800							13.4600	3.3000	9.2000							9.6300	5.3600		216.4770
			Total	162.6470	24.6500							13.4600	3.3000	9.2000							9.6300	5.3600		228.2470
5	GOA	2015-16	Ongoing									3.4349												3.4349
			Total									3.4349												3.4349
6	GUJARAT	2015-16	Ongoing				10.5600		1.0900	5.9200	2.2000	18.5500			99.3834					95.6000			20.8000	254.1034
			Total				10.5600		1.0900	5.9200	2.2000	18.5500			99.3834					95.6000			20.8000	254.1034
7	HARYANA	2015-16	Ongoing	43.5650	31.9860		60.8096	84.8660	8.9500						36.8475		7.0800		68.4111	5.0000	84.0000		3.6000	435.1152
			Total	43.5650	31.9860		60.8096	84.8660	8.9500						36.8475		7.0800		68.4111	5.0000	84.0000		3.6000	435.1152
8	HIMACHAL PRADESH	2015-16	Completed											1.7500								7.6000		9.3500
			Ongoing	3.6250	9.4202		14.9670	1.0598	1.2600						1.3000						8.7513			40.3833
			Total	3.6250	9.4202		14.9670	1.0598	1.2600					1.7500	1.3000						8.7513	7.6000		49.7333
9	JHARKHAND	2015-16	Ongoing	40.6438	0.4310					38.2500	1.2667	20.3800		10.6200	5.1265		6.0000		0.5000					123.2180

CROP - CROP DEVELOPMENT; HORT - HORTICULTURE; SERI - SERICULTURE; ANHB - ANIMAL HUSBANDRY; OTHR - INNOVATIVE PROGRAMMES / OTHERS; FISH - FISHERIES; COOP - COOPERATIVES/COOPERATION;IPMT - INTEGRATED PEST MANAGEMENT; SEED - SEED; FINM - FERTILISERS AND INM; AMEC - AGRICULTURE MECHANISATION; EXTN - EXTENSION; MRKT - MARKETING AND POST HARVEST MANAGEMENT; NONF - NON FARM ACTIVITIES; ITEC - INFORMATION TECHNOLOGY; AGRE - RESEARCH (AGRI/HORTI/ANIMAL HUSBANDRY etc); NRM - NATURAL RESOURCE MANAGEMENT; IRRI -

MICRO/MINOR IRRIGATION; ORFM - ORGANIC FARMING / BIO FERTILISER; DDEV - DAIRY DEVELOPMENT

Sl. No.	State	Fin Year	Approved Project	CROP	HORT	SERI	ANHB	OTHR	FISH	COOP	IPMT	SEED	FINM	AMEC	EXTN	MRKT	NONF	ITEC	AGRE	NRM	IRRI	ORFM	DDEV	Total
			Total	40.6438	0.4310					38.2500	1.2667	20.3800		10.6200	5.1265		6.0000		0.5000					123.2180
10	KARNATAKA	2015-16	Ongoing	16.2000	51.5150	13.0500	94.8800	19.0000	5.2200			0.5600		146.0500	2.5500	128.4400			3.6700		0.9147	0.8900		482.9397
			Total	16.2000	51.5150	13.0500	94.8800	19.0000	5.2200			0.5600		146.0500	2.5500	128.4400			3.6700		0.9147	0.8900		482.9397
11	KERALA	2015-16	Ongoing	89.7550	15.5512		30.0823	2.0000	43.0000			0.9442				0.5500			5.7500	15.6919	15.5830		22.0031	240.9107
			Total	89.7550	15.5512		30.0823	2.0000	43.0000			0.9442				0.5500			5.7500	15.6919	15.5830		22.0031	240.9107
12	MADHYA PRADESH	2015-16	Ongoing	8.3859	64.8709	5.5012	55.1346	8.3609	7.6560			107.2606	30.0000	138.2424	75.2226	31.2835				15.5110	142.7737	12.9250	11.4021	714.5304
			Total	8.3859	64.8709	5.5012	55.1346	8.3609	7.6560			107.2606	30.0000	138.2424	75.2226	31.2835				15.5110	142.7737	12.9250	11.4021	714.5304
13	MAHARASHTRA	2015-16	Ongoing	55.2914	133.2446	8.7780	195.1100	0.5300				22.1800	7.6000		30.8842	15.4700			4.1109	127.5820	75.0000	1.1110	21.7889	698.6810
			Total	55.2914	133.2446	8.7780	195.1100	0.5300				22.1800	7.6000		30.8842	15.4700			4.1109	127.5820	75.0000	1.1110	21.7889	698.6810
14	MEGHALAYA	2015-16	Ongoing											2.1690	1.9551									4.1241
			Total											2.1690	1.9551									4.1241
15	MIZORAM	2015-16	Ongoing	0.4032	1.4960	2.0770	2.7230	0.7882	2.4240		0.6263	0.3114		0.3665	0.4150	0.9479			0.1350	6.1319	0.7515			19.5969
			Total	0.4032	1.4960	2.0770	2.7230	0.7882	2.4240		0.6263	0.3114		0.3665	0.4150	0.9479			0.1350	6.1319	0.7515			19.5969
16	NAGALAND	2015-16	Ongoing	5.5000	4.2499	2.0000	4.2500	19.0000	4.0000		1.1000	1.0000	1.0000	1.0000		2.5000	4.9000			1.0000		1.2500		52.7499
			Total	5.5000	4.2499	2.0000	4.2500	19.0000	4.0000		1.1000	1.0000	1.0000	1.0000		2.5000	4.9000			1.0000		1.2500		52.7499
17	ORISSA	2015-16	Completed															1.5700					2.0000	3.5700
			Ongoing	224.5779	64.8296		99.8311		55.1453	1.2250	2.0534	56.6238		1.8092	16.0089	0.4875		7.0795	40.0145		10.1316		24.8620	604.6793
			Total	224.5779	64.8296		99.8311		55.1453	1.2250	2.0534	56.6238		1.8092	16.0089	0.4875		8.6495	40.0145		10.1316		26.8620	608.2493
18	PUNJAB	2015-16	Ongoing														5.2700		19.2395	53.0000				77.5095
			Total														5.2700		19.2395	53.0000				77.5095
19	RAJASTHAN	2015-16	Ongoing				56.1820	66.6045				5.1000			9.4900		3.6500		18.2178			0.1205	87.4650	246.8298

CROP - CROP DEVELOPMENT; HORT - HORTICULTURE; SERI - SERICULTURE; ANHB - ANIMAL HUSBANDRY; OTHR - INNOVATIVE PROGRAMMES / OTHERS; FISH - FISHERIES; COOP - COOPERATIVES/COOPERATION;IPMT - INTEGRATED PEST MANAGEMENT; SEED - SEED; FINM - FERTILISERS AND INM; AMEC - AGRICULTURE MECHANISATION; EXTN - EXTENSION; MRKT - MARKETING AND POST HARVEST MANAGEMENT; NONF - NON FARM ACTIVITIES; ITEC - INFORMATION TECHNOLOGY; AGRE - RESEARCH (AGRI/HORTI/ANIMAL HUSBANDRY etc); NRM - NATURAL RESOURCE MANAGEMENT; IRRI -

MICRO/MINOR IRRIGATION; ORFM - ORGANIC FARMING / BIO FERTILISER; DDEV - DAIRY DEVELOPMENT

Sl. No.	State	Fin Year	Approved Project	CROP	HORT	SERI	ANHB	OTHR	FISH	COOP	IPMT	SEED	FINM	AMEC	EXTN	MRKT	NONF	ITEC	AGRE	NRM	IRRI	ORFM	DDEV	Total
			Total				56.1820	66.6045				5.1000			9.4900		3.6500		18.2178			0.1205	87.4650	246.8298
20	TAMILNADU	2015-16	Completed												9.0000									9.0000
			Ongoing	135.4875	18.8072		33.6996		15.2148			15.0000	7.8000	60.3602	63.5900	4.3050			28.6934	4.4500			25.1500	412.5577
			Total	135.4875	18.8072		33.6996		15.2148			15.0000	7.8000	60.3602	72.5900	4.3050			28.6934	4.4500			25.1500	421.5577
21	TELANGANA	2015-16	Ongoing		36.0000	2.9980	47.2550		3.6670		9.6000	20.0000	2.4850	49.0100	2.8000	9.0000	0.9900		12.0250			1.7000	9.7300	207.2600
			Total		36.0000	2.9980	47.2550		3.6670		9.6000	20.0000	2.4850	49.0100	2.8000	9.0000	0.9900		12.0250			1.7000	9.7300	207.2600
22	TRIPURA	2015-16	Ongoing	38.0225			3.7800	1.4065	3.7900									7.2010						54.2000
			Total	38.0225			3.7800	1.4065	3.7900									7.2010						54.2000
23	UTTAR PRADESH	2015-16	Ongoing	205.2940	27.5661	23.2961	81.7095	22.2932	7.6786			32.5326	27.8797	118.8233	1.0232	11.5827		3.1635	0.4700	2.8948	59.2707	0.7200		626.1980
			Total	205.2940	27.5661	23.2961	81.7095	22.2932	7.6786			32.5326	27.8797	118.8233	1.0232	11.5827		3.1635	0.4700	2.8948	59.2707	0.7200		626.1980
24	UTTARAKHAND	2015-16	Ongoing											2.3093										2.3093
			Total											2.3093										2.3093
25	WEST BENGAL	2015-16	Completed						27.4968															27.4968
			Ongoing		0.2400		2.3400		24.7567	23.2500		2.1900		15.0000	0.6000						14.1462			82.5229
			Total		0.2400		2.3400		52.2535	23.2500		2.1900		15.0000	0.6000						14.1462			110.0197
					Total Approved (Ongoing / Completed) Project Cost (in Crore) : =							5987.9072												

CROP - CROP DEVELOPMENT; HORT - HORTICULTURE; SERI - SERICULTURE; ANHB - ANIMAL HUSBANDRY; OTHR - INNOVATIVE PROGRAMMES / OTHERS; FISH - FISHERIES; COOP - COOPERATIVES/COOPERATION;IPMT - INTEGRATED PEST MANAGEMENT; SEED - SEED; FINM - FERTILISERS AND INM; AMEC - AGRICULTURE MECHANISATION; EXTN - EXTENSION; MRKT - MARKETING AND POST HARVEST MANAGEMENT; NONF - NON FARM ACTIVITIES; ITEC - INFORMATION TECHNOLOGY; AGRE - RESEARCH (AGRI/HORTI/ANIMAL HUSBANDRY etc); NRM - NATURAL RESOURCE MANAGEMENT; IRRI -

MICRO/MINOR IRRIGATION; ORFM - ORGANIC FARMING / BIO FERTILISER; DDEV - DAIRY DEVELOPMENT

Annexure – 13.2

State-wise list of flagship projects documented by NIRD

Sl. No.	Name of the State	Name of the Project
1.	Andhra Pradesh	1. Seed Management<br>2. Intensified mechanization<br>3. Integrated vegetable programme in A.P.<br>4. Induction of milch animals
2.	Arunachal Pradesh	1. Area expansion by land terracing for paddy.<br>2. Development of way side market sheds for fruits andvegetables<br>3. Development of commercial fish farms through privatefish farmers
3.	Assam	1. Shallow Tube Wells with electrically operated pump sets - increase in irrigated area and crop productionin areas where SWT have been installed.<br>2. Establishment of Organic agriculture produce market<br>3. Agri.mechanization through increased use of power tillers
4.	Bihar	1. Farm mechanization especially Power tillers<br>2. Seed production and distribution programme<br>3. Dairy activities undertaken under RKVY
5.	Chhattisgarh	1. Establishment of model villages 'Adarsh Gram'<br>2. Performance linked incentives to AI workers
6.	Goa	1. Establishing modern rice mill processing facilities<br>2. Incentivizing paddy cultivation through SHGs
7.	Gujarat	1. Water & Soil Conservation Project includingreclamation of degraded Bhal areas and checking ofsalinity ingress in coastal areas and farm ponds.<br>2. Export oriented clustering and infrastructure in PPPmode for banana<br>3. Large animal surgery at Anand.<br>4. Soil testing lab. operated in PPP mode.
8.	Haryana	1. 1 Providing assistance on laying underground pipelinesystem for water conveyance so that water lossescan be avoided and additional land brought undercultivation from the irrigation channels<br>2. 100% treatment of certified wheat seed<br>3. Cattle health management to optimize fertility
9.	Himachal Pradesh	1. Construction of low cost greenhouses/polyhouses<br>2. Organic cultivation<br>3. Micro/minor irrigation and water harvesting
10.	Jammu & Kashmir	1. Protected cultivation of flowers & vegetables<br>2. Creation of irrigation infrastructure for Horticulture<br>3. Organic farming
11.	Jharkhand	1. Micro Lift Irrigation<br>2. Vermi-composting
12.	Karnataka	1. Karnataka Seed Mission project<br>2. Automated weather monitoring system<br>3. Rainfed agriculture – Bhoo-chetana<br>4. E-tendering project
13.	Kerala	1. Paddy cultivation in fallow lands by leasing land toPadasekaramSamities<br>2. Farm mechanisation for paddy harvesting, includescustom hiring of farm machinery by KAICO and givingagricultural machinery to district Panchayats andPadasekarams for collective use.<br>3. Development of fishery seed farms, seed productionand group mobilization

Sl. No.	Name of the State	Name of the Project
14.	Madhya Pradesh	1. Micro irrigation schemes - Percolation tanks, minorirrigation tanks<br>2. Seed replacement<br>3. 3 Development and promotion of hybrid rice varieties<br>4. 4 Kisan Call Centers
15.	Maharashtra	1. Construction of farm ponds<br>2. Creation of low cost onion storage structures<br>3. Surveillance and Monitoring of Pest and Disease onsoybean and cotton<br>4. AI delivery system
16.	Manipur	
17.	Meghalaya	1. Water conservation structures mini irrigation checkdams<br>2. Horticulture hubs<br>3. Fisheries
18.	Mizoram	
19.	Nagaland	
20.	Odisha	1. Development of 100 new watersheds<br>2. Acidic soil treatment<br>3. Renovation of horticulture farms
21.	Punjab	1. Animal insurance<br>2. Conservation of irrigation water
22.	Rajasthan	1. PPP for innovation in olive, date palm and jojoba<br>2. Operation golden rays PPP for livelihood of 7.5 lakh families through hybrid maize<br>3. DiggiProgramme
23.	Sikkim	1. Hi-tech green houses<br>2. Integrated farming system for sustainable agriculture
24.	Tamil Nadu	1. Precision Farming sugarcane, banana<br>2. Automated weather stations in 224 Blocks<br>3. Promoting SRI through conduct of demonstrations in 40,000 ha<br>4. Use of Paddy transplanters in outsourced mode
25.	Telangana	
26.	Tripura	1. SRI<br>2. Pineapple cultivation<br>3. Development of demonstration unit on piggery
27.	Uttar Pradesh	1. Production of high value vegetable crops through nursery production in low tunnel Polyhouses<br>2. Land Reclamation of Katri Area<br>3. Adoption & certification of Organic Agriculture Management System<br>4. Saghan Mini Dairy Yojna
28.	Uttarakhand	1. Strengthening organic vision
29.	West Begnal	1. PraniBandhu Scheme<br>2. Hybrid paddy seed production involving NGOs<br>3. Implement hub at block level

Annexure 13.3

Statement Showing allocation release and expenditure of the State under RKVY during the XIth plan as on 31.12.2016

(Rs. in Cr ore)

Sl. No	Name of the State/UT	2007-08				2008-09				2009-10				2010-11				2011-12			
		Allocation	Release	UC Recvd	UC Pending	Allocation	Release	UC Recvd	UC Pending	Allocation	Release	UC Recvd	UC Pending	Allocation	Release	UC Recvd	UC Pending	Allocation	Release	UC Recvd	UC Pending
1	Andhra Pradesh	93.13	61.08	61.08	0.00	316.57	297.17	297.17	0.00	410.00	410.00	410.00	0.00	★393.45	432.29	432.29	0.00	727.74	734.20	734.20	0.00
2	Arunachal Pradesh	2.85	1.90	1.90	0.00	6.88	0.00	0.00	0.00	16.10	15.98	15.98	0.00	39.08	28.95	28.95	0.00	8.26	10.68	10.68	0.00
3	Assam	23.77	0.00		0.00	142.62	144.12	144.12	0.00	79.86	79.86	79.86	0.00	★256.87	216.87	216.87	0.00	227.77	227.77	227.77	0.00
4	Bihar	64.02	57.77	57.77	0.00	148.54	148.54	148.54	0.00	110.79	110.79	110.79	0.00	380.94	415.10	415.10	0.00	506.82	506.82	506.82	0.00
5	Chhatisgarh	60.54	52.96	52.96	0.00	116.48	117.45	117.45	0.00	131.78	136.14	136.14	0.00	461.00	503.42	503.42	0.00	230.57	212.61	212.61	0.00
6	Goa	2.29	1.70	1.70	0.00	6.91	0.00	0.00	0.00	11.87	0.00		0.00	11.31	7.07	7.07	0.00	49.55	24.78	24.78	0.00
7	Gujarat	53.71	49.81	49.81	0.00	243.39	243.39	243.39	0.00	386.19	386.19	386.19	0.00	353.45	388.63	388.63	0.00	515.48	515.48	515.48	0.00
8	Haryana	23.12	21.52	21.52	0.00	74.00	39.50	39.50	0.00	112.77	112.77	112.77	0.00	204.74	226.80	226.80	0.00	168.92	176.87	176.57	0.30
9	Himachal Pradesh	17.39	16.17	16.17	0.00	15.11	15.11	15.11	0.00	33.02	33.03	33.03	0.00	94.85	94.85	94.85	0.00	99.93	99.93	99.93	0.00
10	Jammu & Kashmir	6.85	0.00		0.00	16.17	1.20	1.20	0.00	42.05	42.85	42.85	0.00	★162.16	96.42	96.41	0.01	103.03	63.03	59.28	3.75
11	Jharkhand	61.66	55.68	55.68	0.00	58.62	29.31	29.31	0.00	70.13	70.13	70.13	0.00	160.96	96.90	96.90	0.00	168.56	174.56	174.56	0.00
12	Karnataka	171.97	154.30	154.30	0.00	316.57	314.14	314.14	0.00	410.00	410.00	410.00	0.00	284.03	284.03	284.03	0.00	595.90	595.90	595.90	0.00
13	Kerala	61.41	55.40	55.40	0.00	60.11	30.06	30.06	0.00	110.92	110.92	110.92	0.00	192.35	149.65	149.65	0.00	173.93	182.89	182.45	0.44
14	Madhya Pradesh	110.01	101.62	101.62	0.00	146.05	146.05	146.05	0.00	247.44	247.44	247.44	0.00	589.09	559.18	559.18	0.00	398.37	398.37	398.37	0.00
15	Maharashtra	142.20	128.20	128.20	0.00	269.63	261.77	261.77	0.00	407.24	404.39	404.39	0.00	653.00	653.00	653.00	0.00	727.67	735.44	735.44	0.00
16	Manipur	1.35	0.00		0.00	4.14	0.90	0.90	0.00	5.86	5.86	5.86	0.00	24.81	15.50	15.50	0.00	22.25	22.25	22.25	0.00
17	Meghalaya	7.00	6.37	6.37	0.00	13.53	6.77	6.77	0.00	24.68	24.68	24.68	0.00	46.12	46.12	46.12	0.00	14.66	20.44	20.44	0.00
18	Mizoram	1.05	0.00		0.00	4.29	0.80	0.80	0.00	4.15	0.00		0.00	7.49	3.75	3.75	0.00	34.61	36.63	36.63	0.00
19	Nagaland	9.45	3.19	3.19	0.00	13.89	6.95	6.95	0.00	20.38	20.38	20.38	0.00	13.24	13.25	13.25	0.00	37.54	37.54	37.54	0.00
20	Orissa	46.59	39.30	39.30	0.00	115.44	115.44	115.44	0.00	121.49	121.49	121.49	0.00	274.40	274.40	274.40	0.00	356.96	356.96	356.96	0.00
21	Punjab	39.85	36.05	36.05	0.00	87.52	87.52	87.52	0.00	43.23	43.23	43.23	0.00	179.12	179.12	179.12	0.00	138.87	145.87	145.87	0.00
22	Rajasthan	71.68	55.76	55.76	0.00	233.75	233.76	233.76	0.00	186.12	186.12	186.12	0.00	572.47	628.01	628.01	0.00	685.04	692.08	692.08	0.00
23	Sikkim	2.77	2.77	2.77	0.00	11.37	5.68	5.68	0.00	15.29	15.29	15.29	0.00	6.56	6.56	6.56	0.00	20.08	24.64	24.64	0.00
24	Tamil Nadu	188.21	153.60	153.60	0.00	140.38	140.38	140.38	0.00	127.90	127.90	127.90	0.00	225.71	250.03	250.03	0.00	333.06	333.06	333.06	0.00
25	Tripura	4.69	4.16	4.16	0.00	34.02	16.08	16.08	0.00	31.28	31.28	31.28	0.00	116.86	116.48	116.48	0.00	17.99	25.63	25.63	0.00
26	Uttar Pradesh	116.15	103.90	103.90	0.00	316.57	316.57	316.57	0.00	390.97	390.97	390.97	0.00	635.92	695.36	695.36	0.00	757.26	762.83	762.83	0.00
27	Uttarakhand	30.54	28.25	28.25	0.00	20.60	10.30	10.30	0.00	71.36	71.46	71.46	0.00	2.61	1.31	1.31	0.00	131.77	128.84	128.82	0.02
28	West Bengal	60.87	54.93	54.93	0.00	147.38	147.38	147.38	0.00	147.38	147.38	147.38	0.00	476.15	335.98	335.98	0.00	476.65	486.65	486.65	0.00
	**Total States**	**1475.12**	**1246.39**	**1246.39**	**0.00**	**3080.53**	**2876.34**	**2876.34**	**0.00**	**3770.25**	**3756.53**	**3756.53**	**0.00**	**6662.00**	**6719.03**	**6719.02**	**0.01**	**7729.24**	**7732.75**	**7728.24**	**4.51**

Sl. No	Name of the State/UT	2007-08				2008-09				2009-10				2010-11				2011-12			
		Allocation	Release	UC Recvd	UC Pending	Allocation	Release	UC Recvd	UC Pending	Allocation	Release	UC Recvd	UC Pending	Allocation	Release	UC Recvd	UC Pending	Allocation	Release	UC Recvd	UC Pending
29	A & N Islands	9.52			0.00	6.43	2.26	0.01	2.25	12.21	1.28		1.28	Being done by MHA							
30	Chandigarh	0.22			0.00	2.20	0.14	0.00	0.14	3.70	0.42		0.42								
31	D& N Haveli	0.25			0.00	0.61	0.00		0.00	0.29			0.00								
32	Daman & Diu				0.00	1.42	0.26		0.26	0.30			0.00								
33	Delhi	0.56	0.10		0.10	1.83	0.00		0.00	2.36	0.24		0.24								
34	Lakshadweep	0.92			0.00	12.08	6.14	6.04	0.10	10.12	1.09		1.09								
35	Pudducherry	3.13	0.40	0.40	0.00	6.67	0.00		0.00	0.69	0.00		0.00								
	**Total UTs**	**14.58**	**0.50**	**0.40**	**0.10**	**31.24**	**8.80**	**6.05**	**2.75**	**29.67**	**3.03**	**0.00**	**3.03**								
	District Agri Plan					53.90				6.82			0.00								
	NIRD, ISEC, IEG, IIM-CMA, & Admn Contingency						1.66				1.37		0.00	60.00	1.03			81.63	61.34		
	**Grand Total**	**1489.70**	**1246.89**	**1246.79**	**0.10**	**3165.67**	**2886.80**	**2882.39**	**2.75**	**3806.74**	**3760.93**	**3756.53**	**3.03**	**6722.00**	**6720.06**	**6719.02**	**0.01**	**7810.87**	**7794.09**	**7728.24**	**4.51**

*** This includes two new sub-Schemes of RKVY viz. (i) Extending Green Revolution to Eastern India & (ii) Special Initiative for Pulses & Oilseeds in Dryland Areas.**

*** Allocation of Rs. 35.00 crore and Release Rs. 17.50 croreGreen Revolution in Estern India for Assam, Rs. 39.44 crore for saffron Mission for J & K & increased allocation of Rs. 82.26 crore in respect of Andhra Pradesh is to be met from savings from overall allocation of Rs. 6755.00 crore.**

Statement showing Allocation, Release and Expenditure of the States Under RKVY during XIIth Plan as on 31.12.2016

(Rs. in Crore)

Sl. No.	Name of the State	2012-13				2013-14				2014-15				2015-16				2016-17			
		Allocation	Release	UC Recvd	UC Pending	Allocation	Release	UC Recvd	UC Pending	Allocation	Release	UC Recvd	UC Pending	Allocation	Release	UC Recvd	UC Pending	Allocation	Release	UC Recvd	UC Pending
1	Andhra Pradesh	601.98	577.79	577.79	0.00	483.23	456.87	456.87	0.00	267.07	263.54	263.54	0.00	197.79	192.66	192.66	0.00	216.83	111.89	67.20	44.69
2	Arunachal Pradesh	40.31	24.94	24.94	0.00	33.04	33.04	33.04	0.00	27.83	13.92	13.92	0.00	31.55	31.55	13.92	17.63	23.82	11.92		11.92
3	Assam	399.57	399.57	399.57	0.00	440.03	218.87	206.47	12.40	483.53	267.71	249.00	18.71	128.07	94.10	78.87	15.23	278.15	137.54		137.54
4	Bihar	724.01	700.20	700.20	0.00	527.67	254.26	254.26	0.00	564.55	545.73	456.08	89.65	164.86	111.20	88.41	22.79	258.22	44.05		44.05
5	Chhatis-garh	581.12	571.22	571.22	0.00	407.63	233.82	233.82	0.00	385.44	341.75	341.75	0.00	150.11	145.07	135.85	9.22	200.30	100.40	70.10	30.30
6	Goa	62.43	35.27	35.27	0.00	21.91	10.43	10.43	0.00	25.63	0.00	0.00	0.00	43.40	21.70	19.59	2.11	20.69	10.35		10.35
7	Gujarat #	586.87	610.87	610.87	0.00	557.04	476.89	476.89	0.00	593.55	290.03	290.03	0.00	182.09	174.55	140.72	33.83	315.27	155.31		155.31
8	Haryana	199.49	179.88	179.62	0.26	318.57	159.29	159.29	0.00	372.03	254.68	243.81	10.87	143.10	141.54	81.09	60.45	152.40	71.91		71.91
9	Himachal Pradesh	73.48	59.27	59.27	0.00	77.40	77.40	77.40	0.00	86.11	86.11	86.11	0.00	28.30	28.30	28.30	0.00	52.76	26.38	17.71	8.67
10	Jammu & Kashmir	112.08	103.22	102.01	1.21	148.04	88.52	86.87	1.65	150.50	78.25	75.37	2.88	74.72	37.36	27.63	9.73	88.90	16.16		16.16
11	Jharkhand	241.55	219.38	216.19	3.19	294.18	147.10	136.95	10.15	306.94	153.15	124.51	28.64	87.68	78.32	61.22	17.10	155.27	72.72	17.25	55.47
12	Karnataka	586.52	549.15	549.15	0.00	794.58	467.29	466.53	0.76	884.17	632.22	632.22	0.00	359.94	346.93	340.34	6.59	236.96	204.43	110.69	93.74
13	Kerala	282.26	253.03	252.69	0.34	270.78	256.24	255.64	0.60	321.40	300.70	299.39	1.31	91.86	90.48	89.31	1.17	157.71	78.86		78.86
14	Madhya Pradesh	448.13	448.13	448.13	0.00	545.15	276.25	276.25	0.00	547.55	511.78	511.78	0.00	264.57	264.09	239.98	24.11	454.89	224.56	74.02	150.54
15	Maharash-tra	1025.81	1050.81	1050.81	0.00	1154.93	959.69	959.69	0.00	1013.49	942.09	942.09	0.00	438.11	438.00	308.98	129.02	396.29	200.24	24.00	176.24
16	Manipur	52.94	47.97	47.97	0.00	40.96	23.66	23.66	0.00	43.00	43.00	43.00	0.00	20.46	20.46	6.91	13.55	29.77	11.86	11.86	0.00
17	Meghalaya	105.34	22.68	22.68	0.00	60.89	37.98	37.95	0.03	68.77	60.64	60.64	0.00	16.52	8.26	8.26	0.00	20.19	10.14		10.14
18	Mizoram	200.91	184.73	184.73	0.00	132.01	77.41	77.41	0.00	113.92	113.92	113.92	0.00	16.61	19.39	19.39	0.00	25.95	10.80	6.86	3.94
19	Nagaland	85.75	85.75	85.75	0.00	52.59	30.07	30.07	0.00	52.75	52.75	52.75	0.00	25.25	25.25	25.25	0.00	36.94	17.47	15.00	2.47
20	Orissa	503.10	468.28	468.28	0.00	508.42	529.42	529.42	0.00	504.13	482.07	480.89	1.18	299.67	292.36	254.15	38.21	342.59	171.06	96.03	75.03
21	Punjab	146.93	86.83	86.83	0.00	448.24	229.44	229.44	0.00	508.71	413.71	397.53	16.18	215.94	107.97	74.82	33.15	260.50	95.81		95.81
22	Rajasthan	363.09	348.18	348.18	0.00	735.24	735.24	735.24	0.00	740.55	695.28	695.28	0.00	342.47	325.97	325.97	0.00	261.68	198.71	126.63	72.08
23	Sikkim	29.47	15.21	15.21	0.00	20.20	10.20	10.20	0.00	18.98	9.49	9.49	0.00	24.35	4.07	2.93	1.14	14.71	14.40		14.40
24	Tamil Nadu	659.68	613.27	613.27	0.00	301.53	269.96	269.96	0.00	298.95	298.95	298.95	0.00	259.94	259.74	259.74	0.00	147.39	154.87	127.40	27.47
25	Telengana	-	-	-	-	-	-	-	-	195.27	179.64	179.64	0.00	142.30	135.91	135.91	0.00	427.85	78.68		78.68
26	Tripura	56.43	56.43	56.43	0.00	74.28	70.50	70.50	0.00	80.27	80.27	80.27	0.00	21.54	21.54	21.54	0.00	34.41	34.41	11.20	23.21
27	Uttar Pradesh	432.26	294.52	294.52	0.00	746.69	561.09	548.77	12.32	704.93	589.47	583.23	6.24	358.16	177.41	121.40	56.01	321.98	149.36		149.36
28	Uttara-khand	44.36	8.21	8.21	0.00	88.02	44.03	44.03	0.00	95.39	80.70	80.16	0.54	40.03	36.20	30.66	5.54	46.87	24.89	9.30	15.59
29	West Bengal	464.81	374.58	374.58	0.00	508.14	265.08	265.08	0.00	598.61	582.34	582.19	0.15	285.26	284.56	284.56	0.00	352.47	164.98	98.79	66.19
	**Total States**	**9110.68**	**8389.37**	**8384.37**	**5.00**	**9791.39**	**7000.04**	**6962.13**	**37.90**	**9864.02**	**8363.89**	**8187.54**	**176.35**	**4454.65**	**3914.94**	**3418.36**	**496.58**	**5341.80**	**2604.16**	**884.04**	**1720.12**

Sl. No.	Name of the State	2012-13				2013-14				2014-15				2015-16				2016-17			
		Allocation	Release	UC Recvd	UC Pending	Allocation	Release	UC Recvd	UC Pending	Allocation	Release	UC Recvd	UC Pending	Allocation	Release	UC Recvd	UC Pending	Allocation	Release	UC Recvd	UC Pending
30	Delhi	Being Done by MHA												1.50	0.00		0.00	1.50			0.00
31	Puducherry													1.51	0.76		0.76	1.51			0.00
32	Andman & Nicobar													4.70	2.35		2.35	4.69			0.00
33	Chandigarh													0.31	0.00		0.00	0.33			0.00
34	D & N Haveli													5.43	0.00		0.00	5.42			0.00
35	Daman & Diu													0.94	0.00		0.00	0.94			0.00
36	Lakshadweep													0.63	0.00		0.00	0.63			0.00
CRRI, Cuttack BGREI Cell, Centres of Excellence, Monitoring and other administrative Expenditure		106.58	10.63	-	10.63	90.00	52.47	-	52.47	90.00	79.31	-	79.31	30.33	24.34		24.34	54.00	6.14		6.14
**Grand Total**		**9217.00**	**8400.00**	**8384.37**	**15.63**	**9954.02**	**7052.51**	**6962.13**	**90.37**	**9954.02**	**8443.20**	**8187.54**	**255.66**	**4500.00**	**3942.39**	**3418.36**	**524.03**	**5410.82**	**2610.30**	**884.04**	**1726.26**

(2012-13) -: Rs. 7.00Crore has been allocated to NCT of Delhi Under Vegetable Cluster, Rs. 2.5 crore has been allcoated to Puducherry under NMPS. Rs. 100.00 crore additional allocation and release under the AFDP for the Maharashtra, Haryana, Punjab, Karnataka, Gujarat and Tamil Nadu approved by the EGoM. # The exceeded amount i.e. Rs. 26 lakh would be met from DADF Budget 2012-13 from the relevant schemes under fisheries. **(2013-14) -:** Rs.1.00 crore allocation for delhi under the VIUC, Including Allocation of Rs.1.49 crore for Monitoring and Rs. 3.40 crore for N.E. States., Rs. 1.27 crore for Monitoring under INSIMP, Rs. 3.00 crore for Monitoring under the Nutri Farm, Rs. 1.50 crore for Monitoring under the Crop Diversification and Rs. 2.00 crore for Moitioring under the BGREI.,'@ COE, Monitoring, Evaulation, etc. **(2014-15)-:** Rs. 6.00 Crore Allocation for Delhi. # Allocaton adjusted additional fund of Rs. 250.00 crore will be asked at a later stage., Release of funds to Govt. of Bihar under Rashtriya Krishi Vikas Yojana(RKVY) during 2014-15 for adjustment of expenditure during 2013-14., Rs. 100.00 crore under the AFDP will be meet from overall saving of the Normal and Sub-Schemes. (2014-15) -: # Rs.14.72 crore is refund by Gujarat to GOI during 2013-14, as per this is unspent balance is NIL. **(2015-16) -: Note : Including total allocation for UT's Rs.15.02 and Rs.30.33 crore inculding sub-head 2401 under RKVY, Rs. 10.04 crore for Bee Keeping sub-scheme)**

Annexure-18.1

Women in Agriculture at a Glance

Sl. No	Divisions/Subject Matter Area	Scheme/Component	Flow of benefit to women in schemes/programmes
1.	**Horticulture**	Horticulture Mission for North East & Himalayan States (HMNEH), National Bamboo Mission (NBM), Central Sector Scheme of National Horticulture Board (NHB), Coconut Development Board (CDB) and Central Institute for Horticulture (CIH), Nagaland.	Implementing agencies viz. State Horticulture Missions have been directed to ensure that at least 30% of budget allocation is earmarked for women beneficiaries/farmers. Women beneficiaries such as women farmers, women Self Help Groups, women entrepreneurs are encouraged to obtain the benefits of the schemes under the Mission.
2.	**Agriculture Extension**	Support to States for Extension Reforms	• At least 30% scheme beneficiaries should be women farmers/ farm women.<br>• Minimum 30% of resources meant for programmes and activities are required to be allocated to women farmers and women extension functionaries with specific documentation of expenditure and performance for women being maintained;<br>• Farm *Women's Food and Nutritional Security Groups* (FIGs) @ at least 3 women FSGs/block to be formed annually for ensuring food and nutritional security providing assistance of Rs. 10,000/- per group.<br>• Inclusion of one *'Gender Coordinator'* in every State in the team of committed extension personnel being supported under the Scheme. The role of Gender Coordinator is to ensure flow of support viz. training/ capacity building and extension support as per the specific requirements of women farmers through a strategy suited to their needs<br>• At least 30% scheme beneficiaries are to be women farmers/ farm women;<br>• Representation of Women farmers in different decision making bodies at State, District and Block level such as State Farmers Advisory Committee (SFAC) at State Level; Agriculture Technology Management Agency(ATMA) Governing Board, ATMA Management Committee(MC) and District Farmer Advisory Committee (DFAC) at district level and Block Farmer Advisory Committee (BFAC) at Block Level<br>• Preferential involvement of women as 'Farmer Friends' under the extension delivery mechanism below the block level (@1Farmer Friend/2 Villages)<br>Since inception of the scheme in 2005-06, total 9,63,7720 farm women (26.60% of the total benefited farmers) have participated in farmer oriented activities like Exposure Visits, Training, Demonstrations &KisanMelas including 6,31,572 women farmers benefited during 2016-17 (up to 30th November, 2016).
3.		National Gender Resource Centre in Agriculture (NGRCA)	The Centre acts as a focal point to converge of all gender related activities & issues in agriculture & allied sectors within and outside the Department of Agriculture, Cooperation & Farmers Welfare, add gender dimension to agriculture policies &programmes, render advocacy / advisory services to the States/ UTs to internalize gender specific interventions and ensure that the policies and programmes in agriculture are fully engendered & reflect the national commitment to empowerment of women.

Sl. No	Divisions/Subject Matter Area	Scheme/Component	Flow of benefit to women in schemes/programmes
4.		Establishment of Agri-Clinics & Agri-Business Centres (ACABC)	The AC&ABC scheme has been revised during 2010-11 with changes in relevant operational aspects aiming to provided better services to farmers, improvements in the quality of training and simplify the process of subsidy disbursement and provision of extension service to farmers by these agri-preneurs has been made a mandatory component of the said Scheme.<br>The subsidy is 44% in respect of women, SC/ST & all categories of the candidates from North-Eastern and Hill States and 36% in respect of other categories.<br>During the current year 1930 candidates were trained and 561 have established their ventures of which **188** and **29** are women. Since inception of the scheme, **49745** candidates have been trained and **21010** agri-ventures have been established in the country till **31.10.2016.** Out of these **3706** and **1150** are women.
5.	**Crops**	National Food Security Mission (NFSM)	30% of budgetary allocation is earmarked for women beneficiaries/ farmers. Concerned Implementing Agencies are responsible for monitoring implementation of these components, i.e., allocation of resources for SC/ST/Women beneficiaries.
6.	**Agricultural Marketing**	Integrated Scheme for Agricultural Marketing (ISAM)	Women under AMI are eligible for subsidy @ 33.33% as against 25% for others.
7.	**Mechanization and Technology**	Sub Mission on Agricultural Mechanization (SMAM)	• State Governments have been advised to ensure 30% of allocation under the scheme belongs to women beneficiary and also to furnish reports separately in SMAM guidelines.<br>• 10% more assistance for women beneficiary to procure Agricultural Machinery, implements and equipments including PHT under component 2 and 3.<br>• In order to reduce the drudgery and increasing efficiency in farm operations, a number of agricultural implements and hand tools suitable for farm women have been developed by Research & Development organizations under ICAR. The list of gender friendly equipments has been sent to all States/UTs for popularizing them through various schemes of Government.<br>**Gender Friendly Equipment for Women**: Under the component 1 of SMAM, Agricultural Mechanization through Training, Testing, and Demonstration, a total 1949 women were trained during the current Financial Year 2016-17, (till October. 2016) .During the current Financial Year 2016-17 (till October. 2016) , 1949 women trainees have been trained at FMTTIs.
8.	**Seeds**	Sub-Mission for Seed and Planting Material (SMSP)	States/Implementing agencies are requested to allocate adequate funds/give preference to women farmers.
9.	**Cooperation**	National Cooperative Development Corporation	NCDC encourages women cooperatives to avail assistance under its various schemes. Cumulatively, as on 31.03.2016 NCDC has sanctioned and released financial assistance of Rs. 193.54 crores and Rs. 96.41 crores respectively for the development of cooperative societies exclusively promoted by women. During the year 2015-16, NCDC sanctioned RS. 2.04 crores to 75 Unit under Consumer and ICDP programmes and released Rs. 3.55 crores to women co-operative societies under ICDP programme. In the 2815 projects/ units sanctioned by NCDC in the year 2015-16, 11.98 lakh women are enrolled as members, out of which 369 women members are on the Board of Directors of different Cooperatives.

*****

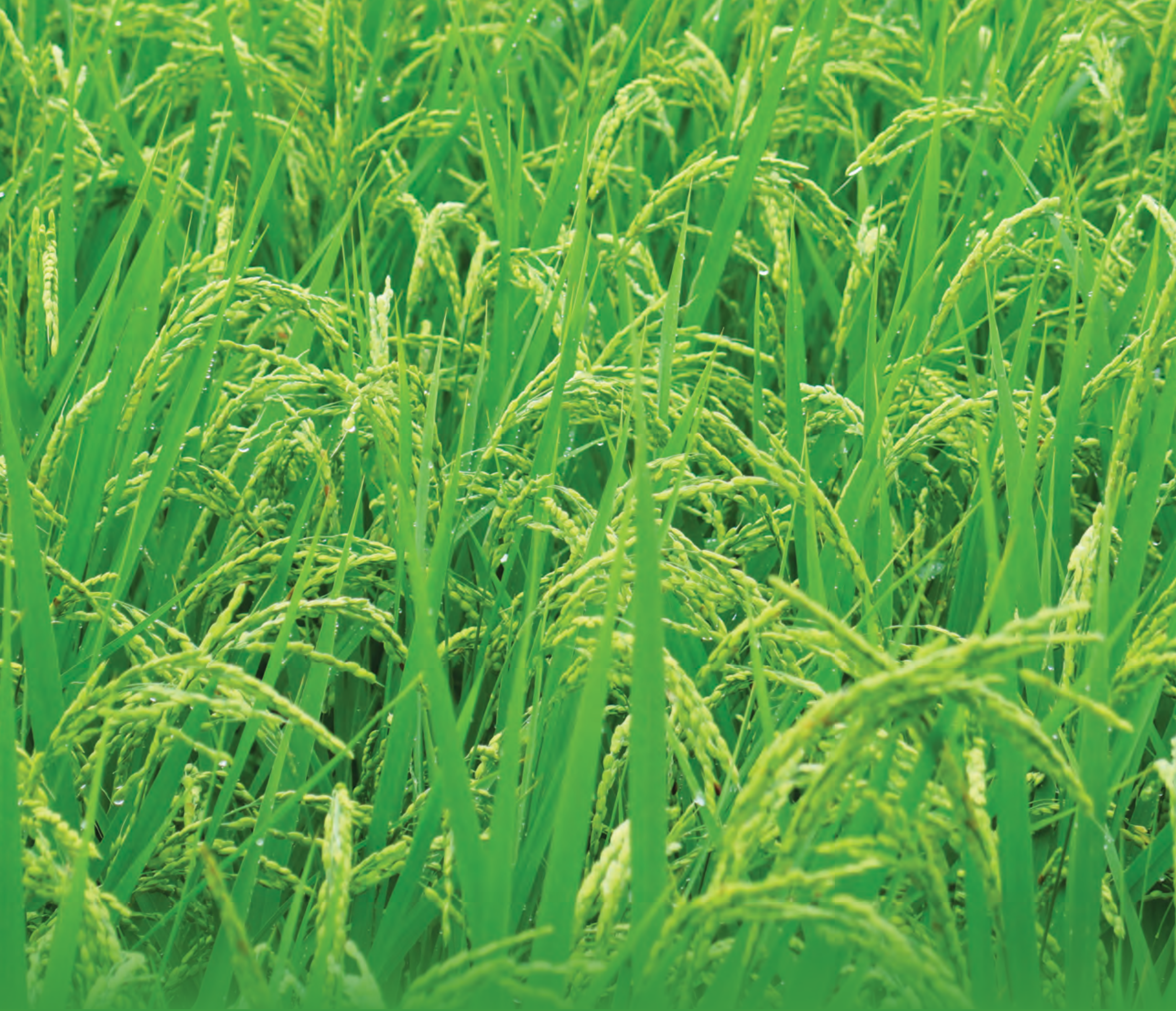

www.agricoop.nic.in

Design & Printed to: Chandu Press - 9810519841
